# Sharp Increase of Problematic Mitogenomes of Birds: Causes, Consequences, and Remedies

**DOI:** 10.1093/gbe/evab210

**Published:** 2021-09-10

**Authors:** George Sangster, Jolanda A Luksenburg

**Affiliations:** 1 Department of Bioinformatics and Genetics, Swedish Museum of Natural History, Stockholm, Sweden; 2 Naturalis Biodiversity Center, Leiden, The Netherlands; 3 Institute of Environmental Sciences, Leiden University, The Netherlands; 4 Department of Environmental Science and Policy, George Mason University, Fairfax, Virginia, USA

**Keywords:** chimera, misidentification, sequencing errors, numts, sequence artifacts

## Abstract

Authentic DNA sequences are crucial for reliable evolutionary inference. Concerns about the identification of DNA sequences have been voiced several times in the past but few quantitative studies exist. Mitogenomes play important roles in phylogenetics, phylogeography, population genetics, and DNA identification. However, the large number of mitogenomes being published routinely, often in brief data papers, has raised questions about their authenticity. In this study, we quantify problematic mitogenomes of birds and their reusage in other papers. Of 1,876 complete or partial mitogenomes of birds published until January 1, 2020, the authenticity of 1,559 could be assessed with sequences of conspecifics. Of these, 78 (5.0%) were found to be problematic, including 45 curated reference sequences. Problems were due to misidentification (33), chimeras of two or three species (23), sequencing errors/numts (18), incorrect sequence assembly (1), mislabeling at GenBank but not in the final paper (2), or vice versa (1). The number of problematic mitogenomes has increased sharply since 2012. Worryingly, these problematic sequences have been reused 436 times in other papers, including 385 times in phylogenies. No less than 53% of all mitogenomic phylogenies/networks published until January 1, 2020 included at least one problematic mitogenome. Problematic mitogenomes have resulted in incorrect phylogenetic hypotheses and proposals for unwarranted taxonomic revision, and may have compromised comparative analyses and measurements of divergence times. Our results indicate that a major upgrade of quality control measures is warranted. We propose a comprehensive set of measures that may serve as a new standard for publishing mitogenome sequences.

**Significance**

The manuscript demonstrates for the first time that a substantial portion of mitochondrial genomes of birds are unreliable and that this is a widespread and rapidly growing problem for systematics, evolutionary biology, and ecology. Because problematic sequences were found to originate from incomplete quality checks during the entire process from the initial identification of specimens to archiving sequences at GenBank, we propose a comprehensive set of measures that will help prevent the publication and reusage of such sequences. This set of measures may serve as a standard for the 1,000s of data papers describing new mitogenomes that are being published each year.

## Introduction

Authentic DNA sequences are crucial for accurate evolutionary inference. Misidentified, chimeric, and nonhomologous sequences may compromise phylogenetic analysis and other downstream applications, including taxonomy, comparative analysis, and DNA identification of other sequences. Misidentifications, nuclear copies of mitochondrial sequence fragments (numts), and PCR artifacts are long-known risks to DNA studies (Clark and Whittam 1992; Arctander 1995; Helbig and Seibold 1996; Ruedas et al. 2000). More recently, several cases of chimeric sequences have been documented (Zuccon and Ericson 2010; Moyle et al. 2013; Norén and Kullander 2018; Sangster and Luksenburg 2020). However, little is known about the causes of problematic animal DNA sequences in public DNA databases (but see Steinegger and Salzberg 2020) and even less is known about the consequences of such sequences when they are reused by other publications.

Past efforts to quantify erroneous sequences have focused on misidentifications and typically used a single mitochondrial marker and a single criterion for diagnosis, such as the phylogenetic position of a sequence or the level of divergence between sequences (e.g., Ross and Murugan 2006; de Mendonça et al. 2011; Shen et al. 2013; Lis et al. 2016; Strohm et al. 2016; Li et al. 2018; but see Botero-Castro et al. 2016; Nacer and do Amaral 2017). Although such studies are able to show that “potential” errors exist, their methods are usually not sophisticated enough to infer a reliable diagnosis. For instance, a sequence phylogenetically misplaced in a gene tree based on a single marker may represent a misidentification but without evidence from other markers it could also represent a chimera (a sequence composed of fragments from two or more individuals concatenated into a single sequence). Similarly, a sequence divergence exceeding a predefined cutoff value (sometimes set as low as 1%) may be due to artifacts such as misidentification, inaccurate taxonomy, contamination, and errors in sequencing, but may also represent intraspecific variability. The quantitative studies conducted so far did not attempt to differentiate among these factors. Nevertheless, these studies indicate that problems exist, which should be addressed by molecular biologists. Indeed, several authors have made recommendations to improve the reliability and documentation of DNA sequences (e.g., Ruedas et al. 2000; Harris 2003; Morin et al. 2010; Botero-Castro et al. 2016; Lis et al. 2016; Strohm et al. 2016).

Complete mitochondrial genomes (hereafter mitogenomes) have become an important source of information for studies of phylogenetics, phylogeography, molecular evolution, speciation, and demography (Saitoh et al 2006; Rohland et al. 2007; Shen et al. 2009; Subramanian et al. 2009; Morin et al. 2010; Bjork et al. 2011; Lerner et al. 2011; Schönberg et al. 2011; Stager et al. 2014; Morales et al. 2015; Nabholz et al. 2016; Rollins et al. 2016; Stryjewski and Sorenson 2017; Urantówka et al. 2018). Of all types of DNA sequences, mitogenomes are perhaps the most likely to be reused in other studies due to the dominance of mitochondrial markers in phylogenetics, phylogeography, and population genetics.

In recent years, large numbers of mitogenomes are being published annually. In 2019, the journal *Mitochondrial DNAPartB* published 1,921 mitogenome data papers. Typically, a data paper includes a single sequence, along with various descriptive data and a mitogenome phylogeny. The purpose of such papers is to announce the first mitogenome of a species or population, which means that there is typically no other mitogenome of the species available to verify its quality and authenticity. However, for such sequences correct identification and sequence integrity are particularly important because the sequences are intended to serve as reference mitogenome for species.

In this study, we analyze complete or partial mitogenome sequences of birds. Birds are a suitable group for assessments of sequence quality and authenticity because birds are taxonomically relatively mature, there are large numbers of reference sequences, and identification of specimens in the hand or in a museum is usually straightforward. We use markers based on three protein-coding genes (PCGs) for which sufficient numbers of reference sequences are available. We quantify the prevalence of problematic sequences, diagnose the causes of the problems, and assess the reusage of problematic sequences. We use our results to propose a series of recommendations which aim to detect and prevent inevitable human errors before publication or reusage.

## Results

### Problematic Mitogenomes

A total of 1,876 avian mitogenomes were analyzed of which 1,559 could be compared with one to three markers of conspecifics (supplementary appendix S1, Supplementary Material online). Of these, 889 were assessed with 3 markers, 513 were assessed with 2 markers, and 157 were assessed with 1 marker. A total of 317 mitogenomes could not be assessed for various reasons, including a lack of *COI*, *cytochrome b*, and *ND2* reference sequences on GenBank, insufficient phylogenetic structure (resolution) among species, absence of *COI*, *cytochrome b*, and *ND2* fragments in the mitogenome sequence, unavailability of the mitogenome sequence on GenBank (and no known GenBank accession number), or doubts about the identity of the reference sequence(s) on GenBank.

A total of 78 mitogenomes (5.0%; 78/1559) representing 74 species were found to be problematic (Appendix A, supplementary table S1, Supplementary Material online). Detailed accounts of these mitogenomes are given in supplementary appendix S2, Supplementary Material online, and gene trees are given in supplementary figures S1−S64, Supplementary Material online. In most cases (72%), the problematic sequence was the only available mitogenome of the relevant species. Problematic mitogenomes were found in 13 taxonomic orders (37 families) across the bird phylogeny. Problematic mitogenomes were produced by 42 research groups in 8 countries: China (25 groups, 53 mitogenomes), the United States (7 groups; 8 mitogenomes), South Korea (2 groups, 6 mitogenomes), Australia (2 groups; 6 mitogenomes), Japan (2 groups; 2 mitogenomes), Brazil (1 group, 1 mitogenome), New Zealand (1 group, 1 mitogenome), and the United Kingdom (1 group, 1 mitogenome).

Unpublished and published mitogenomes did not differ significantly in the proportion of problematic mitogenomes (5.3% vs. 4.9%; chi-squared = 0.068; *P* = 0.79).

### Causes of Problematic Mitogenomes

All six distinct problems with mitogenomic sequences were found (supplementary table S1, Supplementary Material online). Misidentification was the most common cause (*n* = 33), followed by chimeras of mitochondrial DNA of two or three species (23), sequencing errors/numts (18), incorrect sequence assembly (1), mislabeling at GenBank but not in the final paper (2), or vice versa (1).

The 33 mitogenome sequences that were wrongly identified belonged to species that were not necessarily closely related. In 21 cases, the misidentified species were classified in the same genus. In seven cases, the species were from different genera in the same family. In one case, the misidentified species was a member of a different family in same order, and in another case the misidentified species was a member of a different order. The latter was a mitogenome sequence of a shearwater (*Calonectris leucomelas*, Procellariiformes) that was published on GenBank as that of a gull (*Larus vegae*, Charadriiformes).

Of all 33 misidentifications, 31 were detected using 2 or 3 mitochondrial markers. In two cases of misidentification, only one marker was available to test the identity of the relevant mitogenome (MH229988, “*Rallus aquaticus*”); MF435900, “*Anthracoceros coronatus*”). However, in both cases, the true identity of the species was also revealed by the collecting site, which was outside the range of the stated species and inside that of another species (i.e., *Rallus indicus* and *Anthracoceros albirostris*, respectively).

In all 23 chimeric sequences, 1 or more DNA fragments were included that belonged to other species that were classified in a different genus. Five of these were also classified in different families in the same order, six were classified in different orders, and one was classified in a different class (supplementary table S1, Supplementary Material online). The proportion of heterospecific DNA included in chimeric mitogenomes varied from at least 2.0% to at least 98.8% (supplementary table S3, Supplementary Material online). In four cases, it was unclear if the mitogenome included any authentic DNA fragments of the purported species: KJ192198 (*Amaurornis akool*), KT340631 (*Otus bakkamoena*), KM272749 (*Caprimulgus jotaka*), and HM535648 (*Pseudopodoces humilis*). In these cases, the source specimen was likely misidentified. For several chimeric mitogenomes, the proportion of heterospecific DNA could not be assessed due to a lack of authentic mitogenomes of the relevant species. Heterospecific fragments in chimeras differed from authentic sequences by 7.5−23.3% (supplementary table S3, Supplementary Material online). The (minimum) number of heterospecific fragments included in chimeric mitogenomes ranged from one to ten (supplementary table S3, Supplementary Material online). The length of contiguous heterospecific DNA fragments ranged from 70 to 11,911 bp (mean 1,204 bp; *n* = 58; supplementary table S3, Supplementary Material online).

Chimeric mitogenomes were detected in various ways (supplementary table S3 and appendix S1, Supplementary Material online). In five cases, there was a mismatch between gene trees in the position of the mitogenome. Six chimeras were revealed by a long branch in a gene tree. Three chimeric mitogenomes showed a deep divergence in a gene tree. In several cases, there were multiple indicators, including a mismatch between gene trees and the lack of a close match with any other species in one or more gene trees; a mismatch between gene trees and a long branch; and a long branch, no close match with any other species in one gene tree, and a long branch of its sister clade in another gene tree.

The use of multiple markers increased the number of chimeras detected. If *COI* were the only marker used in this study, only seven of the 23 chimeras would have been diagnosed as such, and seven would have been classified as a misidentification. Similarly, if *cytochromeb* were the only marker used, only ten mitogenomes would have been identifiable as a chimera and six would have been wrongly diagnosed as misidentifications. If *ND2* were the only marker used to detect chimeras, only 4 of the 23 chimeras would have been correctly diagnosed and 5 mitogenomes would have been wrongly classified as a misidentification. Thus, using single markers, only four to ten mitogenomes (17−43%) would have been correctly identified as chimeras.

At least 18 mitogenomes included sequencing errors or numts. These problems were detected in various ways (supplementary table S4 and appendix S1, Supplementary Material online), including a long branch in one or more gene trees, a deep divergence from other members of the species in one or more gene trees (sometimes in a distant position without any close match), multiple inferred insertions and/or deletions in an alignment of PCG sequences, or combinations of these.

The use of multiple markers also increased the number of mitogenomes with sequencing errors or numts being detected. If *COI* were the only marker used in this study, 10 of the 18 mitogenomes with sequencing errors or numts would have been diagnosed as such. If *cytochromeb* were the only marker used, 15 of such mitogenomes would have been detected, and if *ND2* were the only marker used, only 8 of the 18 mitogenomes with sequencing errors or numts would have been detected. Thus, using single markers, only 8−15 mitogenomes with sequencing errors or numts (44−83%) would have been identified as such.

One mitogenome sequence showed clear evidence of incorrect sequence assembly that resulted in partial duplication of *cytochromeb* (*Branta bernicla*). The problematic nature of this sequence was first noted due to a long branch in the *cytochromeb* gene tree (supplementary fig. S4, Supplementary Material online).

Two mitogenomes were found to be mislabeled on GenBank but not in the final paper (*Tadorna tadorna*, supplementary fig. S6, Supplementary Material online; *Agapornis pullarius*, supplementary fig. S37, Supplementary Material online). These errors were detected due to differences between the names used on GenBank and those used in the relevant papers (Lin et al. 2016; Chen et al. 2019). Gene trees showed that the names in the papers were correct.

One mitogenome was found to be mislabeled in the final paper but not on GenBank (*Phasianus colchicus*, supplementary fig. S3, Supplementary Material online). Again, this was detected from a difference between the name used on GenBank and that in the relevant paper (Zhu et al. 2017). Here, however, the gene trees showed that the name on GenBank was correct.

### Consequences of Problematic Mitogenomes

**Fig. 1:**
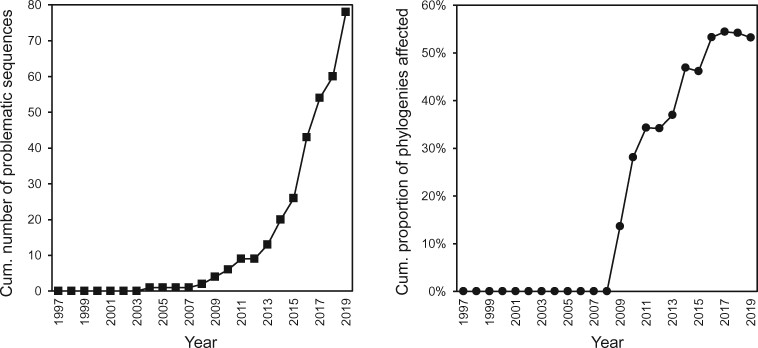
(*a*) Cumulative number of problematic avian mitogenomes published until January 1, 2020 (*n* = 78). (*b*) Cumulative proportion of avian mitogenomic phylogenies (*n* = 344) that included at least one problematic sequence (*n* = 183). Note the rapid increase of problematic mitogenomes after 2012 and their effect on phylogenetics.

Problematic sequences have been reused 436 times in other papers, including 385 times in phylogenies (fig. 1; supplementary table S5, Supplementary Material online). The first phylogeny “contaminated” by a problematic mitogenomic sequence was published in 2009. By January 1, 2020, 53% (183/344) of all published avian mitogenomic phylogenies/networks included at least one problematic mitogenome. In some cases, a phylogeny or analysis included multiple problematic mitogenomic sequences (e.g., 22 in Nabholz et al. 2016; 18 in Mackiewicz et al. 2019; supplementary appendix S1, Supplementary Material online).

Problematic sequences have been used to address at least eight types of research questions (supplementary table S5, Supplementary Material online). 1) In at least one case, an incorrect phylogenetic hypothesis was advanced: The nonmonophyly of a genus of owls (*Bubo*) based on a mitogenome with sequence errors/numts. 2) Several proposals for incorrect taxonomic revisions have been made based on problematic mitogenomes, including the split of the owl genus *Bubo*, the rejection of a recent revision of genus limits of rails (*Porzana*, *Zapornia*), and the transfer of the titmice (Paridae) from Sylvioidea to a different superfamily. 3) A chimeric mitogenome has led some authors to discredit correct mitochondrial sequences. 4) Problematic mitogenomes have been used to infer divergence times, which may have led to overestimation of divergence times due to inflated levels of “substitutions” in chimeras and mitogenomes with sequencing errors/numts), and underestimation of divergence times due to misidentification. 5) A misidentified mitogenome and a mitogenome with sequence errors or numts have been used as reference sequences for DNA identification. 6) A comparative analysis of the effects of body size on the substitution rate of mitochondrial genomes included no less than 22 problematic mitogenomes, comprising nine misidentified sequences, eight chimeras and five mitogenomes with sequence errors/numts (Nabholz et al. 2016). 7) A comparative study of mitochondrial gene order in passerines included 18 problematic mitogenomes, comprising 10 misidentified sequences, 6 chimeras, and 2 mitogenomes with sequence errors/numts (Mackiewicz et al. 2019). 8) Finally, chimeras and mitogenomes with sequence errors/numts have been used to assemble new mitogenomes from NGS sequence fragments.

### Vigilance and Quality Control

The majority of problematic mitogenomes (*n* = 62) were published in peer-reviewed journals. Two other problematic mitogenomes were first described in a prepublication (PeerJ Preprints), and 14 problematic mitogenomes sequences were unpublished at the time of writing but available on GenBank (supplementary table S1, Supplementary Material online). Two further problematic mitogenomes were correctly published in data papers, but the sequences were incorrectly labeled on GenBank.

A total of 45 problematic mitogenomes have been entered into the RefSeq database (recognizable by their NC_ prefix). These comprise 18 misidentified mitogenomes, 16 chimeras, 9 mitogenomes with sequencing errors/numts, and 2 mitogenomes that are mislabeled on GenBank. One of these RefSeq records (NC_021970, *Strix leptogrammica*) was removed by the time of writing.

Problematic mitogenomes were published in 22 peer-reviewed journals (supplementary table S1, Supplementary Material online). Most of these were published as data papers in *Mitochondrial DNA Part B* (*n* = 19) and *Mitochondrial DNA* and its successor *Mitochondrial DNA Part A* (*n* = 18). Other problematic mitogenomes were published in *Molecular Phylogenetics and Evolution* (*n* = 3), *Molecular Biology and Evolution* (*n* = 2), *Molecular Ecology* (*n* = 2), *Genes and Genomics* (*n* = 2), and *Genetics and Molecular Biology* (*n* = 2), and one each in *Biochemical Systematics and Ecology*; *Biology Letters*; *BMC Genetics*; *Conservation Genetics Resources*; *Current Biology*; *Genes, Genetics and Systematics*; *Genetica*; *Genome Biology and Evolution*; *Genome Research*; *International Journal of Biological Macromolecules*; *Journal of Genetics*; *Pakistan Journal of Zoology*; *PloS One*; and *Zoological Research*.

For multiple problematic mitogenomes identified in this study, previously published phylograms already showed tell-tale signs of problematic sequence data but these were not flagged by the relevant authors. Several mitogenomes have been placed on a long branch in published phylogenies, indicative of a problematic sequence, including those of *Anser fabalis* (Liu et al. 2014; Dai et al. 2016; He et al. 2016; Zhang et al. 2017, Lin et al. 2018), *Vanellus cinereus* (Eo and An 2016; Chen et al. 2018), *Accipiter gularis* (Kim et al. 2019), *Strix leptogrammica* (Park et al. 2019a, 2019b), *Ninox strenua* (Kang et al. 2018; Park et al. 2019b), *Garrulax poecilorhynchus* (Qi et al. 2016), and *Motacilla lugens* (Sun et al. 2016; Mackiewicz et al. 2019).

Similarly, a short branch or very shallow divergence, which is suggestive of a misidentification, has been shown in published phylogenies of *Charadrius placidus* (Chen et al. 2018), *Aquila heliaca* (Knapp et al. 2019; Sarker et al. 2019a; Zhou et al. 2019), *Falco naumanni* (Yang et al. 2018), and *Emberiza aureola* (Zhang et al. 2016; Mackiewicz et al. 2019; Wu et al. 2019).

Finally, several misidentified or chimeric mitogenomes have turned up in unexpected positions in published phylogenies but were not flagged as possible cases of problematic sequence data in the relevant papers. These include a mitogenome of a nightjar in a clade of owls (Xu et al. 2016), a mitogenome of a gull in a clade of albatrosses, petrels, and shearwaters (Bi 2017; Jiang et al. 2018; Jiang 2019), a newly published mitogenome of Siberian Crane *Grus leucogeranus* that turned up as the sister to White-naped Crane *G. vipio* rather than to another Siberian Crane (Wang et al. 2018), a newly published cockatoo mitogenome placed amongst *Psittacula* parakeets (Sarker et al. 2019b), and a ground tit *Pseudopodoces humilis* (Paridae) amongst chats and flycatchers (Muscicapidae) (Ma et al. 2014; Peng et al. 2016).

Of the 78 problematic mitogenomes identified in this study, only 5 were previously flagged by other authors. AB918148 (*Bubo bubo*) was excluded by Kang et al. (2018) from their phylogenetic analysis based on “numerous ambiguities in their PCGs” (p. 10). Problems with KC953095 (*Strix leptogrammica*) were noted by Spiridonova and Surmach (2018) and Hanna et al. (2017). Spiridonova and Surmach (2018) correctly identified the published mitogenome of *Caprimulgus indicus* (KM272749) as a chimera. The first published mitogenome of *Pseudopodoces humilis* (HM535648) was flagged as problematic by Barker (2014), Gibb et al. (2015), and Xin et al. (2016). However, both Barker (2014) and Gibb et al. (2015) suggested that this was an unknown or mislabeled species of chat, whereas in fact, this sequence was a chimera of a chat (Muscicapidae) and a long-tailed tit (Aegithalidae) (supplementary table S3 and appendix S2, Supplementary Material online). Finally, Mackiewicz et al. (2019) noted that in some of their analyses KT895996 (*Passer ammodendri*) was placed among *Emberiza* buntings and that parts of this mitogenome clustered with *Passer* whereas other parts clustered with *Emberiza.* Nevertheless, most of these studies still included multiple other problematic sequences in their analyses (supplementary appendix S1, Supplementary Material online).

Three mitogenomes that were previously diagnosed as problematic (Kang et al. 2018; Spiridonova and Surmach 2018) could not be corroborated in the present study: AY309457 (*Ninox novaeseelandiae*), LC099104 (*Bubo blakistoni*) and KT350612 (*Sternula albifrons*) (supplementary appendix S3, Supplementary Material online).

## Discussion

### Problematic Mitogenomes

We detected 78 problematic sequences, corresponding to 5.0% of all 1,559 verifiable avian mitogenomes. This likely represents a low boundary of the true number of problematic mitogenomes in our data set. First, mitogenomes were compared with reference sequences of only three PCG markers (2,880 bp), which together represented only 17% of the median length of mitogenomes in this study (16,824 bp; *n* = 1,876). As a consequence, problems in other markers could not be detected. Second, our methodology focused on errors that could be detected visually from sequence alignments (i.e., insertions and deletions) or phylogenies. Thus, our methods were not sensitive enough to detect single nucleotide errors (e.g., Shi et al. 2014; Peng et al. 2015) or chimeras of closely related species.

### Causes of Problematic Mitogenomes

**Fig. 2:**
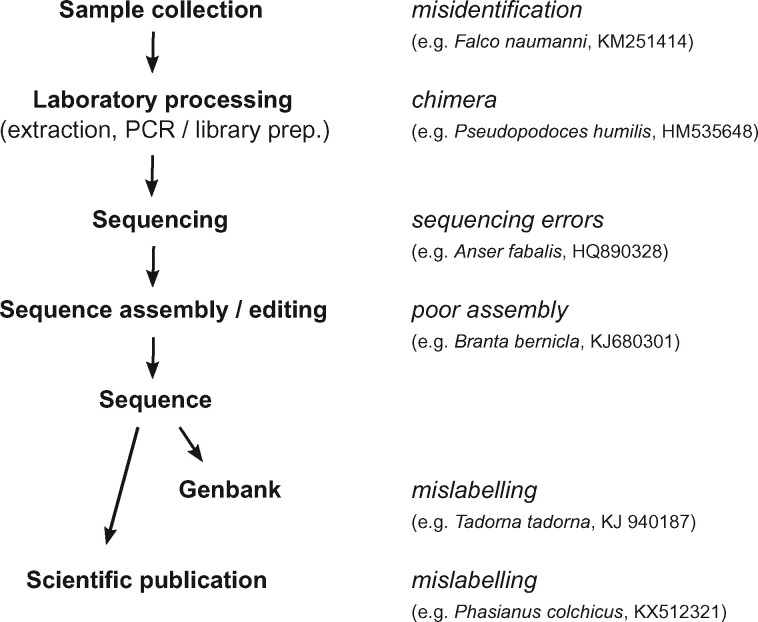
Authenticity problems with mitogenomes may originate at various steps, from sample collection in the field or museum to formal publication and deposition at GenBank.

We found six types of problematic sequences. These likely originated at multiple steps in the chain of events from identification in the field or museum to the registration of sequences on GenBank (fig. 2). Whilst misidentification was the most common cause of problems detected in our study, it accounted for less than half of all problematic mitogenomes and chimeras and sequencing errors/numts were also major sources of problematic mitogenomes.

Misidentification may be caused by morphological crypsis (cryptic species); a mismatch between an organism’s morphology and mitogenome due to introgressive hybridization; recent taxonomic change; or poor identification skills. Seven misidentifications in our study could be attributed to morphologically cryptic species (supplementary table S2, Supplementary Material online). However, for all these species diagnostic morphological characters have been documented in field guides and handbooks, and a taxonomic expert would have had no problem correctly identifying these species from adequate specimen or photographic material. In six cases, the two species were sympatric and known to hybridize in the wild. Thus, some misidentifications may have resulted from introgressive hybridization (supplementary table S2, Supplementary Material online). In nine cases, the misidentification of mitogenomes likely resulted from using samples labeled with an outdated taxonomic name (supplementary table S2, Supplementary Material online). In five of these cases the revision was adopted during the last decade. At least 12 misidentifications could not be readily explained by morphological crypsis, introgression, or outdated taxonomy. In these cases, we suspect the initial identifications were made by inexperienced workers or were based on inadequate materials, or both, and were not verified prior to DNA sequencing.

Chimeras result from a transfer of a DNA fragment of one species into a sequence of another. This may occur in the laboratory, as template DNA prior to PCR amplification or as PCR product prior to sequencing, or during sequence assembly/editing when a reference genome used to assemble the sequence fragments is not removed before calculating the consensus sequence (Norén and Kullander 2018; Sangster and Luksenburg 2020). Identification of the exact cause of the chimeras in this study is impossible without detailed laboratory records. In the case of KJ909187 (*Bombycilla cedrorum*), we suspect that the fragments of *Gracula religiosa* were incorporated during assembly because this is the closest relative of *Bombycilla* for which a mitogenome was available prior to the relevant study, which would make this a suitable reference genome, and because the author did not sequence any *Gracula religiosa* himself (Barker 2014). In any case, the chimeras identified in this study demonstrate that the relevant laboratories had insufficient quality checks to prevent the transfer of DNA fragments from sequences of one species to those of another, and to detect such transfers after sequence assembly. Other sequences produced by these laboratories, including nonavian mitogenomes, should be verified before their reusage in evolutionary studies.

The chimeras in our study were highly diverse in the number and the length of heterospecific fragments and in the proportion of heterospecific DNA. As a result, some were difficult to detect even with reference sequences of three PCG markers. For instance, some chimeras consisted of a single heterospecific fragment, or included only 2% heterospecific DNA, and could only be detected by showing a slightly longer branch in a gene tree of one of the three PCG markers.

Sequence errors/numts were detected in 18 mitogenomes. Whereas numts have received much attention in the context of shorter fragments of mitochondrial DNA (Arctander 1995; Sorenson and Fleischer 1996; Sorenson and Quinn 1998; Bensasson et al. 2001; Den Tex et al. 2010), our study shows that sequence errors or numts are still frequently being overlooked and find their way into published mitogenomes.

### Consequences of Problematic Mitogenomes

The number of problematic sequences and their usage in phylogenies has increased strongly since 2012. By January 1, 2020, more than half of all published avian mitogenomic phylogenies/networks included at least one problematic mitogenome. In some cases, a phylogeny or analysis included multiple problematic mitogenomic sequences (including two papers using >15 of such sequences; Nabholz et al. 2016; Mackiewicz et al. 2019). In most cases, the problematic mitogenome(s) were the only mitogenome of the species. We found that problematic mitogenome(s) were included in at least eight types of research: 1) phylogenetic inference, 2) taxonomy, 3) “falsification” of other mitochondrial sequences, 4) measurement of divergence times, 5) DNA identification of new sequence data, 6) comparative analysis of molecular evolution, 7) comparative analysis of mitochondrial gene order, and 8) mitogenome assembly of next-generation sequencing data.

In addition, at least two other problems may arise if problematic mitogenomes remain undetected. First, primers designed using misidentified or chimeric reference material may produce negative or suboptimal laboratory results. This would be especially problematic if the mitogenome is phylogenetically distantly related. Second, an undetected problematic mitogenome may dissuade others from generating a mitogenome of the relevant species and thus may slow down progress in the field.

Our study also shows that using outdated taxonomy and nomenclature is not a trivial problem. For instance, a study aimed at calculating the body-mass corrected substitution rate of birds (Nabholz et al. 2016) included a mitogenome sequence of “*Leucostictearctoa*” and associated that sequence with body-mass data of the small Asian species *L. arctoa* (30.75 g) rather than that of the heavier *L. tephronotus* (35.75 g) (Dunning 2007) from which the sequence was actually derived. The two species were formerly included in a single species. Thus, whereas the species names of the body-mass data were up-to-date, that of the sequence on GenBank was not. As minor as such an error may seem, this was just one of 19 problematic mitogenomes included in that study, which collectively may have added considerable—but avoidable—noise to the data set.

### Detection and Diagnosis of Problematic Mitogenomes

**Fig. 3:**
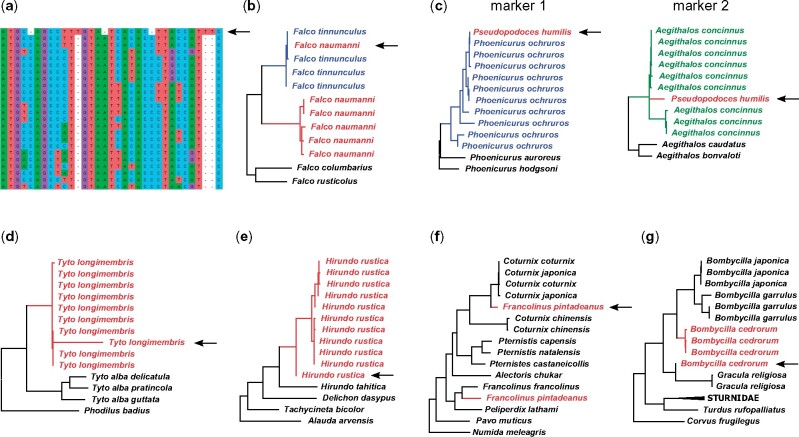
Seven different ways in which problematic mitogenomes were detected in this study: (*a*) Multiple insertions and/or deletions in an alignment of a PCG; (*b*) a close match with another species, outside the clade of other sequences of the same species; (*c*) mismatch between gene trees; (*d*) long branch within a clade of other sequences of the same species; (*e*) deep divergence, outside the clade of other sequences of the same species; (*f*) distant position from other sequence(s) of the same species, without a close match; (*g*) outside the clade of other sequences of the same species, with its sister lineage/clade placed on a long branch. Arrows indicate problematic mitogenomes. In each phylogeny, sequences with the same color are from the same species.

**Fig. 4:**
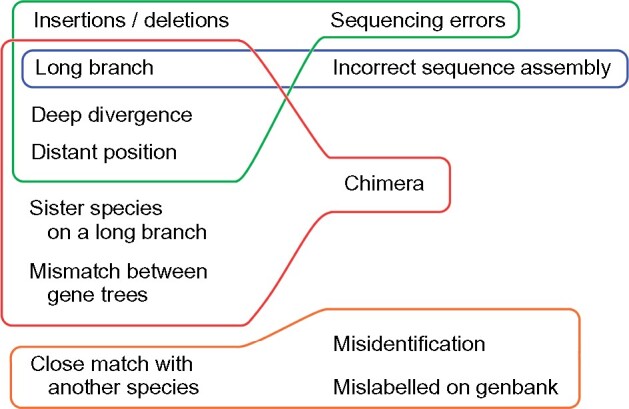
Combinations of “symptoms” (left column) and causes (right column) of problematic mitogenomes in the present study. Note that a single type of problem (e.g., chimera) may present itself in different ways, and that different causes may show the same symptom (e.g., long branch).

We found seven major indicators (“symptoms”) that a mitogenome was problematic: 1) Multiple insertions and/or deletions in an alignment of a PCG; 2) a close match with another species, outside the clade of other sequences of the same species; 3) mismatch between gene trees; 4) long branch within a clade of other sequences of the same species; 5) deep divergence, outside the clade of other sequences of the same species; 6) distant position from other sequence(s) of the same species, without a close match; 7) outside the clade of other sequences of the same species, with its sister lineage/clade placed on a long branch (fig. 3). Some of these indicators were not specific to a single type of problem (fig. 4). For instance, a sequence placed on a long branch may represent a chimera (fig. 3*c*, supplementary table S3, Supplementary Material online), or sequence errors/numts (supplementary table S4, Supplementary Material online) or an incorrectly assembled sequence (supplementary table S1, Supplementary Material online; Appendix A). When a sequence was placed distantly from sequences of the same species, but without a close match to a different species, this often represented a chimera. However, in one case phylogenetic analysis of the *cytochromeb* portion of a mitogenome with sequence errors or numts (AB918148, *Bubo bubo*), placed that sequence in another genus (*Ninox*) with high bootstrap support, but did not match any known species of that genus.

Conversely, a single type of sequence problem may present itself in multiple ways in phylogenies. For instance, in our data set there were five different indications that a mitogenome was a chimera: 1) A mismatch between gene trees; 2) a long branch within a clade of other sequences of the same species; 3) a deep divergence from other sequences of the same species; 4) a distant position from other sequence(s) of the same species, without a close match to another species; and 5) a position outside the clade of other sequences of the same species, with its sister lineage/clade placed on a long branch (fig. 4). Similarly, there were four different indications that a mitogenome contains sequence errors/numts: 1) Insertions/deletions in an alignment of a PCG; 2) a long branch in a gene tree; 3) a deep divergence from other sequences of the same species; and 4) a distant position from other sequence(s) of the same species, without a close match. Thus, detection of problematic sequences should take into account that such sequences may present themselves in a variety of ways, including but not necessarily limited to those observed in the present study.

## Conclusions and Recommendations

Our study highlights that erroneous sequences are a widespread problem. Problematic sequences 1) originate from incomplete quality checks during the entire process, 2) have been produced by 42 research groups in 8 countries, 3) have been published in 22 peer-reviewed journals, 4) are found across the bird phylogeny, (5) have been reused hundreds of times, and (6) have been used to address multiple research questions in systematics, evolutionary biology and ecology. The problems highlighted here show similarities to the discovery of ancient DNA artifacts (e.g., Wayne et al. 1999) and nuclear pseudogenes of mitochondrial DNA (e.g., Sorenson and Quinn 1998) in the 1990s, in that problematic sequences are being published that have detrimental effects on many studies. We believe a major upgrade of quality control standards for mitogenome sequences is warranted.

Previous recommendations for quality improvement typically addressed a single problem (e.g., misidentification, numts) for which a single solution was then suggested. As useful as these suggestions are, most of these cannot fully solve the problem they intend to. For instance, preserved voucher specimens can be used to independently verify and, if necessary, correct the original identification (Griffiths and Bates 2002; Bates et al. 2004; Peterson et al. 2007; Pleijel et al. 2008), but do not preclude the *publication* and *reuse* of misidentified or otherwise problematic sequences.

Second, the use of fresh tissue material makes it possible to produce long PCR amplicons which may help prevent the amplification of numts (Moyle et al. 2015). However, long numts have been documented (e.g., Nacer and do Amaral 2017); therefore, long sequence fragments amplified from fresh tissue samples may still represent numts.

Third, it has been proposed that DNA sequences be made available for peer review alongside the manuscript, so that the identity and quality of the sequences can be evaluated prior to publication (Harris 2003). Our study shows that even if mitogenome sequences are available on GenBank for verification, problematic ones are not detected and are still being reused.

Fourth, a phylogeny could be used to show the evolutionary position of a mitogenome, so that any major identification problem can be detected. Indeed, the journal *Mitochondrial DNA Part B* now requires the inclusion of a phylogeny in mitogenome data papers. Nevertheless, some published phylogenies still place new mitogenomes at highly dubious positions (e.g., a cockatoo amongst parakeets; Sarker et al. 2019b) or make little sense at all, possibly due to rooting or alignment errors (e.g., Park et al. 2016; Zhao et al. 2016; Lu et al. 2019).

Finally, the publication of phylogenies as a *phylogram* allows one to assess branch lengths, so that unexpectedly long or short branches can be flagged (Botero-Castro et al. 2016). Unfortunately, this advice is often ignored. As noted above, phylogenies that place a mitogenome on a suspiciously long or short branch are still being published.

We suspect ignorance of tell-tale signs of sequence problems is possibly a result of a general lack of awareness of the prevalence of sequence problems. Here we propose a comprehensive set of formal quality checks at multiple steps along the chain of events from identification to the registration and publication of sequences, that will upgrade the quality control standards for publishing and reusing mitogenome sequences.

### A Proposed “Gold Standard” for Publishing and Authenticating Mitogenomes

#### General

1. All quality checks that have been performed should be mentioned in the publication (e.g., in the methods section), so that end-users can ascertain which problems likely have been avoided and which problems may persist. This applies equally to original papers (e.g., data papers) and papers that reuse previously published mitogenomes. Evidence for all quality checks and the results of those checks should be preserved.

#### Identification, prior to Sequencing

2. Identification should be carried out by an appropriate specialist, so that taxonomic names are correctly applied both on GenBank and in the scientific literature (Kholia and Fraser-Jenkins 2011; Bortolus 2012; Vink et al. 2012). As shown in this study, misidentified sequences may compromise phylogenetic inference, taxonomy, historical biogeography, and comparative analysis.

3. A statement should be included about how the sample was identified. This should include any diagnostic character states of the relevant specimen. Such information could be included in the main text or in the online supporting materials. If no specimen was preserved, one or more photographs of the sampled individual which illustrate the diagnostic character states for the relevant taxon should be included.

#### Identification, after Sequencing

4. After sequencing, the draft sequence should be validated against sequences of the same species and other species (Botero-Castro et al. 2016), preferably using multiple markers (Sangster and Luksenburg 2020, 2021).

5. The phylogenetic position of the new sequence is best illustrated with a phylogram so that branch lengths can be evaluated and unusually long or short branches can be flagged by referees and readers (Botero-Castro et al. 2016). A phylogram may be included either in the main text or in the online supporting materials.

6. To avoid losing information about the identity of a sequence, it is recommended that not only the species name but also the relevant subspecies name is mentioned both in the data paper and on GenBank. This allows the reader and subsequent users to correct for outdated taxonomic names in data papers and on GenBank.

#### Peer-review

7. Journals should demand from authors that they make sequence data available for peer review along with the manuscript, so that any errors can be detected prior to publication (Harris 2003).

8. Manuscripts are best reviewed by at least one taxonomic specialist so that any problems resulting from the use of outdated taxonomy can be identified and corrected.

#### Prevention of Reusing Problematic Sequences

9. If no validation (see 4, above) has been carried out in the original publication, then the sequence should not be reused unless its identity has been verified with other, independently published sequences.

#### Other Recommendations

10. Given the critical importance of reliable DNA data, it would be extremely useful if the scientific community were able to comment on any problems associated with sequences published on GenBank (De Silva et al. 2019). One way to achieve this is to connect GenBank with a platform that enables scientists to provide feedback on DNA sequences, analogous to the role PubPeer.com provides for scientific publications.

11. Finally, data papers documenting problematic sequence should be promptly retracted by the authors and/or publishers, as well as hosting sites such as ResearchGate and Academia. If evidence for a problematic sequence is complex, or requires detailed scientific documentation, this is sometimes best published as a separate paper (e.g., Botero-Castro et al. 2016; Sangster and Luksenburg 2020).

12. Consideration should be given to a moratorium on publishing mitogenome data papers until journals have upgraded their quality control procedures.

## Materials and Methods 

### Data Set

The data set included 1,876 complete or partial (>12,000 bp) mitogenome sequences. We derived all sequences from GenBank, which is part of the International Nucleotide Sequence Database Collaboration, which comprises the DNA DataBank of Japan (DDBJ), the European Nucleotide Archive (ENA), and GenBank at NCBI, and which exchange sequence data submitted to each database. Mitogenome sequences were localized on 1) GenBank using the search string “Aves AND (mitochondrial OR mitochondrion) AND 12000:25000[Sequence Length],” 2) journal websites (e.g., those of *Mitochondrial DNAPartA* and *PartB*, *Conservation Genetics Resources*, *Molecular Phylogenetics and Evolution*, *Molecular Ecology*), 3) literature cited in mitogenome announcements, and 4) Google Scholar. We included all sequences that were available on GenBank before January 1, 2020, or were described in research or data papers published before that date. Sequences mentioned in papers published before January 1, 2020 but not yet available on GenBank were requested for release. If a sequence was published before January 1, 2020 but the paper (version of record) was published after that date, the sequence was included in the data set but the paper was not. We excluded all RefSeq sequences (with prefix “NC_”), which are exact copies of selected assembled genomes available in GenBank. We also excluded mitogenomes of putative hybrids and mitogenomes based on tissues derived from material dating from before the year 1800. The latter were excluded because DNA from pre-1800 tissue material was usually derived from skeletal material, which makes it more difficult to verify their identity with closely related species (which tend to differ by plumage only).

### Definition of a Problematic Mitogenome

For the purpose of this study, we recognize six classes of problematic mitogenomes: Misidentification (both in the published paper, if any, and on GenBank), chimeras, poor sequence quality, or numts (i.e., nuclear copies of mitochondrial sequence fragments), incorrect sequence assembly (i.e., parts of mitogenome incorporated at nonhomologous positions in the consensus genome), ID mislabeled in paper or on GenBank.

### Detection of Problematic Mitogenomes

We assessed the phylogenetic position of mitogenomes in gene trees of three PCGs: *NADH dehydrogenase subunit 2* (*ND2*, 1,041 bp), *cytochrome oxidase subunit I* (*COI*, 696 bp), and *cytochromeb* (1,143 bp). These are the three most commonly used mitochondrial markers in ornithology (Jetz et al. 2012). We focused on sequences of >600 bp (*COI*) or >800 bp (*cytochromeb*, *ND2*), but shorter sequences were included when such data were not available. DNA sequences were aligned with ClustalW, implemented in MEGA7 (Kumar et al. 2016). Initial screenings were conducted with MEGA 7 using Maximum Likelihood phylogenies of 100 − 3,500 sequences using a GTR + G + I model. Mitogenome sequences that could not be compared with sequences of conspecifics for any of the three markers were excluded. Mitogenome sequences were also excluded if there was no resolution in gene trees of any of the three markers, unless there were clear signs that a sequence is problematic (e.g., a long branch, see below).

A mitogenome sequence was flagged if at least one of the following was observed: 1) Multiple insertions and/or deletions in an alignment of a PCG, 2) a close match with another species, outside the clade of other sequences of the same species; 3) a mismatch between the phylogenetic position in different gene trees, controlling for genus name revisions and differences in spelling; 4) a long or very short branch (either in our analyses, or in published phylogenies of these sequences); and 5) an unexpected phylogenetic position without a close match with another species. All flagged sequences were visually inspected and compared with sequences of conspecifics, or at least close relatives, to localize any divergent fragment(s). The divergent fragment(s) were entered in a BLAST search to help identify their identity.

### Diagnosis of Problematic Mitogenomes

#### Misidentification

A mitogenome was diagnosed as resulting from misidentification if the sequence ended up at the same incorrect position in all gene trees and if the sequence was not placed on a long branch. Identification was only attempted at the level of species. Subspecies typically do not show reciprocally monophyletic groups (Zink 2004) which is expected because the phenotypic differences on which subspecies tend to be based may be ephemeral or represent local adaptations, and often originate before coalescence of mitochondrial DNA (Patten and Remsen 2017). If reference sequences are unresolved (did not show reciprocal monophyly of species) the identity of the mitogenome was scored as “could not be verified.”

Mitochondrial introgression may produce the same effect as misidentification: A mismatch between the species label of a mitogenome and its position in a mitochondrial gene tree. We did not attempt to differentiate between these processes due to lack of access to morphological (e.g., specimen) data or nuclear DNA evidence of the relevant samples. However, we noted those cases where the species were known to hybridize (McCarthy 2006).

#### Chimera

A mitogenome was diagnosed as a chimera if different fragments showed a close match to different species. For each chimera we identified the homospecific (if any) and heterospecific fragments by direct comparison with sequences of these species or those of closely related species. The combined length of these fragments was used to estimate the heterospecific proportion of the mitogenome. We also calculated the sequence divergence between the homo- and heterospecific fragments. In some cases, if reference sequences of *ND2*, *COI* or *cytochromeb* allowed identification of the homo- or heterospecific fragment(s) but no authentic full mitogenome of these species was available, we did not identify the homo- or heterospecific fragments, and did not calculate the homo- or heterospecific proportions of the mitogenome.

#### Sequencing Errors/Numts

A mitogenome was diagnosed as having sequencing errors or possible numts if there were multiple insertions or deletions in at least one of the three PCGs, or if the divergent part(s) of the sequence—as established from side-by-side comparison with sequences from conspecifics (which we assumed to be trustworthy)—did not closely match any species (as verified with BlastN). No distinction was made between sequencing errors and numts due to difficulties in formally diagnosing the latter (which requires matching the fragment with a known nuclear copy; Nacer and do Amaral 2017).

#### Incorrect Sequence Assembly

In cases where a species-specific DNA fragment of a PCG was located at a nonhomologous position, or partially duplicated, the problem was classified as “incorrect sequence assembly.”

#### Mislabeled in Paper

If a sequence was correctly identified on GenBank but incorrectly identified in the paper, the sequence was diagnosed as being “mislabeled in paper.”

#### Mislabeled on GenBank

A sequence that was published under the correct name in the paper but incorrectly on GenBank was diagnosed as being “mislabeled on GenBank.”

### Reusage of Problematic Mitogenomes

To assess whether problematic mitogenomes have been reused in phylogenetic studies, we examined all mitogenomic phylogenies and networks (*n* = 342) published until December 31, 2019, including phylogenies of papers that did not describe a new mitogenome (supplementary appendix S1, Supplementary Material online). We searched Google Scholar (January 2020) for other uses of problematic mitogenomes, using the GenBank accession numbers, and the titles of the relevant data papers, as search terms.

## Supplementary Material

Supplementary data are available at *Genome Biology and Evolution* online.

## Supplementary Material

evab210_Supplementary_Data

## Appendix A Problematic Mitogenomes of Birds Published before January 1, 2020 (*n* = 78)

GALLIFORMES: *Centrocercus minimus*, CM016737, sequencing errors/numts; *Francolinus pintadeanus*, EU165707 (NC_011817), chimera; *Gallus*, KT626849, sequencing errors/numts; *Phasianus colchicus*, KX512321, mislabeled in original data paper; *Phasianus versicolor*, AB164626 (NC_010778), chimera; ANSERIFORMES: *Branta bernicla*, KJ680301, poor sequence assembly; *Anser fabalis*, HQ890328 (NC_016922), sequencing errors/numts; *Tadorna tadorna*, KJ794187 (NC_024750), mislabeled on GenBank; *Tadorna*, MN258348, sequencing errors/numts; *Aix galericulata*, KJ169568 (NC_023969), chimera; *Anas crecca*, KF203133 (NC_022452), sequencing errors/numts; *Anas clypeata*, KT345702 (NC_028346), sequencing errors/numts; *Anas falcata*, KC759527 (NC_023352), misidentification; *Netta rufina*, KC466568 (NC_024922), chimera; CAPRIMULGIFORMES: *Caprimulgus jotaka*, KM272749 (NC_025773), chimera; *Phaethornis ma*laris, KP853097 (NC_030288), misidentification; CUCULIFORMES: *Cuculus canorus*, MN067867, sequencing errors/numts; GRUIFORMES: *Coturnicops exquisitus*, AP010823 (NC_012143), sequencing errors/numts; *Amaurornis akool*, KJ192198 (NC_023982), chimera; *Amaurornis phoenicurus*, KJ874440 (NC_024593), chimera; *Rallus aquaticus*, MH229988 (NC_041578), misidentification; *Grus leucogeranus*, MH041490, misidentification; CHARADRIIFORMES: *Vanellus cinereus*, KM873665, chimera; *Charadrius placidus*, KY419888, misidentification; *Tringa totanus*, MK922124 (NC_044648), sequencing errors/numts; *Larus vegae*, KT943749, misidentification; PROCELLARIIFORMES: *Hydrobates castro*, MH433599 (NC_041251), misidentification; *Hydrobates castro*, MK170187, misidentification; ACCIPITRIFORMES: *Aquila heliaca*, KU646835 (NC_035806), misidentification; *Aquila audax*, MG873530, sequencing errors/numts; *Accipiter gularis*, KX585864, chimera; STRIGIFORMES: *Tyto longimembris*, KP893332, chimera; *Otus bakkamoena*, KT340631 (NC_028163), chimera; *Otus scops*, KT340630 (NC_028162), misidentification; *Otus scops*, KY471456, misidentification; *Bubo bubo*, AB918148, sequencing errors/numts; *Strix leptogrammica*, KC953095 (NC_021970), sequencing errors/numts; *Glaucidium brodiei*, MF155890, misidentification + additional sequencing errors/numts; *Ninox strenua*, KX529654 (NC_033967), sequencing errors/numts; TROGONIFORMES: *Trogon viridis*, EU410490 (NC_011714), misidentification; BUCEROTIFORMES: *Anthracoceros coronatus*, MF435900 (NC_038152), misidentification; CORACIIFORMES: *Alcedo atthis*, KY964271 (NC_035868), chimera; *Megaceryle lugubris*, KY940558 (NC_035658), misidentification + additional sequencing errors/numts; *Ceryle rudis*, KJ461938 (NC_024280), chimera; PICIFORMES: *Yungipicus canicapillus*, MK335534, chimera; FALCONIFORMES: *Falco naumanni*, KM251414 (NC_029846), misidentification; *Falco mexicanus*, sequence available at Dryad (https://doi.org/10.5061/dryad.8b0s04t), sequencing errors/numts; PSITTACIFORMES: *Cacatua sanguinea*, MN126573, misidentification; *Agapornis pullarius*, MN481404 (NC_045368), mislabeled on GenBank; *Brotogeris cyanoptera*, HM627323 (NC_015530), sequencing errors/numts; PASSERIFORMES: *Thamnophilus nigrocinereus*, KJ909192, misidentification + additional sequencing errors/numts; *Lanius tephronotus*, JX486029 (NC_021105), misidentification; *Corvus coronoides*, MF370524 (NC_035877), chimera; *Quoyornis georgiana*, KM374638, misidentification; *Eopsaltria griseogularis*, KM374625, misidentification; *Bombycilla cedrorum*, KJ909187, chimera; *Periparus ater*, KM588075 (NC_026223), misidentification; *Poecile palustris*, KX388475, misidentification; *Pseudopodoces humilis*, HM535648 (NC_014341), chimera; *Hirundo rustica*, KP148840, sequencing errors/numts; *Seicercus burkii*, KX977449, misidentification; *Garrulax albogularis*, KX082660 (NC_037464), chimera; *Garrulax poecilorhynchus*, KR909134 (NC_028082), chimera; *Garrulax perspicillatus*, KF997865 (NC_026068), misidentification; *Garrulax milnei*, MH238447 (NC_041141), chimera; *Sturnus nigricollis*, JQ003192, chimera; *Turdus merula*, KT373849 (NC_028188), misidentification; *Turdus merula*, KT601060, misidentification; *Muscicapa griseisticta*, MK390479 (NC_045181), misidentification; *Cyanoptila cyanomelana*, HQ896033 (NC_015232), misidentification; *Passer ammodendri*, KT895996, chimera; *Passer montanus*, MH211399, misidentification; *Motacilla lugens*, KU246035 (NC_029703), chimera; *Leucosticte arctoa*, KM078791 (NC_025615), misidentification; *Haemorhous mexicanus*, FJ236300, misidentification; *Emberiza chrysophrys*, HQ896034 (NC_015233), sequencing errors/numts; *Emberiza aureola*, KF111713 (NC_022150), misidentification + additional sequencing errors/numts; *Emberiza pallasi*, MK687386, sequencing errors/numts.
