# Supplementary material for: Sharp Increase of Problematic Mitogenomes of Birds: Causes, Consequences, and Remedies
Source: Genome Biol Evol. 2021 Sep 10;13(9):evab210. doi: 10.1093/gbe/evab210 (PMC8462277; doi:10.1093/gbe/evab210)
Supplement: evab210_Supplementary_Data [file evab210_supplementary_data.pdf]

ONLINE SUPPORTING MATERIALS

Sharp increase of problematic mitogenomes of birds: causes, consequences and remedies

George Sangster & Jolanda A. Luksenburg

pp. 2–10	**TABLE S1.** Summary data on problematic mitogenomes of birds (n=78).
pp. 11–12	**TABLE S2.** Characteristics of 33 misidentified mitogenomes.
pp. 13–14	**TABLE S3.** Characteristics of 23 chimeras identified in this paper.
p. 15	**TABLE S4.** Characteristics of 18 mitogenomes with sequencing errors / numts identified in this paper.
pp. 16–19	**TABLE S5.** Applications of problematic mitogenomes and their potential effects.
pp. 20–119	**APPENDIX S1.** Mitogenomes (n=1876) analysed for this study.
pp. 120–272	**APPENDIX S2.** Problematic mitogenomes identified in this study (n=78), including evidence from gene trees and re-use in subsequent studies.
	**FIGURES S1 – S64** Maximum Likelihood phylogenies based on mitochondrial ND2, COI and cytochrome *b* sequences showing the phylogenetic position and branch length of 78 problematic mitogenomes identified in this study.
pp. 273–278	**APPENDIX S3.** Problematic mitogenomes reported by others which could not be confirmed in the present study.
	**FIGURE S65.** Maximum Likelihood phylogenies of *Ninox novaeseelandiae* (AY309457), *Bubo blakistoni* (LC099104) and related taxa based on mitochondrial sequences. Numbers at branches are bootstrap support values (>70%) based on 1000 replicates.
	**FIGURE S66.** Maximum Likelihood phylogenies of *Sternula albifrons* (KT350612) and related taxa based on three mitochondrial markers. Numbers at branches are bootstrap support values (>70%) based on 1000 replicates.
	**FIGURE S67.** Maximum Likelihood phylogenies of *Sternula albifrons* (KT350612) and related taxa based on twelve mitochondrial markers. Numbers at branches are bootstrap support values based on 1000 replicates. Note that in some phylogenies (a - f) a chimeric sequence of the owl *Otus bakkamoena* (KT340631) clusters with KT350612.

TABLE S1. Summary data on problematic mitogenomes of birds (n=71). Species names marked with an asterisk* indicate species for which no unproblematic mitogenome has been published. NCBI reference sequence numbers marked with a hash # have been removed from the RefSeq database.

No	Species name	GenBank Accession, NCBI reference sequence	Reference	Research group	Journal	Problem	Mitogenomic phylogenies re-using sequence (non-mitogenomic)	Other uses	Previously flagged	Gene tree(s)
	**Galliformes**									
1	"*Centrocercus minimus*"*	CM016737	Oh et al. (2019)	U.S. Geological Survey, Fort Collins Sci. Center, Fort Collins, CO, **USA**	Genome Biology and Evolution	**Sequencing errors / numts**	-	-	-	S1
2	"*Francolinus pintadeanus*"	EU165707, NC_011817	Shen et al. (2009)	Kunming Inst. of Zool., The Chinese Academy of Sciences, Kunming, **China**	Genome Research	**Chimera** (*Francolinus pintadeanus, Coturnix japonica*)	20 (2)	2	-	S2
3	"*Gallus gallus*"	KT626849	Alexander et al. (2015)	Univ. York, York / Univ. Aberdeen, Aberdeen, **UK**	Biology Letters	**Sequencing errors / numts**	-	-	-	S3
4	"*Phasianus colchicus*"	KX512321	Zhu et al. (2017)	Sichuan Agricultural Univ., Chengdu, Sichuan, **China**	Mitochondrial DNA Part B	**Mislabelled** in Zhu et al. (2017) (correctly listed on GenBank as *Gallus gallus*)	-	-	-	S3
5	"*Phasianus versicolor*"*	AB164626, NC_010778	Kato, S. et al., *unpublished*; 2004	Hiroshi Yasue Natl. Inst. of Agrobiol.ogical Sciences, Ikenodai, Ibaraki, **Japan**	-	**Chimera** (*Phasianus versicolor, Syrmaticus soemmerringii*)	27 (1)	4	-	S3
	**Anseriformes**									
6	"*Branta bernicla*"*	KJ680301	Lee, Y.J. et al., *unpublished*; 2014	Kyungpook Natl. Univ., Daegu, **South Korea**	-	**Poor sequence assembly** resulting in partial duplication of cytochrome b	5	-	-	S4
7	"*Anser fabalis*"*	HQ890328, NC_016922	Liu et al. (2013)	Anhui Univ., Hefei, Anhui, **China**	PLoS One	**Sequencing errors / numts**	17	1	-	S5

No	Species name	GenBank Accession, NCBI reference sequence	Reference	Research group	Journal	Problem	Mitogenomic phylogenies re-using sequence (non-mitogenomic)	Other uses	Previously flagged	Gene tree(s)
8	"*Tadorna tadorna*"	KJ794187, NC_024750	Lin et al. (2016a)	Hunan Agricultural Univ., Changsha, Hunan, **China**	Mitochondrial DNA Part A	**Mislabelled** on GenBank (correctly identified in paper as *Anas platyrhynchos*)	2	-	-	S6
9	"*Tadorna tadorna*"	MN258348	Liu et al. (2019k)	Anhui Medical Univ., Hefei, Anhui, **China**	Mitochondrial DNA Part B	**Sequencing errors / numts**	-	-	-	S6
10	"*Aix galericulata*"	KJ169568, NC_023969	Meng et al. (2016a)	Guangxi Normal Univ. / Yulin Normal Univ., Guangxi, **China**	Mitochondrial DNA	**Chimera** (*Aix galericulata, Aythya ferina*)	5	-	-	S7
11	"*Anas crecca*"	KF203133, NC_022452	Hu et al. (2015)	Nanjing Normal Univ., Nanjing, Jiangsu, **China**	Mitochondrial DNA	**Sequencing errors / numts**	14	-	-	S8
12	"*Anas clypeata*"*	KT345702, NC_028346	Sun et al. (2016a)	Anhui Univ., Hefei, Anhui, **China**	Mitochondrial DNA Part A	**Sequencing errors / numts**	3	1	-	S9
13	"*Anas falcata*"*	KC759527, NC_023352	Pan et al. (2014)	Anhui Univ., Hefei, Anhui, **China**	Mitochondrial DNA	**Misidentification** (*Anas penelope* or hybrid)	15	6	-	S10
14	"*Netta rufina*"*	KC466568, NC_024922	Kan, X.-Z. & Li, X.-X., *unpublished*; 2013	Anhui Normal Univ., Wuhu, Anhui, **China**	-	**Chimera** (*Netta rufina, Syrmaticus reevesii*)	6 (1)	-	-	S11
	**<u>Caprimulgiformes</u>**									
15	"*Caprimulgus jotaka*"*	KM272749, NC_025773	Zhao et al. (2016a)	Anhui Univ., Hefei, Anhui, **China**	Mitochondrial DNA Part A	**Chimera** (*Otus semitorques, Glaucidium cuculoides*)	5 (1)	-	1	S12
16	"*Phaethornis malaris*"*	KP853097, NC_030288	Costa, I.R. et al., *unpublished*; 2015	Univ. Federal do Rio de Janeiro, RJ, **Brazil**	-	**Misidentification** (*Threnetes leucurus* or *T. niger*)	1	-	-	S13
	**<u>Cuculiformes</u>**									
17	"*Cuculus canorus*"*	MN067867	Qiu et al. (2019)	Sichuan Univ., Chengdu, Sichuan, **China**	Mitochondrial DNA Part B	**Sequencing errors / numts**	-	-	-	S14
	**<u>Gruiformes</u>**									

No	Species name	GenBank Accession, NCBI reference sequence	Reference	Research group	Journal	Problem	Mitogenomic phylogenies re-using sequence (non-mitogenomic)	Other uses	Previously flagged	Gene tree(s)
18	"*Coturnicops exquisitus*"*	AP010823, NC_012143	Ozaki et al. (2010)	Yamashina Inst. of Ornithology, Abiko, Chiba, **Japan**	Genes, Genetics and Systematics	**Sequencing errors / numts**	7 (1)	1	-	S15
19	"*Amaurornis akool*"*	KJ192198, NC_023982	Gong et al. (2017)	Ludong Univ., Yantai, Shandong, **China**	Genes & Genomics	**Chimera** (*Amaurornis phoenicurus, Gallinula chloropus*)	2	-	-	S16
20	"*Amaurornis phoenicurus*"*	KJ874440, NC_024593	Gong et al. (2017)	Ludong Univ., Yantai, Shandong, **China**	Genes & Genomics	**Chimera** (*Amaurornis phoenicurus, Gallinula chloropus*)	4	-	-	S16
21	"*Rallus aquaticus*"*	MH229988, NC_041578	Chen et al. (2020)	Nanchang Univ., Nanchang, Jiangxi / Nanjing Inst. Env. Sci., Ministry Ecol. Env., Nanjing **China**	Pakistan Journal of Zoology	**Misidentification** (*Rallus indicus*) resulting from outdated taxonomy	-	-	-	S17
22	"*Grus leucogeranus*"	MH041490	Wang et al. (2018)	Heilongjiang Acad. Agricultural Sciences / Northeast Forestry Univ., Harbin, **China**	Mitochondrial DNA Part B	**Misidentification** (*Grus vipio*)	-	-	-	S18
	**<u>Charadriiformes</u>**									
23	"*Vanellus cinereus*"	KM873665	She et al. (2016)	Anhui Univ., Hefei, Anhui, **China**	Mitochondrial DNA Part A	**Chimera** (*Vanellus cinereus, Motacilla alba*)	7	2	-	S19
24	"*Charadrius placidus*"*	KY419888	Lee et al. (2017a)	Natl. Inst. of Biol. Resources, Incheon, **South Korea**	Mitochondrial DNA Part B	**Misidentification** (*Charadrius alexandrinus*)	5	-	-	S20
25	"*Tringa totanus*"*	MK922124, NC_044648	Ren et al. (2019b)	Sichuan Univ., Chengdu, Sichuan, **China**	Mitochondrial DNA Part B	**Sequencing errors / numts**	-	-	-	S21

No	Species name	GenBank Accession, NCBI reference sequence	Reference	Research group	Journal	Problem	Mitogenomic phylogenies re-using sequence (non-mitogenomic)	Other uses	Previously flagged	Gene tree(s)
26	"*Larus vegae*"*	KT943749	Park, C.E. et al., *unpublished*; 2015	Kyungpook Natl. Univ., Daegu, **South Korea**	-	**Misidentification** (*Calonectris leucomelas*)	5	1	-	S22
	**Procellariiformes**									
27	"*Hydrobates castro*"*	MH433599, NC_041251	Jiang et al. (2018)	Henan Inst. of Science and Technology, Xinxiang, **China**	Mitochondrial DNA Part B	**Misidentification** (*Hydrobates jabejabe*) resulting from outdated taxonomy	-	-	-	S23
28	"*Hydrobates castro*"*	MK170187	Antaky et al. (2019)	Univ. of Hawai'i, Honolulu, HI, **USA**	Mitochondrial DNA Part B	**Misidentification** (*Hydrobates leucorhous*)	-	-	-	S23
	**Accipitriformes**									
29	"*Aquila heliaca*"*	KU646835, NC_035806	Zhou, L. & Dong, Y., *unpublished*; 2016	Anhui Univ., Hefei, Anhui, **China**	-	**Misidentification** (*Aquila chrysaetos*)	4	1	-	S24
30	"*Aquila audax*"	MG873530	Sarker et al. (2019a)	La Trobe Univ., Melbourne, **Australia**	Mitochondrial DNA Part B	**Sequencing errors / numts**	-	-	-	S24
31	"*Accipiter gularis*"*	KX585864	Liu et al. (2017a)	Anhui Medical Univ., Hefei, Anhui, **China**	Biochemical Genetics and Ecology	**Chimera** (*Buteo buteo burmanicus, Streptopelia orientalis, Accipiter gularis*)	4	-	-	S25
	**Strigiformes**									
32	"*Tyto longimembris*"*	KP893332	Xu et al. (2016b)	Jiangxi Normal Univ., Nanchang, Jiangxi, **China**	Mitochondrial DNA Part A	**Chimera** (*Tyto longimembris, Buteo buteo burmanicus*)	5	-	-	S26
33	"*Otus bakkamoena*"*	KT340631, NC_028163	Park et al. (2019b)	Kyungpook Natl. Univ., Daegu, **South Korea**	Mitochondrial DNA Part B	**Chimera** (*Otus semitorques, Sternula albifrons*)	6	-	-	S27
34	"*Otus scops*"*	KT340630, NC_028162	Park et al. (2019a)	Kyungpook Natl. Univ., Daegu, **South Korea**	Mitochondrial DNA Part B	**Misidentification** (*Otus sunia*) resulting from outdated taxonomy	8	-	-	S27

No	Species name	GenBank Accession, NCBI reference sequence	Reference	Research group	Journal	Problem	Mitogenomic phylogenies re-using sequence (non-mitogenomic)	Other uses	Previously flagged	Gene tree(s)
35	"*Otus scops*"*	KY471456	Liu et al. (2019a)	Anhui Medical Univ., Hefei, Anhui, **China**	(PeerJ Preprints)	**Misidentification** (*Otus sunia*) resulting from outdated taxonomy	-	-	-	S27
36	"*Bubo bubo*"	AB918148	Tian et al. (2016)	Beijing Wildlife Rescue and Rehabilitation Center, Beijing, **China**	Mitochondrial DNA Part A	**Sequencing errors / numts**	4	3	1	S28
37	"*Strix leptogrammica*"*	KC953095, NC_021970#	Liu et al. (2014)	Anhui Univ., Hefei, Anhui, **China**	Mitochondrial DNA	**Sequencing errors / numts**	13 (1)	3	2	S29
38	"*Glaucidium brodiei*"*	MF155890	Liu et al. (2019a)	Anhui Medical Univ., Hefei, Anhui, **China**	(PeerJ Preprints)	**Misidentification** (*Otus sunia*)	-	-	-	S30
39	"*Ninox strenua*"*	KX529654, NC_033967	Sarker et al. (2016)	La Trobe Univ., Melbourne, **Australia**	Mitochondrial DNA Part B	**Sequencing errors / numts**	6	-	-	S31
	**<u>Trogoniformes</u>**									
40	"*Trogon viridis*" *	EU410490, NC_011714	Pratt et al. (2009)	Massey Univ., Palmerston North, **New Zealand**	Molecular Biology and Evolution	**Misidentification** (*Trogon chionurus*) resulting from outdated taxonomy	7	-	-	S32
	**<u>Bucerotiformes</u>**									
41	"*Anthracoceros coronatus*"*	MF435900, NC_038152	Li et al. (2018)	Yunnan Univ., Kunming, Yunnan, **China**	Conservation Genetics Resources	**Misidentification** (*Anthracoceros albirostris*) resulting from outdated taxonomy	-	1	-	S33
	**<u>Coraciiformes</u>**									
42	"*Alcedo atthis*"*	KY964271, NC_035868	Jing et al. (2020)	Nantong Univ., Nantong, Shandong, **China**	Genetics and Molecular Biology	**Chimera** (*Alcedo atthis, Gallinula chloropus*)	1	-	-	S34
43	"*Megaceryle lugubris*"*	KY940558, NC_035658	Jing et al. (2020)	Nantong Univ., Nantong, Shandong, **China**	Genetics and Molecular Biology	**Misidentification** (*Ceryle rudis*) + additional sequencing errors / numts	1	-	-	S35
44	"*Ceryle rudis*"*	KJ461938, NC_024280	Sun et al. (2017c)	Ludong Univ., Yantai, Shandong, **China**	Genetica	**Chimera** (*Ceryle rudis, Megaceryle lugubris*)	5	-	-	S35

No	Species name	GenBank Accession, NCBI reference sequence	Reference	Research group	Journal	Problem	Mitogenomic phylogenies re-using sequence (non-mitogenomic)	Other uses	Previously flagged	Gene tree(s)
	**Piciformes**									
45	"*Yungipicus canicapillus*"	MK335534	Bi et al. (2019)	Anhui Normal Univ., Wuhu, Anhui, **China**	International Journal of Biological Macromolecules	**Chimera** (*Yungipicus canicapillus, Dendrocopos darjellensis*)	-	-	-	S36
	**Falconiformes**									
46	"*Falco naumanni*"*	KM251414, NC_029846	Wang et al. (2016)	Kunming Medical Univ., Kunming, Yunnan, **China**	Mitochondrial DNA Part A	**Misidentification** (*Falco tinnunculus*)	7	-	-	S37
47	"*Falco mexicanus*"*	n.a.	Doyle et al. (2018)	Towson Univ., Baltimore, MD / Purdue Univ., West Lafayette, IN, **USA**	BMC Genetics	**Sequencing errors / numts**	-	-	-	S38
	**Psittaciformes**									
48	"*Cacatua sanguinea*"	MN126573	Sarker et al. (2019c)	La Trobe Univ., Melbourne, **Australia**	Mitochondrial DNA Part B	**Misidentification** (*Psittacula krameri*)	-	-	-	S39
49	"*Agapornis pullarius*"*	MN481404, NC_045368	Chen et al. (2019e)	Key Lab. of Wildlife Evid. Technology, Nanjing, **China**	Mitochondrial DNA Part B	**Mislabelled** on GenBank (correctly identified in paper as *Agapornis personatus*)	-	-	-	S40
50	"*Brotogeris cyanoptera*" *	HM627323, NC_015530	Pacheco et al. (2011)	Arizona State University, AZ, **USA**	Molecular Biology and Evolution	**Sequencing errors / numts**	10	-	-	S41
	**Passeriformes**									
51	"*Thamnophilus nigrocinereus*"*	KJ909192	Barker (2014)	Univ. Minnesota, Saint Paul, MN, **USA**	Molecular Phylogenetics and Evolution	**Misidentification** (*Percnostola rufifrons*) + additional issues with cyt b	5	-	-	S42
52	"*Lanius tephronotus*"*	JX486029, NC_021105	Qian et al. (2013)	Prov. Key Lab. of the Conserv. and Expl. Res. of Biol. Res., Anhui, Wuhu, **China**	Mitochondrial DNA	**Misidentification** (*Lanius schach*)	13	4	-	S43
53	"*Corvus coronoides*"*	MF370524, NC_035877	Sarker et al. (2017a)	La Trobe Univ., Melbourne, **Australia**	Mitochondrial DNA Part B	**Chimera** (*Corvus coronoides, Mus musculus*)	2	-	-	S44

No	Species name	GenBank Accession, NCBI reference sequence	Reference	Research group	Journal	Problem	Mitogenomic phylogenies re-using sequence (non-mitogenomic)	Other uses	Previously flagged	Gene tree(s)
54	"*Quoyornis georgiana*"	KM374638	Morales et al. (2015)	Monash Univ., Melbourne, **Australia**	Molecular Ecology	**Misidentification** (*Eopsaltria griseogularis*)	-	1	-	S45
55	"*Eopsaltria griseogularis*"	KM374625	Morales et al. (2015)	Monash Univ., Melbourne, **Australia**	Molecular Ecology	**Misidentification** (*Quoyornis georgiana*)	1	-	-	S45
56	"*Bombycilla cedrorum*"*	KJ909187	Barker (2014)	Univ. Minnesota, Saint Paul, MN, **USA**	Molecular Phylogenetics and Evolution	**Chimera** (*Bombycilla cedrorum, Gracula religiosa*)	2	1	-	S46
57	"*Periparus ater*"*	KM588075, NC_026223	Zhang et al. (2016g)	Shaanxi Normal Univ., Xi'an, Shaanxi, **China**	Mitochondrial DNA Part A	**Misidentification** (*Periparus rubidiventris*)	7	1	-	S47
58	"*Poecile palustris*"	KX388475	Li et al. (2016b)	Shaanxi Normal Univ., Xi'an, Shaanxi, **China**	Molecular Phylogenetics and Evolution	**Misidentification** (*Poecile weigoldicus*)	1	-	-	S48
59	"*Pseudopodoces humilis*"	HM535648, NC_014341	Yang et al. (2010)	Shaanxi Normal Univ., Xi'an, Shaanxi, **China**	Zoological Research	**Chimera** (*Phoenicurus ochruros, Aegithalos concinnus*)	10	10	3	S49
60	"*Hirundo rustica*"	KP148840	Liu et al., *unpublished*; 2014	Fudan Univ., Shanghai, **China**	-	**Sequencing errors / numts**	4	-	-	S50
61	"*Seicercus burkii*"*	KX977449	Zhang et al., *unpublished*, 2019	Sichuan Univ., Chengdu, Sichuan, **China**	-	**Misidentification** (*Seicercus affinis*)	-	-	-	S51
62	"*Garrulax albogularis*"*	KX082660, NC_037464	Liu et al. (2018a)	Sichuan Agricultural Univ., Ya'an / Chengdu, Sichuan, **China**	Mitochondrial DNA Part B	**Chimera** (*Garrulax albogularis, Minla ignotincta*)	2	-	-	S52
63	"*Garrulax poecilorhynchus*"*	KR909134, NC_028082	Qi et al. (2016b)	Sichuan Agricultural Univ., Ya'an / Chengdu, Sichuan, **China**	Mitochondrial DNA Part A	**Chimera** (*Garrulax poecilorhynchus, Leiothrix lutea*)	4	-	-	S52

No	Species name	GenBank Accession, NCBI reference sequence	Reference	Research group	Journal	Problem	Mitogenomic phylogenies re-using sequence (non-mitogenomic)	Other uses	Previously flagged	Gene tree(s)
64	“*Garrulax perspicillatus*”*	KF997865, NC_026068	Zhang et al. (2016d)	Anhui Normal Univ., Wuhu, Anhui / Yancheng Teachers Univ., Yancheng, Jiangsu, **China**	Mitochondrial DNA	**Misidentification** (*Garrulax pectoralis*)	21	-	-	S52
65	“*Trochalopteron milnei*”*	MH238447, NC_041141	Zhang et al. (2018a)	Qufu Normal University, Qufu, **China**	Mitochondrial DNA Part B	**Chimera** (*Trochalopteron milnei, Abroscopus schisticeps*)	1		-	S53
66	“*Sturnus nigricollis*”*	JQ003192	Kan, X.-Z. & Qian, C.-J., *unpublished*; 2013	Anhui Normal Univ., Wuhu, Anhui, **China**	-	**Chimera** (*Sturnus nigricollis, Acridotheres cristatellus*)	3	2	-	S54
67	“*Turdus merula*”	KT373849, NC_028188	Wen et al. *unpublished*; 2015	Leshan Normal Univ., Leshan, Sichuan, **China**	-	**Misidentification** (*Turdus mandarinus*) resulting from outdated taxonomy	4 (3)	-	-	S55
68	“*Turdus merula*”	KT601060	Peng et al. (2016c)	Nanjing Forestry Univ., Nanjing, Jiangsu, **China**	Mitochondrial DNA Part A	**Misidentification** (*Turdus mandarinus*) resulting from outdated taxonomy	1	-	-	S55
69	“*Muscicapa griseisticta*”*	MK390479, NC_045181	Liu et al. (2019g)	Nanjing Forestry Univ., Nanjing, Jiangsu, **China**	Mitochondrial DNA Part B	**Misidentification** (*Muscicapa sibirica*)	-	-	-	S56
70	“*Cyanoptila cyanomelana*”*	HQ896033, NC_015232	Kan, X.-Z. & Li, X.-F., *unpublished*; 2011	Anhui Normal Univ., Wuhu, Anhui, **China**	-	**Misidentification** (*Cyornis hainanus* or *C. rubeculoides*)	23 (1)	3	-	S57
71	“*Passer ammodendri*”*	KT895996	Fan et al. (2017)	Lanzhou Univ., Lanzhou, Gansu, **China**	Mitochondrial DNA Part A	**Chimera** (*Passer ammodendri, Emberiza godlewskii*)	5	-	1	S58
72	“*Passer montanus*”	MH211399	Huang, Z. & Tu, F., *unpublished*, 2019	Jiangxi Acad. Forestry, Nanchang, Jiangxi, **China**	-	**Misidentification** (*Passer domesticus*)	-	-	-	S59

No	Species name	GenBank Accession, NCBI reference sequence	Reference	Research group	Journal	Problem	Mitogenomic phylogenies re-using sequence (non-mitogenomic)	Other uses	Previously flagged	Gene tree(s)
73	"*Motacilla lugens*"*	KU246035, NC_029703	Park, C.E. et al., *unpublished*; 2015	Kyungpook Natl. Univ., Daegu, **South Korea**	-	**Chimera** (*Motacilla alba/lugens, Cyanopica cyanus*)	4 (1)	-	-	S60
74	"*Leucosticte arctoa*"*	KM078791, NC_025615	Lerner et al. (2011)	Smithsonian Conserv. Biology Inst., Washington DC/ Univ. Maryland, MD 20742, **USA**	Current Biology	**Misidentification** (*Leucosticte tephrocotis*) resulting from outdated taxonomy	5	-	-	S61
75	"*Haemorhous mexicanus*"	FJ236300	Carson, R.J. & Spicer, G.S., *unpublished*; 2008	San Francisco State Univ., San Francisco, CA, **USA**	-	**Misidentification** (*Haemorhous purpureus*)	-	-	-	S62
76	"*Emberiza chrysophrys*"*	HQ896034, NC_015233	Ren et al. (2014a)	Anhui Normal Univ., Wuhu, Anhui, **China**	Journal of Genetics	**Sequencing errors / numts**	12	4	-	S63
77	"*Emberiza aureola*"*	KF111713, NC_022150	Pan et al. (2015a)	Anhui Univ., Hefei, Anhui, **China**	Mitochondrial DNA	**Misidentification** (*Emberiza rutila*) + poor sequence quality of ND2	12	1	-	S63
78	"*Emberiza pallasi*"*	MK687386	Wu et al. (2019b)	Anhui Univ., Hefei, Anhui, **China**	Mitochondrial DNA Part B	**Sequencing errors / numts**	-	-	-	S64
						**TOTAL**	**373 (12)**	**51**	**8**	

TABLE S2. Details of 33 misidentified mitogenomes.

Species name	GenBank Accession, NCBI reference sequence	Identity	Cryptic species	Hybridization in the wild (McCarthy 2006)	Recent taxonomic revision of species
"Anas falcata"	KC759527, NC_023352	*Anas penelope*	-	Yes	-
"Phaethornis malaris"	KP853097, NC_030288	*Threnetes leucurus or T. niger*	-	-	-
"Rallus aquaticus"	MH229988, NC_041578	*Rallus indicus*	Yes	-	Split in 2011 (Sangster et al. 2011)
"Grus leucogeranus"	MH041490	*Grus vipio*	-	-	-
"Charadrius placidus"	KY419888	*Charadrius alexandrinus*	Yes	-	-
"Larus vegae"	KT943749	*Calonectris leucomelas*	-	-	-
"Hydrobates castro"	MH433599, NC_041251	*Hydrobates jabejabe*	Yes	?	Split in 2012 (Sangster et al. 2012)
"Hydrobates castro"	MK170187	*Hydrobates leucorhous*	Yes	-	-
"Aquila heliaca"	KU646835, NC_035806	*Aquila chrysaetos*	-	Yes (once, Romania; Corso & Forsman 2008)	-
"Otus scops"	KT340630, NC_028162	*Otus sunia*	Yes (but allopatric)	-	Split in 1990 (Sibley & Monroe 1990)
"Otus scops"	KY471456	*Otus sunia*	Yes (but allopatric)	-	Split in 1990 (Sibley & Monroe 1990)
"Glaucidium brodiei"	MF155890	*Otus sunia*	-	-	-
"Trogon viridis"	EU410490, NC_011714	*Trogon chionurus*	Yes	-	Split in 2010 (Chesser et al. 2010)
"Anthracoceros coronatus"	MF435900, NC_038152	*Anthracoceros albirostris*	-	-	Split in 1990 (Sibley & Monroe 1990)
"Megaceryle lugubris"	KY940558, NC_035658	*Ceryle rudis*	-	-	-
"Falco naumanni"	KM251414, NC_029846	*Falco tinnunculus*	-	Yes	-
"Cacatua sanguinea"	MN126573	*Psittacula krameri*	-	-	-
"Thamnophilus nigrocinereus"	KJ909192	*Percnostola rufifrons*	-	-	-
"Lanius tephronotus"	JX486029, NC_021105	*Lanius schach*	-	Yes	-
"Quoyornis georgiana"	KM374638	*Eopsaltria griseogularis*	-	-	-
"Eopsaltria griseogularis"	KM374625	*Quoyornis georgiana*	-	-	-
"Periparus ater"	KM588075, NC_026223	*Periparus rubidiventris*	-	-	-

Species name	GenBank Accession, NCBI reference sequence	Identity	Cryptic species	Hybridization in the wild (McCarthy 2006)	Recent taxonomic revision of species
"Poecile palustris"	KX388475	*Poecile weigoldicus*	Yes	-	*P. weigoldicus* was split from *P. montanus* in the 2010s; it has never been associated with *P. palustris*
"Seicercus burkii"	KX977449	*Seicercus affinis*	Yes	-	-
"Garrulax perspicillatus"	KF997865, NC_026068	*Garrulax pectoralis*	-	-	-
"Turdus merula"	KT373849, NC_028188	*Turdus mandarinus*	Yes	-	Split in 2014 (Dickinson & Christidis 2014)
"Turdus merula"	KT601060	*Turdus mandarinus*	Yes	-	Split in 2014 (Dickinson & Christidis 2014)
"Muscicapa griseisticta"	MK390479, NC_045181	*Muscicapa sibirica*	Yes	-	-
"Cyanoptila cyanomelana"	HQ896033, NC_015232	*Cyornis hainanus* or *C. rubeculoides*	-	-	-
"Passer montanus"	MH211399	*Passer domesticus*	-	Yes (hybrid males resemble *P. montanus*)	-
"Leucosticte arctoa"	KM078791, NC_025615	*Leucosticte tephrocotis*	-	-	Split in 1990 (Sibley & Monroe 1990)
"Haemorhous mexicanus"	FJ236300	*Haemorhous purpureus*	-	Yes (once)	-
"Emberiza aureola"	KF111713, NC_022150	*Emberiza rutila*	Yes (female, immature); No (male)	-	-

References

Chesser, RT, Banks, RC, Barker, FK, Cicero C, Dunn, JL, Kratter, AW, Lovette, IJ, Rasmussen, PC, Remsen, JV, Rising, JD, Stotz, DF & Winker, K 2010. Fifty-first supplement to the American Ornithologists' Union: Check-list of North American Birds. Auk 127: 726–744.

Corso, A & Forsman, D 2008. A hybrid Imperial Eagle × Golden Eagle in Romania. Birding World 21: 304-305.

Dickinson, EC & Christidis, L (editors) 2014. The Howard and Moore complete checklist of the birds of the world. Fourth edition, vol. 2: Passerines. Aves Press, London.

McCarthy, EM 2006. Handbook of Avian Hybrids of the World. Oxford, UK: Oxford University Press.

Sangster, G, Collinson, M, Crochet, P-A, Knox, AG, Parkin, DT, Svensson, L & Votier, SC 2011. Taxonomic recommendations for British birds: seventh report. Ibis 153: 883-892.

Sangster, G, Collinson, M, Crochet, P-A, Knox, AG, Parkin, DT & Votier, SC 2012. Taxonomic recommendations for British birds: eighth report. Ibis 154: 874-883.

Sibley, CG & Monroe, BL 1990. Distribution and taxonomy of birds of the world. Yale Univ. Press, New Haven.

TABLE S3. Details of 23 chimeras identified in this paper. Species names marked with an asterisk* indicate species for which no unproblematic mitogenome has been published. If homo- and heterospecific proportions do not add to 100%, the remaining sequence fragments could not be assigned to one species or the other (due to being either identical to both species of too divergent from both).

Species name	GenBank Accession, NCBI reference sequence	Detection in gene trees	Other species	Heterospecific fragment(s), bp (positions on mitogenome, excl. gaps)	Heterospecific fragment length (bp)	Heterospecific proportion of mitogenome	Homospecific proportion of mitogenome	Sequence divergence (p-distance) between chimeric fragment and focal species (or close relative)
“*Francolinus pintadeanus*”	EU165707, NC_011817	mismatch of gene trees; no close match (*cyt b*)	*Coturnix japonica*	15098-16010	913	>5.5%	<94.5%	14.2% from *F. pintadeanus* (n=1)
“*Phasianus versicolor*”*	AB164626, NC_010778	deep divergence (*cyt b*)	*Syrmaticus soemmerringii*	15232-15565	334	>2.0%	<98.0%	9.0-9.3% from *P. colchicus* (n=4)
“*Aix galericulata*”	KJ169568, NC_023969	deep divergence (*cyt b*)	*Aythya ferina*	2171-3170<br>13553-14257	1705	>10.3%	<89.7%	7.5% from *Aix galericulata* (n=1)
“*Netta rufina*”*	KC466568, NC_024922	long branch (*COI*)	*Syrmaticus reevesii*	5879-7769	1891	>11.4%	<88.6%	16.6-17.2% from *Aythya* spp. (n=3)
“*Caprimulgus jotaka*”*	KM272749, NC_025773	long branch (*cyt b*)	*Otus semitorques**	?	?	?	?	?
			*Glaucidium cuculoides**	?	?	?	?	?
“*Amaurornis akool*”*	KJ192198, NC_023982	mismatch of gene trees; no close match (*COI*)	*Gallinula chloropus*	1-406<br>619-1640<br>3931-5787<br>6384-7433<br>8474-9277<br>11580-12629<br>14448-15146<br>16214-17021	406<br>1022<br>1857<br>1050<br>804<br>1050<br>699<br>808	>45.4%	Likely 0%	17.4% from *Porzana pusilla* (n=1)
			*Amaurornis phoenicurus**	?	?	?	?	?
“*Amaurornis phoenicurus*”*	KJ874440, NC_024593	mismatch of gene trees; no close match (*cyt b, ND2*)	*Gallinula chloropus*	875-1174<br>14173-14964	1092	>6.3%	<93.7%	15.9% from *Gallirex cinerea* (n=1)
“*Vanellus cinereus*”	KM873665	long branch (*cyt b*)	*Motacilla alba*	5165-5308<br>12553-13814	144<br>1262	>8.2%	<91.8%	23.3% from *V. cinereus* (n=1)
“*Accipiter gularis*”*	KX585864	mismatch of gene trees	*Buteo buteo burmanicus*	4114-4997<br>6914-8506<br>13744-14689	884<br>1593<br>946	>19.1%	>43.2%	14.4% from *A. virgatus* (n=2)
			Streptopelia orientalis	1-2258<br>5056-6830<br>8734-9800	2258<br>1775<br>1067	>28.5%		16.6% from *A. virgatus* (n=2)
“*Tyto longimembris*”*	KP893332	mismatch of gene trees; long branch (*COI*)	*Buteo buteo burmanicus*	3276-5435	2160	>11.7%	<88.3%	22.1% from *T. alba* (n=1)
“*Otus bakkamoena*”*	KT340631, NC_028163	mismatch of gene trees	*Sternula albifrons*	2555-9890	7336	>42.2%	Likely 0%	17.8-17.9% from *O. sunia* (n=3)
			*Otus semitorques**	?	?	?	?	?

**Species name**	**GenBank Accession, NCBI reference sequence**	**Detection in gene trees**	**Other species**	**Heterospecific fragment(s), bp** (positions on mitogenome, excl. gaps)	**Heterospecific fragment length (bp)**	**Heterospecific proportion of mitogenome**	**Homospecific proportion of mitogenome**	**Sequence divergence (p-distance) between chimeric fragment and focal species (or close relative)**
"*Alcedo atthis*"*	KY964271,<br>NC_035868	long branch (*COI*)	*Gallinula chloropus*	5820-6204	385	>2.2%	<97.8%	18.3% from *Corythornis leucogaster* (n=1)
"*Ceryle rudis*"*	KJ461938,<br>NC_024280	long branch (*ND2*)	*Megaceryle lugubris**	?	?	?	?	?
"*Yungipicus canicapillus*"	MK335534	deep divergence (*cyt b*)	*Dendrocopos darjellensis**	7520-8161<br>14334-16125	642<br>1792	>14.4%	>85.0%	13.2% from *Y. canicapillus* (n=1)
"*Corvus coronoides*" *	MF370524,<br>NC_035877	long branch (*COI, cyt b*)	*Mus musculus*	5561-5852<br>11128-11230<br>12528-12643<br>14162-14377	292<br>103<br>116<br>216	4.3%	95.7%	18.7-20.6% from seven spp. of *Corvus*
"*Bombycilla cedrorum*"*	KJ909187	long branch (*COI*); no close match (*cyt b*); long branch of sister clade (*cyt b*)	*Gracula religiosa*	5548-5833<br>13813-14386	286<br>574	>5.2%	<94.8%	13.0% from *Moho braccatus* (n=1)
"*Pseudopodoces humilis*"	HM535648,<br>NC_014341	mismatch of gene trees	*Phoenicurus ochruros**	1-3913<br>4624-4693<br>4835-16745	3913<br>70<br>11911	>94.6%	Likely 0%	16.2% from *A. c. concinnus* (n=1)
			Aegithalos concinnus	3990-4604<br>4712-4825	615<br>114	>4.3%		21.8% from *P. auroreus* (n=1)
"*Garrulax albogularis*"*	KX082660,<br>NC_037464	mismatch of gene trees; no close match (*cyt b*)	*Minla ignotincta*	3-627<br>3924-3994<br>6664-7811<br>9109-10322<br>14212-15436<br>15673-16516<br>16919-17217<br>17451-17868	625<br>71<br>1148<br>1214<br>1225<br>844<br>299<br>418	>32.7%	<67.3%	14.9-15.0% from *G. sannio* (n=2)
"*Garrulax poecilorhynchus*"*	KR909134,<br>NC_028082	mismatch of gene trees	*Leiothrix lutea*	3983-5288	1306	>7.8%	<92.7%	17.1-17.2% from *G. sannio* (n=2)
"*Trochalopteron milnei*"*	MH238447,<br>NC_041141	long branch (*cyt b*)	*Abroscopus schisticeps**	?	?	?	?	?
"*Sturnus nigricollis*"	JQ003192	long branch (*COI*)	*Acridotheres cristatellus*	2664-3486<br>5134-5674	823<br>541	>8.1%	>89.7%	9.0% from *S. nigricollis* (n=1)
"*Passer ammodendri*"*	KT895996	mismatch of gene trees	*Emberiza godlewskii**	?	?	?	?	?
Motacilla lugens"*	KU246035,<br>NC_029703	mismatch of gene trees; long branch (*ND2*)	*Cyanopica cyanus*	2-2022<br>3338-3656<br>4901-5458<br>6793-7429<br>7675-7861<br>8052-8355<br>8667-9273<br>9688-10316<br>11860-12652<br>13787-14958	2021<br>319<br>558<br>637<br>187<br>304<br>607<br>629<br>793<br>1172	>42.9%	>55.7%	14.1% from *M. alba* (n=1)

TABLE S4. Detection of 18 mitogenomes with sequencing errors / numts identified in this paper.

Species name	GenBank Accession, NCBI reference sequence	Detection
Centrocercus minimus	CM016737	multiple insertions and deletions (*ND2, COI, cyt b*); long branch (*cyt b*)
Gallus gallus	KT626849	long branch (COI)
Anser fabalis	HQ890328, NC_016922	12-bp deletion (*cyt b*); long branch (*COI*)
Tadorna tadorna	MN258348	long branch (*COI, cyt b*)
Anas crecca	KF203133, NC_022452	deep divergence (*cyt b*)
Anas clypeata	KT345702, NC_028346	deep divergence (*cyt b*)
Cuculus canorus	MN067867	long branch (*COI, ND2*)
Coturnicops exquisitus	AP010823, NC_012143	deep divergence (*cyt b*)
Tringa totanus	MK922124, NC_044648	long branch (*cyt b*)
Aquila audax	MG873530	multiple deletions (*COI, cyt b, ND2*); long branch (*cyt b, ND2*); deep divergence (*COI*)
Bubo bubo	AB918148	multiple insertions (*COI, ND2*); long branch (*COI*); distant position without close match (*cyt b*)
Strix leptogrammica	KC953095, NC_021970	long branch (*cyt b*)
Ninox strenua	KX529654, NC_033967	long branch (*cyt b, ND2*)
Falco mexicanus	Dryad (https://doi.org/10.5061/dryad.8b0s04t)	long branch (*ND2*); deep divergence (*COI*)
Brotogeris cyanoptera	HM627323, NC_015530	distant position without close match (*cyt b*)
Hirundo rustica	KP148840	deep divergence (*COI, cyt b, ND2*)
Emberiza chrysophrys	HQ896034, NC_015233	distant position without close match (*COI*)
Emberiza pallasi	MK687386	long branch (*ND2*); deep divergence (*cyt b*)

TABLE S5. Applications of problematic mitogenomes and their (potential) effects

Application	Problematic mitogenome(s)	Reference	Effects
**Phylogenetic inference**	AB918148 ("*Bubo bubo*")	Spiridinova & Surmach (2018)	**Incorrect hypothesis**: species in the former genus '*Ketupa*' were inferred to be distant from *Bubo*, but this was based on a single, problematic mitogenome of the latter (AB918148). Re-analysis shows that '*Ketupa*' and *Bubo* form a single clade (**Fig. S28**).
**Taxonomy**	KJ192198, NC_023982 ("*Amaurornis akool*")	Boast et al. (2019)	**Unwarranted proposal for generic revision**: the placement of *Amaurornis akool* in the genus *Zapornia* was questioned but this was based on a chimeric mitogenome of *Amaurornis akool.*
	AB918148 ("*Bubo bubo*")	Spiridinova & Surmach (2018)	**Unwarranted proposal for generic revision**: separation of *Ketupa* from *Bubo* based on a single, problematic mitogenome of the latter (AB918148).
	HM535648, NC_014341 ("*Pseudopodoces humilis*")	Zhang et al. 2018b	**Unwarranted proposal for taxonomic rearrangement**: separation of Paridae from the superfamily Sylvioidea and placement within the superfamily Muscicapoidea. This was based on a chimeric mitogenome (HM535648) that contained a large portion of DNA from a member of Muscicapoidea.
**Establishing sequence identity**	KJ192198, NC_023982 ("*Amaurornis akool*")	Boast et al. (2019)	**Discrediting correct sequences**: Boast et al. (2019) erroneously concluded that the *Z. akool* sequences of Ruan et al. (2012) were likely to be spurious, and those of Gong et al. (2017) to be correct. In fact, the reverse is true.
**Measurement of divergence time**	EU165707, NC_011817 ("*Francolinus pintadeanus*")<br>AB164626, NC_010778 ("*Phasianus versicolor*")	Kan et al. (2010c)	**Likely overestimation of divergence times** of *Phasianus versicolor* and its sister taxon, and *Francolinus pintadeanus* and its sister taxon, due to chimeric sequences.
	AB164626, NC_010778 ("*Phasianus versicolor*")	He et al. (2009)	**Likely overestimation of divergence time** of *Phasianus versicolor* and its sister taxon.
	KJ680301 ("*Branta bernicla*")<br>HQ890328, NC_016922 ("*Anser fabalis*")<br>KJ169568, NC_023969 ("*Aix galericulata*")<br>KC466568, NC_024922 ("*Netta rufina*")	Sun et al. (2017a)	**Likely overestimation of divergence time** due to non-homologous DNA (in the mitogenome of *Branta bernicla*), chimeric DNA (in the mitogenomes of *Aix galericulata* and *Netta rufina*) and sequencing errors / numts (in the mitogenome of *Anser fabalis*).
	KJ192198, NC_023982 ("*Amaurornis akool*")<br>KJ874440, NC_024593 ("*Amaurornis phoenicurus*")<br>AP010823, NC_012143 ("*Coturnicops exquisitus*")	Chen et al. (2017)	**Likely overestimation of divergence time** due to chimeric DNA (in the mitogenomes of *Amaurornis akool* and *A. phoenicurus*) and sequencing errors / numts (in the mitogenome of *Coturnicops exquisitus*).
	KJ874440, NC_024593 ("*Amaurornis phoenicurus*")<br>AP010823, NC_012143 ("*Coturnicops exquisitus*")	Stervander et al. (2018)	**Likely overestimation of divergence time** due to chimeric DNA (in the mitogenome of *Amaurornis phoenicurus*) and sequencing errors / numts (in the mitogenome of *Coturnicops exquisitus*).
	KU646835, NC_035806 ("*Aquila heliaca*")	Knapp et al. (2019)	**Underestimation of divergence time** of *Aquila helica* and *A. chrysaetos* due to the fact that the mitogenome of "*Aquila heliaca*" is actually a misidentified *A. chrysaetos.*
**DNA identification**	KT345702, NC_028346 ("*Anas clypeata*")	Shao et al. (2019)	**Potential misidentification or false negatives** due to sequence errors / numts in the reference sequence fragment (12S rRNA).

Application	Problematic mitogenome(s)	Reference	Effects
	KT943749 ("*Larus vegae*")	Yoon et al. (2017)	**Potential misidentification or false negatives** due to misidentification of the reference sequence.
**Comparative analysis** (of the effects of body size on the substitution rate of mitochondrial genomes)	22 problematic sequences, comprising<br><br><u>9 misidentified sequences</u>:<br>KC759527, NC_023352 ("*Anas falcata*")<br>EU410490, NC_011714 ("*Trogon viridis*")<br>KJ909192 ("*Thamnophilus nigrocinereus*")<br>JX486029, NC_021105 ("*Lanius tephronotus*")<br>KM588075, NC_026223 ("*Periparus ater*")<br>KF997865, NC_026068 ("*Garrulax perspicillatus*")<br>HQ896033, NC_015232 ("*Cyanoptila cyanomelana*")<br>KM078791, NC_025615 ("*Leucosticte arctoa*")<br>KF111713, NC_022150 ("*Emberiza aureola*")<br><br><u>8 chimeric sequences</u>:<br>EU165707, NC_011817 ("*Francolinus pintadeanus*")<br>AB164626, NC_010778 ("*Phasianus versicolor*")<br>KJ169568, NC_023969 ("*Aix galericulata*")<br>KC466568, NC_024922 ("*Netta rufina*")<br>KJ874440, NC_024593 ("*Amaurornis phoenicurus*")<br>KM873665 ("*Vanellus cinereus*")<br>KJ461938, NC_024280 ("*Ceryle rudis*")<br>KJ909187 ("*Bombycilla cedrorum*")<br><br><u>5 sequences with sequence errors / numts</u>:<br>KF203133, NC_022452 ("*Anas crecca*")<br>AP010823, NC_012143 ("*Coturnicops exquisitus*")<br>HM627323, NC_015530 ("*Brotogeris cyanoptera*")<br>KP148840 ("*Hirundo rustica*")<br>HQ896034, NC_015233 ("*Emberiza chrysophrys*")	Nabholz et al. (2016)	**Compromised associations between body size and substitution rate.** Misidentified species will be associated with the wrong body mass; chimeric sequences and sequence errors / numts will result in overestimation of substitutions (artificially long terminal branches).
**Comparative analysis** (of the gene order in passerine mitogenomes)	18 problematic sequences, comprising<br><br><u>10 misidentified sequences</u>:<br>KM251414, NC_029846 ("*Falco naumanni*")<br>KJ909192 ("*Thamnophilus nigrocinereus*")<br>JX486029, NC_021105 ("*Lanius tephronotus*")<br>KM374625 ("*Eopsaltria griseogularis*")<br>KM588075, NC_026223 ("*Periparus ater*")<br>KF997865, NC_026068 ("*Garrulax perspicillatus*")	Mackiewicz et al. (2019)	**Potential errors in inferred gene order.** Misidentified sequences may have a different gene order; chimeric sequences and numts may also lead to errors in the inferred position (or length) of genes in a mitogenome.

Application	Problematic mitogenome(s)	Reference	Effects
	KT373849, NC_028188 (“*Turdus merula*”)<br>HQ896033, NC_015232 (“*Cyanoptila cyanomelana*”)<br>KM078791, NC_025615 (“*Leucosticte arctoa*”)<br>KF111713, NC_022150 (“*Emberiza aureola*”)<br><br>6 chimeric sequences:<br>MF370524,NC_035877 (“*Corvus coronoides*”)<br>KJ909187 (“*Bombycilla cedrorum*”)<br>KR909134, NC_028082 (“*Garrulax poecilorhynchus*”)<br>JQ003192 (“*Sturnus nigricollis*”)<br>KT895996 (“*Passer ammodendri*”)<br>KU246035, NC_029703 (“*Motacillla lugens*”)<br><br>2 sequences with sequence errors / numts:<br>KP148840 (“*Hirundo rustica*”)<br>HQ896034, NC_015233 (“*Emberiza chrysophrys*”)		
**Mitogenome assembly of NGS data**	KC466568, NC_024922 (“*Netta rufina*”)	Ericson et al. (2017)	**Potential errors in mitogenome assembly** of *Rhodonessa caryophyllacea* due to the chimeric nature of the reference sequence of *Netta rufina.*
	KC953095, NC_021970 (“*Strix leptogrammica*”)	Harvey et al. (2017)	**Potential errors in mitogenome assembly** of *Megascops* due to sequence errors / numts in the reference sequence of *Strix leptogrammica.*
	HQ896034, NC_015233 (“*Emberiza chrysophrys*”)	Toews et al. (2013)	**Potential errors in mitogenome assembly** of *Setophaga coronata* due to sequence errors / numts in the reference sequence of *Emberiza chrysophrys.*

References:

Boast, AP, Chapman, B, Herrera, MB, Worthy, TH, Scofield, RP, Tennyson, AJ, Houde, P, Bunce, M, Cooper, A & Mitchell, KJ 2019. Mitochondrial genomes from New Zealand’s extinct adzebills (Aves: Aptornithidae: *Aptornis*) support a sister-taxon relationship with the Afro-Madagascan Sarothruridae. Diversity 11(2), 24.

Chen, P, Han, Y, Zhu, C, Gao, B & Ruan, L 2017. Complete mitochondrial genome of *Porzana fusca* and *Porzana pusilla* and phylogenetic relationship of 16 Rallidae species. Genetica 145: 559–573.

Ericson, PGP, Qu, Y, Blom, MP, Johansson, US & Irestedt, M 2017. A genomic perspective of the pink-headed duck *Rhodonessa caryophyllacea* suggests a long history of low effective population size. Scientific Reports 7(1), 16853.

Harvey, MG, Aleixo, A, Ribas, CC & Brumfield, RT 2017. Habitat association predicts genetic diversity and population divergence in Amazonian birds. American Naturalist 190: 631-648.

He, L, Dai, B, Zeng, B, Zhang, X, Chen, B, Yue, B, & Li, J 2009. The complete mitochondrial genome of the Sichuan Hill Partridge (*Arborophila rufipectus*) and a phylogenetic analysis with related species. Gene 435 23-28.
Kan, XZ, Li, XF, Lei, ZP, Chen, L, Gao, H, Yang, ZY, Yang, JK, Guo, ZC, Yu, L, Zhang, LQ & Qian, CJ 2010c. Estimation of divergence times for major lineages of galliform birds: Evidence from complete mitochondrial genome sequences. African Journal of Biotechnology 9: 3073-3078.
Knapp, M, Thomas, JE, Haile, J, Prost, S, Ho, SY, Dussex, N, Cameron-Christie, S, Kardailsky, O, Barnett, R, Bunce, M, Gilbert, MTP & Scofield, RP 2019. Mitogenomic evidence of close relationships between New Zealand's extinct giant raptors and small-sized Australian sister-taxa. Molecular Phylogenetics and Evolution 134: 122-128.
Mackiewicz, P, Urantówka, AD, Kroczak, A & Mackiewicz, D 2019. Resolving phylogenetic relationships within Passeriformes based on mitochondrial genes and inferring the evolution of their mitogenomes in terms of duplications. Genome Biology and Evolution, 11: 2824-2849.
Nabholz, B, Lanfear, R & Fuchs, J 2016. Body mass-corrected molecular rate for bird mitochondrial DNA. Mol. Ecol. 25: 4438-4449.
Shao, X, Song, D, Huang, Q, Li, S & Yao, M 2019. Fast surveys and molecular diet analysis of carnivores based on fecal DNA and metabarcoding. Environmental Science 27: 543-556. doi: 10.17520/biods.2018214 [In Chinese.]
Spiridonova, LN & Surmach, SG 2018. Whole mitochondrial genome of Blakiston's Fish Owl *Bubo* (*Ketupa*) *blakistoni* suggests its redescription in the genus *Ketupa*. Russian Journal of Genetics 54: 369-373.
Stervander, M, Ryan, PG, Melo, M & Hansson, B 2019. The origin of the world's smallest flightless bird, the Inaccessible Island Rail *Atlantisia rogersi* (Aves: Rallidae). Molecular Phylogenetics and Evolution 130: 92-98.
Sun, Z, Pan, T, Hu, C, Sun, L, Ding, H, Wang, H, Zhang, C, Jin, H, Chang, Q, Kan, X & Zhang, B 2017a. Rapid and recent diversification patterns in Anseriformes birds: Inferred from molecular phylogeny and diversification analyses. PloS one 12(9), e0184529.
Toews, DPL, Mandic, M, Richards, JG & Irwin, DE 2013. Migration, mitochondria, and the Yellow-rumped Warbler. Evolution 68: 241-255.
Yoon, TH, Kang, HE, Lee, SR, Lee, JB, Baeck, GW, Park, H & Kim, HW 2017. Metabarcoding analysis of the stomach contents of the Antarctic Toothfish (*Dissostichus mawsoni*) collected in the Antarctic Ocean. PeerJ 5, e3977. (suppl data 2).
Zhang, H, Bai, Y, Shi, X, Sun, L, Wang, Z & Wu, X 2018b. The complete mitochondrial genomes of *Tarsiger cyanurus* and *Phoenicurus auroreus*: a phylogenetic analysis of Passeriformes. Genes & Genomics 40: 151-165.

Appendix 1. Mitogenomes (n=1876) analysed for this study.

Sequence database	Phylogeny database	Genus	species	family	GenBank#	RefSeq#	ND2 verified	COI verified	cyt b verified	erroneous sequence?	reference	submitted to GenBank	sequence released on GenBank	publication of paper	YEAR	Phylogeny (mitogenomic)	problematic sequences in tree
included	included	*Acanthis*	*flammea*	Fringillidae	KR422696	NC_027285	could not be verified: no structure	could not be verified: no structure	could not be verified: no structure	?	Li et al 2016e			2016	2016	Phylogram	*Leucosticte arctoa*
included	included	*Acanthisitta*	*chloris*	Acanthisittidae	AY325307		verified	verified	could not be verified: no cyt b	no	Harrison et al 2004			2004	2004	Phylogram	-
included	included	*Acanthiza*	*nana*	Acanthizidae	KY994589		verified	verified	verified	no	Lamb et al 2018			2018	2018	Phylogram	-
included	included	*Acanthiza*	*nana*	Acanthizidae	KY994614		verified	verified	verified	no	Lamb et al 2018			2018	2018	Phylogram	(duplicate)
included	excluded: no paper (yet)	*Accipiter*	*gentilis*	Accipitridae	AP010797	NC_011818	verified	verified	verified	no	Asai,S., Akoshima,D., Yamamoto,Y., Shigeta,Y. and Momose,H.	2008	2012	unpubl	2012	-	
included	included	*Accipiter*	*gularis*	Accipitridae	KX585864		misidentified	misidentified	misidentified	yes (chimera)	Liu et al 2017a			2017	2017	Cladogram	*Accipiter gularis, Tyto longimembris, Otus scops, Otus bakkamoena, Caprimulgus jotaka, Falco naumanni, Strix leptogrammica*
included	excluded: no paper (yet)	*Accipiter*	*nisus*	Accipitridae	KJ680300		verified	verified	verified	no	Lee,Y.J., Ryu,S.H. and Hwang,U.W.	2014	?	unpubl	2014	-	
included	included	*Accipiter*	*nisus*	Accipitridae	KM360148	NC_025580	verified	verified	verified	no	Zhang et al 2016a			2016	2016	no	
included	excluded: no paper (yet)	*Accipiter*	*soloensis*	Accipitridae	KJ680303		could not be verified: no ND2	verified	could not be verified: no cyt b	no	Lee,Y.J., Ryu,S.H. and Hwang,U.W.	2014	?	unpubl	2014	-	
included	included	*Accipiter*	*trivirgatus*	Accipitridae	MK953813	NC_045364	verified	verified	verified	no	Zhang et al 2019b		2019	2019	2019	Cladogram	*Falco naumanni*
included	included	*Accipiter*	*virgatus*	Accipitridae	KJ699124	NC_026082	could not be verified: no ND2	could not be verified: no structure	could not be verified: no cyt b	?	Song et al 2015			2015	2015	Phylogram	-
included	excluded: no paper (yet)	*Accipiter*	*virgatus*	Accipitridae	KP336714		could not be verified: no ND2	could not be verified: no structure	could not be verified: no cyt b	?	Zou,Y., Bi,X., Huang,L. and Jing,M.	2015	2015	unpubl	2015	-	
included	included	*Aceros*	*corrugatus*	Bucerotidae	HM755883		could not be verified: no ND2	could not be verified: no COI	verified	no	Pacheco et al 2011			2011	2011	Phylogram	*Trogon viridis, Brotogeris cyanoptera*
included	included	*Aceros*	*waldeni*	Bucerotidae	HQ834450	NC_015085	verified	could not be verified: no COI	could not be verified: cyt b could not be aligned	no	Sammler et al 2011			2011	2011	no	
included	excluded: no paper (yet)	*Acridotheres*	*cristatellus*	Sturnidae	JF810423	NC_015613	verified	verified	could not be verified: no cyt b	no	Kan,X.-Z. and Qian,C.-J.	2011	?	unpubl	2011	-	
included	included	*Acrocephalus*	*scirpaceus*	Acrocephalidae	AM889139	NC_010227	verified	verified	verified	no	Singh et al. 2008			2008	2008	no	

Sequence database	Phylogeny database	Genus	species	family	GenBank#	RefSeq#	ND2 verified	COI verified	cyt b verified	erroneous sequence?	reference	submitted to GenBank	sequence released on GenBank	publication of paper	YEAR	Phylogeny (mitogenomic)	problematic sequences in tree
included	included	*Acryllium*	*vulturinum*	Numididae	FJ752436	NC_014180	verified	verified	verified	no	Shen et al 2010			2010	2010	Phylogram	*Phasianus versicolor, Francolinus pintadeanus*
included	included	*Aegithalos*	*bonvaloti*	Aegithalidae	KF951087	NC_024267	verified	verified	verified	no	Wang et al 2015a			2015	2015	Phylogram	-
included	included	*Aegithalos*	*caudatus caudatus*	Aegithalidae	KF951088		could not be verified: no structure	could not be verified: no structure	verified	no	Wang et al 2015a			2015	2015	Phylogram	(duplicate)
included	included	*Aegithalos*	*concinnus concinnus*	Aegithalidae	KF951091		verified	verified	verified	no	Wang et al 2015a			2015	2015	Phylogram	(duplicate)
included	included	*Aegithalos*	*concinnus talifuensis*	Aegithalidae	KF951092		verified	verified	verified	no	Wang et al 2015a			2015	2015	Phylogram	(duplicate)
included	included	*Aegithalos*	*fuliginosus*	Aegithalidae	KF951086	NC_024266	verified	verified	verified	no	Wang et al 2015a			2015	2015	Phylogram	(duplicate)
included	included	*Aegithalos*	*glaucogularis glaucogularis*	Aegithalidae	KF951089	NC_024268	could not be verified: no structure	could not be verified: no COI	verified	no	Wang et al 2015a			2015	2015	Phylogram	(duplicate)
included	included	*Aegithalos*	*glaucogularis vinaceus*	Aegithalidae	KF951090		could not be verified: no structure	could not be verified: no COI	verified	no	Wang et al 2015a			2015	2015	Phylogram	(duplicate)
included	included	*Aegotheles*	*cristatus*	Aegothelidae	EU344979	NC_011718	could not be verified: no ND2	verified	verified	no	Pratt et al 2009			2009	2009	Phylogram	*Trogon viridis*
included	included	*Aegypius*	*monachus*	Accipitridae	KF682364	NC_022957	verified	verified	verified	no	Li et al 2015b			2015	2015	no	
included	excluded: paper publ. after 1-1-2020	*Aethia*	*cristatella*	Alcidae	MN337912	NC_045517	verified	verified	verified	no	Kim et al 2020a		2019	2020	2019	Phylogram	-
included	included	*Aethopyga*	*gouldiae*	Nectariniidae	KP772257	NC_027241	verified	could not be verified: no structure	verified	no	Wang & Liang 2016			2016	2016	Phylogram	*Cyanoptila cyanomelana, Lanius tephronotus, Emberiza aureola*
included	included	*Agapornis*	*fischeri*	Psittaculidae	MK348530		could not be verified: no ND2	could not be verified: no structure	verified	no	Liu et al 2019e			2019	2019	Phylogram	-
included	included	*Agapornis*	*lilianae*	Psittaculidae	MN481406	NC_045369	could not be verified: no ND2	could not be verified: no COI	could not be verified: no structure	?	Chen et al 2019c		2019	2019	2019	Phylogram	-
included	included	*Agapornis*	*nigrigenys*	Psittaculidae	MN481405	NC_045367	could not be verified: no ND2	could not be verified: no COI	could not be verified: no structure	?	Chen et al 2019d		2019	2019	2019	Phylogram	-
included	included	*Agapornis*	*personatus (GenBank: pullarius)*	Psittaculidae	MN481404	NC_045368	could not be verified: no ND2	could not be verified: no COI	misidentified	yes (mislabelled)	Chen et al 2019e		2019	2019	2019	Phylogram	-
included	included	*Agapornis*	*roseicollis*	Psittaculidae	EU410486	NC_011708	verified	verified	verified	no	Pratt et al 2009			2009	2009	Phylogram	(duplicate)
included	included	*Agelaioides (Molothrus)*	*badius*	Icteridae	JX516074	NC_018811	verified	verified	verified	no	Powell et al 2013			2013	2013	Cladogram	-
included	excluded: no paper (yet)	*Agelaius*	*phoeniceus*	Icteridae	FJ236289		verified	verified	verified	no	Carson,R.J. and Spicer,G.S.	2008	?	unpubl	2008	-	
included	included	*Agelaius*	*phoeniceus*	Icteridae	KM078767		verified	verified	verified	no	Lerner et al 2011			2011	2011	Cladogram	*Leucosticte arctoa*
included	included	*Agelaius*	*phoeniceus*	Icteridae	JX516062	NC_018801	verified	verified	verified	no	Powell et al 2013			2013	2013	Cladogram	(duplicate)

Sequence database	Phylogeny database	Genus	species	family	GenBank#	RefSeq#	ND2 verified	COI verified	cyt b verified	erroneous sequence?	reference	submitted to GenBank	sequence released on GenBank	publication of paper	YEAR	Phylogeny (mitogenomic)	problematic sequences in tree
included	included	*Agelasticus (Chrysomus)*	*cyanopus*	Icteridae	JX516076	NC_018813	verified	verified	verified	no	Powell et al 2013			2013	2013	Cladogram	(duplicate)
included	included	*Agelasticus (Chrysomus)*	*thilius*	Icteridae	JX516069	NC_018807	verified	verified	verified	no	Powell et al 2013			2013	2013	Cladogram	(duplicate)
included	included	*Agelasticus (Chrysomus)*	*xanthophthalmus*	Icteridae	JX516059	NC_018798	verified	could not be verified: no COI	verified	no	Powell et al 2013			2013	2013	Cladogram	(duplicate)
included	included	*Aix*	*galericulata*	Anatidae	KF437906		verified	verified	verified	no	Liu et al 2014a			2014	2014	Phylogram	*Anser fabalis*
included	included	*Aix*	*galericulata*	Anatidae	KJ169568	NC_023969	verified	verified	problematic	yes (chimera)	Meng et al 2016a			2016	2016	no	
included	included	*Akialoa*	*obscura*	Fringillidae	KU158190	NC_031349	could not be verified: no ND2	could not be verified: no structure	could not be verified: no cyt b	?	Anmarkrud & Lifjeld 2017			2017	2017	no	
included	included	*Alauda*	*arvensis*	Alaudidae	JQ322641	NC_020425	could not be verified	verified	could not be verified	no	Qian et al 2013a			2013	2013	no	
included	excluded: no paper (yet)	*Alca*	*torda*	Alcidae	CM018102		verified	verified	verified	no	Gilbert,T., Fjeldsa,J., Rhie,A., Koren,S., Phillippy,A., Howe,K., Formenti,G., Fedrigo,O. and Jarvis,E.D.	2019	2019	unpubl	2019	-	
included	excluded: paper publ. after 1-1-2020	*Alcedo*	*atthis*	Alcedinidae	KY964271	NC_035868	verified	problematic	verified	yes (chimera)	Jing et al 2020	2017	2017	2020	2017	Phylogram	*Alcedo atthis, Megaceryle lugubris, Ceryle rudis, Trogon viridis*
included	included	*Alcedo (Corythornis)*	*leucogaster*	Alcedinidae	MK060152		verified	verified	could not be verified: no cyt b	no	Tamashiro et al 2019			2019	2019	Phylogram	*Ceryle rudis, Trogon viridis*
included	included	*Alcippe*	*morrisonia hueti*	Timaliidae	KX376475		verified	verified	verified	no	Huang et al 2019a			2019	2019	Phylogram	*Garrulax perspicillatus, Garrulax poecilorhynchus*
included	included	*Alectoris*	*chukar*	Phasianidae	FJ752426	NC_020585	verified	verified	verified	no	Shen et al 2010			2010	2010	Phylogram	(duplicate)
included	included	*Alectoris*	*chukar*	Phasianidae	KY829450		verified	verified	verified	no	Zhang et al 2017b			2017	2017	Phylogram	-
included	included	*Alectoris*	*chukar potanini*	Phasianidae	KT806484		verified	verified	verified	no	Gao et al 2019a	2015		2019	2019	Cladogram	*Francolinus pintadeanus*
included	included	*Alectura*	*lathami*	Megapodiidae	AY346091	NC_007227	verified	verified	verified	no	Slack et al 2007			2007	2007	network	-
included	included	*Alopecoenas*	*salamonis*	Columbidae	KX902250	NC_031871	could not be verified: no ND2	could not be verified: no COI	could not be verified: no cyt b	?	Soares et al 2016			2016	2016	Cladogram	-
included	included	*Amaurornis*	*akool*	Rallidae	KJ192198	NC_023982	misidentified	problematic	misidentified	yes (chimera)	Gong et al 2017			2017	2017	Phylogram	*Amaurornis akool, Amaurornis phoenicurus, Coturnicops exquisitus*
included	included	*Amaurornis*	*phoenicurus*	Rallidae	KJ874440	NC_024593	could not be verified: no ND2	verified	misidentified	yes (chimera)	Gong et al 2017			2017	2017	Phylogram	(duplicate)

Sequence database	Phylogeny database	Genus	species	family	GenBank#	RefSeq#	ND2 verified	COI verified	cyt b verified	erroneous sequence?	reference	submitted to GenBank	sequence released on GenBank	publication of paper	YEAR	Phylogeny (mitogenomic)	problematic sequences in tree
included	excluded: no paper (yet)	*Amazilia*	*brevirostris*	Trochilidae	KP722043	NC_033406	dubious	could not be verified: reference COI may be misidentified	could not be verified: no cyt b	?	Ruschi,P., Souto,H.M., Jennings,W.B. and Prosdocimi,F.	2015	2016	unpubl	2016	-	
included	excluded: no paper (yet)	*Amazilia*	*milleri*	Trochilidae	KP722042	NC_033405	could not be verified: no ND2	could not be verified: no COI	could not be verified: no cyt b	?	Ruschi,P., Souto,H.M., Jennings,W.B. and Prosdocimi,F.	2015	2016	unpubl	2016	-	
included	excluded: no paper (yet)	*Amazilia*	*rondoniae*	Trochilidae	KP722041	NC_033404	could not be verified: no ND2	could not be verified: no COI	could not be verified: no cyt b	?	Ruschi,P., Souto,H.M., Jennings,W.B. and Prosdocimi,F.	2015	2016	unpubl	2016	-	
included	included	*Amazilia*	*versicolor*	Trochilidae	KF624601	NC_024156	could not be verified: one A. versicolor very close, but another more distant	could not be verified: reference COI may be misidentified	could not be verified: no cyt b	?	Prosdocimi et al 2016			2016	2016	no	
included	included	*Amazona*	*aestiva*	Psittacidae	KT361659	NC_033336	could not be verified: no structure	verified	verified	no	Lima et al 2018			2018	2018	Cladogram	*Brotogeris cyanopterus*
included	included	*Amazona*	*barbadensis barbadensis*	Psittacidae	JX524615		could not be verified: no ND2	verified	verified	no	Urantowka et al 2013b			2013	2013	no	
included	included	*Amazona*	*ochrocephala*	Psittacidae	KM611467	NC_027840	could not be verified: no structure	could not be verified: no structure	dubious	?	Eberhard & Wright 2016			2016	2016	Cladogram	*Brotogeris cyanopterus*
included	included	*Amazona*	*ventralis*	Psittacidae	KX925977	NC_034679	could not be verified: no ND2	verified	verified	no	Urantowka et al 2016e			2017	2017	no	
included	included	*Amblyramphus*	*holosericeus*	Icteridae	JX516063	NC_018802	verified	verified	verified	no	Powell et al 2013			2013	2013	Cladogram	(duplicate)
included	included	*Anas*	*acuta*	Anatidae	KF312717	NC_024631	verified	verified	verified	no	Yan et al 2015			2015	2015	no	
included	included	*Anas*	*chathamica*	Anatidae	KF562761		could not be verified: no ND2	could not be verified: no COI	could not be verified: no cyt b	?	Mitchell et al 2014b			2014	2014	no	
included	included	*Anas*	*clypeata*	Anatidae	KT345702	NC_028346	could not be verified: no structure	verified	problematic	yes (seq errors/numts)	Sun et al 2016a			2016	2016	Cladogram	*Anser fabalis, Anas clypeata, Anas crecca, Aix galericulata*
included	included	*Anas*	*crecca*	Anatidae	KF203133	NC_022452	verified	verified	problematic	yes (seq errors/numts)	Hu et al 2015			2015	2015	no	
included	included	*Anas*	*crecca*	Anatidae	KC771255		verified	verified	verified	no	Zhang et al 2017a			2017	2017	Phylogram	*Anas falcata, Anser fabalis, Aix galericulata*
included	included	*Anas*	*formosa*	Anatidae	JF730435	NC_015482	verified	verified	verified	no	Ryu & Hwang 2011			2011	2011	no	
included	included	*Anas*	*platyrhynchos*	Anatidae	EU755253		could not be verified: no structure	could not be verified: no structure	could not be verified: no structure	?	Tu et al 2012			2012	2012	no	
included	excluded: no paper (yet)	*Anas*	*platyrhynchos*	Anatidae	EU009397	NC_009684	could not be verified: no structure	could not be verified: no structure	could not be verified: no structure	?	Tu,J.F., Huang,Y.H. and Li,N.	2007	?	unpubl	2007	-	

Sequence database	Phylogeny database	Genus	species	family	GenBank#	RefSeq#	ND2 verified	COI verified	cyt b verified	erroneous sequence?	reference	submitted to GenBank	sequence released on GenBank	publication of paper	YEAR	Phylogeny (mitogenomic)	problematic sequences in tree
included	excluded: no paper (yet)	*Anas*	*platyrhynchos*	Anatidae	MH744426		could not be verified: no structure	could not be verified: no structure	could not be verified: no structure	?	Pneg,S.	2018	2018	unpubl	2018	-	
included	excluded: no paper (yet)	*Anas*	*platyrhynchos (Beijing)*	Anatidae	EU755252		could not be verified: no structure	could not be verified: no structure	could not be verified: no structure	?	Tu,J.F., Huang,Y.H. and Li,N.	2008	?	unpubl	2008	-	
included	excluded: no paper (yet)	*Anas*	*platyrhynchos (Jianchang)*	Anatidae	FJ167857		could not be verified: no structure	could not be verified: no structure	could not be verified: no structure	?	Tu,J.F., Huang,Y.H. and Li,N.	2008	?	unpubl	2008	-	
included	excluded: no paper (yet)	*Anas*	*platyrhynchos (Jinding)*	Anatidae	MF069248		could not be verified: no structure	could not be verified: no structure	could not be verified: no structure	?	Lin,R., Xiao,T. and Lin,W.	2017	2017	unpubl	2017	-	
included	included	*Anas*	*platyrhynchos (Jingxi)*	Anatidae	KJ689447		could not be verified: no structure	could not be verified: no structure	could not be verified: no structure	?	Xie et al 2016a			2016	2016	no	
included	excluded: no paper (yet)	*Anas*	*platyrhynchos (Liancheng White)*	Anatidae	MF069249		could not be verified: no structure	could not be verified: no structure	could not be verified: no structure	?	Lin,R., Xiao,T. and Lin,W.	2017	2017	unpubl	2017	-	
included	included	*Anas*	*platyrhynchos (Linwu duck)*	Anatidae	KJ637997		could not be verified: no structure	could not be verified: no structure	could not be verified: no structure	?	Tian et al 2016			2016	2016	no	
included	excluded: no paper (yet)	*Anas*	*platyrhynchos (Longsheng)*	Anatidae	KJ739616		could not be verified: no structure	could not be verified: no structure	could not be verified: no structure	?	Xie,Z., Zhang,Y., Xie,L., Liu,J., Deng,X., Xie,Z., Fan,Q., Luo,S.	2014	?	unpubl	2014	-	
included	excluded: no paper (yet)	*Anas*	*platyrhynchos (Putian Black)*	Anatidae	MF069250		could not be verified: no structure	could not be verified: no structure	could not be verified: no structure	?	Lin,R., Xiao,T. and Lin,W.	2017	2017	unpubl	2017	-	
included	included	*Anas*	*platyrhynchos (Rongshui)*	Anatidae	KJ833587		could not be verified: no structure	could not be verified: no structure	could not be verified: no structure	?	Zhang et al 2016i			2016	2016	no	
included	excluded: no paper (yet)	*Anas*	*platyrhynchos (Shan partridge)*	Anatidae	MF069251		could not be verified: no structure	could not be verified: no structure	could not be verified: no structure	?	Lin,R., Xiao,T. and Lin,W.	2017	2017	unpubl	2017	-	
included	excluded: no paper (yet)	*Anas*	*platyrhynchos (Shaoxing)*	Anatidae	HM010684		could not be verified: no structure	could not be verified: no structure	could not be verified: no structure	?	Tu,J.F.	2010	?	unpubl	2010	-	
included	included	*Anas*	*platyrhynchos (Shengjin Lake)*	Anatidae	MK770342		could not be verified: no structure	could not be verified: no structure	could not be verified: no structure	?	Liu et al 2019f		2019	2019	2019	Cladogram	-
included	excluded: no paper (yet)	*Anas*	*platyrhynchos (Sichuan)*	Anatidae	KX592536		could not be verified: no structure	could not be verified: no structure	could not be verified: no structure	?	Xie,A.	2016	2016	unpubl	2016	-	
included	included	*Anas*	*platyrhynchos (Xilin)*	Anatidae	KJ833586		could not be verified: no structure	could not be verified: no structure	could not be verified: no structure	?	Xie et al 2016c			2016	2016	no	
included	included	*Anas*	*platyrhynchos (Youxian duck)*	Anatidae	KJ778676		could not be verified: no structure	could not be verified: no structure	could not be verified: no structure	?	He et al 2016b			2016	2016	Phylogram	*Anas falcata, Anas crecca, Anser fabalis*
included	included	*Anas*	*platyrhynchos (Youxian duck)*	Anatidae	KJ883269		could not be verified: no structure	could not be verified: no structure	could not be verified: no structure	?	He et al 2016c			2016	2016	no	

Sequence database	Phylogeny database	Genus	species	family	GenBank#	RefSeq#	ND2 verified	COI verified	cyt b verified	erroneous sequence?	reference	submitted to GenBank	sequence released on GenBank	publication of paper	YEAR	Phylogeny (mitogenomic)	problematic sequences in tree
included	excluded: no paper (yet)	*Anas*	*poecilorhyncha*	Anatidae	KC466567		could not be verified: no structure	could not be verified: no structure	could not be verified: no structure	?	Kan,X.-Z. and Li,X.-X.	2013	?	unpubl	2013	-	
included	excluded: no paper (yet)	*Anas*	*poecilorhyncha*	Anatidae	KF751616		could not be verified: no structure	could not be verified: no structure	could not be verified: no structure	?	Mu,C.	2013	?	unpubl	2013	-	
included	included	*Anas*	*poecilorhyncha*	Anatidae	KF156760	NC_022418	could not be verified: no structure	could not be verified: no structure	could not be verified: no structure	?	Zhou et al 2015a			2015	2015	no	
included	included	*Anas (Mareca)*	*falcata*	Anatidae	KC759527	NC_023352	misidentified	misidentified	misidentified	yes (misID)	Pan et al 2014			2014	2014	no	
included	included	*Anas (Mareca)*	*strepera*	Anatidae	MN186586	NC_045373	verified	verified	verified	no	Wei et al 2019		2019	2019	2019	Phylogram	*Anas crecca, Netta rufina*
included	included	*Anhinga*	*rufa*	Phalacrocoracidae	GU071055		could not be verified: no ND2	could not be verified: no COI	could not be verified: no cyt b	?	Gibb et al 2013			2013	2013	Phylogram	-
included	included	*Anser*	*albifrons*	Anatidae	AF363031	NC_004539	could not be verified: no structure	could not be verified: no structure	verified	no	Slack et al 2003			2003	2003	network	-
included	included	*Anser*	*albifrons frontalis*	Anatidae	MH000287	NC_039888	could not be verified: no structure	could not be verified: no structure	verified	no	Fu et al 2018			2018	2018	Phylogram	*Anas falcata*
included	excluded: no paper (yet)	*Anser*	*anser*	Anatidae	MN122908		could not be verified: no structure	verified	verified	no	Margaryan,A.		2019	unpubl	2019	-	
included	excluded: no paper (yet)	*Anser*	*anser*	Anatidae	EU932689	NC_011196	could not be verified: no structure	could not be verified: no structure	verified	no	Wang,C.M., Kao,J.Y., Chen,L.R., Chung,Y.C., Yen,N.T., Hu,C.L., Nien,P.C. and Jea,Y.S.	2008	?	unpubl	2008	-	
included	included	*Anser*	*anser (Landes)*	Anatidae	MK133021		could not be verified: no structure	could not be verified: no structure	verified	no	Lin et al 2019b			2019	2019	Phylogram	*Anas falcata, Anas crecca, Anser fabalis*
included	included	*Anser*	*cygnoides (Daozhou)*	Anatidae	KU211647		could not be verified: no structure	verified	verified	no	Lin et al 2018			2018	2018	Phylogram	*Anas falcata, Anas crecca, Anser fabalis*
included	excluded: no paper (yet)	*Anser*	*cygnoides (Hepu)*	Anatidae	KP943133		could not be verified: no structure	verified	verified	no	Zhang,Y.F., Xie,Z.X., Liu,J.B., Deng,X.W., Xie,Z.Q., Huang,L., Huang,J.L., Zeng,T.T. and Wang,S.	2015	2015	unpubl	2015	-	
included	included	*Anser*	*cygnoides (Mayang white)*	Anatidae	MK102803		could not be verified: no structure	verified	verified	no	Lin et al 2019a			2019	2019	Phylogram	*Anas falcata, Anas crecca, Anser fabalis*
included	included	*Anser*	*cygnoides (Sichuan white)*	Anatidae	MK133022		could not be verified: no structure	verified	verified	no	Lin et al 2019c			2019	2019	Phylogram	*Anas falcata, Anas crecca, Anser fabalis*
included	included	*Anser*	*cygnoides (wild)*	Anatidae	KY767671		could not be verified: no structure	verified	verified	no	Lee et al 2017a			2017	2017	Phylogram	-

Sequence database	Phylogeny database	Genus	species	family	GenBank#	RefSeq#	ND2 verified	COI verified	cyt b verified	erroneous sequence?	reference	submitted to GenBank	sequence released on GenBank	publication of paper	YEAR	Phylogeny (mitogenomic)	problematic sequences in tree
included	included	*Anser*	*cygnoides (wild)*	Anatidae	KP238480		could not be verified: no structure	dubious	dubious	dubious	Zhu et al 2016			2016	2016	no	
included	included	*Anser*	*cygnoides (Wugangtong Grey)*	Anatidae	KJ794189		could not be verified: no structure	verified	verified	no	Lin et al 2016c			2016	2016	no	
included	included	*Anser*	*cygnoides (Wugangtong White)*	Anatidae	KP026178		could not be verified: no structure	verified	verified	no	Jiang et al 2016			2016	2016	no	
included	included	*Anser*	*cygnoides (Xupu)*	Anatidae	KJ794188		could not be verified: no structure	verified	verified	no	Lin et al 2016b			2016	2016	no	
included	included	*Anser*	*cygnoides (Yanling White)*	Anatidae	KJ778677		could not be verified: no structure	verified	verified	no	Lin et al 2016d			2016	2016	no	
included	excluded: no paper (yet)	*Anser*	*cygnoides (Youjiang)*	Anatidae	KP881611		could not be verified: no structure	verified	verified	no	Zhang, Y.F., Xie, Z.X., Liu, J.B., Deng, X.W., Xie, Z.Q., Huang, L., Huang, J.L., Zeng, T.T. and Wang, S.	2015	2015	unpubl	2015	-	
included	included	*Anser*	*cygnoides (Zhedong White)*	Anatidae	KT427463		could not be verified: no structure	verified	verified	no	Ren et al 2016d			2016	2016	no	
included	included	*Anser*	*cygnoides (zoo animal)*	Anatidae	KJ124555	NC_023832	could not be verified: no structure	dubious	dubious	dubious	Mu et al 2014 (Liu et al 2016)			2014	2014	Cladogram	*Anas falcata, Anser fabalis*
included	included	*Anser*	*fabalis*	Anatidae	HQ890328	NC_016922	could not be verified: no structure	could not be verified: no structure	problematic	yes (seq errors/numts)	Liu et al 2013			2013	2013	Cladogram	*Anser fabalis*
included	included	*Anser*	*indicus*	Anatidae	KM455570	NC_025654	could not be verified: no structure	could not be verified: no structure	verified	no	Mu et al 2016			2016	2016	no	
included	included	*Anseranas*	*semipalmata*	Anatidae	AY309455	NC_005933	verified	could not be verified: sequence contains too little COI	could not be verified: no cyt b	no	Harrison et al 2004			2004	2004	Phylogram	(duplicate)
included	included	*Anthornis*	*melanura*	Meliphagidae	KC545408		could not be verified: too little ND2 in seq	could not be verified: sequence does not contain COI	could not be verified: too little cyt b in seq	?	Gibb et al 2015			2015	2015	Phylogram	*Lanius tephronotus, Pseudopodoces humilis, Cyanoptila cyanomelana, Emberiza aureola*
included	included	*Anthracoceros*	*coronatus*	Bucerotidae	MF435900	NC_038152	could not be verified: no ND2	could not be verified: no COI	misidentified	yes (taxonomy)	Li et al 2018			2018	2018	no	
included	included	*Anthus*	*hodgsoni*	Motacillidae	KX189345		verified	verified	verified	no	Sun et al 2016b			2016	2016	no	
included	included	*Anthus*	*novaeseelandiae*	Motacillidae	KC545397	NC_029137	could not be verified: no ND2	verified	could not be verified: no cyt b	no	Gibb et al 2015			2015	2015	Phylogram	(duplicate)

Sequence database	Phylogeny database	Genus	species	family	GenBank#	RefSeq#	ND2 verified	COI verified	cyt b verified	erroneous sequence?	reference	submitted to GenBank	sequence released on GenBank	publication of paper	YEAR	Phylogeny (mitogenomic)	problematic sequences in tree
included	included	*Anthus*	*richardi*	Motacillidae	MH593382	NC_041109	could not be verified: no ND2	verified	verified	no	Wang et al 2019a			2019	2019	Cladogram	*Motacilla lugens*
included	included	*Apaloderma*	*narina*	Trogonidae	MK060122		verified	could not be verified: no COI	verified	no	Tamashiro et al 2019			2019	2019	Phylogram	(duplicate)
included	included	*Aptenodytes*	*forsteri*	Spheniscidae	KT159230	NC_027938	could not be verified: no ND2	verified	verified	no	Xu et al 2016a			2016	2016	Cladogram	*Pseudopodoces humilis*
included	included	*Aptenodytes*	*patagonicus*	Spheniscidae	MK290241		verified	verified	verified	no	Cole et al 2019			2019	2019	Cladogram	-
included	included	*Aptenodytes*	*patagonicus*	Spheniscidae	MK801135	NC_045377	verified	verified	verified	no	Du et al 2019		2019	2019	2019	Phylogram	-
included	excluded: no paper (yet)	*Apteryx*	*australis mantelli*	Palaeognathae	LK054805		could not be verified: too few sequences of Palaeognathae	verified	verified	no	Prufer, K, Dannemann, M, Kelso, J, LeDuc, D	2014	?	unpubl	2014	-	
included	included	*Apteryx*	*haastii*	Palaeognathae	AF338708	NC_002782	could not be verified: too few sequences of Palaeognathae	verified	verified	no	Haddrath & Baker 2001			2001	2001	Phylogram	-
included	included	*Apteryx*	*mantelli*	Palaeognathae	AY016010		could not be verified: too few sequences of Palaeognathae	verified	verified	no	Cooper et al 2001			2001	2001	Phylogram	-
included	included	*Apteryx*	*mantelli*	Palaeognathae	KU695537		could not be verified: too few sequences of Palaeognathae	verified	verified	no	Liu et al 2017b			2017	2017	Phylogram	-
included	included	*Apteryx*	*owenii*	Palaeognathae	GU071052	NC_013806	could not be verified: too few sequences of Palaeognathae	verified	verified	no	Phillips et al 2010			2010	2010	Phylogram	-
included	included	*Aptornis*	*defossor*	Rallidae	MK434264		could not be verified: no ND2	could not be verified: no COI	could not be verified: no cyt b	?	Boast et al 2019			2019	2019	Phylogram	(duplicate)
included	included	*Aptornis*	*otidiformis*	Rallidae	MK434262		could not be verified: no ND2	could not be verified: no COI	could not be verified: no cyt b	?	Boast et al 2019			2019	2019	Phylogram	(duplicate)
included	included	*Aptornis*	*otidiformis*	Rallidae	MK434265		could not be verified: no ND2	could not be verified: no COI	could not be verified: no cyt b	?	Boast et al 2019			2019	2019	Phylogram	(duplicate)
included	included	*Apus*	*apus*	Apodidae	AM237310	NC_008540	verified	verified	verified	no	Morgan-Richards et al 2008			2008	2008	Phylogram	-
included	included	*Aquila*	*audax*	Accipitridae	MK453378		verified	could not be verified: no COI	verified	no	Grealy et al 2019			2019	2019	Phylogram	-
included	included	*Aquila*	*audax*	Accipitridae	MG873530		verified	problematic	problematic	yes (seq errors/numts)	Sarker et al 2019			2019	2019	Cladogram	*Aquila heliaca, Aquila audax*
included	included	*Aquila*	*chrysaetos*	Accipitridae	KF905228	NC_024087	verified	verified	verified	no	Doyle et al 2014			2014	2014	no	

Sequence database	Phylogeny database	Genus	species	family	GenBank#	RefSeq#	ND2 verified	COI verified	cyt b verified	erroneous sequence?	reference	submitted to GenBank	sequence released on GenBank	publication of paper	YEAR	Phylogeny (mitogenomic)	problematic sequences in tree
included	included	*Aquila*	*fasciata*	Accipitridae	KP329567	NC_029188	verified	verified	verified	no	Jiang et al 2015			2015	2015	Phylogram	*Strix leptogrammica*
included	excluded: no paper (yet)	*Aquila*	*heliaca*	Accipitridae	KU646835	NC_035806	misidentified	misidentified	misidentified	yes (misID)	Zhou, L. and Dong, Y.	2016	2017	unpubl	2017	-	
included	included	*Aquila*	*nipalensis*	Accipitridae	MK860035	NC_045042	verified	verified	verified	no	Zhou et al 2019c		2019	2019	2019	Phylogram	*Aquila heliaca*
included	included	*Aquila (Harpagornis)*	*moorei*	Accipitridae	MK294166		could not be verified: no ND2	could not be verified: no COI	verified	no	Knapp et al 2019			2019	2019	Cladogram	(duplicate)
included	included	*Aquila (Hieraaetus)*	*morphnoides*	Accipitridae	MK294164		verified	could not be verified: no COI	verified	no	Knapp et al 2019			2019	2019	Cladogram	(duplicate)
included	included	*Aquila (Hieraaetus)*	*pennatus*	Accipitridae	MK294165		verified	could not be verified: no COI	verified	no	Knapp et al 2019			2019	2019	Cladogram	(duplicate)
included	included	*Ara*	*ararauna*	Psittacidae	KF010315	NC_029319	verified	verified	verified	no	Urantowka et al 2017a			2016	2016	no	
included	included	*Ara*	*glaucogularis*	Psittacidae	JQ782215	NC_026029	verified	verified	could not be verified: no cyt b	no	Urantowka 2016b			2016	2016	no	
included	excluded: paper publ. after 1-1-2020	*Ara*	*macao*	Psittacidae	MK351783	NC_045076	verified	verified	verified	no	Schmidt et al 2020	2019	2019	2020	2019	Phylogram	-
included	excluded: paper publ. after 1-1-2020	*Ara*	*macao*	Psittacidae	MK351784		verified	verified	verified	no	Schmidt et al 2020	2019	2019	2020	2019	Phylogram	(duplicate)
included	included	*Ara*	*macao*	Psittacidae	CM002021		verified	verified	verified	no	Seabury et al 2013			2013	2013	no	
included	included	*Ara*	*militaris*	Psittacidae	KM611466	NC_027839	verified	could not be verified: no COI	verified	no	Eberhard & Wright 2016			2016	2016	Cladogram	(duplicate)
included	included	*Ara*	*militaris mexicanus*	Psittacidae	JX524613		verified	could not be verified: no COI	verified	no	Urantowka 2016d			2016	2016	no	
included	included	*Ara*	*severus*	Psittacidae	KF946546		verified	verified	verified	no	Urantowka et al 2017c			2017	2017	no	
included	included	*Ara*	*tricolor*	Psittacidae	MG432916	NC_037895	could not be verified: no ND2	could not be verified: no COI	could not be verified: no cyt b	?	Johansson et al 2018a			2018	2018	Cladogram	*Brotogeris cyanopterus*
included	included	*Ara*	*tricolor*	Psittacidae	MG432917		could not be verified: no ND2	could not be verified: no COI	could not be verified: no cyt b	?	Johansson et al 2018a			2018	2018	Cladogram	(duplicate)
included	included	*Aramus*	*guarauna*	Rallidae	MK434260		could not be verified: no ND2	verified	verified	no	Boast et al 2019			2019	2019	Phylogram	(duplicate)
included	included	*Aratinga*	*nenday*	Psittacidae	MK965540	NC_045371	verified	verified	verified	no	Liu et al 2019l		2019	2019	2019	Phylogram	-
included	included	*Aratinga*	*solstitialis*	Psittacidae	MK343132		verified	verified	verified	no	Liu et al 2019j		2019	2019	2019	Phylogram	-
included	included	*Aratinga*	*solstitialis*	Psittacidae	JX441869	NC_026039	verified	verified	could not be verified: no structure	no	Urantowka et al 2016a			2016	2016	no	
included	included	*Aratinga (Eupsittula)*	*pertinax chrysogenys*	Psittacidae	HM640208	NC_015197	verified	verified	verified	no	Pacheco et al 2011			2011	2011	Phylogram	(duplicate)

Sequence database	Phylogeny database	Genus	species	family	GenBank#	RefSeq#	ND2 verified	COI verified	cyt b verified	erroneous sequence?	reference	submitted to GenBank	sequence released on GenBank	publication of paper	YEAR	Phylogeny (mitogenomic)	problematic sequences in tree
included	included	*Arborophila*	*ardens*	Phasianidae	KJ716444		verified	verified	verified	no	Li et al 2014a			2014	2014	Phylogram	*Phasianus versicolor, Francolinus pintadeanus*
included	included	*Arborophila*	*ardens*	Phasianidae	KC352729	NC_022683	verified	verified	verified	no	Yan et al 2014			2014	2014	Phylogram	*Phasianus versicolor, Francolinus pintadeanus*
included	included	*Arborophila*	*brunneopectus*	Phasianidae	KC352730	NC_022684	verified	verified	verified	no	Yan et al 2017			2017	2017	Phylogram	*Phasianus versicolor, Francolinus pintadeanus*
included	included	*Arborophila*	*gingica*	Phasianidae	FJ752425		verified	verified	verified	no	Shen et al 2010			2010	2010	Phylogram	(duplicate)
included	included	*Arborophila*	*rufipectus*	Phasianidae	FJ194942	NC_012453	verified	verified	verified	no	He et al 2009			2009	2009	Phylogram	*Phasianus versicolor*
included	included	*Arborophila*	*rufogularis*	Phasianidae	FJ752424	NC_020584	verified	verified	verified	no	Shen et al 2010			2010	2010	Phylogram	(duplicate)
included	included	*Archilochus*	*colubris*	Trochilidae	EF532935	NC_010094	verified	verified	verified	no	Morgan-Richards et al 2008			2008	2008	Phylogram	(duplicate)
included	included	*Ardea*	*cinerea*	Ardeidae	KJ190947	NC_025900	could not be verified: no ND2	verified	verified	no	Zhou et al 2014b			2014	2014	Phylogram	-
included	included	*Ardea*	*insignis*	Ardeidae	MH737740	NC_040004	could not be verified: no ND2	could not be verified: no COI	could not be verified: no cyt b	?	Duan et al 2018a			2018	2018	Phylogram	-
included	included	*Ardea*	*intermedia*	Ardeidae	KX592585		could not be verified: no ND2	verified	verified	no	Tu et al 2017			2017	2017	Phylogram	-
included	included	*Ardea*	*intermedia*	Ardeidae	KJ190946	NC_025918	could not be verified: no ND2	verified	verified	no	Zhou et al 2014b			2014	2014	Phylogram	(duplicate)
included	included	*Ardea*	*modesta*	Ardeidae	KJ190944	NC_025916	could not be verified: no ND2	verified	could not be verified: no cyt b	no	Zhou et al 2014b			2014	2014	Phylogram	(duplicate)
included	included	*Ardea*	*novaehollandiae*	Ardeidae	DQ780878	NC_008551	could not be verified: no ND2	verified	could not be verified: no cyt b	no	Gibb et al 2007			2007	2007	Phylogram	-
included	included	*Ardea*	*purpurea*	Ardeidae	KJ190948	NC_025919	could not be verified: no ND2	verified	verified	no	Zhou et al 2014b			2014	2014	Phylogram	(duplicate)
included	included	*Ardeola*	*bacchus*	Ardeidae	KJ190952	NC_025921	could not be verified: no ND2	verified	could not be verified: no cyt b	no	Zhou et al 2014b			2014	2014	Phylogram	(duplicate)
included	included	*Arenaria*	*interpres*	Scolopacidae	AY074885	NC_003712	could not be verified: no ND2	verified	verified	no	Paton et al 2002			2002	2002	Phylogram	-
included	included	*Argusianus*	*argus*	Phasianidae	JQ713768		verified	verified	verified	no	Shen et al 2014			2014	2014	Phylogram	*Phasianus versicolor, Francolinus pintadeanus*
included	included	*Argusianus*	*argus*	Phasianidae	KY411590		verified	verified	verified	no	Wang et al 2017a			2017	2017	Phylogram	-
included	included	*Arremon*	*aurantiirostris*	Passerellidae	KR780063	NC_027731	verified	verified	verified	no	Lopez et al 2015			2015	2015	no	
included	included	*Arremon*	*aurantiirostris*	Passerellidae	KR780064		verified	verified	verified	no	Lopez et al 2015			2015	2015	no	

Sequence database	Phylogeny database	Genus	species	family	GenBank#	RefSeq#	ND2 verified	COI verified	cyt b verified	erroneous sequence?	reference	submitted to GenBank	sequence released on GenBank	publication of paper	YEAR	Phylogeny (mitogenomic)	problematic sequences in tree
included	included	*Artamus*	*cinereus*	Artamidae	MF784400	NC_041197	verified	could not be verified: no COI	could not be verified: no cyt b	no	Quan et al 2018			2018	2018	Phylogram	-
included	included	*Artamus*	*cyanopterus*	Artamidae	KY994603		verified	could not be verified: no COI	could not be verified: no cyt b	no	Lamb et al 2018			2018	2018	Phylogram	(duplicate)
included	included	*Artamus*	*cyanopterus*	Artamidae	KY994613		verified	could not be verified: no COI	could not be verified: no cyt b	no	Lamb et al 2018			2018	2018	Phylogram	(duplicate)
included	included	*Asio*	*flammeus*	Strigidae	KP889214	NC_027606	verified	verified	verified	no	Zhang et al 2016b			2016	2016	no	
included	included	*Asio*	*otus*	Strigidae	MG916810	NC_039736	verified	verified	verified	no	Lee et al 2018			2018	2018	Phylogram	*Otus bakkamoena, Otus scops, Strix leptogrammica, Ninox strenua*
included	included	*Athene*	*brama*	Strigidae	KF961185		could not be verified: no ND2	verified	verified	no	Mahmood et al 2014			2014	2014	Phylogram	-
included	included	*Atlantisia*	*rogersi*	Rallidae	MH029238	NC_039814	could not be verified: no ND2	could not be verified: no COI	could not be verified: no cyt b	?	Stervander et al 2018			2018	2018	Cladogram	*Amaurornis phoenicurus, Coturnicops exquisitus*
included	included	*Aythya*	*americana*	Anatidae	AF090337	NC_000877	could not be verified: no ND2	verified	could not be verified: no cyt b	no	Mindell et al 1999			1999	1999	Phylogram	-
included	included	*Aythya*	*ferina*	Anatidae	KJ710708	NC_024602	verified	could not be verified: no structure	verified	no	Zhou et al 2016d			2016	2016	no	
included	included	*Aythya*	*fuligula*	Anatidae	KJ722069	NC_024595	verified	verified	verified	no	Ding et al 2016a			2016	2016	no	
included	included	*Babax*	*lanceolatus*	Timaliidae	KR818090		verified	verified	verified	no	Qi et al 2016a			2016	2016	Phylogram	*Garrulax perspicillatus*
included	included	*Balaeniceps*	*rex*	Pelecanidae	GU071053		verified	verified	verified	no	Gibb et al 2013			2013	2013	Phylogram	(duplicate)
included	included	*Balearica*	*pavonina*	Gruidae	FJ769842	NC_020570	could not be verified: no ND2	verified	verified	no	Krajewski et al 2010			2010	2010	Phylogram	-
included	included	*Balearica*	*regulorum*	Gruidae	FJ769841	NC_020569	could not be verified: no ND2	verified	verified	no	Krajewski et al 2010			2010	2010	Phylogram	(duplicate)
included	included	*Bambusicola*	*fytchii*	Phasianidae	FJ752423	NC_020583	could not be verified: no ND2	could not be verified: reference COI may be misidentified	verified	no	Shen et al 2010			2010	2010	Phylogram	(duplicate)
included	included	*Bambusicola*	*thoracica*	Phasianidae	EU165706	NC_011816	verified	could not be verified: no COI	verified	no	Shen et al 2009			2009	2009	Cladogram	*Francolinus pintadeanus*

Sequence database	Phylogeny database	Genus	species	family	GenBank#	RefSeq#	ND2 verified	COI verified	cyt b verified	erroneous sequence?	reference	submitted to GenBank	sequence released on GenBank	publication of paper	YEAR	Phylogeny (mitogenomic)	problematic sequences in tree
included	included	*Bombycilla*	*cedrorum*	Bombycillidae	KJ909187		verified	problematic	misidentified	yes (chimera)	Barker 2014			2014	2014	Cladogram	*Lanius tephronotus, Pseudopodoces humilis, Cyanoptila cyanomelana, Bombycilla cedrorum, Thamnophilus nigrocinereus*
included	included	*Botaurus*	*stellaris*	Ardeidae	KJ190955	NC_025923	could not be verified: no ND2	could not be verified: no structure	verified	no	Zhou et al 2014b			2014	2014	Phylogram	(duplicate)
included	included	*Brachygalba*	*albogularis*	Galbulidae	MK060123		could not be verified: no ND2	could not be verified: no COI	could not be verified: no cyt b	?	Tamashiro et al 2019			2019	2019	Phylogram	(duplicate)
included	excluded: no paper (yet)	*Branta*	*bernicla*	Anatidae	KJ680301		verified	verified	problematic	yes (incorrect assembly)	Lee, Y.J., Ryu, S.H. and Hwang, U.W.	2014	?	unpubl	2014	-	
included	included	*Branta*	*canadensis*	Anatidae	DQ019124	NC_007011	verified	verified	verified	no	Snyder et al 2015			2015	2015	no	
included	included	*Brotogeris*	*cyanoptera*	Psittacidae	HM627323	NC_015530	verified	verified	problematic	yes (seq errors/numts)	Pacheco et al 2011			2011	2011	Phylogram	(duplicate)
included	included	*Bubo*	*blakistoni*	Strigidae	LC099101		could not be verified: no ND2	verified	verified	no	Spiridonova & Surmach 2018			2018	2018	Phylogram	*Tyto longimembris, Bubo bubo, Otus bakkamoena, Otus scops, Strix leptogrammica*
included	included	*Bubo*	*blakistoni*	Strigidae	LC099102		could not be verified: no ND2	verified	verified	no	Spiridonova & Surmach 2018			2018	2018	Phylogram	(duplicate)
included	included	*Bubo*	*blakistoni*	Strigidae	LC099103		could not be verified: no ND2	verified	verified	no	Spiridonova & Surmach 2018			2018	2018	Phylogram	(duplicate)
included	included	*Bubo*	*blakistoni*	Strigidae	LC099104		could not be verified: no ND2	verified	verified	no	Spiridonova & Surmach 2018			2018	2018	Phylogram	(duplicate)
included	included	*Bubo*	*blakistoni*	Strigidae	LC099105		could not be verified: no ND2	verified	verified	no	Spiridonova & Surmach 2018			2018	2018	Phylogram	(duplicate)
included	included	*Bubo*	*blakistoni*	Strigidae	LC099106		could not be verified: no ND2	verified	verified	no	Spiridonova & Surmach 2018			2018	2018	Phylogram	(duplicate)
included	included	*Bubo*	*blakistoni*	Strigidae	LC099107		could not be verified: no ND2	verified	verified	no	Spiridonova & Surmach 2018			2018	2018	Phylogram	(duplicate)
included	included	*Bubo*	*blakistoni*	Strigidae	LT671412		could not be verified: no ND2	verified	verified	no	Spiridonova & Surmach 2018			2018	2018	Phylogram	(duplicate)
included	included	*Bubo*	*blakistoni*	Strigidae	LT671413		could not be verified: no ND2	verified	verified	no	Spiridonova & Surmach 2018			2018	2018	Phylogram	(duplicate)

Sequence database	Phylogeny database	Genus	species	family	GenBank#	RefSeq#	ND2 verified	COI verified	cyt b verified	erroneous sequence?	reference	submitted to GenBank	sequence released on GenBank	publication of paper	YEAR	Phylogeny (mitogenomic)	problematic sequences in tree
included	included	*Bubo*	*blakistoni*	Strigidae	LT671414		could not be verified: no ND2	verified	verified	no	Spiridonova & Surmach 2018			2018	2018	Phylogram	(duplicate)
included	included	*Bubo*	*bubo*	Strigidae	MG681083	NC_038219	verified	verified	verified	no	Kang et al 2018			2018	2018	Phylogram	*Tyto longimembris, Otus bakkamoena, Otus scops, Strix leptogrammica, Ninox strenua*
included	excluded: no paper (yet)	*Bubo*	*bubo*	Strigidae	MK656273		verified	verified	verified	no	Meng,M.	2019	2019	unpubl	2019	-	
included	excluded: no paper (yet)	*Bubo*	*bubo*	Strigidae	MK656274		verified	verified	verified	no	Meng,M.	2019	2019	unpubl	2019	-	
included	excluded: no paper (yet)	*Bubo*	*bubo*	Strigidae	MK656275		verified	verified	verified	no	Meng,M.	2019	2019	unpubl	2019	-	
included	excluded: no paper (yet)	*Bubo*	*bubo*	Strigidae	MK656276		verified	verified	verified	no	Meng,M.	2019	2019	unpubl	2019	-	
included	excluded: no paper (yet)	*Bubo*	*bubo*	Strigidae	MK656277		verified	verified	verified	no	Meng,M.	2019	2019	unpubl	2019	-	
included	excluded: no paper (yet)	*Bubo*	*bubo*	Strigidae	MK656278		verified	verified	verified	no	Meng,M.	2019	2019	unpubl	2019	-	
included	excluded: no paper (yet)	*Bubo*	*bubo*	Strigidae	MK656279		verified	verified	verified	no	Meng,M.	2019	2019	unpubl	2019	-	
included	excluded: no paper (yet)	*Bubo*	*bubo*	Strigidae	MK656280		verified	verified	verified	no	Meng,M.	2019	2019	unpubl	2019	-	
included	excluded: no paper (yet)	*Bubo*	*bubo*	Strigidae	MK656281		verified	verified	verified	no	Meng,M.	2019	2019	unpubl	2019	-	
included	excluded: no paper (yet)	*Bubo*	*bubo*	Strigidae	MK656282		verified	verified	verified	no	Meng,M.	2019	2019	unpubl	2019	-	
included	excluded: no paper (yet)	*Bubo*	*bubo*	Strigidae	MK656283		verified	verified	verified	no	Meng,M.	2019	2019	unpubl	2019	-	
included	excluded: no paper (yet)	*Bubo*	*bubo*	Strigidae	MK656284		verified	verified	verified	no	Meng,M.	2019	2019	unpubl	2019	-	
included	excluded: no paper (yet)	*Bubo*	*bubo*	Strigidae	MK656285		verified	verified	verified	no	Meng,M.	2019	2019	unpubl	2019	-	
included	excluded: no paper (yet)	*Bubo*	*bubo*	Strigidae	MK656286		verified	verified	verified	no	Meng,M.	2019	2019	unpubl	2019	-	

Sequence database	Phylogeny database	Genus	species	family	GenBank#	RefSeq#	ND2 verified	COI verified	cyt b verified	erroneous sequence?	reference	submitted to GenBank	sequence released on GenBank	publication of paper	YEAR	Phylogeny (mitogenomic)	problematic sequences in tree
included	excluded: no paper (yet)	*Bubo*	*bubo*	Strigidae	MK656287		verified	verified	verified	no	Meng,M.	2019	2019	unpubl	2019	-	
included	included	*Bubo*	*bubo*	Strigidae	AB918148		verified	verified	problematic	yes (seq errors/numts)	Tian et al 2016			2016	2016	no	
included	included	*Bubo*	*flavipes*	Strigidae	LC099100		could not be verified: reference seq may be misid'd	could not be verified: no COI	verified	no	Spiridonova & Surmach 2018			2018	2018	Phylogram	(duplicate)
included	included	*Bubo*	*scandiacus*	Strigidae	MG681084	NC_038220	could not be verified: no ND2	verified	verified	no	Kang et al 2018			2018	2018	Phylogram	(duplicate)
included	excluded: no paper (yet)	*Bubulcus*	*ibis*	Ardeidae	KJ722534		could not be verified: no ND2	verified	verified	no	Zhang, B., Sun, Z., Chang, Q., Hu, C. and Pan, T.	2014	?	unpubl	2014	-	
included	included	*Bubulcus*	*ibis*	Ardeidae	KJ190945	NC_025917	could not be verified: no ND2	verified	verified	no	Zhou et al 2014b			2014	2014	Phylogram	(duplicate)
included	included	*Bucco*	*macrodactylus*	Bucconidae	MK060124		could not be verified: no ND2	could not be verified: no COI	could not be verified: no cyt b	?	Tamashiro et al 2019			2019	2019	Phylogram	(duplicate)
included	included	*Buceros*	*bicornis*	Bucerotidae	MF325011	NC_038201	verified	verified	verified	no	Chen et al 2018c			2018	2018	no	
included	included	*Buceros*	*rhinoceros*	Bucerotidae	MK060125		could not be verified: no ND2	could not be verified: no COI	verified	no	Tamashiro et al 2019			2019	2019	Phylogram	(duplicate)
included	included	*Buceros*	*rhinoceros silvestris*	Bucerotidae	MG596878		could not be verified: no ND2	could not be verified: no COI	verified	no	Lan et al 2019ip			2019	2019	no	
included	included	*Bucorvus*	*leadbeateri*	Bucerotidae	HM640209	NC_015199	could not be verified: no ND2	verified	verified	no	Pacheco et al 2011			2011	2011	Phylogram	(duplicate)
included	excluded: no paper (yet)	*Butastur*	*indicus*	Accipitridae	AB830616	NC_032362	verified	verified	verified	no	Nagai,K. and Azuma,A.	2013	?	unpubl	2013	-	
included	excluded: no paper (yet)	*Butastur*	*liventer*	Accipitridae	AB830617	NC_032363	could not be verified: no ND2	could not be verified: no COI	could not be verified: no cyt b	?	Nagai, K. and Azuma, A.	2013	?	unpubl	2013	-	
included	included	*Buteo*	*buteo*	Accipitridae	AF380305	NC_003128	could not be verified: no ND2	verified	verified	no	Haring et al 2001			2001	2001	Phylogram	-
included	included	*Buteo*	*buteo burmanicus*	Accipitridae	KM364882		could not be verified: no ND2	verified	verified	no	Peng et al 2016a			2016	2016	no	
included	included	*Buteo*	*hemilasius*	Accipitridae	KT935541	NC_029377	could not be verified: no ND2	verified	could not be verified: no cyt b	no	Sun et al 2017b			2017	2017	Cladogram	-
included	included	*Buteo*	*lagopus*	Accipitridae	KP337337	NC_029189	verified	verified	verified	no	Jiang et al 2015			2015	2015	Phylogram	(duplicate)
included	included	*Butorides*	*striatus*	Ardeidae	KJ190953	NC_025922	could not be verified: no ND2	verified	verified	no	Zhou et al 2014b			2014	2014	Phylogram	(duplicate)
included	included	*Bycanistes*	*brevis*	Bucerotidae	HM640210	NC_015201	could not be verified: no ND2	verified	verified	no	Pacheco et al 2011			2011	2011	Phylogram	(duplicate)

Sequence database	Phylogeny database	Genus	species	family	GenBank#	RefSeq#	ND2 verified	COI verified	cyt b verified	erroneous sequence?	reference	submitted to GenBank	sequence released on GenBank	publication of paper	YEAR	Phylogeny (mitogenomic)	problematic sequences in tree
included	included	*Cacatua*	*moluccensis*	Cacatuidae	MH133972		could not be verified: no ND2	could not be verified: no COI	verified	no	Urantowka et al 2018			2018	2018	Cladogram	*Brotogeris cyanopterus*
included	included	*Cacatua*	*moluccensis*	Cacatuidae	JF414239	NC_020592	dubious	could not be verified: no COI	verified	?	White et al 2011			2011	2011	no	
included	included	*Cacatua*	*pastinator*	Cacatuidae	JF414240		could not be verified: no structure	could not be verified: no structure	could not be verified: no cyt b	?	White et al 2011			2011	2011	no	
included	included	*Cacatua*	*pastinator pastinator*	Cacatuidae	MH133973	NC_040142	could not be verified: no structure	could not be verified: no structure	could not be verified: no cyt b	?	Urantowka et al 2018			2018	2018	Cladogram	(duplicate)
included	included	*Cacatua*	*sanguinea*	Cacatuidae	MN126573		misidentified	misidentified	misidentified	yes (misID)	Sarker et al 2019c		2019	2019	2019	Cladogram	*Cacatua sanguinea, Brotogeris cyanopterus*
included	included	*Cairina*	*moschata*	Anatidae	EU755254	NC_010965	verified	verified	could not be verified: no cyt b	no	Tu et al 2014			2014	2014	no	
included	included	*Calidris*	*ruficollis*	Scolopacidae	MG736926	NC_040990	verified	verified	verified	no	Chen et al 2019a			2019	2019	Phylogram	-
included	included	*Callaeas*	*cinereus*	Callaeatidae	KU158191	NC_031350	verified	could not be verified: no COI	verified	no	Anmarkrud & Lifjeld 2017			2017	2017	no	
included	included	*Callipepla*	*squamata*	Odonthophoridae	KT722338	NC_029340	verified	verified	verified	no	Halley et al 2015			2015	2015	Phylogram	-
included	excluded: no paper (yet)	*Calliphlox*	*amethystina*	Trochilidae	KP853095	NC_030286	verified	dubious	could not be verified: no cyt b	no	Prosdocimi, F., Ruschi, P. and Jennings, B.	2015	2016	unpubl	2016	-	
included	included	*Caloenas*	*nicobarica*	Columbidae	MG590264		verified	verified	verified	no	Bruxaux et al 2018			2018	2018	Phylogram	-
included	included	*Caloenas*	*maculata*	Columbidae	KX902249	NC_031870	could not be verified: no ND2	could not be verified: no COI	could not be verified: no cyt b	?	Soares et al 2016			2016	2016	Cladogram	(duplicate)
included	included	*Caloenas*	*nicobarica*	Columbidae	KX902248	NC_031869	verified	verified	verified	no	Soares et al 2016			2016	2016	Cladogram	(duplicate)
included	included	*Caloperdix*	*oculeus*	Phasianidae	KJ914546	NC_024619	verified	could not be verified: no COI	could not be verified: no cyt b	no	Meiklejohn et al 2014			2014	2014	Phylogram	*Phasianus versicolor, Francolinus pintadeanus*
included	excluded: no paper (yet)	*Calypte*	*anna*	Trochilidae	CM016612		verified	verified	verified	no	Jarvis, E.D., Rhei, A., Korlach, J., Fedrigo, O., et al	2018	2019	unpubl	2019	-	
included	included	*Calyptorhynchus*	*baudinii*	Cacatuidae	MH133969		could not be verified: no structure	could not be verified: no structure	could not be verified: no cyt b	?	Urantowka et al 2018			2018	2018	Cladogram	(duplicate)
included	included	*Calyptorhynchus*	*baudinii*	Cacatuidae	JF414242	NC_020594	could not be verified: no structure	could not be verified: no structure	could not be verified: no cyt b	?	White et al 2011			2011	2011	no	
included	included	*Calyptorhynchus*	*lathami*	Cacatuidae	JF414241	NC_020593	verified	verified	could not be verified: no cyt b	no	White et al 2011			2011	2011	no	
included	included	*Calyptorhynchus*	*latirostris*	Cacatuidae	JF414243	NC_020595	could not be verified: no structure	could not be verified: no structure	could not be verified: no cyt b	?	White et al 2011			2011	2011	no	
included	included	*Campephilus*	*guatemalensis*	Picidae	KT443920	NC_028020	verified	verified	verified	no	Fuchs et al 2015			2015	2015	Phylogram	*Trogon viridis*

Sequence database	Phylogeny database	Genus	species	family	GenBank#	RefSeq#	ND2 verified	COI verified	cyt b verified	erroneous sequence?	reference	submitted to GenBank	sequence released on GenBank	publication of paper	YEAR	Phylogeny (mitogenomic)	problematic sequences in tree
included	included	*Campephilus*	*imperialis*	Picidae	KU158198	NC_034278	could not be verified: no ND2	could not be verified: no COI	could not be verified: no cyt b	?	Anmarkrud & Lifjeld 2017			2017	2017	no	
included	included	*Camptorhynchus*	*labradorius*	Anatidae	n.a.		could not be verified: no ND2	could not be verified: no COI	could not be verified: no cyt b	?	Buckner et al 2018			2018	2018	Phylogram	*Anas clypeata, Anas falcata, Anas crecca, Branta bernicla, Netta rufina, Aix galericulata*
included	excluded: no paper (yet)	*Campylorhynchus*	*brunneicapillus*	Troglodytidae	KU057376	NC_029482	verified	verified	verified	no	Zhao, H	2015	2016	unpubl	2016	-	
included	included	*Campylorhynchus*	*zonatus*	Troglodytidae	KF509924	NC_022840	verified	could not be verified: no COI	verified	no	Barker et al 2015			2015	2015	no	
included	included	*Canirallus*	*oculeus*	Rallidae	MK434261		could not be verified: no ND2	could not be verified: no COI	could not be verified: no cyt b	?	Boast et al 2019			2019	2019	Phylogram	(duplicate)
included	included	*Capito*	*niger*	Capitonidae	MK060126		verified	verified	verified	no	Tamashiro et al 2019			2019	2019	Phylogram	(duplicate)
included	included	*Caprimulgus*	*indicus / jotaka*	Caprimulgidae	KM272749	NC_025773	misidentified	misidentified	misidentified	yes (chimera)	Zhao et al 2016a (Liu et al 2019)			2016	2016	no	
included	included	*Caracara*	*cheriway*	Falconidae	MN231451	NC_044673	could not be verified: no structure	verified	could not be verified: no structure	no	Oswald et al 2019		2019	2019	2019	Phylogram	(duplicate)
included	included	*Caracara*	*plancus*	Falconidae	MN231450	NC_044672	could not be verified: no structure	verified	could not be verified: no structure	no	Oswald et al 2019		2019	2019	2019	Phylogram	-
included	included	*Cardellina*	*canadensis*	Parulidae	MK033135	NC_041111	verified	verified	verified	no	Coughlin et al 2019			2019	2019	Phylogram	-
included	included	*Cardinalis*	*cardinalis*	Thraupidae	MH700631		verified	verified	verified	no	Campillo et al 2019	2018		2019	2019	Phylogram	-
included	excluded: no paper (yet)	*Cardinalis*	*cardinalis*	Cardinalidae	FJ236295		verified	verified	verified	no	Carson, R.J. and Spicer, G.S.	2008	?	unpubl	2008	-	
included	included	*Cardinalis*	*cardinalis*	Cardinalidae	KM078795	NC_025618	verified	verified	verified	no	Lerner et al 2011			2011	2011	Cladogram	(duplicate)
included	excluded: no paper (yet)	*Carduelis (Choris)*	*sinica*	Fringillidae	HQ915865	NC_015196	verified	verified	verified	no	Kan, X.-Z. and Zhang, L.-Q.	2011	?	unpubl	2011	-	
included	included	*Carduelis (Choris)*	*sinica*	Fringillidae	KM078783		verified	verified	verified	no	Lerner et al 2011			2011	2011	Cladogram	(duplicate)
included	included	*Carduelis (Choris)*	*sinica*	Fringillidae	MH047558	NC_041094	verified	verified	verified	no	Kim et al 2018a			2018	2018	Phylogram	-
included	included	*Carduelis (Choris)*	*sinica ussuriensis*	Fringillidae	MH047559		verified	verified	verified	no	Kim et al 2018a			2018	2018	Phylogram	(duplicate)
included	included	*Carduelis (Spinus)*	*pinus*	Fringillidae	KM078796		verified	verified	verified	no	Lerner et al 2011			2011	2011	Cladogram	(duplicate)
included	included	*Carduelis (Spinus)*	*psaltria*	Fringillidae	KM078806	NC_025627	verified	verified	verified	no	Lerner et al 2011			2011	2011	Cladogram	(duplicate)
included	included	*Carduelis (Spinus)*	*spinus*	Fringillidae	HQ915866	NC_015198	verified	verified	verified	no	Kan et al 2016			2016	2016	no	
included	excluded: no paper (yet)	*Carduelis (Spinus)*	*tristis*	Fringillidae	FJ236301		verified	verified	verified	no	Carson, R.J. and Spicer, G.S.	2008	?	unpubl	2008	-	

Sequence database	Phylogeny database	Genus	species	family	GenBank#	RefSeq#	ND2 verified	COI verified	cyt b verified	erroneous sequence?	reference	submitted to GenBank	sequence released on GenBank	publication of paper	YEAR	Phylogeny (mitogenomic)	problematic sequences in tree
included	excluded: no paper (yet)	*Cariama*	*cristata*	Cariamidae	CM020379		verified	verified	could not be verified: no cytb	no	Gilbert, M.T.P., Uliano Da Silva, M., Formenti, G., Chow, W., Collins, J., Howe, K., Bertelson, M., Rhie, A., Fedrigo, O., Jarvis, E.D.		2019	unpubl	2019	-	
included	included	*Carpodacus*	*erythrinus*	Fringillidae	KM078766	NC_025597	verified	verified	verified	no	Lerner et al 2011			2011	2011	Cladogram	(duplicate)
included	included	*Carpodacus*	*roseus*	Fringillidae	KM078779	NC_025607	verified	verified	verified	no	Lerner et al 2011			2011	2011	Cladogram	(duplicate)
included	included	*Carpodacus*	*rubicilloides*	Fringillidae	MH363715	NC_040975	verified	could not be verified: no COI	verified	no	Qin et al 2019			2019	2019	Cladogram	-
included	included	*Casuarius*	*bennetti*	Palaeognathae	AY016011		could not be verified: too few sequences of Palaeognathae	verified	verified	no	Cooper et al 2001			2001	2001	Phylogram	(duplicate)
included	included	*Casuarius*	*casuarius*	Palaeognathae	AF338713	NC_002778	could not be verified: too few sequences of Palaeognathae	could not be verified: no COI	verified	no	Haddrath & Baker 2001			2001	2001	Phylogram	(duplicate)
included	included	*Cathartes*	*aura*	Cathartidae	AY463690	NC_007628	verified	verified	verified	no	Slack et al 2007			2007	2007	network	(duplicate)
included	excluded: no paper (yet)	*Catharus*	*ustulatus*	Turdidae	CM020378		verified	verified	verified	no	Delmore, K., Vafadar, M., Formenti, G., Chow, W., Pelan, S., Howe, K., Rhie, A., Mountcastle, J., Fedrigo, O. and Jarvis, E.D.		2019	unpubl	2019	-	
included	included	*Cecropis*	*daurica*	Hirundinidae	KJ499911	NC_024107	verified	verified	verified	no	Liu et al 2016e			2016	2016	no	
included	included	*Centrocercus*	*minimus*	Phasianidae	CM016737		problematic	could not be verified: no resolution	problematic	yes (seq errors/numts)	Oh et al 2019		2019	2019	2019	no	-
included	included	*Centropus*	*sinensis*	Cuculidae	KT947122		verified	dubious	verified	no	Qu et al 2017			2017	2017	no	
included	included	*Ceryle*	*rudis*	Alcedinidae	KJ461938	NC_024280	misidentified	misidentified	could not be verified: reference seq may be misid'd	yes (chimera)	Sun et al 2017c			2017	2017	Phylogram	*Otus bakkamoena, Otus scops, Ceryle rudis, Trogon viridis*
included	excluded: paper publ. after 1-1-2020	*Ceryle (Megaceryle)*	*lugubris*	Alcedinidae	KY940558	NC_035658	misidentified	problematic	could not be verified: no cyt b	yes (misID)	Jing et al 2020	2017	2017	2020	2017	Phylogram	(duplicate)
included	included	*Chaetura*	*pelagica*	Apodidae	KT809406	NC_028545	verified	verified	verified	no	Xu & Zhang 2017			2017	2017	Cladogram	-
included	included	*Chalcophaps*	*indica*	Columbidae	HM746789		verified	verified	verified	no	Pacheco et al 2011			2011	2011	Phylogram	(duplicate)
included	included	*Charadrius*	*alexandrinus*	Charadriidae	MF565382	NC_041118	could not be verified: no ND2	verified	verified	no	Chen et al 2018b			2018	2018	Phylogram	*Charadrius placidus, Vanellus cinereus*

Sequence database	Phylogeny database	Genus	species	family	GenBank#	RefSeq#	ND2 verified	COI verified	cyt b verified	erroneous sequence?	reference	submitted to GenBank	sequence released on GenBank	publication of paper	YEAR	Phylogeny (mitogenomic)	problematic sequences in tree
included	included	*Charadrius*	*placidus*	Charadriidae	KY419888		dubious	misidentified	could not be verified: no cyt b	yes (misID)	Lee et al 2017d			2017	2017	Phylogram	*Charadrius placidus, Vanellus cinereus*
included	included	*Chendytes*	*lawi*	Anatidae	n.a.		could not be verified: no ND2	could not be verified: no COI	could not be verified: no cyt b	?	Buckner et al 2018			2018	2018	Phylogram	(duplicate)
included	included	*Chlamydotis*	*macqueenii*	Otididae	MK714019		could not be verified: no ND2	verified	verified	no	Jiang 2019a		2019	2019	2019	network	*Larus vegae*
included	included	*Chloroceryle*	*aenea*	Alcedinidae	MK060127		verified	verified	verified	no	Tamashiro et al 2019			2019	2019	Phylogram	(duplicate)
included	included	*Chlorophanes*	*spiza*	Fringillidae	KM078778	NC_025606	verified	verified	verified	no	Lerner et al 2011			2011	2011	Cladogram	(duplicate)
included	included	*Chroicocephalus*	*brunnicephalus*	Laridae	JX155863	NC_018548	could not be verified: no ND2	could not be verified: no COI	could not be verified: no structure	?	Yang et al 2012			2012	2012	no	
included	included	*Chroicocephalus*	*ridibundus*	Laridae	KM577662	NC_025649	verified	verified	could not be verified: no structure	no	Dong et al 2016a			2016	2016	no	
included	included	*Chrysolampis*	*mosquitus*	Trochilidae	KJ619585	NC_025786	verified	verified	could not be verified: no cyt b	no	Souto et al 2016			2016	2016	no	
included	included	*Chrysolophus*	*amherstiae*	Phasianidae	FJ752434	NC_020590	verified	verified	verified	no	Shen et al 2010			2010	2010	Phylogram	(duplicate)
included	included	*Chrysolophus*	*pictus*	Phasianidae	HQ221859	NC_014576	verified	verified	verified	no	Li et al 2011			2011	2011	Cladogram	*Phasianus versicolor*
included	included	*Chrysolophus*	*pictus*	Phasianidae	MG735217		verified	verified	verified	no	Ren et al 2019a			2019	2019	Phylogram	*Phasianus versicolor*
included	included	*Chrysolophus*	*pictus*	Phasianidae	FJ752433		verified	verified	verified	no	Shen et al 2010			2010	2010	Phylogram	(duplicate)
included	included	*Chrysomus*	*icterocephalus*	Icteridae	JX516060	NC_018799	verified	verified	verified	no	Powell et al 2013			2013	2013	Cladogram	(duplicate)
included	included	*Chrysomus*	*ruficapillus*	Icteridae	JX516056	NC_018796	verified	verified	verified	no	Powell et al 2013			2013	2013	Cladogram	(duplicate)
included	included	*Ciconia*	*boyciana*	Ciconiidae	AB026193	NC_002196	could not be verified: no ND2	verified	verified	no	Yamamoto et al 2000			2000	2000	no	
included	excluded: no paper (yet)	*Ciconia*	*ciconia*	Ciconiidae	AB026818	NC_002197	verified	verified	verified	no	Yamamoto, Y.	1999	2000	unpubl	2000	-	
included	included	*Ciconia*	*nigra*	Ciconiidae	KY767670		could not be verified: no ND2	could not be verified: no COI	verified	no	Lee et al 2017b			2017	2017	Phylogram	*Charadrius placidus*
included	included	*Ciconia*	*nigra*	Ciconiidae	MK818509		could not be verified: no ND2	could not be verified: no COI	verified	no	Liang et al 2019		2019	2019	2019	Phylogram	-
included	included	*Ciconia*	*nigra*	Ciconiidae	KF906246	NC_023946	could not be verified: no ND2	could not be verified: no COI	verified	no	Liu et al 2016f			2016	2016	Phylogram	-
included	included	*Circus*	*assimilis*	Accipitridae	MK294163		could not be verified: no ND2	could not be verified: no COI	could not be verified: no cyt b	?	Knapp et al 2019			2019	2019	Cladogram	*Aquila heliaca*
included	excluded: paper publ. after 1-1-2020	*Circus*	*cyaneus*	Accipitridae	KU237286		could not be verified: no ND2	verified	verified	no	Choi et al 2021	2015	2018	2021	2018	Phylogram	*Aquila heliaca*

Sequence database	Phylogeny database	Genus	species	family	GenBank#	RefSeq#	ND2 verified	COI verified	cyt b verified	erroneous sequence?	reference	submitted to GenBank	sequence released on GenBank	publication of paper	YEAR	Phylogeny (mitogenomic)	problematic sequences in tree
included	included	*Circus*	*cyaneus*	Accipitridae	KX925606		could not be verified: no ND2	verified	verified	no	Gao et al 2018			2018	2018	Cladogram	-
included	excluded: no paper (yet)	*Circus*	*melanoleucos*	Accipitridae	KT438620	NC_035801	could not be verified: no ND2	could not be verified: no COI	could not be verified: no cyt b	?	Zhou, L. and Dong, Y.	2015	2017	unpubl	2017	-	
included	included	*Circus*	*teauteensis*	Accipitridae	MK386464		could not be verified: no ND2	could not be verified: no COI	could not be verified: no cyt b	?	Knapp et al 2019			2019	2019	Cladogram	(duplicate)
included	included	*Climacteris*	*picumnus*	Climacteridae	KY994579		verified	could not be verified: no COI	could not be verified: no cyt b	no	Lamb et al 2018			2018	2018	Phylogram	(duplicate)
included	included	*Climacteris*	*picumnus*	Climacteridae	KY994598		verified	could not be verified: no COI	could not be verified: no cyt b	no	Lamb et al 2018			2018	2018	Phylogram	(duplicate)
included	included	*Cnemotriccus*	*fuscatus*	Tyrannidae	AY596278	NC_007975	verified	verified	verified	no	Slack et al 2007			2007	2007	network	(duplicate)
included	included	*Coccothraustes*	*coccothraustes*	Fringillidae	KM078789	NC_025614	verified	verified	verified	no	Lerner et al 2011			2011	2011	Cladogram	(duplicate)
included	included	*Colinus*	*virginianus*	Odonthophoridae	KJ914548	NC_024620	verified	verified	verified	no	Meiklejohn et al 2014			2014	2014	Phylogram	(duplicate)
included	excluded: no paper (yet)	*Colinus*	*virginianus*	Odonthophoridae	CM018327		verified	verified	verified	no	Salter, J.F., Johnson, O., Stafford, N.J., Herrin, W.F., Schilling, D., Cedotal, C., Brumfield, R.T. and Faircloth, B.C.	2019	2019	unpubl	2019	-	
included	included	*Colluricincla*	*harmonica*	Pachycephalidae	KY994582		verified	could not be verified: no COI	verified	no	Lamb et al 2018			2018	2018	Phylogram	(duplicate)
included	included	*Colluricincla*	*harmonica*	Pachycephalidae	KY994593		verified	could not be verified: no COI	verified	no	Lamb et al 2018			2018	2018	Phylogram	(duplicate)
included	included	*Colluricincla*	*harmonica*	Pachycephalidae	KY994599		verified	could not be verified: no COI	verified	no	Lamb et al 2018			2018	2018	Phylogram	(duplicate)
included	included	*Colluricincla*	*harmonica*	Pachycephalidae	KY994601		verified	could not be verified: no COI	verified	no	Lamb et al 2018			2018	2018	Phylogram	(duplicate)
included	included	*Colluricincla*	*harmonica*	Pachycephalidae	KY994605		verified	could not be verified: no COI	verified	no	Lamb et al 2018			2018	2018	Phylogram	(duplicate)
included	included	*Columba*	*janthina*	Columbidae	KM926619		could not be verified: no ND2	could not be verified: no COI	could not be verified: no cyt b	?	Jang et al 2016			2016	2016	no	
included	included	*Columba*	*jouyi*	Columbidae	KX902247	NC_031868	could not be verified: no ND2	verified	could not be verified: no cyt b	no	Soares et al 2016			2016	2016	Cladogram	(duplicate)
included	excluded: no paper (yet)	*Columba*	*livia*	Columbidae	GQ240309		verified	verified	verified	no	Tsai, L.C., Lee, J.C., Liao, S.P., Linacre, A. and Hsieh, H.M.	2009	?	unpubl	2009	-	
included	included	*Columba*	*livia (Archangel)*	Columbidae	KJ722068		verified	verified	verified	no	Wu et al 2016b			2016	2016	no	
included	included	*Columba*	*livia (domestic)*	Columbidae	GU908131	NC_013978	verified	verified	verified	no	Kan et al 2010b			2010	2010	no	

Sequence database	Phylogeny database	Genus	species	family	GenBank#	RefSeq#	ND2 verified	COI verified	cyt b verified	erroneous sequence?	reference	submitted to GenBank	sequence released on GenBank	publication of paper	YEAR	Phylogeny (mitogenomic)	problematic sequences in tree
included	included	*Columba*	*livia (Egyptian swift)*	Columbidae	KF926376		verified	verified	verified	no	Li et al 2015c			2015	2015	no	
included	included	*Columba*	*livia (fancy pigeons)*	Columbidae	KP168712		verified	verified	verified	no	Zhang et al 2015b			2015	2015	no	
included	included	*Columba*	*livia (feral)*	Columbidae	KF907308		verified	verified	verified	no	Li et al 2014c			2014	2014	no	
included	included	*Columba*	*livia (ice pigeon)*	Columbidae	KP306517		verified	verified	verified	no	Zhang et al 2015c			2015	2015	no	
included	included	*Columba*	*livia (Jacobin pigeon)*	Columbidae	KP319029		verified	verified	verified	no	He et al 2015			2015	2015	no	
included	included	*Columba*	*livia (king pigeon)*	Columbidae	KP258178		verified	verified	verified	no	Zhang et al 2015a			2015	2015	no	
included	included	*Columba*	*rupestris*	Columbidae	KX902246	NC_031867	verified	verified	verified	no	Soares et al 2016			2016	2016	Cladogram	(duplicate)
included	included	*Copsychus*	*saularis*	Muscicapidae	KU058637	NC_030603	verified	verified	verified	no	Peng et al 2016b			2016	2016	Phylogram	*Cyanoptila cyanomelana, Pseudopodoces humilis, Turdus merula 1*
included	included	*Coracopsis*	*vasa*	Psittaculidae	KM611468	NC_027841	verified	verified	verified	no	Eberhard & Wright 2016			2016	2016	Cladogram	(duplicate)
included	included	*Cormobates*	*leucophaeus*	Climacteridae	KY994580		verified	could not be verified: no COI	could not be verified: no cyt b	no	Lamb et al 2018			2018	2018	Phylogram	(duplicate)
included	included	*Cormobates*	*leucophaeus*	Climacteridae	KY994590		verified	could not be verified: no COI	could not be verified: no cyt b	no	Lamb et al 2018			2018	2018	Phylogram	(duplicate)
included	included	*Cormobates*	*leucophaeus*	Climacteridae	KY994608		verified	could not be verified: no COI	could not be verified: no cyt b	no	Lamb et al 2018			2018	2018	Phylogram	(duplicate)
included	included	*Corvus*	*brachyrhynchos*	Corvidae	KP403809	NC_026461	verified	could not be verified: no structure	verified	no	Li et al 2016a			2016	2016	Phylogram	-
included	included	*Corvus*	*corax*	Corvidae	KX245133	NC_034838	verified	verified	verified	no	Johnsen et al 2017			2017	2017	Phylogram	-
included	included	*Corvus*	*corax*	Corvidae	KX245134		verified	verified	verified	no	Johnsen et al 2017			2017	2017	Phylogram	(duplicate)
included	included	*Corvus*	*corax*	Corvidae	KX245135		verified	verified	verified	no	Johnsen et al 2017			2017	2017	Phylogram	(duplicate)
included	included	*Corvus*	*corax*	Corvidae	KX245136		verified	verified	verified	no	Johnsen et al 2017			2017	2017	Phylogram	(duplicate)
included	included	*Corvus*	*corax*	Corvidae	KX245137		verified	verified	verified	no	Johnsen et al 2017			2017	2017	Phylogram	(duplicate)
included	included	*Corvus*	*corax*	Corvidae	KX245138		verified	verified	verified	no	Johnsen et al 2017			2017	2017	Phylogram	(duplicate)
included	included	*Corvus*	*corax*	Corvidae	KX245140		verified	verified	verified	no	Johnsen et al 2017			2017	2017	Phylogram	(duplicate)
included	included	*Corvus*	*corax*	Corvidae	KX245141		verified	verified	verified	no	Johnsen et al 2017			2017	2017	Phylogram	(duplicate)
included	included	*Corvus*	*corax*	Corvidae	KX245142		verified	verified	verified	no	Johnsen et al 2017			2017	2017	Phylogram	(duplicate)
included	included	*Corvus*	*corax*	Corvidae	KX245145		verified	verified	verified	no	Johnsen et al 2017			2017	2017	Phylogram	(duplicate)
included	included	*Corvus*	*corax*	Corvidae	KX245146		verified	verified	verified	no	Johnsen et al 2017			2017	2017	Phylogram	(duplicate)
included	included	*Corvus*	*corax*	Corvidae	KX245148		verified	verified	verified	no	Johnsen et al 2017			2017	2017	Phylogram	(duplicate)
included	included	*Corvus*	*cornix*	Corvidae	CM002877	NC_024698	could not be verified: no structure	could not be verified: no structure	could not be verified: no structure	?	Poelstra et al 2014			2014	2014	no	
included	included	*Corvus*	*corone orientalis*	Corvidae	MK714020		could not be verified: no ND2	verified	verified	no	Jiang 2019b		2019	2019	2019	Phylogram	-
included	included	*Corvus*	*coronoides*	Corvidae	MF370524	NC_035877	verified	problematic	problematic	yes (chimera)	Sarker et al 2017a			2017	2017	Cladogram	*Corvus coronoides*
included	included	*Corvus*	*cryptoleucus*	Corvidae	KX245139	NC_034839	verified	verified	verified	no	Johnsen et al 2017			2017	2017	Phylogram	(duplicate)
included	included	*Corvus*	*cryptoleucus*	Corvidae	KX245143		verified	verified	verified	no	Johnsen et al 2017			2017	2017	Phylogram	(duplicate)
included	included	*Corvus*	*cryptoleucus*	Corvidae	KX245147		verified	verified	verified	no	Johnsen et al 2017			2017	2017	Phylogram	(duplicate)

Sequence database	Phylogeny database	Genus	species	family	GenBank#	RefSeq#	ND2 verified	COI verified	cyt b verified	erroneous sequence?	reference	submitted to GenBank	sequence released on GenBank	publication of paper	YEAR	Phylogeny (mitogenomic)	problematic sequences in tree
included	included	*Corvus*	*frugilegus*	Corvidae	Y18522	NC_002069	verified	verified	verified	no	Härlid & Arnason 1999 (Nilsson et al. 2004)			1999	1999	Phylogram	-
included	included	*Corvus*	*hawaiiensis*	Corvidae	KP161619		verified	could not be verified: no COI	verified	no	Hoeck et al 2015			2015	2015	no	
included	included	*Corvus*	*hawaiiensis*	Corvidae	KP161620	NC_026783	verified	could not be verified: no COI	verified	no	Hoeck et al 2015			2015	2015	no	
included	included	*Corvus*	*macrorhynchos*	Corvidae	KR057957	NC_027173	verified	verified	verified	no	Krzeminska et al 2016a			2016	2016	Phylogram	*Lanius tephronotus*
included	included	*Corvus*	*macrorhynchos*	Corvidae	KR072661		verified	verified	verified	no	Krzeminska et al 2016a			2016	2016	Phylogram	(duplicate)
included	excluded: paper publ. after 1-1-2020	*Corvus*	*macrorhynchos intermedius*	Corvidae	MN069302		verified	verified	verified	no	Iqbal et al 2020		2019	2020	2019	Cladogram	-
included	excluded: no paper (yet)	*Corvus*	*moneduloides*	Corvidae	CM018859		verified	could not be verified: no COI	could not be verified: no cyt b	no	Rutz, C., Fungtammasan, C., Mountcastle, J., Formenti, G. et al	2019	2019	unpubl	2019	-	
included	included	*Corvus*	*moriorum*	Corvidae	KX822153	NC_031518	could not be verified: no ND2	could not be verified: no COI	could not be verified: no cyt b	?	Scofield et al 2017			2017	2017	no	
included	included	*Corvus*	*moriorum*	Corvidae	KX822154		could not be verified: no ND2	could not be verified: no COI	could not be verified: no cyt b	?	Scofield et al 2017			2017	2017	no	
included	included	*Corvus*	*pectoralis*	Corvidae	MN310552	NC_045381	verified	dubious	dubious	no	Huang et al 2019c		2019	2019	2019	Phylogram	-
included	included	*Corvus*	*ruficollis*	Corvidae	KX245144		verified	could not be verified: no COI	verified	no	Johnsen et al 2017			2017	2017	Phylogram	(duplicate)
included	included	*Corvus*	*splendens*	Corvidae	KJ766304	NC_024607	verified	verified	verified	no	Krzeminska et al 2016b			2016	2016	no	
included	included	*Corvus*	*splendens*	Corvidae	KP019935		verified	verified	verified	no	Krzeminska et al 2018			2018	2018	Phylogram	-
included	included	*Corvus*	*splendens*	Corvidae	KP019936		verified	verified	verified	no	Krzeminska et al 2018			2018	2018	Phylogram	(duplicate)
included	included	*Corvus*	*splendens*	Corvidae	KP019937		verified	verified	verified	no	Krzeminska et al 2018			2018	2018	Phylogram	(duplicate)
included	included	*Corvus*	*splendens*	Corvidae	KP019938		verified	verified	verified	no	Krzeminska et al 2018			2018	2018	Phylogram	(duplicate)
included	included	*Corvus*	*splendens*	Corvidae	KP019939		verified	verified	verified	no	Krzeminska et al 2018			2018	2018	Phylogram	(duplicate)
included	included	*Corvus*	*splendens*	Corvidae	KP019940		verified	verified	verified	no	Krzeminska et al 2018			2018	2018	Phylogram	(duplicate)
included	included	*Corvus*	*splendens*	Corvidae	KY018606		verified	verified	verified	no	Krzeminska et al 2018			2018	2018	Phylogram	(duplicate)
included	included	*Corvus*	*splendens*	Corvidae	KY018607		verified	verified	verified	no	Krzeminska et al 2018			2018	2018	Phylogram	(duplicate)
included	included	*Corvus*	*splendens*	Corvidae	KY018608		verified	verified	verified	no	Krzeminska et al 2018			2018	2018	Phylogram	(duplicate)
included	included	*Corvus*	*splendens*	Corvidae	KY018609		verified	verified	verified	no	Krzeminska et al 2018			2018	2018	Phylogram	(duplicate)

Sequence database	Phylogeny database	Genus	species	family	GenBank#	RefSeq#	ND2 verified	COI verified	cyt b verified	erroneous sequence?	reference	submitted to GenBank	sequence released on GenBank	publication of paper	YEAR	Phylogeny (mitogenomic)	problematic sequences in tree
included	included	*Corvus*	*splendens*	Corvidae	KY018610		verified	verified	verified	no	Krzeminska et al 2018			2018	2018	Phylogram	(duplicate)
included	included	*Corvus*	*splendens*	Corvidae	KY018611		verified	verified	verified	no	Krzeminska et al 2018			2018	2018	Phylogram	(duplicate)
included	included	*Corvus*	*splendens*	Corvidae	KY018612		verified	verified	verified	no	Krzeminska et al 2018			2018	2018	Phylogram	(duplicate)
included	included	*Corvus*	*splendens*	Corvidae	KY018613		verified	verified	verified	no	Krzeminska et al 2018			2018	2018	Phylogram	(duplicate)
included	included	*Corvus*	*splendens*	Corvidae	KY018614		verified	verified	verified	no	Krzeminska et al 2018			2018	2018	Phylogram	(duplicate)
included	included	*Corvus*	*splendens*	Corvidae	KY018615		verified	verified	verified	no	Krzeminska et al 2018			2018	2018	Phylogram	(duplicate)
included	included	*Corvus*	*splendens*	Corvidae	KY018616		verified	verified	verified	no	Krzeminska et al 2018			2018	2018	Phylogram	(duplicate)
included	included	*Corvus*	*splendens*	Corvidae	KY018617		verified	verified	verified	no	Krzeminska et al 2018			2018	2018	Phylogram	(duplicate)
included	included	*Corvus*	*splendens*	Corvidae	KY018618		verified	verified	verified	no	Krzeminska et al 2018			2018	2018	Phylogram	(duplicate)
included	included	*Corvus*	*splendens*	Corvidae	KY018619		verified	verified	verified	no	Krzeminska et al 2018			2018	2018	Phylogram	(duplicate)
included	included	*Corvus*	*splendens*	Corvidae	KY018620		verified	verified	verified	no	Krzeminska et al 2018			2018	2018	Phylogram	(duplicate)
included	included	*Corvus*	*splendens*	Corvidae	KY018621		verified	verified	verified	no	Krzeminska et al 2018			2018	2018	Phylogram	(duplicate)
included	included	*Corvus*	*splendens*	Corvidae	KY018622		verified	verified	verified	no	Krzeminska et al 2018			2018	2018	Phylogram	(duplicate)
included	included	*Corvus*	*splendens*	Corvidae	KY018623		verified	verified	verified	no	Krzeminska et al 2018			2018	2018	Phylogram	(duplicate)
included	included	*Corvus*	*splendens*	Corvidae	KY018624		verified	verified	verified	no	Krzeminska et al 2018			2018	2018	Phylogram	(duplicate)
included	included	*Corvus*	*splendens*	Corvidae	KY018625		verified	verified	verified	no	Krzeminska et al 2018			2018	2018	Phylogram	(duplicate)
included	included	*Corvus*	*splendens*	Corvidae	KY018626		verified	verified	verified	no	Krzeminska et al 2018			2018	2018	Phylogram	(duplicate)
included	included	*Corvus*	*splendens*	Corvidae	KY018627		verified	verified	verified	no	Krzeminska et al 2018			2018	2018	Phylogram	(duplicate)
included	included	*Corvus*	*splendens*	Corvidae	KY018628		verified	verified	verified	no	Krzeminska et al 2018			2018	2018	Phylogram	(duplicate)
included	included	*Corvus*	*splendens*	Corvidae	KY018629		verified	verified	verified	no	Krzeminska et al 2018			2018	2018	Phylogram	(duplicate)
included	included	*Corvus*	*splendens*	Corvidae	KY018630		verified	verified	verified	no	Krzeminska et al 2018			2018	2018	Phylogram	(duplicate)
included	included	*Corvus*	*splendens*	Corvidae	KY018631		verified	verified	verified	no	Krzeminska et al 2018			2018	2018	Phylogram	(duplicate)
included	included	*Corvus*	*splendens*	Corvidae	KY018632		verified	verified	verified	no	Krzeminska et al 2018			2018	2018	Phylogram	(duplicate)
included	included	*Corvus*	*splendens*	Corvidae	KY018633		verified	verified	verified	no	Krzeminska et al 2018			2018	2018	Phylogram	(duplicate)
included	included	*Corvus*	*splendens*	Corvidae	KY018634		verified	verified	verified	no	Krzeminska et al 2018			2018	2018	Phylogram	(duplicate)
included	included	*Corvus*	*splendens*	Corvidae	KY018635		verified	verified	verified	no	Krzeminska et al 2018			2018	2018	Phylogram	(duplicate)
included	included	*Corvus*	*splendens*	Corvidae	KY018636		verified	verified	verified	no	Krzeminska et al 2018			2018	2018	Phylogram	(duplicate)

Sequence database	Phylogeny database	Genus	species	family	GenBank#	RefSeq#	ND2 verified	COI verified	cyt b verified	erroneous sequence?	reference	submitted to GenBank	sequence released on GenBank	publication of paper	YEAR	Phylogeny (mitogenomic)	problematic sequences in tree
included	included	*Corvus*	*splendens*	Corvidae	KY018637		verified	verified	verified	no	Krzeminska et al 2018			2018	2018	Phylogram	(duplicate)
included	included	*Corvus*	*splendens*	Corvidae	KY018638		verified	verified	verified	no	Krzeminska et al 2018			2018	2018	Phylogram	(duplicate)
included	included	*Corvus*	*splendens*	Corvidae	KY018639		verified	verified	verified	no	Krzeminska et al 2018			2018	2018	Phylogram	(duplicate)
included	included	*Corvus*	*splendens*	Corvidae	KY018640		verified	verified	verified	no	Krzeminska et al 2018			2018	2018	Phylogram	(duplicate)
included	included	*Corvus*	*splendens*	Corvidae	KY018641		verified	verified	verified	no	Krzeminska et al 2018			2018	2018	Phylogram	(duplicate)
included	included	*Corvus*	*splendens*	Corvidae	KY018642		verified	verified	verified	no	Krzeminska et al 2018			2018	2018	Phylogram	(duplicate)
included	included	*Corvus*	*splendens*	Corvidae	KY018643		verified	verified	verified	no	Krzeminska et al 2018			2018	2018	Phylogram	(duplicate)
included	included	*Corvus*	*splendens*	Corvidae	KY018644		verified	verified	verified	no	Krzeminska et al 2018			2018	2018	Phylogram	(duplicate)
included	included	*Corvus*	*splendens*	Corvidae	KY018645		verified	verified	verified	no	Krzeminska et al 2018			2018	2018	Phylogram	(duplicate)
included	included	*Corvus*	*splendens*	Corvidae	KY018646		verified	verified	verified	no	Krzeminska et al 2018			2018	2018	Phylogram	(duplicate)
included	included	*Corvus*	*splendens*	Corvidae	KY018647		verified	verified	verified	no	Krzeminska et al 2018			2018	2018	Phylogram	(duplicate)
included	included	*Corvus*	*splendens*	Corvidae	KY018648		verified	verified	verified	no	Krzeminska et al 2018			2018	2018	Phylogram	(duplicate)
included	included	*Corvus*	*splendens*	Corvidae	KY018649		verified	verified	verified	no	Krzeminska et al 2018			2018	2018	Phylogram	(duplicate)
included	included	*Corvus*	*splendens*	Corvidae	KY018650		verified	verified	verified	no	Krzeminska et al 2018			2018	2018	Phylogram	(duplicate)
included	included	*Corvus*	*splendens*	Corvidae	KY018651		verified	verified	verified	no	Krzeminska et al 2018			2018	2018	Phylogram	(duplicate)
included	included	*Corvus*	*splendens*	Corvidae	KY018652		verified	verified	verified	no	Krzeminska et al 2018			2018	2018	Phylogram	(duplicate)
included	included	*Corvus*	*splendens*	Corvidae	KY018653		verified	verified	verified	no	Krzeminska et al 2018			2018	2018	Phylogram	(duplicate)
included	included	*Corvus*	*splendens*	Corvidae	KY018654		verified	verified	verified	no	Krzeminska et al 2018			2018	2018	Phylogram	(duplicate)
included	included	*Corvus*	*splendens*	Corvidae	KY018655		verified	verified	verified	no	Krzeminska et al 2018			2018	2018	Phylogram	(duplicate)
included	included	*Corvus*	*splendens*	Corvidae	KY018656		verified	verified	verified	no	Krzeminska et al 2018			2018	2018	Phylogram	(duplicate)
included	included	*Corvus*	*splendens*	Corvidae	KY018657		verified	verified	verified	no	Krzeminska et al 2018			2018	2018	Phylogram	(duplicate)
included	included	*Corvus*	*splendens*	Corvidae	KY018658		verified	verified	verified	no	Krzeminska et al 2018			2018	2018	Phylogram	(duplicate)
included	included	*Corvus*	*splendens*	Corvidae	KY018659		verified	verified	verified	no	Krzeminska et al 2018			2018	2018	Phylogram	(duplicate)
included	included	*Corvus*	*splendens*	Corvidae	KY018660		verified	verified	verified	no	Krzeminska et al 2018			2018	2018	Phylogram	(duplicate)
included	included	*Corvus*	*splendens*	Corvidae	KY018661		verified	verified	verified	no	Krzeminska et al 2018			2018	2018	Phylogram	(duplicate)
included	included	*Corvus*	*splendens*	Corvidae	KY018662		verified	verified	verified	no	Krzeminska et al 2018			2018	2018	Phylogram	(duplicate)
included	included	*Corvus*	*splendens*	Corvidae	KY018663		verified	verified	verified	no	Krzeminska et al 2018			2018	2018	Phylogram	(duplicate)

Sequence database	Phylogeny database	Genus	species	family	GenBank#	RefSeq#	ND2 verified	COI verified	cyt b verified	erroneous sequence?	reference	submitted to GenBank	sequence released on GenBank	publication of paper	YEAR	Phylogeny (mitogenomic)	problematic sequences in tree
included	included	*Corvus*	*splendens*	Corvidae	KY018664		verified	verified	verified	no	Krzeminska et al 2018			2018	2018	Phylogram	(duplicate)
included	included	*Corvus*	*splendens*	Corvidae	KY018665		verified	verified	verified	no	Krzeminska et al 2018			2018	2018	Phylogram	(duplicate)
included	included	*Corvus*	*splendens*	Corvidae	KY018666		verified	verified	verified	no	Krzeminska et al 2018			2018	2018	Phylogram	(duplicate)
included	included	*Corvus*	*splendens*	Corvidae	KY018667		verified	verified	verified	no	Krzeminska et al 2018			2018	2018	Phylogram	(duplicate)
included	included	*Corvus*	*splendens*	Corvidae	KY018668		verified	verified	verified	no	Krzeminska et al 2018			2018	2018	Phylogram	(duplicate)
included	included	*Corvus*	*splendens*	Corvidae	KY018669		verified	verified	verified	no	Krzeminska et al 2018			2018	2018	Phylogram	(duplicate)
included	included	*Corvus*	*splendens*	Corvidae	KY018670		verified	verified	verified	no	Krzeminska et al 2018			2018	2018	Phylogram	(duplicate)
included	included	*Corvus*	*splendens*	Corvidae	KY018671		verified	verified	verified	no	Krzeminska et al 2018			2018	2018	Phylogram	(duplicate)
included	included	*Corvus*	*splendens*	Corvidae	KY018672		verified	verified	verified	no	Krzeminska et al 2018			2018	2018	Phylogram	(duplicate)
included	included	*Corvus*	*splendens*	Corvidae	KY018673		verified	verified	verified	no	Krzeminska et al 2018			2018	2018	Phylogram	(duplicate)
included	included	*Corvus*	*splendens*	Corvidae	KY018674		verified	verified	verified	no	Krzeminska et al 2018			2018	2018	Phylogram	(duplicate)
included	included	*Corvus*	*splendens*	Corvidae	KY018675		verified	verified	verified	no	Krzeminska et al 2018			2018	2018	Phylogram	(duplicate)
included	included	*Corvus*	*splendens*	Corvidae	KY018676		verified	verified	verified	no	Krzeminska et al 2018			2018	2018	Phylogram	(duplicate)
included	included	*Corvus*	*splendens*	Corvidae	KY018677		verified	verified	verified	no	Krzeminska et al 2018			2018	2018	Phylogram	(duplicate)
included	included	*Corvus*	*splendens*	Corvidae	KY018678		verified	verified	verified	no	Krzeminska et al 2018			2018	2018	Phylogram	(duplicate)
included	included	*Corvus*	*splendens*	Corvidae	KY018679		verified	verified	verified	no	Krzeminska et al 2018			2018	2018	Phylogram	(duplicate)
included	included	*Corvus*	*splendens*	Corvidae	KY018680		verified	verified	verified	no	Krzeminska et al 2018			2018	2018	Phylogram	(duplicate)
included	included	*Corvus*	*splendens*	Corvidae	KY018681		verified	verified	verified	no	Krzeminska et al 2018			2018	2018	Phylogram	(duplicate)
included	included	*Corvus*	*splendens*	Corvidae	KY018682		verified	verified	verified	no	Krzeminska et al 2018			2018	2018	Phylogram	(duplicate)
included	included	*Corvus*	*splendens*	Corvidae	KY018683		verified	verified	verified	no	Krzeminska et al 2018			2018	2018	Phylogram	(duplicate)
included	included	*Corvus*	*splendens*	Corvidae	KY018684		verified	verified	verified	no	Krzeminska et al 2018			2018	2018	Phylogram	(duplicate)
included	included	*Corvus*	*splendens*	Corvidae	KY018685		verified	verified	verified	no	Krzeminska et al 2018			2018	2018	Phylogram	(duplicate)
included	included	*Corvus*	*splendens*	Corvidae	KY018686		verified	verified	verified	no	Krzeminska et al 2018			2018	2018	Phylogram	(duplicate)
included	included	*Corvus*	*splendens*	Corvidae	KY018687		verified	verified	verified	no	Krzeminska et al 2018			2018	2018	Phylogram	(duplicate)
included	included	*Corvus*	*splendens*	Corvidae	KY050718		verified	verified	verified	no	Krzeminska et al 2018			2018	2018	Phylogram	(duplicate)
included	included	*Coturnicops*	*exquisitus*	Rallidae	AP010823	NC_012143	could not be verified: no ND2	could not be verified: no COI	problematic	yes (seq errors/numts)	Ozaki et al 2010			2010	2010	no	

Sequence database	Phylogeny database	Genus	species	family	GenBank#	RefSeq#	ND2 verified	COI verified	cyt b verified	erroneous sequence?	reference	submitted to GenBank	sequence released on GenBank	publication of paper	YEAR	Phylogeny (mitogenomic)	problematic sequences in tree
included	included	*Coturnix*	*chinensis*	Phasianidae	AB073301	NC_004575	verified	could not be verified: no COI	verified	no	Nishibori et al 2002			2002	2002	no	
included	included	*Coturnix*	*japonica*	Phasianidae	KX712089		could not be verified: no ND2	verified	verified	no	Liu & Zhang 2016			2016	2016	Cladogram	-
included	included	*Coturnix*	*japonica*	Phasianidae	AP003195	NC_003408	could not be verified: no ND2	verified	verified	no	Nishibori et al 2001			2001	2001	no	
included	included	*Crax*	*daubentoni*	Cracidae	KJ914544	NC_024617	verified	could not be verified: no COI	verified	no	Meiklejohn et al 2014			2014	2014	Phylogram	(duplicate)
included	included	*Crax*	*rubra*	Cracidae	KJ914545	NC_024618	verified	could not be verified: no COI	verified	no	Meiklejohn et al 2014			2014	2014	Phylogram	(duplicate)
included	included	*Crossoptilon*	*auritum*	Phasianidae	JF937589	NC_015897	could not be verified: no structure	verified	could not be verified: no structure	no	Ren et al 2016e			2016	2016	Phylogram	*Phasianus versicolor*
included	included	*Crossoptilon*	*crossoptilon*	Phasianidae	KP259808		could not be verified: no structure	verified	could not be verified: no structure	no	Li et al 2015a			2015	2015	Phylogram	-
included	included	*Crossoptilon*	*crossoptilon*	Phasianidae	JQ713767		could not be verified: no structure	could not be verified: no structure	could not be verified: no structure	?	Shen et al 2014			2014	2014	Phylogram	(duplicate)
included	included	*Crossoptilon*	*crossoptilon*	Phasianidae	HQ891119	NC_016679	could not be verified: no structure	could not be verified: no structure	could not be verified: no structure	?	Zhao et al 2012			2012	2012	Phylogram	*Phasianus versicolor, Francolinus pintadeanus*
included	included	*Crossoptilon*	*harmani*	Phasianidae	KP259806	NC_026547	could not be verified: no structure	could not be verified: no COI	could not be verified: no structure	?	Li et al 2015a			2015	2015	Phylogram	(duplicate)
included	included	*Crossoptilon*	*mantchuricum*	Phasianidae	KP259807	NC_026548	could not be verified: no structure	could not be verified: no COI	could not be verified: no structure	?	Li et al 2015a			2015	2015	Phylogram	(duplicate)
included	excluded: no paper (yet)	*Crossoptilon*	*mantchuricum*	Phasianidae	KY070317		could not be verified: no structure	could not be verified: no COI	could not be verified: no structure	?	Ren, Z. and Takahiro, Y.	2016	2017	unpubl	2017	-	
included	included	*Crotophaga*	*ani*	Cuculidae	HM746794		verified	verified	verified	no	Pacheco et al 2011			2011	2011	Phylogram	(duplicate)
included	included	*Crypturellus*	*tataupa*	Palaeognathae	AY016012		could not be verified: too few sequences of Palaeognathae	verified	could not be verified: no cyt b	no	Cooper et al 2001			2001	2001	Phylogram	(duplicate)
included	included	*Cuculus*	*canorus bakeri*	Cuculidae	MN067867		dubious	dubious	could not be verified: no structure	yes (seq errors/numts)	Qiu et al 2019		2019	2019	2019	Phylogram	*Caprimulgus indicus, Cuculus canorus*
included	included	*Cuculus*	*poliocephalus*	Cuculidae	KT378620	NC_028414	verified	verified	verified	no	Wang et al 2016a			2016	2016	Phylogram	-
included	included	*Culicicapa*	*ceylonensis*	Stenostiridae	MH880820	NC_042191	verified	verified	verified	no	Tu et al 2019			2019	2019	Phylogram	-
included	included	*Curaeus*	*curaeus*	Icteridae	JX516070	NC_018808	verified	verified	verified	no	Powell et al 2013			2013	2013	Cladogram	(duplicate)
included	included	*Cyanistes*	*cyanus*	Paridae	KX388472		verified	verified	verified	no	Li et al 2016b (Li et al 2017)			2016	2016	Phylogram	*Periparus ater, Poecile palustris*

Sequence database	Phylogeny database	Genus	species	family	GenBank#	RefSeq#	ND2 verified	COI verified	cyt b verified	erroneous sequence?	reference	submitted to GenBank	sequence released on GenBank	publication of paper	YEAR	Phylogeny (mitogenomic)	problematic sequences in tree
included	excluded: no paper (yet)	*Cyanopica*	*cyanus*	Corvidae	JN108020	NC_015824	verified	verified	verified	no	Kan, X.-Z. and Chen, L.	2011	?	unpubl	2011	-	
included	excluded: no paper (yet)	*Cyanopica*	*cyanus koreensis*	Corvidae	KT934323		verified	verified	verified	no	Park, C.E., Hong, S.J., Park, G.S., Kim, M.C., Park, H.C. and Shin, J.H.	2015	2016	unpubl	2016	-	
included	excluded: no paper (yet)	*Cyanoptila*	*cyanomelana*	Muscicapidae	HQ896033	NC_015232	misidentified	misidentified	misidentified	yes (misID)	Kan, X.-Z. and Li, X.-F.	2011	?	unpubl	2011	-	
included	included	*Cygnus*	*atratus*	Anatidae	FJ379295	NC_012843	verified	verified	verified	no	Jiang et al 2010			2010	2010	Phylogram	*Phasianus versicolor*
included	included	*Cygnus*	*columbianus bewickii*	Anatidae	JQ282800	NC_017604	verified	verified	verified	no	Lee et al 2012			2012	2012	no	
included	included	*Cygnus*	*columbianus columbianus*	Anatidae	DQ083161	NC_007691	verified	verified	verified	no	Feinstein 2006			2006	2006	Cladogram	-
included	included	*Cygnus*	*columbianus jankowskii*	Anatidae	KF800698		verified	verified	verified	no	Wang et al 2016b			2016	2016	no	
included	included	*Cygnus*	*cygnus*	Anatidae	KP981363	NC_027095	verified	verified	verified	no	Park et al 2016a			2016	2016	Phylogram	*Branta bernicla, Anas falcata, Anser fabalis, Tadorna tadorna, Netta rufina, Anas crecca*
included	excluded: no paper (yet)	*Cygnus*	*cygnus*	Anatidae	KR821135		verified	verified	verified	no	Wang, Y.	2015	2015	unpubl	2015	-	
included	excluded: no paper (yet)	*Cygnus*	*olor*	Anatidae	CM020139		verified	verified	verified	no	Kraus, R., Fedrigo, O., Formenti, G., Mountcastle, J., Chow, W., Collins, J., Howe, K., Rhie, A., Karawita, A., Short, K. and Jarvis, E.D.		2019	unpubl	2019	-	
included	included	*Cygnus*	*olor*	Anatidae	KP981364	NC_027096	verified	verified	verified	no	Park et al 2016b			2016	2016	Phylogram	*Branta bernicla, Anas falcata, Anser fabalis, Tadorna tadorna, Netta rufina, Anas crecca*
included	excluded: no paper (yet)	*Cygnus*	*olor*	Anatidae	KR867679		verified	verified	verified	no	Wang, Y., Bai, S., Zhou, Q., Pang, X. and Yang, Y.	2015	2015	unpubl	2015	-	
included	included	*Cypseloides*	*fumigatus*	Apodidae	KY688216	NC_034933	could not be verified: no ND2	could not be verified: no COI	could not be verified: no cyt b	?	Biancalana et al 2017			2017	2017	no	
included	included	*Cyrtonyx*	*montezumae*	Odonthophoridae	n.a.		verified	verified	verified	no	Mathur et al 2019		2019	2019	2019	no	
included	included	*Dacelo*	*novaeguineae*	Alcedinidae	MK060128		verified	verified	verified	no	Tamashiro et al 2019			2019	2019	Phylogram	(duplicate)

Sequence database	Phylogeny database	Genus	species	family	GenBank#	RefSeq#	ND2 verified	COI verified	cyt b verified	erroneous sequence?	reference	submitted to GenBank	sequence released on GenBank	publication of paper	YEAR	Phylogeny (mitogenomic)	problematic sequences in tree
included	included	*Daphoenositta*	*chrysoptera*	Pachycephalidae	KY994578		verified	could not be verified: no COI	verified	no	Lamb et al 2018			2018	2018	Phylogram	(duplicate)
included	included	*Daphoenositta*	*chrysoptera*	Pachycephalidae	KY994586		verified	could not be verified: no COI	verified	no	Lamb et al 2018			2018	2018	Phylogram	(duplicate)
included	included	*Daphoenositta*	*chrysoptera*	Pachycephalidae	KY994604		verified	could not be verified: no COI	verified	no	Lamb et al 2018			2018	2018	Phylogram	(duplicate)
included	included	*Daphoenositta*	*chrysoptera*	Pachycephalidae	KY994611		verified	could not be verified: no COI	verified	no	Lamb et al 2018			2018	2018	Phylogram	(duplicate)
included	included	*Daptrion*	*capense*	Procellariidae	MH924023	NC_043899	could not be verified: no ND2	verified	verified	no	Jung et al 2019		2019	2019	2019	Cladogram	-
included	included	*Dendrocitta*	*formosae*	Corvidae	MK875763	NC_045376	verified	could not be verified: no COI	verified	no	Liu et al 2019h		2019	2019	2019	Phylogram	-
included	included	*Dendrocopos*	*darjellensis*	Picidae	MK335535	NC_042683	verified	could not be verified: no COI	dubious	dubious	Bi et al 2019		2019	2019	2019	Phylogram	*Dendrocopos canicapillus*
included	included	*Dendrocopos*	*leucotos*	Picidae	KU131555	NC_029862	verified	verified	verified	no	Eo 2017			2017	2017	Phylogram	*Ceryle rudis, Strix leptogrammica, Trogon viridis*
included	included	*Dendrocopos*	*major*	Picidae	KT350609	NC_028174	dubious	verified	verified	no	Park et al 2019c			2019	2019	Phylogram	-
included	included	*Dendrocopos (Yungipicus)*	*canicapillus*	Picidae	MK335534		verified	verified	dubious	yes (chimera)	Bi et al 2019		2019	2019	2019	Phylogram	(duplicate)
included	included	*Dendrocopos (Yungipicus)*	*canicapillus*	Picidae	MK015644	NC_041121	verified	verified	verified	no	Lai et al 2019			2019	2019	Cladogram	-
included	included	*Dendrocygna*	*javanica*	Anatidae	FJ379296	NC_012844	verified	verified	verified	no	Jiang et al 2010			2010	2010	Phylogram	(duplicate)
included	excluded: no paper (yet)	*Dendroica*	*coronata*	Parulidae	FJ236285		verified	verified	verified	no	Carson, R.J. and Spicer, G.S.	2008	?	unpubl	2008	-	
included	included	*Deroptyus*	*accipitrinus*	Psittacidae	KM611476		verified	verified	verified	no	Eberhard & Wright 2016			2016	2016	Cladogram	(duplicate)
included	included	*Dicrurus*	*hottentottus*	Dicruridae	MK814795	NC_043948	verified	verified	verified	no	Sun et al 2019b		2019	2019	2019	Cladogram	*Cyanoptila cyanomelanus, Lanius tephronotus*
included	included	*Didunculus*	*strigirostris*	Columbidae	MG590266		verified	could not be verified: no COI	verified	no	Bruxaux et al 2018			2018	2018	Phylogram	(duplicate)
included	included	*Didunculus*	*strigirostris*	Columbidae	KX902245	NC_031866	could not be verified: no ND2	could not be verified: no COI	verified	no	Soares et al 2016			2016	2016	Cladogram	(duplicate)
included	included	*Dives*	*dives*	Icteridae	JX516061	NC_018800	verified	verified	verified	no	Powell et al 2013			2013	2013	Cladogram	(duplicate)
included	included	*Dromaius*	*baudinianus*	Palaeognathae	MK625178	NC_045365	could not be verified: no ND2	could not be verified: no COI	could not be verified: no cyt b	?	Cibois et al 2019		2019	2019	2019	no	

Sequence database	Phylogeny database	Genus	species	family	GenBank#	RefSeq#	ND2 verified	COI verified	cyt b verified	erroneous sequence?	reference	submitted to GenBank	sequence released on GenBank	publication of paper	YEAR	Phylogeny (mitogenomic)	problematic sequences in tree
included	included	*Dromaius*	*novaehollandiae*	Palaeognathae	AY016014		could not be verified: too few sequences of Palaeognathae	verified	verified	no	Cooper et al 2001			2001	2001	Phylogram	(duplicate)
included	included	*Dromaius*	*novaehollandiae*	Palaeognathae	AF338711	NC_002784	could not be verified: too few sequences of Palaeognathae	verified	verified	no	Haddrath & Baker 2001			2001	2001	Phylogram	(duplicate)
included	included	*Dryocopus*	*pileatus*	Picidae	DQ780879	NC_008546	verified	verified	verified	no	Gibb et al 2007			2007	2007	Phylogram	(duplicate)
included	included	*Dryocopus*	*pileatus*	Picidae	MK060129		verified	verified	verified	no	Tamashiro et al 2019			2019	2019	Phylogram	(duplicate)
included	included	*Eclectus*	*roratus*	Psittaculidae	KM611469	NC_027842	verified	verified	verified	no	Eberhard & Wright 2016			2016	2016	Cladogram	(duplicate)
included	included	*Ectopistes*	*migratorius*	Columbidae	KU158192		could not be verified: no ND2	verified	verified	no	Anmarkrud & Lifjeld 2017			2017	2017	no	
included	included	*Ectopistes*	*migratorius*	Columbidae	KC489473	NC_042502	could not be verified: no ND2	verified	verified	no	Hung et al 2013			2013	2013	no	
included	included	*Ectopistes*	*migratorius*	Columbidae	KC489474		could not be verified: no ND2	verified	verified	no	Hung et al 2013			2013	2013	no	
included	included	*Ectopistes*	*migratorius*	Columbidae	KY260683		could not be verified: no ND2	verified	verified	no	Murray et al 2017			2017	2017	Cladogram	-
included	included	*Ectopistes*	*migratorius*	Columbidae	KY974335		could not be verified: no ND2	verified	verified	no	Murray et al 2017			2017	2017	Cladogram	(duplicate)
included	included	*Ectopistes*	*migratorius*	Columbidae	KY974336		could not be verified: no ND2	verified	verified	no	Murray et al 2017			2017	2017	Cladogram	(duplicate)
included	included	*Ectopistes*	*migratorius*	Columbidae	KY974337		could not be verified: no ND2	verified	verified	no	Murray et al 2017			2017	2017	Cladogram	(duplicate)
included	included	*Ectopistes*	*migratorius*	Columbidae	KY974338		could not be verified: no ND2	verified	verified	no	Murray et al 2017			2017	2017	Cladogram	(duplicate)
included	included	*Ectopistes*	*migratorius*	Columbidae	KY974339		could not be verified: no ND2	verified	verified	no	Murray et al 2017			2017	2017	Cladogram	(duplicate)
included	included	*Ectopistes*	*migratorius*	Columbidae	KY974340		could not be verified: no ND2	verified	verified	no	Murray et al 2017			2017	2017	Cladogram	(duplicate)
included	included	*Ectopistes*	*migratorius*	Columbidae	KY974341		could not be verified: no ND2	verified	verified	no	Murray et al 2017			2017	2017	Cladogram	(duplicate)
included	included	*Ectopistes*	*migratorius*	Columbidae	KY974342		could not be verified: no ND2	verified	verified	no	Murray et al 2017			2017	2017	Cladogram	(duplicate)
included	included	*Ectopistes*	*migratorius*	Columbidae	KY974343		could not be verified: no ND2	verified	verified	no	Murray et al 2017			2017	2017	Cladogram	(duplicate)

Sequence database	Phylogeny database	Genus	species	family	GenBank#	RefSeq#	ND2 verified	COI verified	cyt b verified	erroneous sequence?	reference	submitted to GenBank	sequence released on GenBank	publication of paper	YEAR	Phylogeny (mitogenomic)	problematic sequences in tree
included	included	*Ectopistes*	*migratorius*	Columbidae	KY974344		could not be verified: no ND2	verified	verified	no	Murray et al 2017			2017	2017	Cladogram	(duplicate)
included	included	*Ectopistes*	*migratorius*	Columbidae	KY974345		could not be verified: no ND2	verified	verified	no	Murray et al 2017			2017	2017	Cladogram	(duplicate)
included	included	*Ectopistes*	*migratorius*	Columbidae	KY974346		could not be verified: no ND2	verified	verified	no	Murray et al 2017			2017	2017	Cladogram	(duplicate)
included	included	*Ectopistes*	*migratorius*	Columbidae	KY974347		could not be verified: no ND2	verified	verified	no	Murray et al 2017			2017	2017	Cladogram	(duplicate)
included	included	*Ectopistes*	*migratorius*	Columbidae	KY974348		could not be verified: no ND2	verified	verified	no	Murray et al 2017			2017	2017	Cladogram	(duplicate)
included	included	*Ectopistes*	*migratorius*	Columbidae	KY974349		could not be verified: no ND2	verified	verified	no	Murray et al 2017			2017	2017	Cladogram	(duplicate)
included	included	*Ectopistes*	*migratorius*	Columbidae	KY974350		could not be verified: no ND2	verified	verified	no	Murray et al 2017			2017	2017	Cladogram	(duplicate)
included	included	*Ectopistes*	*migratorius*	Columbidae	KY974351		could not be verified: no ND2	verified	verified	no	Murray et al 2017			2017	2017	Cladogram	(duplicate)
included	included	*Ectopistes*	*migratorius*	Columbidae	KY974352		could not be verified: no ND2	verified	verified	no	Murray et al 2017			2017	2017	Cladogram	(duplicate)
included	included	*Ectopistes*	*migratorius*	Columbidae	KY974353		could not be verified: no ND2	verified	verified	no	Murray et al 2017			2017	2017	Cladogram	(duplicate)
included	included	*Ectopistes*	*migratorius*	Columbidae	KY974354		could not be verified: no ND2	verified	verified	no	Murray et al 2017			2017	2017	Cladogram	(duplicate)
included	included	*Ectopistes*	*migratorius*	Columbidae	KY974355		could not be verified: no ND2	verified	verified	no	Murray et al 2017			2017	2017	Cladogram	(duplicate)
included	included	*Ectopistes*	*migratorius*	Columbidae	KY974356		could not be verified: no ND2	verified	verified	no	Murray et al 2017			2017	2017	Cladogram	(duplicate)
included	included	*Ectopistes*	*migratorius*	Columbidae	KY974357		could not be verified: no ND2	verified	verified	no	Murray et al 2017			2017	2017	Cladogram	(duplicate)
included	included	*Ectopistes*	*migratorius*	Columbidae	KY974358		could not be verified: no ND2	verified	verified	no	Murray et al 2017			2017	2017	Cladogram	(duplicate)
included	included	*Ectopistes*	*migratorius*	Columbidae	KY974359		could not be verified: no ND2	verified	verified	no	Murray et al 2017			2017	2017	Cladogram	(duplicate)
included	included	*Ectopistes*	*migratorius*	Columbidae	KY974360		could not be verified: no ND2	verified	verified	no	Murray et al 2017			2017	2017	Cladogram	(duplicate)
included	included	*Ectopistes*	*migratorius*	Columbidae	KY974361		could not be verified: no ND2	verified	verified	no	Murray et al 2017			2017	2017	Cladogram	(duplicate)

Sequence database	Phylogeny database	Genus	species	family	GenBank#	RefSeq#	ND2 verified	COI verified	cyt b verified	erroneous sequence?	reference	submitted to GenBank	sequence released on GenBank	publication of paper	YEAR	Phylogeny (mitogenomic)	problematic sequences in tree
included	included	*Ectopistes*	*migratorius*	Columbidae	KY974362		could not be verified: no ND2	verified	verified	no	Murray et al 2017			2017	2017	Cladogram	(duplicate)
included	included	*Ectopistes*	*migratorius*	Columbidae	KY974363		could not be verified: no ND2	verified	verified	no	Murray et al 2017			2017	2017	Cladogram	(duplicate)
included	included	*Ectopistes*	*migratorius*	Columbidae	KY974364		could not be verified: no ND2	verified	verified	no	Murray et al 2017			2017	2017	Cladogram	(duplicate)
included	included	*Ectopistes*	*migratorius*	Columbidae	KY974365		could not be verified: no ND2	verified	verified	no	Murray et al 2017			2017	2017	Cladogram	(duplicate)
included	included	*Ectopistes*	*migratorius*	Columbidae	KY974366		could not be verified: no ND2	verified	verified	no	Murray et al 2017			2017	2017	Cladogram	(duplicate)
included	included	*Ectopistes*	*migratorius*	Columbidae	KY974367		could not be verified: no ND2	verified	verified	no	Murray et al 2017			2017	2017	Cladogram	(duplicate)
included	included	*Ectopistes*	*migratorius*	Columbidae	KY974368		could not be verified: no ND2	verified	verified	no	Murray et al 2017			2017	2017	Cladogram	(duplicate)
included	included	*Ectopistes*	*migratorius*	Columbidae	MF595515		could not be verified: no ND2	verified	verified	no	Murray et al 2017			2017	2017	Cladogram	(duplicate)
included	included	*Ectopistes*	*migratorius*	Columbidae	MF595516		could not be verified: no ND2	verified	verified	no	Murray et al 2017			2017	2017	Cladogram	(duplicate)
included	included	*Ectopistes*	*migratorius*	Columbidae	MF595517		could not be verified: no ND2	verified	verified	no	Murray et al 2017			2017	2017	Cladogram	(duplicate)
included	included	*Ectopistes*	*migratorius*	Columbidae	MF595518		could not be verified: no ND2	verified	verified	no	Murray et al 2017			2017	2017	Cladogram	(duplicate)
included	included	*Ectopistes*	*migratorius*	Columbidae	KX902243		could not be verified: no ND2	verified	verified	no	Soares et al 2016			2016	2016	Cladogram	(duplicate)
included	included	*Ectopistes*	*migratorius*	Columbidae	KX902244		could not be verified: no ND2	verified	verified	no	Soares et al 2016			2016	2016	Cladogram	(duplicate)
included	included	*Egretta*	*eulophotes*	Ardeidae	EU072995	NC_009736	could not be verified: no ND2	verified	could not be verified: no cyt b	no	Zhou et al 2008			2008	2008	no	
included	included	*Egretta*	*eulophotes*	Ardeidae	KJ190949		could not be verified: no ND2	verified	could not be verified: no cyt b	no	Zhou et al 2014b			2014	2014	Phylogram	(duplicate)
included	included	*Egretta*	*garzetta*	Ardeidae	KJ192197	NC_023981	could not be verified: no ND2	dubious	verified	no	Zou et al 2015			2015	2015	Phylogram	-
included	included	*Egretta*	*garzetta*	Ardeidae	KJ190950		could not be verified: no ND2	could not be verified: no structure	verified	no	Zhou et al 2014b			2014	2014	Phylogram	(duplicate)
included	included	*Egretta*	*sacra*	Ardeidae	KJ190951	NC_025920	could not be verified: no ND2	could not be verified: no COI	could not be verified: no cyt b	?	Zhou et al 2014b			2014	2014	Phylogram	(duplicate)

Sequence database	Phylogeny database	Genus	species	family	GenBank#	RefSeq#	ND2 verified	COI verified	cyt b verified	erroneous sequence?	reference	submitted to GenBank	sequence released on GenBank	publication of paper	YEAR	Phylogeny (mitogenomic)	problematic sequences in tree
included	included	*Emberiza*	*aureola*	Emberizidae	KF111713	NC_022150	problematic	misidentified	misidentified	yes (misID)	Pan et al 2015a			2015	2015	no	
included	included	*Emberiza*	*chrysophrys*	Emberizidae	HQ896034	NC_015233	could not be verified: no ND2	problematic	verified	yes (seq errors/numts)	Ren et al 2014a			2014	2014	Phylogram	*Emberiza aureola, Emberiza chrysophrys*
included	included	*Emberiza*	*cioides*	Emberizidae	KF322027	NC_024524	verified	verified	verified	no	Pan et al 2015b			2015	2015	no	
included	included	*Emberiza*	*elegans*	Emberizidae	KY349099		verified	verified	verified	no	Liu et al 2019b			2019	2019	Cladogram	*Anser fabalis, Leucosticte arctoa, Emberiza aureola, Emberiza chrysophrys*
included	included	*Emberiza*	*elegans*	Emberizidae	KJ813903	NC_030368	verified	verified	verified	no	Sun et al 2016c			2016	2016	no	
included	excluded: no paper (yet)	*Emberiza*	*fucata*	Emberizidae	KT737824	NC_033338	verified	verified	verified	no	Peng, Z., Pan, T. and Zhang, B.	2015	2016	unpubl	2016	-	
included	included	*Emberiza*	*jankowskii*	Emberizidae	KP738714	NC_027251	could not be verified: no ND2	could not be verified: no COI	verified	no	Zhao et al 2016c			2016	2016	Cladogram	*Emberiza aureola, Emberiza chrysophrys*
included	included	*Emberiza*	*leucocephalos*	Emberizidae	KY349100	NC_037692	could not be verified: no structure	could not be verified: no structure	could not be verified: no structure	?	Liu et al 2019b			2019	2019	Cladogram	(duplicate)
included	included	*Emberiza*	*pallasi*	Emberizidae	MK687386		dubious	verified	dubious	yes (seq errors/numts)	Wu et al 2019b		2020	2019	2019	Phylogram	*Emberiza pallasi, Emberiza aureola, Emberiza chrysophrys*
included	included	*Emberiza*	*pusilla*	Emberizidae	KC407232	NC_021408	verified	verified	verified	no	Pan et al 2013			2013	2013	no	
included	included	*Emberiza*	*rustica*	Emberizidae	KC831775	NC_024924	verified	verified	verified	no	Chen et al 2014			2014	2014	no	
included	included	*Emberiza*	*rutila*	Emberizidae	KC952874	NC_024925	could not be verified: no ND2	verified	verified	no	Ren et al 2014b			2014	2014	no	
included	included	*Emberiza*	*siemsseni*	Emberizidae	KX809695	NC_032304	could not be verified: no ND2	verified	verified	no	Shi et al 2017b			2016	2016	Phylogram	*Leucosticte arctoa, Emberiza chrysophrys*
included	included	*Emberiza*	*spodocephala*	Emberizidae	KC758647	NC_021445	verified	verified	verified	no	Hu et al 2014			2014	2014	no	
included	included	*Emberiza*	*sulphurata*	Emberizidae	KY419885		could not be verified: no ND2	verified	verified	no	Lee et al 2017e			2017	2017	Phylogram	*Emberiza aureola, Emberiza chrysophrys*
included	included	*Emberiza*	*tristrami*	Emberizidae	HQ896035	NC_015234	verified	verified	verified	no	Kan et al 2013			2013	2013	no	
included	included	*Emberiza (Melophus)*	*lathami*	Emberizidae	KX702277	NC_031845	verified	could not be verified: no COI	could not be verified: no cyt b	no	Zhang et al 2016c			2016	2016	Phylogram	*Emberiza aureola, Emberiza chrysophrys*
included	included	*Eolophus*	*roseicapilla*	Cacatuidae	MH133971	NC_040154	verified	verified	could not be verified: no cyt b	no	Urantowka et al 2018			2018	2018	Cladogram	(duplicate)

Sequence database	Phylogeny database	Genus	species	family	GenBank#	RefSeq#	ND2 verified	COI verified	cyt b verified	erroneous sequence?	reference	submitted to GenBank	sequence released on GenBank	publication of paper	YEAR	Phylogeny (mitogenomic)	problematic sequences in tree
included	included	*Eophona*	*migratoria*	Fringillidae	KX423959	NC_031374	verified	could not be verified: no COI	verified	no	Sun et al 2016d			2016	2016	Cladogram	-
included	excluded: paper publ. after 1-1-2020	*Eophona*	*personata*	Fringillidae	KX812499		verified	verified	verified	no	Sun et al 2020e	2016	2017	2020	2017	Cladogram	-
included	included	*Eopsaltria*	*australis*	Petroicidae	JX901073	NC_019665	verified	verified	verified	no	Cooke et al 2012			2012	2012	no	
included	included	*Eopsaltria*	*australis*	Petroicidae	KM374621		verified	verified	verified	no	Morales et al 2015			2015	2015	Cladogram	-
included	included	*Eopsaltria*	*australis*	Petroicidae	KM374622		verified	verified	verified	no	Morales et al 2015			2015	2015	Cladogram	(duplicate)
included	included	*Eopsaltria*	*australis*	Petroicidae	KM374623		verified	verified	verified	no	Morales et al 2015			2015	2015	Cladogram	(duplicate)
included	included	*Eopsaltria*	*australis*	Petroicidae	KM374624		verified	verified	verified	no	Morales et al 2015			2015	2015	Cladogram	(duplicate)
included	included	*Eopsaltria*	*australis*	Petroicidae	KM374626		verified	verified	verified	no	Morales et al 2015			2015	2015	Cladogram	(duplicate)
included	included	*Eopsaltria*	*australis*	Petroicidae	KM374627		verified	verified	verified	no	Morales et al 2015			2015	2015	Cladogram	(duplicate)
included	included	*Eopsaltria*	*australis*	Petroicidae	KM374628		verified	verified	verified	no	Morales et al 2015			2015	2015	Cladogram	(duplicate)
included	included	*Eopsaltria*	*australis*	Petroicidae	KM374631		verified	verified	verified	no	Morales et al 2015			2015	2015	Cladogram	(duplicate)
included	included	*Eopsaltria*	*australis*	Petroicidae	KM374632		verified	verified	verified	no	Morales et al 2015			2015	2015	Cladogram	(duplicate)
included	included	*Eopsaltria*	*australis*	Petroicidae	KM374633		verified	verified	verified	no	Morales et al 2015			2015	2015	Cladogram	(duplicate)
included	included	*Eopsaltria*	*australis*	Petroicidae	KM374634		verified	verified	verified	no	Morales et al 2015			2015	2015	Cladogram	(duplicate)
included	included	*Eopsaltria*	*australis*	Petroicidae	KM374636		verified	verified	verified	no	Morales et al 2015			2015	2015	Cladogram	(duplicate)
included	included	*Eopsaltria*	*australis*	Petroicidae	KM374639		verified	verified	verified	no	Morales et al 2015			2015	2015	Cladogram	(duplicate)
included	included	*Eopsaltria*	*australis*	Petroicidae	KM374640		verified	verified	verified	no	Morales et al 2015			2015	2015	Cladogram	(duplicate)
included	included	*Eopsaltria*	*australis*	Petroicidae	KM374641		verified	verified	verified	no	Morales et al 2015			2015	2015	Cladogram	(duplicate)
included	included	*Eopsaltria*	*australis*	Petroicidae	KM374642		verified	verified	verified	no	Morales et al 2015			2015	2015	Cladogram	(duplicate)
included	included	*Eopsaltria*	*australis*	Petroicidae	KM374644		verified	verified	verified	no	Morales et al 2015			2015	2015	Cladogram	(duplicate)
included	included	*Eopsaltria*	*australis*	Petroicidae	KM374645		verified	verified	verified	no	Morales et al 2015			2015	2015	Cladogram	(duplicate)
included	included	*Eopsaltria*	*australis*	Petroicidae	KM374646		verified	verified	verified	no	Morales et al 2015			2015	2015	Cladogram	(duplicate)
included	included	*Eopsaltria*	*australis*	Petroicidae	KM374647		verified	verified	verified	no	Morales et al 2015			2015	2015	Cladogram	(duplicate)
included	included	*Eopsaltria*	*australis*	Petroicidae	KM374648		verified	verified	verified	no	Morales et al 2015			2015	2015	Cladogram	(duplicate)
included	included	*Eopsaltria*	*australis*	Petroicidae	KM374649		verified	verified	verified	no	Morales et al 2015			2015	2015	Cladogram	(duplicate)
included	included	*Eopsaltria*	*australis*	Petroicidae	KM374650		verified	verified	verified	no	Morales et al 2015			2015	2015	Cladogram	(duplicate)
included	included	*Eopsaltria*	*australis*	Petroicidae	KM374651		verified	verified	verified	no	Morales et al 2015			2015	2015	Cladogram	(duplicate)
included	included	*Eopsaltria*	*australis*	Petroicidae	KM374652		verified	verified	verified	no	Morales et al 2015			2015	2015	Cladogram	(duplicate)
included	included	*Eopsaltria*	*australis*	Petroicidae	KM374653		verified	verified	verified	no	Morales et al 2015			2015	2015	Cladogram	(duplicate)
included	included	*Eopsaltria*	*australis*	Petroicidae	KM374654		verified	verified	verified	no	Morales et al 2015			2015	2015	Cladogram	(duplicate)
included	included	*Eopsaltria*	*australis*	Petroicidae	KM374656		verified	verified	verified	no	Morales et al 2015			2015	2015	Cladogram	(duplicate)
included	included	*Eopsaltria*	*australis*	Petroicidae	KM374657		verified	verified	verified	no	Morales et al 2015			2015	2015	Cladogram	(duplicate)
included	included	*Eopsaltria*	*australis*	Petroicidae	KM374658		verified	verified	verified	no	Morales et al 2015			2015	2015	Cladogram	(duplicate)
included	included	*Eopsaltria*	*australis*	Petroicidae	KM374659		verified	verified	verified	no	Morales et al 2015			2015	2015	Cladogram	(duplicate)
included	included	*Eopsaltria*	*australis*	Petroicidae	KM374661		verified	verified	verified	no	Morales et al 2015			2015	2015	Cladogram	(duplicate)
included	included	*Eopsaltria*	*griseogularis*	Petroicidae	KM374625		misidentified	misidentified	misidentified	yes (misID)	Morales et al 2015			2015	2015	Cladogram	(duplicate)
included	included	*Eopsaltria*	*griseogularis*	Petroicidae	KM374629		verified	verified	could not be verified: no cyt b	no	Morales et al 2015			2015	2015	Cladogram	(duplicate)
included	included	*Eopsaltria*	*griseogularis*	Petroicidae	KM374630		verified	verified	could not be verified: no cyt b	no	Morales et al 2015			2015	2015	Cladogram	(duplicate)
included	included	*Eopsaltria*	*griseogularis*	Petroicidae	KM374643		verified	verified	could not be verified: no cyt b	no	Morales et al 2015			2015	2015	Cladogram	(duplicate)
included	included	*Eopsaltria*	*griseogularis*	Petroicidae	KM374660		verified	verified	could not be verified: no cyt b	no	Morales et al 2015			2015	2015	Cladogram	(duplicate)

Sequence database	Phylogeny database	Genus	species	family	GenBank#	RefSeq#	ND2 verified	COI verified	cyt b verified	erroneous sequence?	reference	submitted to GenBank	sequence released on GenBank	publication of paper	YEAR	Phylogeny (mitogenomic)	problematic sequences in tree
included	included	*Eopsaltria (Quoyornis)*	*georgiana*	Petroicidae	KM374635	NC_027230	verified	verified	could not be verified: no cyt b	no	Morales et al 2015			2015	2015	Cladogram	(duplicate)
included	included	*Eopsaltria (Quoyornis)*	*georgiana*	Petroicidae	KM374638		misidentified	misidentified	misidentified	yes (misID)	Morales et al 2015			2015	2015	Cladogram	(duplicate)
included	included	*Epthianura*	*albifrons*	Meliphagidae	JX901072	NC_019664	verified	could not be verified: no COI	verified	no	Cooke et al 2012			2012	2012	no	
included	included	*Eremopsaltria*	*mongolica*	Fringillidae	KM078792	NC_025616	verified	verified	verified	no	Lerner et al 2011			2011	2011	Cladogram	(duplicate)
included	included	*Erythrogenys*	*gravivox*	Timaliidae	MH737742	NC_040006	could not be verified: no ND2	could not be verified: no COI	could not be verified: no cyt b	?	Duan et al 2019			2019	2019	Phylogram	*Garrulax poecilorhynchus*
included	included	*Eudocimus*	*ruber*	Threskiornithidae	KR862292	NC_027504	verified	verified	verified	no	Chang et al 2016			2016	2016	Phylogram	-
included	included	*Eudromia*	*elegans*	Palaeognathae	AY016016		could not be verified: too few sequences of Palaeognathae	verified	verified	no	Cooper et al 2001			2001	2001	Phylogram	(duplicate)
included	included	*Eudromia*	*elegans*	Palaeognathae	AF338710	NC_002772	could not be verified: too few sequences of Palaeognathae	verified	verified	no	Haddrath & Baker 2001			2001	2001	Phylogram	(duplicate)
included	included	*Eudynamys*	*taitensis*	Cuculidae	EU410487	NC_011709	verified	verified	could not be verified: no cyt b	no	Pratt et al 2009			2009	2009	Phylogram	(duplicate)
included	included	*Eudyptes*	*chrysocome*	Spheniscidae	AP009189	NC_008138	could not be verified: no ND2	verified	verified	no	Watanabe et al 2006			2006	2006	Phylogram	-
included	included	*Eudyptes*	*chrysolophus*	Spheniscidae	MK290242		could not be verified: no ND2	could not be verified: no structure	could not be verified: no structure	?	Cole et al 2019			2019	2019	Cladogram	(duplicate)
included	included	*Eudyptes*	*filholi*	Spheniscidae	MK290245		could not be verified: no ND2	could not be verified: no structure	could not be verified: no structure	?	Cole et al 2019			2019	2019	Cladogram	(duplicate)
included	included	*Eudyptes*	*moseleyi*	Spheniscidae	MK290243		could not be verified: no ND2	verified	could not be verified: no structure	no	Cole et al 2019			2019	2019	Cladogram	(duplicate)
included	included	*Eudyptes*	*pachyrhynchus*	Spheniscidae	MK290246		could not be verified: no ND2	verified	verified	no	Cole et al 2019			2019	2019	Cladogram	(duplicate)
included	included	*Eudyptes*	*robustus*	Spheniscidae	MK290247		could not be verified: no ND2	verified	verified	no	Cole et al 2019			2019	2019	Cladogram	(duplicate)
included	included	*Eudyptes*	*schlegeli*	Spheniscidae	MK290249		could not be verified: no ND2	could not be verified: no structure	could not be verified: no structure	?	Cole et al 2019			2019	2019	Cladogram	(duplicate)
included	included	*Eudyptes*	*schlegeli*	Spheniscidae	MK290250		could not be verified: no ND2	could not be verified: no structure	could not be verified: no structure	?	Cole et al 2019			2019	2019	Cladogram	(duplicate)
included	included	*Eudyptes*	*schlegeli*	Spheniscidae	MK290251		could not be verified: no ND2	could not be verified: no structure	could not be verified: no structure	?	Cole et al 2019			2019	2019	Cladogram	(duplicate)

Sequence database	Phylogeny database	Genus	species	family	GenBank#	RefSeq#	ND2 verified	COI verified	cyt b verified	erroneous sequence?	reference	submitted to GenBank	sequence released on GenBank	publication of paper	YEAR	Phylogeny (mitogenomic)	problematic sequences in tree
included	included	*Eudyptes*	*schlegeli*	Spheniscidae	MK290252		could not be verified: no ND2	could not be verified: no structure	could not be verified: no structure	?	Cole et al 2019			2019	2019	Cladogram	(duplicate)
included	included	*Eudyptes*	*sclateri*	Spheniscidae	MK290248		could not be verified: no ND2	verified	verified	no	Cole et al 2019			2019	2019	Cladogram	(duplicate)
included	included	*Eudyptes*	*warhami*	Spheniscidae	MK290253		could not be verified: no ND2	could not be verified: no COI	could not be verified: no cyt b	?	Cole et al 2019			2019	2019	Cladogram	(duplicate)
included	included	*Eudyptes*	*warhami*	Spheniscidae	MK290254		could not be verified: no ND2	could not be verified: no COI	could not be verified: no cyt b	?	Cole et al 2019			2019	2019	Cladogram	(duplicate)
included	included	*Eudyptula*	*minor*	Spheniscidae	AF362763	NC_004538	verified	verified	verified	no	Slack et al 2003			2003	2003	network	(duplicate)
included	included	*Eudyptula*	*minor novaehollandiae*	Spheniscidae	MF370525		verified	verified	verified	no	Sarker et al 2017b			2017	2017	Cladogram	-
included	included	*Eulabeornis*	*castaneoventris*	Rallidae	KF644583	NC_025501	could not be verified: no ND2	verified	verified	no	Garcia et al 2014			2014	2014	Phylogram	*Coturnicops exquisitus*
included	included	*Euphagus*	*cyanocephalus*	Icteridae	JX516072	NC_018827	verified	verified	verified	no	Powell et al 2013			2013	2013	Cladogram	(duplicate)
included	included	*Eurynorhynchus*	*pygmeus*	Scolopacidae	KP742478	NC_027496	verified	verified	verified	no	Ge et al 2016			2016	2016	Phylogram	-
included	included	*Eurynorhynchus*	*pygmeus*	Scolopacidae	KY434065		verified	verified	verified	no	Joen et al 2017			2017	2017	Phylogram	-
included	included	*Eurystomus*	*orientalis*	Coraciidae	EU344978	NC_011716	verified	verified	verified	no	Pratt et al 2009			2009	2009	Phylogram	(duplicate)
included	included	*Falco*	*amurensis*	Falconidae	KX987839	NC_039842	verified	verified	verified	no	Yang et al 2018			2019	2019	Cladogram	*Accipiter gularis, Falco naumanni*
included	included	*Falco*	*cherrug*	Falconidae	KP337902	NC_026715	could not be verified: no structure	could not be verified: no COI	verified	no	Lu et al 2016			2016	2016	no	
included	included	*Falco*	*columbarius*	Falconidae	KM264304	NC_025579	verified	verified	verified	no	Dou et al 2016			2016	2016	no	
included	included	*Falco*	*mexicanus*	Falconidae	n.a.		dubious	dubious	verified	yes (seq errors/numts)	Doyle et al 2018			2018	2018	Cladogram	*Falco naumanni, Falco mexicanus*
included	included	*Falco*	*naumanni*	Falconidae	KM251414	NC_029846	misidentified	misidentified	misidentified	yes (misID)	Wang et al 2016c			2016	2016	Cladogram	*Falco naumanni*
included	included	*Falco*	*peregrinus*	Falconidae	AF090338	NC_000878	verified	verified	verified	no	Mindell et al 1997			1997	1997	Cladogram	-
included	excluded: no paper (yet)	*Falco*	*peregrinus*	Falconidae	JX029991		verified	verified	verified	no	Mindell, D.P., Sorenson, M.D. and Dimcheff, D.E.	2012	?	unpubl	2012	-	
included	included	*Falco*	*peregrinus*	Falconidae	JQ282801		verified	verified	verified	no	Ryu et al 2012			2012	2012	no	
included	included	*Falco*	*rusticolus*	Falconidae	KT989235	NC_029359	could not be verified: no structure	verified	verified	no	Sveinsdottir et al 2017			2017	2017	no	
included	included	*Falco*	*sparverius*	Falconidae	DQ780880	NC_008547	verified	verified	verified	no	Gibb et al 2007			2007	2007	Phylogram	(duplicate)
included	excluded: no paper (yet)	*Falco*	*tinnunculus*	Falconidae	EU196361	NC_011307	verified	verified	verified	no	Wu, J.-W., Chen, S.-F., Chen, I.-C., Tsai, C.-L. and Chou, Y.C.	2007	?	unpubl	2007	-	
included	included	*Ficedula*	*albicilla*	Muscicapidae	MN125374		verified	verified	verified	no	Zhang & Lu 2019		2019	2019	2019	no	
included	included	*Ficedula*	*albicollis*	Muscicapidae	KF293721	NC_021621	verified	verified	verified	no	Ekblom et al 2014			2014	2014	no	
included	excluded: no paper (yet)	*Ficedula*	*zanthopygia*	Muscicapidae	JN018411	NC_015802	verified	verified	verified	no	Li, X.-F. and Kan, X.-Z.	2011	?	unpubl	2011	-	
included	excluded: no paper (yet)	*Florisuga*	*fusca*	Trochilidae	KP853096	NC_030287	verified	verified	could not be verified: no cyt b	no	Prosdocimi, F., Ruschi, P. and Jennings, B.	2015	2016	unpubl	2016	-	

Sequence database	Phylogeny database	Genus	species	family	GenBank#	RefSeq#	ND2 verified	COI verified	cyt b verified	erroneous sequence?	reference	submitted to GenBank	sequence released on GenBank	publication of paper	YEAR	Phylogeny (mitogenomic)	problematic sequences in tree
included	excluded: no paper (yet)	*Florisuga*	*mellivora*	Trochilidae	KJ619588	NC_027455	verified	verified	could not be verified: no cyt b	no	Magarinos Souto, H., A McGuire, J., Mudge, J., Witt, C., Howard, J., Jarvis, E., Bryan Jennings, W. and Prosdocimi, F.	2014	?	unpubl	2014	-	
included	included	*Forpus*	*modestus*	Psittacidae	HM755882		verified	verified	verified	no	Pacheco et al 2011			2011	2011	Phylogram	(duplicate)
included	included	*Forpus*	*passerinus*	Psittacidae	KM611470	NC_027843	verified	verified	verified	no	Eberhard & Wright 2016			2016	2016	Cladogram	(duplicate)
included	included	*Francolinus*	*pintadeanus*	Phasianidae	KX196445		could not be verified: no ND2	verified	could not be verified: no cyt b	no	Li & Lin 2016			2016	2016	Phylogram	*Phasianus versicolor*
included	included	*Francolinus*	*pintadeanus*	Phasianidae	EU165707	NC_011817	could not be verified: no ND2	verified	misidentified	yes (chimera)	Shen et al 2009			2009	2009	Cladogram	(duplicate)
included	included	*Fregata*	*sp.*	Fregatidae	AP009192		verified	verified	verified	no	Watanabe et al 2006			2006	2006	Phylogram	(duplicate)
included	included	*Fringilla*	*coelebs*	Fringillidae	KM078769	NC_025599	verified	verified	verified	no	Marshall et al 2013			2013	2013	Phylogram	*Cyanoptila cyanomelana, Pseudopodoces humilis, Emberiza chrysophrys*
included	excluded: no paper (yet)	*Fringilla*	*montifringilla*	Fringillidae	JQ922259	NC_024048	verified	verified	verified	no	Qian, C.-J. and Kan, X.-Z.	2012	?	unpubl	2012	-	
included	included	*Fringilla*	*polatzeki*	Fringillidae	KU705761	NC_031157	could not be verified: no ND2	verified	verified	no	Lifjeld et al 2016			2016	2016	network	-
included	included	*Fringilla*	*polatzeki*	Fringillidae	KU705762		could not be verified: no ND2	verified	verified	no	Lifjeld et al 2016			2016	2016	network	(duplicate)
included	included	*Fringilla*	*polatzeki*	Fringillidae	KU705763		could not be verified: no ND2	verified	verified	no	Lifjeld et al 2016			2016	2016	network	(duplicate)
included	included	*Fringilla*	*polatzeki*	Fringillidae	KU705764		could not be verified: no ND2	verified	verified	no	Lifjeld et al 2016			2016	2016	network	(duplicate)
included	included	*Fringilla*	*teydea*	Fringillidae	KU705740		could not be verified: no ND2	verified	verified	no	Lifjeld et al 2016			2016	2016	network	(duplicate)
included	included	*Fringilla*	*teydea*	Fringillidae	KU705741		could not be verified: no ND2	verified	verified	no	Lifjeld et al 2016			2016	2016	network	(duplicate)
included	included	*Fringilla*	*teydea*	Fringillidae	KU705742		could not be verified: no ND2	verified	verified	no	Lifjeld et al 2016			2016	2016	network	(duplicate)
included	included	*Fringilla*	*teydea*	Fringillidae	KU705743		could not be verified: no ND2	verified	verified	no	Lifjeld et al 2016			2016	2016	network	(duplicate)

Sequence database	Phylogeny database	Genus	species	family	GenBank#	RefSeq#	ND2 verified	COI verified	cyt b verified	erroneous sequence?	reference	submitted to GenBank	sequence released on GenBank	publication of paper	YEAR	Phylogeny (mitogenomic)	problematic sequences in tree
included	included	*Fringilla*	*teydea*	Fringillidae	KU705744		could not be verified: no ND2	verified	verified	no	Lifjeld et al 2016			2016	2016	network	(duplicate)
included	included	*Fringilla*	*teydea*	Fringillidae	KU705745		could not be verified: no ND2	verified	verified	no	Lifjeld et al 2016			2016	2016	network	(duplicate)
included	included	*Fringilla*	*teydea*	Fringillidae	KU705746		could not be verified: no ND2	verified	verified	no	Lifjeld et al 2016			2016	2016	network	(duplicate)
included	included	*Fringilla*	*teydea*	Fringillidae	KU705747		could not be verified: no ND2	verified	verified	no	Lifjeld et al 2016			2016	2016	network	(duplicate)
included	included	*Fringilla*	*teydea*	Fringillidae	KU705748		could not be verified: no ND2	verified	verified	no	Lifjeld et al 2016			2016	2016	network	(duplicate)
included	included	*Fringilla*	*teydea*	Fringillidae	KU705749		could not be verified: no ND2	verified	verified	no	Lifjeld et al 2016			2016	2016	network	(duplicate)
included	included	*Fringilla*	*teydea*	Fringillidae	KU705750		could not be verified: no ND2	verified	verified	no	Lifjeld et al 2016			2016	2016	network	(duplicate)
included	included	*Fringilla*	*teydea*	Fringillidae	KU705751		verified	verified	verified	no	Lifjeld et al 2016			2016	2016	network	(duplicate)
included	included	*Fringilla*	*teydea*	Fringillidae	KU705752		verified	verified	verified	no	Lifjeld et al 2016			2016	2016	network	(duplicate)
included	included	*Fringilla*	*teydea*	Fringillidae	KU705753		could not be verified: no ND2	verified	verified	no	Lifjeld et al 2016			2016	2016	network	(duplicate)
included	included	*Fringilla*	*teydea*	Fringillidae	KU705754		could not be verified: no ND2	verified	verified	no	Lifjeld et al 2016			2016	2016	network	(duplicate)
included	included	*Fringilla*	*teydea*	Fringillidae	KU705755		could not be verified: no ND2	verified	verified	no	Lifjeld et al 2016			2016	2016	network	(duplicate)
included	included	*Fringilla*	*teydea*	Fringillidae	KU705756		could not be verified: no ND2	verified	verified	no	Lifjeld et al 2016			2016	2016	network	(duplicate)
included	included	*Fringilla*	*teydea*	Fringillidae	KU705757		could not be verified: no ND2	verified	verified	no	Lifjeld et al 2016			2016	2016	network	(duplicate)
included	included	*Fringilla*	*teydea*	Fringillidae	KU705758		could not be verified: no ND2	verified	verified	no	Lifjeld et al 2016			2016	2016	network	(duplicate)
included	included	*Fringilla*	*teydea*	Fringillidae	KU705759		verified	verified	verified	no	Lifjeld et al 2016			2016	2016	network	(duplicate)
included	included	*Fringilla*	*teydea*	Fringillidae	KU705760		verified	verified	verified	no	Lifjeld et al 2016			2016	2016	network	(duplicate)
included	included	*Fulica*	*atra*	Rallidae	KF644582	NC_025500	could not be verified: no ND2	verified	verified	no	Garcia et al 2014			2014	2014	Phylogram	(duplicate)
included	included	*Fulica*	*atra*	Rallidae	KP313718		could not be verified: no ND2	verified	verified	no	He et al 2016a			2016	2016	no	
included	excluded: no paper (yet)	*Fulica*	*atra*	Rallidae	MN122918		could not be verified: no ND2	verified	verified	no	Margaryan, A.		2019	unpubl	2019	-	

Sequence database	Phylogeny database	Genus	species	family	GenBank#	RefSeq#	ND2 verified	COI verified	cyt b verified	erroneous sequence?	reference	submitted to GenBank	sequence released on GenBank	publication of paper	YEAR	Phylogeny (mitogenomic)	problematic sequences in tree
included	excluded: paper publ. after 1-1-2020	*Fulvetta (Alcippe)*	*ruficapilla*	Timaliidae	MK988446	NC_045401	verified	verified	verified	no	Chong et al 2020		2019	2020	2019	Cladogram	-
included	included	*Galbula*	*albirostris*	Galbulidae	MK060130		dubious	verified	verified	no	Tamashiro et al 2019			2019	2019	Phylogram	(duplicate)
included	included	*Gallicolumba*	*luzonica*	Columbidae	HM746790		verified	verified	verified	no	Pacheco et al 2011			2011	2011	Phylogram	(duplicate)
included	excluded: no paper (yet)	*Gallicrex*	*cinerea*	Rallidae	KP057881	NC_028408	could not be verified: no ND2	verified	verified	no	Lee, S.H., Ryu, S.H. and Hwang, U.W.	2014	?	unpubl	2014	-	
included	excluded: no paper (yet)	*Gallinago*	*stenura*	Scolopacidae	KY888681		verified	verified	verified	no	Cheng, Y. and Zhou, L.	2017	2017	unpubl	2017	-	
included	included	*Gallinago*	*stenura*	Scolopacidae	KY056596	NC_034741	verified	verified	verified	no	Hu et al 2017			2017	2017	Phylogram	*Vanellus cinereus*
included	excluded: no paper (yet)	*Gallinula*	*chloropus*	Rallidae	HQ896036	NC_015236	verified	verified	verified	no	Kan, X.-Z. and Li, X.-F.	2011	?	unpubl	2011	-	
included	included	*Gallirallus*	*australis*	Rallidae	KF701060		could not be verified: no ND2	verified	verified	no	Garcia et al 2014			2014	2014	Phylogram	(duplicate)
included	excluded: no paper (yet)	*Gallirallus*	*australis australis*	Rallidae	KF425525		verified	verified	verified	no	Pilkington, S.	2013	?	unpubl	2013	-	
included	included	*Gallirallus*	*okinawae*	Rallidae	AP010821	NC_012140	could not be verified: no ND2	verified	could not be verified: no cyt b	no	Ozaki et al 2010			2010	2010	no	
included	included	*Gallirallus*	*philippensis*	Rallidae	KF701061	NC_025507	could not be verified: no ND2	verified	verified	no	Garcia et al 2014			2014	2014	Phylogram	(duplicate)
included	excluded: paper publ. after 1-1-2020	*Gallirallus*	*striatus*	Rallidae	MH219930	NC_041577	could not be verified: no ND2	verified	verified	no	Chen et al 2020c	2018	2019	2020	2019	Phylogram	*Amaurornis akool, Amaurornis phoenicurus, Coturnicops exquisitus, Larus vegae, Charadrius placidus, Rallus aquaticus*
included	included	*Gallus*	*gallus*	Phasianidae	KT626847		verified	verified	verified	no	Alexander et al 2015			2015	2015	no	
included	included	*Gallus*	*gallus*	Phasianidae	KT626848		verified	verified	verified	no	Alexander et al 2015			2015	2015	no	
included	included	*Gallus*	*gallus*	Phasianidae	KT626849		verified	problematic	verified	yes (seq errors/numts)	Alexander et al 2015			2015	2015	no	
included	included	*Gallus*	*gallus*	Phasianidae	KT626850		verified	verified	verified	no	Alexander et al 2015			2015	2015	no	
included	included	*Gallus*	*gallus*	Phasianidae	KT626851		verified	verified	verified	no	Alexander et al 2015			2015	2015	no	
included	included	*Gallus*	*gallus*	Phasianidae	KT626852		verified	verified	verified	no	Alexander et al 2015			2015	2015	no	

Sequence database	Phylogeny database	Genus	species	family	GenBank#	RefSeq#	ND2 verified	COI verified	cyt b verified	erroneous sequence?	reference	submitted to GenBank	sequence released on GenBank	publication of paper	YEAR	Phylogeny (mitogenomic)	problematic sequences in tree
included	included	*Gallus*	*gallus*	Phasianidae	KT626853		verified	verified	verified	no	Alexander et al 2015			2015	2015	no	
included	included	*Gallus*	*gallus*	Phasianidae	KT626854		verified	verified	verified	no	Alexander et al 2015			2015	2015	no	
included	included	*Gallus*	*gallus*	Phasianidae	KT626855		verified	verified	verified	no	Alexander et al 2015			2015	2015	no	
included	included	*Gallus*	*gallus*	Phasianidae	KT626856		verified	verified	verified	no	Alexander et al 2015			2015	2015	no	
included	included	*Gallus*	*gallus*	Phasianidae	KT626857		verified	verified	verified	no	Alexander et al 2015			2015	2015	no	
included	included	*Gallus*	*gallus*	Phasianidae	KT626858		verified	verified	verified	no	Alexander et al 2015			2015	2015	no	
included	excluded: no paper (yet)	*Gallus*	*gallus*	Phasianidae	KP211423		verified	verified	verified	no	Bhattacharya, T.K., Chatterjee, R.N. and Reddy, M.R.	2014	?	unpubl	2014	-	
included	included	*Gallus*	*gallus*	Phasianidae	X52392	NC_001323	verified	verified	verified	no	Desjardins & Morais 1990			1990	1990	no	
included	included	*Gallus*	*gallus*	Phasianidae	AY235570		verified	verified	verified	no	Froman & Kirby 2005			2005	2005	no	
included	included	*Gallus*	*gallus*	Phasianidae	AY235571		verified	verified	verified	no	Froman & Kirby 2005			2005	2005	no	
included	excluded: no paper (yet)	*Gallus*	*gallus*	Phasianidae	KT958484		verified	verified	verified	no	Hu, Y.D.	2016	2016	unpubl	2016	-	
included	excluded: no paper (yet)	*Gallus*	*gallus*	Phasianidae	MK163559		verified	verified	verified	no	Kong, M. and Zhao, X.		2019	unpubl	2019	-	
included	excluded: no paper (yet)	*Gallus*	*gallus*	Phasianidae	MK163560		verified	verified	verified	no	Kong, M. and Zhao, X.		2019	unpubl	2019	-	
included	excluded: no paper (yet)	*Gallus*	*gallus*	Phasianidae	MK163561		verified	verified	verified	no	Kong, M. and Zhao, X.		2019	unpubl	2019	-	
included	excluded: no paper (yet)	*Gallus*	*gallus*	Phasianidae	MK163562		verified	verified	verified	no	Kong, M. and Zhao, X.		2019	unpubl	2019	-	
included	excluded: no paper (yet)	*Gallus*	*gallus*	Phasianidae	MK163563		verified	verified	verified	no	Kong, M. and Zhao, X.		2019	unpubl	2019	-	
included	excluded: no paper (yet)	*Gallus*	*gallus*	Phasianidae	MK163564		verified	verified	verified	no	Kong, M. and Zhao, X.		2019	unpubl	2019	-	
included	excluded: no paper (yet)	*Gallus*	*gallus*	Phasianidae	MK163565		verified	verified	verified	no	Kong, M. and Zhao, X.		2019	unpubl	2019	-	
included	included	*Gallus*	*gallus*	Phasianidae	MG837547	NC_040970	verified	verified	verified	no	Liu et al 2018f			2018	2018	no	
included	included	*Gallus*	*gallus*	Phasianidae	MG837548		verified	verified	verified	no	Liu et al 2018f			2018	2018	no	
included	excluded: no paper (yet)	*Gallus*	*gallus*	Phasianidae	HQ857209		verified	verified	verified	no	Miao, Y., Peng, M.-S. and Zhang, Y.-P.	2010	?	unpubl	2010	-	
included	excluded: no paper (yet)	*Gallus*	*gallus*	Phasianidae	HQ857210		verified	verified	verified	no	Miao, Y., Peng, M.-S. and Zhang, Y.-P.	2010	?	unpubl	2010	-	

Sequence database	Phylogeny database	Genus	species	family	GenBank#	RefSeq#	ND2 verified	COI verified	cyt b verified	erroneous sequence?	reference	submitted to GenBank	sequence released on GenBank	publication of paper	YEAR	Phylogeny (mitogenomic)	problematic sequences in tree
included	excluded: no paper (yet)	*Gallus*	*gallus*	Phasianidae	HQ857211		verified	verified	verified	no	Miao, Y., Peng, M.-S. and Zhang, Y.-P.	2010	?	unpubl	2010	-	
included	excluded: no paper (yet)	*Gallus*	*gallus*	Phasianidae	HQ857212		verified	verified	verified	no	Miao, Y., Peng, M.-S. and Zhang, Y.-P.	2010	?	unpubl	2010	-	
included	excluded: no paper (yet)	*Gallus*	*gallus*	Phasianidae	KY094500		verified	verified	verified	no	Nguyen, D.T., Nguyen, H.H., Nguyen, T.H., Ma, T.H.T., Vu, P.N., Tran, T.B.N., Nguyen, T.D. and Nong, V.H.	2017	2017	unpubl	2017	-	
included	included	*Gallus*	*gallus*	Phasianidae	AP003580		verified	verified	verified	no	Nishibori et al 2003			2003	2003	no	
included	included	*Gallus*	*gallus*	Phasianidae	AP003317		verified	verified	verified	no	Nishibori et al 2005			2005	2005	Phylogram	-
included	included	*Gallus*	*gallus*	Phasianidae	AP003318		verified	verified	verified	no	Nishibori et al 2005			2005	2005	Phylogram	(duplicate)
included	included	*Gallus*	*gallus*	Phasianidae	AP003319		verified	verified	verified	no	Nishibori et al 2005			2005	2005	Phylogram	(duplicate)
included	included	*Gallus*	*gallus*	Phasianidae	AP003322		verified	verified	verified	no	Nishibori et al 2005			2005	2005	Phylogram	(duplicate)
included	included	*Gallus*	*gallus*	Phasianidae	MG605671		verified	verified	verified	no	Suwannapoom et al 2018			2018	2018	Cladogram	-
included	included	*Gallus*	*gallus*	Phasianidae	KJ778617		verified	verified	verified	no	Wang et al 2016f			2016	2016	no	
included	included	*Gallus*	*gallus*	Phasianidae	MN013407		verified	verified	verified	no	Wang et al 2019c		2019	2019	2019	Phylogram	-
included	excluded: no paper (yet)	*Gallus*	*gallus (Alubijid Philippines)*	Phasianidae	KY039397		verified	verified	verified	no	Herrera, M.B., Thomson, V.A., Wadley, J.J., Macqueen, P. et al	2016	2018	unpubl	2018	-	
included	excluded: no paper (yet)	*Gallus*	*gallus (Aseel)*	Phasianidae	KP211418		verified	verified	verified	no	Bhattacharya, T.K., Chatterjee, R.N. and Reddy, M.R.	2014	?	unpubl	2014	-	
included	excluded: no paper (yet)	*Gallus*	*gallus (Asipulo Philippines)*	Phasianidae	KY039408		verified	verified	verified	no	Herrera, M.B., Thomson, V.A., Wadley, J.J., Macqueen, P. et al	2016	2018	unpubl	2018	-	
included	included	*Gallus*	*gallus (Autochthonic)*	Phasianidae	GU261679		verified	verified	verified	no	Miao et al 2013			2013	2013	cladogram	-
included	included	*Gallus*	*gallus (Autochthonic)*	Phasianidae	GU261680		verified	verified	verified	no	Miao et al 2013			2013	2013	cladogram	(duplicate)
included	included	*Gallus*	*gallus (Autochthonic)*	Phasianidae	GU261681		verified	verified	verified	no	Miao et al 2013			2013	2013	cladogram	(duplicate)
included	included	*Gallus*	*gallus (Autochthonic)*	Phasianidae	GU261682		verified	verified	verified	no	Miao et al 2013			2013	2013	cladogram	(duplicate)
included	included	*Gallus*	*gallus (Autochthonic)*	Phasianidae	GU261685		verified	verified	verified	no	Miao et al 2013			2013	2013	cladogram	(duplicate)
included	included	*Gallus*	*gallus (Autochthonic)*	Phasianidae	GU261686		verified	verified	verified	no	Miao et al 2013			2013	2013	cladogram	(duplicate)
included	included	*Gallus*	*gallus (Autochthonic)*	Phasianidae	GU261687		verified	verified	verified	no	Miao et al 2013			2013	2013	cladogram	(duplicate)

Sequence database	Phylogeny database	Genus	species	family	GenBank#	RefSeq#	ND2 verified	COI verified	cyt b verified	erroneous sequence?	reference	submitted to GenBank	sequence released on GenBank	publication of paper	YEAR	Phylogeny (mitogenomic)	problematic sequences in tree
included	included	*Gallus*	*gallus (Autochthonic)*	Phasianidae	GU261691		verified	verified	verified	no	Miao et al 2013			2013	2013	cladogram	(duplicate)
included	included	*Gallus*	*gallus (Autochthonic)*	Phasianidae	GU261694		verified	verified	verified	no	Miao et al 2013			2013	2013	cladogram	(duplicate)
included	included	*Gallus*	*gallus (Autochthonic)*	Phasianidae	GU261697		verified	verified	verified	no	Miao et al 2013			2013	2013	cladogram	(duplicate)
included	included	*Gallus*	*gallus (Autochthonic)*	Phasianidae	GU261698		verified	verified	verified	no	Miao et al 2013			2013	2013	cladogram	(duplicate)
included	included	*Gallus*	*gallus (Autochthonic)*	Phasianidae	GU261700		verified	verified	verified	no	Miao et al 2013			2013	2013	cladogram	(duplicate)
included	excluded: no paper (yet)	*Gallus*	*gallus (Batanes Philippines)*	Phasianidae	KY039410		verified	verified	verified	no	Herrera, M.B., Thomson, V.A., Wadley, J.J., Macqueen, P. et al	2016	2018	unpubl	2018	-	
included	excluded: no paper (yet)	*Gallus*	*gallus (Cagayan Valley Philippines)*	Phasianidae	KY039415		verified	verified	verified	no	Herrera, M.B., Thomson, V.A., Wadley, J.J., Macqueen, P. et al	2016	2018	unpubl	2018	-	
included	included	*Gallus*	*gallus (Cenxi classical three-buff)*	Phasianidae	KM433666		verified	verified	verified	no	Xie et al 2016d			2016	2016	no	
included	included	*Gallus*	*gallus (Chigulu)*	Phasianidae	GU261684		verified	verified	verified	no	Miao et al 2013			2013	2013	cladogram	(duplicate)
included	included	*Gallus*	*gallus (Chigulu)*	Phasianidae	GU261705		verified	verified	verified	no	Miao et al 2013			2013	2013	cladogram	(duplicate)
included	included	*Gallus*	*gallus (Chigulu)*	Phasianidae	GU261715		verified	verified	verified	no	Miao et al 2013			2013	2013	cladogram	(duplicate)
included	included	*Gallus*	*gallus (Chigulu)*	Phasianidae	GU261717		verified	verified	verified	no	Miao et al 2013			2013	2013	cladogram	(duplicate)
included	included	*Gallus*	*gallus (Chigulu)*	Phasianidae	GU261719		verified	verified	verified	no	Miao et al 2013			2013	2013	cladogram	(duplicate)
included	excluded: no paper (yet)	*Gallus*	*gallus (Cotabato Philippines)*	Phasianidae	KY039398		verified	verified	verified	no	Herrera, M.B., Thomson, V.A., Wadley, J.J., Macqueen, P. et al	2016	2018	unpubl	2018	-	
included	excluded: no paper (yet)	*Gallus*	*gallus (Dagu)*	Phasianidae	KT283576		verified	verified	verified	no	Gu, J. and Li, S.	2014	?	unpubl	2014	-	
included	included	*Gallus*	*gallus (Daweishan Mini)*	Phasianidae	KF939304		verified	verified	verified	no	Yan et al 2016c			2016	2016	no	
included	included	*Gallus*	*gallus (Dong An black)*	Phasianidae	KM886936		verified	verified	verified	no	Peng et al 2018			2018	2018	phylogram	-
included	included	*Gallus*	*gallus (Dong An yellow)*	Phasianidae	KM886937		verified	verified	verified	no	Lin et al 2019d			2019	2019	phylogram	-
included	excluded: no paper (yet)	*Gallus*	*gallus (Easter Island)*	Phasianidae	KY039386		verified	verified	verified	no	Herrera, M.B., Thomson, V.A., Wadley, J.J., Macqueen, P. et al	2016	2018	unpubl	2018	-	
included	excluded: no paper (yet)	*Gallus*	*gallus (Easter Island)*	Phasianidae	KY039387		verified	verified	verified	no	Herrera, M.B., Thomson, V.A., Wadley, J.J., Macqueen, P. et al	2016	2018	unpubl	2018	-	
included	excluded: no paper (yet)	*Gallus*	*gallus (Easter Island)*	Phasianidae	KY039388		verified	verified	verified	no	Herrera, M.B., Thomson, V.A., Wadley, J.J., Macqueen, P. et al	2016	2018	unpubl	2018	-	

Sequence database	Phylogeny database	Genus	species	family	GenBank#	RefSeq#	ND2 verified	COI verified	cyt b verified	erroneous sequence?	reference	submitted to GenBank	sequence released on GenBank	publication of paper	YEAR	Phylogeny (mitogenomic)	problematic sequences in tree
included	excluded: no paper (yet)	*Gallus*	*gallus (Easter Island)*	Phasianidae	KY039389		verified	verified	verified	no	Herrera, M.B., Thomson, V.A., Wadley, J.J., Macqueen, P. et al	2016	2018	unpubl	2018	-	
included	excluded: no paper (yet)	*Gallus*	*gallus (Easter Island)*	Phasianidae	KY039390		verified	verified	verified	no	Herrera, M.B., Thomson, V.A., Wadley, J.J., Macqueen, P. et al	2016	2018	unpubl	2018	-	
included	excluded: no paper (yet)	*Gallus*	*gallus (Fiji)*	Phasianidae	KY039391		verified	verified	verified	no	Herrera, M.B., Thomson, V.A., Wadley, J.J., Macqueen, P. et al	2016	2018	unpubl	2018	-	
included	excluded: no paper (yet)	*Gallus*	*gallus (Garut Java Indonesia)*	Phasianidae	KY039418		verified	verified	verified	no	Herrera, M.B., Thomson, V.A., Wadley, J.J., Macqueen, P. et al	2016	2018	unpubl	2018	-	
included	excluded: no paper (yet)	*Gallus*	*gallus (Ghagus)*	Phasianidae	KP211419		verified	verified	verified	no	Bhattacharya, T.K., Chatterjee, R.N. and Reddy, M.R.	2014	?	unpubl	2014	-	
included	excluded: no paper (yet)	*Gallus*	*gallus (Goma Sulawesi Indonesia)*	Phasianidae	KY039429		verified	verified	verified	no	Herrera, M.B., Thomson, V.A., Wadley, J.J., Macqueen, P. et al	2016	2018	unpubl	2018	-	
included	included	*Gallus*	*gallus (Gongxi)*	Phasianidae	MH879470		verified	verified	verified	no	Yan et al 2019			2019	2019	Phylogram	-
included	excluded: no paper (yet)	*Gallus*	*gallus (Guangxi Partridge)*	Phasianidae	KP681580		verified	verified	verified	no	Zhang, Y.F., Xie, Z.X., Liu, J.B., Deng, X.W., Xie, Z.Q., Huang, L., Huang, J.L., Zeng, T.T. and Wang, S.	2015	2015	unpubl	2015	-	
included	excluded: no paper (yet)	*Gallus*	*gallus (Guangxi three-buff)*	Phasianidae	KP681581		verified	verified	verified	no	Zhang, Y.F., Xie, Z.X., Liu, J.B., Deng, X.W., Xie, Z.Q., Huang, L., Huang, J.L., Zeng, T.T. and Wang, S.	2015	2015	unpubl	2015	-	
included	included	*Gallus*	*gallus (Gushi)*	Phasianidae	GU261678		verified	verified	verified	no	Miao et al 2013			2013	2013	cladogram	(duplicate)
included	excluded: no paper (yet)	*Gallus*	*gallus (Hainan)*	Phasianidae	KY039433		verified	verified	verified	no	Herrera, M.B., Thomson, V.A., Wadley, J.J., Macqueen, P. et al	2016	2018	unpubl	2018	-	
included	excluded: no paper (yet)	*Gallus*	*gallus (Hainan)*	Phasianidae	KY039434		verified	verified	verified	no	Herrera, M.B., Thomson, V.A., Wadley, J.J., Macqueen, P. et al	2016	2018	unpubl	2018	-	
included	excluded: no paper (yet)	*Gallus*	*gallus (Hainan)*	Phasianidae	KY039436		verified	verified	verified	no	Herrera, M.B., Thomson, V.A., Wadley, J.J., Macqueen, P. et al	2016	2018	unpubl	2018	-	
included	excluded: no paper (yet)	*Gallus*	*gallus (Haringhata black)*	Phasianidae	KP211420		verified	verified	verified	no	Bhattacharya, T.K., Chatterjee, R.N. and Reddy, M.R.	2014	?	unpubl	2014	-	

Sequence database	Phylogeny database	Genus	species	family	GenBank#	RefSeq#	ND2 verified	COI verified	cyt b verified	erroneous sequence?	reference	submitted to GenBank	sequence released on GenBank	publication of paper	YEAR	Phylogeny (mitogenomic)	problematic sequences in tree
included	excluded: no paper (yet)	*Gallus*	*gallus (Hawaii)*	Phasianidae	KY039383		verified	verified	verified	no	Herrera, M.B., Thomson, V.A., Wadley, J.J., Macqueen, P. et al	2016	2018	unpubl	2018	-	
included	excluded: no paper (yet)	*Gallus*	*gallus (Hawaii)*	Phasianidae	KY039384		verified	verified	verified	no	Herrera, M.B., Thomson, V.A., Wadley, J.J., Macqueen, P. et al	2016	2018	unpubl	2018	-	
included	excluded: no paper (yet)	*Gallus*	*gallus (Hawaii)*	Phasianidae	KY039385		verified	verified	verified	no	Herrera, M.B., Thomson, V.A., Wadley, J.J., Macqueen, P. et al	2016	2018	unpubl	2018	-	
included	excluded: no paper (yet)	*Gallus*	*gallus (Hengshan Yellow)*	Phasianidae	KP244335		verified	verified	verified	no	He, J.	2014	?	unpubl	2014	-	
included	included	*Gallus*	*gallus (Huaiyang)*	Phasianidae	GU261701		verified	verified	verified	no	Miao et al 2013			2013	2013	cladogram	(duplicate)
included	included	*Gallus*	*gallus (Huang Lang)*	Phasianidae	KF954727		verified	verified	verified	no	Yu et al 2016b			2016	2016	no	
included	excluded: no paper (yet)	*Gallus*	*gallus (Ifugao Philippines)*	Phasianidae	KY039404		verified	verified	verified	no	Herrera, M.B., Thomson, V.A., Wadley, J.J., Macqueen, P. et al	2016	2018	unpubl	2018	-	
included	excluded: no paper (yet)	*Gallus*	*gallus (Ifugao Philippines)*	Phasianidae	KY039405		verified	verified	verified	no	Herrera, M.B., Thomson, V.A., Wadley, J.J., Macqueen, P. et al	2016	2018	unpubl	2018	-	
included	excluded: no paper (yet)	*Gallus*	*gallus (Ifugao Philippines)*	Phasianidae	KY039406		verified	verified	verified	no	Herrera, M.B., Thomson, V.A., Wadley, J.J., Macqueen, P. et al	2016	2018	unpubl	2018	-	
included	excluded: no paper (yet)	*Gallus*	*gallus (Ifugao Philippines)*	Phasianidae	KY039407		verified	verified	verified	no	Herrera, M.B., Thomson, V.A., Wadley, J.J., Macqueen, P. et al	2016	2018	unpubl	2018	-	
included	excluded: no paper (yet)	*Gallus*	*gallus (Ifugao Philippines)*	Phasianidae	KY039409		verified	verified	verified	no	Herrera, M.B., Thomson, V.A., Wadley, J.J., Macqueen, P. et al	2016	2018	unpubl	2018	-	
included	included	*Gallus*	*gallus (Jiangbian)*	Phasianidae	GU261713		verified	verified	verified	no	Miao et al 2013			2013	2013	cladogram	(duplicate)
included	included	*Gallus*	*gallus (Jiangbian)*	Phasianidae	GU261714		verified	verified	verified	no	Miao et al 2013			2013	2013	cladogram	(duplicate)
included	excluded: no paper (yet)	*Gallus*	*gallus (Jinhu Wufeng)*	Phasianidae	KR347464		verified	verified	verified	no	Zhao, F. and Fan, H.	2015	2015	unpubl	2015	-	
included	excluded: no paper (yet)	*Gallus*	*gallus (Kab Manokwari Indonesia)*	Phasianidae	KY039423		verified	verified	verified	no	Herrera, M.B., Thomson, V.A., Wadley, J.J., Macqueen, P. et al	2016	2018	unpubl	2018	-	
included	excluded: no paper (yet)	*Gallus*	*gallus (Kab Manokwari Indonesia)*	Phasianidae	KY039424		verified	verified	verified	no	Herrera, M.B., Thomson, V.A., Wadley, J.J., Macqueen, P. et al	2016	2018	unpubl	2018	-	

Sequence database	Phylogeny database	Genus	species	family	GenBank#	RefSeq#	ND2 verified	COI verified	cyt b verified	erroneous sequence?	reference	submitted to GenBank	sequence released on GenBank	publication of paper	YEAR	Phylogeny (mitogenomic)	problematic sequences in tree
included	excluded: no paper (yet)	*Gallus*	*gallus (Kadaknath)*	Phasianidae	KP211425		verified	verified	verified	no	Bhattacharya, T.K., Chatterjee, R.N. and Reddy, M.R.	2014	?	unpubl	2014	-	
included	excluded: no paper (yet)	*Gallus*	*gallus (Kendu Java Indonesia)*	Phasianidae	KY039419		verified	verified	verified	no	Herrera, M.B., Thomson, V.A., Wadley, J.J., Macqueen, P. et al	2016	2018	unpubl	2018	-	
included	excluded: no paper (yet)	*Gallus*	*gallus (Kendu Java Indonesia)*	Phasianidae	KY039420		verified	verified	verified	no	Herrera, M.B., Thomson, V.A., Wadley, J.J., Macqueen, P. et al	2016	2018	unpubl	2018	-	
included	included	*Gallus*	*gallus (Lindian)*	Phasianidae	MH732978		verified	verified	verified	no	Xu et al 2018			2018	2018	phylogram	-
included	excluded: no paper (yet)	*Gallus*	*gallus (Lombok Indonesia)*	Phasianidae	KY039426		verified	verified	verified	no	Herrera, M.B., Thomson, V.A., Wadley, J.J., Macqueen, P. et al	2016	2018	unpubl	2018	-	
included	excluded: no paper (yet)	*Gallus*	*gallus (Long Island New Guinea)*	Phasianidae	KY039394		verified	verified	verified	no	Herrera, M.B., Thomson, V.A., Wadley, J.J., Macqueen, P. et al	2016	2018	unpubl	2018	-	
included	included	*Gallus*	*gallus (Lueyang Black-boned)*	Phasianidae	KY054997		verified	verified	verified	no	Zhang et al 2018c			2018	2018	Cladogram	-
included	included	*Gallus*	*gallus (Lverwu)*	Phasianidae	GU261712		verified	verified	verified	no	Miao et al 2013			2013	2013	cladogram	(duplicate)
included	included	*Gallus*	*gallus (Lverwu)*	Phasianidae	GU261718		verified	verified	verified	no	Miao et al 2013			2013	2013	cladogram	(duplicate)
included	excluded: no paper (yet)	*Gallus*	*gallus (Malacca)*	Phasianidae	KY039435		verified	verified	verified	no	Herrera, M.B., Thomson, V.A., Wadley, J.J., Macqueen, P. et al	2016	2018	unpubl	2018	-	
included	excluded: no paper (yet)	*Gallus*	*gallus (Maluku Indonesia)*	Phasianidae	KY039395		verified	verified	verified	no	Herrera, M.B., Thomson, V.A., Wadley, J.J., Macqueen, P. et al	2016	2018	unpubl	2018	-	
included	excluded: no paper (yet)	*Gallus*	*gallus (Maluku Indonesia)*	Phasianidae	KY039425		verified	verified	verified	no	Herrera, M.B., Thomson, V.A., Wadley, J.J., Macqueen, P. et al	2016	2018	unpubl	2018	-	
included	excluded: no paper (yet)	*Gallus*	*gallus (Manado Sulawesi Indonesia)*	Phasianidae	KY039427		verified	verified	verified	no	Herrera, M.B., Thomson, V.A., Wadley, J.J., Macqueen, P. et al	2016	2018	unpubl	2018	-	
included	excluded: no paper (yet)	*Gallus*	*gallus (Manado Sulawesi Indonesia)*	Phasianidae	KY039428		verified	verified	verified	no	Herrera, M.B., Thomson, V.A., Wadley, J.J., Macqueen, P. et al	2016	2018	unpubl	2018	-	
included	excluded: no paper (yet)	*Gallus*	*gallus (Manticao Philippines)*	Phasianidae	KY039396		verified	verified	verified	no	Herrera, M.B., Thomson, V.A., Wadley, J.J., Macqueen, P. et al	2016	2018	unpubl	2018	-	
included	excluded: no paper (yet)	*Gallus*	*gallus (Marquesas)*	Phasianidae	KY039381		verified	verified	verified	no	Herrera, M.B., Thomson, V.A., Wadley, J.J., Macqueen, P. et al	2016	2018	unpubl	2018	-	

Sequence database	Phylogeny database	Genus	species	family	GenBank#	RefSeq#	ND2 verified	COI verified	cyt b verified	erroneous sequence?	reference	submitted to GenBank	sequence released on GenBank	publication of paper	YEAR	Phylogeny (mitogenomic)	problematic sequences in tree
included	excluded: no paper (yet)	*Gallus*	*gallus (Mindoro Philippines)*	Phasianidae	KY039416		verified	verified	verified	no	Herrera, M.B., Thomson, V.A., Wadley, J.J., Macqueen, P. et al	2016	2018	unpubl	2018	-	
included	excluded: no paper (yet)	*Gallus*	*gallus (Mindoro Philippines)*	Phasianidae	KY039417		verified	verified	verified	no	Herrera, M.B., Thomson, V.A., Wadley, J.J., Macqueen, P. et al	2016	2018	unpubl	2018	-	
included	excluded: no paper (yet)	*Gallus*	*gallus (Nandan)*	Phasianidae	KP269069		verified	verified	verified	no	Zhang, Y.F., Xie, Z.X., Liu, J.B., Deng, X.W., Xie, Z.Q., Huang, L., Huang, J.L., Zeng, T.T. and Wang, S.	2014	?	unpubl	2014	-	
included	excluded: no paper (yet)	*Gallus*	*gallus (New Caledonia)*	Phasianidae	KY039431		verified	verified	verified	no	Herrera, M.B., Thomson, V.A., Wadley, J.J., Macqueen, P. et al	2016	2018	unpubl	2018	-	
included	excluded: no paper (yet)	*Gallus*	*gallus (New Caledonia)*	Phasianidae	KY039432		verified	verified	verified	no	Herrera, M.B., Thomson, V.A., Wadley, J.J., Macqueen, P. et al	2016	2018	unpubl	2018	-	
included	excluded: no paper (yet)	*Gallus*	*gallus (Nicobari Black)*	Phasianidae	KP211421		verified	verified	verified	no	Bhattacharya, T.K., Chatterjee, R.N. and Reddy, M.R.	2014	2015	unpubl	2015	-	
included	excluded: no paper (yet)	*Gallus*	*gallus (Nicobari Brown)*	Phasianidae	KP211422		verified	verified	verified	no	Bhattacharya, T.K., Chatterjee, R.N. and Reddy, M.R.	2014	?	unpubl	2014	-	
included	excluded: no paper (yet)	*Gallus*	*gallus (Niue)*	Phasianidae	KY039382		verified	verified	verified	no	Herrera, M.B., Thomson, V.A., Wadley, J.J., Macqueen, P. et al	2016	2018	unpubl	2018	-	
included	included	*Gallus*	*gallus (Nixi)*	Phasianidae	GU261710		verified	verified	verified	no	Miao et al 2013			2013	2013	cladogram	(duplicate)
included	included	*Gallus*	*gallus (Nixi)*	Phasianidae	GU261711		verified	verified	verified	no	Miao et al 2013			2013	2013	cladogram	(duplicate)
included	excluded: no paper (yet)	*Gallus*	*gallus (Nunukan Kalimantan Indonesia)*	Phasianidae	KY039422		verified	verified	verified	no	Herrera, M.B., Thomson, V.A., Wadley, J.J., Macqueen, P. et al	2016	2018	unpubl	2018	-	
included	excluded: no paper (yet)	*Gallus*	*gallus (Palawan Philippines)*	Phasianidae	KY039411		verified	verified	verified	no	Herrera, M.B., Thomson, V.A., Wadley, J.J., Macqueen, P. et al	2016	2018	unpubl	2018	-	
included	excluded: no paper (yet)	*Gallus*	*gallus (Palawan Philippines)*	Phasianidae	KY039412		verified	verified	verified	no	Herrera, M.B., Thomson, V.A., Wadley, J.J., Macqueen, P. et al	2016	2018	unpubl	2018	-	
included	excluded: no paper (yet)	*Gallus*	*gallus (Palawan Philippines)*	Phasianidae	KY039413		verified	verified	verified	no	Herrera, M.B., Thomson, V.A., Wadley, J.J., Macqueen, P. et al	2016	2018	unpubl	2018	-	

Sequence database	Phylogeny database	Genus	species	family	GenBank#	RefSeq#	ND2 verified	COI verified	cyt b verified	erroneous sequence?	reference	submitted to GenBank	sequence released on GenBank	publication of paper	YEAR	Phylogeny (mitogenomic)	problematic sequences in tree
included	excluded: no paper (yet)	*Gallus*	*gallus (Palawan Philippines)*	Phasianidae	KY039414		verified	verified	verified	no	Herrera, M.B., Thomson, V.A., Wadley, J.J., Macqueen, P. et al	2016	2018	unpubl	2018	-	
included	excluded: no paper (yet)	*Gallus*	*gallus (Palawan Philippines)*	Phasianidae	KY039437		verified	verified	verified	no	Herrera, M.B., Thomson, V.A., Wadley, J.J., Macqueen, P. et al	2016	2018	unpubl	2018	-	
included	excluded: no paper (yet)	*Gallus*	*gallus (Rugao yellow)*	Phasianidae	KP742951		verified	verified	verified	no	Zhao, F. and Fan, H.	2015	2015	unpubl	2015	-	
included	included	*Gallus*	*gallus (Taoyuan)*	Phasianidae	KF981434		verified	verified	verified	no	Liu et al 2016h			2016	2016	no	
included	excluded: no paper (yet)	*Gallus*	*gallus (Tarakan Kalimantan Indonesia)*	Phasianidae	KY039421		verified	verified	verified	no	Herrera, M.B., Thomson, V.A., Wadley, J.J., Macqueen, P. et al	2016	2018	unpubl	2018	-	
included	excluded: no paper (yet)	*Gallus*	*gallus (Tellicherry)*	Phasianidae	KP211424		verified	verified	verified	no	Bhattacharya, T.K., Chatterjee, R.N. and Reddy, M.R.	2014	?	unpubl	2014	-	
included	included	*Gallus*	*gallus (Tengchongxue)*	Phasianidae	GU261688		verified	verified	verified	no	Miao et al 2013			2013	2013	cladogram	(duplicate)
included	included	*Gallus*	*gallus (Tengchongxue)*	Phasianidae	GU261689		verified	verified	verified	no	Miao et al 2013			2013	2013	cladogram	(duplicate)
included	included	*Gallus*	*gallus (Tibetan)*	Phasianidae	DQ648776		verified	verified	verified	no	Tong et al 2006			2006	2006	xxxREQUESTED ResearchGateXXX	*???????*
included	excluded: no paper (yet)	*Gallus*	*gallus (Tugop Philippines)*	Phasianidae	KY039399		verified	verified	verified	no	Herrera, M.B., Thomson, V.A., Wadley, J.J., Macqueen, P. et al	2016	2018	unpubl	2018	-	
included	excluded: no paper (yet)	*Gallus*	*gallus (Tugop Philippines)*	Phasianidae	KY039400		verified	verified	verified	no	Herrera, M.B., Thomson, V.A., Wadley, J.J., Macqueen, P. et al	2016	2018	unpubl	2018	-	
included	excluded: no paper (yet)	*Gallus*	*gallus (Tugop Philippines)*	Phasianidae	KY039401		verified	verified	verified	no	Herrera, M.B., Thomson, V.A., Wadley, J.J., Macqueen, P. et al	2016	2018	unpubl	2018	-	
included	included	*Gallus*	*gallus (Tulufan)*	Phasianidae	GU261683		verified	verified	verified	no	Miao et al 2013			2013	2013	cladogram	(duplicate)
included	excluded: no paper (yet)	*Gallus*	*gallus (Vanuatu)*	Phasianidae	KY039392		verified	verified	verified	no	Herrera, M.B., Thomson, V.A., Wadley, J.J., Macqueen, P. et al	2016	2018	unpubl	2018	-	
included	excluded: no paper (yet)	*Gallus*	*gallus (Vanuatu)*	Phasianidae	KY039393		verified	verified	verified	no	Herrera, M.B., Thomson, V.A., Wadley, J.J., Macqueen, P. et al	2016	2018	unpubl	2018	-	
included	excluded: no paper (yet)	*Gallus*	*gallus (Vietnam)*	Phasianidae	KY039430		verified	verified	verified	no	Herrera, M.B., Thomson, V.A., Wadley, J.J., Macqueen, P. et al	2016	2018	unpubl	2018	-	
included	included	*Gallus*	*gallus (Wenshanshandi)*	Phasianidae	GU261699		verified	verified	verified	no	Miao et al 2013			2013	2013	cladogram	(duplicate)
included	included	*Gallus*	*gallus (Wuding)*	Phasianidae	GU261676		verified	verified	verified	no	Miao et al 2013			2013	2013	cladogram	(duplicate)

Sequence database	Phylogeny database	Genus	species	family	GenBank#	RefSeq#	ND2 verified	COI verified	cyt b verified	erroneous sequence?	reference	submitted to GenBank	sequence released on GenBank	publication of paper	YEAR	Phylogeny (mitogenomic)	problematic sequences in tree
included	included	*Gallus*	*gallus (Xianju)*	Phasianidae	GU261677		verified	verified	verified	no	Miao et al 2013			2013	2013	cladogram	(duplicate)
included	excluded: no paper (yet)	*Gallus*	*gallus (Xiaoxiang)*	Phasianidae	KX781319		verified	verified	verified	no	Liu, L., Zhu, X., Yang, Y., Liu, Y., Yang, N., Xie, D. and Huang, Z.	2016	2016	unpubl	2016	-	
included	included	*Gallus*	*gallus (Xuefeng black-boned)*	Phasianidae	KF826490		verified	verified	verified	no	Liu et al 2016g			2016	2016	no	
included	included	*Gallus*	*gallus (Xuefeng)*	Phasianidae	GU261675		verified	verified	verified	no	Miao et al 2013			2013	2013	cladogram	(duplicate)
included	included	*Gallus*	*gallus (Yeonsan Ogye)*	Phasianidae	CM008858		verified	verified	verified	no	Sohn et al 2018			2018	2018	no	
included	excluded: no paper (yet)	*Gallus*	*gallus (Zamboanga Philippines)*	Phasianidae	KY039402		verified	verified	verified	no	Herrera, M.B., Thomson, V.A., Wadley, J.J., Macqueen, P. et al	2016	2018	unpubl	2018	-	
included	excluded: no paper (yet)	*Gallus*	*gallus (Zamboanga Philippines)*	Phasianidae	KY039403		verified	verified	verified	no	Herrera, M.B., Thomson, V.A., Wadley, J.J., Macqueen, P. et al	2016	2018	unpubl	2018	-	
included	included	*Gallus*	*gallus (Zhengyang Yellow)*	Phasianidae	KX987152		verified	verified	verified	no	Huang et al 2017b			2017	2017	Cladogram	-
included	excluded: no paper (yet)	*Gallus*	*gallus (Zhuxiang)*	Phasianidae	KX781318		verified	verified	verified	no	Liu, L., Yang, Y., Chen, F., Liu, Y., Yang, N., Xie, D. and Huang, Z.	2016	2016	unpubl	2016	-	
included	included	*Gallus*	*gallus bankiva*	Phasianidae	AP003323		verified	verified	verified	no	Nishibori et al 2005			2005	2005	Phylogram	(duplicate)
included	included	*Gallus*	*gallus jabouillei*	Phasianidae	GU261674		verified	verified	verified	no	Miao et al 2013			2013	2013	cladogram	(duplicate)
included	included	*Gallus*	*gallus jabouillei*	Phasianidae	GU261696		verified	verified	verified	no	Miao et al 2013			2013	2013	cladogram	(duplicate)
included	included	*Gallus*	*gallus murghi*	Phasianidae	GU261707		verified	verified	verified	no	Miao et al 2013			2013	2013	cladogram	(duplicate)
included	included	*Gallus*	*gallus murghi*	Phasianidae	GU261708		verified	verified	verified	no	Miao et al 2013			2013	2013	cladogram	(duplicate)
included	included	*Gallus*	*gallus murghi*	Phasianidae	GU261709		verified	verified	verified	no	Miao et al 2013			2013	2013	cladogram	(duplicate)
included	included	*Gallus*	*gallus spadiceus*	Phasianidae	GU261690		verified	verified	verified	no	Miao et al 2013			2013	2013	cladogram	(duplicate)
included	included	*Gallus*	*gallus spadiceus*	Phasianidae	GU261692		verified	verified	verified	no	Miao et al 2013			2013	2013	cladogram	(duplicate)
included	included	*Gallus*	*gallus spadiceus*	Phasianidae	GU261693		verified	verified	verified	no	Miao et al 2013			2013	2013	cladogram	(duplicate)
included	included	*Gallus*	*gallus spadiceus*	Phasianidae	GU261695		verified	verified	verified	no	Miao et al 2013			2013	2013	cladogram	(duplicate)
included	included	*Gallus*	*gallus spadiceus*	Phasianidae	GU261702		verified	verified	verified	no	Miao et al 2013			2013	2013	cladogram	(duplicate)
included	included	*Gallus*	*gallus spadiceus*	Phasianidae	GU261703		verified	verified	verified	no	Miao et al 2013			2013	2013	cladogram	(duplicate)
included	included	*Gallus*	*gallus spadiceus*	Phasianidae	GU261704		verified	verified	verified	no	Miao et al 2013			2013	2013	cladogram	(duplicate)
included	included	*Gallus*	*gallus spadiceus*	Phasianidae	GU261706		verified	verified	verified	no	Miao et al 2013			2013	2013	cladogram	(duplicate)
included	included	*Gallus*	*gallus spadiceus*	Phasianidae	GU261716	NC_040902	verified	verified	verified	no	Miao et al 2013			2013	2013	cladogram	(duplicate)
included	included	*Gallus*	*gallus spadiceus*	Phasianidae	AP003321		verified	verified	verified	no	Nishibori et al 2005			2005	2005	Phylogram	(duplicate)
included	included	*Gallus*	*lafayetti*	Phasianidae	AP003325	NC_007239	could not be verified: no ND2	could not be verified: no COI	verified	no	Nishibori et al 2005			2005	2005	Phylogram	(duplicate)
included	included	*Gallus*	*sonneratii*	Phasianidae	AP006741	NC_007240	could not be verified: too few ND2	could not be verified: no COI	could not be verified: no cyt b of pure bird	?	Nishibori et al 2005			2005	2005	Phylogram	(duplicate)
included	included	*Gallus*	*sonneratii*	Phasianidae	AP003320		dubious	dubious	could not be verified: no structure	?	Nishibori et al 2005			2005	2005	Phylogram	(duplicate)

Sequence database	Phylogeny database	Genus	species	family	GenBank#	RefSeq#	ND2 verified	COI verified	cyt b verified	erroneous sequence?	reference	submitted to GenBank	sequence released on GenBank	publication of paper	YEAR	Phylogeny (mitogenomic)	problematic sequences in tree
included	included	*Gallus*	*sonneratii*	Phasianidae	AP006746		could not be verified: no structure	could not be verified: no structure	could not be verified: no structure	?	Nishibori et al 2005			2005	2005	Phylogram	(duplicate)
included	included	*Gallus*	*varius*	Phasianidae	AP003324	NC_007238	dubious	could not be verified: no COI	could not be verified: too few cyt b	?	Nishibori et al 2005			2005	2005	Phylogram	(duplicate)
included	included	*Garrulax (Garrulax)*	*canorus*	Timaliidae	JQ348398		verified	verified	verified	no	Chen et al 2015			2015	2015	no	
included	included	*Garrulax (Garrulax)*	*canorus*	Timaliidae	KT633399	NC_020429	verified	verified	verified	no	Huang & Zeng 2016a			2016	2016	Phylogram	*Garrulax perspicillatus, Hirundo rustica*
included	included	*Garrulax (Ianthocincla)*	*cineraceus*	Timaliidae	KF926988	NC_024553	verified	verified	verified	no	Xue et al 2016			2016	2016	no	
included	included	*Garrulax (Ianthocincla)*	*ocellatus*	Timaliidae	KP995195	NC_027657	verified	verified	verified	no	Zhou et al 2016c			2016	2016	Phylogram	*Garrulax perspicillatus*
included	included	*Garrulax (Pterorhinus)*	*albogularis*	Timaliidae	KX082660	NC_037464	verified	verified	problematic	yes (chimera)	Liu et al 2018a			2018	2018	Phylogram	*Garrulax perspicillatus, Garrulax albogularis*
included	included	*Garrulax (Pterorhinus)*	*perspicillatus*	Timaliidae	KF997865	NC_026068	dubious	misidentified	misidentified	yes (misID)	Zhang et al 2016d			2016	2016	no	
included	included	*Garrulax (Pterorhinus)*	*poecilorhynchus*	Timaliidae	KR909134	NC_028082	misidentified	verified	verified	yes (chimera)	Qi et al 2016b			2016	2016	Phylogram	*Garrulax perspicillatus, Garrulax poecilorhynchus*
included	included	*Garrulax (Pterorhinus)*	*sannio*	Timaliidae	KT373847	NC_028186	verified	verified	verified	no	Wen et al 2017c			2017	2017	Phylogram	*Garrulax perspicillatus, Hirundo rustica*
included	included	*Garrulax (Pterorhinus)*	*sannio*	Timaliidae	KR869824		verified	verified	verified	no	Zhou et al 2016a			2016	2016	Phylogram	*Garrulax perspicillatus*
included	included	*Garrulax (Trochalopteron)*	*affinis (affine)*	Timaliidae	KT182082	NC_029402	verified	verified	verified	no	Huang et al 2016a			2016	2016	Phylogram	*Garrulax perspicillatus*
included	included	*Garrulax (Trochalopteron)*	*elliotti*	Timaliidae	KT272404	NC_034373	verified	verified	verified	no	Zhou et al 2016b			2016	2016	Phylogram	-
included	included	*Garrulax (Trochalopteron)*	*formosus*	Timaliidae	KR020504	NC_034353	verified	verified	verified	no	Huan et al 2016			2016	2016	Cladogram	*Garrulax perspicillatus*
included	included	*Garrulax (Trochalopteron)*	*milnei*	Timaliidae	MH238447	NC_041141	verified	verified	problematic	yes (chimera)	Zhang et al 2018a			2018	2018	Cladogram	*Garrulax perspicillatus, Garrulax milnei*
included	excluded: no paper (yet)	*Garrulus*	*glandarius*	Corvidae	JN018413	NC_015810	verified	verified	verified	no	Kan, X.-Z. and Chen, L.	2011	?	unpubl	2011	-	
included	included	*Gavia*	*arctica*	Gaviidae	MH064399	NC_041165	verified	verified	verified	no	Moon et al 2018			2018	2018	Phylogram	-
included	included	*Gavia*	*pacifica*	Gaviidae	MK342599		verified	verified	verified	no	Moon et al 2019			2019	2019	Phylogram	-
included	included	*Gavia*	*pacifica*	Gaviidae	AP009190	NC_008139	verified	verified	verified	no	Watanabe et al 2006			2006	2006	Phylogram	(duplicate)
included	included	*Gavia*	*stellata*	Gaviidae	AY293618	NC_007007	verified	verified	verified	no	Slack et al 2006			2006	2006	Cladogram	-
included	included	*Gelochelidon*	*nilotica*	Laridae	MF582631	NC_036344	verified	verified	verified	no	Yang et al 2017a			2017	2017	Cladogram	*Charadrius placidus, Larus vegae*
included	included	*Geobiastes*	*squamiger*	Brachypteraciidae	MK060131		could not be verified: no ND2	could not be verified: no COI	could not be verified: no cyt b	?	Tamashiro et al 2019			2019	2019	Phylogram	(duplicate)

Sequence database	Phylogeny database	Genus	species	family	GenBank#	RefSeq#	ND2 verified	COI verified	cyt b verified	erroneous sequence?	reference	submitted to GenBank	sequence released on GenBank	publication of paper	YEAR	Phylogeny (mitogenomic)	problematic sequences in tree
included	included	*Geococcyx*	*californianus*	Cuculidae	HM640212		verified	verified	could not be verified: cyt b could not be aligned	no	Pacheco et al 2011			2011	2011	Phylogram	(duplicate)
included	included	*Geococcyx*	*californianus*	Cuculidae	EU410488	NC_011711	verified	verified	could not be verified: cyt b could not be aligned	no	Pratt et al 2009			2009	2009	Phylogram	(duplicate)
included	included	*Geokichla*	*sibirica*	Turdidae	MK377247		verified	verified	verified	no	Sun et al 2019			2019	2019	no	
included	included	*Geopelia*	*striata*	Columbidae	MG590276		verified	verified	verified	no	Bruxaux et al 2018			2018	2018	Phylogram	(duplicate)
included	included	*Geopelia*	*striata*	Columbidae	HM746791		verified	verified	verified	no	Pacheco et al 2011			2011	2011	Phylogram	(duplicate)
included	included	*Geospiza*	*fortis*	Thraupidae	KM891730		verified	could not be verified: no COI	could not be verified: no structure	no	Lamichhaney et al 2015			2015	2015	Phylogram	-
included	included	*Geospiza*	*magnirostris*	Thraupidae	MG682351	NC_039770	could not be verified: no ND2	could not be verified: no COI	could not be verified: no structure	?	Wu et al 2019a			2019	2019	Cladogram	-
included	included	*Geotrygon*	*violacea*	Columbidae	HM640213	NC_015207	verified	could not be verified: no COI	verified	no	Pacheco et al 2011			2011	2011	Phylogram	(duplicate)
included	included	*Gerygone*	*igata*	Acanthizidae	KC545399	NC_029139	verified	verified	could not be verified: no cyt b	no	Gibb et al 2015			2015	2015	Phylogram	(duplicate)
included	included	*Glaucidium*	*brodiei*	Strigidae	MF155890		misidentified	misidentified	could not be verified: no cyt b	yes (misID)	Liu et al 2019a			2019	2019	Cladogram	*Glaucidium brodiei, Otus scops, Accipiter gularis, Tyto longimembris, Bubo bubo, Caprimulgus jotaka, Phaethornis malaris, Ceryle rudis, Falco naumanni, Strix leptogrammica, Ninox strenua*
included	included	*Glaucidium*	*brodiei*	Strigidae	KP684122		verified	could not be verified: no COI	could not be verified: no cyt b	no	Sun et al 2016e			2016	2016	no	
included	included	*Glaucidium*	*cuculoides*	Strigidae	KY092431	NC_034296	verified	verified	verified	no	Liu et al 2019a			2019	2019	Cladogram	(duplicate)
included	excluded: no paper (yet)	*Glaucis*	*hirsutus*	Trochilidae	KT265275	NC_033413	verified	verified	could not be verified: no cyt b	no	Ruschi, P., Jennings, B. and Prosdocimi, F.	2015	2016	unpubl	2016	-	
included	included	*Gnorimopsar*	*chopi*	Icteridae	JX516055	NC_018795	verified	verified	verified	no	Powell et al 2013			2013	2013	Cladogram	(duplicate)
included	included	*Gorsachius*	*goisagi*	Ardeidae	KT364530	NC_028194	could not be verified: no ND2	verified	could not be verified: no cyt b	no	Zhou et al 2016e			2016	2016	Phylogram	-
included	included	*Gorsachius*	*magnificus*	Ardeidae	KT364529	NC_028193	could not be verified: no ND2	could not be verified: no COI	could not be verified: no cyt b	?	Zhou et al 2016e			2016	2016	Phylogram	(duplicate)

Sequence database	Phylogeny database	Genus	species	family	GenBank#	RefSeq#	ND2 verified	COI verified	cyt b verified	erroneous sequence?	reference	submitted to GenBank	sequence released on GenBank	publication of paper	YEAR	Phylogeny (mitogenomic)	problematic sequences in tree
included	included	*Gorsachius*	*melanolophus*	Ardeidae	KT364531	NC_028195	could not be verified: no ND2	verified	could not be verified: no cyt b	no	Zhou et al 2016e			2016	2016	Phylogram	(duplicate)
included	included	*Goura*	*cristata*	Columbidae	LN589994		verified	could not be verified: no COI	verified	no	Besnard et al 2016			2016	2016	no	
included	included	*Goura*	*cristata*	Columbidae	MG590267		verified	could not be verified: no COI	verified	no	Bruxaux et al 2018			2018	2018	Phylogram	(duplicate)
included	included	*Goura*	*cristata*	Columbidae	MG590268		verified	could not be verified: no COI	verified	no	Bruxaux et al 2018			2018	2018	Phylogram	(duplicate)
included	included	*Goura*	*cristata*	Columbidae	MG590269		verified	could not be verified: no COI	verified	no	Bruxaux et al 2018			2018	2018	Phylogram	(duplicate)
included	included	*Goura*	*cristata*	Columbidae	MG590270		verified	could not be verified: no COI	verified	no	Bruxaux et al 2018			2018	2018	Phylogram	(duplicate)
included	included	*Goura*	*cristata*	Columbidae	MG590271		verified	could not be verified: no COI	verified	no	Bruxaux et al 2018			2018	2018	Phylogram	(duplicate)
included	included	*Goura*	*cristata*	Columbidae	MG590272		verified	could not be verified: no COI	verified	no	Bruxaux et al 2018			2018	2018	Phylogram	(duplicate)
included	included	*Goura*	*cristata*	Columbidae	MG590273		verified	could not be verified: no COI	verified	no	Bruxaux et al 2018			2018	2018	Phylogram	(duplicate)
included	included	*Goura*	*cristata*	Columbidae	MG590274		verified	could not be verified: no COI	verified	no	Bruxaux et al 2018			2018	2018	Phylogram	(duplicate)
included	included	*Goura*	*cristata*	Columbidae	MG590275		verified	could not be verified: no COI	verified	no	Bruxaux et al 2018			2018	2018	Phylogram	(duplicate)
included	included	*Goura*	*cristata*	Columbidae	KX902242	NC_031865	verified	could not be verified: no COI	verified	no	Soares et al 2016			2016	2016	Cladogram	(duplicate)
included	included	*Goura*	*scheepmakeri*	Columbidae	LN589995	NC_027947	could not be verified: no ND2	could not be verified: no COI	could not be verified: no cyt b	?	Besnard et al 2016			2016	2016	no	
included	included	*Goura*	*scheepmakeri*	Columbidae	MG590280		could not be verified: no ND2	could not be verified: no COI	could not be verified: no cyt b	?	Bruxaux et al 2018			2018	2018	Phylogram	(duplicate)
included	included	*Goura*	*scheepmakeri*	Columbidae	MG590281		could not be verified: no ND2	could not be verified: no COI	could not be verified: no cyt b	?	Bruxaux et al 2018			2018	2018	Phylogram	(duplicate)
included	included	*Goura*	*scheepmakeri*	Columbidae	MG590282		could not be verified: no ND2	could not be verified: no COI	could not be verified: no cyt b	?	Bruxaux et al 2018			2018	2018	Phylogram	(duplicate)
included	included	*Goura*	*scheepmakeri*	Columbidae	MG590283		could not be verified: no ND2	could not be verified: no COI	could not be verified: no cyt b	?	Bruxaux et al 2018			2018	2018	Phylogram	(duplicate)
included	included	*Goura*	*scheepmakeri*	Columbidae	MG590284		could not be verified: no ND2	could not be verified: no COI	could not be verified: no cyt b	?	Bruxaux et al 2018			2018	2018	Phylogram	(duplicate)

Sequence database	Phylogeny database	Genus	species	family	GenBank#	RefSeq#	ND2 verified	COI verified	cyt b verified	erroneous sequence?	reference	submitted to GenBank	sequence released on GenBank	publication of paper	YEAR	Phylogeny (mitogenomic)	problematic sequences in tree
included	included	*Goura*	*sclaterii*	Columbidae	MG590277		could not be verified: no ND2	could not be verified: no COI	could not be verified: no cyt b	?	Bruxaux et al 2018			2018	2018	Phylogram	(duplicate)
included	included	*Goura*	*sclaterii*	Columbidae	MG590278		could not be verified: no ND2	could not be verified: no COI	could not be verified: no cyt b	?	Bruxaux et al 2018			2018	2018	Phylogram	(duplicate)
included	included	*Goura*	*sclaterii*	Columbidae	MG590279		could not be verified: no ND2	could not be verified: no COI	could not be verified: no cyt b	?	Bruxaux et al 2018			2018	2018	Phylogram	(duplicate)
included	included	*Goura*	*sclaterii*	Columbidae	MG590285		could not be verified: no ND2	could not be verified: no COI	could not be verified: no cyt b	?	Bruxaux et al 2018			2018	2018	Phylogram	(duplicate)
included	included	*Goura*	*sclaterii*	Columbidae	MG590286		could not be verified: no ND2	could not be verified: no COI	could not be verified: no cyt b	?	Bruxaux et al 2018			2018	2018	Phylogram	(duplicate)
included	included	*Goura*	*sclaterii*	Columbidae	MG590287		could not be verified: no ND2	could not be verified: no COI	could not be verified: no cyt b	?	Bruxaux et al 2018			2018	2018	Phylogram	(duplicate)
included	included	*Goura*	*sclaterii*	Columbidae	MG590288		could not be verified: no ND2	could not be verified: no COI	could not be verified: no cyt b	?	Bruxaux et al 2018			2018	2018	Phylogram	(duplicate)
included	included	*Goura*	*victoria*	Columbidae	LN589993		verified	could not be verified: no COI	verified	no	Besnard et al 2016			2016	2016	no	
included	included	*Goura*	*victoria*	Columbidae	MG590289		verified	could not be verified: no COI	verified	no	Bruxaux et al 2018			2018	2018	Phylogram	(duplicate)
included	included	*Goura*	*victoria*	Columbidae	MG590290		verified	could not be verified: no COI	verified	no	Bruxaux et al 2018			2018	2018	Phylogram	(duplicate)
included	included	*Goura*	*victoria*	Columbidae	MG590291		verified	could not be verified: no COI	verified	no	Bruxaux et al 2018			2018	2018	Phylogram	(duplicate)
included	included	*Goura*	*victoria*	Columbidae	MG590292		verified	could not be verified: no COI	verified	no	Bruxaux et al 2018			2018	2018	Phylogram	(duplicate)
included	included	*Goura*	*victoria*	Columbidae	MG590293		verified	could not be verified: no COI	verified	no	Bruxaux et al 2018			2018	2018	Phylogram	(duplicate)
included	included	*Goura*	*victoria*	Columbidae	MG590294		verified	could not be verified: no COI	verified	no	Bruxaux et al 2018			2018	2018	Phylogram	(duplicate)
included	included	*Goura*	*victoria*	Columbidae	MG590295		verified	could not be verified: no COI	verified	no	Bruxaux et al 2018			2018	2018	Phylogram	(duplicate)
included	included	*Goura*	*victoria*	Columbidae	MG590296		verified	could not be verified: no COI	verified	no	Bruxaux et al 2018			2018	2018	Phylogram	(duplicate)
included	included	*Goura*	*victoria*	Columbidae	MG590297		verified	could not be verified: no COI	verified	no	Bruxaux et al 2018			2018	2018	Phylogram	(duplicate)
included	included	*Goura*	*victoria*	Columbidae	MG590298		verified	could not be verified: no COI	verified	no	Bruxaux et al 2018			2018	2018	Phylogram	(duplicate)

Sequence database	Phylogeny database	Genus	species	family	GenBank#	RefSeq#	ND2 verified	COI verified	cyt b verified	erroneous sequence?	reference	submitted to GenBank	sequence released on GenBank	publication of paper	YEAR	Phylogeny (mitogenomic)	problematic sequences in tree
included	included	*Goura*	*victoria*	Columbidae	MG590299		verified	could not be verified: no COI	verified	no	Bruxaux et al 2018			2018	2018	Phylogram	(duplicate)
included	included	*Goura*	*victoria*	Columbidae	MG590300		verified	could not be verified: no COI	verified	no	Bruxaux et al 2018			2018	2018	Phylogram	(duplicate)
included	included	*Goura*	*victoria*	Columbidae	MG590301		verified	could not be verified: no COI	verified	no	Bruxaux et al 2018			2018	2018	Phylogram	(duplicate)
included	included	*Goura*	*victoria*	Columbidae	MG590302		verified	could not be verified: no COI	verified	no	Bruxaux et al 2018			2018	2018	Phylogram	(duplicate)
included	included	*Goura*	*victoria*	Columbidae	MG590303	NC_036613	verified	could not be verified: no COI	verified	no	Bruxaux et al 2018			2018	2018	Phylogram	(duplicate)
included	excluded: no paper (yet)	*Gracula*	*religiosa*	Sturnidae	JF937590	NC_015898	verified	could not be verified: no COI	verified	no	Kan, X.-Z. and Qian, C.-J.	2011	?	unpubl	2011	-	
included	included	*Grus*	*americana*	Gruidae	FJ769848	NC_020576	could not be verified: no ND2	verified	verified	no	Krajewski et al 2010			2010	2010	Phylogram	(duplicate)
included	included	*Grus*	*antigone*	Gruidae	FJ769854	NC_020581	could not be verified: no ND2	verified	verified	no	Krajewski et al 2010			2010	2010	Phylogram	(duplicate)
included	included	*Grus*	*canadensis*	Gruidae	FJ769855	NC_020582	verified	verified	verified	no	Krajewski et al 2010			2010	2010	Phylogram	(duplicate)
included	included	*Grus*	*grus*	Gruidae	FJ769849	NC_020577	could not be verified: no ND2	verified	verified	no	Krajewski et al 2010			2010	2010	Phylogram	(duplicate)
included	excluded: no paper (yet)	*Grus*	*japonensis*	Gruidae	JN380204		could not be verified: no ND2	verified	verified	no	Bi, X., Huang, L. and Jing, M.	2011	?	unpubl	2011	-	
included	included	*Grus*	*japonensis*	Gruidae	MH041485		could not be verified: no ND2	verified	verified	no	Hua et al 2018a			2018	2018	Phylogram	-
included	included	*Grus*	*japonensis*	Gruidae	FJ769847	NC_020575	could not be verified: no ND2	verified	verified	no	Krajewski et al 2010			2010	2010	Phylogram	(duplicate)
included	included	*Grus*	*leucogeranus*	Gruidae	FJ769846	NC_020574	could not be verified: no ND2	verified	verified	no	Krajewski et al 2010			2010	2010	Phylogram	(duplicate)
included	included	*Grus*	*leucogeranus*	Gruidae	MH041490		could not be verified: no ND2	misidentified	misidentified	yes (misID)	Wang et al 2018			2018	2018	Phylogram	*Grus leucogeranus*
included	included	*Grus*	*monachus*	Gruidae	FJ769850	NC_020578	could not be verified: no ND2	verified	verified	no	Krajewski et al 2010			2010	2010	Phylogram	(duplicate)
included	included	*Grus*	*nigricollis*	Gruidae	FJ769851	NC_020579	could not be verified: no ND2	verified	verified	no	Krajewski et al 2010			2010	2010	Phylogram	(duplicate)
included	included	*Grus*	*paradisea*	Gruidae	FJ769844	NC_020572	verified	verified	verified	no	Krajewski et al 2010			2010	2010	Phylogram	(duplicate)

Sequence database	Phylogeny database	Genus	species	family	GenBank#	RefSeq#	ND2 verified	COI verified	cyt b verified	erroneous sequence?	reference	submitted to GenBank	sequence released on GenBank	publication of paper	YEAR	Phylogeny (mitogenomic)	problematic sequences in tree
included	included	*Grus*	*rubicunda*	Gruidae	FJ769853	NC_020580	could not be verified: no ND2	could not be verified: no COI	verified	no	Krajewski et al 2010			2010	2010	Phylogram	(duplicate)
included	included	*Grus*	*vipio*	Gruidae	FJ769852	NC_021368	could not be verified: no ND2	verified	verified	no	Krajewski et al 2010			2010	2010	Phylogram	(duplicate)
included	included	*Grus*	*virgo*	Gruidae	FJ769845	NC_020573	could not be verified: no ND2	verified	verified	no	Krajewski et al 2010			2010	2010	Phylogram	(duplicate)
included	included	*Grus (Bugeranus)*	*carunculatus*	Gruidae	MH041486		could not be verified: no ND2	verified	verified	no	Hua et al 2018b			2018	2018	Phylogram	-
included	included	*Grus (Bugeranus)*	*carunculatus*	Gruidae	FJ769843	NC_020571	could not be verified: no ND2	verified	verified	no	Krajewski et al 2010			2010	2010	Phylogram	(duplicate)
included	included	*Guaruba*	*guarouba*	Psittacidae	JQ782217	NC_026031	verified	verified	verified	no	Urantówka et al 2017e			2017	2017	no	
included	included	*Gymnomystax*	*mexicanus*	Icteridae	JX516075	NC_018812	verified	could not be verified: no COI	verified	no	Powell et al 2013			2013	2013	Cladogram	(duplicate)
included	included	*Gymnorhina (Cracticus)*	*tibicen*	Artamidae	MK453379		verified	verified	verified	no	Grealy et al 2019			2019	2019	Phylogram	(duplicate)
included	included	*Gyps*	*fulvus*	Accipitridae	KX893247	NC_036050	verified	verified	verified	no	Mereu et al 2017			2017	2017	Cladogram	*Branta bernicla, Passer ammodendri*
included	included	*Gyps*	*himalayensis*	Accipitridae	KY594709	NC_039095	verified	verified	verified	no	Jiang et al 2019b	2017	2019	2019	2019	Phylogram	*Strix leptogrammica, Falco naumanni*
included	included	*Haematopus*	*ater*	Haematopodidae	AY074886	NC_003713	could not be verified: no ND2	verified	could not be verified: no cyt b	no	Paton et al 2002			2002	2002	Phylogram	(duplicate)
included	included	*Haematopus*	*ostralegus*	Haematopodidae	KY419886	NC_034237	verified	could not be verified: no structure	verified	no	Lee et al 2017c			2017	2017	Phylogram	*Charadrius placidus, Vanellus cinereus*
included	included	*Haematortyx*	*sanguiniceps*	Phasianidae	KY411591	NC_034001	verified	could not be verified: no COI	verified	no	Wang et al 2017a			2017	2017	Phylogram	(duplicate)
included	included	*Haemorhous*	*cassinii*	Fringillidae	KM078786	NC_025613	verified	verified	verified	no	Lerner et al 2011			2011	2011	Cladogram	(duplicate)
included	excluded: no paper (yet)	*Haemorhous*	*mexicanus*	Fringillidae	FJ236300		misidentified	misidentified	misidentified	yes (misID)	Carson, R.J. and Spicer, G.S.	2008	?	unpubl	2008	-	
included	included	*Haemorhous*	*mexicanus*	Fringillidae	KM078782	NC_025610	verified	verified	verified	no	Lerner et al 2011			2011	2011	Cladogram	(duplicate)
included	excluded: no paper (yet)	*Halcyon*	*coromanda*	Alcedinidae	KT356219	NC_028177	verified	verified	verified	no	Park, C.E., Park, G.S., Jung, B.K., Park, Y.J., Kim, M.C., Park, H.C., Shin, J.H.	2015	2015	unpubl	2015	-	
included	included	*Halcyon*	*pileata*	Alcedinidae	KJ476742	NC_024198	verified	could not be verified: no COI	could not be verified: no cyt b	no	Sun et al 2017c			2017	2017	Phylogram	(duplicate)
included	included	*Halcyon*	*sancta vagans*	Alcedinidae	EU410489	NC_011712	verified	verified	could not be verified: no cyt b	no	Pratt et al 2009			2009	2009	Phylogram	(duplicate)

Sequence database	Phylogeny database	Genus	species	family	GenBank#	RefSeq#	ND2 verified	COI verified	cyt b verified	erroneous sequence?	reference	submitted to GenBank	sequence released on GenBank	publication of paper	YEAR	Phylogeny (mitogenomic)	problematic sequences in tree
included	included	*Halcyon*	*smyrnensis*	Alcedinidae	KT965614		verified	verified	verified	no	Qu et al 2016			2016	2016	no	
included	excluded: no paper (yet)	*Halcyon*	*smyrnensis*	Alcedinidae	KY940559	NC_035746	verified	verified	verified	no	Wang, Y.-C., Zou, Y., Jing, M.-D., Huang, L., Xu, J., Bi, X.-X.	2017	2017	unpubl	2017	-	
included	included	*Haliaeetus*	*albicilla*	Accipitridae	MK043028	NC_040858	verified	verified	verified	no	Kim et al 2019a			2019	2019	Phylogram	*Accipiter gularis*
included	excluded: no paper (yet)	*Heliodoxa (Polyplancta)*	*aurescens*	Trochilidae	KP853094	NC_030285	verified	verified	could not be verified: no cyt b	no	Costa, I.R., Souto, H.M., Furtado, C., Mudge, J., McGuire, J., Witt, C., Jennings, B., Ruschi, P. and Prosdocimi, F.	2015	2016	unpubl	2016	-	
included	included	*Heliornis*	*fulica*	Rallidae	KF644581	NC_025499	could not be verified: no ND2	verified	verified	no	Garcia et al 2014			2014	2014	Phylogram	(duplicate)
included	included	*Hemignathus*	*flavus*	Fringillidae	KM078780	NC_025608	could not be verified: no ND2	could not be verified: no structure	could not be verified: no cyt b	?	Lerner et al 2011			2011	2011	Cladogram	(duplicate)
included	included	*Hemignathus*	*munroi*	Fringillidae	KM078774		could not be verified: no ND2	could not be verified: no structure	verified	no	Lerner et al 2011			2011	2011	Cladogram	(duplicate)
included	included	*Hemignathus*	*parvus*	Fringillidae	KM078799	NC_025622	could not be verified: no ND2	could not be verified: no structure	verified	no	Lerner et al 2011			2011	2011	Cladogram	(duplicate)
included	included	*Hemignathus*	*stejnegeri*	Fringillidae	KM078801	NC_025624	could not be verified: no ND2	could not be verified: no structure	could not be verified: no cyt b	?	Lerner et al 2011			2011	2011	Cladogram	(duplicate)
included	included	*Hemignathus*	*virens virens*	Fringillidae	KM078788		verified	could not be verified: no structure	verified	no	Lerner et al 2011			2011	2011	Cladogram	(duplicate)
included	included	*Hemignathus*	*virens wilsoni*	Fringillidae	KM078802		verified	could not be verified: failed alignment	could not be verified: no cyt b	no	Lerner et al 2011			2011	2011	Cladogram	(duplicate)
included	included	*Hemiphaga*	*novaeseelandiae*	Columbidae	EU725864		verified	verified	verified	no	Gibb & Penny 2010			2010	2010	Phylogram	-
included	included	*Henicorhina*	*leucosticta*	Troglodytidae	KJ719074		verified	verified	verified	no	Aguilar et al 2014			2014	2014	no	
included	included	*Henicorhina*	*leucosticta*	Troglodytidae	KJ746107	NC_024673	verified	verified	verified	no	Aguilar et al 2014			2014	2014	no	(duplicate)
included	included	*Hesperiphona*	*vespertina*	Fringillidae	KM078770	NC_025600	verified	verified	verified	no	Lerner et al 2011			2011	2011	Cladogram	(duplicate)
included	included	*Heteralocha*	*acutirostris*	Callaeatidae	KU158193	NC_031351	verified	could not be verified: no COI	could not be verified: no cyt b	no	Anmarkrud & Lifjeld 2017			2017	2017	no	
included	included	*Heterophasia*	*melanoleuca*	Timaliidae	MK408609		verified	verified	verified	no	Wang et al 2019b		2019	2019	2019	Phylogram	-
included	included	*Himantopus*	*himantopus*	Recurvirostridae	KY623656	NC_035423	could not be verified: no ND2	verified	verified	no	Yang et al 2017b			2017	2017	Cladogram	*Larus vegae, Vanellus cinereus*
included	included	*Himatione*	*sanguinea*	Fringillidae	KM078773	NC_025602	could not be verified: no ND2	could not be verified: no structure	verified	no	Lerner et al 2011			2011	2011	Cladogram	(duplicate)

Sequence database	Phylogeny database	Genus	species	family	GenBank#	RefSeq#	ND2 verified	COI verified	cyt b verified	erroneous sequence?	reference	submitted to GenBank	sequence released on GenBank	publication of paper	YEAR	Phylogeny (mitogenomic)	problematic sequences in tree
included	excluded: no paper (yet)	*Hirundo*	*rustica erythrogaster*	Hirundinidae	KX398931		verified	verified	verified	no	Keepers, K.G., Scordato, E.S.C., Jenkins, B., Safran, R.J., Kane, N.C.	2016	2016	unpubl	2016	-	
included	excluded: no paper (yet)	*Hirundo*	*rustica gutturalis*	Hirundinidae	KP148840		problematic	dubious	problematic	yes (seq errors/numts)	Liu, J., Liu, S., Shao, C., Zhang, Y., Xie, Y., Tang, Q., Shen, Y., Xie, J.	2014	?	unpubl	2014	-	
included	included	*Horonis (Cettia)*	*fortipes*	Scotocercidae	MK051002		verified	verified	verified	no	Jiang et al 2019a		2020	2019	2019	Cladogram	*Garrulax albogularis, Garrulax perspicillatus*
included	included	*Hydrobates (Oceanodroma)*	*castro*	Hydrobatidae	MK170187		could not be verified: no ND2	misidentified	misidentified	yes (misID)	Antaky et al 2019			2019	2019	Phylogram	*Hydrobates castro*
included	included	*Hydrobates (Oceanodroma)*	*castro*	Hydrobatidae	MH433599	NC_041251	could not be verified: no ND2	problematic	misidentified	yes (taxonomy)	Jiang et al 2018			2018	2018	network	*Larus vegae, Hydrobates castro*
included	excluded: paper publ. after 1-1-2020	*Hydrophasianus*	*chirurgus*	Jacanidae	MH219929	NC_041576	verified	verified	verified	no	Chen et al 2020c	2018	2019	2020	2019	Phylogram	(duplicate)
included	included	*Hyliota*	*flavigaster*	Sylviidae	KJ909191	NC_024868	verified	could not be verified: no COI	could not be verified: no cyt b	no	Barker 2014			2014	2014	Cladogram	(duplicate)
included	excluded: no paper (yet)	*Hylocharis*	*cyanus*	Trochilidae	KJ619586	NC_027453	verified	verified	could not be verified: no cyt b	no	Magarinos Souto, H., Angeli Ruschi, P., Furtado, C., Bryan Jennings, W. and Prosdocimi, F.	2014	?	unpubl	2014	-	
included	excluded: no paper (yet)	*Icterus*	*bullockii*	Icteridae	FJ236287		verified	verified	verified	no	Carson, R.J. and Spicer, G.S.	2008	?	unpubl	2008	-	
included	included	*Icterus*	*mesomelas*	Icteridae	JX516068		verified	verified	verified	no	Powell et al 2013			2013	2013	Cladogram	(duplicate)
included	included	*Indicator*	*maculatus*	Indicatoridae	MK060132	NC_039889	verified	verified	could not be verified: no cyt b	no	Tamashiro et al 2019			2019	2019	Phylogram	(duplicate)
included	included	*Indicator*	*xanthonotus*	Indicatoridae	MH737741	NC_040005	could not be verified: no ND2	could not be verified: no COI	could not be verified: no cyt b	?	Duan et al 2018b			2018	2018	Phylogram	*Alcedo atthis, Ceryle rudis, Megaceryle lugubris, Trogon viridis*
included	included	*Ithaginis*	*cruentus*	Phasianidae	JQ713766		verified	verified	verified	no	Shen et al 2014			2014	2014	Phylogram	(duplicate)
included	included	*Ithaginis*	*cruentus*	Phasianidae	KY411592		verified	verified	verified	no	Wang et al 2017a			2017	2017	Phylogram	(duplicate)
included	included	*Ithaginis*	*cruentus*	Phasianidae	JF921875	NC_018033	verified	verified	verified	no	Zeng et al 2013			2013	2013	Phylogram	*Phasianus versicolor, Francolinus pintadeanus*
included	included	*Ixobrychus*	*cinnamomeus*	Ardeidae	HQ690247	NC_015077	could not be verified: no ND2	verified	verified	no	Zhang et al 2012			2012	2012	Phylogram	-

Sequence database	Phylogeny database	Genus	species	family	GenBank#	RefSeq#	ND2 verified	COI verified	cyt b verified	erroneous sequence?	reference	submitted to GenBank	sequence released on GenBank	publication of paper	YEAR	Phylogeny (mitogenomic)	problematic sequences in tree
included	included	*Ixobrychus*	*cinnamomeus*	Ardeidae	KJ190959		could not be verified: no ND2	verified	verified	no	Zhou et al 2014b			2014	2014	Phylogram	(duplicate)
included	included	*Ixobrychus*	*eurhythmus*	Ardeidae	KJ190956	NC_025924	could not be verified: no ND2	verified	verified	no	Zhou et al 2014b			2014	2014	Phylogram	(duplicate)
included	included	*Ixobrychus*	*sinensis*	Ardeidae	KJ190958	NC_025925	could not be verified: no ND2	verified	verified	no	Zhou et al 2014b			2014	2014	Phylogram	(duplicate)
included	included	*Ixobrychus (Dupetor)*	*flavicollis*	Ardeidae	KJ643141	NC_024575	could not be verified: no ND2	verified	verified	no	Wang et al 2016d			2016	2016	no	
included	included	*Ixobrychus (Dupetor)*	*flavicollis*	Ardeidae	KJ190957		could not be verified: no ND2	verified	verified	no	Zhou et al 2014b			2014	2014	Phylogram	(duplicate)
included	excluded: no paper (yet)	*Ixos*	*mcclellandii*	Pycnonotidae	KX640824	NC_039396	verified	dubious	verified	?	Chen, Y., Song, X., Du, C., Zhou, C., Lu, T., Zhang, X. and Yue, B.	2016	2018	unpubl	2018	-	
included	included	*Jacana*	*jacana*	Jacanidae	KJ631049	NC_024069	verified	verified	verified	no	Miller et al 2016			2016	2016	no	
included	included	*Jacana*	*spinosa*	Jacanidae	KJ631048	NC_024068	could not be verified: no ND2	verified	could not be verified: reference seq may be misid'd	no	Miller et al 2016			2016	2016	no	
included	excluded: no paper (yet)	*Junco*	*hyemalis*	Passerellidae	FJ236293		could not be verified: no structure	could not be verified: no structure	verified	no	Carson, R.J. and Spicer, G.S.	2008	?	unpubl	2008	-	
included	included	*Jynx*	*ruficollis*	Picidae	MK060133	NC_039890	verified	could not be verified: no COI	verified	no	Tamashiro et al 2019			2019	2019	Phylogram	(duplicate)
included	included	*Lagopus*	*lagopus*	Phasianidae	KX609784	NC_035568	verified	verified	verified	no	Sveinsdottir & Magnusson 2017			2017	2017	Phylogram	-
included	included	*Lagopus*	*muta*	Phasianidae	KX609785		verified	verified	verified	no	Sveinsdottir & Magnusson 2017			2017	2017	Phylogram	(duplicate)
included	included	*Lagopus*	*muta*	Phasianidae	KY411593	NC_034002	verified	verified	verified	no	Wang et al 2017a			2017	2017	Phylogram	(duplicate)
included	included	*Lalage*	*tricolor*	Campephagidae	KY994597		could not be verified: no structure	could not be verified: no COI	could not be verified: no cyt b	?	Lamb et al 2018			2018	2018	Phylogram	(duplicate)
included	included	*Lampropsar*	*tanagrinus*	Icteridae	JX516057		verified	verified	verified	no	Powell et al 2013			2013	2013	Cladogram	(duplicate)
included	included	*Lanius*	*cristatus*	Laniidae	KT004451	NC_028333	verified	verified	verified	no	Liu et al 2016c			2016	2016	Cladogram	*Lanius tephronotus*
included	included	*Lanius*	*isabellinus*	Laniidae	KP995437	NC_027655	could not be verified: no ND2	could not be verified: no structure	verified	no	Liu et al 2016d			2016	2016	Cladogram	*Lanius tephronotus*
included	included	*Lanius*	*schach*	Laniidae	KU058639	NC_030604	verified	verified	verified	no	Yang et al 2016a			2016	2016	Phylogram	*Lanius tephronotus*
included	included	*Lanius*	*sphenocercus*	Laniidae	KU884610		could not be verified: no ND2	could not be verified: no COI	verified	no	Yang et al 2016b			2016	2016	Phylogram	*Lanius tephronotus*
included	included	*Lanius*	*tephronotus*	Laniidae	JX486029	NC_021105	misidentified	misidentified	misidentified	yes (misID)	Qian et al 2013b			2013	2013	no	
included	excluded: no paper (yet)	*Lanius*	*tigrinus*	Laniidae	LC428205	NC_039818	verified	verified	verified	no	Yamamoto, Y., Kakizawa, R. and Yamagishi, S.	2018	2018	unpubl	2018	-	

Sequence database	Phylogeny database	Genus	species	family	GenBank#	RefSeq#	ND2 verified	COI verified	cyt b verified	erroneous sequence?	reference	submitted to GenBank	sequence released on GenBank	publication of paper	YEAR	Phylogeny (mitogenomic)	problematic sequences in tree
included	included	*Larus*	*crassirostris*	Laridae	KM507782	NC_025556	could not be verified: no ND2	verified	verified	no	Kim & Park 2016	2014		2016	2016	no	
included	included	*Larus*	*dominicanus*	Laridae	AY293619	NC_007006	verified	could not be verified: no structure	verified	no	Slack et al 2007			2007	2007	network	(duplicate)
included	included	*Larus*	*relictus*	Laridae	KC760146	NC_023777	verified	could not be verified: no COI	verified	no	Yang et al 2016c			2016	2016	no	
included	excluded: no paper (yet)	*Larus*	*vegae*	Laridae	KT943749		misidentified	misidentified	misidentified	yes (misID)	Park, C.E., Hong, S.J., Park, G.S., Kim, M.C., Park, H.C. and Shin, J.H.	2015	2016	unpubl	2016	-	
included	excluded: no paper (yet)	*Leiothrix*	*argentauris*	Timaliidae	HQ690245	NC_015114	verified	verified	verified	no	Kan, X.-Z. and Chen, L.	2010	?	unpubl	2010	-	
included	excluded: no paper (yet)	*Leiothrix*	*lutea*	Timaliidae	JQ423933	NC_020427	verified	verified	verified	no	Chen, L. and Kan, X.-Z.	2012	?	unpubl	2012	-	
included	included	*Lepidocolaptes*	*angustirostris*	Furnariidae	KY628989	NC_037154	verified	verified	verified	no	Caparroz et al 2018			2018	2018	Cladogram	*Garrulax perspicillatus, Cyanoptila cyanomelana, Thamnophilus nigrocinereus*
included	included	*Lepidothrix*	*coronata*	Pipridae	KJ909196		verified	verified	verified	no	Barker 2014			2014	2014	Cladogram	(duplicate)
included	included	*Leptotila*	*verreauxi*	Columbidae	HM640214	NC_015190	verified	verified	verified	no	Pacheco et al 2011			2011	2011	Phylogram	(duplicate)
included	included	*Lerwa*	*lerwa*	Phasianidae	KY411594		could not be verified: no ND2	could not be verified: no COI	verified	no	Wang et al 2017a			2017	2017	Phylogram	(duplicate)
included	included	*Leucosticte*	*arctoa*	Fringillidae	KM078791	NC_025615	misidentified	misidentified	could not be verified: no structure	yes (taxonomy)	Lerner et al 2011			2011	2011	Cladogram	(duplicate)
included	included	*Leucosticte*	*brandti*	Fringillidae	KM078775	NC_025604	verified	verified	verified	no	Lerner et al 2011			2011	2011	Cladogram	(duplicate)
included	included	*Lewinia*	*muelleri*	Rallidae	KF644584	NC_025502	could not be verified: no ND2	verified	verified	no	Garcia et al 2014			2014	2014	Phylogram	(duplicate)
included	excluded: no paper (yet)	*Limosa*	*lapponica baueri*	Scolopacidae	KX371106		verified	verified	verified	no	Lima, N.C.B., Parody-Merino, A.M., Battley, P.F., Fidler, A.E., Prosdocimi, F.	2016	2017	unpubl	2017	-	
included	included	*Liocichla*	*omeiensis*	Timaliidae	KU886092		verified	verified	verified	no	Zhao et al 2019			2019	2019	Phylogram	*Garrulax perspicillatus, Garrulax poecilorhynchus*
included	included	*Locustella*	*pryeri*	Locustellidae	KJ001760	NC_029151	verified	verified	verified	no	Master et al 2016			2016	2016	no	
included	included	*Lonchura*	*caniceps*	Estrildidae	MF770312	NC_036397	could not be verified: no structure	could not be verified: no structure	could not be verified: no structure	?	Stryjewski & Sorenson 2017			2017	2017	Cladogram	-

Sequence database	Phylogeny database	Genus	species	family	GenBank#	RefSeq#	ND2 verified	COI verified	cyt b verified	erroneous sequence?	reference	submitted to GenBank	sequence released on GenBank	publication of paper	YEAR	Phylogeny (mitogenomic)	problematic sequences in tree
included	included	*Lonchura*	*caniceps*	Estrildidae	MF770313		could not be verified: no structure	could not be verified: no structure	could not be verified: no structure	?	Stryjewski & Sorenson 2017			2017	2017	Cladogram	(duplicate)
included	included	*Lonchura*	*caniceps*	Estrildidae	MF770314		could not be verified: no structure	could not be verified: no structure	could not be verified: no structure	?	Stryjewski & Sorenson 2017			2017	2017	Cladogram	(duplicate)
included	included	*Lonchura*	*caniceps*	Estrildidae	MF770315		could not be verified: no structure	could not be verified: no structure	could not be verified: no structure	?	Stryjewski & Sorenson 2017			2017	2017	Cladogram	(duplicate)
included	included	*Lonchura*	*caniceps*	Estrildidae	MF770316		could not be verified: no structure	could not be verified: no structure	could not be verified: no structure	?	Stryjewski & Sorenson 2017			2017	2017	Cladogram	(duplicate)
included	included	*Lonchura*	*caniceps*	Estrildidae	MF770317		could not be verified: no structure	could not be verified: no structure	could not be verified: no structure	?	Stryjewski & Sorenson 2017			2017	2017	Cladogram	(duplicate)
included	included	*Lonchura*	*caniceps*	Estrildidae	MF770318		could not be verified: no structure	could not be verified: no structure	could not be verified: no structure	?	Stryjewski & Sorenson 2017			2017	2017	Cladogram	(duplicate)
included	included	*Lonchura*	*caniceps*	Estrildidae	MF770319		could not be verified: no structure	could not be verified: no structure	could not be verified: no structure	?	Stryjewski & Sorenson 2017			2017	2017	Cladogram	(duplicate)
included	included	*Lonchura*	*caniceps*	Estrildidae	MF770320		could not be verified: no structure	could not be verified: no structure	could not be verified: no structure	?	Stryjewski & Sorenson 2017			2017	2017	Cladogram	(duplicate)
included	included	*Lonchura*	*castaneothorax*	Estrildidae	MF770321	NC_036398	could not be verified: no structure	could not be verified: no structure	could not be verified: no structure	?	Stryjewski & Sorenson 2017			2017	2017	Cladogram	(duplicate)
included	included	*Lonchura*	*castaneothorax*	Estrildidae	MF770322		could not be verified: no structure	could not be verified: no structure	could not be verified: no structure	?	Stryjewski & Sorenson 2017			2017	2017	Cladogram	(duplicate)
included	included	*Lonchura*	*castaneothorax*	Estrildidae	MF770323		could not be verified: no structure	could not be verified: no structure	could not be verified: no structure	?	Stryjewski & Sorenson 2017			2017	2017	Cladogram	(duplicate)
included	included	*Lonchura*	*castaneothorax*	Estrildidae	MF770324		could not be verified: no structure	could not be verified: no structure	could not be verified: no structure	?	Stryjewski & Sorenson 2017			2017	2017	Cladogram	(duplicate)
included	included	*Lonchura*	*castaneothorax*	Estrildidae	MF770325		could not be verified: no structure	could not be verified: no structure	could not be verified: no structure	?	Stryjewski & Sorenson 2017			2017	2017	Cladogram	(duplicate)
included	included	*Lonchura*	*castaneothorax*	Estrildidae	MF770326		could not be verified: no structure	could not be verified: no structure	could not be verified: no structure	?	Stryjewski & Sorenson 2017			2017	2017	Cladogram	(duplicate)
included	included	*Lonchura*	*castaneothorax*	Estrildidae	MF770327		could not be verified: no structure	could not be verified: no structure	could not be verified: no structure	?	Stryjewski & Sorenson 2017			2017	2017	Cladogram	(duplicate)
included	included	*Lonchura*	*castaneothorax*	Estrildidae	MF770328		could not be verified: no structure	could not be verified: no structure	could not be verified: no structure	?	Stryjewski & Sorenson 2017			2017	2017	Cladogram	(duplicate)
included	included	*Lonchura*	*castaneothorax*	Estrildidae	MF770329		could not be verified: no structure	could not be verified: no structure	could not be verified: no structure	?	Stryjewski & Sorenson 2017			2017	2017	Cladogram	(duplicate)
included	included	*Lonchura*	*castaneothorax*	Estrildidae	MF770330		could not be verified: no structure	could not be verified: no structure	could not be verified: no structure	?	Stryjewski & Sorenson 2017			2017	2017	Cladogram	(duplicate)

Sequence database	Phylogeny database	Genus	species	family	GenBank#	RefSeq#	ND2 verified	COI verified	cyt b verified	erroneous sequence?	reference	submitted to GenBank	sequence released on GenBank	publication of paper	YEAR	Phylogeny (mitogenomic)	problematic sequences in tree
included	included	*Lonchura*	*castaneothorax*	Estrildidae	MF770331		could not be verified: no structure	could not be verified: no structure	could not be verified: no structure	?	Stryjewski & Sorenson 2017			2017	2017	Cladogram	(duplicate)
included	included	*Lonchura*	*castaneothorax*	Estrildidae	MF770332		could not be verified: no structure	could not be verified: no structure	could not be verified: no structure	?	Stryjewski & Sorenson 2017			2017	2017	Cladogram	(duplicate)
included	included	*Lonchura*	*castaneothorax*	Estrildidae	MF770333		could not be verified: no structure	could not be verified: no structure	could not be verified: no structure	?	Stryjewski & Sorenson 2017			2017	2017	Cladogram	(duplicate)
included	included	*Lonchura*	*castaneothorax*	Estrildidae	MF770334		could not be verified: no structure	could not be verified: no structure	could not be verified: no structure	?	Stryjewski & Sorenson 2017			2017	2017	Cladogram	(duplicate)
included	included	*Lonchura*	*castaneothorax*	Estrildidae	MF770335		could not be verified: no structure	could not be verified: no structure	could not be verified: no structure	?	Stryjewski & Sorenson 2017			2017	2017	Cladogram	(duplicate)
included	included	*Lonchura*	*castaneothorax*	Estrildidae	MF770336		could not be verified: no structure	could not be verified: no structure	could not be verified: no structure	?	Stryjewski & Sorenson 2017			2017	2017	Cladogram	(duplicate)
included	included	*Lonchura*	*castaneothorax*	Estrildidae	MF770337		could not be verified: no structure	could not be verified: no structure	could not be verified: no structure	?	Stryjewski & Sorenson 2017			2017	2017	Cladogram	(duplicate)
included	included	*Lonchura*	*castaneothorax*	Estrildidae	MF770338		could not be verified: no structure	could not be verified: no structure	could not be verified: no structure	?	Stryjewski & Sorenson 2017			2017	2017	Cladogram	(duplicate)
included	included	*Lonchura*	*castaneothorax*	Estrildidae	MF770339		could not be verified: no structure	could not be verified: no structure	could not be verified: no structure	?	Stryjewski & Sorenson 2017			2017	2017	Cladogram	(duplicate)
included	included	*Lonchura*	*castaneothorax*	Estrildidae	MF770340		could not be verified: no structure	could not be verified: no structure	could not be verified: no structure	?	Stryjewski & Sorenson 2017			2017	2017	Cladogram	(duplicate)
included	included	*Lonchura*	*castaneothorax*	Estrildidae	MF770341		could not be verified: no structure	could not be verified: no structure	could not be verified: no structure	?	Stryjewski & Sorenson 2017			2017	2017	Cladogram	(duplicate)
included	included	*Lonchura*	*castaneothorax*	Estrildidae	MF770342		could not be verified: no structure	could not be verified: no structure	could not be verified: no structure	?	Stryjewski & Sorenson 2017			2017	2017	Cladogram	(duplicate)
included	included	*Lonchura*	*castaneothorax*	Estrildidae	MF770343		could not be verified: no structure	could not be verified: no structure	could not be verified: no structure	?	Stryjewski & Sorenson 2017			2017	2017	Cladogram	(duplicate)
included	included	*Lonchura*	*castaneothorax*	Estrildidae	MF770344		could not be verified: no structure	could not be verified: no structure	could not be verified: no structure	?	Stryjewski & Sorenson 2017			2017	2017	Cladogram	(duplicate)
included	included	*Lonchura*	*castaneothorax*	Estrildidae	MF770345		could not be verified: no structure	could not be verified: no structure	could not be verified: no structure	?	Stryjewski & Sorenson 2017			2017	2017	Cladogram	(duplicate)
included	included	*Lonchura*	*castaneothorax*	Estrildidae	MF770346		could not be verified: no structure	could not be verified: no structure	could not be verified: no structure	?	Stryjewski & Sorenson 2017			2017	2017	Cladogram	(duplicate)
included	included	*Lonchura*	*castaneothorax*	Estrildidae	MF770347		could not be verified: no structure	could not be verified: no structure	could not be verified: no structure	?	Stryjewski & Sorenson 2017			2017	2017	Cladogram	(duplicate)
included	included	*Lonchura*	*castaneothorax*	Estrildidae	MF770348		could not be verified: no structure	could not be verified: no structure	could not be verified: no structure	?	Stryjewski & Sorenson 2017			2017	2017	Cladogram	(duplicate)

Sequence database	Phylogeny database	Genus	species	family	GenBank#	RefSeq#	ND2 verified	COI verified	cyt b verified	erroneous sequence?	reference	submitted to GenBank	sequence released on GenBank	publication of paper	YEAR	Phylogeny (mitogenomic)	problematic sequences in tree
included	included	*Lonchura*	*castaneothorax*	Estrildidae	MF770349		could not be verified: no structure	could not be verified: no structure	could not be verified: no structure	?	Stryjewski & Sorenson 2017			2017	2017	Cladogram	(duplicate)
included	included	*Lonchura*	*castaneothorax*	Estrildidae	MF770350		could not be verified: no structure	could not be verified: no structure	could not be verified: no structure	?	Stryjewski & Sorenson 2017			2017	2017	Cladogram	(duplicate)
included	included	*Lonchura*	*castaneothorax*	Estrildidae	MF770351		could not be verified: no structure	could not be verified: no structure	could not be verified: no structure	?	Stryjewski & Sorenson 2017			2017	2017	Cladogram	(duplicate)
included	included	*Lonchura*	*castaneothorax*	Estrildidae	MF770352		could not be verified: no structure	could not be verified: no structure	could not be verified: no structure	?	Stryjewski & Sorenson 2017			2017	2017	Cladogram	(duplicate)
included	included	*Lonchura*	*castaneothorax*	Estrildidae	MF770353		could not be verified: no structure	could not be verified: no structure	could not be verified: no structure	?	Stryjewski & Sorenson 2017			2017	2017	Cladogram	(duplicate)
included	included	*Lonchura*	*castaneothorax*	Estrildidae	MF770354		could not be verified: no structure	could not be verified: no structure	could not be verified: no structure	?	Stryjewski & Sorenson 2017			2017	2017	Cladogram	(duplicate)
included	included	*Lonchura*	*castaneothorax*	Estrildidae	MF770355		could not be verified: no structure	could not be verified: no structure	could not be verified: no structure	?	Stryjewski & Sorenson 2017			2017	2017	Cladogram	(duplicate)
included	included	*Lonchura*	*castaneothorax*	Estrildidae	MF770356		could not be verified: no structure	could not be verified: no structure	could not be verified: no structure	?	Stryjewski & Sorenson 2017			2017	2017	Cladogram	(duplicate)
included	included	*Lonchura*	*castaneothorax*	Estrildidae	MF770357		could not be verified: no structure	could not be verified: no structure	could not be verified: no structure	?	Stryjewski & Sorenson 2017			2017	2017	Cladogram	(duplicate)
included	included	*Lonchura*	*castaneothorax*	Estrildidae	MF770358		could not be verified: no structure	could not be verified: no structure	could not be verified: no structure	?	Stryjewski & Sorenson 2017			2017	2017	Cladogram	(duplicate)
included	included	*Lonchura*	*castaneothorax*	Estrildidae	MF770359		could not be verified: no structure	could not be verified: no structure	could not be verified: no structure	?	Stryjewski & Sorenson 2017			2017	2017	Cladogram	(duplicate)
included	included	*Lonchura*	*castaneothorax*	Estrildidae	MF770360		could not be verified: no structure	could not be verified: no structure	could not be verified: no structure	?	Stryjewski & Sorenson 2017			2017	2017	Cladogram	(duplicate)
included	included	*Lonchura*	*castaneothorax*	Estrildidae	MF770361		could not be verified: no structure	could not be verified: no structure	could not be verified: no structure	?	Stryjewski & Sorenson 2017			2017	2017	Cladogram	(duplicate)
included	included	*Lonchura*	*castaneothorax*	Estrildidae	MF770362		could not be verified: no structure	could not be verified: no structure	could not be verified: no structure	?	Stryjewski & Sorenson 2017			2017	2017	Cladogram	(duplicate)
included	included	*Lonchura*	*castaneothorax*	Estrildidae	MF770363		could not be verified: no structure	could not be verified: no structure	could not be verified: no structure	?	Stryjewski & Sorenson 2017			2017	2017	Cladogram	(duplicate)
included	included	*Lonchura*	*castaneothorax*	Estrildidae	MF770364		could not be verified: no structure	could not be verified: no structure	could not be verified: no structure	?	Stryjewski & Sorenson 2017			2017	2017	Cladogram	(duplicate)
included	included	*Lonchura*	*castaneothorax*	Estrildidae	MF770365		could not be verified: no structure	could not be verified: no structure	could not be verified: no structure	?	Stryjewski & Sorenson 2017			2017	2017	Cladogram	(duplicate)
included	included	*Lonchura*	*castaneothorax*	Estrildidae	MF770366		could not be verified: no structure	could not be verified: no structure	could not be verified: no structure	?	Stryjewski & Sorenson 2017			2017	2017	Cladogram	(duplicate)

Sequence database	Phylogeny database	Genus	species	family	GenBank#	RefSeq#	ND2 verified	COI verified	cyt b verified	erroneous sequence?	reference	submitted to GenBank	sequence released on GenBank	publication of paper	YEAR	Phylogeny (mitogenomic)	problematic sequences in tree
included	included	*Lonchura*	*castaneothorax*	Estrildidae	MF770367		could not be verified: no structure	could not be verified: no structure	could not be verified: no structure	?	Stryjewski & Sorenson 2017			2017	2017	Cladogram	(duplicate)
included	included	*Lonchura*	*castaneothorax*	Estrildidae	MF770368		could not be verified: no structure	could not be verified: no structure	could not be verified: no structure	?	Stryjewski & Sorenson 2017			2017	2017	Cladogram	(duplicate)
included	included	*Lonchura*	*castaneothorax*	Estrildidae	MF770369		could not be verified: no structure	could not be verified: no structure	could not be verified: no structure	?	Stryjewski & Sorenson 2017			2017	2017	Cladogram	(duplicate)
included	included	*Lonchura*	*flaviprymna*	Estrildidae	MF770370	NC_036399	could not be verified: no structure	could not be verified: no structure	could not be verified: no structure	?	Stryjewski & Sorenson 2017			2017	2017	Cladogram	(duplicate)
included	included	*Lonchura*	*flaviprymna*	Estrildidae	MF770371		could not be verified: no structure	could not be verified: no structure	could not be verified: no structure	?	Stryjewski & Sorenson 2017			2017	2017	Cladogram	(duplicate)
included	included	*Lonchura*	*flaviprymna*	Estrildidae	MF770372		could not be verified: no structure	could not be verified: no structure	could not be verified: no structure	?	Stryjewski & Sorenson 2017			2017	2017	Cladogram	(duplicate)
included	included	*Lonchura*	*flaviprymna*	Estrildidae	MF770373		could not be verified: no structure	could not be verified: no structure	could not be verified: no structure	?	Stryjewski & Sorenson 2017			2017	2017	Cladogram	(duplicate)
included	included	*Lonchura*	*flaviprymna*	Estrildidae	MF770374		could not be verified: no structure	could not be verified: no structure	could not be verified: no structure	?	Stryjewski & Sorenson 2017			2017	2017	Cladogram	(duplicate)
included	included	*Lonchura*	*flaviprymna*	Estrildidae	MF770375		could not be verified: no structure	could not be verified: no structure	could not be verified: no structure	?	Stryjewski & Sorenson 2017			2017	2017	Cladogram	(duplicate)
included	included	*Lonchura*	*flaviprymna*	Estrildidae	MF770376		could not be verified: no structure	could not be verified: no structure	could not be verified: no structure	?	Stryjewski & Sorenson 2017			2017	2017	Cladogram	(duplicate)
included	included	*Lonchura*	*flaviprymna*	Estrildidae	MF770377		could not be verified: no structure	could not be verified: no structure	could not be verified: no structure	?	Stryjewski & Sorenson 2017			2017	2017	Cladogram	(duplicate)
included	included	*Lonchura*	*flaviprymna*	Estrildidae	MF770378		could not be verified: no structure	could not be verified: no structure	could not be verified: no structure	?	Stryjewski & Sorenson 2017			2017	2017	Cladogram	(duplicate)
included	included	*Lonchura*	*flaviprymna*	Estrildidae	MF770379		could not be verified: no structure	could not be verified: no structure	could not be verified: no structure	?	Stryjewski & Sorenson 2017			2017	2017	Cladogram	(duplicate)
included	included	*Lonchura*	*forbesi*	Estrildidae	MF770380	NC_036400	could not be verified: no structure	could not be verified: no structure	could not be verified: no structure	?	Stryjewski & Sorenson 2017			2017	2017	Cladogram	(duplicate)
included	included	*Lonchura*	*forbesi*	Estrildidae	MF770381		could not be verified: no structure	could not be verified: no structure	could not be verified: no structure	?	Stryjewski & Sorenson 2017			2017	2017	Cladogram	(duplicate)
included	included	*Lonchura*	*forbesi*	Estrildidae	MF770382		could not be verified: no structure	could not be verified: no structure	could not be verified: no structure	?	Stryjewski & Sorenson 2017			2017	2017	Cladogram	(duplicate)
included	included	*Lonchura*	*forbesi*	Estrildidae	MF770383		could not be verified: no structure	could not be verified: no structure	could not be verified: no structure	?	Stryjewski & Sorenson 2017			2017	2017	Cladogram	(duplicate)
included	included	*Lonchura*	*forbesi*	Estrildidae	MF770384		could not be verified: no structure	could not be verified: no structure	could not be verified: no structure	?	Stryjewski & Sorenson 2017			2017	2017	Cladogram	(duplicate)

Sequence database	Phylogeny database	Genus	species	family	GenBank#	RefSeq#	ND2 verified	COI verified	cyt b verified	erroneous sequence?	reference	submitted to GenBank	sequence released on GenBank	publication of paper	YEAR	Phylogeny (mitogenomic)	problematic sequences in tree
included	included	*Lonchura*	*forbesi*	Estrildidae	MF770385		could not be verified: no structure	could not be verified: no structure	could not be verified: no structure	?	Stryjewski & Sorenson 2017			2017	2017	Cladogram	(duplicate)
included	included	*Lonchura*	*forbesi*	Estrildidae	MF770386		could not be verified: no structure	could not be verified: no structure	could not be verified: no structure	?	Stryjewski & Sorenson 2017			2017	2017	Cladogram	(duplicate)
included	included	*Lonchura*	*forbesi*	Estrildidae	MF770387		could not be verified: no structure	could not be verified: no structure	could not be verified: no structure	?	Stryjewski & Sorenson 2017			2017	2017	Cladogram	(duplicate)
included	included	*Lonchura*	*forbesi*	Estrildidae	MF770388		could not be verified: no structure	could not be verified: no structure	could not be verified: no structure	?	Stryjewski & Sorenson 2017			2017	2017	Cladogram	(duplicate)
included	included	*Lonchura*	*forbesi*	Estrildidae	MF770389		could not be verified: no structure	could not be verified: no structure	could not be verified: no structure	?	Stryjewski & Sorenson 2017			2017	2017	Cladogram	(duplicate)
included	included	*Lonchura*	*grandis*	Estrildidae	MF770390	NC_036401	could not be verified: no structure	could not be verified: no structure	could not be verified: no structure	?	Stryjewski & Sorenson 2017			2017	2017	Cladogram	(duplicate)
included	included	*Lonchura*	*grandis*	Estrildidae	MF770391		could not be verified: no structure	could not be verified: no structure	could not be verified: no structure	?	Stryjewski & Sorenson 2017			2017	2017	Cladogram	(duplicate)
included	included	*Lonchura*	*grandis*	Estrildidae	MF770392		could not be verified: no structure	could not be verified: no structure	could not be verified: no structure	?	Stryjewski & Sorenson 2017			2017	2017	Cladogram	(duplicate)
included	included	*Lonchura*	*grandis*	Estrildidae	MF770393		could not be verified: no structure	could not be verified: no structure	could not be verified: no structure	?	Stryjewski & Sorenson 2017			2017	2017	Cladogram	(duplicate)
included	included	*Lonchura*	*grandis*	Estrildidae	MF770394		could not be verified: no structure	could not be verified: no structure	could not be verified: no structure	?	Stryjewski & Sorenson 2017			2017	2017	Cladogram	(duplicate)
included	included	*Lonchura*	*grandis*	Estrildidae	MF770395		could not be verified: no structure	could not be verified: no structure	could not be verified: no structure	?	Stryjewski & Sorenson 2017			2017	2017	Cladogram	(duplicate)
included	included	*Lonchura*	*grandis*	Estrildidae	MF770396		could not be verified: no structure	could not be verified: no structure	could not be verified: no structure	?	Stryjewski & Sorenson 2017			2017	2017	Cladogram	(duplicate)
included	included	*Lonchura*	*grandis*	Estrildidae	MF770397		could not be verified: no structure	could not be verified: no structure	could not be verified: no structure	?	Stryjewski & Sorenson 2017			2017	2017	Cladogram	(duplicate)
included	included	*Lonchura*	*grandis*	Estrildidae	MF770398		could not be verified: no structure	could not be verified: no structure	could not be verified: no structure	?	Stryjewski & Sorenson 2017			2017	2017	Cladogram	(duplicate)
included	included	*Lonchura*	*grandis*	Estrildidae	MF770399		could not be verified: no structure	could not be verified: no structure	could not be verified: no structure	?	Stryjewski & Sorenson 2017			2017	2017	Cladogram	(duplicate)
included	included	*Lonchura*	*grandis*	Estrildidae	MF770400		could not be verified: no structure	could not be verified: no structure	could not be verified: no structure	?	Stryjewski & Sorenson 2017			2017	2017	Cladogram	(duplicate)
included	included	*Lonchura*	*grandis*	Estrildidae	MF770401		could not be verified: no structure	could not be verified: no structure	could not be verified: no structure	?	Stryjewski & Sorenson 2017			2017	2017	Cladogram	(duplicate)
included	included	*Lonchura*	*grandis*	Estrildidae	MF770402		could not be verified: no structure	could not be verified: no structure	could not be verified: no structure	?	Stryjewski & Sorenson 2017			2017	2017	Cladogram	(duplicate)

Sequence database	Phylogeny database	Genus	species	family	GenBank#	RefSeq#	ND2 verified	COI verified	cyt b verified	erroneous sequence?	reference	submitted to GenBank	sequence released on GenBank	publication of paper	YEAR	Phylogeny (mitogenomic)	problematic sequences in tree
included	included	*Lonchura*	*grandis*	Estrildidae	MF770403		could not be verified: no structure	could not be verified: no structure	could not be verified: no structure	?	Stryjewski & Sorenson 2017			2017	2017	Cladogram	(duplicate)
included	included	*Lonchura*	*grandis*	Estrildidae	MF770404		could not be verified: no structure	could not be verified: no structure	could not be verified: no structure	?	Stryjewski & Sorenson 2017			2017	2017	Cladogram	(duplicate)
included	included	*Lonchura*	*grandis*	Estrildidae	MF770405		could not be verified: no structure	could not be verified: no structure	could not be verified: no structure	?	Stryjewski & Sorenson 2017			2017	2017	Cladogram	(duplicate)
included	included	*Lonchura*	*grandis*	Estrildidae	MF770406		could not be verified: no structure	could not be verified: no structure	could not be verified: no structure	?	Stryjewski & Sorenson 2017			2017	2017	Cladogram	(duplicate)
included	included	*Lonchura*	*grandis*	Estrildidae	MF770407		could not be verified: no structure	could not be verified: no structure	could not be verified: no structure	?	Stryjewski & Sorenson 2017			2017	2017	Cladogram	(duplicate)
included	included	*Lonchura*	*grandis*	Estrildidae	MF770408		could not be verified: no structure	could not be verified: no structure	could not be verified: no structure	?	Stryjewski & Sorenson 2017			2017	2017	Cladogram	(duplicate)
included	included	*Lonchura*	*grandis*	Estrildidae	MF770409		could not be verified: no structure	could not be verified: no structure	could not be verified: no structure	?	Stryjewski & Sorenson 2017			2017	2017	Cladogram	(duplicate)
included	included	*Lonchura*	*hunsteini*	Estrildidae	MF770410	NC_036402	could not be verified: no structure	could not be verified: no structure	could not be verified: no structure	?	Stryjewski & Sorenson 2017			2017	2017	Cladogram	(duplicate)
included	included	*Lonchura*	*hunsteini*	Estrildidae	MF770411		could not be verified: no structure	could not be verified: no structure	could not be verified: no structure	?	Stryjewski & Sorenson 2017			2017	2017	Cladogram	(duplicate)
included	included	*Lonchura*	*hunsteini*	Estrildidae	MF770412		could not be verified: no structure	could not be verified: no structure	could not be verified: no structure	?	Stryjewski & Sorenson 2017			2017	2017	Cladogram	(duplicate)
included	included	*Lonchura*	*hunsteini*	Estrildidae	MF770413		could not be verified: no structure	could not be verified: no structure	could not be verified: no structure	?	Stryjewski & Sorenson 2017			2017	2017	Cladogram	(duplicate)
included	included	*Lonchura*	*hunsteini*	Estrildidae	MF770414		could not be verified: no structure	could not be verified: no structure	could not be verified: no structure	?	Stryjewski & Sorenson 2017			2017	2017	Cladogram	(duplicate)
included	included	*Lonchura*	*hunsteini*	Estrildidae	MF770415		could not be verified: no structure	could not be verified: no structure	could not be verified: no structure	?	Stryjewski & Sorenson 2017			2017	2017	Cladogram	(duplicate)
included	included	*Lonchura*	*hunsteini*	Estrildidae	MF770416		could not be verified: no structure	could not be verified: no structure	could not be verified: no structure	?	Stryjewski & Sorenson 2017			2017	2017	Cladogram	(duplicate)
included	included	*Lonchura*	*hunsteini*	Estrildidae	MF770417		could not be verified: no structure	could not be verified: no structure	could not be verified: no structure	?	Stryjewski & Sorenson 2017			2017	2017	Cladogram	(duplicate)
included	included	*Lonchura*	*hunsteini*	Estrildidae	MF770418		could not be verified: no structure	could not be verified: no structure	could not be verified: no structure	?	Stryjewski & Sorenson 2017			2017	2017	Cladogram	(duplicate)
included	included	*Lonchura*	*hunsteini*	Estrildidae	MF770419		could not be verified: no structure	could not be verified: no structure	could not be verified: no structure	?	Stryjewski & Sorenson 2017			2017	2017	Cladogram	(duplicate)
included	included	*Lonchura*	*leucosticta*	Estrildidae	MF770311	NC_036396	could not be verified: no ND2	could not be verified: no structure	could not be verified: no cyt b	?	Stryjewski & Sorenson 2017			2017	2017	Cladogram	(duplicate)

Sequence database	Phylogeny database	Genus	species	family	GenBank#	RefSeq#	ND2 verified	COI verified	cyt b verified	erroneous sequence?	reference	submitted to GenBank	sequence released on GenBank	publication of paper	YEAR	Phylogeny (mitogenomic)	problematic sequences in tree
included	included	*Lonchura*	*melaena*	Estrildidae	MF770421	NC_036404	could not be verified: no structure	could not be verified: no structure	could not be verified: no structure	?	Stryjewski & Sorenson 2017			2017	2017	Cladogram	(duplicate)
included	included	*Lonchura*	*melaena*	Estrildidae	MF770422		could not be verified: no structure	could not be verified: no structure	could not be verified: no structure	?	Stryjewski & Sorenson 2017			2017	2017	Cladogram	(duplicate)
included	included	*Lonchura*	*melaena*	Estrildidae	MF770423		could not be verified: no structure	could not be verified: no structure	could not be verified: no structure	?	Stryjewski & Sorenson 2017			2017	2017	Cladogram	(duplicate)
included	included	*Lonchura*	*melaena*	Estrildidae	MF770424		could not be verified: no structure	could not be verified: no structure	could not be verified: no structure	?	Stryjewski & Sorenson 2017			2017	2017	Cladogram	(duplicate)
included	included	*Lonchura*	*melaena*	Estrildidae	MF770425		could not be verified: no structure	could not be verified: no structure	could not be verified: no structure	?	Stryjewski & Sorenson 2017			2017	2017	Cladogram	(duplicate)
included	included	*Lonchura*	*melaena*	Estrildidae	MF770426		could not be verified: no structure	could not be verified: no structure	could not be verified: no structure	?	Stryjewski & Sorenson 2017			2017	2017	Cladogram	(duplicate)
included	included	*Lonchura*	*melaena*	Estrildidae	MF770427		could not be verified: no structure	could not be verified: no structure	could not be verified: no structure	?	Stryjewski & Sorenson 2017			2017	2017	Cladogram	(duplicate)
included	included	*Lonchura*	*melaena*	Estrildidae	MF770428		could not be verified: no structure	could not be verified: no structure	could not be verified: no structure	?	Stryjewski & Sorenson 2017			2017	2017	Cladogram	(duplicate)
included	included	*Lonchura*	*melaena*	Estrildidae	MF770429		could not be verified: no structure	could not be verified: no structure	could not be verified: no structure	?	Stryjewski & Sorenson 2017			2017	2017	Cladogram	(duplicate)
included	included	*Lonchura*	*melaena*	Estrildidae	MF770430		could not be verified: no structure	could not be verified: no structure	could not be verified: no structure	?	Stryjewski & Sorenson 2017			2017	2017	Cladogram	(duplicate)
included	included	*Lonchura*	*melaena*	Estrildidae	MF770431		could not be verified: no structure	could not be verified: no structure	could not be verified: no structure	?	Stryjewski & Sorenson 2017			2017	2017	Cladogram	(duplicate)
included	included	*Lonchura*	*melaena*	Estrildidae	MF770432		could not be verified: no structure	could not be verified: no structure	could not be verified: no structure	?	Stryjewski & Sorenson 2017			2017	2017	Cladogram	(duplicate)
included	included	*Lonchura*	*melaena*	Estrildidae	MF770433		could not be verified: no structure	could not be verified: no structure	could not be verified: no structure	?	Stryjewski & Sorenson 2017			2017	2017	Cladogram	(duplicate)
included	included	*Lonchura*	*melaena*	Estrildidae	MF770434		could not be verified: no structure	could not be verified: no structure	could not be verified: no structure	?	Stryjewski & Sorenson 2017			2017	2017	Cladogram	(duplicate)
included	included	*Lonchura*	*nevermanni*	Estrildidae	MF770435	NC_036405	could not be verified: no structure	could not be verified: no structure	could not be verified: no structure	?	Stryjewski & Sorenson 2017			2017	2017	Cladogram	(duplicate)
included	included	*Lonchura*	*nevermanni*	Estrildidae	MF770436		could not be verified: no structure	could not be verified: no structure	could not be verified: no structure	?	Stryjewski & Sorenson 2017			2017	2017	Cladogram	(duplicate)
included	included	*Lonchura*	*nevermanni*	Estrildidae	MF770437		could not be verified: no structure	could not be verified: no structure	could not be verified: no structure	?	Stryjewski & Sorenson 2017			2017	2017	Cladogram	(duplicate)
included	included	*Lonchura*	*nevermanni*	Estrildidae	MF770438		could not be verified: no structure	could not be verified: no structure	could not be verified: no structure	?	Stryjewski & Sorenson 2017			2017	2017	Cladogram	(duplicate)

Sequence database	Phylogeny database	Genus	species	family	GenBank#	RefSeq#	ND2 verified	COI verified	cyt b verified	erroneous sequence?	reference	submitted to GenBank	sequence released on GenBank	publication of paper	YEAR	Phylogeny (mitogenomic)	problematic sequences in tree
included	included	*Lonchura*	*nevermanni*	Estrildidae	MF770439		could not be verified: no structure	could not be verified: no structure	could not be verified: no structure	?	Stryjewski & Sorenson 2017			2017	2017	Cladogram	(duplicate)
included	included	*Lonchura*	*nevermanni*	Estrildidae	MF770440		could not be verified: no structure	could not be verified: no structure	could not be verified: no structure	?	Stryjewski & Sorenson 2017			2017	2017	Cladogram	(duplicate)
included	included	*Lonchura*	*nevermanni*	Estrildidae	MF770441		could not be verified: no structure	could not be verified: no structure	could not be verified: no structure	?	Stryjewski & Sorenson 2017			2017	2017	Cladogram	(duplicate)
included	included	*Lonchura*	*nevermanni*	Estrildidae	MF770442		could not be verified: no structure	could not be verified: no structure	could not be verified: no structure	?	Stryjewski & Sorenson 2017			2017	2017	Cladogram	(duplicate)
included	included	*Lonchura*	*nevermanni*	Estrildidae	MF770443		could not be verified: no structure	could not be verified: no structure	could not be verified: no structure	?	Stryjewski & Sorenson 2017			2017	2017	Cladogram	(duplicate)
included	included	*Lonchura*	*nevermanni*	Estrildidae	MF770444		could not be verified: no structure	could not be verified: no structure	could not be verified: no structure	?	Stryjewski & Sorenson 2017			2017	2017	Cladogram	(duplicate)
included	included	*Lonchura*	*nigerrima*	Estrildidae	MF770445	NC_036406	could not be verified: no structure	could not be verified: no structure	could not be verified: no structure	?	Stryjewski & Sorenson 2017			2017	2017	Cladogram	(duplicate)
included	included	*Lonchura*	*nigerrima*	Estrildidae	MF770446		could not be verified: no structure	could not be verified: no structure	could not be verified: no structure	?	Stryjewski & Sorenson 2017			2017	2017	Cladogram	(duplicate)
included	included	*Lonchura*	*nigerrima*	Estrildidae	MF770447		could not be verified: no structure	could not be verified: no structure	could not be verified: no structure	?	Stryjewski & Sorenson 2017			2017	2017	Cladogram	(duplicate)
included	included	*Lonchura*	*nigerrima*	Estrildidae	MF770448		could not be verified: no structure	could not be verified: no structure	could not be verified: no structure	?	Stryjewski & Sorenson 2017			2017	2017	Cladogram	(duplicate)
included	included	*Lonchura*	*nigerrima*	Estrildidae	MF770449		could not be verified: no structure	could not be verified: no structure	could not be verified: no structure	?	Stryjewski & Sorenson 2017			2017	2017	Cladogram	(duplicate)
included	included	*Lonchura*	*nigerrima*	Estrildidae	MF770450		could not be verified: no structure	could not be verified: no structure	could not be verified: no structure	?	Stryjewski & Sorenson 2017			2017	2017	Cladogram	(duplicate)
included	included	*Lonchura*	*nigerrima*	Estrildidae	MF770451		could not be verified: no structure	could not be verified: no structure	could not be verified: no structure	?	Stryjewski & Sorenson 2017			2017	2017	Cladogram	(duplicate)
included	included	*Lonchura*	*nigerrima*	Estrildidae	MF770452		could not be verified: no structure	could not be verified: no structure	could not be verified: no structure	?	Stryjewski & Sorenson 2017			2017	2017	Cladogram	(duplicate)
included	included	*Lonchura*	*nigerrima*	Estrildidae	MF770453		could not be verified: no structure	could not be verified: no structure	could not be verified: no structure	?	Stryjewski & Sorenson 2017			2017	2017	Cladogram	(duplicate)
included	included	*Lonchura*	*nigerrima*	Estrildidae	MF770454		could not be verified: no structure	could not be verified: no structure	could not be verified: no structure	?	Stryjewski & Sorenson 2017			2017	2017	Cladogram	(duplicate)
included	included	*Lonchura*	*punctulata*	Estrildidae	KR184724	NC_028036	verified	verified	verified	no	Bao et al 2016			2016	2016	Cladogram	-
included	included	*Lonchura*	*spectabilis*	Estrildidae	MF770455	NC_036407	could not be verified: no structure	could not be verified: no structure	could not be verified: no structure	?	Stryjewski & Sorenson 2017			2017	2017	Cladogram	(duplicate)

Sequence database	Phylogeny database	Genus	species	family	GenBank#	RefSeq#	ND2 verified	COI verified	cyt b verified	erroneous sequence?	reference	submitted to GenBank	sequence released on GenBank	publication of paper	YEAR	Phylogeny (mitogenomic)	problematic sequences in tree
included	included	*Lonchura*	*spectabilis*	Estrildidae	MF770456		could not be verified: no structure	could not be verified: no structure	could not be verified: no structure	?	Stryjewski & Sorenson 2017			2017	2017	Cladogram	(duplicate)
included	included	*Lonchura*	*spectabilis*	Estrildidae	MF770457		could not be verified: no structure	could not be verified: no structure	could not be verified: no structure	?	Stryjewski & Sorenson 2017			2017	2017	Cladogram	(duplicate)
included	included	*Lonchura*	*spectabilis*	Estrildidae	MF770458		could not be verified: no structure	could not be verified: no structure	could not be verified: no structure	?	Stryjewski & Sorenson 2017			2017	2017	Cladogram	(duplicate)
included	included	*Lonchura*	*spectabilis*	Estrildidae	MF770459		could not be verified: no structure	could not be verified: no structure	could not be verified: no structure	?	Stryjewski & Sorenson 2017			2017	2017	Cladogram	(duplicate)
included	included	*Lonchura*	*spectabilis*	Estrildidae	MF770460		could not be verified: no structure	could not be verified: no structure	could not be verified: no structure	?	Stryjewski & Sorenson 2017			2017	2017	Cladogram	(duplicate)
included	included	*Lonchura*	*spectabilis*	Estrildidae	MF770461		could not be verified: no structure	could not be verified: no structure	could not be verified: no structure	?	Stryjewski & Sorenson 2017			2017	2017	Cladogram	(duplicate)
included	included	*Lonchura*	*spectabilis*	Estrildidae	MF770462		could not be verified: no structure	could not be verified: no structure	could not be verified: no structure	?	Stryjewski & Sorenson 2017			2017	2017	Cladogram	(duplicate)
included	included	*Lonchura*	*spectabilis*	Estrildidae	MF770463		could not be verified: no structure	could not be verified: no structure	could not be verified: no structure	?	Stryjewski & Sorenson 2017			2017	2017	Cladogram	(duplicate)
included	included	*Lonchura*	*spectabilis*	Estrildidae	MF770464		could not be verified: no structure	could not be verified: no structure	could not be verified: no structure	?	Stryjewski & Sorenson 2017			2017	2017	Cladogram	(duplicate)
included	included	*Lonchura*	*spectabilis*	Estrildidae	MF770465		could not be verified: no structure	could not be verified: no structure	could not be verified: no structure	?	Stryjewski & Sorenson 2017			2017	2017	Cladogram	(duplicate)
included	included	*Lonchura*	*spectabilis*	Estrildidae	MF770466		could not be verified: no structure	could not be verified: no structure	could not be verified: no structure	?	Stryjewski & Sorenson 2017			2017	2017	Cladogram	(duplicate)
included	included	*Lonchura*	*spectabilis*	Estrildidae	MF770467		could not be verified: no structure	could not be verified: no structure	could not be verified: no structure	?	Stryjewski & Sorenson 2017			2017	2017	Cladogram	(duplicate)
included	included	*Lonchura*	*spectabilis*	Estrildidae	MF770468		could not be verified: no structure	could not be verified: no structure	could not be verified: no structure	?	Stryjewski & Sorenson 2017			2017	2017	Cladogram	(duplicate)
included	included	*Lonchura*	*spectabilis*	Estrildidae	MF770469		could not be verified: no structure	could not be verified: no structure	could not be verified: no structure	?	Stryjewski & Sorenson 2017			2017	2017	Cladogram	(duplicate)
included	included	*Lonchura*	*spectabilis*	Estrildidae	MF770470		could not be verified: no structure	could not be verified: no structure	could not be verified: no structure	?	Stryjewski & Sorenson 2017			2017	2017	Cladogram	(duplicate)
included	included	*Lonchura*	*spectabilis*	Estrildidae	MF770471		could not be verified: no structure	could not be verified: no structure	could not be verified: no structure	?	Stryjewski & Sorenson 2017			2017	2017	Cladogram	(duplicate)
included	included	*Lonchura*	*spectabilis*	Estrildidae	MF770472		could not be verified: no structure	could not be verified: no structure	could not be verified: no structure	?	Stryjewski & Sorenson 2017			2017	2017	Cladogram	(duplicate)
included	included	*Lonchura*	*spectabilis*	Estrildidae	MF770473		could not be verified: no structure	could not be verified: no structure	could not be verified: no structure	?	Stryjewski & Sorenson 2017			2017	2017	Cladogram	(duplicate)

Sequence database	Phylogeny database	Genus	species	family	GenBank#	RefSeq#	ND2 verified	COI verified	cyt b verified	erroneous sequence?	reference	submitted to GenBank	sequence released on GenBank	publication of paper	YEAR	Phylogeny (mitogenomic)	problematic sequences in tree
included	included	*Lonchura*	*spectabilis*	Estrildidae	MF770474		could not be verified: no structure	could not be verified: no structure	could not be verified: no structure	?	Stryjewski & Sorenson 2017			2017	2017	Cladogram	(duplicate)
included	excluded: no paper (yet)	*Lonchura*	*striata domestica*	Estrildidae	CM016794		verified	could not be verified: no structure	could not be verified: no structure	no	Mets, D.G., Colquitt, B.M. and Brainard, M.S.	2019	2019	unpubl	2019	-	
included	included	*Lonchura*	*striata swinhoei*	Estrildidae	KR080134	NC_029475	verified	could not be verified: no structure	could not be verified: no structure	no	Yang et al 2016d			2016	2016	Cladogram	-
included	included	*Lonchura*	*stygia*	Estrildidae	MF770475	NC_036408	could not be verified: no structure	could not be verified: no structure	could not be verified: no structure	?	Stryjewski & Sorenson 2017			2017	2017	Cladogram	(duplicate)
included	included	*Lonchura*	*stygia*	Estrildidae	MF770476		could not be verified: no structure	could not be verified: no structure	could not be verified: no structure	?	Stryjewski & Sorenson 2017			2017	2017	Cladogram	(duplicate)
included	included	*Lonchura*	*stygia*	Estrildidae	MF770477		could not be verified: no structure	could not be verified: no structure	could not be verified: no structure	?	Stryjewski & Sorenson 2017			2017	2017	Cladogram	(duplicate)
included	included	*Lonchura*	*stygia*	Estrildidae	MF770478		could not be verified: no structure	could not be verified: no structure	could not be verified: no structure	?	Stryjewski & Sorenson 2017			2017	2017	Cladogram	(duplicate)
included	included	*Lonchura*	*stygia*	Estrildidae	MF770479		could not be verified: no structure	could not be verified: no structure	could not be verified: no structure	?	Stryjewski & Sorenson 2017			2017	2017	Cladogram	(duplicate)
included	included	*Lonchura*	*stygia*	Estrildidae	MF770480		could not be verified: no structure	could not be verified: no structure	could not be verified: no structure	?	Stryjewski & Sorenson 2017			2017	2017	Cladogram	(duplicate)
included	included	*Lonchura*	*stygia*	Estrildidae	MF770481		could not be verified: no structure	could not be verified: no structure	could not be verified: no structure	?	Stryjewski & Sorenson 2017			2017	2017	Cladogram	(duplicate)
included	included	*Lonchura*	*stygia*	Estrildidae	MF770482		could not be verified: no structure	could not be verified: no structure	could not be verified: no structure	?	Stryjewski & Sorenson 2017			2017	2017	Cladogram	(duplicate)
included	included	*Lonchura*	*stygia*	Estrildidae	MF770483		could not be verified: no structure	could not be verified: no structure	could not be verified: no structure	?	Stryjewski & Sorenson 2017			2017	2017	Cladogram	(duplicate)
included	included	*Lonchura*	*stygia*	Estrildidae	MF770484		could not be verified: no structure	could not be verified: no structure	could not be verified: no structure	?	Stryjewski & Sorenson 2017			2017	2017	Cladogram	(duplicate)
included	included	*Lophoceros (Tockus)*	*camurus*	Bucerotidae	MK060134		could not be verified: no ND2	verified	could not be verified: no cyt b	no	Tamashiro et al 2019			2019	2019	Phylogram	(duplicate)
included	included	*Lophophanes*	*dichrous*	Paridae	KX388477		verified	verified	verified	no	Li et al 2016b (Li et al 2017)			2016	2016	Phylogram	(duplicate)
included	included	*Lophophorus*	*impejanus*	Phasianidae	MF975712	NC_040850	verified	could not be verified: no COI	verified	no	Chen et al 2018a			2018	2018	Cladogram	-
included	included	*Lophophorus*	*lhuysii*	Phasianidae	GQ871234	NC_013979	verified	verified	verified	no	Ma et al 2010			2010	2010	Phylogram	*Phasianus versicolor, Francolinus pintadeanus*
included	included	*Lophophorus*	*sclateri*	Phasianidae	FJ752432	NC_020589	verified	could not be verified: no COI	verified	no	Shen et al 2010			2010	2010	Phylogram	(duplicate)

Sequence database	Phylogeny database	Genus	species	family	GenBank#	RefSeq#	ND2 verified	COI verified	cyt b verified	erroneous sequence?	reference	submitted to GenBank	sequence released on GenBank	publication of paper	YEAR	Phylogeny (mitogenomic)	problematic sequences in tree
included	excluded: no paper (yet)	*Lophornis*	*magnificus*	Trochilidae	KT265276	NC_033414	could not be verified: no ND2	could not be verified: no COI	could not be verified: no cyt b	?	Ruschi, P.A., Jennings, W.B. and Prosdocimi, F.	2015	2016	unpubl	2016	-	
included	included	*Lophura*	*edwardsi*	Phasianidae	KY411595		could not be verified: no ND2	could not be verified: no COI	verified	no	Wang et al 2017a			2017	2017	Phylogram	(duplicate)
included	excluded: no paper (yet)	*Lophura*	*ignita*	Phasianidae	AB164627	NC_010781	could not be verified: no ND2	could not be verified: no COI	verified	no	Kato, S., Nishibori, M. and Yasue, H.	2004		unpubl	2004	-	
included	included	*Lophura*	*nycthemera*	Phasianidae	EU417810		could not be verified: no structure	verified	verified	no	Shen et al 2009			2009	2009	Cladogram	(duplicate)
included	included	*Lophura*	*swinhoii*	Phasianidae	KF218954	NC_023779	verified	could not be verified: reference COI may be misidentified	verified	no	Jiang et al 2014			2014	2014	Phylogram	*Phasianus versicolor*
included	included	*Lorius*	*chlorocercus*	Psittaculidae	MN515396		verified	verified	verified	no	Chen 2019		2020	2019	2019	Phylogram	-
included	included	*Loxia*	*curvirostra*	Fringillidae	KM078800	NC_025623	could not be verified: no structure	could not be verified: no structure	could not be verified: no structure	?	Lerner et al 2011			2011	2011	Cladogram	(duplicate)
included	included	*Loxops*	*mana*	Fringillidae	KM078768	NC_025598	could not be verified: no ND2	could not be verified: no structure	verified	no	Lerner et al 2011			2011	2011	Cladogram	(duplicate)
included	included	*Loxops*	*caeruleirostris*	Fringillidae	KM078776	NC_025605	could not be verified: no ND2	could not be verified: no structure	verified	no	Lerner et al 2011			2011	2011	Cladogram	(duplicate)
included	included	*Loxops*	*coccineus*	Fringillidae	KM078785	NC_025612	could not be verified: no ND2	could not be verified: no structure	verified	no	Lerner et al 2011			2011	2011	Cladogram	(duplicate)
included	excluded: no paper (yet)	*Luscinia (Calliope)*	*calliope*	Muscicapidae	HQ690246	NC_015074	verified	verified	verified	no	Kan, X.-Z. and Li, X.-F.	2010	?	unpubl	2010	-	
included	included	*Lyrurus*	*tetrix*	Phasianidae	KF955638	NC_024554	verified	verified	verified	no	Li et al 2016i			2016	2016	no	
included	included	*Machlolophus*	*spilonotus*	Paridae	KX388476		verified	verified	verified	no	Li et al 2016b (Li et al 2017)			2016	2016	Phylogram	(duplicate)
included	included	*Macroagelaius*	*imthurni*	Icteridae	JX516073	NC_018810	could not be verified: no ND2	verified	verified	no	Powell et al 2013			2013	2013	Cladogram	(duplicate)
included	included	*Malacoptila*	*fusca*	Bucconidae	MK060135		could not be verified: no ND2	verified	verified	no	Tamashiro et al 2019			2019	2019	Phylogram	(duplicate)
included	excluded: no paper (yet)	*Malurus*	*cyaneus samueli*	Maluridae	CM019239		verified	could not be verified: no COI	could not be verified: no resolution	no	Penalba, J., Deng, Y., Fang, Q., Joseph, L., Moritz, C. and Cockburn, A.		2019	unpubl	2019	-	
included	included	*Malurus*	*melanocephalus*	Maluridae	KJ909199	NC_024873	verified	could not be verified: no COI	could not be verified: no cyt b	no	Barker 2014			2014	2014	Cladogram	(duplicate)
included	included	*Manorina*	*melanocephala*	Meliphagidae	KY994587		verified	could not be verified: no COI	could not be verified: no structure	no	Lamb et al 2018			2018	2018	Phylogram	(duplicate)

Sequence database	Phylogeny database	Genus	species	family	GenBank#	RefSeq#	ND2 verified	COI verified	cyt b verified	erroneous sequence?	reference	submitted to GenBank	sequence released on GenBank	publication of paper	YEAR	Phylogeny (mitogenomic)	problematic sequences in tree
included	included	*Megadyptes*	*antipodes antipodes*	Spheniscidae	MK290255		could not be verified: no ND2	verified	could not be verified: no cyt b	no	Cole et al 2019			2019	2019	Cladogram	(duplicate)
included	included	*Megadyptes*	*antipodes antipodes*	Spheniscidae	MK290256		could not be verified: no ND2	verified	could not be verified: no cyt b	no	Cole et al 2019			2019	2019	Cladogram	(duplicate)
included	included	*Megalaima*	*virens*	Megalaimidae	MK060136	NC_039891	verified	could not be verified: no COI	verified	no	Tamashiro et al 2019			2019	2019	Phylogram	(duplicate)
included	included	*Megalurus*	*punctatus*	Locustellidae	KC545398	NC_029138	could not be verified: no ND2	verified	verified	no	Gibb et al 2015			2015	2015	Phylogram	(duplicate)
included	included	*Melamprosops*	*phaeosoma*	Fringillidae	KM078793	NC_025617	could not be verified: no ND2	could not be verified: no structure	could not be verified: no cyt b	?	Lerner et al 2011			2011	2011	Cladogram	(duplicate)
included	included	*Melanitta*	*deglandi*	Anatidae	n.a.		could not be verified: no ND2	could not be verified: no structure	could not be verified: no structure	?	Buckner et al 2018			2018	2018	Phylogram	(duplicate)
included	included	*Melanitta*	*nigra (americana)*	Anatidae	n.a.		verified	verified	dubious	no	Buckner et al 2018			2018	2018	Phylogram	(duplicate)
included	included	*Melanocorypha*	*mongolica*	Alaudidae	KY887027	NC_036760	could not be verified: no ND2	verified	verified	no	Zeng et al 2017			2017	2017	Phylogram	-
included	included	*Meleagris*	*gallopavo*	Phasianidae	EF153719		verified	verified	verified	no	Guan et al 2009			2009	2009	Phylogram	-
included	excluded: no paper (yet)	*Meleagris*	*gallopavo*	Phasianidae	JF275060	NC_010195	verified	verified	verified	no	Reed, K.M.	2011	?	unpubl	2011	-	
included	included	*Meleagris (Agriocharis)*	*ocellata*	Phasianidae	KU094576		could not be verified: no ND2	could not be verified: no COI	could not be verified: no cyt b	?	Persons et al 2016			2016	2016	Cladogram	-
included	included	*Melopsittacus*	*undulatus*	Psittaculidae	KM611477		verified	verified	verified	no	Eberhard & Wright 2016			2016	2016	Cladogram	(duplicate)
included	excluded: no paper (yet)	*Melopsittacus*	*undulatus*	Psittaculidae	EF450826	NC_009134	verified	verified	verified	no	Guan, X., Samuels, D.C. and Smith, E.J.	2007	?	unpubl	2007	-	
included	excluded: no paper (yet)	*Melospiza*	*melodia*	Passerellidae	FJ236290		verified	verified	verified	no	Carson, R.J. and Spicer, G.S.	2008	?	unpubl	2008	-	
included	included	*Menura*	*novaehollandiae*	Menuridae	AY542313	NC_007883	verified	could not be verified: no COI	verified	no	Slack et al 2007			2007	2007	network	(duplicate)
included	included	*Mergus*	*merganser*	Anatidae	MK862101		could not be verified: unclear if misid'd or ref misid'd	verified	verified	no	Lee et al 2019		2019	2019	2019	Phylogram	*Anas clypeata, Anas falcata, Anas crecca*
included	included	*Mergus*	*merganser*	Anatidae	KU140667	NC_040986	could not be verified: unclear if misid'd or ref misid'd	verified	verified	no	Sun et al 2017a			2017	2017	Cladogram	*Branta bernicla, Anas falcata, Anas crecca, Anser fabalis, Netta rufina, Aix galericulata*
included	included	*Mergus*	*merganser americanus*	Anatidae	n.a.		verified	verified	verified	no	Buckner et al 2018			2018	2018	Phylogram	(duplicate)

Sequence database	Phylogeny database	Genus	species	family	GenBank#	RefSeq#	ND2 verified	COI verified	cyt b verified	erroneous sequence?	reference	submitted to GenBank	sequence released on GenBank	publication of paper	YEAR	Phylogeny (mitogenomic)	problematic sequences in tree
included	included	*Mergus*	*serrator*	Anatidae	n.a.		verified	verified	could not be verfied: some reference taxa may be misidentified	no	Buckner et al 2018			2018	2018	Phylogram	(duplicate)
included	included	*Mergus*	*squamatus*	Anatidae	HQ833701	NC_016723	could not be verified: no ND2	verified	could not be verified: no cyt b	no	Liu et al 2012			2012	2012	no	
included	excluded: no paper (yet)	*Merops*	*nubicus*	Meropidae	CM020464		verified	verified	verified	no	Gilbert, M.T.P., Caswara, C., Chow, W., Wood, J., Howe, K., Bertelson, M., Rhie, A., Fedrigo, O. and Jarvis, E.D.		2019	unpubl	2019	-	
included	included	*Merops*	*nubicus*	Meropidae	MK060137		verified	verified	verified	?	Tamashiro et al 2019			2019	2019	Phylogram	(duplicate)
included	included	*Merops*	*viridis*	Meropidae	KU821702	NC_034642	verified	could not be verified: no COI	dubious	?	Huang et al 2017a			2017	2017	no	
included	included	*Micrastur*	*gilvicollis*	Falconidae	DQ780881	NC_008548	verified	verified	verified	no	Gibb et al 2007			2007	2007	Phylogram	(duplicate)
included	included	*Milvus*	*migrans*	Accipitridae	MG930481	NC_038195	verified	verified	verified	no	Jeon et al 2018			2018	2018	Phylogram	*Accipiter gularis*
included	included	*Minla*	*ignotincta*	Timaliidae	KT995474	NC_030588	verified	verified	verified	no	Li et al 2016f			2016	2016	Phylogram	*Garrulax perspicillatus*
included	included	*Minla (Siva)*	*cyanouroptera*	Timaliidae	MK779708	NC_045387	verified	verified	verified	no	He et al 2019c		2019	2019	2019	Phylogram	*Garrulax albogularis, Garrulax perspicillatus*
included	included	*Minla (Siva)*	*cyanouroptera*	Timaliidae	MK940810		verified	verified	verified	no	Huang et al 2019d		2019	2019	2019	Phylogram	*Garrulax perspicillatus*
included	included	*Mionectes*	*oleagineus*	Tyrannidae	KJ742590		verified	verified	verified	no	Loaiza et al 2014			2014	2014	no	
included	included	*Mionectes*	*oleagineus*	Tyrannidae	KJ742591	NC_024682	verified	verified	verified	no	Loaiza et al 2014			2014	2014	no	
included	included	*Moho*	*braccathus*	Mohoidae	KU158189	NC_031348	could not be verified: no ND2	could not be verified: no COI	could not be verified: no cyt b	?	Anmarkrud & Lifjeld 2017			2017	2017	no	
included	included	*Mohoua*	*novaeseelandiae*	Acanthizidae	KC545409		verified	could not be verified: sequence does not contain COI	could not be verified: no cyt b	no	Gibb et al 2015			2015	2015	Phylogram	(duplicate)
included	included	*Molothrus*	*aeneus*	Icteridae	JX516067	NC_018806	verified	verified	could not be verified: close, but not sister to cyt b	no	Powell et al 2013			2013	2013	Cladogram	(duplicate)
included	included	*Momotus*	*momota*	Momotidae	MK060138		verified	verified	dubious	?	Tamashiro et al 2019			2019	2019	Phylogram	(duplicate)
included	included	*Monticola*	*cinclorhynchus gularis*	Muscicapidae	KX506858	NC_033536	verified	verified	verified	no	Zhang et al 2016e			2016	2016	Cladogram	*Cyanoptila cyanomelana*
included	excluded: no paper (yet)	*Montifringilla*	*adamsi*	Passeridae	KJ148630	NC_025913	could not be verified: no ND2	could not be verified: no COI	verified	no	Ma, Y.-G., Huang, Y. and Lei, F.-M.	2014	?	unpubl	2014	-	

Sequence database	Phylogeny database	Genus	species	family	GenBank#	RefSeq#	ND2 verified	COI verified	cyt b verified	erroneous sequence?	reference	submitted to GenBank	sequence released on GenBank	publication of paper	YEAR	Phylogeny (mitogenomic)	problematic sequences in tree
included	excluded: no paper (yet)	*Montifringilla*	*blanfordi*	Passeridae	KJ148629	NC_025912	could not be verified: no ND2	could not be verified: no COI	verified	no	Ma, Y.-G., Huang, Y. and Lei, F.-M.	2014	?	unpubl	2014	-	
included	excluded: no paper (yet)	*Montifringilla*	*henrici*	Passeridae	MK183040	NC_042414	could not be verified: no ND2	verified	verified	no	Ma, Y.	2019	2019	unpubl	2019	-	
included	excluded: no paper (yet)	*Montifringilla*	*nivalis*	Passeridae	KJ148628	NC_025911	could not be verified: reference seq may be misidentied	verified	verified	no	Ma, Y.-G., Huang, Y. and Lei, F.-M.	2014	?	unpubl	2014	-	
included	included	*Montifringilla (Onychostruthus)*	*taczanowskii*	Passeridae	KJ148631	NC_025914	could not be verified: no ND2	verified	verified	no	Ma et al 2016			2016	2016	Cladogram	-
included	excluded: no paper (yet)	*Montifringilla (Pyrgilauda)*	*davidiana*	Passeridae	KJ148632	NC_025915	verified	verified	could not be verified; inconsistent with 2 cyt b	no	Ma, Y.-G., Huang, Y. and Lei, F.-M.	2014	?	unpubl	2014	-	
included	included	*Montifringilla (Pyrgilauda)*	*ruficollis*	Passeridae	KC836121	NC_022815	verified	could not be verified: no COI	verified	no	Ma et al 2014			2014	2014	Cladogram	*Cyanoptila cyanomelana, Pseudopodoces humilis, Emberiza chrysophrys*
included	included	*Morus*	*serrator*	Sulidae	GU071056		verified	verified	verified	no	Gibb et al 2013			2013	2013	Phylogram	(duplicate)
included	included	*Motacilla*	*alba*	Motacillidae	KT736087	NC_029229	verified	verified	verified	no	Dong et al 2016b			2016	2016	no	
included	included	*Motacilla*	*cinerea*	Motacillidae	KR092187	NC_027933	verified	verified	verified	no	Zhang et al 2016f			2016	2016	no	
included	excluded: no paper (yet)	*Motacilla*	*lugens*	Motacillidae	KU246035	NC_029703	dubious	verified	misidentified	yes (chimera)	Park, C.E., Park, G.S., Park, Y.J., Kim, M.C., Park, H.C. and Shin, J.H.	2015	2016	unpubl	2016	-	
included	included	*Motacilla*	*tschutschensis*	Motacillidae	MN217252		verified	verified	verified	no	Gao et al 2019b		2020	2019	2019	Cladogram	*Passer ammodendri, Motacilla lugens*
included	included	*Muscicapa*	*griseisticta*	Muscicapidae	MK390479	NC_045181	misidentified	misidentified	misidentified	yes (misID)	Liu et al 2019g		2019	2019	2019	Cladogram	*Cyanoptila cyanomelana, Pseudopodoces humilis, Muscicapa griseisticta*
included	included	*Muscicapa*	*latirostris*	Muscicapidae	MK770602	NC_045375	verified	verified	verified	no	Min et al 2019		2019	2019	2019	no	
included	included	*Muscicapa*	*sibirica*	Muscicapidae	MK770601	NC_045374	verified	verified	verified	no	Lu et al 2019		2019	2019	2019	no	
included	included	*Myadestes*	*myadestinus*	Turdidae	KU158194	NC_031352	could not be verified: no ND2	could not be verified: no COI	could not be verified: no cyt b	?	Anmarkrud & Lifjeld 2017			2017	2017	no	
included	included	*Myiopsitta*	*monachus*	Psittacidae	KM611471	NC_027844	verified	verified	verified	no	Eberhard & Wright 2016			2016	2016	Cladogram	(duplicate)
included	included	*Napothera*	*epilepidota*	Timaliidae	KX831093	NC_035626	verified	verified	verified	no	Huang et al 2019a			2019	2019	Phylogram	(duplicate)
included	included	*Neophema*	*chrysogaster*	Psittaculidae	JX133087	NC_019804	could not be verified: no ND2	could not be verified: no COI	could not be verified: no cyt b	?	Miller et al 2013			2013	2013	no	

Sequence database	Phylogeny database	Genus	species	family	GenBank#	RefSeq#	ND2 verified	COI verified	cyt b verified	erroneous sequence?	reference	submitted to GenBank	sequence released on GenBank	publication of paper	YEAR	Phylogeny (mitogenomic)	problematic sequences in tree
included	included	*Nesopsar*	*nigerrimus*	Icteridae	JX516054	NC_018794	verified	could not be verified: no COI	verified	no	Powell et al 2013			2013	2013	Cladogram	(duplicate)
included	included	*Nesoptilotis (Lichenostomus)*	*leucotis*	Meliphagidae	KY994583		verified	verified	could not be verified: no cyt b	no	Lamb et al 2018			2018	2018	Phylogram	(duplicate)
included	included	*Nesoptilotis (Lichenostomus)*	*leucotis*	Meliphagidae	KY994594		verified	verified	could not be verified: no cyt b	no	Lamb et al 2018			2018	2018	Phylogram	(duplicate)
included	included	*Nestor*	*notabilis*	Strigopidae	KM611472	NC_027845	verified	verified	verified	no	Eberhard & Wright 2016			2016	2016	Cladogram	(duplicate)
included	included	*Nestor*	*notabilis*	Strigopidae	KX369037		verified	verified	verified	no	Mitchell et al 2016			2016	2016	Cladogram	*Thamnophilus nigrocinereus*
included	included	*Nestor*	*notabilis*	Strigopidae	MH133967		verified	verified	verified	no	Urantowka et al 2018			2018	2018	Cladogram	(duplicate)
included	excluded: no paper (yet)	*Netta*	*rufina*	Anatidae	KC466568	NC_024922	verified	dubious	verified	yes (chimera)	Kan, X.-Z. and Li, X.-X.	2013	?	unpubl	2013	-	
included	included	*Niltava*	*davidi*	Muscicapidae	KY024217	NC_039538	verified	verified	verified	no	Zhou et al 2019b			2019	2019	Phylogram	*Cyanoptila cyanomelana, Turdus merula 2,*
included	included	*Ninox*	*novaeseelandiae*	Strigidae	AY309457	NC_005932	could not be verified: no ND2	verified	verified	no	Harrison et al 2004			2004	2004	Phylogram	(duplicate)
included	excluded: no paper (yet)	*Ninox*	*scutulata*	Strigidae	KT943750	NC_029384	verified	verified	verified	no	Park, C.E., Hong, S.J., Park, G.S., Kim, M.C., Park, H.C. and Shin, J.H.	2015	2016	unpubl	2016	-	
included	included	*Ninox*	*strenua*	Strigidae	KX529654	NC_033967	problematic	could not be verified: no COI	problematic	yes (seq errors/numts)	Sarker et al 2016			2016	2016	Phylogram	*Bubo bubo, Otus bakkamoena, Otus scops, Strix leptogrammica, Ninox strenua*
included	included	*Ninox (Sceloglaux)*	*albifacies*	Strigidae	KX098448		could not be verified: no ND2	could not be verified: no COI	could not be verified: no cyt b	?	Wood et al 2017			2017	2017	no	
included	included	*Nipponia*	*nippon*	Threskiornithidae	MN047457		could not be verified: no ND2	verified	verified	no	Kim et al 2019b		2019	2019	2019	Phylogram	-
included	excluded: no paper (yet)	*Nipponia*	*nippon*	Threskiornithidae	AB104902	NC_008132	could not be verified: no ND2	verified	verified	no	Kodama, Y., Kawasaki, D., Segawa, R.D., Ishii, S. and Aotsuka, T.	2003		unpubl	2003	-	
included	excluded: no paper (yet)	*Nisaetus*	*alboniger*	Accipitridae	AP008239	NC_007599	could not be verified: not sister to one ND2	could not be verified: no COI	verified	no	Asai, S., Yamamoto, Y., Kakizawa, R. and Yamagishi, S.	2004		unpubl	2004	-	
included	included	*Nisaetus*	*nipalensis*	Accipitridae	AP008238	NC_007598	verified	verified	verified	no	Asai et al 2006			2006	2006	no	

Sequence database	Phylogeny database	Genus	species	family	GenBank#	RefSeq#	ND2 verified	COI verified	cyt b verified	erroneous sequence?	reference	submitted to GenBank	sequence released on GenBank	publication of paper	YEAR	Phylogeny (mitogenomic)	problematic sequences in tree
included	included	*Notiomystis*	*cincta*	Meliphagidae	KJ909194		verified	verified	could not be verified: no cyt b	no	Barker 2014			2014	2014	Cladogram	(duplicate)
included	included	*Notiomystis*	*cincta*	Meliphagidae	KC545400	NC_029140	verified	verified	could not be verified: no cyt b	no	Gibb et al 2015			2015	2015	Phylogram	(duplicate)
included	included	*Nucifraga*	*columbiana*	Corvidae	KF509923	NC_022839	could not be verified: no ND2	verified	could not be verified: no cyt b	no	Barker et al 2015			2015	2015	no	
included	included	*Numenius*	*phaeopus*	Scolopacidae	KP308149	NC_030507	verified	verified	verified	no	Ding et al 2016c			2016	2016	no	
included	included	*Numenius*	*tenuirostris*	Scolopacidae	MK108195	NC_042233	could not be verified: no ND2	could not be verified: no COI	could not be verified: no cyt b	?	Sharko et al 2019		2019	2019	2019	Phylogram	-
included	excluded: no paper (yet)	*Numida*	*meleagris*	Numididae	KP218504		verified	verified	verified	no	Adeola, A.C.	2014	?	unpubl	2014	-	
included	included	*Numida*	*meleagris*	Numididae	AP005595		verified	verified	verified	no	Nishibori et al 2004			2004	2004	no	
included	excluded: no paper (yet)	*Numida*	*meleagris*	Numididae	KY865420	NC_034374	verified	verified	verified	no	Vignal, A. and Warren, W.	2017	2017	unpubl	2017	-	
included	included	*Nyctibius*	*grandis*	Nyctibiidae	EU344977		could not be verified: no ND2	verified	verified	no	Pratt et al 2009			2009	2009	Phylogram	(duplicate)
included	included	*Nyctibius*	*griseus*	Nyctibiidae	HM746792		could not be verified: no ND2	verified	verified	no	Pacheco et al 2011			2011	2011	Phylogram	(duplicate)
included	excluded: no paper (yet)	*Nycticorax*	*nycticorax*	Ardeidae	JN018412	NC_015807	verified	verified	verified	no	Zhang, L.-Q. and Kan, X.-Z.	2011	?	unpubl	2011	-	
included	included	*Nycticorax*	*nycticorax*	Ardeidae	KJ190954		verified	verified	verified	no	Zhou et al 2014b			2014	2014	Phylogram	(duplicate)
included	included	*Nyctyornis*	*amictus*	Meropidae	MK060139		verified	verified	could not be verified: no cyt b	no	Tamashiro et al 2019			2019	2019	Phylogram	(duplicate)
included	included	*Nymphicus*	*hollandicus*	Cacatuidae	HM640215	NC_015192	verified	verified	verified	no	Pacheco et al 2011			2011	2011	Phylogram	(duplicate)
included	included	*Nymphicus*	*hollandicus*	Cacatuidae	MH133968		verified	verified	verified	no	Urantowka et al 2018			2018	2018	Cladogram	(duplicate)
included	included	*Oedistoma*	*iliolophum*	Melanocharitidae	KJ909186	NC_024865	could not be verified: no ND2	could not be verified: no COI	could not be verified: no cyt b	?	Barker 2014			2014	2014	Cladogram	(duplicate)
included	included	*Oenanthe*	*isabellina*	Muscicapidae	KU097327	NC_040290	verified	verified	verified	no	Li et al 2016g			2016	2016	Cladogram	*Cyanoptila cyanomelana, Periparus ater*
included	excluded: no paper (yet)	*Oporornis*	*tolmiei*	Parulidae	FJ236286		verified	verified	verified	no	Carson, R.J. and Spicer, G.S.	2008	?	unpubl	2008	-	
included	included	*Oreomystis*	*bairdi*	Fringillidae	KM078807	NC_025628	could not be verified: no ND2	could not be verified: no structure	verified	no	Lerner et al 2011			2011	2011	Cladogram	(duplicate)
included	included	*Oreopsar*	*bolivianus*	Icteridae	JX516058	NC_018797	verified	could not be verified: no COI	verified	no	Powell et al 2013			2013	2013	Cladogram	(duplicate)

Sequence database	Phylogeny database	Genus	species	family	GenBank#	RefSeq#	ND2 verified	COI verified	cyt b verified	erroneous sequence?	reference	submitted to GenBank	sequence released on GenBank	publication of paper	YEAR	Phylogeny (mitogenomic)	problematic sequences in tree
included	excluded: no paper (yet)	*Oreotrochilus*	*melanogaster*	Trochilidae	KJ619587	NC_027454	verified	could not be verified: no COI	could not be verified: no cyt b	no	Magarinos Souto, H., A McGuire, J., Mudge, J., Witt, C., Howard, J., Jarvis, E., Bryan Jennings, W. and Prosdocimi, F.	2014	?	unpubl	2014	-	
included	excluded: no paper (yet)	*Oriolus*	*chinensis*	Oriolidae	JQ083495	NC_020424	verified	verified	verified	no	Kan, X.-Z. and Qian, C.-J.	2011	?	unpubl	2011	-	
included	included	*Orthopsittaca*	*manilata*	Psittacidae	KJ579139	NC_029161	verified	verified	verified	no	Urantowka 2016c			2016	2016	no	
included	included	*Otidiphaps*	*nobilis*	Columbidae	MG590265	NC_036612	verified	verified	verified	no	Bruxaux et al 2018			2018	2018	Phylogram	(duplicate)
included	included	*Otidiphaps*	*nobilis*	Columbidae	KX902241		verified	verified	verified	no	Soares et al 2016			2016	2016	Cladogram	(duplicate)
included	included	*Otis*	*tarda*	Otididae	FJ751803	NC_014046	could not be verified: no ND2	verified	verified	no	Yang et al 2010b			2010	2010	no	
included	included	*Otus*	*bakkamoena*	Strigidae	KT340631	NC_028163	misidentified	misidentified	misidentified	yes (chimera)	Park et al 2019b			2019	2019	Phylogram	*Otus scops*, *Otus bakkamoena*, *Strix leptogrammica*, *Ninox strenua*
included	included	*Otus*	*scops*	Strigidae	KY471456		old taxonomy	old taxonomy	old taxonomy	yes (taxonomy)	Liu et al 2019a			2019	2019	Cladogram	(duplicate)
included	included	*Otus*	*scops*	Strigidae	KT340630	NC_028162	old taxonomy	old taxonomy	old taxonomy	yes (taxonomy)	Park et al 2019a			2019	2019	Phylogram	*Otus scops*, *Strix leptogrammica*, *Ninox strenua*
included	included	*Otus*	*sunia*	Strigidae	MF346692	NC_041422	verified	verified	verified	no	Zhou et al 2019a			2019	2019	Phylogram	*Tyto longimembris*, *Otus scops*, *Strix leptogrammica*, *Ninox strenua*
included	included	*Pachycephala*	*melanura*	Pachycephalidae	KY994577		verified	verified	could not be verified: no cyt b	no	Lamb et al 2018			2018	2018	Phylogram	(duplicate)
included	included	*Pachycephala*	*melanura*	Pachycephalidae	KY994595		verified	verified	could not be verified: no cyt b	no	Lamb et al 2018			2018	2018	Phylogram	(duplicate)
included	included	*Pachycephala*	*melanura*	Pachycephalidae	KY994607		verified	verified	could not be verified: no cyt b	no	Lamb et al 2018			2018	2018	Phylogram	(duplicate)
included	included	*Pachycephala*	*melanura*	Pachycephalidae	KY994610		verified	verified	could not be verified: no cyt b	no	Lamb et al 2018			2018	2018	Phylogram	(duplicate)
included	included	*Pachycephala*	*melanura*	Pachycephalidae	KY994612		verified	verified	could not be verified: no cyt b	no	Lamb et al 2018			2018	2018	Phylogram	(duplicate)
included	included	*Pachycephala*	*occidentalis*	Pachycephalidae	KY994609		verified	verified	could not be verified: no cyt b	no	Lamb et al 2018			2018	2018	Phylogram	(duplicate)
included	included	*Pachycephala*	*pectoralis*	Pachycephalidae	KY994581		verified	verified	verified	no	Lamb et al 2018			2018	2018	Phylogram	(duplicate)
included	included	*Pachycephala*	*pectoralis*	Pachycephalidae	KY994600		verified	verified	verified	no	Lamb et al 2018			2018	2018	Phylogram	(duplicate)

Sequence database	Phylogeny database	Genus	species	family	GenBank#	RefSeq#	ND2 verified	COI verified	cyt b verified	erroneous sequence?	reference	submitted to GenBank	sequence released on GenBank	publication of paper	YEAR	Phylogeny (mitogenomic)	problematic sequences in tree
included	included	*Pachyplichas*	*yaldwyni*	Acanthisittidae	KX369036		could not be verified: no ND2	could not be verified: no COI	could not be verified: no cyt b	?	Mitchell et al 2016			2016	2016	Cladogram	(duplicate)
included	included	*Padda*	*oryzivora*	Estrildidae	KT633398	NC_028441	verified	verified	verified	no	Huang & Zeng 2016b			2016	2016	Phylogram	-
included	included	*Pandion*	*haliaetus*	Accipitridae	DQ780884	NC_008550	verified	verified	verified	no	Gibb et al 2007			2007	2007	Phylogram	(duplicate)
included	included	*Paradoxornis*	*fulvifrons*	Timaliidae	KT598466	NC_028436	verified	could not be verified: no COI	verified	no	Wen et al 2017a			2017	2017	Phylogram	*Cyanoptila cyanomelana, Sturnus nigricollis*
included	excluded: no paper (yet)	*Paradoxornis*	*nipalensis*	Timaliidae	KT598467	NC_028437	verified	verified	verified	no	Wen, L., Yang, X., Liao, J., Fu, Y. and Dai, B.	2015	2015	unpubl	2015	-	
included	included	*Paradoxornis*	*webbianus*	Timaliidae	KF725775	NC_024539	verified	verified	verified	no	Zhang et al 2015d			2015	2015	no	
included	included	*Paradoxornis (Psittiparus)*	*gularis*	Timaliidae	MK900637		verified	verified	verified	no	He et al 2019d		2019	2019	2019	Phylogram	-
included	included	*Paradoxornis (Psittiparus)*	*gularis*	Timaliidae	KX397391	NC_039536	verified	verified	verified	no	Zhou et al 2019b			2019	2019	Phylogram	(duplicate)
included	included	*Pardalotus*	*punctatus*	Pardalotidae	KY994592		verified	verified	verified	no	Lamb et al 2018			2018	2018	Phylogram	(duplicate)
included	included	*Pardalotus*	*striatus*	Pardalotidae	KY994585		verified	could not be verified: no COI	verified	no	Lamb et al 2018			2018	2018	Phylogram	(duplicate)
included	included	*Pardalotus*	*striatus*	Pardalotidae	KY994602		verified	could not be verified: no COI	verified	no	Lamb et al 2018			2018	2018	Phylogram	(duplicate)
included	included	*Paroreomyza*	*montana*	Fringillidae	KM078771	NC_025601	verified	could not be verified: no structure	verified	no	Lerner et al 2011			2011	2011	Cladogram	(duplicate)
included	included	*Parus*	*major*	Paridae	MH638304	NC_040875	verified	verified	verified	no	Laine et al 2019			2019	2019	no	
included	included	*Parus*	*major*	Paridae	KX388473		verified	verified	verified	no	Li et al 2016b (Li et al 2017)			2016	2016	Phylogram	(duplicate)
included	included	*Parus*	*major*	Paridae	KX388480		verified	verified	verified	no	Li et al 2016b (Li et al 2017)			2016	2016	Phylogram	(duplicate)
included	included	*Parus*	*major*	Paridae	KP137624	NC_026293	verified	verified	verified	no	Meng et al 2016b			2016	2016	no	
included	included	*Parus*	*monticolus*	Paridae	KX388474		verified	verified	verified	no	Li et al 2016b (Li et al 2017)			2016	2016	Phylogram	(duplicate)
included	included	*Parus*	*monticolus*	Paridae	KX388481		verified	verified	verified	no	Li et al 2016b (Li et al 2017)			2016	2016	Phylogram	(duplicate)
included	included	*Parus*	*monticolus*	Paridae	KT373848	NC_028187	verified	verified	verified	no	Wen et al 2017b			2017	2017	Phylogram	*Periparus ater, Cyanoptila cyanomelana, Sturnus nigricollis*
included	included	*Parus*	*venustulus*	Paridae	KP313823	NC_026701	verified	verified	verified	no	Li et al 2016c			2016	2016	no	
included	included	*Passer*	*ammodendri*	Passeridae	KT895996		misidentified	misidentified	verified	yes (chimera)	Fan et al 2017			2017	2017	Cladogram	*Passer ammodendri*
included	excluded: no paper (yet)	*Passer*	*domesticus*	Passeridae	CM004555		verified	verified	verified	no	Elgvin, T.O., Trier, C.N., Toerresen, O.K., Lien, S., Jensen, H., Saetre, G.-P.	2014	?	unpubl	2014	-	
included	included	*Passer*	*domesticus*	Passeridae	KM078784	NC_025611	verified	verified	verified	no	Lerner et al 2011			2011	2011	Cladogram	(duplicate)

Sequence database	Phylogeny database	Genus	species	family	GenBank#	RefSeq#	ND2 verified	COI verified	cyt b verified	erroneous sequence?	reference	submitted to GenBank	sequence released on GenBank	publication of paper	YEAR	Phylogeny (mitogenomic)	problematic sequences in tree
included	excluded: no paper (yet)	*Passer*	*montanus*	Passeridae	MH211396		verified	verified	verified	no	Huang, Z. and Tu, F.	2018	2019	unpubl	2019	-	
included	excluded: no paper (yet)	*Passer*	*montanus*	Passeridae	MH211397		verified	verified	verified	no	Huang, Z. and Tu, F.	2018	2019	unpubl	2019	-	
included	excluded: no paper (yet)	*Passer*	*montanus*	Passeridae	MH211398		verified	verified	verified	no	Huang, Z. and Tu, F.	2018	2019	unpubl	2019	-	
included	excluded: no paper (yet)	*Passer*	*montanus*	Passeridae	MH211399		misidentified	misidentified	misidentified	yes (misID)	Huang, Z. and Tu, F.	2018	2019	unpubl	2019	-	
included	excluded: no paper (yet)	*Passer*	*montanus*	Passeridae	JX486030	NC_024821	verified	verified	verified	no	Kan, X.-Z. and Qian, C.-J.	2012	?	unpubl	2012	-	
included	included	*Passer*	*montanus saturatus*	Passeridae	KM577704		verified	verified	verified	no	Yang et al 2016e			2016	2016	no	
included	excluded: no paper (yet)	*Passerina*	*amoenis*	Cardinalidae	FJ236297		verified	verified	verified	no	Carson, R.J. and Spicer, G.S.	2008	?	unpubl	2008	-	
included	included	*Patagioenas*	*fasciata*	Columbidae	KX902239		verified	verified	verified	no	Soares et al 2016			2016	2016	Cladogram	(duplicate)
included	included	*Patagioenas*	*fasciata*	Columbidae	KX902240		verified	verified	verified	no	Soares et al 2016			2016	2016	Cladogram	(duplicate)
included	included	*Pavo*	*cristatus*	Phasianidae	KF444060	NC_024533	verified	verified	verified	no	Zhou et al 2015b			2015	2015	Cladogram	*Phasianus versicolor, Francolinus pintadeanus*
included	included	*Pavo*	*muticus*	Phasianidae	EU417811	NC_012897	verified	could not be verified: no COI	verified	no	Shen et al 2009			2009	2009	Cladogram	(duplicate)
included	included	*Pelagodroma*	*marina*	Oceanitidae	KC875856		verified	verified	verified	no	Gibb et al 2013			2013	2013	Phylogram	(duplicate)
included	included	*Pelecanus*	*conspicillatus*	Pelecanidae	DQ780883		verified	verified	could not be verified: no cyt b	no	Gibb et al 2007			2007	2007	Phylogram	(duplicate)
included	included	*Pelecanus*	*crispus*	Pelecanidae	MK855120		verified	verified	could not be verified: no cyt b	no	Huang et al 2019b		2019	2019	2019	Cladogram	-
included	included	*Pelecanus*	*occidentalis*	Pelecanidae	MH041272		verified	verified	verified	no	Huang et al 2018			2018	2018	Phylogram	-
included	included	*Penelopides*	*panini*	Bucerotidae	HQ834451	NC_015087	could not be verified: no ND2	verified	verified	no	Sammler et al 2011			2011	2011	no	
included	included	*Perdix*	*dauurica*	Phasianidae	FJ752431	NC_020588	verified	verified	verified	no	Shen et al 2010			2010	2010	Phylogram	(duplicate)
included	included	*Perdix*	*dauurica*	Phasianidae	KY411596		verified	verified	verified	no	Wang et al 2017a			2017	2017	Phylogram	(duplicate)
included	excluded: no paper (yet)	*Perdix*	*hodgsoniae*	Phasianidae	KX838507		verified	could not be verified: no COI	verified	no	Li, X.-J., Huang, Y. and Lei, F.-M.	2016	2018	unpubl	2018	-	
included	included	*Perdix*	*hodgsoniae*	Phasianidae	KF027440	NC_023940	verified	could not be verified: no COI	verified	no	Zhou et al 2014a			2014	2014	Cladogram	*Phasianus versicolor*
included	excluded: no paper (yet)	*Perdix*	*perdix*	Phasianidae	KX838508	NC_039843	verified	verified	verified	no	Li, X.-J., Huang, Y. and Lei, F.-M.	2016	2018	unpubl	2018	-	
included	excluded: no paper (yet)	*Pericrocotus*	*ethologus*	Pericrocotidae	JX256246	NC_024257	verified	could not be verified: no COI	verified	no	Qian, C.-J. and Kan, X.-Z.	2012	?	unpubl	2012	-	

Sequence database	Phylogeny database	Genus	species	family	GenBank#	RefSeq#	ND2 verified	COI verified	cyt b verified	erroneous sequence?	reference	submitted to GenBank	sequence released on GenBank	publication of paper	YEAR	Phylogeny (mitogenomic)	problematic sequences in tree
included	included	*Periparus*	*ater*	Paridae	KM588075	NC_026223	misidentified	problematic	misidentified	yes (misID)	Zhang et al 2016g			2016	2016	no	
included	included	*Pernis*	*ptilorhynchus*	Accipitridae	MK043029		could not be verified: no ND2	verified	verified	no	Kim et al 2019a			2019	2019	Phylogram	(duplicate)
included	included	*Petroica*	*australis*	Petroicidae	KC545401	NC_029141	verified	verified	verified	no	Gibb et al 2015			2015	2015	Phylogram	(duplicate)
included	included	*Petroica*	*boodang*	Petroicidae	JX901074	NC_019666	verified	verified	could not be verified: no cyt b	no	Cooke et al 2012			2012	2012	no	
included	included	*Petroica*	*goodenovii*	Petroicidae	JX901075	NC_019667	verified	verified	could not be verified: no cyt b	no	Cooke et al 2012			2012	2012	no	
included	included	*Petroica*	*macrocephala*	Petroicidae	KC545402	NC_029142	verified	verified	verified	no	Gibb et al 2015			2015	2015	Phylogram	(duplicate)
included	included	*Petroica*	*phoenicea*	Petroicidae	JX901076	NC_019668	verified	verified	could not be verified: no cyt b	no	Cooke et al 2012			2012	2012	no	
included	included	*Petronia*	*petronia*	Passeridae	MF071218		verified	verified	verified	no	Shi et al 2017a			2017	2017	Cladogram	*Passer ammodendri, Emberiza aureola*
included	included	*Phaethon*	*lepturus*	Phaethontidae	KR349465	NC_027275	verified	verified	verified	no	Wang et al 2016e			2016	2016	Phylogram	-
included	included	*Phaethon*	*rubricauda*	Phaethontidae	AP009043	NC_007979	verified	verified	verified	no	Yamamoto et al 2005b	2005	?	2005	2005	no	
included	excluded: no paper (yet)	*Phaethornis*	*hispidus*	Trochilidae	KP853098		verified	could not be verified: no COI	verified	no	Costa, I.R., Souto, H.M., Furtado, C., Mudge, J., McGuire, J., Witt, C., Jennings, B., Ruschi, P. and Prosdocimi, F.	2015	2016	unpubl	2016	-	
included	excluded: no paper (yet)	*Phaethornis*	*malaris*	Trochilidae	KP853097	NC_030288	misidentified	misidentified	could not be verified: no cyt b	yes (misID)	Costa, I.R., Souto, H.M., Furtado, C., Mudge, J., McGuire, J., Witt, C., Jennings, B., Ruschi, P. and Prosdocimi, F.	2015	2016	unpubl	2016	-	
included	included	*Phalacrocorax*	*brasilianus*	Phalacrocoracidae	KT626611	NC_029758	verified	verified	verified	no	Rodrigues et al 2017			2017	2017	Phylogram	-
included	included	*Phalacrocorax*	*carbo*	Phalacrocoracidae	KR215630	NC_027267	verified	verified	verified	no	Zhang et al 2017c			2017	2017	Cladogram	-
included	included	*Phalacrocorax*	*chalconotus*	Phalacrocoracidae	GU071054		verified	could not be verified: no structure	could not be verified: no cyt b	no	Gibb et al 2013			2013	2013	Phylogram	(duplicate)
included	included	*Phalaropus*	*lobatus*	Scolopacidae	KY765409		verified	verified	verified	no	Liu et al 2018b			2018	2018	Cladogram	-
included	excluded: no paper (yet)	*Phalcoboenus (Daptrius)*	*australis*	Falconidae	KP064202	NC_031897	verified	could not be verified: no COI	dubious	dubious	Mahmood, M.T., McLenachan, P.A., Zhong, B., Wink, M. and Penny, D	2014	?	unpubl	2014	-	
included	included	*Pharomachrus*	*auriceps*	Trogonidae	MK060140		verified	could not be verified: no COI	verified	no	Tamashiro et al 2019			2019	2019	Phylogram	(duplicate)
included	included	*Phasianus*	*colchicus*	Phasianidae	JF739859	NC_015526	verified	verified	verified	no	Li et al 2013			2013	2013	no	
included	included	*Phasianus*	*colchicus*	Phasianidae	FJ752430		verified	verified	verified	no	Shen et al 2010			2010	2010	Phylogram	(duplicate)

Sequence database	Phylogeny database	Genus	species	family	GenBank#	RefSeq#	ND2 verified	COI verified	cyt b verified	erroneous sequence?	reference	submitted to GenBank	sequence released on GenBank	publication of paper	YEAR	Phylogeny (mitogenomic)	problematic sequences in tree
included	excluded: no paper (yet)	*Phasianus*	*colchicus*	Phasianidae	KJ502237		verified	verified	verified	no	Wu, Q., Yang, F. and Xing, X.	2014	?	unpubl	2014	-	
included	excluded: no paper (yet)	*Phasianus*	*colchicus*	Phasianidae	KT364526		verified	verified	verified	no	Zhang, Y.F., Xie, Z.X., Liu, J.B., Deng, X.W., Xie, Z.Q., Huang, L., Huang, J.L., Zeng, T.T. and Wang, S.	2015	2015	unpubl	2015	-	
included	included	*Phasianus*	*colchicus*	Phasianidae	KX512321		misidentified	misidentified	misidentified	yes (mislabelled)	Zhu et al 2017			2017	2017	Cladogram	*Phasianus colchicus*
included	included	*Phasianus*	*colchicus alaschanicus*	Phasianidae	KU049722		verified	verified	verified	no	Zhao et al 2017			2017	2017	Phylogram	*Phasianus versicolor, Francolinus pintadeanus*
included	included	*Phasianus*	*colchicus kiangsuensis*	Phasianidae	KP637175		verified	verified	verified	no	Wu et al 2016a			2016	2016	Phylogram	*Phasianus versicolor*
included	excluded: no paper (yet)	*Phasianus*	*versicolor*	Phasianidae	AB164626	NC_010778	could not be verified: no ND2	verified	problematic	yes (chimera)	Kato, S., Nishibori, M. and Yasue, H.	2004		unpubl	2004	-	
included	excluded: no paper (yet)	*Pheucticus*	*melanocephalus*	Cardinalidae	FJ236298		verified	verified	verified	no	Carson, R.J. and Spicer, G.S.	2008	?	unpubl	2008	-	
included	included	*Philesturnus (Creadion)*	*carunculatus*	Callaeatidae	KC545403	NC_029143	verified	verified	verified	no	Gibb et al 2015			2015	2015	Phylogram	(duplicate)
included	included	*Phodilus*	*badius*	Tytonidae	KF961183	NC_023787	verified	could not be verified: no COI	verified	no	Mahmood et al 2014			2014	2014	Phylogram	(duplicate)
included	included	*Phoebastria*	*albatrus*	Diomedeidae	KJ735514	NC_026190	could not be verified: no ND2	verified	verified	no	Lounsberry et al 2015			2015	2015	no	
included	included	*Phoebastria*	*immutabilis*	Diomedeidae	KJ735513	NC_026189	could not be verified: no ND2	verified	verified	no	Lounsberry et al 2015			2015	2015	no	
included	included	*Phoebastria*	*nigripes*	Diomedeidae	KJ735512	NC_026188	verified	verified	verified	no	Lounsberry et al 2015			2015	2015	no	
included	included	*Phoenicopterus*	*roseus*	Phoenicopteridae	EF532932	NC_010089	could not be verified: no ND2	verified	verified	no	Morgan-Richards et al 2008			2008	2008	Phylogram	(duplicate)
included	included	*Phoenicopterus*	*ruber*	Phoenicopteridae	KT159835	NC_027934	verified	verified	verified	no	Luo et al 2016			2016	2016	Phylogram	*Anas falcata, Caprimulgus jotaka*
included	included	*Phoeniculus*	*purpureus*	Phoeniculidae	MK060141		could not be verified: too few ND2	could not be verified: no COI	verified	no	Tamashiro et al 2019			2019	2019	Phylogram	(duplicate)
included	included	*Phoenicurus*	*auroreus*	Muscicapidae	KF997863	NC_026066	verified	verified	verified	no	Zhang et al 2018b			2018	2018	Phylogram	*Lanius tephronotus, Cyanoptila cyanomelana, Pseudopodoces humilis, Garrulax perspicillatus, Emberiza chrysophrys*

Sequence database	Phylogeny database	Genus	species	family	GenBank#	RefSeq#	ND2 verified	COI verified	cyt b verified	erroneous sequence?	reference	submitted to GenBank	sequence released on GenBank	publication of paper	YEAR	Phylogeny (mitogenomic)	problematic sequences in tree
included	included	*Phylloscopus*	*occisinensis*	Phylloscopidae	MK513447		could not be verified: no ND2	verified	verified	no	Liu et al 2019m		2019	2019	2019	Cladogram	*Periparus ater, Poecile palustris, Garrulax milnei,*
included	included	*Phylloscopus (Abrornis)*	*inornatus*	Phylloscopidae	KF742677	NC_024726	could not be verified: no ND2	verified	verified	no	Qing et al 2015			2015	2015	no	
included	included	*Phylloscopus (Abrornis)*	*proregulus*	Phylloscopidae	MG189603	NC_037189	could not be verified: no ND2	verified	verified	no	Jiao et al 2018			2018	2018	Phylogram	*Cyanoptila cyanomelana*
included	excluded: no paper (yet)	*Pica*	*pica*	Corvidae	HQ915867	NC_015200	verified	verified	verified	no	Kan, X.-Z. and Chen, L.	2011	?	unpubl	2011	-	
included	included	*Picathartes*	*gymnocephalus*	Picathartidae	KJ909200		verified	could not be verified: no COI	verified	no	Barker 2014			2014	2014	Cladogram	(duplicate)
included	included	*Picoides*	*pubescens*	Picidae	KT119343	NC_027936	verified	verified	verified	no	Zhang et al 2016h			2016	2016	Phylogram	-
included	included	*Picumnus*	*innominatus*	Picidae	KX831678	NC_039537	verified	could not be verified: no COI	verified	no	Zhou et al 2017			2017	2017	no	
included	included	*Picus*	*canus*	Picidae	MK348064	NC_045372	verified	verified	verified	no	Yao et al 2019		2019	2019	2019	Cladogram	-
included	included	*Pinguinus*	*impennis*	Alcidae	KU158188	NC_031347	could not be verified: no ND2	could not be verified: no COI	verified	no	Anmarkrud & Lifjeld 2017			2017	2017	no	
included	included	*Pinguinus*	*impennis*	Alcidae	MF188883		could not be verified: no ND2	could not be verified: no COI	verified	no	Thomas et al 2017			2017	2017	network	-
included	included	*Pinguinus*	*impennis*	Alcidae	MF188884		could not be verified: no ND2	could not be verified: no COI	verified	no	Thomas et al 2017			2017	2017	network	(duplicate)
included	included	*Pinguinus*	*impennis*	Alcidae	MF188885		could not be verified: no ND2	could not be verified: no COI	verified	no	Thomas et al 2017			2017	2017	network	(duplicate)
included	included	*Pinguinus*	*impennis*	Alcidae	MF188886		could not be verified: no ND2	could not be verified: no COI	verified	no	Thomas et al 2017			2017	2017	network	(duplicate)
included	included	*Pinguinus*	*impennis*	Alcidae	MF188887		could not be verified: no ND2	could not be verified: no COI	verified	no	Thomas et al 2017			2017	2017	network	(duplicate)
included	included	*Pinguinus*	*impennis*	Alcidae	MF188888		could not be verified: no ND2	could not be verified: no COI	verified	no	Thomas et al 2017			2017	2017	network	(duplicate)
included	included	*Pinguinus*	*impennis*	Alcidae	MF188889		could not be verified: no ND2	could not be verified: no COI	verified	no	Thomas et al 2017			2017	2017	network	(duplicate)
included	included	*Pinicola*	*enucleator*	Fringillidae	KM078781	NC_025609	verified	verified	verified	no	Lerner et al 2011			2011	2011	Cladogram	(duplicate)
included	included	*Pionites*	*leucogaster*	Psittacidae	MK759905	NC_044184	verified	could not be verified: no COI	verified	no	Liu et al 2019j		2019	2019	2019	Phylogram	(duplicate)
included	included	*Pionus*	*chalcopterus*	Psittacidae	MF784450		could not be verified: no structure	verified	verified	no	Urantowka et al 2017f			2018	2018	no	
included	included	*Pionus*	*menstruus*	Psittacidae	KX925978		verified	verified	verified	no	Urantowka & Mackiewicz 2016a			2016	2016	no	

Sequence database	Phylogeny database	Genus	species	family	GenBank#	RefSeq#	ND2 verified	COI verified	cyt b verified	erroneous sequence?	reference	submitted to GenBank	sequence released on GenBank	publication of paper	YEAR	Phylogeny (mitogenomic)	problematic sequences in tree
included	included	*Pipile*	*pipile*	Cracidae	KU221051		could not be verified: no ND2	could not be verified: no structure	could not be verified: no cyt b	?	Grass et al 2016			2016	2016	Phylogram	*Francolinus pintadeanus*
included	included	*Pipile*	*pipile*	Cracidae	KU221052		could not be verified: no ND2	could not be verified: no structure	could not be verified: no cyt b	?	Grass et al 2016			2016	2016	Phylogram	(duplicate)
included	included	*Pipile*	*pipile*	Cracidae	KU221053		could not be verified: no ND2	could not be verified: no structure	could not be verified: no cyt b	?	Grass et al 2016			2016	2016	Phylogram	(duplicate)
included	excluded: no paper (yet)	*Pipilo*	*maculatus*	Passerellidae	FJ236291		could not be verified: no structure	verified	could not be verified: no structure	no	Carson, R.J. and Spicer, G.S.	2008	?	unpubl	2008	-	
included	included	*Piranga*	*bidentata*	Thraupidae	MH700646		verified	verified	verified	no	Campillo et al 2019	2018		2019	2019	Phylogram	(duplicate)
included	included	*Piranga*	*bidentata*	Thraupidae	MH700647	NC_041665	verified	verified	verified	no	Campillo et al 2019	2018		2019	2019	Phylogram	(duplicate)
included	included	*Piranga*	*erythrocephala*	Thraupidae	MH700648		verified	could not be verified: no COI	verified	no	Campillo et al 2019	2018		2019	2019	Phylogram	(duplicate)
included	included	*Piranga*	*erythrocephala*	Thraupidae	MH700649	NC_041666	verified	could not be verified: no COI	verified	no	Campillo et al 2019	2018		2019	2019	Phylogram	(duplicate)
included	included	*Piranga*	*flava*	Thraupidae	MH700637	NC_041661	dubious	verified	verified	?	Campillo et al 2019	2018		2019	2019	Phylogram	(duplicate)
included	included	*Piranga*	*flava*	Thraupidae	MH700638		dubious	verified	verified	?	Campillo et al 2019	2018		2019	2019	Phylogram	(duplicate)
included	included	*Piranga*	*hepatica*	Thraupidae	MH700640	NC_041662	could not be verified: no ND2	could not be verified: no COI	could not be verified: no cyt b	?	Campillo et al 2019	2018		2019	2019	Phylogram	(duplicate)
included	included	*Piranga*	*hepatica*	Thraupidae	MH700641		could not be verified: no ND2	could not be verified: no COI	could not be verified: no cyt b	?	Campillo et al 2019	2018		2019	2019	Phylogram	(duplicate)
included	included	*Piranga*	*leucoptera*	Thraupidae	MH700652	NC_041668	verified	verified	verified	no	Campillo et al 2019	2018		2019	2019	Phylogram	(duplicate)
included	included	*Piranga*	*leucoptera*	Thraupidae	MH700653		verified	verified	verified	no	Campillo et al 2019	2018		2019	2019	Phylogram	(duplicate)
included	included	*Piranga*	*ludoviciana*	Thraupidae	MH700644	NC_041664	verified	verified	verified	no	Campillo et al 2019	2018		2019	2019	Phylogram	(duplicate)
included	included	*Piranga*	*ludoviciana*	Thraupidae	MH700645		verified	verified	verified	no	Campillo et al 2019	2018		2019	2019	Phylogram	(duplicate)
included	excluded: no paper (yet)	*Piranga*	*ludoviciana*	Thraupidae	FJ236296		could not be verified: no structure	verified	verified	no	Carson, R.J. and Spicer, G.S.	2008	?	unpubl	2008	-	
included	included	*Piranga*	*lutea*	Thraupidae	MH700636	NC_041660	could not be verified: no ND2	could not be verified: no COI	could not be verified: no cyt b	?	Campillo et al 2019	2018		2019	2019	Phylogram	(duplicate)
included	included	*Piranga*	*lutea*	Thraupidae	MH700639		could not be verified: no ND2	could not be verified: no COI	could not be verified: no cyt b	?	Campillo et al 2019	2018		2019	2019	Phylogram	(duplicate)
included	included	*Piranga*	*olivacea*	Thraupidae	MH700642	NC_041663	verified	verified	verified	no	Campillo et al 2019	2018		2019	2019	Phylogram	(duplicate)
included	included	*Piranga*	*olivacea*	Thraupidae	MH700643		verified	verified	verified	no	Campillo et al 2019	2018		2019	2019	Phylogram	(duplicate)

Sequence database	Phylogeny database	Genus	species	family	GenBank#	RefSeq#	ND2 verified	COI verified	cyt b verified	erroneous sequence?	reference	submitted to GenBank	sequence released on GenBank	publication of paper	YEAR	Phylogeny (mitogenomic)	problematic sequences in tree
included	included	*Piranga*	*roseogularis*	Thraupidae	MH700632	NC_041658	could not be verified: no ND2	could not be verified: no COI	verified	no	Campillo et al 2019	2018		2019	2019	Phylogram	(duplicate)
included	included	*Piranga*	*roseogularis*	Thraupidae	MH700633		could not be verified: no ND2	could not be verified: no COI	verified	no	Campillo et al 2019	2018		2019	2019	Phylogram	(duplicate)
included	included	*Piranga*	*rubra*	Thraupidae	MH700634	NC_041659	verified	verified	verified	no	Campillo et al 2019	2018		2019	2019	Phylogram	(duplicate)
included	included	*Piranga*	*rubra*	Thraupidae	MH700635		verified	verified	verified	no	Campillo et al 2019	2018		2019	2019	Phylogram	(duplicate)
included	included	*Piranga*	*rubriceps*	Thraupidae	MH700650	NC_041667	verified	could not be verified: no COI	could not be verified: no cyt b	no	Campillo et al 2019	2018		2019	2019	Phylogram	(duplicate)
included	included	*Piranga*	*rubriceps*	Thraupidae	MH700651		verified	could not be verified: no COI	could not be verified: no cyt b	no	Campillo et al 2019	2018		2019	2019	Phylogram	(duplicate)
included	excluded: no paper (yet)	*Pitta*	*nympha*	Pittidae	KJ680302		could not be verified: no ND2	verified	verified	no	Lee, Y.J., Ryu, S.H. and Hwang, U.W.	2014	?	unpubl	2014	-	
included	excluded: no paper (yet)	*Platalea*	*leucorodia*	Threskiornithidae	KT901459		verified	verified	verified	no	An, J.	2015	2016	unpubl	2016	-	
included	excluded: no paper (yet)	*Platalea*	*leucorodia*	Threskiornithidae	GQ199608	NC_012772	verified	verified	verified	no	Cheng, Y.-Y., Chang, H.-W., Yao, C.-T., Chiu, C.-C., Hsin, C.-H., Chang, W.-C. and Chou, Y.C	2009	?	unpubl	2009	-	
included	excluded: no paper (yet)	*Platalea*	*minor*	Threskiornithidae	EF455490	NC_010962	verified	could not be verified: no COI	verified	no	Lee, Y.I., Tsai, C.-L., Fang, S.Y. and Chou, Y.C.	2007	?	unpubl	2007	-	
included	included	*Pluvialis*	*fulva*	Charadriidae	KX639757	NC_033966	verified	verified	could not be verified: no cyt b	no	Ding et al 2016b			2016	2016	Phylogram	-
included	included	*Podiceps*	*cristatus*	Podicipedidae	AP009194	NC_008140	could not be verified: no ND2	verified	could not be verified: no cyt b	no	Watanabe et al 2006			2006	2006	Phylogram	(duplicate)
included	included	*Podoces*	*hendersoni*	Corvidae	GU592504	NC_014879	could not be verified: no ND2	verified	verified	no	Ke et al 2010			2010	2010	no	
included	included	*Poecile*	*atricapilla*	Paridae	KJ909190	NC_024867	verified	verified	verified	no	Barker 2014			2014	2014	Cladogram	(duplicate)
included	included	*Poecile*	*montanus*	Paridae	KX388478		verified	verified	verified	no	Li et al 2016b (Li et al 2017)			2016	2016	Phylogram	(duplicate)
included	included	*Poecile*	*montanus baicalensis*	Paridae	KX388479		verified	verified	verified	no	Li et al 2016b (Li et al 2017)			2016	2016	Phylogram	(duplicate)
included	included	*Poecile*	*palustris*	Paridae	KP184518	NC_026911	verified	verified	verified	no	Day et al 2016			2016	2016	no	
included	included	*Poecile*	*palustris*	Paridae	KX388475		misidentified	misidentified	misidentified	yes (misID)	Li et al 2016b (Li et al 2017)			2016	2016	Phylogram	(duplicate)
included	included	*Pogoniulus*	*bilineatus*	Lybiidae	MK060142		could not be verified: no ND2	could not be verified: no COI	verified	no	Tamashiro et al 2019			2019	2019	Phylogram	(duplicate)
included	included	*Poicephalus*	*gulielmi*	Psittacidae	MF977813		could not be verified: no ND2	verified	verified	no	Urantowka et al 2017d			2017	2017	no	

Sequence database	Phylogeny database	Genus	species	family	GenBank#	RefSeq#	ND2 verified	COI verified	cyt b verified	erroneous sequence?	reference	submitted to GenBank	sequence released on GenBank	publication of paper	YEAR	Phylogeny (mitogenomic)	problematic sequences in tree
included	included	*Poicephalus*	*rufiventris*	Psittacidae	MG736916		dubious	verified	could not be verified: no cyt b	no	Sarker et al 2018			2018	2018	Cladogram	*Brotogeris cyanopterus*
included	included	*Poicephalus*	*senegalus*	Psittacidae	MK749396	NC_044083	verified	verified	verified	no	Liu et al 2019j		2019	2019	2019	Phylogram	(duplicate)
included	included	*Polyplectron*	*bicalcaratum*	Phasianidae	EU417812	NC_012900	verified	could not be verified: no COI	verified	no	Shen et al 2009			2009	2009	Cladogram	(duplicate)
included	included	*Polyplectron*	*germaini*	Phasianidae	KF422893	NC_023264	verified	could not be verified: no COI	verified	no	Omeire et al 2015			2015	2015	no	
included	excluded: no paper (yet)	*Polyplectron*	*malacense*	Phasianidae	MN240360	NC_044743	verified	could not be verified: no COI	verified	no	Pineda, L., Caballero, B., McSweeny, T., Brooks, D. and Miranda, H. Jr.		2019	unpubl	2019	-	
included	included	*Polyplectron*	*napoleonis*	Phasianidae	KJ939353	NC_024615	verified	could not be verified: no COI	could not be verified: no cyt b	no	Quach et al 2016			2016	2016	no	
included	included	*Pomatorhinus*	*ruficollis*	Timaliidae	KT970675	NC_029769	verified	verified	verified	no	Zhao et al 2016b			2016	2016	no	
included	included	*Poospiza*	*lateralis*	Thraupidae	KT272190	NC_028039	verified	verified	verified	no	Amaral et al 2015			2015	2015	no	
included	included	*Poospiza*	*thoracica*	Thraupidae	KT272188	NC_028037	could not be verified: no ND2	verified	verified	no	Amaral et al 2015			2015	2015	no	(duplicate)
included	included	*Poospiza (Microspingus)*	*cabanisi*	Thraupidae	KT272189	NC_028038	could not be verified: no ND2	could not be verified: no COI	could not be verified: no cyt b	?	Amaral et al 2015			2015	2015	no	(duplicate)
included	included	*Porphyrio*	*hochstetteri*	Rallidae	EF532934	NC_010092	could not be verified: no ND2	verified	verified	no	Morgan-Richards et al 2008			2008	2008	Phylogram	(duplicate)
included	included	*Porphyrio*	*porphyrio*	Rallidae	KF701062	NC_025508	could not be verified: no ND2	verified	verified	no	Garcia et al 2014			2014	2014	Phylogram	(duplicate)
included	included	*Porzana (Zapornia)*	*fusca*	Rallidae	KY009736		could not be verified: no ND2	verified	verified	no	Chen et al 2017a			2017	2017	Phylogram	*Amaurornis akool, Amaurornis phoenicurus, Coturnicops exquisitus*
included	excluded: paper publ. after 1-1-2020	*Porzana (Zapornia)*	*paykullii*	Rallidae	MG200164	NC_037406	could not be verified: no ND2	verified	verified	no	Chen et al 2020c	2017	2018	2020	2018	Phylogram	(duplicate)
included	included	*Porzana (Zapornia)*	*pusilla*	Rallidae	KY009737		could not be verified: no ND2	verified	verified	no	Chen et al 2017a			2017	2017	Phylogram	(duplicate)
included	included	*Primolius*	*couloni*	Psittacidae	KF836419	NC_025742	verified	verified	could not be verified: no structure	no	Urantowka 2016a			2016	2016	no	
included	included	*Primolius*	*maracana*	Psittacidae	KJ562357	NC_029322	could not be verified: no ND2	verified	could not be verified: no structure	no	Urantowka & Mackiewicz 2017			2017	2017	no	
included	included	*Prioniturus*	*luconensis*	Psittaculidae	KM611473	NC_027846	verified	verified	could not be verified: no cyt b	no	Eberhard & Wright 2016			2016	2016	Cladogram	(duplicate)

Sequence database	Phylogeny database	Genus	species	family	GenBank#	RefSeq#	ND2 verified	COI verified	cyt b verified	erroneous sequence?	reference	submitted to GenBank	sequence released on GenBank	publication of paper	YEAR	Phylogeny (mitogenomic)	problematic sequences in tree
included	included	*Probosciger*	*aterrimus goliath*	Cacatuidae	MH133970		verified	verified	verified	no	Urantowka et al 2018			2018	2018	Cladogram	(duplicate)
included	included	*Procellaria*	*cinerea*	Diomedeidae	AP009191		could not be verified: no ND2	verified	verified	no	Watanabe et al 2006			2006	2006	Phylogram	(duplicate)
included	included	*Prodotiscus*	*insignis*	Indicatoridae	MK060143	NC_039892	could not be verified: no ND2	verified	could not be verified: no cyt b	no	Tamashiro et al 2019			2019	2019	Phylogram	(duplicate)
included	included	*Progne*	*chalybea*	Hirundinidae	JQ071623	NC_020605	could not be verified: no structure	dubious	verified	?	Cerasale et al 2012			2012	2012	Phylogram	-
included	included	*Prosthemadera*	*novaeseelandiae*	Meliphagidae	KC545404	NC_029144	verified	verified	verified	no	Gibb et al 2015			2015	2015	Phylogram	(duplicate)
included	included	*Prunella*	*fulvescens*	Prunellidae	KY471556	NC_035747	verified	verified	verified	no	Cao et al 2017			2017	2017	Cladogram	*Emberiza aureola*
included	included	*Prunella*	*montanella*	Prunellidae	KR422695	NC_027284	verified	verified	verified	no	Yao et al 2016			2016	2016	Phylogram	-
included	included	*Prunella*	*strophiata*	Prunellidae	KU975800	NC_031819	verified	could not be verified: no COI	verified	no	Sun et al 2016f			2016	2016	Cladogram	*Motacilla lugens*
included	included	*Psephotellus*	*pulcherrimus*	Psittaculidae	KU158195	NC_031358	could not be verified: no ND2	could not be verified: no COI	could not be verified: no cyt b	?	Anmarkrud & Lifjeld 2017			2017	2017	no	
included	included	*Pseudoleistes*	*guirahuro*	Icteridae	JX516071	NC_018809	verified	verified	verified	no	Powell et al 2013			2013	2013	Cladogram	(duplicate)
included	included	*Pseudoleistes*	*virescens*	Icteridae	JX516066	NC_018805	verified	verified	verified	no	Powell et al 2013			2013	2013	Cladogram	(duplicate)
included	included	*Pseudonestor*	*xanthophrys*	Fringillidae	KM078809	NC_025630	could not be verified: no ND2	could not be verified: no structure	verified	no	Lerner et al 2011			2011	2011	Cladogram	(duplicate)
included	included	*Pseudopodoces*	*humilis*	Paridae	KP001174		verified	verified	verified	no	Xin et al 2016			2016	2016	no	
included	included	*Pseudopodoces*	*humilis*	Paridae	HM535648	NC_014341	problematic	misidentified	misidentified	yes (chimera)	Yang et al 2010a			2010	2010	no	
included	included	*Psittacara*	*leucophthalmus*	Psittacidae	KF444466	NC_041257	verified	verified	verified	no	Urantowka & Mackiewicz 2016b			2016	2016	no	
included	included	*Psittacara (Aratinga)*	*acuticaudatus*	Psittacidae	JQ782214	NC_020325	verified	verified	verified	no	Urantowka et al 2013a			2013	2013	no	
included	included	*Psittacara (Aratinga)*	*brevipes*	Psittacidae	KC936100	NC_021764	verified	could not be verified: no structure	verified	no	Urantowka et al 2014a			2014	2014	no	
included	included	*Psittacara (Aratinga)*	*mitrata*	Psittacidae	JX215256		verified	verified	verified	no	Urantowka et al 2016b			2016	2016	no	
included	included	*Psittacara (Aratinga)*	*rubritorquis*	Psittacidae	JX524614	NC_026042	verified	dubious	verified	?	Urantowka et al 2016c			2016	2016	no	
included	included	*Psittacula*	*alexandri*	Psittaculidae	MK986660	NC_045378	verified	could not be verified: no COI	could not be verified: no structure	no	Li & Duan 2019		2019	2019	2019	Cladogram	*Brotogeris cyanopterus*
included	included	*Psittacula*	*derbiana*	Psittaculidae	MK343133	NC_042409	could not be verified: no ND2	could not be verified: no COI	could not be verified: no structure	?	Liu et al 2019c			2019	2019	Phylogram	-
included	included	*Psittacula*	*eupatria*	Psittaculidae	MK343134	NC_042765	verified	verified	verified	no	Liu et al 2019c			2019	2019	Phylogram	(duplicate)
included	included	*Psittacula*	*krameri*	Psittaculidae	MN065674		verified	verified	verified	no	Sarker et al 2019b		2019	2019	2019	Cladogram	*Brotogeris cyanopterus*
included	excluded: paper publ. after 1-1-2020	*Psittacula*	*roseata*	Psittaculidae	MK986661	NC_045379	verified	verified	verified	no	Zhang et al 2020b	2019	2019	2020	2019	Cladogram	-

Sequence database	Phylogeny database	Genus	species	family	GenBank#	RefSeq#	ND2 verified	COI verified	cyt b verified	erroneous sequence?	reference	submitted to GenBank	sequence released on GenBank	publication of paper	YEAR	Phylogeny (mitogenomic)	problematic sequences in tree
included	included	*Psittacus*	*erithacus*	Psittacidae	KM611474	NC_027847	verified	verified	verified	no	Eberhard & Wright 2016			2016	2016	Cladogram	(duplicate)
included	included	*Psittirostra*	*psittacea*	Fringillidae	KU158196	NC_031353	could not be verified: no ND2	could not be verified: no structure	could not be verified: no cyt b	?	Anmarkrud & Lifjeld 2017			2017	2017	no	
included	included	*Psittrichas*	*fulgidus*	Psittaculidae	KM611475	NC_027848	verified	verified	verified	no	Eberhard & Wright 2016			2016	2016	Cladogram	(duplicate)
included	included	*Psophia*	*crepitans*	Rallidae	MK434259		could not be verified: no ND2	could not be verified: no structure	verified	no	Boast et al 2019			2019	2019	Phylogram	*Amaurornis akool, Amaurornis phoenicurus, Coturnicops exquisitus*
included	excluded: no paper (yet)	*Pterocles*	*gutturalis*	Pteroclidae	CM020177		could not be verified: no ND2	could not be verified: no COI	could not be verified: no cyt b	?	Gilbert, M.T.P., Lee, C., Fedrigo, O., Formenti, G., Mountcastle, J., Chow, W., Tracey, A., Howe, K., Pas, A. and Jarvis, E.D.		2019	unpubl	2019	-	
included	included	*Pterocles*	*gutturalis*	Pteroclidae	KX902237		could not be verified: no ND2	could not be verified: no COI	could not be verified: no cyt b	?	Soares et al 2016			2016	2016	Cladogram	(duplicate)
included	included	*Pterocles*	*namaqua*	Pteroclidae	DQ385216 (cyt b), DQ385080 (ND2), DQ385165 (COI)		could not be verified: no ND2	could not be verified: no COI	could not be verified: no cyt b	?	Gibb & Penny 2010			2010	2010	Phylogram	(duplicate)
included	included	*Pterocnemia*	*pennata*	Palaeognathae	AF338709	NC_002783	could not be verified: too few sequences of Palaeognathae	verified	verified	no	Haddrath & Baker 2001			2001	2001	Phylogram	(duplicate)
included	included	*Pterodroma (Lugensa)*	*brevirostris*	Procellariidae	AY158678	NC_007174	could not be verified: no ND2	verified	verified	no	Slack et al 2006			2006	2006	Cladogram	(duplicate)
included	included	*Pteroglossus*	*azara flavirostris*	Ramphastidae	DQ780882	NC_008549	could not be verified: no structure	verified	verified	no	Gibb et al 2007			2007	2007	Phylogram	(duplicate)
included	included	*Ptilopachus*	*petrosus*	Ptilopachidae	KJ914543	NC_024616	verified	could not be verified: no COI	verified	no	Meiklejohn et al 2014			2014	2014	Phylogram	(duplicate)
included	included	*Ptilotula (Lichenostomus)*	*penicillata*	Meliphagidae	KY994588		verified	could not be verified: no COI	verified	no	Lamb et al 2018			2018	2018	Phylogram	(duplicate)
included	included	*Ptilotula (Lichenostomus)*	*penicillata*	Meliphagidae	KY994591		verified	could not be verified: no COI	verified	no	Lamb et al 2018			2018	2018	Phylogram	(duplicate)
included	included	*Ptychoramphus*	*aleuticus*	Alcidae	MH382811		verified	verified	verified	no	Liu et al 2018c			2018	2018	Phylogram	-
included	included	*Pucrasia*	*macrolopha*	Phasianidae	FJ752429	NC_020587	verified	verified	verified	no	Shen et al 2010			2010	2010	Phylogram	(duplicate)
included	included	*Puffinus*	*lherminieri*	Procellariidae	MH206162		could not be verified: no ND2	verified	verified	no	Torres et al 2019			2019	2019	no	

Sequence database	Phylogeny database	Genus	species	family	GenBank#	RefSeq#	ND2 verified	COI verified	cyt b verified	erroneous sequence?	reference	submitted to GenBank	sequence released on GenBank	publication of paper	YEAR	Phylogeny (mitogenomic)	problematic sequences in tree
included	included	*Puffinus*	*lherminieri*	Procellariidae	MH206163		could not be verified: no ND2	verified	verified	no	Torres et al 2019			2019	2019	no	
included	included	*Pycnonotus*	*melanicterus*	Pycnonotidae	KJ186975	NC_024730	could not be verified: no ND2	could not be verified: no COI	could not be verified: no cyt b	?	Ren et al 2016b			2016	2016	no	
included	excluded: no paper (yet)	*Pycnonotus*	*sinensis*	Pycnonotidae	GU475148	NC_013838	could not be verified: no structure	verified	verified	no	Chang, H.-W., Lin, Z.H., Su, Y.F., Yao, C.-T. and Chou, Y.C.	2010	?	unpubl	2010	-	
included	included	*Pycnonotus*	*sinensis hainanus*	Pycnonotidae	KJ147475		could not be verified: no structure	verified	verified	no	Ren et al 2016c			2016	2016	no	
included	excluded: no paper (yet)	*Pycnonotus*	*taivanus*	Pycnonotidae	FJ378536	NC_013483	could not be verified: no structure	could not be verified: no COI	verified	no	Chang, H.-W., Su, Y.-F., Yao, C.-T., Cheng, C.-C. and Chou, Y.C.	2008	?	unpubl	2008	-	
included	included	*Pycnonotus*	*xanthorrhous*	Pycnonotidae	KX129905	NC_031830	verified	verified	verified	no	Wen & Liao 2016			2016	2016	Phylogram	*Cyanoptila cyanomelana, Pseudopodoces humilis, Passer ammodendri*
included	included	*Pygoscelis*	*adeliae*	Spheniscidae	KC875855	NC_021137	could not be verified: no ND2	verified	verified	no	Gibb et al 2013			2013	2013	Phylogram	(duplicate)
included	included	*Pygoscelis*	*adeliae*	Spheniscidae	KU356675		could not be verified: no ND2	verified	verified	no	Ramos et al. 2018			2018	2018	no	
included	included	*Pygoscelis*	*adeliae*	Spheniscidae	KU356676		could not be verified: no ND2	verified	verified	no	Ramos et al. 2018			2018	2018	no	
included	included	*Pygoscelis*	*adeliae*	Spheniscidae	GQ925782		could not be verified: no ND2	verified	verified	no	Subramanian et al. 2009			2009	2009	no	
included	included	*Pygoscelis*	*adeliae*	Spheniscidae	GQ925783		could not be verified: no ND2	verified	verified	no	Subramanian et al. 2009			2009	2009	no	
included	included	*Pygoscelis*	*adeliae*	Spheniscidae	GQ925784		could not be verified: no ND2	verified	verified	no	Subramanian et al. 2009			2009	2009	no	
included	included	*Pygoscelis*	*adeliae*	Spheniscidae	GQ925785		could not be verified: no ND2	verified	verified	no	Subramanian et al. 2009			2009	2009	no	
included	included	*Pygoscelis*	*adeliae*	Spheniscidae	GQ925786		could not be verified: no ND2	verified	verified	no	Subramanian et al. 2009			2009	2009	no	
included	included	*Pygoscelis*	*adeliae*	Spheniscidae	GQ925787		could not be verified: no ND2	verified	verified	no	Subramanian et al. 2009			2009	2009	no	
included	included	*Pygoscelis*	*adeliae*	Spheniscidae	GQ925788		could not be verified: no ND2	verified	verified	no	Subramanian et al. 2009			2009	2009	no	

Sequence database	Phylogeny database	Genus	species	family	GenBank#	RefSeq#	ND2 verified	COI verified	cyt b verified	erroneous sequence?	reference	submitted to GenBank	sequence released on GenBank	publication of paper	YEAR	Phylogeny (mitogenomic)	problematic sequences in tree
included	included	*Pygoscelis*	*adeliae*	Spheniscidae	GQ925789		could not be verified: no ND2	verified	verified	no	Subramanian et al. 2009			2009	2009	no	
included	included	*Pygoscelis*	*adeliae*	Spheniscidae	GQ925790		could not be verified: no ND2	verified	verified	no	Subramanian et al. 2009			2009	2009	no	
included	included	*Pygoscelis*	*adeliae*	Spheniscidae	GQ925791		could not be verified: no ND2	verified	verified	no	Subramanian et al. 2009			2009	2009	no	
included	included	*Pygoscelis*	*adeliae*	Spheniscidae	GQ925792		could not be verified: no ND2	verified	verified	no	Subramanian et al. 2009			2009	2009	no	
included	included	*Pygoscelis*	*adeliae*	Spheniscidae	GQ925793		could not be verified: no ND2	verified	verified	no	Subramanian et al. 2009			2009	2009	no	
included	included	*Pygoscelis*	*adeliae*	Spheniscidae	GQ925794		could not be verified: no ND2	verified	verified	no	Subramanian et al. 2009			2009	2009	no	
included	included	*Pygoscelis*	*adeliae*	Spheniscidae	GQ925795		could not be verified: no ND2	verified	verified	no	Subramanian et al. 2009			2009	2009	no	
included	included	*Pygoscelis*	*adeliae*	Spheniscidae	GQ925796		could not be verified: no ND2	verified	verified	no	Subramanian et al. 2009			2009	2009	no	
included	included	*Pygoscelis*	*adeliae*	Spheniscidae	GQ925797		could not be verified: no ND2	verified	verified	no	Subramanian et al. 2009			2009	2009	no	
included	included	*Pygoscelis*	*adeliae*	Spheniscidae	GQ925798		could not be verified: no ND2	verified	verified	no	Subramanian et al. 2009			2009	2009	no	
included	included	*Pygoscelis*	*adeliae*	Spheniscidae	GQ925799		could not be verified: no ND2	verified	verified	no	Subramanian et al. 2009			2009	2009	no	
included	included	*Pygoscelis*	*adeliae*	Spheniscidae	GQ925800		could not be verified: no ND2	verified	verified	no	Subramanian et al. 2009			2009	2009	no	
included	included	*Pygoscelis*	*adeliae*	Spheniscidae	GQ925801		could not be verified: no ND2	verified	verified	no	Subramanian et al. 2009			2009	2009	no	
included	included	*Pygoscelis*	*antarcticus*	Spheniscidae	KU356673		verified	verified	verified	no	Ramos et al. 2018			2018	2018	no	
included	included	*Pygoscelis*	*antarcticus*	Spheniscidae	KU356674		verified	verified	verified	no	Ramos et al. 2018			2018	2018	no	
included	excluded: no paper (yet)	*Pygoscelis*	*antarcticus*	Spheniscidae	KF020634	NC_021474	verified	verified	verified	no	Subramanian, S., Lambert, D., Huynen, L., Swaminathan, S. and Gowri, S.	2013	?	unpubl	2013	-	
included	included	*Pygoscelis*	*papua*	Spheniscidae	KU356677	NC_037702	could not be verified: no ND2	verified	verified	no	Ramos et al. 2018			2018	2018	no	
included	included	*Pyrrhocorax*	*graculus*	Corvidae	KJ598623	NC_025927	verified	verified	verified	no	Morinha et al 2016			2016	2016	no	
included	included	*Pyrrhocorax*	*pyrrhocorax*	Corvidae	KJ598622	NC_025926	verified	verified	verified	no	Morinha et al 2016			2016	2016	no	
included	included	*Pyrrhula*	*pyrrhula*	Fringillidae	KM078804	NC_025625	verified	verified	verified	no	Lerner et al 2011			2011	2011	Cladogram	(duplicate)

Sequence database	Phylogeny database	Genus	species	family	GenBank#	RefSeq#	ND2 verified	COI verified	cyt b verified	erroneous sequence?	reference	submitted to GenBank	sequence released on GenBank	publication of paper	YEAR	Phylogeny (mitogenomic)	problematic sequences in tree
included	included	*Pyrrhura*	*rupicola*	Psittacidae	KF751801	NC_028404	could not be verified: no ND2	could not be verified: no COI	verified	no	Urantowka et al 2016d			2016	2016	no	
included	included	*Quiscalus*	*quiscula*	Icteridae	JX516064	NC_018803	verified	verified	verified	no	Powell et al 2013			2013	2013	Cladogram	(duplicate)
included	included	*Rallina*	*euryzinoides*	Rallidae	AP010822	NC_012142	could not be verified: no ND2	verified	could not be verified: no cyt b	no	Ozaki et al 2010			2010	2010	no	
included	excluded: paper publ. after 1-1-2020	*Rallus*	*aquaticus*	Rallidae	MH229988	NC_041578	could not be verified: no ND2	verified	verified	yes (taxonomy)	Chen et al 2020c	2018	2019	2020	2019	Phylogram	(duplicate)
included	included	*Recurvirostra*	*avosetta*	Recurvirostridae	KP757766	NC_027420	could not be verified: no ND2	verified	verified	no	Hu et al 2016a			2016	2016	Cladogram	-
included	included	*Recurvirostra*	*avosetta*	Recurvirostridae	KY623657		could not be verified: no ND2	verified	verified	no	Yang et al 2017b			2017	2017	Cladogram	(duplicate)
included	included	*Regulus*	*calendula*	Regulidae	KJ909188	NC_024866	verified	verified	could not be verified: sister may be misid'd	no	Barker 2014			2014	2014	Cladogram	(duplicate)
included	excluded: no paper (yet)	*Regulus*	*regulus*	Regulidae	KT934324	NC_029837	verified	verified	verified	no	Park, C.E., Hong, S.J., Park, G.S., Kim, M.C., Park, H.C. and Shin, J.H.	2015	2016	unpubl	2016	-	
included	included	*Remiz*	*consobrinus*	Remizidae	KC463856	NC_021641	could not be verified: no ND2	verified	verified	no	Gao et al 2013			2013	2013	Phylogram	*Cyanoptila cyanomelana*, *Emberiza chrysophrys*
included	excluded: no paper (yet)	*Rhagologus*	*leucostigma*	Pachycephalidae	MF784399	NC_040956	verified	could not be verified: no COI	verified	no	Quan, J., Zhang, X., Zhou, T., Hou, Y. and Liu, X.	2017	2019	unpubl	2019	-	
included	included	*Rhea*	*americana*	Palaeognathae	Y16884	NC_000846	could not be verified: too few sequences of Palaeognathae	verified	verified	no	Harlid et al 1998			1998	1998	Phylogram	-
included	included	*Rhea*	*americana*	Palaeognathae	AF090339		could not be verified: too few sequences of Palaeognathae	verified	verified	no	Mindell et al 1999			1999	1999	Phylogram	(duplicate)
included	included	*Rhinopomastus*	*cyanomelas*	Phoeniculidae	MK060144		could not be verified: no ND2	could not be verified: no COI	could not be verified: no cyt b	?	Tamashiro et al 2019			2019	2019	Phylogram	(duplicate)
included	included	*Rhipidura*	*fuliginosa*	Rhipiduridae	KC545405	NC_029145	verified	verified	could not be verified: no structure	no	Gibb et al 2015			2015	2015	Phylogram	(duplicate)
included	included	*Rhizothera*	*longirostris*	Phasianidae	KY411597		could not be verified: no ND2	could not be verified: no COI	verified	no	Wang et al 2017a			2017	2017	Phylogram	(duplicate)

Sequence database	Phylogeny database	Genus	species	family	GenBank#	RefSeq#	ND2 verified	COI verified	cyt b verified	erroneous sequence?	reference	submitted to GenBank	sequence released on GenBank	publication of paper	YEAR	Phylogeny (mitogenomic)	problematic sequences in tree
included	included	*Rhizothera*	*longirostris*	Phasianidae	KY411598		could not be verified: no ND2	could not be verified: no COI	verified	no	Wang et al 2017a			2017	2017	Phylogram	(duplicate)
included	included	*Rhynchopsitta*	*terrisi*	Psittacidae	KF010318	NC_021771	could not be verified: no ND2	verified	could not be verified: no cyt b	no	Urantowka et al 2014b			2013	2013	no	
included	included	*Rhynchortyx*	*cinctus*	Phasianidae	KJ914547		verified	could not be verified: no COI	could not be verified: no cyt b	no	Meiklejohn et al 2014			2014	2014	Phylogram	(duplicate)
included	included	*Rhynochetos*	*jubatus*	Rhynochetidae	EF532933	NC_010091	could not be verified: no ND2	could not be verified: no COI	could not be verified: no cyt b	?	Morgan-Richards et al 2008			2008	2008	Phylogram	(duplicate)
included	included	*Rhyticeros*	*undulatus*	Bucerotidae	MG171195	NC_039950	verified	could not be verified: no COI	verified	no	Chen et al 2019b			2017	2017	no	
included	included	*Rupicola*	*peruvianus*	Cotingidae	MN602289	NC_045370	verified	could not be verified: no COI	verified	no	Bustamante et al 2019		2019	2019	2019	Phylogram	*Thamnophilus nigrocinereus*
included	included	*Sagittarius*	*serpentarius*	Accipitridae	KF961184	NC_023788	verified	could not be verified: no COI	verified	no	Mahmood et al 2014			2014	2014	Phylogram	(duplicate)
included	included	*Saltator*	*similis*	Thraupidae	MK419316	NC_045366	verified	verified	verified	no	Queiroz et al 2019		2019	2019	2019	Cladogram	-
included	included	*Sarothrura*	*ayresi*	Rallidae	KY075897	NC_034316	could not be verified: no ND2	could not be verified: no COI	could not be verified: no cyt b	?	Du Plessis et al 2017			2017	2017	Phylogram	*Coturnicops exquisitus*
included	included	*Sarothrura*	*rufa*	Rallidae	MK434263		could not be verified: no ND2	verified	verified	no	Boast et al 2019			2019	2019	Phylogram	(duplicate)
included	included	*Sasia*	*ochracea*	Picidae	KT443919	NC_028019	verified	verified	verified	no	Fuchs et al 2015			2015	2015	Phylogram	(duplicate)
included	included	*Saundersilarus*	*saundersi*	Laridae	JQ071443	NC_017601	could not be verified: no ND2	verified	verified	no	Ryu & Hwang 2012			2012	2012	no	
included	included	*Saundersilarus*	*saundersi*	Laridae	KJ631624		could not be verified: no ND2	verified	verified	no	Yoon et al 2015			2015	2015	Phylogram	-
included	included	*Scolopax*	*rusticola*	Scolopacidae	KM434134	NC_025521	verified	verified	verified	no	Yu et al 2016a			2016	2016	no	
included	included	*Scytalopus*	*magellanicus*	Rhinocryptidae	KJ909189		verified	verified	verified	no	Barker 2014			2014	2014	Cladogram	(duplicate)
included	excluded: no paper (yet)	*Seicercus*	*burkii*	Phylloscopidae	KX977449		could not be verified: no ND2	misidentified	misidentified	yes (misID)	Zhang, W., Yue, B. and Jing, J.	2016	2019	unpubl	2019	-	
included	excluded: no paper (yet)	*Seicercus*	*examinandus*	Phylloscopidae	LR026996		could not be verified: no ND2	verified	verified	no	Spiridonova, L.	2018	2019	unpubl	2019	-	
included	excluded: no paper (yet)	*Seicercus*	*examinandus*	Phylloscopidae	LR026997		could not be verified: no ND2	verified	verified	no	Spiridonova, L.	2018	2019	unpubl	2019	-	
included	included	*Serinus*	*albogularis*	Fringillidae	KM078764	NC_025595	could not be verified: no ND2	could not be verified: no COI	verified	no	Lerner et al 2011			2011	2011	Cladogram	(duplicate)
included	excluded: no paper (yet)	*Serinus*	*canaria*	Fringillidae	CM017350		verified	could not be verified: no COI	verified	no	Gazda, M.A., Sabatino, S.J., Larson, T. and Carneiro, M.	2019	2019	unpubl	2019	-	

Sequence database	Phylogeny database	Genus	species	family	GenBank#	RefSeq#	ND2 verified	COI verified	cyt b verified	erroneous sequence?	reference	submitted to GenBank	sequence released on GenBank	publication of paper	YEAR	Phylogeny (mitogenomic)	problematic sequences in tree
included	included	*Serinus*	*canaria*	Fringillidae	KM078794	NC_037521	verified	could not be verified: no COI	verified	no	Lerner et al 2011			2011	2011	Cladogram	(duplicate)
included	included	*Serinus*	*dorsostriatus*	Fringillidae	KM078798	NC_025621	could not be verified: no ND2	could not be verified: no COI	verified	no	Lerner et al 2011			2011	2011	Cladogram	(duplicate)
included	included	*Sicalis*	*olivascens*	Thraupidae	KY628988	NC_037153	verified	verified	verified	no	Caparroz et al 2018			2018	2018	Cladogram	(duplicate)
included	included	*Sitta*	*carolinensis*	Sittidae	KJ909195	NC_024870	verified	verified	verified	no	Barker 2014			2014	2014	Cladogram	(duplicate)
included	excluded: no paper (yet)	*Sitta*	*himalayensis*	Sittidae	MK343426	NC_042730	verified	verified	verified	no	Duan, Y., Li, Y. and Luo, X.	2018	2019	unpubl	2019	-	
included	excluded: no paper (yet)	*Sitta*	*nagaensis*	Sittidae	MK343427	NC_042731	verified	verified	verified	no	Duan, Y., Li, Y. and Luo, X.	2018	2019	unpubl	2019	-	
included	included	*Smicrornis*	*brevirostris*	Acanthizidae	KY994584		verified	verified	verified	no	Lamb et al 2018			2018	2018	Phylogram	(duplicate)
included	included	*Smicrornis*	*brevirostris*	Acanthizidae	KY994596		verified	verified	verified	no	Lamb et al 2018			2018	2018	Phylogram	(duplicate)
included	included	*Smicrornis*	*brevirostris*	Acanthizidae	KY994606		verified	verified	verified	no	Lamb et al 2018			2018	2018	Phylogram	(duplicate)
included	included	*Smithornis*	*sharpei*	Eurylaimidae	AF090340	NC_000879	could not be verified: no ND2	could not be verified: no COI	could not be verified: no cyt b	?	Mindell et al 1999			1999	1999	Phylogram	(duplicate)
included	included	*Spheniscus*	*demersus*	Spheniscidae	KC914350	NC_022817	verified	verified	verified	no	Labuschagne et al 2014			2014	2014	Cladogram	-
included	included	*Spheniscus*	*humboldti*	Spheniscidae	KM891593	NC_036337	verified	could not be verified: no structure	verified	no	Ramos et al. 2018			2018	2018	no	
included	included	*Spheniscus*	*humboldti*	Spheniscidae	KU361805		verified	could not be verified: no structure	verified	no	Ramos et al. 2018			2018	2018	no	
included	included	*Spheniscus*	*magellanicus*	Spheniscidae	KU361803	NC_036264	verified	verified	verified	no	Ramos et al. 2018			2018	2018	no	
included	included	*Spheniscus*	*magellanicus*	Spheniscidae	KU361804		verified	verified	verified	no	Ramos et al. 2018			2018	2018	no	
included	included	*Spheniscus*	*magellanicus*	Spheniscidae	KU361806		verified	verified	verified	no	Ramos et al. 2018			2018	2018	no	
included	included	*Spheniscus*	*mendiculus*	Spheniscidae	KU361807	NC_036297	could not be verified: no ND2	could not be verified: no structure	verified	no	Ramos et al. 2018			2018	2018	no	
included	excluded: no paper (yet)	*Spilornis*	*cheela*	Accipitridae	JN191388	NC_015887	verified	verified	could not be verified: unclear if discrepancy due to tax, seq issues, or misid	no	Qin, X., Shi, J., Guan, Q. and Zeng, D.	2011	?	unpubl	2011	-	
included	excluded: no paper (yet)	*Spizella*	*atrogularis*	Passerellidae	FJ236294		verified	verified	verified	no	Carson, R.J. and Spicer, G.S.	2008	?	unpubl	2008	-	
included	included	*Spizixos*	*semitorques*	Pycnonotidae	KJ174511	NC_029321	verified	verified	verified	no	Ren et al 2016a			2016	2016	no	
included	excluded: no paper (yet)	*Spizixos*	*semitorques*	Pycnonotidae	KU058638		verified	verified	verified	no	Yang, D. and Lu, C.	2015	2017	unpubl	2017	-	
included	included	*Sporophila*	*angolensis*	Thraupidae	MK433296		could not be verified: no structure	could not be verified: no structure	could not be verified: no structure	?	Lima-Rezende et al 2019		2019	2019	2019	no	

Sequence database	Phylogeny database	Genus	species	family	GenBank#	RefSeq#	ND2 verified	COI verified	cyt b verified	erroneous sequence?	reference	submitted to GenBank	sequence released on GenBank	publication of paper	YEAR	Phylogeny (mitogenomic)	problematic sequences in tree
included	included	*Sporophila*	*maximiliani*	Thraupidae	MF327582	NC_035673	verified	could not be verified: no COI	could not be verified: no structure	no	Ludwig et al 2017			2017	2017	Phylogram	-
included	included	*Stachyris*	*ruficeps*	Timaliidae	KU362930	NC_030771	verified	verified	verified	no	Wu et al 2016c			2016	2016	Phylogram	-
included	included	*Stercorarius*	*maccormicki*	Stercorariidae	KM401546	NC_026125	could not be verified: no ND2	could not be verified: no structure	could not be verified: no structure	?	Han et al 2016			2016	2016	no	
included	excluded: no paper (yet)	*Sterna*	*hirundo*	Laridae	CM020500		verified	verified	verified	no	Liedvogel, M., Caswara, C., Formenti, G., Chow, W., Wood, J., Howe, K., Bouwhuis, S., Fedrigo, O. and Jarvis, E.D.		2019	unpubl	2019	-	
included	included	*Sterna*	*hirundo*	Laridae	MF582632	NC_036345	verified	verified	verified	no	Yang et al 2017a			2017	2017	Cladogram	(duplicate)
included	included	*Sterna*	*paradisaea*	Laridae	MK946458	NC_045282	verified	verified	verified	no	Skujina et al 2019		2019	2019	2019	Phylogram	-
included	excluded: no paper (yet)	*Sternula (Sterna)*	*albifrons*	Laridae	KT350612	NC_028176	verified	verified	verified	no	Park, C.E., Park, G.S., Jung, B.K., Park, Y.J., Kim, M.C., Park, H.C., Shin, J.H.	2015	2015	unpubl	2015	-	
included	included	*Streptopelia*	*chinensis*	Columbidae	KP636801		verified	verified	verified	no	Huang et al 2016c			2016	2016	Phylogram	-
included	included	*Streptopelia*	*chinensis*	Columbidae	KP273832	NC_026459	verified	verified	verified	no	Yan et al 2016a			2016	2016	no	
included	excluded: no paper (yet)	*Streptopelia*	*decaocto*	Columbidae	KY827036	NC_037513	verified	verified	verified	no	Qu, J.	2017	2018	unpubl	2018	-	
included	excluded: no paper (yet)	*Streptopelia*	*decaocto*	Columbidae	KX372273		verified	verified	verified	no	Qu, J., Guo, C., Ma, J., Chen, Y., Li, Y. and Wang, X.	2016	2016	unpubl	2016	-	
included	included	*Streptopelia*	*orientalis*	Columbidae	KT182929	NC_031447	verified	verified	verified	no	Huang et al 2016b			2016	2016	no	
included	excluded: no paper (yet)	*Streptopelia*	*orientalis*	Columbidae	KY827037		verified	verified	verified	no	Qu, J.	2017	2018	unpubl	2018	-	
included	excluded: no paper (yet)	*Streptopelia*	*orientalis*	Columbidae	KX156849		verified	verified	verified	no	Qu, J., Guo, C. and Shi, B.	2016	2017	unpubl	2017	-	
included	included	*Strigops*	*habroptila*	Strigopidae	AY309456	NC_005931	verified	verified	could not be verified: no cyt b	no	Harrison et al 2004			2004	2004	Phylogram	(duplicate)
included	excluded: no paper (yet)	*Strigops*	*habroptila*	Strigopidae	CM013787		verified	verified	could not be verified: no cyt b	no	Jarvis, E.D., Howard, J., Rhie, A., Phillippy, A., Korlach, J., et al	2018	2019	unpubl	2019	-	
included	included	*Strix*	*leptogrammica*	Strigidae	KC953095	NC_021970	verified	could not be verified: no COI	problematic	yes (seq errors/numts)	Liu et al 2014c (Liu et al 2019)			2014	2014	no	
included	included	*Strix*	*occidentalis caurina*	Strigidae	MF431746		verified	verified	could not be verified: no cyt b	no	Hanna et al 2017a			2017	2017	no	
included	excluded: no paper (yet)	*Strix*	*uralensis*	Strigidae	KU237289		verified	verified	verified	no	Hwang, U.W.	2015	2018	unpubl	2018	-	

Sequence database	Phylogeny database	Genus	species	family	GenBank#	RefSeq#	ND2 verified	COI verified	cyt b verified	erroneous sequence?	reference	submitted to GenBank	sequence released on GenBank	publication of paper	YEAR	Phylogeny (mitogenomic)	problematic sequences in tree
included	included	*Strix*	*uralensis*	Strigidae	MG681081	NC_038218	could not be verified: no ND2	verified	verified	no	Kang et al 2018			2018	2018	Phylogram	(duplicate)
included	included	*Strix*	*uralensis*	Strigidae	MG681082		could not be verified: no ND2	verified	verified	no	Kang et al 2018			2018	2018	Phylogram	(duplicate)
included	included	*Strix*	*varia*	Strigidae	MF431745		could not be verified: no ND2	verified	could not be verified: no cyt b	no	Hanna et al 2017			2017	2017	no	
included	included	*Struthio*	*camelus*	Palaeognathae	AF338715	NC_002785	could not be verified: too few sequences of Palaeognathae	verified	verified	no	Haddrath & Baker 2001			2001	2001	Phylogram	(duplicate)
included	included	*Struthio*	*camelus*	Palaeognathae	Y12025		could not be verified: too few sequences of Palaeognathae	verified	verified	no	Harlid et al 1997			1997	1997	Phylogram	-
included	excluded: no paper (yet)	*Sturnella*	*neglecta*	Icteridae	FJ236288		verified	verified	verified	no	Carson, R.J. and Spicer, G.S.	2008	?	unpubl	2008	-	
included	excluded: no paper (yet)	*Sturnus*	*cineraceus*	Sturnidae	HQ896037	NC_015237	verified	verified	could not be verified: no cyt b	no	Kan, X.-Z. and Qian, C.-J.	2011	?	unpubl	2011	-	
included	excluded: no paper (yet)	*Sturnus*	*nigricollis*	Sturnidae	JQ003191	NC_020423	verified	verified	could not be verified: no cyt b	no	Kan, X.-Z. and Qian, C.-J.	2011	2013	unpubl	2013	-	
included	excluded: no paper (yet)	*Sturnus*	*nigricollis*	Sturnidae	JQ003192		verified	problematic	could not be verified: no cyt b	yes (chimera)	Kan, X.-Z. and Qian, C.-J.	2011	2013	unpubl	2013	-	
included	included	*Sturnus*	*sericeus*	Sturnidae	HM859900	NC_014455	verified	could not be verified: no COI	could not be verified: no cyt b	no	Qian et al 2013c			2013	2013	no	
included	included	*Sturnus*	*vulgaris*	Sturnidae	KT946691	NC_029360	could not be verified: no structure	verified	verified	no	Rollins et al 2016			2016	2016	no	
included	included	*Sturnus*	*vulgaris*	Sturnidae	KT946692		could not be verified: no structure	verified	verified	no	Rollins et al 2016			2016	2016	no	
included	excluded: no paper (yet)	*Sturnus (Acridotheres)*	*tristis*	Sturnidae	HQ915864	NC_015195	verified	verified	verified	no	Kan, X.-Z. and Qian, C.-J.	2011	?	unpubl	2011	-	
included	included	*Sula*	*dactylatra*	Sulidae	KC875857		verified	verified	could not be verified: too little cyt b in seq	no	Gibb et al 2013			2013	2013	Phylogram	(duplicate)
included	excluded: no paper (yet)	*Sylvia*	*atricapilla*	Sylviidae	MN122898		verified	verified	verified	no	Margaryan, A.		2019	unpubl	2019	-	
included	included	*Sylvia*	*atricapilla*	Sylviidae	AM889140	NC_010228	verified	verified	verified	no	Singh et al. 2008			2008	2008	no	
included	included	*Sylvia*	*crassirostris*	Sylviidae	AM889141	NC_010229	verified	verified	verified	no	Singh et al. 2008			2008	2008	no	
included	included	*Sylviparus*	*modestus*	Paridae	KP642167	NC_026793	verified	verified	verified	no	Wang & Huang 2016			2016	2016	no	

Sequence database	Phylogeny database	Genus	species	family	GenBank#	RefSeq#	ND2 verified	COI verified	cyt b verified	erroneous sequence?	reference	submitted to GenBank	sequence released on GenBank	publication of paper	YEAR	Phylogeny (mitogenomic)	problematic sequences in tree
included	included	*Synthliboramphus*	*antiquus*	Alcidae	AP009042	NC_007978	verified	verified	verified	no	Yamamoto et al 2005a	2005		2005	2005	no	
included	included	*Synthliboramphus*	*wumiusume*	Alcidae	KT592378	NC_029328	verified	verified	verified	no	Eo & An 2016a			2016	2016	Phylogram	*Vanellus cinereus*
included	excluded: no paper (yet)	*Syrmaticus*	*elliotti*	Phasianidae	AB164624	NC_010771	could not be verified: no structure	verified	verified	no	Kato, S., Nishibori, M. and Yasue, H.	2004		unpubl	2004	-	
included	excluded: no paper (yet)	*Syrmaticus*	*humiae*	Phasianidae	AB164625	NC_010774	could not be verified: no structure	could not be verified: no COI	dubious	no	Kato, S., Nishibori, M. and Yasue, H.	2004		unpubl	2004	-	
included	excluded: no paper (yet)	*Syrmaticus*	*reevesii*	Phasianidae	AB164623	NC_010770	verified	verified	verified	no	Kato, S., Nishibori, M. and Yasue, H.	2004		unpubl	2004	-	
included	excluded: no paper (yet)	*Syrmaticus*	*soemmerringi ijimae*	Phasianidae	AB164622	NC_010767	could not be verified: no ND2	verified	verified	no	Kato, S., Nishibori, M. and Yasue, H.	2004		unpubl	2004	-	
included	included	*Tachybaptus*	*novaehollandiae*	Podicipedidae	EF532936	NC_010095	could not be verified: no ND2	verified	could not be verified: no cyt b	no	Morgan-Richards et al 2008			2008	2008	Phylogram	(duplicate)
included	included	*Tachybaptus*	*ruficollis*	Podicipedidae	KJ913674	NC_024594	verified	verified	verified	no	Wang et al 2017b			2017	2017	Phylogram	-
included	included	*Tachycineta*	*albilinea*	Hirundinidae	JQ071619	NC_020601	verified	verified	verified	no	Cerasale et al 2012			2012	2012	Phylogram	(duplicate)
included	included	*Tachycineta*	*albiventer*	Hirundinidae	JQ071620	NC_020602	verified	verified	verified	no	Cerasale et al 2012			2012	2012	Phylogram	(duplicate)
included	included	*Tachycineta*	*bicolor*	Hirundinidae	JQ071614	NC_020596	verified	verified	verified	no	Cerasale et al 2012			2012	2012	Phylogram	(duplicate)
included	included	*Tachycineta*	*cyaneoviridis*	Hirundinidae	JQ071617	NC_020599	verified	could not be verified: no COI	verified	no	Cerasale et al 2012			2012	2012	Phylogram	(duplicate)
included	included	*Tachycineta*	*euchrysea*	Hirundinidae	JQ071616	NC_020598	verified	could not be verified: no COI	verified	no	Cerasale et al 2012			2012	2012	Phylogram	(duplicate)
included	included	*Tachycineta*	*leucorrhoa*	Hirundinidae	JQ071621	NC_020603	verified	verified	verified	no	Cerasale et al 2012			2012	2012	Phylogram	(duplicate)
included	included	*Tachycineta*	*meyeni*	Hirundinidae	JQ071622	NC_020604	verified	verified	verified	no	Cerasale et al 2012			2012	2012	Phylogram	(duplicate)
included	included	*Tachycineta*	*stolzmanni*	Hirundinidae	JQ071618	NC_020600	verified	could not be verified: no COI	verified	no	Cerasale et al 2012			2012	2012	Phylogram	(duplicate)
included	included	*Tachycineta*	*thalassina*	Hirundinidae	JQ071615	NC_020597	verified	verified	verified	no	Cerasale et al 2012			2012	2012	Phylogram	(duplicate)
included	included	*Tadorna*	*ferruginea*	Anatidae	KF684946	NC_024640	verified	verified	verified	no	Liu et al 2014a			2014	2014	Phylogram	(duplicate)
included	included	*Tadorna*	*tadorna*	Anatidae	MN258348		verified	dubious	dubious	yes (seq errors/numts)	Liu et al 2019k		2019	2019	2019	Phylogram	*Anser fabalis, Tadorna tadorna*
included	included	*Tadorna*	*tadorna*	Anatidae	KU140668		verified	verified	verified	no	Sun et al 2017a			2017	2017	Cladogram	(duplicate)
included	included	*Tadorna*	*tadorna (Lin Wu breed)*	Anatidae	KJ794187	NC_024750	incorrect on GenBank	incorrect on GenBank	incorrect on GenBank	yes (mislabelled)	Lin et al 2016a (He et al 2016b)			2016	2016	no	
included	excluded: no paper (yet)	*Taeniopygia*	*guttata*	Estrildidae	CM018288		verified	could not be verified: no COI	verified	no	Jarvis, E.D., Rhie, A., Fedrigo, O., Mountcastle, J. et al	2019	2019	unpubl	2019	-	

Sequence database	Phylogeny database	Genus	species	family	GenBank#	RefSeq#	ND2 verified	COI verified	cyt b verified	erroneous sequence?	reference	submitted to GenBank	sequence released on GenBank	publication of paper	YEAR	Phylogeny (mitogenomic)	problematic sequences in tree
included	excluded: no paper (yet)	*Taeniopygia*	*guttata*	Estrildidae	CM020865		verified	could not be verified: no COI	verified	no	Jarvis, E.D., Rhie, A., Fedrigo, O., Mountcastle, J. et al		2019	unpubl	2019	-	
included	excluded: no paper (yet)	*Taeniopygia*	*guttata*	Estrildidae	CM016613		verified	could not be verified: no COI	verified	no	Jarvis, E.D., Rhie, A., Korlach, J., Fedrigo, O., et al	2018	2019	unpubl	2019	-	
included	excluded: no paper (yet)	*Taeniopygia*	*guttata*	Estrildidae	CM018229		verified	could not be verified: no COI	verified	no	Jarvis, E.D., Rhie, A., Mountcastle, J., Biegler, M. et al	2019	2019	unpubl	2019	-	
included	included	*Taeniopygia*	*guttata*	Estrildidae	DQ422742	NC_007897	verified	could not be verified: no COI	verified	no	Mossman et al 2006			2006	2006	no	
included	included	*Taeniopygia*	*guttata*	Estrildidae	DQ453512		verified	could not be verified: no COI	verified	no	Mossman et al 2006			2006	2006	no	
included	included	*Taeniopygia*	*guttata*	Estrildidae	DQ453513		verified	could not be verified: no COI	verified	no	Mossman et al 2006			2006	2006	no	
included	included	*Taeniopygia*	*guttata*	Estrildidae	DQ453514		verified	could not be verified: no COI	verified	no	Mossman et al 2006			2006	2006	no	
included	included	*Taeniopygia*	*guttata*	Estrildidae	DQ453515		verified	could not be verified: no COI	verified	no	Mossman et al 2006			2006	2006	no	
included	excluded: no paper (yet)	*Tangara (Thraupis)*	*episcopus*	Thraupidae	FJ236299		verified	verified	verified	no	Carson, R.J. and Spicer, G.S.	2008	?	unpubl	2008	-	
included	included	*Tangara (Thraupis)*	*episcopus*	Thraupidae	KM078765	NC_025596	verified	verified	verified	no	Lerner et al 2011			2011	2011	Cladogram	(duplicate)
included	included	*Tanygnathus*	*lucionensis*	Psittaculidae	KM611480		verified	verified	could not be verified: no cyt b	no	Eberhard & Wright 2016			2016	2016	Cladogram	(duplicate)
included	included	*Tarsiger*	*cyanurus*	Muscicapidae	KF997864	NC_026067	verified	verified	verified	no	Zhang et al 2018b			2018	2018	Phylogram	(duplicate)
included	included	*Telespiza*	*ultima*	Fringillidae	KM078787		could not be verified: no ND2	could not be verified: failed alignment	could not be verified: no structure	?	Lerner et al 2011			2011	2011	Cladogram	(duplicate)
included	included	*Terpsiphone*	*atrocaudata*	Monarchidae	KT901458	NC_032725	verified	verified	verified	no	Eo & An 2016b			2016	2016	Phylogram	*Lanius tephronotus*
included	excluded: paper publ. after 1-1-2020	*Tetrao*	*parvirostris kamtschaticus*	Phasianidae	MK820676	NC_043950	verified	verified	verified	no	Spiridonova et al 2020	2019	2019	2020	2019	no	
included	excluded: paper publ. after 1-1-2020	*Tetrao*	*parvirostris kamtschaticus*	Phasianidae	MK820677		verified	verified	verified	no	Spiridonova et al 2020	2019	2019	2020	2019	no	
included	excluded: paper publ. after 1-1-2020	*Tetrao*	*parvirostris parvirostris*	Phasianidae	MK820675	NC_043949	verified	verified	verified	no	Spiridonova et al 2020	2019	2019	2020	2019	no	

Sequence database	Phylogeny database	Genus	species	family	GenBank#	RefSeq#	ND2 verified	COI verified	cyt b verified	erroneous sequence?	reference	submitted to GenBank	sequence released on GenBank	publication of paper	YEAR	Phylogeny (mitogenomic)	problematic sequences in tree
included	excluded: paper publ. after 1-1-2020	*Tetrao*	*parvirostris parvirostris*	Phasianidae	MK820678		verified	verified	verified	no	Spiridonova et al 2020	2019	2019	2020	2019	no	
included	included	*Tetrao*	*urogallus aquitanicus*	Phasianidae	MG583885		verified	verified	verified	no	Aleix-Mata et al 2019			2019	2019	no	
included	included	*Tetraogallus*	*himalayensis*	Phasianidae	KR349185	NC_027279	verified	could not be verified: no COI	verified	no	An et al 2016a			2016	2016	Phylogram	*Francolinus pintadeanus*
included	excluded: no paper (yet)	*Tetraogallus*	*himalayensis*	Phasianidae	KY766922		verified	could not be verified: no COI	verified	no	Li, X.-J., Huang, Y. and Lei, F.-M.	2017	2019	unpubl	2019	-	
included	included	*Tetraogallus*	*himalayensis*	Phasianidae	KY411599		verified	could not be verified: no COI	verified	no	Wang et al 2017a			2017	2017	Phylogram	(duplicate)
included	excluded: no paper (yet)	*Tetraogallus*	*tibetanus*	Phasianidae	KY766921		verified	could not be verified: no COI	verified	no	Li, X.-J., Huang, Y. and Lei, F.-M.	2017	2019	unpubl	2019	-	
included	included	*Tetraogallus*	*tibetanus*	Phasianidae	KF027439	NC_023939	verified	could not be verified: no COI	verified	no	Zhou et al 2014a			2014	2014	Cladogram	(duplicate)
included	included	*Tetraophasis*	*obscurus*	Phasianidae	JF921876	NC_018034	verified	could not be verified: no COI	verified	no	Liu et al 2014b			2014	2014	Cladogram	*Phasianus versicolor*
included	included	*Tetraophasis*	*szechenyii*	Phasianidae	FJ799728		verified	verified	verified	no	Meng et al 2010			2010	2010	Phylogram	*Phasianus versicolor, Francolinus pintadeanus*
included	included	*Tetraophasis*	*szechenyii*	Phasianidae	FJ752428	NC_020613	verified	verified	verified	no	Shen et al 2010			2010	2010	Phylogram	(duplicate)
included	included	*Tetrastes (Bonasa)*	*bonasia*	Phasianidae	FJ752435	NC_020591	verified	verified	verified	no	Shen et al 2010			2010	2010	Phylogram	(duplicate)
included	included	*Tetrastes (Bonasa)*	*bonasia*	Phasianidae	KY411600		verified	verified	verified	no	Wang et al 2017a			2017	2017	Phylogram	(duplicate)
included	included	*Tetrastes (Bonasa)*	*sewerzowi*	Phasianidae	KJ997914	NC_025318	verified	could not be verified: no COI	verified	no	Li et al 2014b			2014	2014	Phylogram	-
included	included	*Thalassarche*	*chrysostoma*	Diomedeidae	AP009193		could not be verified: no ND2	verified	verified	no	Watanabe et al 2006			2006	2006	Phylogram	(duplicate)
included	included	*Thalassarche*	*melanophris*	Diomedeidae	AY158677	NC_007172	could not be verified: no ND2	verified	verified	no	Slack et al 2006			2006	2006	Cladogram	(duplicate)
included	included	*Thamnophilus*	*nigrocinereus*	Thamnophilidae	KJ909192		misidentified	misidentified	problematic	yes (misID)	Barker 2014			2014	2014	Cladogram	(duplicate)
included	excluded: no paper (yet)	*Threskiornis*	*aethiopicus*	Threskiornithidae	GQ358927	NC_013146	could not be verified: no ND2	verified	verified	no	Cheng, Y.-Y., Chang, H.-W., Yao, C.-T., Chiu, C.-C., Chang, W.-C. and Chou, Y.C.	2009	?	unpubl	2009	-	
included	included	*Threskiornis*	*aethiopicus*	Threskiornithidae	MN196512		could not be verified: no ND2	verified	verified	no	Wasef et al 2019		2019	2019	2019	Phylogram	-
included	included	*Threskiornis*	*aethiopicus*	Threskiornithidae	MN196513		could not be verified: no ND2	verified	verified	no	Wasef et al 2019		2019	2019	2019	Phylogram	(duplicate)

Sequence database	Phylogeny database	Genus	species	family	GenBank#	RefSeq#	ND2 verified	COI verified	cyt b verified	erroneous sequence?	reference	submitted to GenBank	sequence released on GenBank	publication of paper	YEAR	Phylogeny (mitogenomic)	problematic sequences in tree
included	included	*Threskiornis*	*aethiopicus*	Threskiornithidae	MN196514		could not be verified: no ND2	verified	verified	no	Wasef et al 2019		2019	2019	2019	Phylogram	(duplicate)
included	included	*Threskiornis*	*aethiopicus*	Threskiornithidae	MN196515		could not be verified: no ND2	verified	verified	no	Wasef et al 2019		2019	2019	2019	Phylogram	(duplicate)
included	included	*Threskiornis*	*aethiopicus*	Threskiornithidae	MN196516		could not be verified: no ND2	verified	verified	no	Wasef et al 2019		2019	2019	2019	Phylogram	(duplicate)
included	included	*Threskiornis*	*aethiopicus*	Threskiornithidae	MN196517		could not be verified: no ND2	verified	verified	no	Wasef et al 2019		2019	2019	2019	Phylogram	(duplicate)
included	included	*Threskiornis*	*aethiopicus*	Threskiornithidae	MN196518		could not be verified: no ND2	verified	verified	no	Wasef et al 2019		2019	2019	2019	Phylogram	(duplicate)
included	included	*Threskiornis*	*aethiopicus*	Threskiornithidae	MN196519		could not be verified: no ND2	verified	verified	no	Wasef et al 2019		2019	2019	2019	Phylogram	(duplicate)
included	included	*Threskiornis*	*aethiopicus*	Threskiornithidae	MN196520		could not be verified: no ND2	verified	verified	no	Wasef et al 2019		2019	2019	2019	Phylogram	(duplicate)
included	included	*Threskiornis*	*aethiopicus*	Threskiornithidae	MN196521		could not be verified: no ND2	verified	verified	no	Wasef et al 2019		2019	2019	2019	Phylogram	(duplicate)
included	included	*Threskiornis*	*aethiopicus*	Threskiornithidae	MN196522		could not be verified: no ND2	verified	verified	no	Wasef et al 2019		2019	2019	2019	Phylogram	(duplicate)
included	included	*Threskiornis*	*aethiopicus*	Threskiornithidae	MN196523		could not be verified: no ND2	verified	verified	no	Wasef et al 2019		2019	2019	2019	Phylogram	(duplicate)
included	included	*Threskiornis*	*aethiopicus*	Threskiornithidae	MN196524		could not be verified: no ND2	verified	verified	no	Wasef et al 2019		2019	2019	2019	Phylogram	(duplicate)
included	included	*Threskiornis*	*aethiopicus*	Threskiornithidae	MN196525		could not be verified: no ND2	verified	verified	no	Wasef et al 2019		2019	2019	2019	Phylogram	(duplicate)
included	included	*Threskiornis*	*aethiopicus*	Threskiornithidae	MN196526		could not be verified: no ND2	verified	verified	no	Wasef et al 2019		2019	2019	2019	Phylogram	(duplicate)
included	included	*Threskiornis*	*aethiopicus*	Threskiornithidae	MN196527		could not be verified: no ND2	verified	verified	no	Wasef et al 2019		2019	2019	2019	Phylogram	(duplicate)
included	included	*Threskiornis*	*aethiopicus*	Threskiornithidae	MN196528		could not be verified: no ND2	verified	verified	no	Wasef et al 2019		2019	2019	2019	Phylogram	(duplicate)
included	included	*Threskiornis*	*aethiopicus*	Threskiornithidae	MN196529		could not be verified: no ND2	verified	verified	no	Wasef et al 2019		2019	2019	2019	Phylogram	(duplicate)
included	included	*Threskiornis*	*aethiopicus*	Threskiornithidae	MN196530		could not be verified: no ND2	verified	verified	no	Wasef et al 2019		2019	2019	2019	Phylogram	(duplicate)
included	included	*Threskiornis*	*aethiopicus*	Threskiornithidae	MN196531		could not be verified: no ND2	verified	verified	no	Wasef et al 2019		2019	2019	2019	Phylogram	(duplicate)

Sequence database	Phylogeny database	Genus	species	family	GenBank#	RefSeq#	ND2 verified	COI verified	cyt b verified	erroneous sequence?	reference	submitted to GenBank	sequence released on GenBank	publication of paper	YEAR	Phylogeny (mitogenomic)	problematic sequences in tree
included	included	*Threskiornis*	*aethiopicus*	Threskiornithidae	MN196532		could not be verified: no ND2	verified	verified	no	Wasef et al 2019		2019	2019	2019	Phylogram	(duplicate)
included	included	*Threskiornis*	*aethiopicus*	Threskiornithidae	MN196533		could not be verified: no ND2	verified	verified	no	Wasef et al 2019		2019	2019	2019	Phylogram	(duplicate)
included	included	*Threskiornis*	*aethiopicus*	Threskiornithidae	MN196534		could not be verified: no ND2	verified	verified	no	Wasef et al 2019		2019	2019	2019	Phylogram	(duplicate)
included	included	*Threskiornis*	*aethiopicus*	Threskiornithidae	MN196535		could not be verified: no ND2	verified	verified	no	Wasef et al 2019		2019	2019	2019	Phylogram	(duplicate)
included	included	*Threskiornis*	*aethiopicus*	Threskiornithidae	MN196536		could not be verified: no ND2	verified	verified	no	Wasef et al 2019		2019	2019	2019	Phylogram	(duplicate)
included	included	*Threskiornis*	*aethiopicus*	Threskiornithidae	MN196537		could not be verified: no ND2	verified	verified	no	Wasef et al 2019		2019	2019	2019	Phylogram	(duplicate)
included	included	*Tinamus*	*guttatus*	Palaeognathae	KR149454	NC_027260	could not be verified: too few sequences of Palaeognathae	could not be verified: no COI	could not be verified: no cyt b	?	An et al 2016b			2016	2016	Cladogram	-
included	included	*Tinamus*	*major*	Palaeognathae	AF338707	NC_002781	could not be verified: too few sequences of Palaeognathae	verified	could not be verified: no cyt b	no	Haddrath & Baker 2001			2001	2001	Phylogram	(duplicate)
included	included	*Todus*	*angustirostris*	Todidae	MK060145		verified	could not be verified: no COI	verified	no	Tamashiro et al 2019			2019	2019	Phylogram	(duplicate)
included	included	*Tragopan*	*caboti*	Phasianidae	GU187969	NC_013619	verified	verified	verified	no	Kan et al 2010a			2010	2010	no	
included	included	*Tragopan*	*temminckii*	Phasianidae	FJ752427	NC_020586	verified	verified	verified	no	Shen et al 2010			2010	2010	Phylogram	(duplicate)
included	included	*Traversia*	*lyalli*	Acanthisittidae	KX369034		could not be verified: no ND2	could not be verified: no COI	could not be verified: no cyt b	?	Mitchell et al 2016			2016	2016	Cladogram	(duplicate)
included	included	*Tregellasia*	*capito*	Petroicidae	KM374637	NC_027231	verified	verified	could not be verified: no cyt b	no	Morales et al 2015			2015	2015	Cladogram	(duplicate)
included	included	*Tregellasia*	*leucops*	Petroicidae	KJ909197	NC_024871	verified	verified	verified	no	Barker 2014			2014	2014	Cladogram	(duplicate)
included	included	*Tregellasia*	*leucops*	Petroicidae	KM374655		verified	verified	verified	no	Morales et al 2015			2015	2015	Cladogram	(duplicate)
included	included	*Trichoglossus*	*rubritorquis*	Psittaculidae	MN182499		verified	verified	verified	no	Xu et al 2019		2019	2019	2019	Phylogram	*Aquila heliaca, Brotogeris cyanoptera*
included	included	*Tringa*	*erythropus*	Scolopacidae	KX230491	NC_030585	verified	verified	verified	no	Cheng et al 2016			2016	2016	Phylogram	-
included	excluded: no paper (yet)	*Tringa*	*glareola*	Scolopacidae	KY128485	NC_039096	verified	verified	verified	no	Cheng, Y. and Zhou, L.	2016	2018	unpubl	2018	-	
included	included	*Tringa*	*guttifer*	Scolopacidae	MK905885	NC_044665	could not be verified: no ND2	could not be verified: no COI	could not be verified: no cyt b	?	Liu et al 2019i		2019	2019	2019	Phylogram	-
included	included	*Tringa*	*nebularia*	Scolopacidae	MK460251	NC_044651	verified	verified	verified	no	Zhang et al 2019		2019	2019	2019	Phylogram	-
included	included	*Tringa*	*ochropus*	Scolopacidae	KX668223	NC_033974	verified	verified	verified	no	Chen et al 2016			2016	2016	Cladogram	-

Sequence database	Phylogeny database	Genus	species	family	GenBank#	RefSeq#	ND2 verified	COI verified	cyt b verified	erroneous sequence?	reference	submitted to GenBank	sequence released on GenBank	publication of paper	YEAR	Phylogeny (mitogenomic)	problematic sequences in tree
included	included	*Tringa*	*semipalmata*	Scolopacidae	MF036175	NC_036016	verified	verified	verified	no	Bi 2017			2017	2017	network	*Charadrius placidus, Larus vegae*
included	included	*Tringa*	*totanus*	Scolopacidae	MK922124	NC_044648	verified	verified	problematic	yes (seq errors/numts)	Ren et al 2019b		2019	2019	2019	Phylogram	*Tringa totanus*
included	included	*Trogon*	*personatus*	Trogonidae	MK060146		verified	dubious	dubious	?	Tamashiro et al 2019			2019	2019	Phylogram	(duplicate)
included	included	*Trogon*	*viridis*	Trogonidae	EU410490	NC_011714	verified	verified	verified	yes (taxonomy)	Pratt et al 2009			2009	2009	Phylogram	(duplicate)
included	included	*Trugon*	*terrestris*	Columbidae	MG590263	NC_036611	verified	dubious	dubious	no	Bruxaux et al 2018			2018	2018	Phylogram	(duplicate)
included	included	*Turdus*	*eunomus*	Turdidae	KM015261	NC_028273	could not be verified: no structure	could not be verified: no COI	could not be verified: no structure	?	Dong et al 2018			2018	2018	no	
included	included	*Turdus*	*hortulorum*	Turdidae	KF926987	NC_024552	verified	could not be verified: no structure	verified	no	Yan et al 2016b			2016	2016	no	
included	included	*Turdus*	*kessleri*	Turdidae	MG912943	NC_041095	verified	could not be verified: no COI	could not be verified: no structure	no	Song et al 2018			2018	2018	Phylogram	*Turdus merula 2*
included	included	*Turdus*	*merula 1*	Turdidae	KT601060		could not be verified: no ND2 (sister to KT373849)	could not be verified: no COI (sister to KT373849)	old taxonomy	yes (taxonomy)	Peng et al 2016c			2016	2016	Phylogram	*Cyanoptila cyanomelana, Pseudopodoces humilis, Turdus merula 1*
included	excluded: no paper (yet)	*Turdus*	*merula 2*	Turdidae	KT373849	NC_028188	could not be verified: no ND2 (sister to KT601060)	could not be verified: no COI (sister to KT601060)	old taxonomy	yes (taxonomy)	Wen, L., Fu, Y., Dai, B. and Liao, J.	2015	2015	unpubl	2015	-	
included	included	*Turdus*	*migratorius*	Turdidae	KJ909198	NC_024872	verified	verified	verified	no	Barker 2014			2014	2014	Cladogram	(duplicate)
included	included	*Turdus*	*naumanni*	Turdidae	KJ834096		could not be verified: no structure	could not be verified: no structure	could not be verified: no structure	?	Li et al 2016h			2016	2016	no	
included	included	*Turdus*	*philomelos*	Turdidae	KC545406	NC_029147	verified	verified	verified	no	Gibb et al 2015			2015	2015	Phylogram	(duplicate)
included	included	*Turdus*	*rufiventris*	Turdidae	KT346357	NC_028179	verified	verified	verified	no	Gomes de Sa et al 2017			2017	2017	Phylogram	*Cyanoptila cyanomelana*
included	included	*Turnagra*	*capensis*	Pachycephalidae	KU158197		verified	could not be verified: no COI	verified	no	Anmarkrud & Lifjeld 2017			2017	2017	no	
included	included	*Turnagra*	*capensis*	Pachycephalidae	KT894672	NC_028336	verified	could not be verified: no COI	verified	no	Gibb et al 2015			2015	2015	Phylogram	(duplicate)
included	included	*Turnix*	*velox*	Turnicidae	MK453380		could not be verified: no ND2	could not be verified: no COI	could not be verified: no cyt b	?	Grealy et al 2019			2019	2019	Phylogram	(duplicate)
included	included	*Turtur*	*tympanistria*	Columbidae	HM746793		verified	verified	verified	no	Pacheco et al 2011			2011	2011	Phylogram	(duplicate)
included	included	*Tyto*	*alba*	Tytonidae	EU410491		verified	verified	verified	no	Pratt et al 2009			2009	2009	Phylogram	(duplicate)
included	included	*Tyto*	*longimembris*	Tytonidae	KP893332		misidentified	problematic	verified	yes (chimera)	Xu et al 2016b			2016	2016	Cladogram	*Tyto longimembris, Bubo bubo, Caprimulgus jotaka, Strix leptogrammica*

Sequence database	Phylogeny database	Genus	species	family	GenBank#	RefSeq#	ND2 verified	COI verified	cyt b verified	erroneous sequence?	reference	submitted to GenBank	sequence released on GenBank	publication of paper	YEAR	Phylogeny (mitogenomic)	problematic sequences in tree
included	excluded: no paper (yet)	*Upupa*	*epops*	Upupidae	KT356220	NC_028178	verified	verified	verified	no	Park, C.E., Park, G.S., Jung, B.K., Park, Y.J., Kim, M.C., Park, H.C., Shin, J.H.	2015	2015	unpubl	2015	-	
included	included	*Upupa*	*epops*	Upupidae	MK060147		verified	verified	verified	no	Tamashiro et al 2019			2019	2019	Phylogram	(duplicate)
included	included	*Uragus*	*sibiricus*	Fringillidae	KM078763	NC_025594	verified	verified	verified	no	Lerner et al 2011			2011	2011	Cladogram	(duplicate)
included	included	*Urocissa*	*caerulea*	Corvidae	MG932654	NC_037486	verified	could not be verified: no COI	could not be verified: no cyt b	no	Hsieh et al 2018			2018	2018	Phylogram	-
included	included	*Urocissa*	*erythroryncha*	Corvidae	JQ423932	NC_020426	verified	verified	verified	no	Liu et al 2018d			2018	2018	Phylogram	*Corvus coronoides, Cyanoptila cyanomelana*
included	included	*Vanellus*	*cinereus*	Charadriidae	KM873665		could not be verified: no ND2	verified	problematic	yes (chimera)	She et al 2016			2016	2016	no	
included	included	*Vanellus*	*cinereus*	Charadriidae	KM404175	NC_025514	could not be verified: no ND2	verified	could not be verified: no cyt b	no	Xie et al 2016b			2016	2016	no	
included	included	*Vanellus*	*vanellus*	Charadriidae	KM577158	NC_025637	could not be verified: no ND2	verified	verified	no	Hu et al 2016b			2016	2016	no	
included	excluded: no paper (yet)	*Vermivora*	*celata*	Parulidae	FJ236284		verified	verified	verified	no	Carson, R.J. and Spicer, G.S.	2008	?	unpubl	2008	-	
included	included	*Vestiaria*	*coccinea*	Fringillidae	KM078797	NC_025620	verified	could not be verified: no structure	verified	no	Lerner et al 2011			2011	2011	Cladogram	(duplicate)
included	included	*Vidua*	*chalybeata*	Estrildidae	AF090341	NC_000880	could not be verified: no structure	could not be verified: no COI	verified	no	Mindell et al 1999			1999	1999	Phylogram	(duplicate)
included	included	*Vireo*	*olivaceus*	Vireonidae	KJ909193	NC_024869	verified	verified	verified	no	Barker 2014			2014	2014	Cladogram	(duplicate)
included	included	*Xanthopsar*	*flavus*	Icteridae	JX516065	NC_018804	verified	could not be verified: no COI	verified	no	Powell et al 2013			2013	2013	Cladogram	(duplicate)
included	included	*Xenicus*	*gilviventris*	Acanthisittidae	KX369033		verified	could not be verified: no COI	verified	no	Mitchell et al 2016			2016	2016	Cladogram	(duplicate)
included	included	*Xenicus*	*longipes*	Acanthisittidae	KX369035		could not be verified: no ND2	could not be verified: no COI	could not be verified: no cyt b	?	Mitchell et al 2016			2016	2016	Cladogram	(duplicate)
included	included	*Xenus*	*cinereus*	Scolopacidae	KX644890	NC_033973	verified	verified	verified	no	Liu et al 2016b			2016	2016	Cladogram	-
included	included	*Yuhina*	*diademata*	Timaliidae	KT783535	NC_029462	verified	verified	verified	no	Li et al 2017a			2017	2017	Phylogram	*Garrulax perspicillatus*
included	included	*Yuhina*	*gularis*	Timaliidae	MK405666		verified	verified	verified	no	He et al 2019b		2019	2019	2019	Phylogram	-
included	included	*Yuhina*	*nigrimenta*	Timaliidae	MH916608	NC_040991	verified	could not be verified: no COI	verified	no	He et al 2019a			2019	2019	Phylogram	-
included	included	*Zenaida*	*auriculata*	Columbidae	HM640211	NC_015203	verified	verified	verified	no	Pacheco et al 2011			2011	2011	Phylogram	(duplicate)

Sequence database	Phylogeny database	Genus	species	family	GenBank#	RefSeq#	ND2 verified	COI verified	cyt b verified	erroneous sequence?	reference	submitted to GenBank	sequence released on GenBank	publication of paper	YEAR	Phylogeny (mitogenomic)	problematic sequences in tree
included	included	*Zenaida*	*macroura*	Columbidae	KX902235	NC_031863	could not be verified: no structure	verified	verified	no	Soares et al 2016			2016	2016	Cladogram	(duplicate)
included	excluded: no paper (yet)	*Zonotrichia*	*leucophrys*	Passerellidae	FJ236292		could not be verified: no structure	could not be verified: no structure	verified	no	Carson, R.J. and Spicer, G.S.	2008	?	unpubl	2008	-	
included	included	*Zoothera*	*aurea*	Turdidae	KT340629		verified	verified	verified	no	Park et al 2019d			2019	2019	Phylogram	*Turdus merula 2*
included	included	*Zosterops*	*abyssinicus*	Zosteropidae	KX181885	NC_032058	could not be verified: no ND2	could not be verified: no COI	verified	no	Husemann et al 2016			2016	2016	Phylogram	-
included	included	*Zosterops*	*borbonicus*	Zosteropidae	MK529728		could not be verified: no ND2	could not be verified: no COI	verified	no	Leroy et al 2018			2018	2018	no	
included	included	*Zosterops*	*erythropleurus*	Zosteropidae	KT194322	NC_027942	could not be verified: no structure	verified	verified	no	Li et al 2016d			2016	2016	Phylogram	-
included	included	*Zosterops*	*japonicus*	Zosteropidae	KT601061		verified	verified	verified	no	Yang et al 2016f			2016	2016	Phylogram	-
included	included	*Zosterops*	*lateralis*	Zosteropidae	KC545407	NC_029146	verified	verified	verified	no	Gibb et al 2015			2015	2015	Phylogram	(duplicate)
included	included	*Zosterops*	*pallidus*	Zosteropidae	MK524996		could not be verified: no structure	verified	verified	no	Leroy et al 2018			2018	2018	no	
included	included	*Zosterops*	*poliogastrus*	Zosteropidae	KX181886	NC_032059	could not be verified: no ND2	could not be verified: no COI	verified	no	Husemann et al 2016			2016	2016	Phylogram	(duplicate)
included	included	*Zosterops*	*senegalensis (Kakamega forest)*	Zosteropidae	KX181888		verified	could not be verified: no COI	verified	no	Husemann et al 2016			2016	2016	Phylogram	(duplicate)
included	included	*Zosterops*	*senegalensis (Mt Nyeri)*	Zosteropidae	KX181887		dubious	dubious	dubious	dubious	Husemann et al 2016			2016	2016	Phylogram	(duplicate)
excluded: no new mitogenome	included	-	-	Anatidae	mitogenomic study (no new seqs)		-	-	-	-	Dai et al 2016 (Mitochondrial DNA Part A, 2016; 27(4): 2464–2465)	-	-	2016	2016	Phylogram	*Anas falcata, Anas crecca, Anser fabalis*
excluded: no new mitogenome	included	-	-	Galliformes	mitogenomic study (no new seqs)		-	-	-	-	Huang & Ke 2015 (Mitochondrial DNA, 2015; 26(6): 949–950)	-	-	2015	2015	Phylogram	*Phasianus versicolor, Francolinus pintadeanus*
excluded: no new mitogenome	included	-	-	Galliformes	mitogenomic study (no new seqs)		-	-	-	-	Jiang et al 2019 (Mitochondrial DNA Part B 4: 723-724)	-	-	2019	2019	Phylogram	-
excluded: no new mitogenome	included	-	-	Galliformes	mitogenomic study (no new seqs)		-	-	-	-	Kan et al 2010c (Afr J Biotech)	-	-	2010	2010	Cladogram	*Phasianus versicolor, Francolinus pintadeanus*
excluded: no new mitogenome	included	-	-	Galliformes	mitogenomic study (no new seqs)		-	-	-	-	Kan et al 2010d (Gen Mol Res)	-	-	2010	2010	Phylogram	*Phasianus versicolor, Francolinus pintadeanus*
excluded: no new mitogenome	included	-	-	Fringillidae	mitogenomic study (no new seqs)		-	-	-	-	Kim et al 2018b (Mitochondrial DNA Part B 3: 833-834)	-	-	2018	2018	Phylogram	-

Sequence database	Phylogeny database	Genus	species	family	GenBank#	RefSeq#	ND2 verified	COI verified	cyt b verified	erroneous sequence?	reference	submitted to GenBank	sequence released on GenBank	publication of paper	YEAR	Phylogeny (mitogenomic)	problematic sequences in tree
excluded: no new mitogenome	included	-	-	Anatidae	mitogenomic study (no new seqs)		-	-	-	-	Liu et al 2016a	-	-	2016	2016	Cladogram	*Anser fabalis*
excluded: no new mitogenome	included	-	-	Aves	mitogenomic study (no new seqs)		-	-	-	-	Mackiewicz et al 2019 (GBE)	-	-	2019	2019	Phylogram	*Bombycilla cedrorum, Corvus coronoides, Emberiza aureola, Emberiza chrysophrys, Leucosticte arctoa, Hirundo rustica gutturalis, Lanius tephronotus, Pterorhinus perspicillatus, Pterorhinus poecilorhynchus, Motacilla alba lugens, Cyanoptila cyanomelana, Periparus ater, Passer ammodendri, Eopsaltria griseogularis, Gracupica nigricollis, Thamnophilus nigrocinereus, Falco naumanni, Turdus merula 2,*
excluded: no new mitogenome	included	-	-	Aves	mitogenomic study (no new seqs)		-	-	-	-	Nabholz et al 2010 (MPE)	-	-	2010	2010	Phylogram	-
excluded: no new mitogenome	included	-	-	Aves	mitogenomic study (no new seqs)		-	-	-	-	Nabholz et al 2013 (GBE)	-	-	2013	2013	Cladogram	-

Sequence database	Phylogeny database	Genus	species	family	GenBank#	RefSeq#	ND2 verified	COI verified	cyt b verified	erroneous sequence?	reference	submitted to GenBank	sequence released on GenBank	publication of paper	YEAR	Phylogeny (mitogenomic)	problematic sequences in tree
excluded: no new mitogenome	included	-	-	Aves	mitogenomic study (no new seqs)		-	-	-	-	Nabholz et al 2016 (Mol Ecol)	-	-	2016	2016	Phylogram	*Aix galericulata, Anas falcata, Bombycilla cedrorum, Ceryle rudis, Emberiza aureola, Emberiza chrysophrys, Leucosticte arctoa, Lanius tephronotus, Netta rufina, Cyanoptila cyanomelana, Periparus ater, Francolinus pintadeanus, Phasianus versicolor, Thamnophilus nigrocinereus, Garrulax perspicillatus, Hirundo rustica, Vanellus cinereus, Coturnicops exquisitus, Amaurornis phoenicurus, Anas crecca, Brotogeris cyanoptera, Trogon viridis*
excluded: no new mitogenome	included	-	-	Paridae	mitogenomic study (no new seqs)		-	-	-	-	Wang et al 2015b (Sci. Rep.)	-	-	2015	2015	Cladogram	*Periparus ater*

APPENDIX S2. Problematic mitogenomes identified in this study (n=71), including evidence from gene trees and re-use in subsequent studies.

1. "*Centrocercus minimus*" CM016737 (Oh et al. 2019)

Fig. S1

Metadata: "Genomic DNA was extracted ... from a whole blood sample collected in a previous study (Oyler-McCance et al. 2005) from a single *C. minimus* male in a small population (estimated 98 individuals, 2014 U.S. Fish and Wildlife Service) near Dove Creek, Colorado and Monticello, Utah, USA." (Oh et al. 2019, online supporting information, p.1).

Phylogenetic position:

ND2 = *Centrocercus minimus*

CO1 = sister to all *Centrocercus minimus*/*C. urophasianus*

Cyt *b* = *Centrocercus minimus*/*C. urophasianus* (no resolution) but on a long branch

Sequence integrity: COI: deletions at bp 345, 481; insertion at bp 164A.

ND2 deletions at bp 6, 103, 112, 123, 151, 191, 192, 408, 532, 542, 739, 917, 967; insertions at bp 84T, 306T, 437T, 491A, 665A, 698A, 935A, 1005A.

Cyt b: deletions at bp 346, 392, 396, 432, 777, 798, 1019; insertions at bp 14A, 80C, 266T, 364T, 453T, 470A, 471A, 498G, 590C.

Interpretation: Sequencing errors / numts. The (frameshift) insertions and deletions in cytochrome *b* and ND2 suggest that these fragments may consist of numts. A previous study showed that it is difficult to obtain high-coverage authentic mitogenomes from blood of this species (Barker et al. 2015).

Subsequent usage: none so far (January 2020)

Problems noted in other works: none.

Relevance: This was the only complete mitogenome presumed to be of this species (January 2020). A previous study obtained small fragments of mitochondrial DNA from this species (Barker et al. 2015).

References:

Barker, FK, Oyler-McCance, S & Tomback, DF 2015. Blood from a turnip: tissue origin of low-coverage shotgun sequencing libraries affects recovery of mitogenome sequences. Mitochondrial DNA 26: 384–388.

Oh, KP, Aldridge, CL, Forbey, JS, Dadabay, CY & Oyler-McCance, SJ 2019. Conservation genomics in the sagebrush sea: population divergence, demographic history, and local adaptation in sage-grouse (*Centrocercus* spp.). Genome Biology and Evolution 11: 2023–2034.

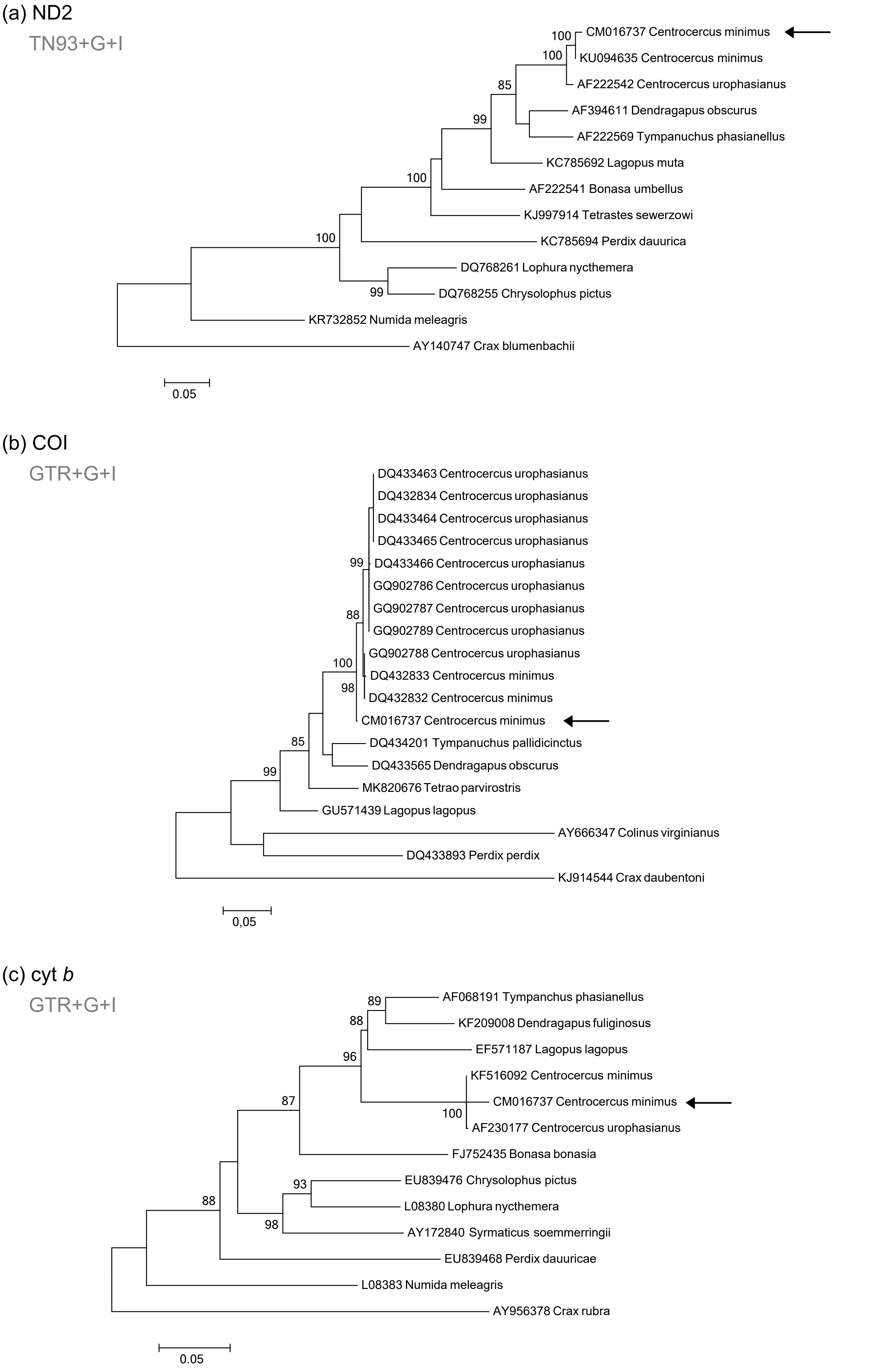

Fig. S1. Maximum Likelihood phylogenies of *Centrocercus minimus* (CM016737) and related taxa based on three mitochondrial markers. Numbers at branches are bootstrap support values (>70%) based on 1000 replicates.

2. “*Francolinus pintadeanus*” EU165707, NC_011817 (Shen et al. 2009)

Fig. S2

Metadata: No information in Shen et al. (2009) or on GenBank.
Phylogenetic position:
ND2 = *Francolinus pintadeanus* (close sister to KX196445).
CO1 = *Francolinus pintadeanus* (close sister to KX196445).
Cyt *b* = among *Coturnix*, and sister to all *C. coturnix/C. japonica* (distant from *Francolinus pintadeanus* KX196445).
Sequence integrity: cyt b: bp 288-1143 show 99.7-99.9% similarity to *Coturnix japonica/C. coturnix* (BLAST).
Interpretation: chimera
Subsequent usage:
Re-used in **20 mitogenomic phylogenies published before 1 January 2020**: Kan et al. 2010c, Kan et al. 2010d, Ma et al. 2010, Shen et al. 2010, Meng et al. 2010, Zhao et al. 2012, Zeng et al. 2013, Yan et al. 2014, Li et al. 2014a, Shen et al. 2014, Meiklejohn et al. 2014, Huang & Ke 2015, Zhou et al. 2015b, An et al. 2016a, Nabholz et al. 2016, Grass et al. 2016, Yan et al. 2017, Zhao et al. 2017, Hirimuthugoda et al. 2018, Gao et al. 2019a.
Re-used in **2 non-mitogenomic phylogenies**: Huang & Ke 2014, Mandiwana-Neudani et al. 2014 (**evolution of vocalizations**).
Cited by **2 other papers**: Huang & Ke 2016, Kan et al. 2010c.
Problems noted in other works: none.
Relevance: Another mitogenome (KX196445) of this species was published by Li & Lin (2016).
References:

An, B, Zhang L, Ruan L, Liu N, Zhang Z, Abutalip A, Suo Y 2016a. The complete mitochondrial genome of Himalayan Snowcock (*Tetraogallus himalayensis*). Mitochondrial DNA A 27: 3751-3752.
Gao, H, Liu, Z, Sun, Y, Zhao, C, Wang, J & Teng, L 2019a. The complete mitochondrial genome of Helan Mountain chukar *Alectoris chukar potanini* (Galliformes: Phasianidae). Mitochondrial DNA Part B 4: 2443-2444.
Grass, AA, Hosie, C & McDowall, IL 2016. The complete mitochondrial genome and phylogenetic position of the critically endangered Trinidad Piping Guan, *Pipile pipile* synonym *Aburria pipile* (Aves: Galliformes). Mitochondrial DNA Part B 1: 649-650.
Hirimuthugoda, NY, Adeola, AC, Chen, X, Perera, PWA, Gunawardena, WWDA, Gunwardana, HGTN, Yin, TT, Wang, MS, Li, GM, Peng, MS & Zhang, YP 2018. Complete mitochondrial genome of Sri Lankan Junglefowl (*Gallus lafayetti*) and phylogenetic study. Mitochondrial DNA Part B 3: 83-84.
Huang, ZH & Ke, DH 2014. DNA barcoding and evolutionary relationships of the Phasianidae family in China. Genet. Mol. Res. 13: 7411-7419.
Huang, Z & Ke, D 2015. Phylogenetic relationship of *Pucrasia* (Aves: Galliformes) based on complete mitochondrial genome sequences. Mitochondrial DNA 26: 949-950.
Huang, Z & Ke, D 2016. Structure and evolution of the Phasianidae mitochondrial DNA control region. Mitochondrial DNA Part A 27: 350-354.
Kan, XZ, Li, XF, Lei, ZP, Chen, L, Gao, H, Yang, ZY, Yang, JK, Guo, ZC, Yu, L, Zhang, LQ & Qian, CJ 2010c. Estimation of divergence times for major lineages of galliform birds: Evidence from complete mitochondrial genome sequences. African Journal of Biotechnology 9: 3073-3078.
Kan, XZ, Yang, JK, Li, XF, Chen, L, Lei, ZP, Wang, M, Qian, CJ, Gao, H & Yang ZY 2010d. Phylogeny of major lineages of galliform birds (Aves: Galliformes) based on complete mitochondrial genomes. Genet. Mol. Res. 9: 1625-1633.
Li, X, Huang, Y & Lei, F 2014a. Comparative and phylogenomic analyses on mitochondrial genomes of *Arborophila* species. Yi chuan= Hereditas 36: 912-920.
Li, XJ & Lin, LL 2016. Complete mitochondrial genome of *Francolinus pintadeanus* (Galliformes: Phasianidae). Mitochondrial DNA Part B 1: 579-580.

Ma, LL, Zhang, XY, Yue, BS & Ran, JH 2010. Complete mitochondrial genome of the Chinese Monal pheasant *Lophophorus lhuysii*, with phylogenetic implication in Phasianidae. Mitochondrial DNA 21: 5-7.

Mandiwana-Neudani, TG, Bowie, RCK, Hausberger, M, Henry, L & Crowe, TM 2014. Taxonomic and phylogenetic utility of variation in advertising calls of francolins and spurfowls (Galliformes: Phasianidae). African Zoology 49: 54-82.

Meiklejohn, KA, Danielson, MJ, Faircloth, BC, Glenn, TC, Braun, EL & Kimball, RT 2014. Incongruence among different mitochondrial regions: A case study using complete mitogenomes. Molecular Phylogenetics and Evolution 78: 314-323.

Meng, Y, He, L, Wu, A, Fan, Z, Ran, J, Yue, B & Li, J 2010. Complete mitochondrial genome of *Tetraophasis szechenyii* Madarász, 1885 (Aves: Galliformes: Phasianidae), and its genetic variation as inferred from the mitochondrial DNA control region. Journal of Natural History 44: 2955-2964.

Nabholz, B, Lanfear, R & Fuchs, J 2016. Body mass-corrected molecular rate for bird mitochondrial DNA. Mol. Ecol. 25: 4438-4449.

Shen, YY, Dai, K, Cao, X, Murphy, RW, Shen, XJ & Zhang, YP 2014. The updated phylogenies of the Phasianidae based on combined data of nuclear and mitochondrial DNA. PLoS One 9(4), e95786.

Shen, YY, Liang, L, Sun, YB, Yue, BS, Yang, XJ, Murphy, RW & Zhang YP 2010. A mitogenomic perspective on the ancient, rapid radiation in the Galliformes with an emphasis on the Phasianidae. BMC Evol. Biol. 10: 132.

Shen, YY, Shi, P, Sun, YB & Zhang, YP 2009. Relaxation of selective constraints on avian mitochondrial DNA following the degeneration of flight ability. Genome Research 19: 1760–1765.

Yan, C, Mou, B, Meng, Y, Tu, F, Fan, Z, Price, M, Yue, B & Zhang, X 2017. A novel mitochondrial genome of *Arborophila* and new insight into *Arborophila* evolutionary history. PloS one 12(7), e0181649.

Yan, C, Zhou, Y, Lu, L, Tu, F, Huang, T, Zhang, X & Yue, B 2014. Complete mitochondrial genome of Hainan partridge, *Arborophila ardens* (Galliformes: Phasianidae). Mitochondrial DNA 25: 259–260.

Zeng, T, Tu, F, Ma, L, Yan, C, Yang, N, Zhang, X, Yue, B & Ran, J 2013. Complete mitochondrial genome of blood pheasant (*Ithaginis cruentus*). Mitochondrial DNA 24: 484-486.

Zhao, S, Ma, Y, Wang, G, Li, H, Liu, X, Yu, J, Yue, B & Zou, F 2012. Molecular phylogeny of major lineages of the avian family Phasianidae inferred from complete mitochondrial genome sequences. J. Nat. Hist. 46: 757-767.

Zhao, C, Gao, H, Sun, Y, Liu, Z & Teng, L 2017. Complete mitochondrial genome of ring-necked pheasant *Phasianus colchicus alaschanicus* from China's Helan Mountains and description of its phylogenetic relationships in Galliformes. Biochemical Systematics and Ecology 70: 43-49.

Zhou, TC, Sha, T, Irwin, DM & Zhang, YP 2015b. Complete mitochondrial genome of the Indian peafowl (*Pavo cristatus*), with phylogenetic analysis in phasianidae. Mitochondrial DNA 26: 912–913.

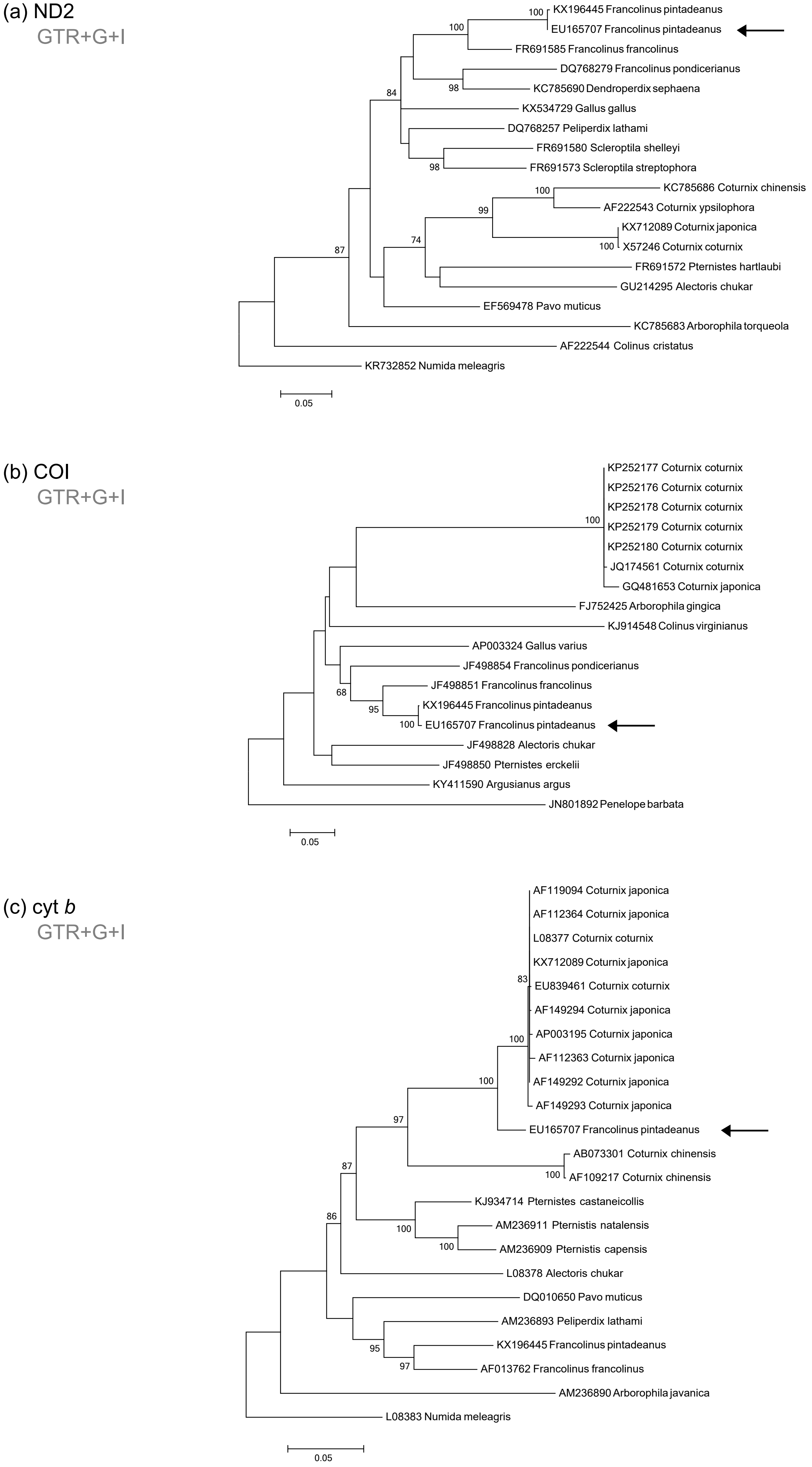

Figure S2. Maximum Likelihood phylogenies of *Francolinus pintadeanus* (EU165707) and related taxa based on mitochondrial sequences. Numbers at branches are bootstrap support values (>70%) based on 1000 replicates.

3. “*Gallus gallus*” KT626849 (Alexander et al. 2015)

Fig. S3

Metadata: A blood sample was taken from an adult female, isolate 5439 (cf. GenBank), from the “Virginia chicken lines, an experimental White Plymouth Rock population spanning more than 50 generations” (p. 1). This number is not mentioned in the supplementary data of Alexander et al. (2015).

Phylogenetic position:

ND2 = *Gallus gallus*

CO1 = *Gallus gallus* but on a very long branch

Cyt *b* = *Gallus gallus*

Sequence integrity: COI: bp 5-318 includes 42 singleton sites (i.e. nucleotide substitutions not found in 173 other complete or partial mitogenomes of this species).

Interpretation: Sequencing errors / numts

Subsequent usage: not re-used in any further studies (January 2020).

Problems noted in other works: none.

Relevance: 173 other mitogenomes of this species are available on GenBank.

References:

Alexander, M, Ho, SYW, Molak, M, Barnett, R, Carlborg, Ö, Dorshorst, B, Honaker, C, Besnier, F, Wahlberg, P, Dobney, K, Siegel, P, Andersson, L & Larson, G 2015. Mitogenomic analysis of a 50-generation chicken pedigree reveals a rapid rate of mitochondrial evolution and evidence for paternal mtDNA inheritance. Biology Letters 11: 20150561.

4. “*Phasianus colchicus*” KX512321 (Zhu et al. 2017)

Fig. S3

Metadata: “In this study, the *J. phasianus colchicus* was obtained from Guangyuan County, Sichuan Province, China. Samples of *J. phasianus colchicus* were recorded by the Ministry of Agriculture of the People's Republic of China (announcement No. 2437).” (Zhu et al. 2017: 205)

Phylogenetic position:

ND2 = *Gallus gallus*

CO1 = *Gallus gallus*

Cyt *b* = *Gallus gallus*

Sequence integrity: not investigated

Interpretation: Incorrectly identified in Zhu et al. (2017) as *Phasianus colchicus*, correctly listed on GenBank as *Gallus gallus*.

Subsequent usage: not re-used (as “*Phasianus colchicus*”) in any further studies (January 2020).

Problems noted in other works: none.

Relevance: Six other mitogenomes of this species are available from GenBank (FJ752430, JF739859, KJ502237, KT364526, KU049722, KP637175).

References:

Zhu, Y, Zhang, Y, Hu, YD, Lan, D, Pang, HZ, Ling, SS & Wang, CD 2017 Complete mitochondrial genome sequence of *Jianmen-guan phasianus colchicus* (Aves, Gallifoemes [sic], Phasianidae) and its phylogenetic analysis. Mitochondrial DNA Part B 2: 205-206.

5. “*Phasianus versicolor*” AB164626, NC_010778 (Kato, S., Nishibori, M. and Yasue, H., unpublished; 2004)

Fig. S3

Metadata: GenBank: /tissue_type="whole blood"; /dev_stage="adult"
Phylogenetic position:
ND2 = could not be verified (due to lack of *P. versicolor* sequences) but sister to *P. colchicus*
CO1 = identical to other *P. versicolor*
Cyt *b* = sister to *Phasianus versicolor* and *P. colchicus*
Sequence integrity: Cyt *b* bp 423 –756 correspond to *Syrmaticus soemmerringii* (99% similarity).
Interpretation: chimera
Subsequent usage:
Re-used in **27 mitogenomic phylogenies published before 1 January 2020**: He et al. 2009, Kan et al. 2010a, Kan et al. 2010b, Ma et al. 2010, Meng et al. 2010, Shen et al. 2010, Jiang et al. 2010, Li et al. 2011, Zhao et al. 2012, Zeng et al. 2013, Liu et al. 2014b, Yan et al. 2014, Li et al. 2014a, Meiklejohn et al. 2014, Zhou et al. 2014a, Shen et al. 2014, Jiang et al. 2014, Huang & Ke 2015, Zhou et al. 2015b, Li & Lin 2016, Ren et al. 2016e, Wu et al. 2016a, Nabholz et al. 2016, Yan et al. 2017, Zhao et al. 2017, Ren et al. 2019, Gao et al. 2019.
Re-used in **1 non-mitogenomic phylogeny**: Liu et al. 2010.
Cited by **4 other papers**: Kan et al. 2010c; Shehzad et al. 2012 (**NGS Leopard Cat diet**), Li et al. 2013, Huang & Ke 2016.
Problems noted in other works: none.
Relevance: This was the only mitogenome presumed to be of this species (January 2020).
References:

Gao, H, Liu, Z, Sun, Y, Zhao, C, Wang, J & Teng, L 2019. The complete mitochondrial genome of Helan Mountain chukar *Alectoris chukar potanini* (Galliformes: Phasianidae). Mitochondrial DNA Part B 4: 2443-2444.

He, L, Dai, B, Zeng, B, Zhang, X, Chen, B, Yue, B, & Li, J 2009. The complete mitochondrial genome of the Sichuan Hill Partridge (*Arborophila rufipectus*) and a phylogenetic analysis with related species. Gene 435 23-28.

Huang, Z & Ke, D 2015. Phylogenetic relationship of *Pucrasia* (Aves: Galliformes) based on complete mitochondrial genome sequences. Mitochondrial DNA 26: 949-950.

Huang, Z & Ke, D 2016. Structure and evolution of the Phasianidae mitochondrial DNA control region. Mitochondrial DNA Part A 27: 350-354.

Jiang, F, Miao, Y, Liang, W, Ye, H, Liu, H & Liu, B 2010. The complete mitochondrial genomes of the whistling duck (*Dendrocygna javanica*) and Black Swan (*Cygnus atratus*): dating evolutionary divergence in the Galloanserae. Mol. Biol. Rep. 37: 3001-3015.

Jiang, L, Wang, G, Peng, R, Peng, Q, & Zou, F 2014. Phylogenetic and molecular dating analysis of Taiwan Blue Pheasant (*Lophura swinhoii*). Gene 539: 21-29.

Kan, XZ, Li, XF, Lei, ZP, Chen, L, Gao, H, Yang, ZY, Yang, JK, Guo, ZC, Yu, L, Zhang, LQ & Qian, CJ 2010a. Estimation of divergence times for major lineages of galliform birds: Evidence from complete mitochondrial genome sequences. African Journal of Biotechnology 9: 3073-3078.

Kan, XZ, Li, XF, Lei, ZP, Wang, M, Chen, L, Gao, H & Yang, ZY 2010c. Complete mitochondrial genome of Cabot's tragopan, *Tragopan caboti* (Galliformes: Phasianidae). Genet. Mol Res. 9: 1204-1016.

Kan, XZ, Yang, JK, Li, XF, Chen, L, Lei, ZP, Wang, M, Qian, CJ, Gao, H & Yang ZY 2010b. Phylogeny of major lineages of galliform birds (Aves: Galliformes) based on complete mitochondrial genomes. Genet. Mol. Res. 9: 1625-1633.

Li, H-M, Shi, J-P, Zeng, D-L, Zeng, Z-H & Qin, X-M 2011. The complete mitochondrial genome of *Chrysolophus pictus* (Galliformes: Phasianidae) and a phylogenetic analysis with related species. Mitochondrial DNA 22: 159-161.

Li, X, Huang, Y & Lei, F 2014a. Comparative and phylogenomic analyses on mitochondrial genomes of *Arborophila* species. Yi chuan= Hereditas 36: 912-920.

Li, X, Ren, Q, Kan, X, Qian, C, Li, X & Qian, M 2013. Complete mitochondrial genome of the ring-necked pheasant, *Phasianus colchicus* (Galliformes: Phasianidae). Mitochondrial DNA 24: 89-90.
Li, XJ & Lin, LL 2016. Complete mitochondrial genome of *Francolinus pintadeanus* (Galliformes: Phasianidae). Mitochondrial DNA Part B 1: 579-580.
Liu, F, Ma, L, Yang, C, Tu, F, Xu, Y, Ran, J, Yue, B & Zhang, X 2014b. Taxonomic status of *Tetraophasis obscurus* and *Tetraophasis szechenyii* (Aves: Galliformes: Phasianidae) based on the complete mitochondrial genome. Zoological Science 31: 160-167.
Liu, Y, Zhan, X, Wang, N, Chang, J & Zhang, Z 2010. Effect of geological vicariance on mitochondrial DNA differentiation in Common Pheasant populations of the Loess Plateau and eastern China. Mol. Phylogen. Evol. 55: 409-417.
Ma, LL, Zhang, XY, Yue, BS & Ran, JH 2010. Complete mitochondrial genome of the Chinese Monal pheasant *Lophophorus lhuysii*, with phylogenetic implication in Phasianidae. Mitochondrial DNA 21: 5-7.
Meiklejohn, KA, Danielson, MJ, Faircloth, BC, Glenn, TC, Braun, EL & Kimball, RT 2014. Incongruence among different mitochondrial regions: A case study using complete mitogenomes. Molecular Phylogenetics and Evolution 78: 314-323.
Meng, Y, He, L, Wu, A, Fan, Z, Ran, J, Yue, B & Li, J 2010. Complete mitochondrial genome of *Tetraophasis szechenyii* Madarász, 1885 (Aves: Galliformes: Phasianidae), and its genetic variation as inferred from the mitochondrial DNA control region. Journal of Natural History 44: 2955-2964.
Nabholz, B, Lanfear, R & Fuchs, J 2016. Body mass-corrected molecular rate for bird mitochondrial DNA. Mol. Ecol. 25: 4438-4449.
Ren, Q, Li, X, Yuan, J, Chen, D, Zhang, L, Guo, W, Jiang, L, Wang, P & Kan, X 2016e. Complete mitochondrial genome of the Blue Eared Pheasant, *Crossoptilon auritum* (Galliformes: Phasianidae). Mitochondrial DNA Part A 27: 615-617.
Ren, Z, Liang, Y, Su, X & Wen, J 2019. Complete mitochondrial genome of *Chrysolophus pictus* (Galliformes: Phasianidae), a protected and endangered pheasant species of China. Conservation Genetics Resources, in press.
Shehzad, W, Riaz, T., Nawaz, M. A., Miquel, C., Poillot, C., Shah, S. A., et al. 2012. Carnivore diet analysis based on next-generation sequencing: application to the leopard cat (Prionailurus bengalensis) in Pakistan. Molecular Ecology 21: 1951-1965.
Shen, YY, Dai, K, Cao, X, Murphy, RW, Shen, XJ & Zhang, YP 2014. The updated phylogenies of the Phasianidae based on combined data of nuclear and mitochondrial DNA. PLoS One 9(4), e95786.
Shen, YY, Liang, L, Sun, YB, Yue, BS, Yang, XJ, Murphy, RW & Zhang YP 2010. A mitogenomic perspective on the ancient, rapid radiation in the Galliformes with an emphasis on the Phasianidae. BMC Evol. Biol. 10: 132.
Wu, Q, Li, Y, Song, C, Ning, H & Xing, X 2016a. Complete mitochondrial genome of the Mongolia pheasant, *Phasianus colchicus kiangsuensis* (Galliformes, Phasianidae, Phasianus). Mitochondrial DNA Part B 1: 399-400.
Yan, C, Mou, B, Meng, Y, Tu, F, Fan, Z, Price, M, Yue, B & Zhang, X 2017. A novel mitochondrial genome of *Arborophila* and new insight into *Arborophila* evolutionary history. PloS one 12(7), e0181649.
Yan, C, Zhou, Y, Lu, L, Tu, F, Huang, T, Zhang, X & Yue, B 2014. Complete mitochondrial genome of Hainan partridge, *Arborophila ardens* (Galliformes: Phasianidae). Mitochondrial DNA 25: 259–260.
Zeng, T, Tu, F, Ma, L, Yan, C, Yang, N, Zhang, X, Yue, B & Ran, J 2013. Complete mitochondrial genome of blood pheasant (*Ithaginis cruentus*). Mitochondrial DNA 24: 484-486.
Zhao, C, Gao, H, Sun, Y, Liu, Z & Teng, L 2017. Complete mitochondrial genome of ring-necked pheasant *Phasianus colchicus alaschanicus* from China's Helan Mountains and description of its phylogenetic relationships in Galliformes. Biochemical Systematics and Ecology 70: 43-49.
Zhao, S, Ma, Y, Wang, G, Li, H, Liu, X, Yu, J, Yue, B & Zou, F 2012. Molecular phylogeny of major lineages of the avian family Phasianidae inferred from complete mitochondrial genome sequences. J. Nat. Hist. 46: 757-767.
Zhou, T, Shen, X, Irwin, DM, Shen, Y & Zhang, Y 2014a. Mitogenomic analyses propose positive selection in mitochondrial genes for high-altitude adaptation in galliform birds. Mitochondrion 18: 70-75.

Zhou, TC, Sha, T, Irwin, DM & Zhang, YP 2015b. Complete mitochondrial genome of the Indian peafowl (*Pavo cristatus*), with phylogenetic analysis in phasianidae. Mitochondrial DNA 26: 912–913.

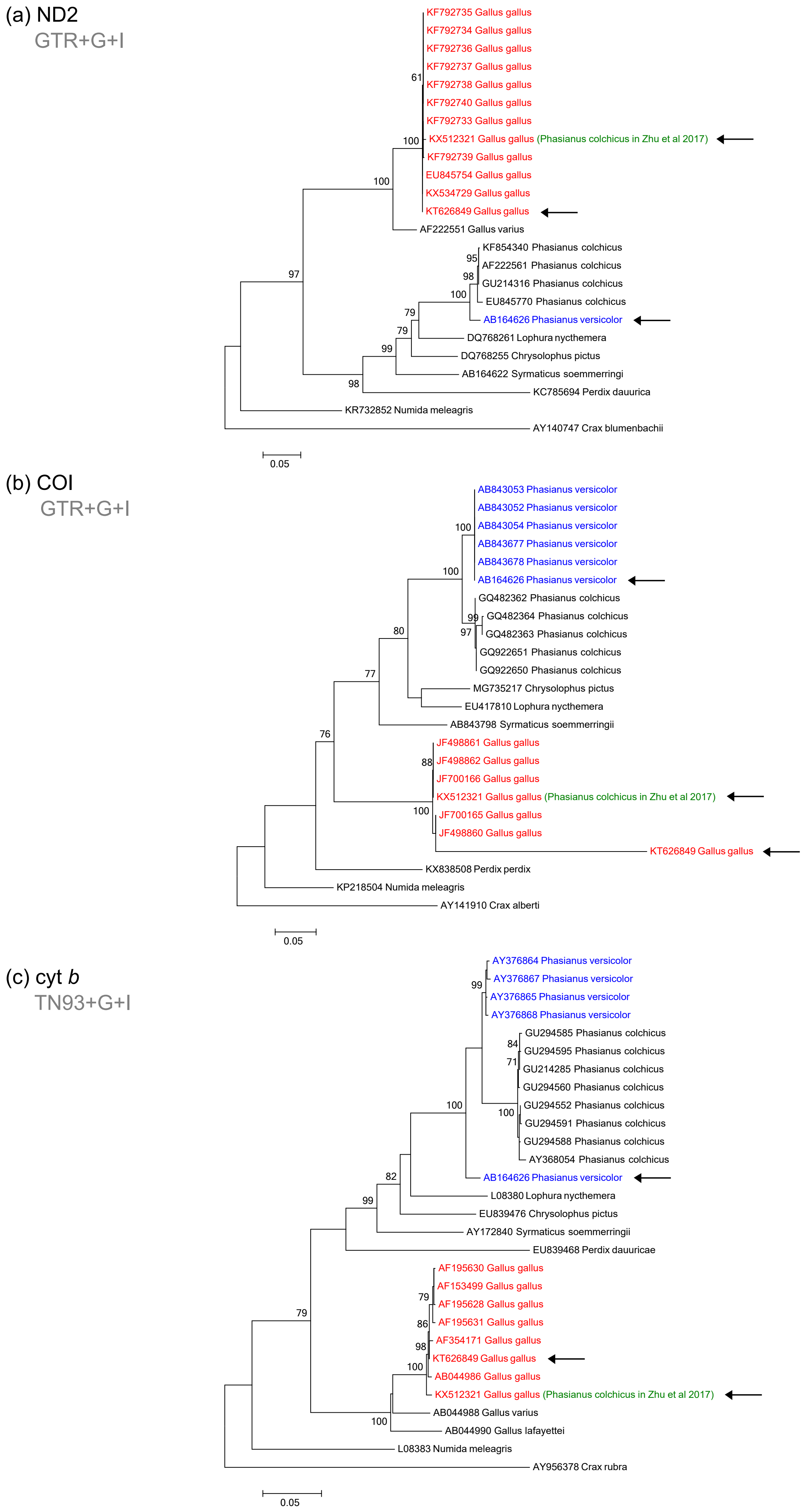

Figure S3. Maximum Likelihood phylogenies of *Phasianus versicolor* (AB164626), *Phasianus colchicus* (KX512321), *Gallus gallus* (KT626849) and related taxa based on mitochondrial sequences. Numbers at branches are bootstrap support values (>70%) based on 1000 replicates.

6. “*Branta bernicla*” KJ680301 (Lee, Y.J., Ryu, S.H. and Hwang, U.W., unpublished; 2014)

Fig. S4

Metadata: none on GenBank
Phylogenetic position:
ND2 = *Branta bernicla*
CO1 = *Branta bernicla*
Cyt *b* = *Branta bernicla* but on a very long branch.
Sequence integrity: cyt *b* problematic sequence. Bp 1 to 1008 are correct (*B. bernicla*). Bp 1009 – 1921 are a duplicate of bp 232 – 1143 of cyt *b* of *B. bernicla*.
Interpretation: poor assembly resulting in partial duplication of cytochrome b.
Subsequent usage:
Re-used in **5 mitogenomic phylogenies published before 1 January 2020**: Park et al. 2016a, Park et al. 2016b, Mereu et al. 2017, Sun et al. 2017a, Buckner et al. 2018.
Problems noted in other works: none.
Relevance: This was the only mitogenome presumed to be of this species (January 2020).
References:

Buckner, JC, Ellingson, R, Gold, DA, Jones, TL & Jacobs, DK 2018. Mitogenomics supports an unexpected taxonomic relationship for the extinct diving duck *Chendytes lawi* and definitively places the extinct Labrador Duck. Molecular Phylogenetics and Evolution 122: 102-109.

Mereu, P, Satta, V, Frongia, GN, Berlinguer, F, Muzzeddu, M, Campus, A, Decandia, L, Pirastru, M, Manca, L, Naitana, S & Leoni, GG 2017. The complete mtDNA sequence of the griffon vulture (*Gyps fulvus*): Phylogenetic analysis and haplotype frequency variations after restocking in the Sardinian population. Biological Conservation 214: 195-205.

Park, CE, Park, GS, Kwak, Y, Hong, SJ, Khan, AR, Jung, BK, Park, YJ, Kim, JG, Park, HC & Shin, JH 2016a. Complete mitochondrial genome of *Cygnus cygnus* (Aves, Anseriformes, Anatidae). Mitochondrial DNA Part A 27: 2907-2908.

Park, CE, Park, GS, Kwak, Y, Hong, SJ, Khan, AR, Jung, BK, Park, YJ, Kim, JG, Park, HC & Shin, JH 2016b. Complete mitochondrial genome of *Cygnus olor* (Aves, Anseriformes, Anatidae). Mitochondrial DNA Part A 27: 3442-3443.

Sun, Z, Pan, T, Hu, C, Sun, L, Ding, H, Wang, H, Zhang, C, Jin, H, Chang, Q, Kan, X & Zhang, B 2017a. Rapid and recent diversification patterns in Anseriformes birds: Inferred from molecular phylogeny and diversification analyses. PloS one 12(9), e0184529.

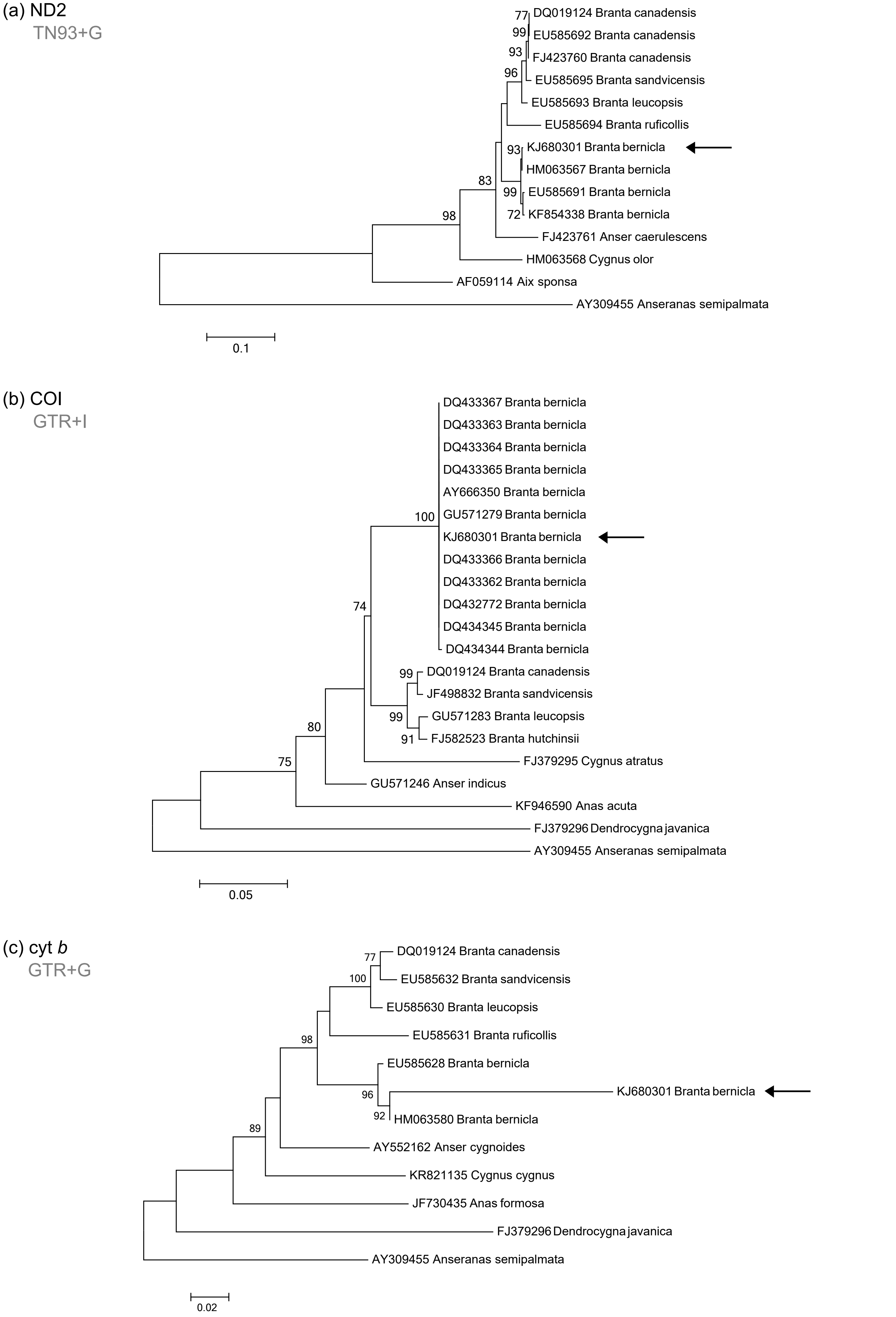

Figure S4. Maximum Likelihood phylogenies of *Branta bernicla* (KJ680301) and related taxa based on mitochondrial sequences. Numbers at branches are bootstrap support values (>70%) based on 1000 replicates.

7. “*Anser fabalis*” HQ890328, NC_016922 (Liu et al. 2013)

Fig. S5

Metadata: “A Bean goose (*Anser fabalis*) injured by power lines was found in the wild in the Shengjin Lake National Nature Reserve, Anhui, China in October 2010. The bird was treated for its injuries but later died (...). Tissues from the dead bird were field–Stored at -20 °C at the Institute of Biodiversity and Wetland Ecology, Anhui University.” (Liu et al. 2013: 8-9)

Phylogenetic position:

ND2 = Among *Anser*. Exact position could not be verified due to lack of structure.

CO1 = Among *Anser* on a long branch. Exact position could not be verified due to lack of structure.

Cyt *b* = *Anser fabalis*

Sequence integrity:

Cyt b: inferred gap at positions 24-35.

Examination of the full mitogenome showed that there are problems in three other genes as well. ND1: inferred gaps at positions 58, 69, 82, 100, 102, 112, 116, 143-148, 153, 258; and multiple unique substitutions (among Anatidae) at pos 160 – 245. COII: multiple unique substitutions (among Anatidae) at pos 52 – 118; ATP6: multiple unique substitutions (among Anatidae) at pos 252 – 423.

Interpretation: Sequencing errors / numts

Subsequent usage:

Re-used in **17 mitogenomic phylogenies published before 1 January 2020**: He et al. 2016b, Liu et al. 2014a, Mu et al. 2014, Dai et al. 2016, Sun et al. 2016a, Park et al. 2016a, Park et al. 2016b, Liu et al. 2016, Lee et al. 2017, Sun et al. 2017a, Zhang et al. 2017a, Lin et al. 2018, Liu et al. 2019b, Liu et al. 2019k, Lin et al. 2019a, Lin et al. 2019b, Lin et al. 2019c.

Cited by **1 further paper**: Souto et al. 2016.

Problems noted in other works: none. This sequence was on a long branch in multiple studies but not flagged as potentially problematic: Liu et al. 2014a (*Aix galericulata* and *Tadorna ferruginea*), He et al. 2016b (*Anas platyrhynchos*), Dai et al. 2016 (*Anser cygnoides*), Zhang et al. 2017 (*Anas crecca*), Lin et al. 2018 (*Anser cygnoides*), Lin et al. 2019a (*Anser cygnoides*), Lin et al. 2019b (*Anser cygnoides*).

Relevance: This was the only mitogenome presumed to be of this species (January 2020).

References:

Dai, QZ, Lin, Q & Jiang, GT 2016. Phylogenetic studies of four *Anser cygnoides* (Anserini: Anserinae) in Hunan province of China based on complete mitochondrial DNA sequences. Mitochondrial DNA 27: 2464-2465.

He, X, Lin, Q, Cao, R, Yuan, YT, Pan, DZ, Yun, L, Zhang, SR & Hou, DX 2016b. Phylogenetic studies of two *Anas platyrhynchos* (Anatini: Anatinae) in Hunan province of China based on complete mitochondrial DNA sequences. Mitochondrial DNA Part A 27: 2462-2463.

Lee, MY, Jeon, HS, Choi, YS, Joo, S & An, J 2017. Sequencing and analyzing complete mitochondrial genome of *Anser cygnoides* (Anserini: Anserinae). Mitochondrial DNA Part B 2: 228-229.

Lin, Q, Jiang, GT & Dai, QZ 2018. The complete mitochondrial genome of the *Anser cygnoides* Linnaeus, 1758 breed Daozhou and its phylogenetic analyses. Russian Journal of Genetics 54: 1493-1497.

Lin, Q, Jiang, GT, Dai, QZ & Li, C 2019a. The complete mitochondrial genome of the Mayang white goose and its phylogenetic analyses. Mitochondrial DNA Part B 4: 664-665.

Lin, Q, Jiang, GT, Dai, QZ, Zhang, X, Huang, X & Li, C 2019b. The complete mitochondrial genome of the Landes goose and its phylogenetic analyses. Mitochondrial DNA Part B 4: 904-905.

Lin, Q, Jiang, GT, Dai, QZ, Zhang, X, Huang, X & Li, C 2019c. The complete mitochondrial genome of the Sichuan white goose and its phylogenetic analyses. Mitochondrial DNA Part B 4: 754-755.

Liu, CZ, Wei, GH, Hu, JH & Liu, XY 2016. Complete mitochondrial genome of the Swan Goose (*Anser cygnoides* L.) and its phylogenetic analysis. Mitochondrial DNA A 27: 2427-8.

Liu, F, Antalffy, J & Wen, L 2019. The complete mitochondrial genome sequences of two *Emberiza* (Aves, Passeriformes). Mitochondrial DNA Part B 4: 914-915.

Liu, G, Li, Q & Gong, Z 2019k. The complete mitochondrial genome of common Shelduck Shengjin Lake *Tadorna tadorna*. Mitochondrial DNA Part B 4: 3060-3061.

Liu, G, Zhou, L, Li, B & Zhang, L 2014a. The complete mitochondrial genome of *Aix galericulata* and *Tadorna ferruginea*: bearings on their phylogenetic position in the Anseriformes. PloS one 9(11), e109701.

Liu, G, Zhou, L, Zhang, L, Luo, Z & Xu, W 2013. The complete mitochondrial genome of Bean Goose (*Anser fabalis*) and implications for Anseriformes taxonomy. PLoS ONE 8(5): e63334.

Mu, CY, Huang, ZY, Chen, Y, Wang, B, Su, YH, Li, Y, Sun, ZM, Xu, Q, Zhao, WM & Chen, GH 2014. [Complete sequence and gene organization of the *Anser cygnoides* mitochondrial genome.] Journal of Agricultural Biotechnology 22: 1482-1493. [In Chinese.]

Park, CE, Park, GS, Kwak, Y, Hong, SJ, Khan, AR, Jung, BK, Park, YJ, Kim, JG, Park, HC & Shin, JH 2016a. Complete mitochondrial genome of *Cygnus cygnus* (Aves, Anseriformes, Anatidae). Mitochondrial DNA Part A 27: 2907-2908.

Park, CE, Park, GS, Kwak, Y, Hong, SJ, Khan, AR, Jung, BK, Park, YJ, Kim, JG, Park, HC & Shin, JH 2016b. Complete mitochondrial genome of *Cygnus olor* (Aves, Anseriformes, Anatidae). Mitochondrial DNA Part A 27: 3442-3443.

Souto, HM, Ruschi, PA, Furtado, C, Jennings, WB & Prosdocimi, F 2016. The complete mitochondrial genome of the ruby-topaz hummingbird *Chrysolampis mosquitus* through Illumina sequencing. Mitochondrial DNA Part A 27: 769-770.

Sun, Z, Wang, B, Sun, X, Yan, L, Pan, T & Zhang, B 2016a. Phylogenetic studies of *Anas clypeata* (Anatidae: Anas) based on complete mitochondrial DNA sequences. Mitochondrial DNA A 27: 4320-4321.

Sun, Z, Pan, T, Hu, C, Sun, L, Ding, H, Wang, H, Zhang, C, Jin, H, Chang, Q, Kan, X & Zhang, B 2017a. Rapid and recent diversification patterns in Anseriformes birds: Inferred from molecular phylogeny and diversification analyses. PloS one 12(9), e0184529.

Zhang, Q, Wang, Y, Chen, R, Liu, B & Kan, X 2017a. The complete mitochondrial genome of *Anas crecca* (Anseriformes: Anatidae). Mitochondrial DNA Part B 2: 352-353.

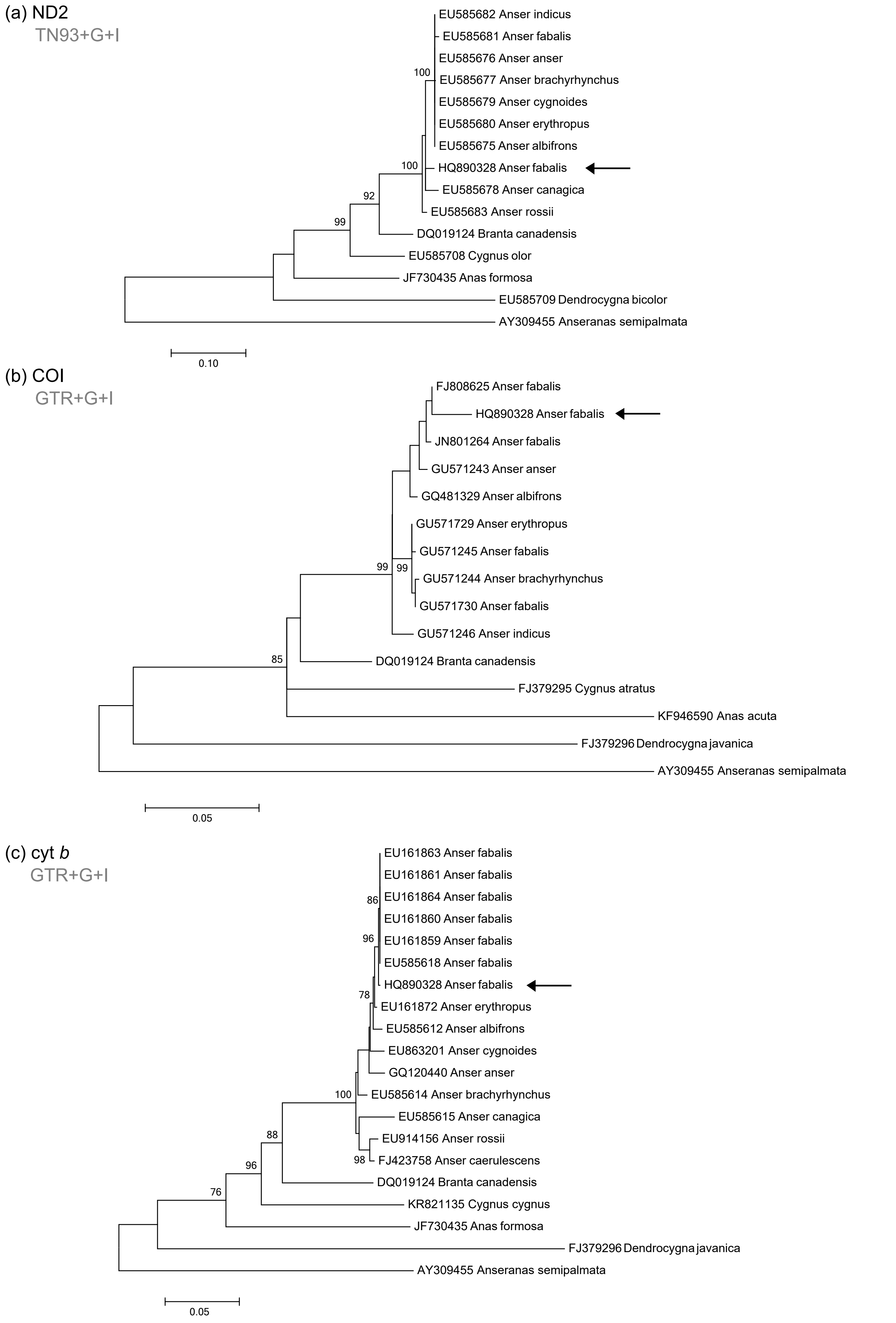

Figure S5. Maximum Likelihood phylogenies of *Anser fabalis* (HQ890328) and related taxa based on mitochondrial sequences. Numbers at branches are bootstrap support values (>70%) based on 1000 replicates.

8. “*Tadorna tadorna*” KJ794187, NC_024750 (Lin et al. 2016a)

Fig. S6

Metadata: “The duck was farmed at Hunan institute of animal science and veterinary medicine (Changsha City, Hunan Province, China).” (Lin et al. 2016a: 992).
Phylogenetic position:
ND2 = among *Anas platyrhynchos* and several other *Anas* species
CO1 = among *Anas platyrhynchos* and several other *Anas* species
Cyt *b* = among *Anas platyrhynchos, A. zonorhyncha, A. poecilorhyncha*
Sequence integrity: not investigated
Interpretation: Correctly identified in Lin et al. (2016a) as *A. platyrhynchos*, but incorrectly listed on GenBank (as *Tadorna tadorna*).
Subsequent usage:
Re-used in **2 mitogenomic phylogenies published before 1 January 2020**: Park et al. 2016a (as “*Tadorna tadorna*”), Park et al. 2016b (as “*Tadorna tadorna*”).
Problems noted in other works: none.
Relevance: A valid mitogenome (KU140668) of this species was published by Sun et al. (2017a).
References:

Lin, Q, Jiang, GT, Yun, L, Li, GJ, Dai, QZ, Zhang, SR, Hou, DX & He, X 2016a. The complete mitochondrial genome of the Linwu duck. Mitochondrial DNA Part A 27: 992-993.

Park, CE, Park, GS, Kwak, Y, Hong, SJ, Khan, AR, Jung, BK, Park, YJ, Kim, JG, Park, HC & Shin, JH 2016a. Complete mitochondrial genome of *Cygnus cygnus* (Aves, Anseriformes, Anatidae). Mitochondrial DNA Part A 27: 2907-2908.

Park, CE, Park, GS, Kwak, Y, Hong, SJ, Khan, AR, Jung, BK, Park, YJ, Kim, JG, Park, HC & Shin, JH 2016b. Complete mitochondrial genome of *Cygnus olor* (Aves, Anseriformes, Anatidae). Mitochondrial DNA Part A 27: 3442-3443.

Sun, Z, Pan, T, Hu, C, Sun, L, Ding, H, Wang, H, Zhang, C, Jin, H, Chang, Q, Kan, X & Zhang, B 2017a. Rapid and recent diversification patterns in Anseriformes birds: Inferred from molecular phylogeny and diversification analyses. PloS one 12(9), e0184529.

9. “*Tadorna tadorna*” MN258348 (Liu et al. 2019k)

Fig. S6

Metadata: “The feather samples were collected from Tadorna tadorna rescued in Shengjin Lake National Nature Reserve (E117º0’42.55’’, N30° 20’30.73’’), Anhui Province of China in October 2017.” (Liu et al. 2019k: 3060)

Phylogenetic position:

ND2 = *Tadorna tadorna*

CO1 = *Tadorna tadorna* but on a long branch

Cyt *b* = *Tadorna tadorna* but on a long branch

Sequence integrity: CO1: MN258348 was 0.8% divergent from 10 other *Tadorna tadorna*. This sequence shows 6 nucleotide substitutions that are unique not only among *Tadorna tadorna* but among all >800 COI sequences of Anatidae (192C [all Anatidae: G], 257A [all Anatidae: T], 326C [all Anatidae: T], 327A [all Anatidae: G], 507C [all Anatidae: G], 657C [all Anatidae: G, except one that has A]).

Cyt b: MN258348 was 1.6-2.3% divergent from two other *T. tadorna*. The pattern of mutations relative to these two *T. tadorna* is suggestive of numts: 6 mutations at 1st position, 9 mutations at 2nd position, 4 mutations at 3rd position.

Interpretation: Sequencing errors / numts

Subsequent usage: not used in any further studies (January 2020)

Problems noted in other works: none.

Relevance: A valid mitogenome (KU140668) of this species was published by Sun et al. (2017a).

References:

Liu, G, Li, Q & Gong, Z 2019k. The complete mitochondrial genome of common Shelduck Shengjin Lake *Tadorna tadorna*. Mitochondrial DNA Part B 4: 3060-3061.

Sun, Z, Pan, T, Hu, C, Sun, L, Ding, H, Wang, H, Zhang, C, Jin, H, Chang, Q, Kan, X & Zhang, B 2017a. Rapid and recent diversification patterns in Anseriformes birds: Inferred from molecular phylogeny and diversification analyses. PloS One 12(9), e0184529.

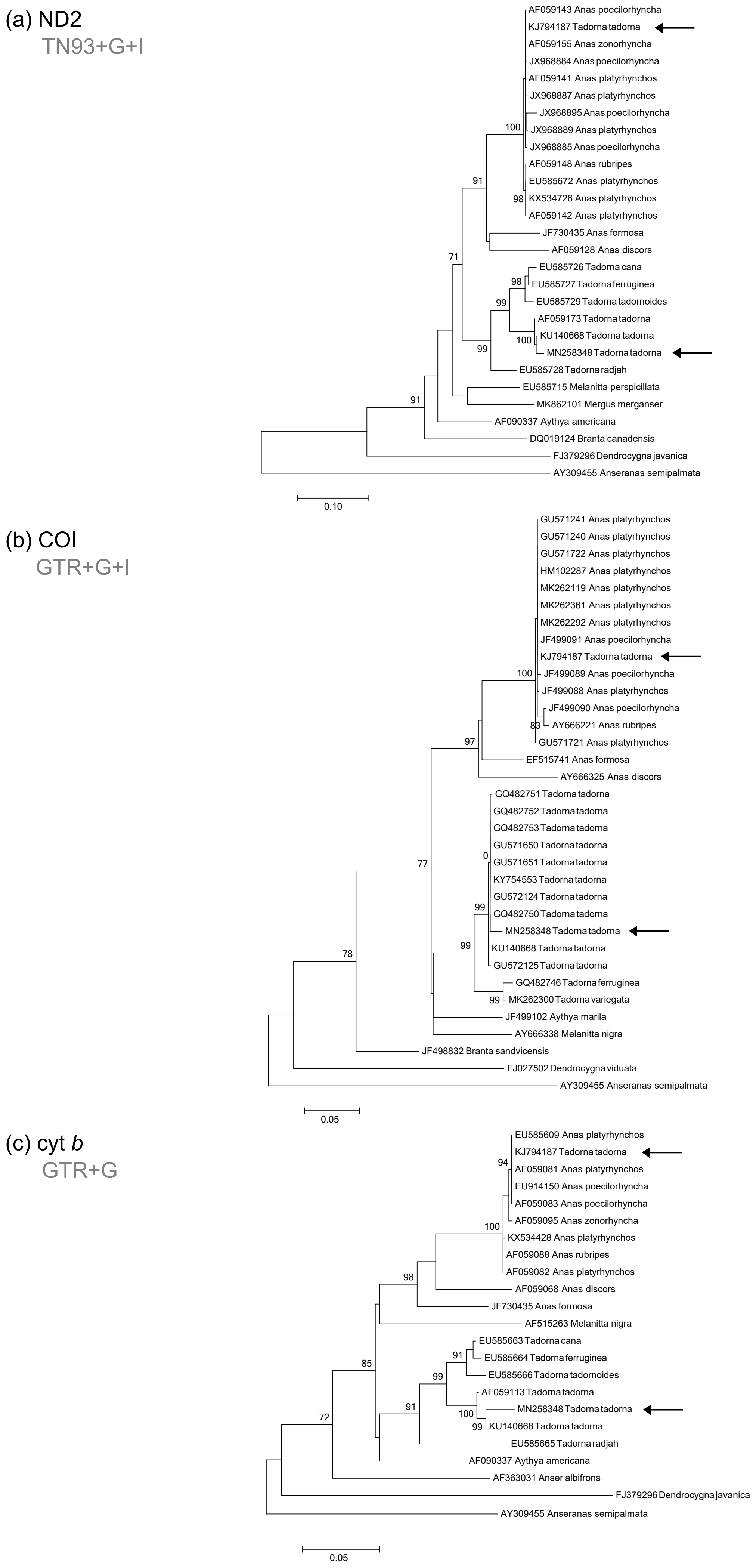

Figure S6. Maximum Likelihood phylogenies of *Tadorna tadorna* (KJ794187, MN258348) and related taxa based on mitochondrial sequences. Numbers at branches are bootstrap support values (>70%) based on 1000 replicates.

10. “*Aix galericulata*” KJ169568, NC_023969 (Meng et al. 2016a)

Fig. S7

Metadata: no information in Meng et al. (2016a) or on GenBank.
Phylogenetic position:
ND2 = *Aix galericulata*
CO1 = *Aix galericulata*
Cyt *b* = sister to two other *Aix galericulata* but with deep divergence
Sequence integrity: cyt b: bp 18-585 identical to two *Aythya ferina* (KJ710708, EU585623)
Interpretation: Chimera
Subsequent usage:
Re-used in **5 mitogenomic phylogenies published before 1 January 2020**: Nabholz et al. 2016, Sun et al. 2016a, Sun et al. 2017a, Zhang et al. 2017a, Buckner et al. 2018.
Problems noted in other works: none.
Relevance: Another mitogenome of this species (KF437906) was published by Liu et al. 2014a).
References:

Buckner, JC, Ellingson, R, Gold, DA, Jones, TL & Jacobs, DK 2018. Mitogenomics supports an unexpected taxonomic relationship for the extinct diving duck *Chendytes lawi* and definitively places the extinct Labrador Duck. Molecular Phylogenetics and Evolution 122: 102-109.

Liu, G, Zhou, L, Li, B & Zhang, L 2014a. The complete mitochondrial genome of *Aix galericulata* and *Tadorna ferruginea*: bearings on their phylogenetic position in the Anseriformes. PloS One 9(11), e109701.

Meng, S, Wu, Z, Pan, T, Yan, L, Bei, Y, Li, G & Zhang, B 2016. Mitochondrial genome of the *Aix galericula* (Anatidae: *Aix*). Mitochondrial DNA Part A 27: 318-319.

Nabholz, B, Lanfear, R & Fuchs, J 2016. Body mass-corrected molecular rate for bird mitochondrial DNA. Mol. Ecol. 25: 4438-4449.

Sun, Z, Wang, B, Sun, X, Yan, L, Pan, T & Zhang, B 2016a. Phylogenetic studies of *Anas clypeata* (Anatidae: Anas) based on complete mitochondrial DNA sequences. Mitochondrial DNA A 27: 4320-4321.

Sun, Z, Pan, T, Hu, C, Sun, L, Ding, H, Wang, H, Zhang, C, Jin, H, Chang, Q, Kan, X & Zhang, B 2017a. Rapid and recent diversification patterns in Anseriformes birds: Inferred from molecular phylogeny and diversification analyses. PloS One 12(9), e0184529.

Zhang, Q, Wang, Y, Chen, R, Liu, B & Kan, X 2017a. The complete mitochondrial genome of *Anas crecca* (Anseriformes: Anatidae). Mitochondrial DNA Part B 2: 352-353.

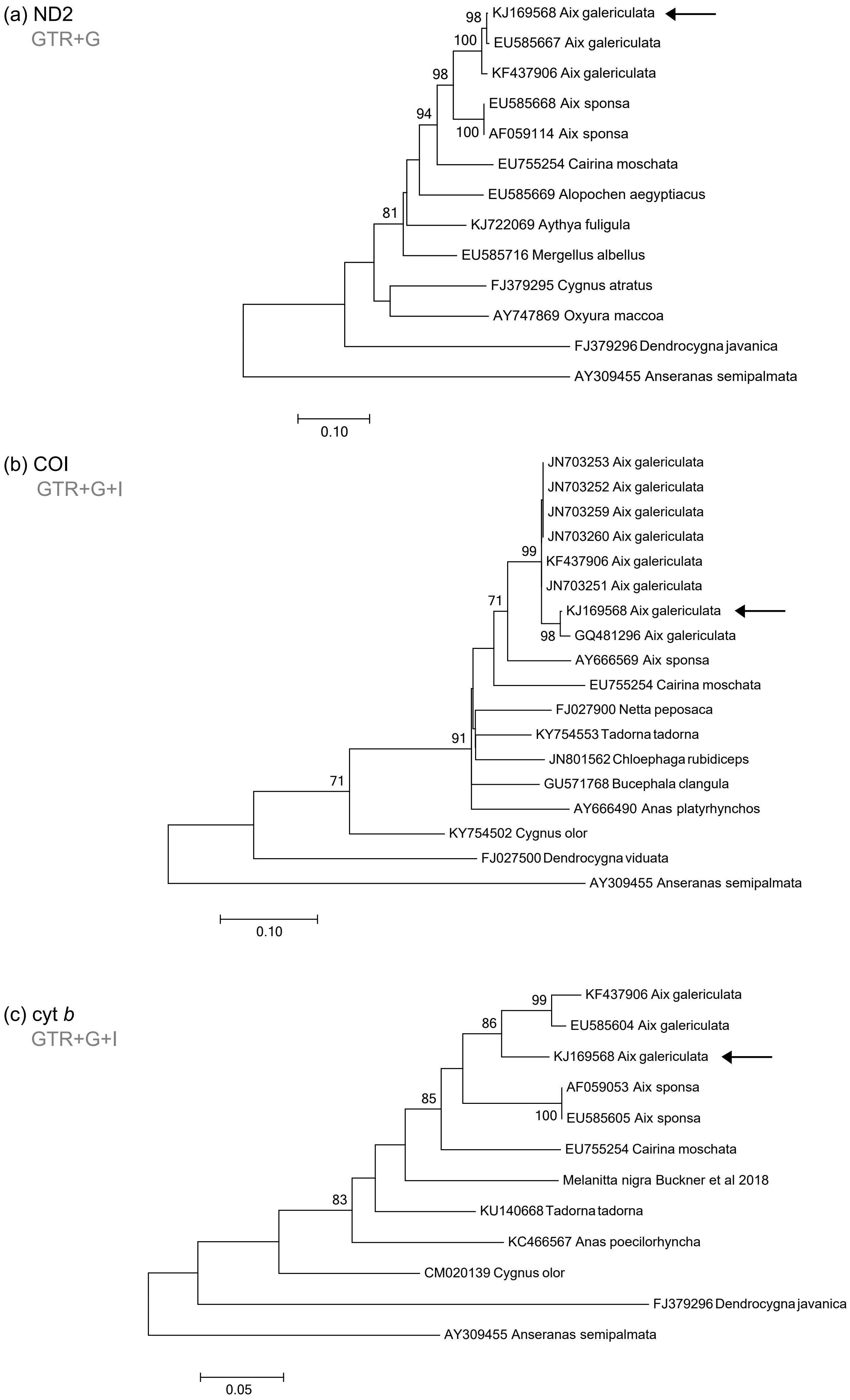

Figure S7. Maximum Likelihood phylogenies of *Aix galericulata* (KJ169568) and related taxa based on mitochondrial sequences. Numbers at branches are bootstrap support values (>70%) based on 1000 replicates.

11. “*Anas crecca*” KF203133, NC_022452 (Hu et al. 2015)

Fig. S8

Metadata: no information in Hu et al. (2015) or on GenBank.
Phylogenetic position:
ND2 = *Anas crecca*
CO1 = *Anas crecca*
Cyt *b* = sister to *Anas crecca* with a deep divergence
Sequence integrity: cyt b: 19 singleton sites in bp 801-1134. The fragment did not closely match that of any other sequence (BLAST; distance >4% to the closest sequence).
Interpretation: Sequencing errors / numts
Subsequent usage:
Re-used in **14 mitogenomic phylogenies published before 1 January 2020**: Dai et al. 2016, He et al. 2016b, Nabholz et al. 2016, Park et al. 2016a, Park et al. 2016b, Sun et al. 2016a, Sun et al. 2017a, Buckner et al. 2018, Lin et al. 2018, Lee et al. 2019, Lin et al. 2019a, Lin et al. 2019b, Lin et al. 2019c, Wei et al. 2019.
Problems noted in other works: none.
Relevance: Another mitogenome was published by Zhang et al. (2017a).
References:

Buckner, JC, Ellingson, R, Gold, DA, Jones, TL & Jacobs, DK 2018. Mitogenomics supports an unexpected taxonomic relationship for the extinct diving duck *Chendytes lawi* and definitively places the extinct Labrador Duck. Molecular Phylogenetics and Evolution 122: 102-109.

Dai, QZ, Lin, Q & Jiang, GT 2016. Phylogenetic studies of four *Anser cygnoides* (Anserini: Anserinae) in Hunan province of China based on complete mitochondrial DNA sequences. Mitochondrial DNA 27: 2464-2465.

He, X, Lin, Q, Cao, R, Yuan, YT, Pan, DZ, Yun, L, Zhang, SR & Hou, DX 2016b. Phylogenetic studies of two *Anas platyrhynchos* (Anatini: Anatinae) in Hunan province of China based on complete mitochondrial DNA sequences. Mitochondrial DNA Part A 27: 2462-2463.

Hu, C, Chang Q, Zhou W, Yan L, Pan T, Xue C, Zhang B 2015. Mitochondrial genome of the Anas crecca (Anatidae: Anas). Mitochondrial DNA 26: 625-626.

Lee, S-M, Jeon, HS, Kim, JA, Kim, J, Park, J & Kil, H-J 2019. The mitochondrial genome of the goosander (*Mergus merganser*) determined using next-generation sequencing. Mitochondrial DNA Part B 4: 2547-2548.

Lin, Q, Jiang, GT & Dai, QZ 2018. The complete mitochondrial genome of the *Anser cygnoides* Linnaeus, 1758 breed Daozhou and its phylogenetic analyses. Russian Journal of Genetics 54: 1493-1497.

Lin, Q, Jiang, GT, Dai, QZ & Li, C 2019a. The complete mitochondrial genome of the Mayang white goose and its phylogenetic analyses. Mitochondrial DNA Part B 4: 664-665.

Lin, Q, Jiang, GT, Dai, QZ, Zhang, X, Huang, X & Li, C 2019b. The complete mitochondrial genome of the Landes goose and its phylogenetic analyses. Mitochondrial DNA Part B 4: 904-905.

Lin, Q, Jiang, GT, Dai, QZ, Zhang, X, Huang, X & Li, C 2019c. The complete mitochondrial genome of the Sichuan white goose and its phylogenetic analyses. Mitochondrial DNA Part B 4: 754-755.

Nabholz, B, Lanfear, R & Fuchs, J 2016. Body mass-corrected molecular rate for bird mitochondrial DNA. Mol. Ecol. 25: 4438-4449.

Park, CE, Park, GS, Kwak, Y, Hong, SJ, Khan, AR, Jung, BK, Park, YJ, Kim, JG, Park, HC & Shin, JH 2016a. Complete mitochondrial genome of *Cygnus cygnus* (Aves, Anseriformes, Anatidae). Mitochondrial DNA Part A 27: 2907-2908.

Park, CE, Park, GS, Kwak, Y, Hong, SJ, Khan, AR, Jung, BK, Park, YJ, Kim, JG, Park, HC & Shin, JH 2016b. Complete mitochondrial genome of *Cygnus olor* (Aves, Anseriformes, Anatidae). Mitochondrial DNA Part A 27: 3442-3443.

Sun, Z, Pan, T, Hu, C, Sun, L, Ding, H, Wang, H, Zhang, C, Jin, H, Chang, Q, Kan, X & Zhang, B 2017a. Rapid and recent diversification patterns in Anseriformes birds: Inferred from molecular phylogeny and diversification analyses. PloS one 12(9), e0184529.

Sun, Z, Wang, B, Sun, X, Yan, L, Pan, T & Zhang, B 2016a. Phylogenetic studies of *Anas clypeata* (Anatidae: Anas) based on complete mitochondrial DNA sequences. Mitochondrial DNA A 27: 4320-4321.

Wei, Q, Wu, X, Hu, Y, Sun, G, Chen, J, Gao, X, Sha, W & Zhang, H 2019. The complete mitochondrial genome of the gadwall (*Anas strepera*). Mitochondrial DNA Part B 4: 3141-3142.

Zhang, Q, Wang, Y, Chen, R, Liu, B & Kan, X 2017a. The complete mitochondrial genome of *Anas crecca* (Anseriformes: Anatidae). Mitochondrial DNA Part B 2: 352-353.

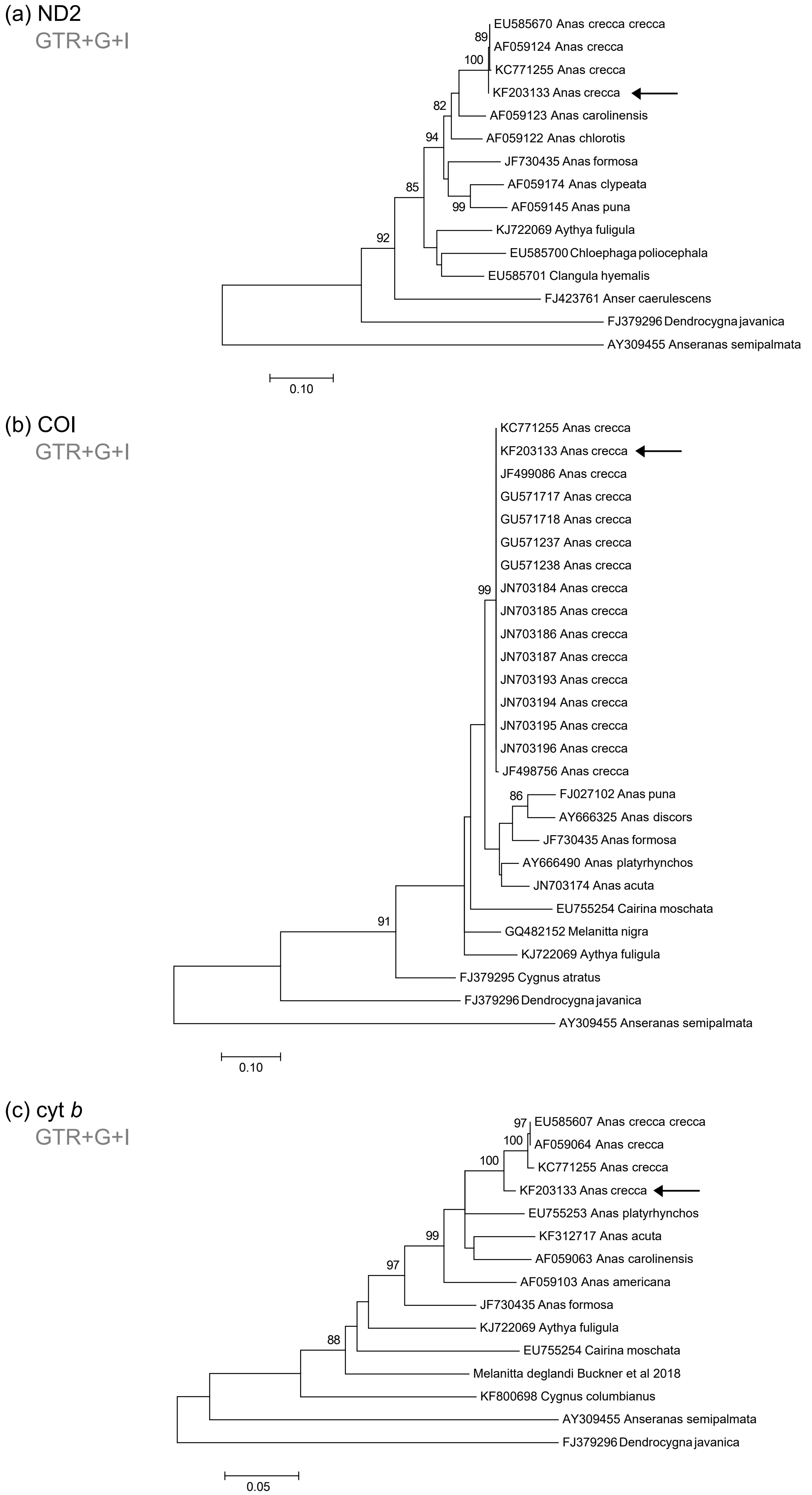

Figure S8. Maximum Likelihood phylogenies of *Anas crecca* (KF203133) and related taxa based on mitochondrial sequences. Numbers at branches are bootstrap support values (>70%) based on 1000 replicates.

12. “*Anas clypeata*” KT345702, NC_028346 (Sun et al. 2016a)

Fig. S9

Metadata: no information in Sun et al. (2016a) or on GenBank.
Phylogenetic position:
ND2 = *Anas clypeata*
CO1 = *Anas clypeata*
Cyt *b* = sister to a clade formed by *Anas clypeata* (n=3)), *Anas smithii* (n=1), *Anas rhynchotus* (n=1), *Anas platalea* (n=1)
Sequence integrity: cyt b: 26 singleton sites in bp 448-1062 (mostly between bp 713 and 1062). The fragment from bp 713-1062 did not closely match that of any other sequence (BLAST; distance >5% to the closest sequence).
Interpretation: Sequencing errors / numts
Subsequent usage:
Re-used in **3 mitogenomic phylogenies published before 1 January 2020**: Sun et al. 2017a, Buckner et al. 2018, Lee et al. 2019.
Also cited by **1 other papers**: Shao et al. 2019 (diet identification of carnivores using 12S rRNA)
Problems noted in other works: none.
Relevance: This was the only mitogenome presumed to be of this species (January 2020).
References:

Buckner, JC, Ellingson, R, Gold, DA, Jones, TL & Jacobs, DK 2018. Mitogenomics supports an unexpected taxonomic relationship for the extinct diving duck *Chendytes lawi* and definitively places the extinct Labrador Duck. Molecular Phylogenetics and Evolution 122: 102-109.

Lee, S-M, Jeon, HS, Kim, JA, Kim, J, Park, J & Kil, H-J 2019. The mitochondrial genome of the goosander (*Mergus merganser*) determined using next-generation sequencing. Mitochondrial DNA Part B 4: 2547-2548.

Shao, X, Song, D, Huang, Q, Li, S & Yao, M 2019. Fast surveys and molecular diet analysis of carnivores based on fecal DNA and metabarcoding. Environmental Science 27: 543-556. doi: 10.17520/biods.2018214 [In Chinese.]

Sun, Z, Wang, B, Sun, X, Yan, L, Pan, T & Zhang, B 2016a. Phylogenetic studies of *Anas clypeata* (Anatidae: Anas) based on complete mitochondrial DNA sequences. Mitochondrial DNA A 27: 4320-4321.

Sun, Z, Pan, T, Hu, C, Sun, L, Ding, H, Wang, H, Zhang, C, Jin, H, Chang, Q, Kan, X & Zhang, B 2017a. Rapid and recent diversification patterns in Anseriformes birds: Inferred from molecular phylogeny and diversification analyses. PloS One 12(9), e0184529.

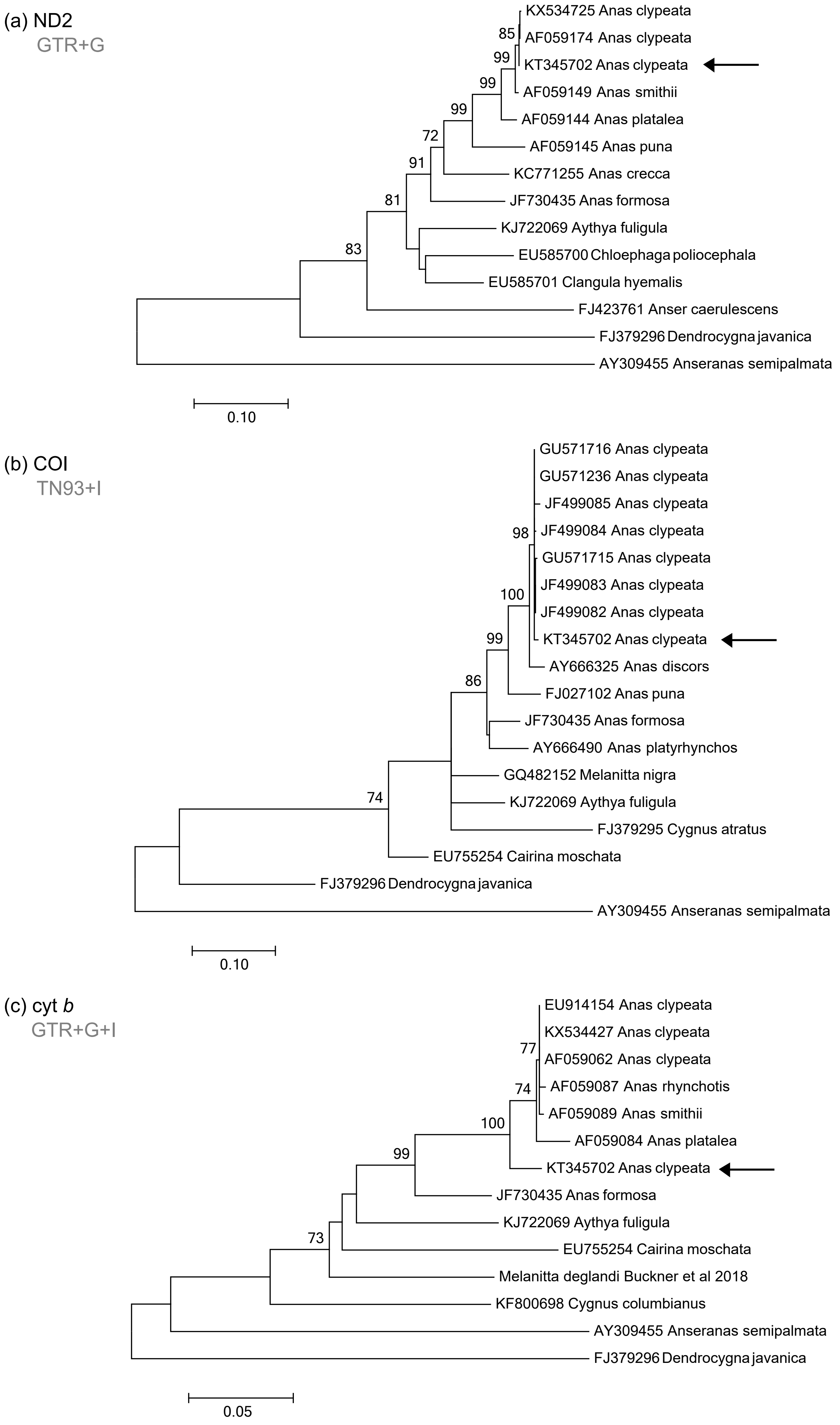

Figure S9. Maximum Likelihood phylogenies of *Anas clypeata* (KT345702) and related taxa based on mitochondrial sequences. Numbers at branches are bootstrap support values (>70%) based on 1000 replicates.

13. "*Anas falcata*" KC759527, NC_023352 (Pan et al. 2014)

Fig. S10

Metadata: No information in Pan et al. (2014) or on GenBank.
Phylogenetic position:
ND2 = *Anas penelope*
CO1 = *Anas penelope*
Cyt *b* = *Anas penelope*
Sequence integrity: not investigated
Interpretation: There are three possibilities: (i) the sequence comes from a misidentified *A. penelope*; (ii) a hybrid male *falcata* x female *penelope*, or (iii) an introgressed bird. Hybrids of *Anas falcata* and *Anas penelope* are known from the wild (McCarthy 2006).
Subsequent usage:
Re-used in **15 mitogenomic phylogenies published before 1 January 2020**: Mu et al. 2014, Luo et al. 2016, He et al. 2016, Park et al. 2016a, Park et al. 2016b, Nabholz et al. 2016, Zhang et al. 2017a, Sun et al. 2017a (PLOS ONE), Buckner et al. 2018, Fu et al. 2018, Lin et al. 2018, Lee et al. 2019, Lin et al. 2019a, Lin et al. 2019b, Lin et al. 2019c.
Also cited by **6 other papers**: Ksepka & Phillips 2015 [**used for molecular dating**], Zhou et al. 2015a, Meng et al. 2016, Sun et al. 2016, Zhou et al. 2016, Wen & Liao 2016.
Problems noted in other works: none.
Relevance: This was the only mitogenome presumed to be of this species (January 2020).
References:

Buckner, JC, Ellingson, R, Gold, DA, Jones, TL & Jacobs, DK 2018. Mitogenomics supports an unexpected taxonomic relationship for the extinct diving duck *Chendytes lawi* and definitively places the extinct Labrador Duck. Molecular Phylogenetics and Evolution 122: 102-109.

Fu, R, Dong, Y, Xuan, J, Liu, G, Xiang, X & Zhou, L 2018. Complete mitochondrial genome of *Anser albifrons frontails* [sic] (Anseriformes: Anatidae). Mitochondrial DNA Part B 3: 796-797.

He, X, Lin, Q, Cao, R, Yuan, YT, Pan, DZ, Yun, L, Zhang, SR & Hou, DX 2016. Phylogenetic studies of two *Anas platyrhynchos* (Anatini: Anatinae) in Hunan province of China based on complete mitochondrial DNA sequences. Mitochondrial DNA Part A 27: 2462-2463.

Ksepka, DT & Phillips, MJ 2015. Avian diversification patterns across the K-Pg boundary: Influence of calibrations, datasets, and model misspecification. Annals of the Missouri Botanical Garden 100: 300-328.

Lee, S-M, Jeon, HS, Kim, JA, Kim, J, Park, J & Kil, H-J 2019. The mitochondrial genome of the goosander (*Mergus merganser*) determined using next-generation sequencing. Mitochondrial DNA Part B 4: 2547-2548.

Lin, Q, Jiang, GT & Dai, QZ 2018. The complete mitochondrial genome of the *Anser cygnoides* Linnaeus, 1758 breed Daozhou and its phylogenetic analyses. Russian Journal of Genetics 54: 1493-1497.

Lin, Q, Jiang, GT, Dai, QZ & Li, C 2019a. The complete mitochondrial genome of the Mayang white goose and its phylogenetic analyses. Mitochondrial DNA Part B 4: 664-665.

Lin, Q, Jiang, GT, Dai, QZ, Zhang, X, Huang, X & Li, C 2019b. The complete mitochondrial genome of the Landes goose and its phylogenetic analyses. Mitochondrial DNA Part B 4: 904-905.

Lin, Q, Jiang, GT, Dai, QZ, Zhang, X, Huang, X & Li, C 2019c. The complete mitochondrial genome of the Sichuan white goose and its phylogenetic analyses. Mitochondrial DNA Part B 4: 754-755.

Luo, X, Kang, X & Zhang, D 2016. Complete mitochondrial genome of the American flamingo, *Phoenicopterus ruber* (Phoenicopteriformes, Phoenicopteridae). Mitochondrial DNA Part A 27: 3519-3520.

McCarthy, EM 2006. Handbook of Avian Hybrids of the World. Oxford, UK: Oxford University Press.

Meng, S, Wu, Z, Pan, T, Yan, L, Bei, Y, Li, G & Zhang, B 2016. Mitochondrial genome of the *Aix galericula* (Anatidae: *Aix*). Mitochondrial DNA Part A 27: 318-319.

Mu, CY, Huang, ZY, Chen, Y, Wang, B, Su, YH, Li, Y, Sun, ZM, Xu, Q, Zhao, WM & Chen, GH 2014. [Complete sequence and gene organization of the *Anser cygnoides* mitochondrial genome.] Journal of Agricultural Biotechnology 22: 1482-1493. [In Chinese.]

Nabholz, B, Lanfear, R & Fuchs, J 2016. Body mass-corrected molecular rate for bird mitochondrial DNA. Mol. Ecol. 25: 4438-4449.

Pan, T, Ren, L, Wang, H, Chen, J & Zhang, B 2014. Mitochondrial genome of the *Anas falcata* (Anatidae: *Anas*). Mitochondrial DNA 25: 111-112.

Park, CE, Park, GS, Kwak, Y, Hong, SJ, Khan, AR, Jung, BK, Park, YJ, Kim, JG, Park, HC & Shin, JH 2016a. Complete mitochondrial genome of *Cygnus cygnus* (Aves, Anseriformes, Anatidae). Mitochondrial DNA Part A 27: 2907-2908.

Park, CE, Park, GS, Kwak, Y, Hong, SJ, Khan, AR, Jung, BK, Park, YJ, Kim, JG, Park, HC & Shin, JH 2016b. Complete mitochondrial genome of *Cygnus olor* (Aves, Anseriformes, Anatidae). Mitochondrial DNA Part A 27: 3442-3443.

Sun, Z, Pan, T, Hu, C, Sun, L, Ding, H, Wang, H, Zhang, C, Jin, H, Chang, Q, Kan, X & Zhang, B 2017a. Rapid and recent diversification patterns in Anseriformes birds: Inferred from molecular phylogeny and diversification analyses. PloS one 12(9), e0184529.

Sun, Z, Wang, B, Sun, X, Yan, L, Pan, T & Zhang, B 2016. Phylogenetic studies of *Anas clypeata* (Anatidae: *Anas*) based on complete mitochondrial DNA sequences. Mitochondrial DNA A 27: 4320-4321.

Wen, L & Liao, F 2016. Complete mitochondrial genome of *Pycnonotus xanthorrhous* (Passeriformes, Pycnonotidae) and phylogenetic consideration. Biochemical Systematics and Ecology 69: 83-90.

Zhang, Q, Wang, Y, Chen, R, Liu, B & Kan, X 2017a. The complete mitochondrial genome of *Anas crecca* (Anseriformes: Anatidae). Mitochondrial DNA Part B 2: 352-353.

Zhou, W, Zhang C, Pan T, Yan L, Hu C, Xue C, Chang Q, Zhang B 2015a. The complete mitochondrial genome of *Anas poecilorhyncha* (Anatidae: Anas). Mitochondrial DNA 26: 265-266.

Zhou, W, Zhang, C, Chang, Q, Yan, L, Pan, T & Zhang, B 2016. The complete mitochondrial genome of *Aythya ferina* (Anatidae: *Aythya*). Mitochondrial DNA A 27: 968-969.

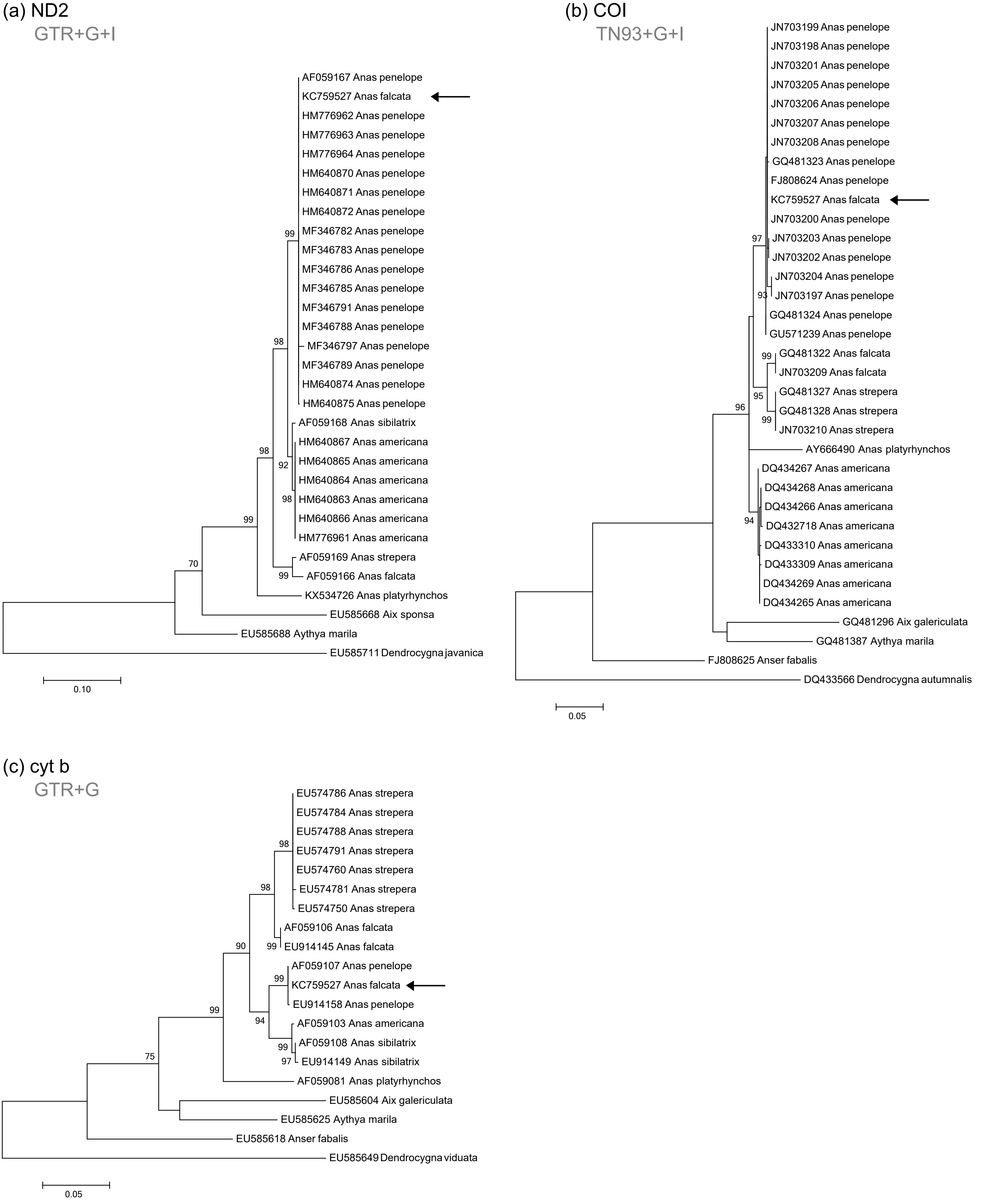

Figure S10. Maximum Likelihood phylogenies of *Anas falcata* (KC759527) and related taxa based on mitochondrial sequences. Numbers at branches are bootstrap support values (>70%) based on 1000 replicates.

14. "*Netta rufina*" KC466568, NC_024922 (Kan, X.-Z. and Li, X.-X., unpublished; 2013)

Fig. S11

Metadata: No information on GenBank.
Phylogenetic position:
ND2 = *Netta rufina*
CO1 = *Netta rufina* but on a very long branch
Cyt *b* = *Netta rufina*
Sequence integrity: COI: bp 436-696 included 41 singleton sites compared with 2 *N. rufina* and 7 *N. peposaca*; this fragment was identical to that of *Syrmaticus reevesi* (AB164623)
Interpretation: chimera
Subsequent usage:
Re-used in **6 mitogenomic mitogenomic phylogenies published before 1 January 2020**: Park et al. 2016a, Park et al. 2016b, Nabholz et al. 2016, Sun et al. 2017a, Buckner et al. 2018, Wei et al. 2019.
Also used in **1 other non-mitogenomic phylogeny**: Ericson et al. 2017 (*Rhodonessa*).
Problems noted in other works: none.
Relevance: This was the only mitogenome presumed to be of this species (January 2020).
References:

Buckner, JC, Ellingson, R, Gold, DA, Jones, TL & Jacobs, DK 2018. Mitogenomics supports an unexpected taxonomic relationship for the extinct diving duck *Chendytes lawi* and definitively places the extinct Labrador Duck. Molecular Phylogenetics and Evolution 122: 102-109.

Ericson, PGP, Qu, Y, Blom, MP, Johansson, US & Irestedt, M 2017. A genomic perspective of the pink-headed duck *Rhodonessa caryophyllacea* suggests a long history of low effective population size. Scientific Reports 7(1), 16853.

Nabholz, B, Lanfear, R & Fuchs, J 2016. Body mass-corrected molecular rate for bird mitochondrial DNA. Mol. Ecol. 25: 4438-4449.

Park, CE, Park, GS, Kwak, Y, Hong, SJ, Khan, AR, Jung, BK, Park, YJ, Kim, JG, Park, HC & Shin, JH 2016a. Complete mitochondrial genome of *Cygnus cygnus* (Aves, Anseriformes, Anatidae). Mitochondrial DNA Part A 27: 2907-2908.

Park, CE, Park, GS, Kwak, Y, Hong, SJ, Khan, AR, Jung, BK, Park, YJ, Kim, JG, Park, HC & Shin, JH 2016b. Complete mitochondrial genome of *Cygnus olor* (Aves, Anseriformes, Anatidae). Mitochondrial DNA Part A 27: 3442-3443.

Sun, Z, Pan, T, Hu, C, Sun, L, Ding, H, Wang, H, Zhang, C, Jin, H, Chang, Q, Kan, X & Zhang, B 2017a. Rapid and recent diversification patterns in Anseriformes birds: Inferred from molecular phylogeny and diversification analyses. PloS one 12(9), e0184529.

Wei, Q, Wu, X, Hu, Y, Sun, G, Chen, J, Gao, X, Sha, W & Zhang, H 2019. The complete mitochondrial genome of the gadwall (*Anas strepera*). Mitochondrial DNA Part B 4: 3141-3142.

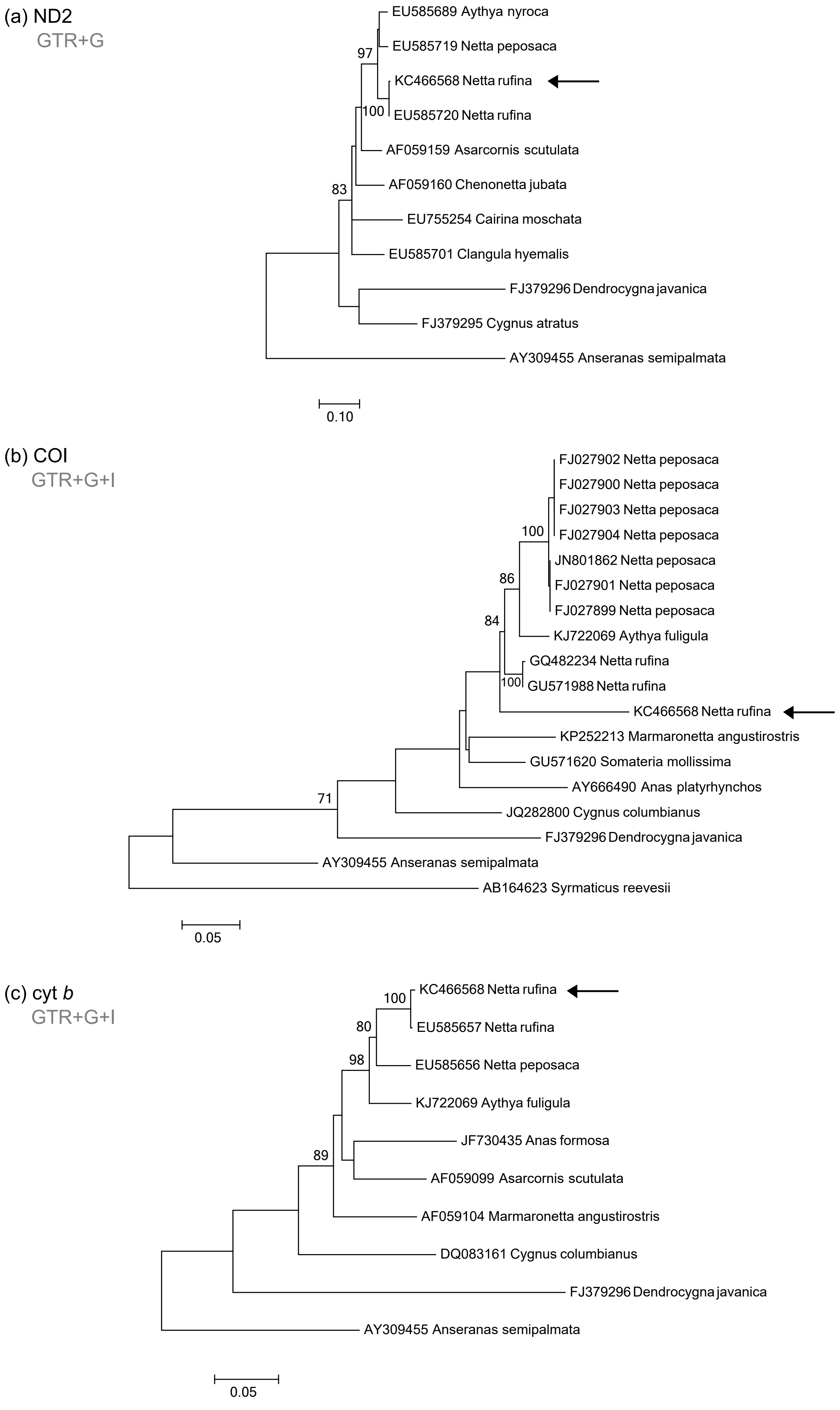

Figure S11. Maximum Likelihood phylogenies of *Netta rufina* (KC466568) and related taxa based on mitochondrial sequences. Numbers at branches are bootstrap support values (>70%) based on 1000 replicates.

15. “*Caprimulgus jotaka*” KM272749, NC_025773 (Zhao et al. 2016a); “*Caprimulgus indicus*” KM272749, NC_025773 (Liu et al. 2019a)

Fig. S12

Note: The same sequence was published twice, under different species names, and different GenBank numbers. However, NC_025773 is the same sequence as KM272749.

Metadata: “The samples were collected from the dead birds illegally hunted and transported, which was confiscated by Huangpu Mountain Forest Police station on 7 May 2013, Chuzhou City, Anhui Province, China.” (Zhao et al. 2016a: 1746). “The *C. indicus* sample was collected from a dead bird that was illegally hunted and transported, and was confiscated by Huangpu Mountain Forest Police Station on 7 May 2013, Chuzhou City, Anhui Province, China.” (Liu et al. 2019a: 8)

Phylogenetic position:

ND2 = *Otus semitorques* (Strigidae)

CO1 = *Otus semitorques* (Strigidae)

Cyt *b* = sister to all *Otus semitorques* (Strigidae), on a long branch

Sequence integrity: cyt *b* bp 1 – 420 = *Glaucidium cuculoides* [cf. two cross-validated sequences of *Glaucidium cuculoides*]; bp 429 – at least 1059 = *Otus semitorques*

ND2 has an insertion of ACTACC at position 976 (or ACACTA at position 971)

Interpretation: chimera of two owl species.

Subsequent usage:

Re-used in **5 mitogenomic phylogenies published before 1 January 2020**: Luo et al. 2016, Liu et al. 2017a, Xu et al. 2016b; Liu et al. 2019a, Qiu et al. 2019**.**

Re-used in **1 non-mitogenomic phylogeny**: Pellegrino et al. 2017 (*Apus pallidus*).

Problems noted in other works: Spiridonova & Surmach (2018) correctly pointed out that this is a chimeric sequence. A phylogeny in Xu et al. (2016b) placed NC_025773 among owls, but made no comments on this surprising result.

Relevance: This was the only mitogenome presumed to be of this species and the family Caprimulgidae (January 2020).

References:

Liu, G, Li, C, Du, Y & Liu, X 2017a. The complete mitochondrial genome of Japanese sparrowhawk (*Accipiter gularis*) and the phylogenetic relationships among some predatory birds. Biochemical Systematics and Ecology 70: 116-125.

Liu, G, Zhou, L & Gu, C 2014. The complete mitochondrial genome of Brown Wood Owl *Strix leptogrammica* (Strigiformes: Strigidae). Mitochondrial DNA 25: 370-371.

Liu, G, Zhou, L & Zhao, G 2019a. Complete mitochondrial genomes of five raptors and implications for the phylogenetic relationships between owls and nightjars. PeerJ Preprints, e27478v1.

Luo, X, Kang, X & Zhang, D 2016. Complete mitochondrial genome of the American flamingo, *Phoenicopterus ruber* (Phoenicopteriformes, Phoenicopteridae). Mitochondrial DNA Part A 27: 3519-3520.

Pellegrino, I, Cucco, M, Harvey, JA, Liberatore, F, Pavia, M, Voelker, G & Boano, G 2017. So similar and yet so different: taxonomic status of Pallid Swift *Apus pallidus* and Common Swift *Apus apus*. Bird Study 64: 344-352.

Qiu, S, Liu, H, Cai, Y-S, Hou, W, Dou, L, Zhang, X-Y & Li, J 2019. Complete mitochondrial genome and the phylogenetic position of the common cuckoo, *Cuculus canorus bakeri* (Aves: Cuculiformes). Mitochondrial DNA Part B 4: 2802-2803.

Spiridonova, LN & Surmach, SG 2018. Whole mitochondrial genome of Blakiston’s Fish Owl *Bubo* (*Ketupa*) *blakistoni* suggests its redescription in the genus *Ketupa*. Russian Journal of Genetics 54: 369-373.

Xu, P, Li, Y, Miao, L, Xie, G & Huang, Y 2016b. Complete mitochondrial genome of the *Tyto longimembris* (Strigiformes: Tytonidae). Mitochondrial DNA Part A 27: 2481-2482.

Zhao, G, Zhou, L, Li, B & Gu, C 2016a. Complete mitochondrial genome of the Grey nightjar *Caprimulgus jotaka* (Caprimulgiformes: Caprimulgidae). Mitochondrial DNA Part A 27: 1746-1747.

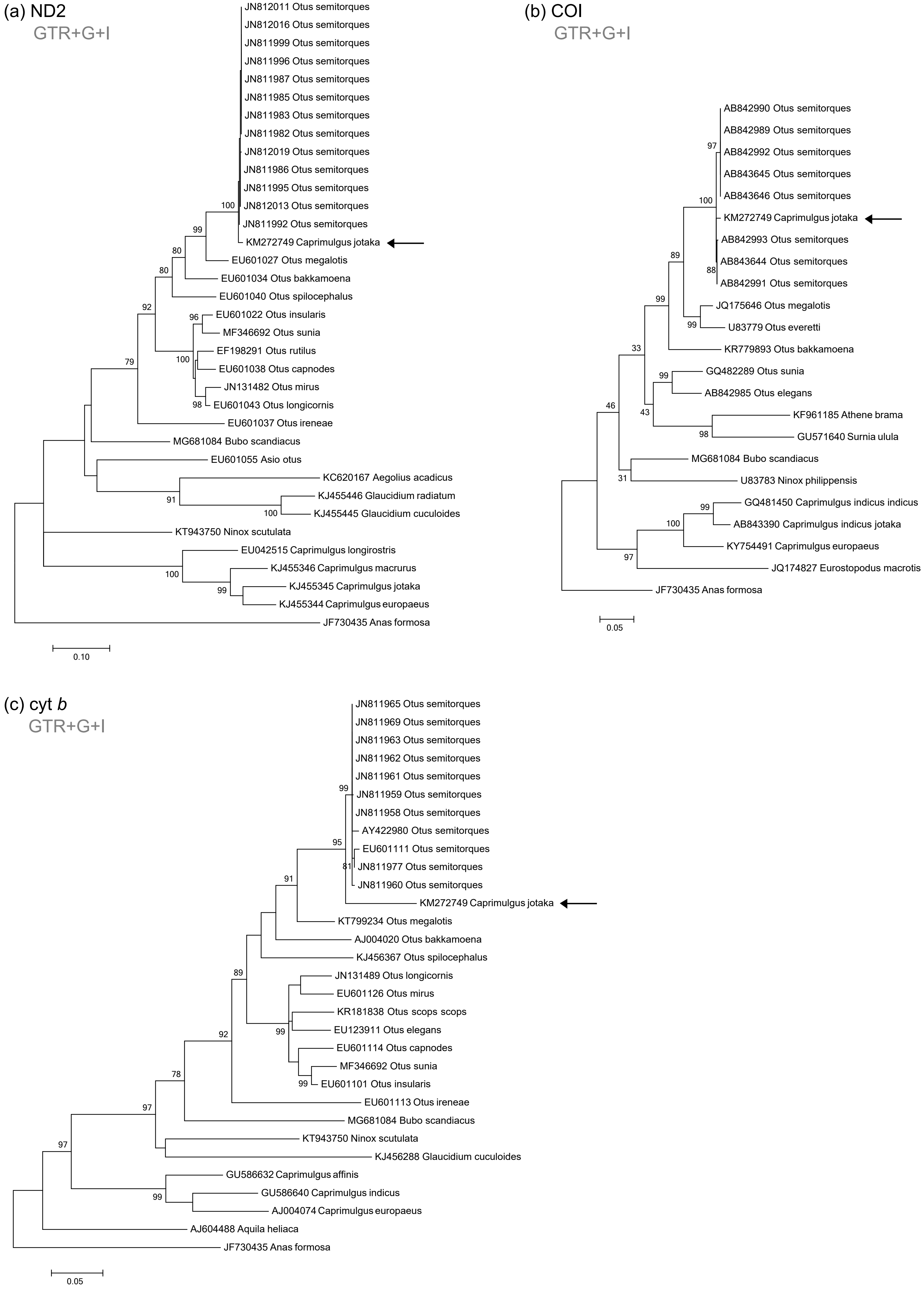

Figure S12. Maximum Likelihood phylogenies of *Caprimulgus jotaka* (KM272749) and related taxa based on mitochondrial sequences. Numbers at branches are bootstrap support values (>70%) based on 1000 replicates.

16. “*Phaethornis malaris*” KP853097, NC_030288 (Costa, I.R., Souto, H.M., Furtado, C., Mudge, J., McGuire, J., Witt, C., Jennings, B., Ruschi, P. and Prosdocimi, F., unpublished; 2015)

Fig. S13

Metadata: none on GenBank
Phylogenetic position:
ND2 = part of *Threnetes* clade, identical to *Threnetes leucurus* (AY830526), distant from other *Phaethornis*
CO1 = part of *Threnetes* clade; almost identical to *Threnetes niger* (JQ176463, JQ176464)
Cyt *b* = closer to *Glaucis* than to *Phaethornis*; no sequences available of *Threnetes* or *Phaethornis malaris*
Sequence integrity: not investigated
Interpretation: misidentification
Subsequent usage:
Re-used in **1 mitogenomic phylogeny published before 1 January 2020**: Liu et al. 2019a.
Problems noted in other works: None.
Relevance: This was the only mitogenome presumed to be of this species (January 2020)
References:
Liu, G, Zhou, L & Zhao, G 2019a. Complete mitochondrial genomes of five raptors and implications for the phylogenetic relationships between owls and nightjars. PeerJ Preprints, e27478v1.

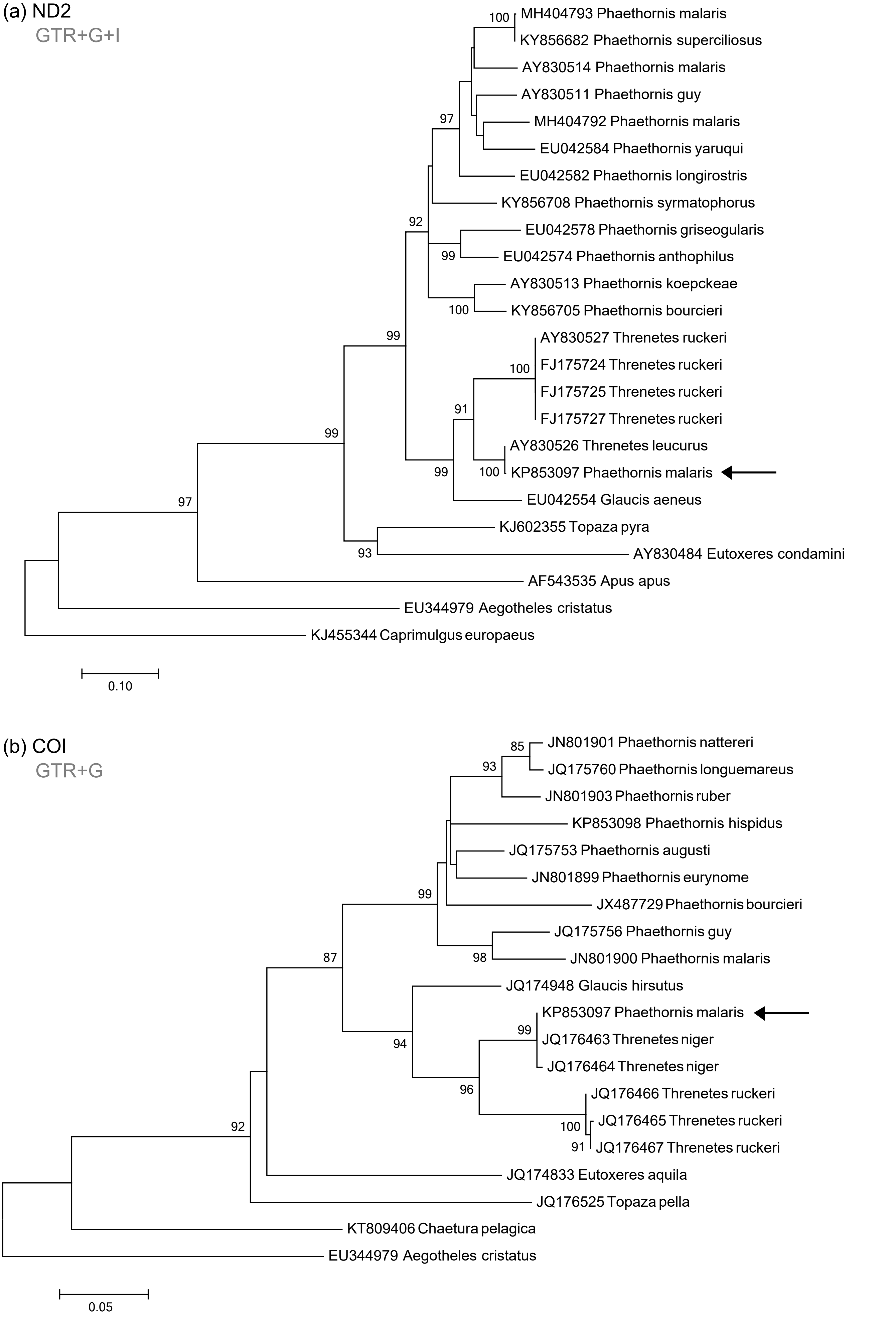

Figure S13. Maximum Likelihood phylogenies of *Phaethornis malaris* (KP853097) and related taxa based on mitochondrial sequences. Numbers at branches are bootstrap support values (>70%) based on 1000 replicates.

17. "*Cuculus canorus*" MN067867 (Qiu et al. 2019)

Fig. S14

Metadata: "The muscle sample from a natural died individual of *C. canorus bakeri* was collected from Laojunshan National Nature Reserve, Yibin, Sichuan Province, China (104º00.99', 28º41.98'). The specimen was stored in the Natural Museum of Sichuan University with a voucher number of 2019052101." (Qiu et al. 2019: 2802)

Phylogenetic position:

ND2 = among *Cuculus canorus* and one *C. saturatus* but on a long branch. Did not cluster with other *C. c. bakeri.*

CO1 = among *Cuculus canorus* and *C. saturatus* but on a long branch. Did not cluster with other *C. c. bakeri.*

Cyt *b* = could not be verified due to lack of phylogenetic structure.

Sequence integrity:

ND2: Base pairs 1 - 259 included 30 unique substitutions relative to *Cuculus* and *Hierococcyx*, and was >10% divergent from other *Cuculus* sequences). This divergent part did not closely match any other species (BLASTn).

COI: 10 unique substitutions relative to 59 *Cuculus* (*C. canorus, C. saturatus, C. micropterus, C. poliocephalus*).

Interpretation: Sequencing errors / numts

Subsequent usage: not re-used in any further studies (January 2020).

Problems noted in other works: none.

Relevance: no other mitogenomes of this species are available from GenBank (January 2020).

References:

Qiu, S, Liu, H, Cai, Y-S, Hou, W, Dou, L, Zhang, X-Y & Li, J 2019. Complete mitochondrial genome and the phylogenetic position of the common cuckoo, *Cuculus canorus bakeri* (Aves: Cuculiformes). Mitochondrial DNA Part B 4: 2802-2803.

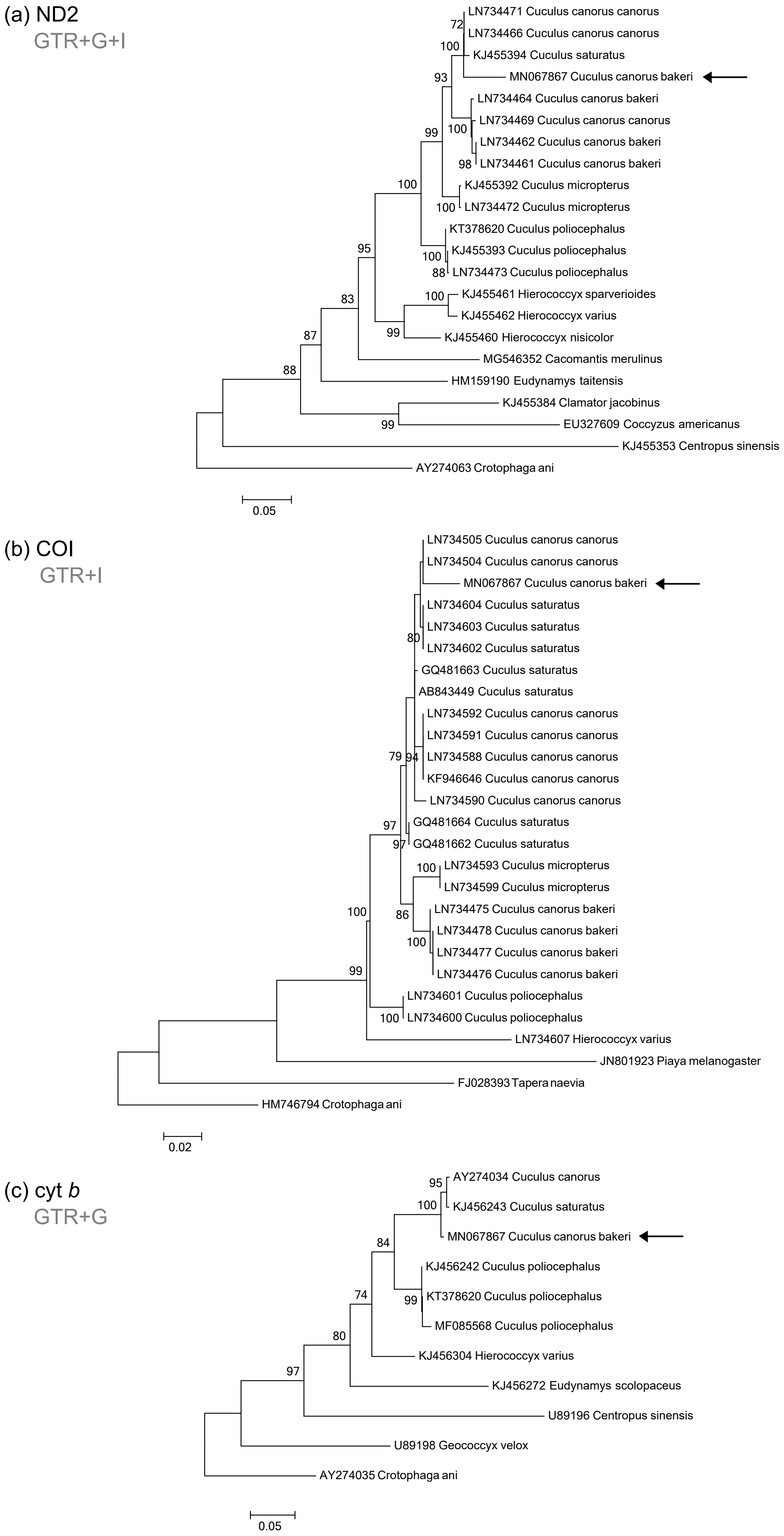

Figure S14. Maximum Likelihood phylogenies of *Cuculus canorus bakeri* (MN067867) and related taxa based on mitochondrial sequences. Numbers at branches are bootstrap support values (>70%) based on 1000 replicates.

18. “*Coturnicops exquisitus*” AP010823, NC_012143 (Ozaki et al. 2010)

Fig. S15

Metadata: No information in Ozaki et al. (2010). GenBank: isolate="CNE01" and tissue_type="muscle"
Phylogenetic position:
ND2 = could not be verified due to lack of reference sequences of *C. exquisitus*.
CO1 = could not be verified due to lack of reference sequences of *C. exquisitus*.
Cyt *b* = sister to *C. exquisitus* (n=4) and *C. noveboracensis* (n=1).
Sequence integrity: cyt *b* (bp 1-1018): highly divergent from four sequences of *C. exquisitus* (10.7-10.9% uncorrected p-value, with complete deletion of missing sites). This fragment did not match any other bird species (BLAST).
Interpretation: Sequencing errors / numts
Subsequent usage:
Re-used in **7 mitogenomic phylogenies published before 1 January 2020**: García-R et al. 2014, Nabholz et al. 2016, Chen et al. 2017, Du Plessis et al. 2017, Gong et al. 2017, Stervander et al. 2018, Boast et al. 2019.
Re-used in **1 non-mitogenomic phylogeny**: Ruan et al. 2012.
Used in **1 further paper**: He et al. 2016 (*Fulica atra*).
Taxonomic conclusion about species status of Yellow Rail based on sequence divergence (Ozaki et al. 2010: 61).
Problems noted in other works: none.
Relevance: This was the only mitogenome presumed to be of this species (January 2020).
References:

Boast, AP, Chapman, B, Herrera, MB, Worthy, TH, Scofield, RP, Tennyson, AJ, Houde, P, Bunce, M, Cooper, A & Mitchell, KJ 2019. Mitochondrial genomes from New Zealand’s extinct adzebills (Aves: Aptornithidae: *Aptornis*) support a sister-taxon relationship with the Afro-Madagascan Sarothruridae. Diversity 11(2), 24.
Chen, P, Han, Y, Zhu, C, Gao, B & Ruan, L 2017. Complete mitochondrial genome of *Porzana fusca* and *Porzana pusilla* and phylogenetic relationship of 16 Rallidae species. Genetica 145: 559–573.
Du Plessis, M, Dalton, DL, Smit-Robinson, HA & Kotze, A 2017. Next generation sequencing yields the mitochondrial genome of the critically endangered *Sarothrura ayresi* (white-winged flufftail). Mitochondrial DNA Part B 2: 236-237.
García-R, JC, Gibb, GC & Trewick, SA 2014. Eocene diversification of crown group rails (Aves: Gruiformes: Rallidae). PLoS ONE 9(10): e109635.
Gong, J, Zhao, R, Huang, Q, Sun, X, Huang, L & Jing, M 2017. Two mitogenomes in Gruiformes (*Amaurornis akool/A. phoenicurus*) and the phylogenetic placement of Rallidae. Genes & Genomics 39: 987–995.
He, K, Ren, T, Zhu, S & Zhao, A 2016a. The complete mitochondrial genome of *Fulica atra* (Avian [sic], Gruiformes, Rallidae). Mitochondrial DNA A 27: 3161-3162.
Nabholz, B, Lanfear, R & Fuchs, J 2016. Body mass-corrected molecular rate for bird mitochondrial DNA. Mol. Ecol. 25: 4438-4449.
Ozaki, K, Yamamoto, Y & Yamagishi, S 2010. Genetic diversity and phylogeny of the endangered Okinawa Rail, *Gallirallus okinawae.* Genes Genet. Syst. 85: 55-63.
Ruan, L, Wang, Y, Hu, J & Ouyang, Y 2012. Polyphyletic origin of the genus *Amaurornis* inferred from molecular phylogenetic analysis of rails. Biochemical Genetics 50: 959-966.
Stervander, M, Ryan, PG, Melo, M & Hansson, B 2019. The origin of the world’s smallest flightless bird, the Inaccessible Island Rail *Atlantisia rogersi* (Aves: Rallidae). Molecular Phylogenetics and Evolution 130: 92-98.

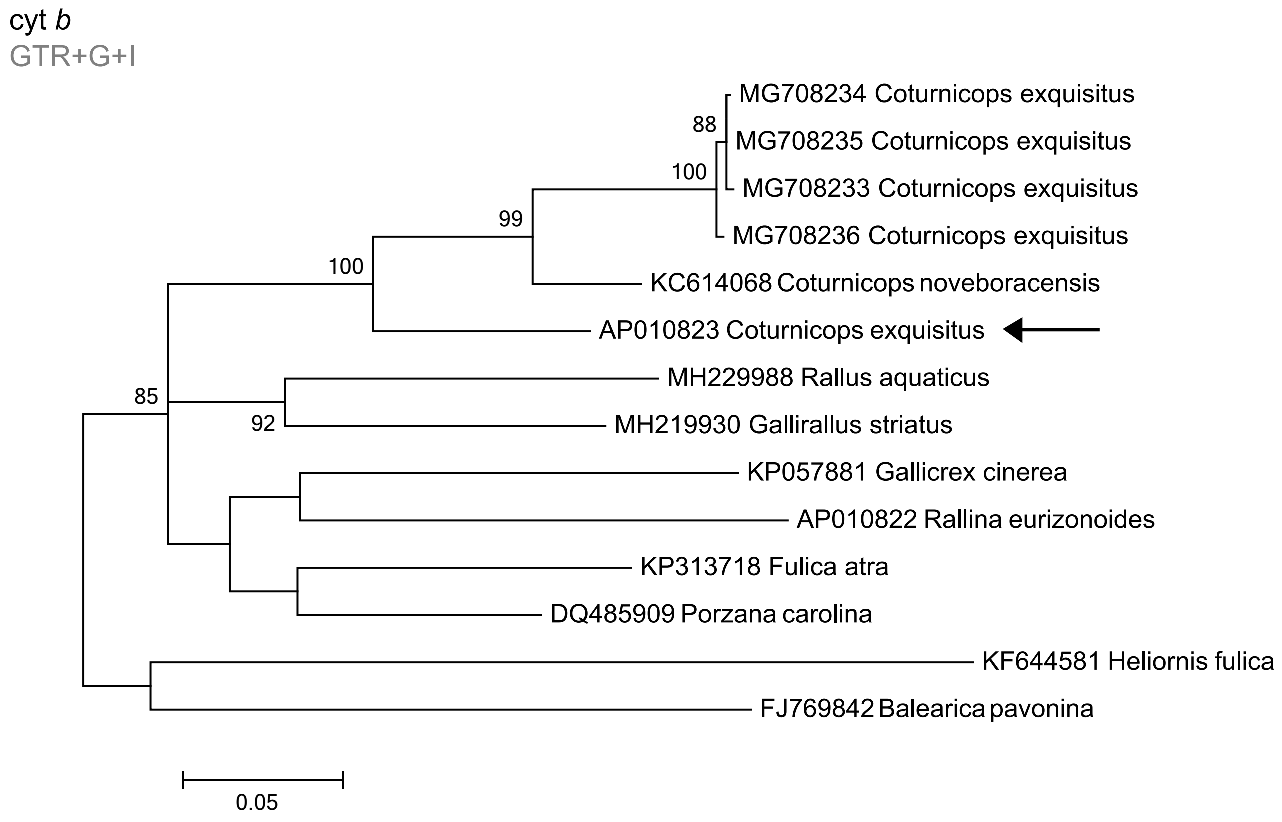

Figure S15. Maximum Likelihood phylogenies of *Coturnicops exquisitus* (AP010823) and related taxa based on mitochondrial sequences. Numbers at branches are bootstrap support values (>70%) based on 1000 replicates.

19. "*Amaurornis akool*" KJ192198, NC_023982 (Gong et al. 2017)

Fig. S16

Metadata: "The specimens of *A. akool* and *A. phoenicurus* were collected from Wuyi Mountain, Fujian Province, China. The specimen was identified based on external characteristics, using the classification criterion of Sibley and Monroe (1990)." (Gong et al. 2017: 988)

Phylogenetic position:

ND2 = *Gallinula chloropus*

CO1 = sister to *Gallinula chloropus* and *G. galeata*

Cyt *b* = sister to *Amaurornis phoenicurus*

Sequence integrity: COI bp 1 – circa 421 = *Gallinula chloropus* (identical); bp circa 433 – circa 694 = *Amaurornis phoenicurus* (identical).

Interpretation: chimera

Subsequent usage:

Re-used in **2 mitogenomic phylogenies published before 1 January 2020**: Chen et al. 2017, Boast et al. 2019.

Problems noted in other works: none. Boast et al. (2019) used Gong et al.'s (2017) sequence of *Amaurornis akool* (now *Zapornia akool*) and found a close relationship with *Amaurornis* (bush-hens and allies), and *Gallicrex cinerea* (Watercock). This contradicted previous results based on Ruan et al.'s (2012) data, which suggested *Z. akool* was nested within *Zapornia*. Boast et al. (2019) erroneously concluded that the *Z. akool* sequences of Ruan et al. (2012) were likely to be spurious and recommended reinstating the original classification of *Z. akool* as *Amaurornis akool*. Boast et al. (2019), however, were unaware that Gong et al.'s (2017) sequence of *Amaurornis akool* was chimeric and that Ruan's sequences of *akool* (COI) and *phoenicurus* (cyt b) match those of independent sequences of these species.

Relevance: This was the only mitogenome presumed to be of this species (January 2020).

References:

Boast, AP, Chapman, B, Herrera, MB, Worthy, TH, Scofield, RP, Tennyson, AJ, Houde, P, Bunce, M, Cooper, A & Mitchell, KJ 2019. Mitochondrial genomes from New Zealand's extinct adzebills (Aves: Aptornithidae: *Aptornis*) support a sister-taxon relationship with the Afro-Madagascan Sarothruridae. Diversity 11(2), 24.

Chen, P, Han, Y, Zhu, C, Gao, B & Ruan, L 2017. Complete mitochondrial genome of *Porzana fusca* and *Porzana pusilla* and phylogenetic relationship of 16 Rallidae species. Genetica 145: 559–573.

Gong, J, Zhao, R, Huang, Q, Sun, X, Huang, L & Jing, M 2017. Two mitogenomes in Gruiformes (*Amaurornis akool/A. phoenicurus*) and the phylogenetic placement of Rallidae. Genes & Genomics 39: 987–995.

Ruan, L, Wang, Y, Hu, J & Ouyang, Y 2012. Polyphyletic origin of the genus *Amaurornis* inferred from molecular phylogenetic analysis of rails. Biochemical Genetics 50: 959-966.

20. "*Amaurornis phoenicurus*" KJ874440, NC_024593 (Gong et al. 2017)

Fig. S16

Metadata: "The specimens of *A. akool* and *A. phoenicurus* were collected from Wuyi Mountain, Fujian Province, China. The specimen was identified based on external characteristics, using the classification criterion of Sibley and Monroe (1990)." (Gong et al. 2017: 988)

Phylogenetic position:

ND2 = could not be verified (no sequence of *Amaurornis phoenicurus* available).

CO1 = *Amaurornis phoenicurus*

Cyt *b* = sister to *Gallinula chloropus, G. galeata* and *G. tenebrosa*

Sequence integrity: cyt *b* bp 92 – circa 513 = *Amaurornis phoenicurus*; bp circa 537 – circa 1020 = *Gallinula chloropus*

Interpretation: chimera

Subsequent usage:

Re-used in **4 mitogenomic phylogenies published before 1 January 2020**: Nabholz et al. 2016, Chen et al. 2017, Stervander et al. 2018, Boast et al. 2019

Problems noted in other works: none.

Relevance: This was the only mitogenome presumed to be of this species (January 2020).

References:

Boast, AP, Chapman, B, Herrera, MB, Worthy, TH, Scofield, RP, Tennyson, AJ, Houde, P, Bunce, M, Cooper, A & Mitchell, KJ 2019. Mitochondrial genomes from New Zealand's extinct adzebills (Aves: Aptornithidae: *Aptornis*) support a sister-taxon relationship with the Afro-Madagascan Sarothruridae. Diversity 11(2), 24.

Chen, P, Han, Y, Zhu, C, Gao, B & Ruan, L 2017. Complete mitochondrial genome of *Porzana fusca* and *Porzana pusilla* and phylogenetic relationship of 16 Rallidae species. Genetica 145: 559–573.

Gong, J, Zhao, R, Huang, Q, Sun, X, Huang, L & Jing, M 2017. Two mitogenomes in Gruiformes (*Amaurornis akool/A. phoenicurus*) and the phylogenetic placement of Rallidae. Genes & Genomics 39: 987–995.

Nabholz, B, Lanfear, R & Fuchs, J 2016. Body mass-corrected molecular rate for bird mitochondrial DNA. Mol. Ecol. 25: 4438-4449.

Stervander, M, Ryan, PG, Melo, M & Hansson, B 2019. The origin of the world's smallest flightless bird, the Inaccessible Island Rail *Atlantisia rogersi* (Aves: Rallidae). Molecular Phylogenetics and Evolution 130: 92-98.

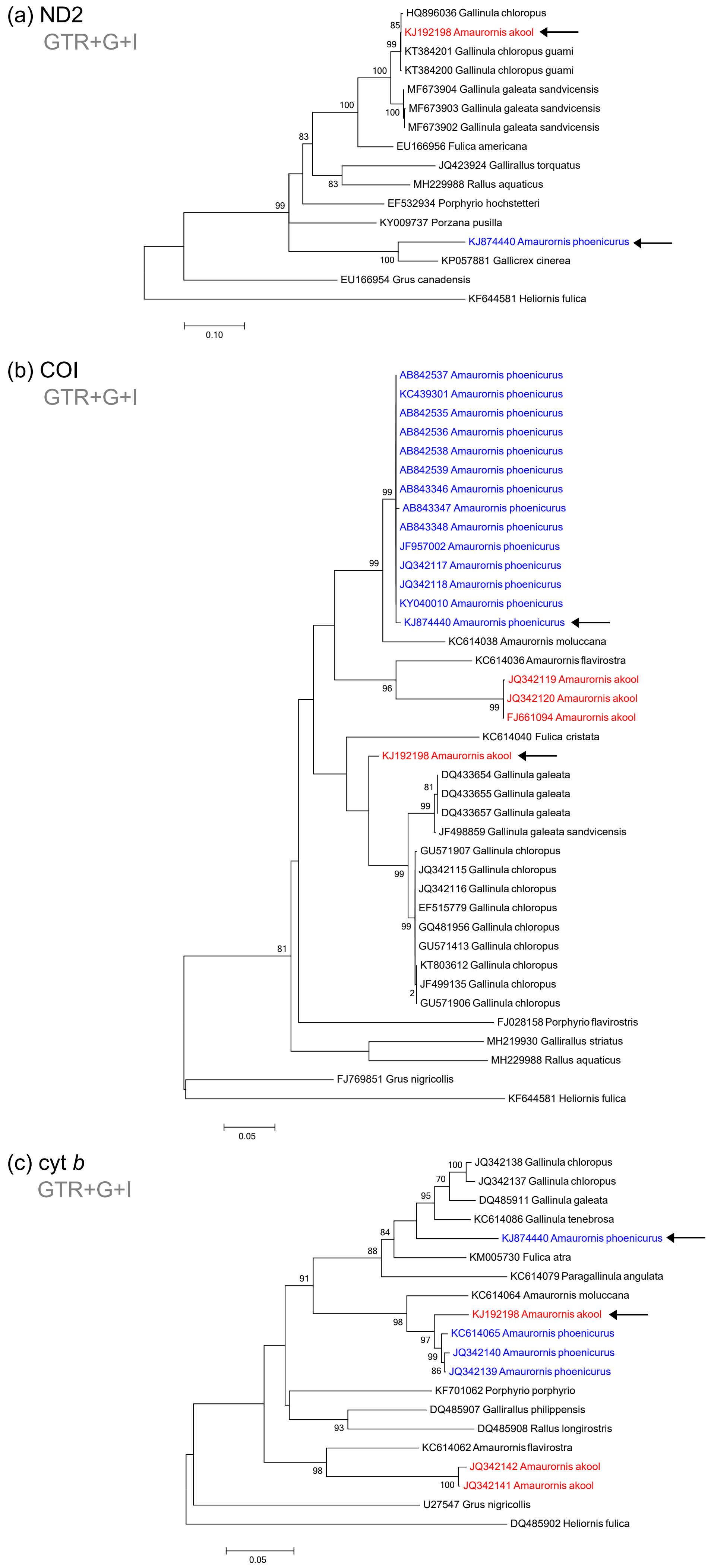

Figure S16. Maximum Likelihood phylogenies of *Amaurornis akool* (KJ192198), *A. phoenicurus* (KJ874440) and related taxa based on mitochondrial sequences. Numbers at branches are bootstrap support values (>70%) based on 1000 replicates.

21. “*Rallus aquaticus*” MH229988, NC_041578 (Chen et al. 2020)

Fig. S17

Metadata: A blood sample of *Rallus aquaticus* was taken in Lianyungang, Jiangsu province, China.

Phylogenetic position:

ND2 = could not be verified due to lack of reference sequences of *Rallus indicus*

CO1 = *Rallus indicus*

Cyt *b* = could not be verified due to lack of reference sequences of *Rallus indicus*

Sequence integrity: not verified

Interpretation: misidentification due to using outdated taxonomy. *R. indicus* was formerly included in *R. aquaticus* but was separated based on molecular and bioacoustic differences (Sangster et al. 2011).

Subsequent usage: No citations or subsequent uses (January 2020).

Problems noted in other works: none.

Relevance: This was the only mitogenome presumed to be of this species (January 2020).

References:

Chen, P, Huang, Z, Zhu, C, Han, Y, Xu, Z, Sun, G, Zhang, Z, Zhao, D, Ge, G & Ruan, L 2020. Complete mitochondrial genome and phylogenetic Analysis of Gruiformes and Charadriiformes. Pakistan Journal of Zoology 52: 425-439.

Sangster, G, Collinson, M, Crochet, P-A, Knox, AG, Parkin, DT, Svensson, L & Votier, SC 2011. Taxonomic recommendations for British birds: seventh report. Ibis 153: 883-892.

COI

GTR+G+I

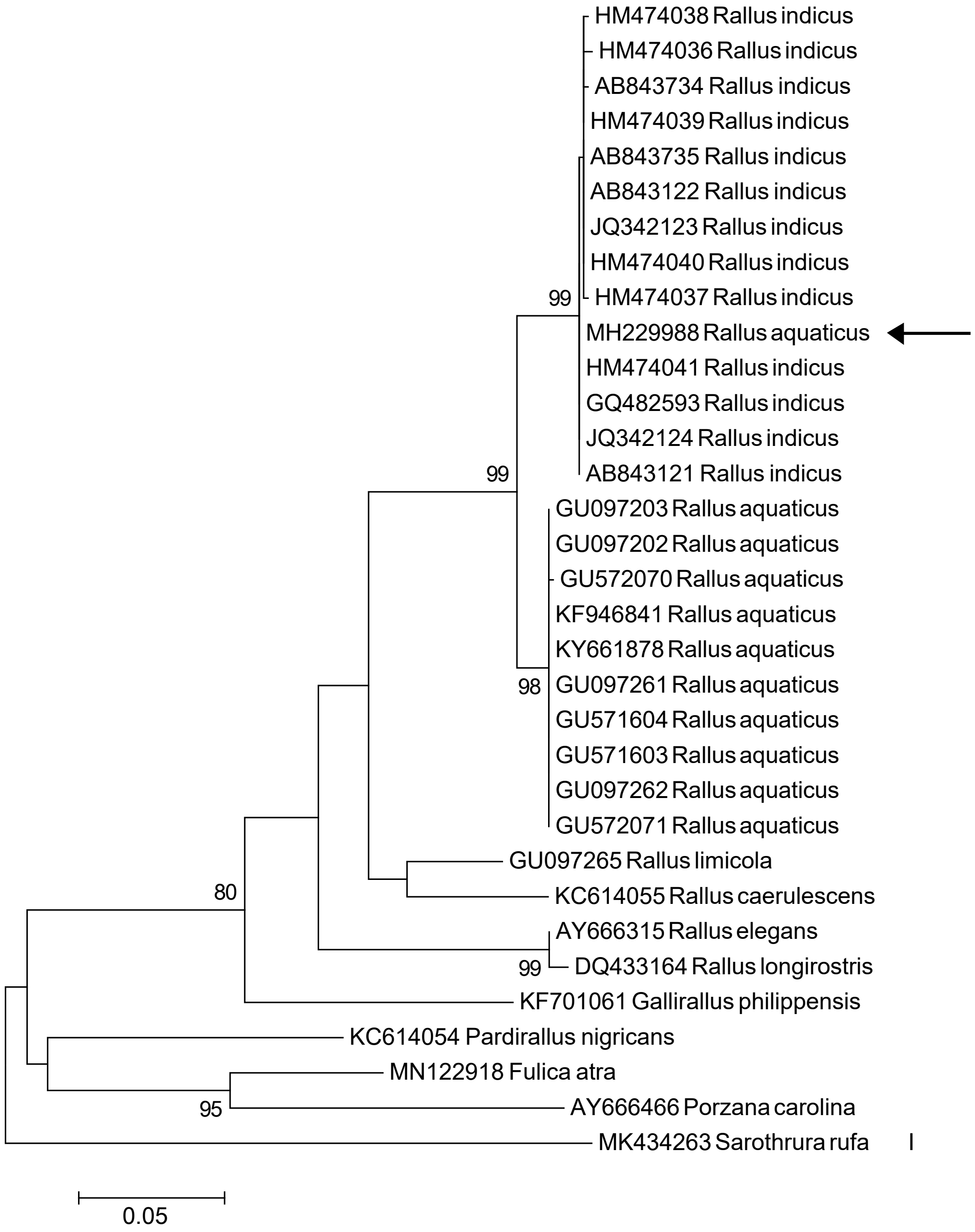

Figure S17. Maximum Likelihood phylogenies of *Rallus aquaticus* (MH229988) and related taxa based on mitochondrial sequences. Numbers at branches are bootstrap support values (>70%) based on 1000 replicates.

22. “*Grus leucogeranus*” MH041490 (Wang et al. 2018)

Fig. S18

Metadata: “Peripheral blood of Siberian Crane (♀) were [sic] collected from Bei Fang Seng Lin Zoo in A’cheng district, Harbin, Heilongjiang Province (126.4200°N, 45.1200°E), China.” (Wang et al. 2018: 575).

Phylogenetic position:

ND2 = sister to *Grus vipio* (complete mitogenome FJ769852) rather than to *G. leucogeranus* (complete mitogenome FJ769846)

CO1 = *Grus vipio*

Cyt *b* = *Grus vipio*

Sequence integrity: not investigated

Interpretation: misidentified.

Subsequent usage: No citations or subsequent uses (January 2020).

Problems noted in other works: none.

Relevance: A mitogenome of *G. leucogeranus* was previously published by Krajewski et al. (2010).

References:

Krajewski, C, Sipiorski, JT & Anderson, FE 2010. Complete mitochondrial genome sequences and the phylogeny of cranes (Gruiformes: Gruidae). Auk 127: 440-452.

Wang, T, Wang, H, Zhao, Z, Wang, Z, Mu, L & Yu, H 2018. Complete mitochondrial genome of a Siberian Crane (*Grus leucogeranus*). Mitochondrial DNA Part B 3: 575-576.

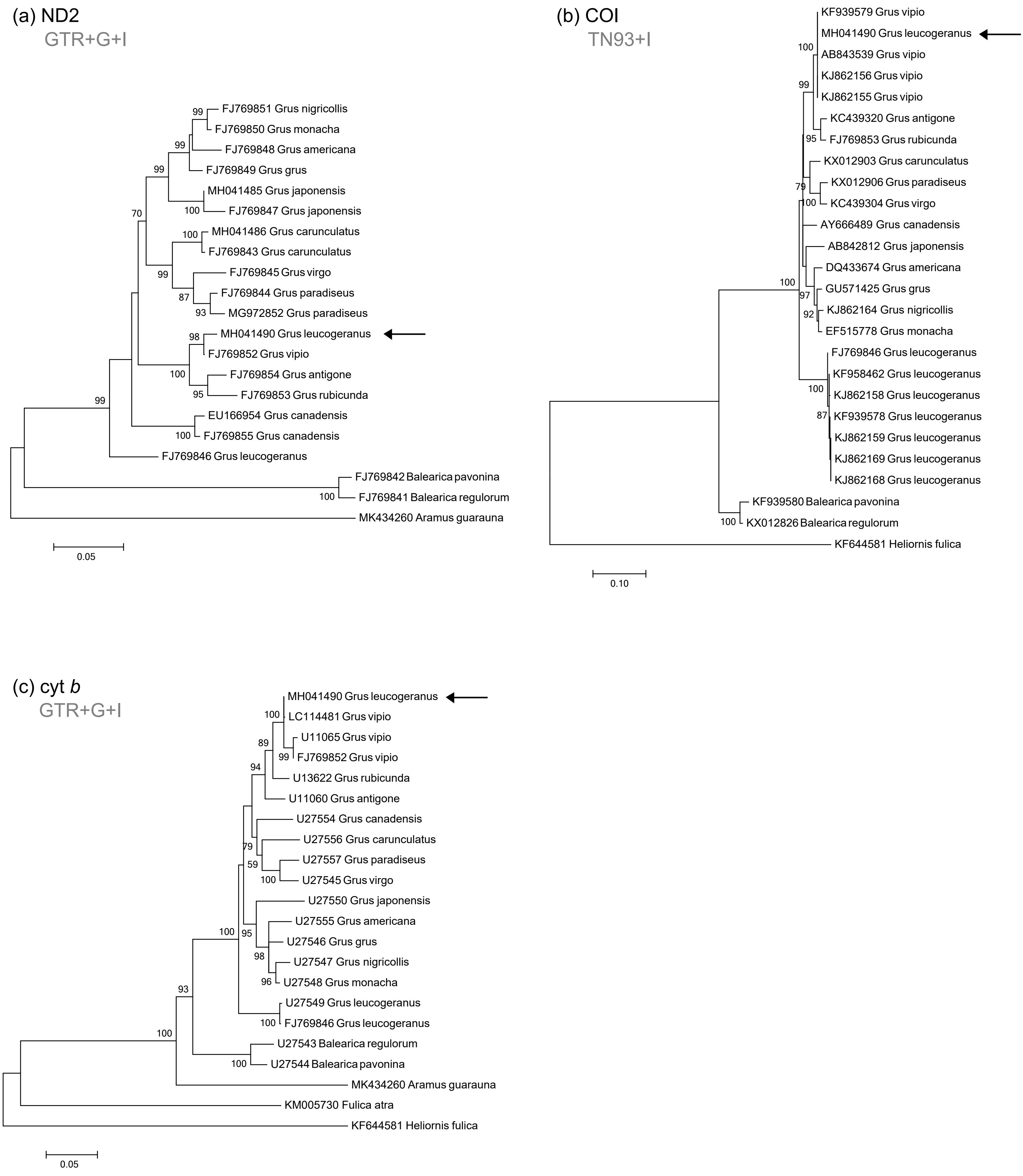

Figure S18. Maximum Likelihood phylogenies of *Grus leucogeranus* (MH041490) and related taxa based on mitochondrial sequences. Numbers at branches are bootstrap support values (>70%) based on 1000 replicates.

23. "*Vanellus cinereus*" KM873665 (She et al. 2016)

Fig. S19

Metadata: "Tissues were collected from an accidental death of individual on 12 September 2013, found in Xinqiao International Airport, Anhui Province, China. It was kept in the Institute of Biodiversity and Wetland Ecology, Anhui University." (She et al. 2016: 3120)

Phylogenetic position:

ND2 = identical to another mitogenome of *Vanellus cinereus* (KM404175)

CO1 = identical to another mitogenome of *Vanellus cinereus* (KM404175)

Cyt *b* = sister to another mitogenome of *Vanellus cinereus* (KM404175) but on a long branch

Sequence integrity: cyt b: Bp 1-153 contain many singleton sites compared with *V. cinereus* (n=1), *V. vanellus* (n=1-2), *V. chilensis* (n=0-1). This fragment is a 100% match (i.e. identical) to a mitogenome of *Motacilla alba* (KT736087).

Interpretation: chimera

Subsequent usage:

Re-used in **7 mitogenomic phylogenies published before 1 January 2020**: Eo & An 2016a, Nabholz et al. 2016, Lee et al. 2017d, Hu et al. 2017, Lee et al. 2017c, Yang et al. 2017b, Chen et al. 2018b.

Cited by **2 other papers**: Gaikwad et al. 2016, Chen et al. 2016.

Problems noted in other works: none.

Relevance: Another mitogenome of *Vanellus cinereus* (KM404175, NC_025514) was published by Xie et al. (2016b).

References:

Chen, W, Hu, C & Chang, Q 2016. The complete mitochondrial genome of *Tringa ochropus* (Charadriiformes, Scolopacidae). Mitochondrial DNA Part B 1: 841-842.

Chen, W, Zhang C, Pan T, Liu W, Li K, Hu C, Chang Q 2018b. The mitochondrial genome of the Kentish Plover *Charadrius alexandrinus* (Charadriiformes: Charadriidae) and phylogenetic analysis of Charadrii. Genes Genomics 40: 955-963.

Eo, SH & An, J 2016a. The complete mitochondrial genome sequence of Japanese murrelet (Aves: Alcidae) and its phylogenetic position in Charadriiformes. Mitochondrial DNA Part A 27: 4574-4575.

Gaikwad, SS, Munot, H & Shouche, YS 2016. Utility of DNA barcoding for identification of bird-strike samples from India. Current Science 110: 25-28.

Hu, C, Zhang C, Sun L, Zhang Y, Xie W, Zhang B, Chang Q 2017. The mitochondrial genome of pin-tailed snipe *Gallinago stenura*, and its implications for the phylogeny of Charadriiformes. PLoS One 12:e0175244.

Lee, MY, Jeon, HS, Choi, YS, Joo, SB & An, JH. 2017c. Complete mitochondrial genome of *Haematopus ostralegus* (Charadriiformes: Haematopodidae). Mitochondrial DNA B. 2:124–125.

Lee, MY, Jeon, HS, Lee, SH & An, J 2017d. The mitochondrial genome of the long-billed plover, *Charadrius placidus* (Charadriiformes: Charadriidae). Mitochondrial DNA Part B 2: 122-123.

Nabholz, B, Lanfear, R & Fuchs, J 2016. Body mass-corrected molecular rate for bird mitochondrial DNA. Mol. Ecol. 25: 4438-4449.

She, H, Zhao, G, Zhou, L & Gu, C 2016. Complete mitochondrial genome of Grey-headed Lapwing *Vanellus cinereus* (Ciconiiformes: Charadriidae). Mitochondrial DNA Part A 27: 3120-3121.

Xie, W, Hu, C, Yu, T, Yang, R & Chang, Q 2016b. The complete mitochondrial genome of *Vanellus cinereus* (Charadriiformes: Charadriidae). Mitochondrial DNA Part A 27: 1726-1727.

Yang, C, Wang, QX, Li, XJ, Xiao, H & Huang, Y 2017b. Characterization of the mitogenomes for two sympatric breeding species in Recurvirostridae (Charadriiformes) and their phylogenetic implications. Mitochondrial DNA Part B 2: 182-184.

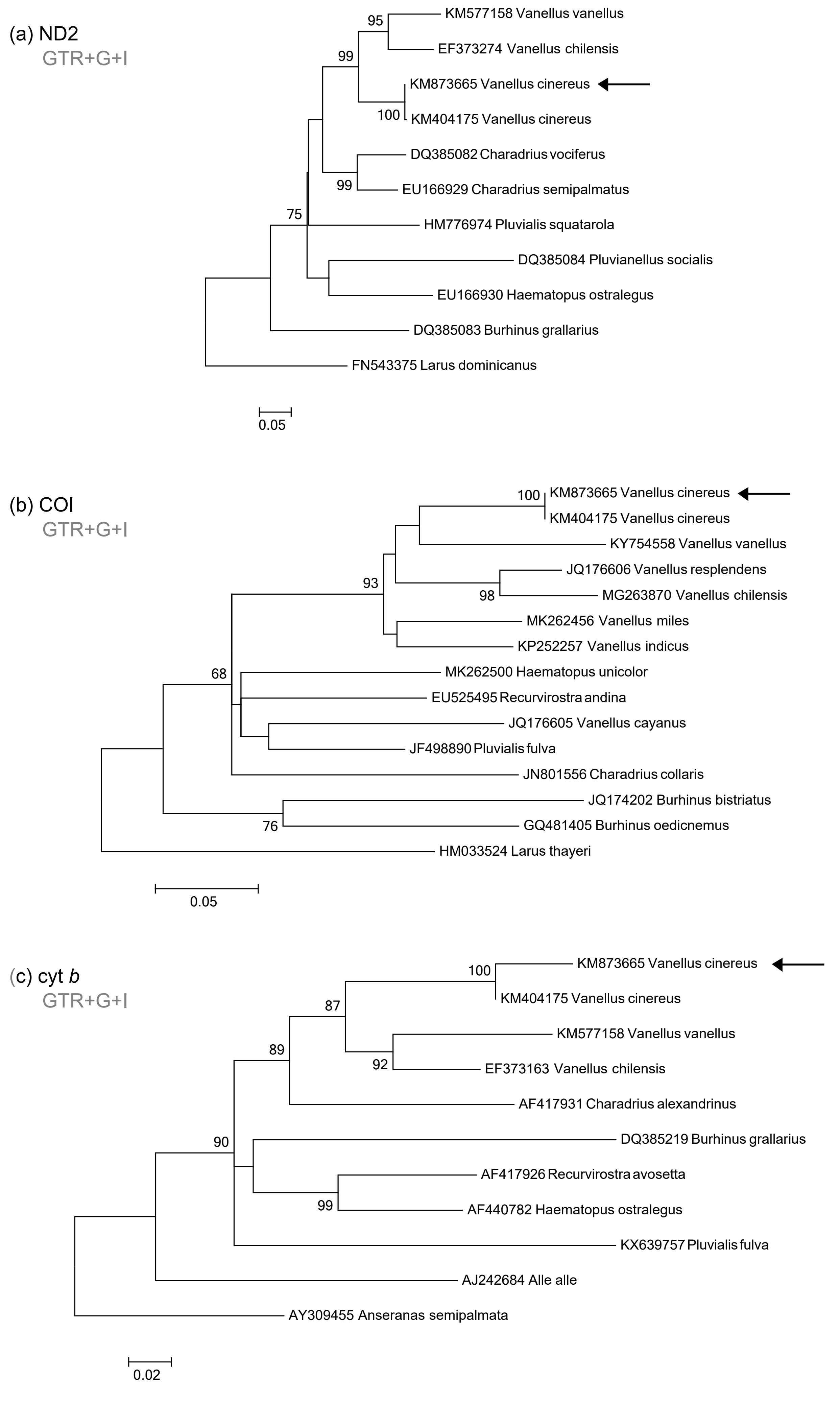

Figure S19. Maximum Likelihood phylogenies of *Vanellus cinereus* (KM873665) and related taxa based on mitochondrial sequences. Numbers at branches are bootstrap support values (>70%) based on 1000 replicates.

24. "*Charadrius placidus*" KY419888 (Lee et al. 2017d)

Fig. S20

Metadata: "A specimen of *C. placidus* (IN671) was collected from the Taehwa River, Wolsan-si, Kyeongsangnam-do, South Korea, and deposited in the National Institute of Biological Resources (NIBR) at Incheon, South Korea." (Lee et al. 2017d: 122-123)

Phylogenetic position:

ND2 = very similar to *C. alexandrinus*, but no sequence of *C. placidus* available

CO1 = *C. alexandrinus*, distant from *C. placidus*

Cyt *b* = very similar to *C. alexandrinus*, but no sequence of *C. placidus* available

Sequence integrity: not investigated

Interpretation: misidentified. Verifiable with specimen.

Subsequent usage:

Re-used in **5 mitogenomic phylogenies or networks published before 1 January 2020**: Lee et al. 2017b, Lee et al. 2017c, Bi 2017, Yang et al. 2017a, Chen et al. 2018b (**very similar to *C. alexandrinus* but no comment**).

Problems noted in other works: none.

Relevance: This was the only mitogenome presumed to be of *Charadrius placidus* (January 2020).

References:

Bi, G 2017. The complete mitochondrial genome of *Tringa semipalmata inornata* (Charadriiformes; Scolopacidae). Mitochondrial DNA Part B 2: 349-351.

Chen, W, Zhang C, Pan T, Liu W, Li K, Hu C, Chang Q 2018b. The mitochondrial genome of the Kentish Plover *Charadrius alexandrinus* (Charadriiformes: Charadriidae) and phylogenetic analysis of Charadrii. Genes Genomics 40: 955-963.

Lee, MY, Jeon, HS, Choi, YS, Joo, SB & An, JH. 2017c. Complete mitochondrial genome of *Haematopus ostralegus* (Charadriiformes: Haematopodidae). Mitochondrial DNA B. 2:124–125.

Lee, MY, Jeon, HS, Kim, YJ & An, J 2017b. Complete mitochondrial genome of *Ciconia nigra* (Ciconiiformes: Ciconiidae). Mitochondrial DNA Part B 2: 230-231.

Lee, MY, Jeon, HS, Lee, SH & An, J 2017d. The mitochondrial genome of the long-billed plover, *Charadrius placidus* (Charadriiformes: Charadriidae). Mitochondrial DNA Part B 2: 122-123.

Yang, C, Wang, QX, Li, XJ, Yuan, H, Xiao, H & Huang, Y 2017a. The mitogenomes of *Gelochelidon nilotica* and *Sterna hirundo* (Charadriiformes, Sternidae) and their phylogenetic implications. Mitochondrial DNA Part B 2: 601-603.

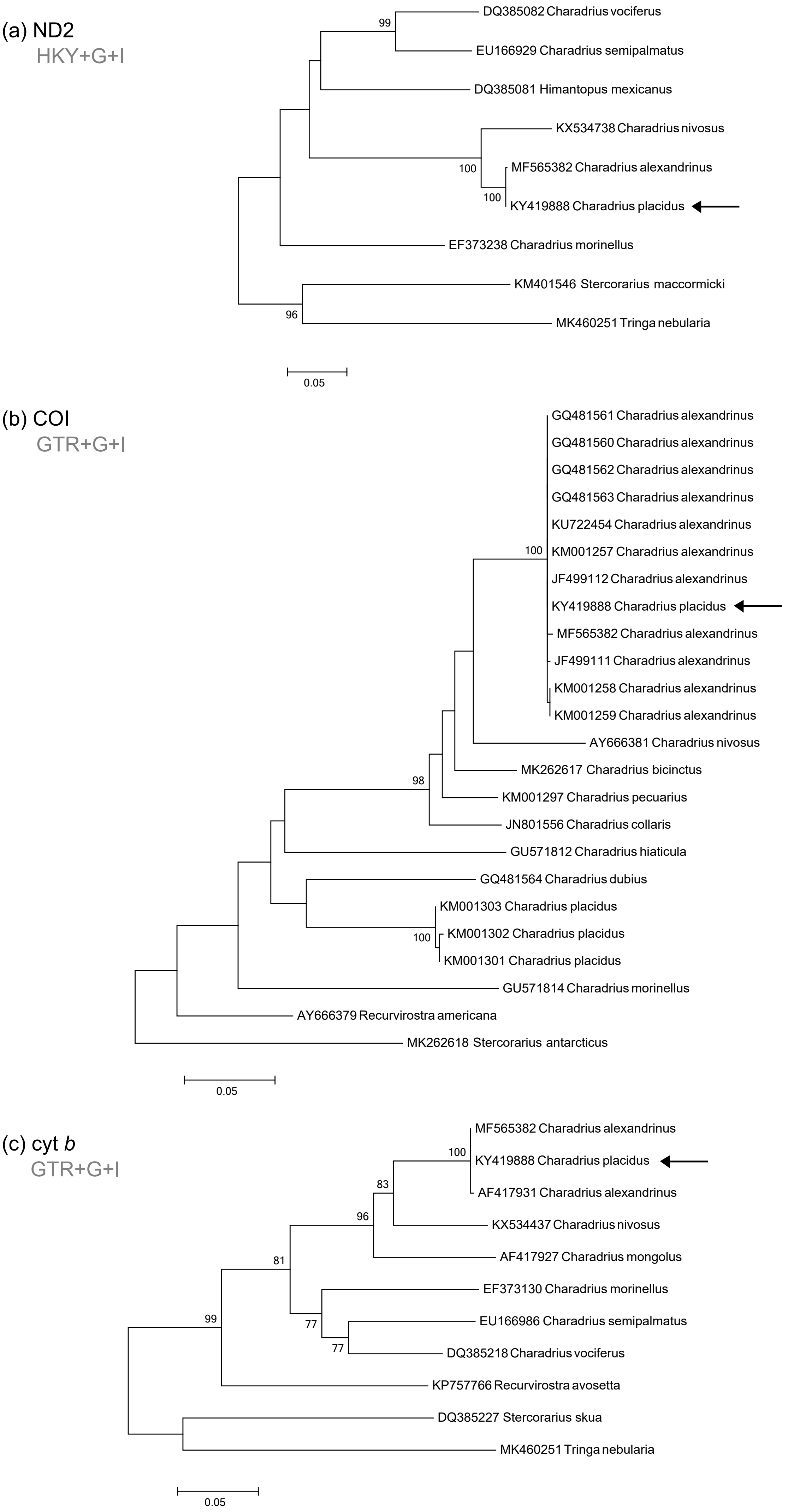

Figure S20. Maximum Likelihood phylogenies of *Charadrius placidus* (KY419888) and related taxa based on mitochondrial sequences. Numbers at branches are bootstrap support values (>70%) based on 1000 replicates.

25. "*Tringa totanus*" MK922124, NC_044648 (Ren et al. 2019b)

Fig. S21

Metadata: In this study, we determined the complete mitochondrial genome of T. totanus from an individual collected at Aba Hongyuan Airport, Sichuan Province, China. The sample was kept in the Key Laboratory of Bioresources and Ecoenvironment, Sichuan University." (Ren et al. 2019b: 2345)

Phylogenetic position:

ND2 = *Tringa totanus*

CO1 = *Tringa totanus*

Cyt *b* = *Tringa totanus* but on a long branch

Sequence integrity: cyt b: bp 1-133 contain 17 singleton sites (compared with all other available *Tringa* sequences for this fragment*: T. totanus* (n=1), *T. glareola* (n=2), *T. nebularia* (n=1), *T. guttifer* (n=1), *T. ochropus* (n=1), *T. erythropus* (n=1), *T. semipalmata* (n=1), which were identical at these sites); this 133 bp fragment is >12% divergent from the other *T. totanus* but did not closely match any other species (BLAST).

Interpretation: sequencing errors / numts

Subsequent usage: no subsequent uses yet (January 2020)

Problems noted in other works: none.

Relevance: This was the only mitogenome presumed to be of this species (January 2020).

References:

Ren, J, Jiang, X, Dou, L, Yan, L, Zhang, X & Zhang, X 2019*b. The complete mitochondrial genome of common redshank (*Tringa totanus*). Mitochondrial DNA Part B 4: 2345-2346.

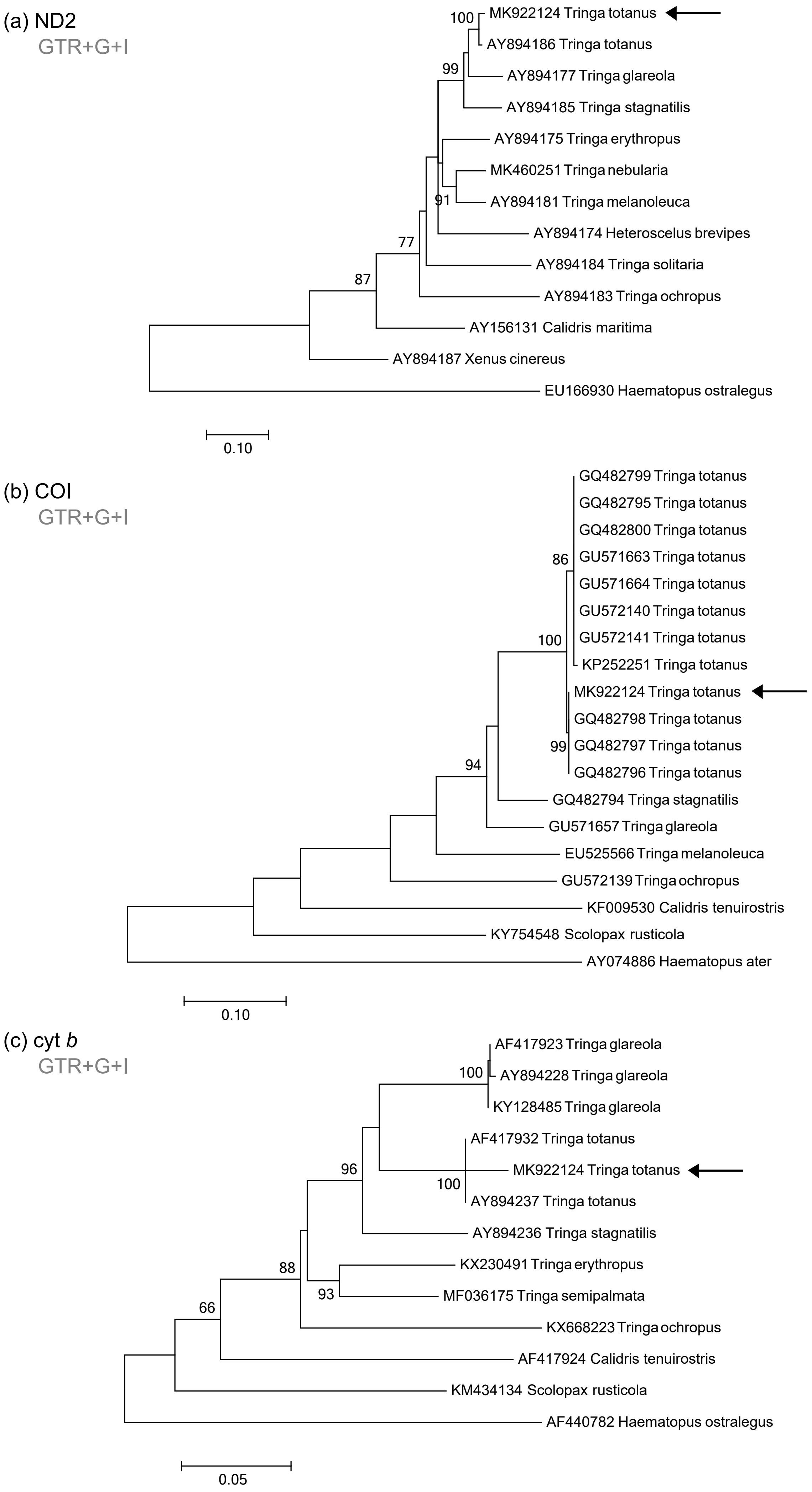

Figure S21. Maximum Likelihood phylogenies of *Tringa totanus* (MK922124) and related taxa based on mitochondrial sequences. Numbers at branches are bootstrap support values (>70%) based on 1000 replicates.

26. “*Larus vegae*” KT943749 (Park, C.E., Hong, S.J., Park, G.S., Kim, M.C., Park, H.C. and Shin, J.H., unpublished; 2015)

Fig. S22

Metadata: no data on GenBank.
Phylogenetic position:
ND2 = near *Calonectris borealis/diomedea*; no sequence available of *C. leucomelas*
CO1 = *Calonectris leucomelas*
Cyt *b* = *Calonectris leucomelas*
Sequence integrity: not investigated
Interpretation: misidentification
Subsequent usage:
Re-used in **5 mitogenomic phylogenies or networks published before 1 January 2020**: Bi 2017, Yang et al. 2017a, Yang et al. 2017b, Jiang et al. 2018, Jiang 2019.
Cited by **1 other paper**: Yoon et al. 2017 (Metabarcoding Antarctic Toothfish; suppl data 2),
Problems noted in other works: None. Yang et al. (2017a) commented that: “the status of *Larus vegae* should be further investigated” and that “Extraordinarily, the *Larus vegae* was more primitive and located in the root of the tree, but not belonged to the branch of Laridae”. The exact same comments were made in (Yang et al. 2017b).
Relevance: This was the only mitogenome presumed to be of this species (January 2020).
References:

Bi, G 2017. The complete mitochondrial genome of *Tringa semipalmata inornata* (Charadriiformes; Scolopacidae). Mitochondrial DNA Part B 2: 349-351.

Jiang, JQ 2019. Characterization of the complete mitochondrial genome of *Chlamydotis macqueenii*. Mitochondrial DNA Part B 4: 1873-1874.

Jiang, JQ, Wang, QH & Gao, TZ 2018. Characterization of the complete mitochondrial genome of band-rumped Storm-petrel, *Hydrobates castro*. Mitochondrial DNA Part B 3: 1193-1194.

Yang, C, Wang, QX, Li, XJ, Yuan, H, Xiao, H & Huang, Y 2017a. The mitogenomes of *Gelochelidon nilotica* and *Sterna hirundo* (Charadriiformes, Sternidae) and their phylogenetic implications. Mitochondrial DNA Part B 2: 601-603.

Yang, C, Wang, QX, Li, XJ, Xiao, H & Huang, Y 2017b. Characterization of the mitogenomes for two sympatric breeding species in Recurvirostridae (Charadriiformes) and their phylogenetic implications. Mitochondrial DNA Part B 2: 182-184.

Yoon, TH, Kang, HE, Lee, SR, Lee, JB, Baeck, GW, Park, H & Kim, HW 2017. Metabarcoding analysis of the stomach contents of the Antarctic Toothfish (*Dissostichus mawsoni*) collected in the Antarctic Ocean. PeerJ 5, e3977. (suppl data 2).

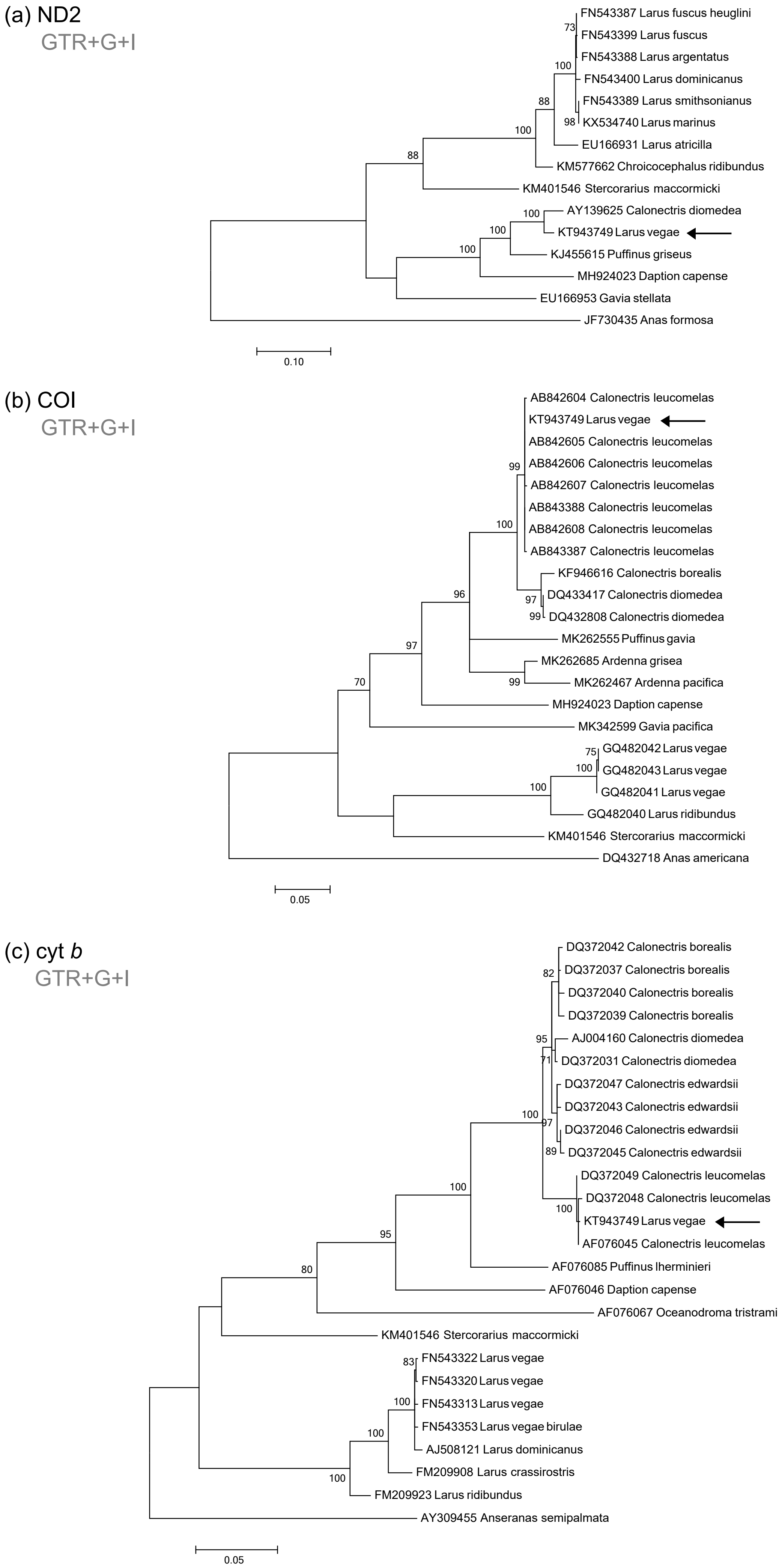

Figure S22. Maximum Likelihood phylogenies of *Larus vegae* (KT943749) and related taxa based on mitochondrial sequences. Numbers at branches are bootstrap support values (>70%) based on 1000 replicates.

27. “*Hydrobates castro*” MH433599, NC_041251 (Jiang et al. 2018)

Fig. S23

Metadata: “the feet of three dead adults were collected at Azores, Portugal (38°60’N, 28°80’W) and stored in lysis buffer. ... Both tissue and extracted genomic DNA was stored in the Zoology Comprehensive Laboratory from College of Animal Science and Veterinary Medicine.” (Jiang et al. 2018: 1193).

Phylogenetic position:

ND2 = could not be verified due to the lack of sequences of *Hydrobates castro* and *Hydrobates jabejabe*.

CO1 = sister to *H. castro* and *H. monteiroi* (as expected if this is actually *H. jabejabe*); no sequences of *H. jabejabe* available

Cyt *b* = *Hydrobates jabejabe*

Sequence integrity: not investigated

Interpretation: misidentified due to outdated taxonomy

Subsequent usage: no subsequent uses yet (January 2020)

Problems noted in other works: none.

Relevance: This was one of two mitogenomes presumed to be of this species (Apr 2019). The other (MK170187) was also misidentified.

References:

Jiang, JQ, Wang, QH & Gao, TZ 2018. Characterization of the complete mitochondrial genome of band-rumped Storm-petrel, *Hydrobates castro*. Mitochondrial DNA Part B 3: 1193-1194.

28. “*Hydrobates castro*” MK170187 (Antaky et al. 2019)

Fig. S23

Metadata: “Blood, tissue, and feather samples from 25 individuals were collected on the islands of Kaua‘i, Hawai‘i, Maui, and O‘ahu. Samples from museum specimens are stored at the Bernice Pauahi Bishop Museum.” (Antaky et al. 2019: 1271). On the next page, however, the collection site was stated to be “from 19°38’N, -155°32’E” which corresponds to the island of Hawai‘i.

Phylogenetic position:

ND2 = could not be verified due to the absense of sequences of *Hydrobates castro* and *H. leucorhous*.

CO1 = *Hydrobates leucorhous*

Cyt *b* = *Hydrobates leucorhous*

Sequence integrity: not investigated

Interpretation: misidentified

Subsequent usage: no subsequent uses yet (January 2020).

Problems noted in other works: none.

Relevance: This was one of two mitogenomes presumed to be of this species (Apr 2019). The other (MH433599) was also misidentified.

References:

Antaky, CC, Kitamura, PK, Knapp, IS, Toonen, RJ & Price, MR 2019. The complete mitochondrial genome of the Band-rumped Storm Petrel (*Oceanodroma castro*). Mitochondrial DNA Part B 4: 1271-1272.

(a) COI

GTR+G+I

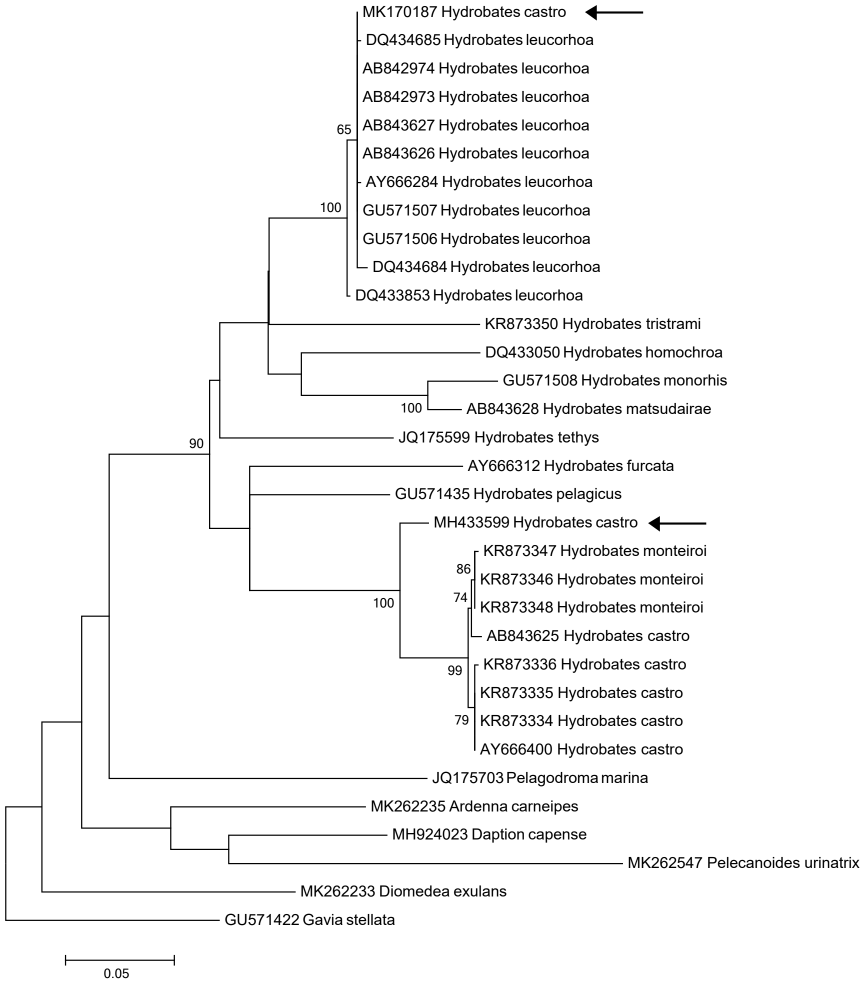

(b) cyt *b*

GTR+G+I

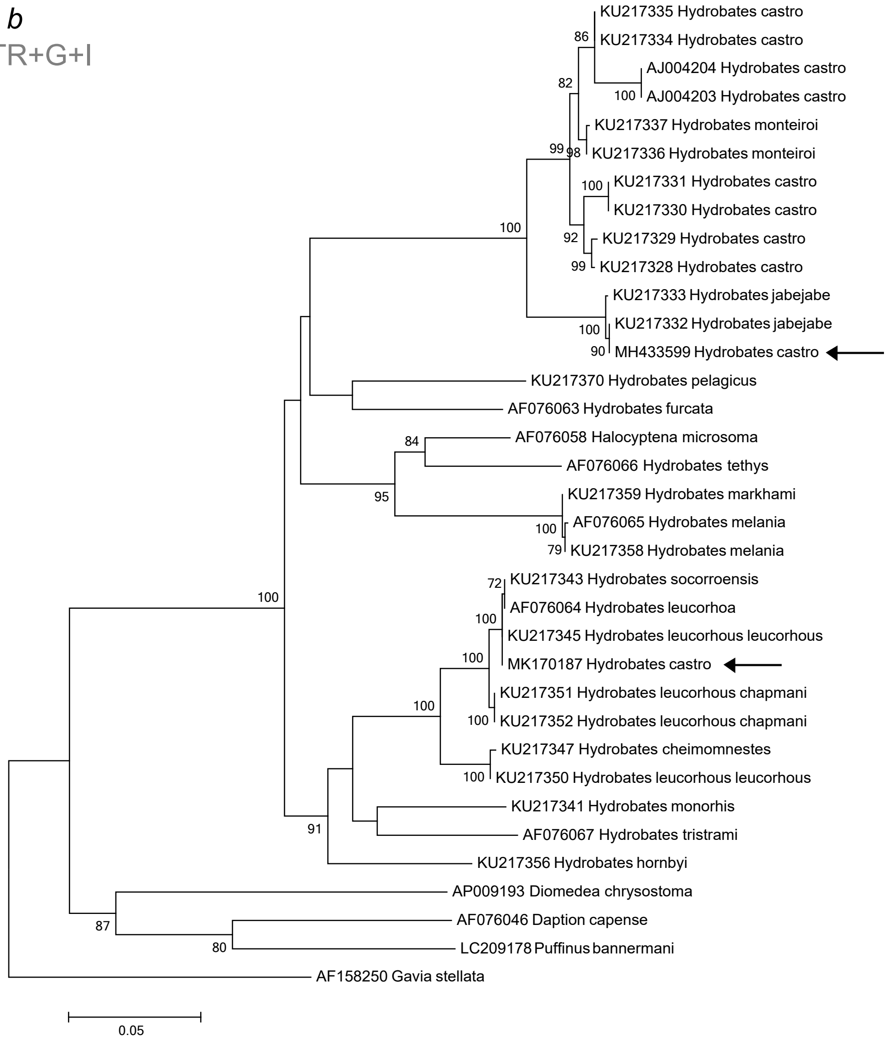

Figure S23. Maximum Likelihood phylogenies of *Hydrobates castro* (MH433599, MK170187) and related taxa based on mitochondrial sequences. Numbers at branches are bootstrap support values (>70%) based on 1000 replicates.

29. “*Aquila heliaca*” KU646835, NC_035806 (Zhou, L. and Dong, Y., unpublished; 2016)

Fig. S23

Metadata: no data on GenBank.
Phylogenetic position:
ND2 = *Aquila chrysaetos*
CO1 = *Aquila chrysaetos*
Cyt *b* = *Aquila chrysaetos*
Sequence integrity: not investigated
Interpretation: misidentified
Subsequent usage:
Re-used in **4 mitogenomic phylogenies published before 1 January 2020**: Sarker et al. 2019a, Knapp et al. 2019, Zhou et al. 2019c, Xu et al. 2019.
Also used by **1 further study**: Grealy et al. (2019).
Problems noted in other works: none. A phylogeny which showed very shallow divergence between *Aquila heliaca* and *A. chrysaetos* was published by Zhou et al. (2019c)
Relevance: This was the only mitogenome presumed to be of this species (January 2020).
References:

Grealy, A, Bunce, M & Holleley, C 2019. Avian mitochondrial genomes retrieved from museum eggshell. Molecular Ecology Resources 19: 1052-1062.

Knapp, M, Thomas, JE, Haile, J, Prost, S, Ho, SY, Dussex, N, Cameron-Christie, S, Kardailsky, O, Barnett, R, Bunce, M, Gilbert, MTP & Scofield, RP 2019. Mitogenomic evidence of close relationships between New Zealand’s extinct giant raptors and small-sized Australian sister-taxa. Molecular Phylogenetics and Evolution 134: 122-128.

Sarker, S, Das, S, Forwood, JK & Raidal, SR 2019a. The first complete mitogenome of Australia’s largest raptor, the Wedge-tailed Eagle (*Aquila audax*). Mitochondrial DNA Part B 4: 434-436.

Xu, N, Zhang, Q, Chen, R & Liu, H 2019. The complete mitogenome of red-collared lorikeet (*Trichoglossus rubritorquis*) and its phylogenetic analysis. Mitochondrial DNA Part B 4: 3116-3117.

Zhou, C, Tu, H, Chen, Y, Dou, L, Meng, Y, Yang, N, Yue, B & Wu, Y 2019c. The complete mitochondrial genome of *Aquila nipalensis* and its phylogenetic position. Mitochondrial DNA Part B 4: 2152-2153.

30. “*Aquila audax*” MG873530 (Sarker et al. 2019a)

Fig. S24

Metadata: “The blood sample used in this study was sourced from a Wedge-tailed eagle (*A. audax*) in the wild (year of sampling: 2017; GPS location: 32°16’42.905’’S, 148°34’54.344’’E), was stored in appropriate conditions by the Veterinary Diagnostic Laboratory (VDL), Charles Sturt University under the accession number CS17-0090.” (Sarker et al. 2019a: 434)

Phylogenetic position:

ND2 = clustered with one *A. audax* but on a rather long branch (sequence divergence 2.6%, uncorrected p-distance, with complete deletion of missing sites)

CO1 = sister to all *Aquila* and on a very long branch; no reference sequences of *A. audax* available.

Cyt *b* = sister to all *A. audax*, but on a long branch (sequence divergence 6.9-7.6%, uncorrected p-distance, with complete deletion of missing sites)

Sequence integrity:

ND2: in an alignment with one *A. audax* (AY987124) there are inferred deletions at positions 204, 247, 272, 296-297, 315, 317, 320, 324, 326-328 and 846-847.

COI: in an alignment with multiple Accipitridae, there are inferred deletions (gaps) at positions 290-291 and 402-419.

Cyt b: in an alignment with four *A. audax* (AJ604484, AY987302, AY754048, EU345514) there are inferred deletions at positions 301, 744, 747, 840, 853 and 867. In addition, there was an insertion at position 474 (G).

Interpretation: sequencing errors / numts

Subsequent usage: no citations or subsequent uses (January 2020).

Problems noted in other works: none.

Relevance: Another mitogenome of this species was published by Grealy et al. (2019).

References:

Grealy, A, Bunce, M & Holleley, C 2019. Avian mitochondrial genomes retrieved from museum eggshell. Molecular Ecology Resources 19: 1052-1062.

Sarker, S, Das, S, Forwood, JK & Raidal, SR 2019a. The first complete mitogenome of Australia’s largest raptor, the Wedge-tailed Eagle (*Aquila audax*). Mitochondrial DNA Part B 4: 434-436.

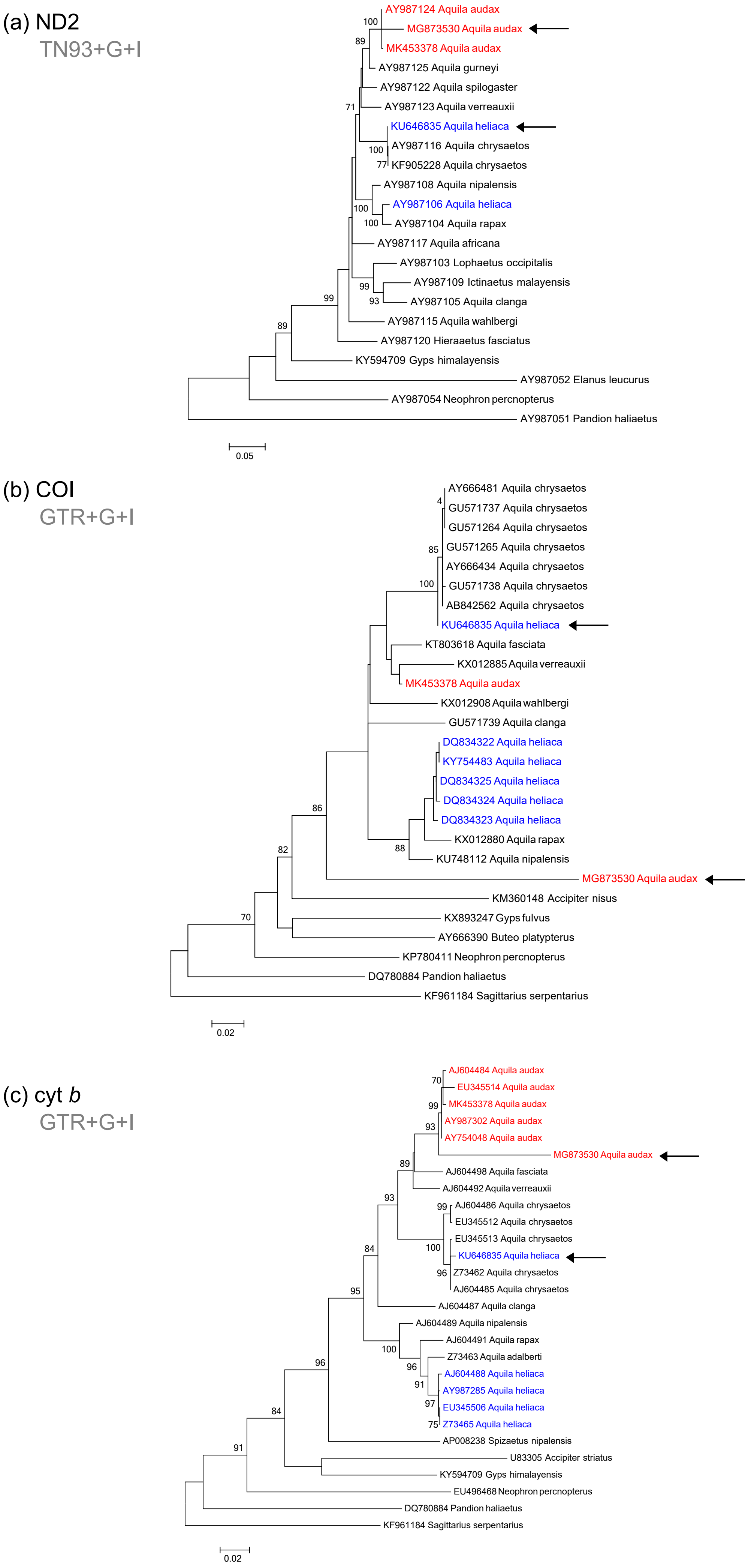

Figure S24. Maximum Likelihood phylogenies of *Aquila heliaca* (KU646835), *A. audax* (MG873530) and related taxa based on mitochondrial sequences. Numbers at branches are bootstrap support values (>70%) based on 1000 replicates.

31. “*Accipiter gularis*” KX585864 (Liu et al. 2017a)

Fig. S25

Metadata: “Blood sample of a rescued injured Japanese sparrowhawk was collected using non-invasive methods in Hefei city, Anhui province in October 2015.” (Liu et al. 2017a: 117).

Phylogenetic position:
ND2 = close to *Buteo buteo burmanicus*
CO1 = *Streptopelia orientalis*
Cyt *b* = close to *Buteo buteo burmanicus*, on a long branch

Sequence integrity:
ND2 bp 136 – 1034: 99.9% similarity to *Buteo buteo burmanicus*. Insertion of 39 bp at pos. 1036.

cyt *b* bp 1 – 84 = 100% similarity to *Accipiter virgatus* (BLAST) but no sequences of *A. gularis* available for this part; bp 85 – 1035 = *Buteo buteo burmanicus* (1 bp difference); bp 1036 – 1044 = unknown (6 nc differences from both *Accipiter gularis* and *B. b. burmanicus*); bp 1047 – 1143 = identical to *Accipiter gularis*.

Interpretation: chimera of three species: *Buteo b. burmanicus* (most of cyt *b* and ND2), *Streptopelia orientalis* (COI) and *Accipiter gularis* (parts of cyt b).

Subsequent usage:
Re-used in **4 mitogenomic phylogenies published before 1 January 2020**: Jeon et al. 2018, Kim et al. 2019 (***A. gularis* has long branch in phylogeny**), Liu et al. 2019a, Yang et al. 2019.

Problems noted in other works: none. The paper by Liu et al. (2017a) includes incorrect, falsified figures. A detailed investigation of this case was presented by Sangster & Luksenburg (2021).

Relevance: This was the only mitogenome presumed to be of this species (January 2020).

References:

Jeon, HS, Myeong, H, Kang, SG, Kim, JA, Lee, SH, Lee, MY & An, J 2018. The mitochondrial genome of *Milvus migrans* (Aves, Accipitriformes, Accipitridae), an endangered species from South Korea. Mitochondrial DNA Part B 3: 498-499.

Kim, JA, Kang, SG, Jeon, HS, Jeon, JH, Jang, JH, Kim, S & An, J 2019. Complete mitogenomes of two Accipitridae, *Haliaeetus albicilla*, and *Pernis ptilorhynchus*. Mitochondrial DNA Part B 4: 391-392.

Liu, G, Li, C, Du, Y & Liu, X 2017a. The complete mitochondrial genome of Japanese sparrowhawk (*Accipiter gularis*) and the phylogenetic relationships among some predatory birds. Biochemical Systematics and Ecology 70: 116-125.

Liu, G, Zhou, L & Zhao, G 2019a. Complete mitochondrial genomes of five raptors and implications for the phylogenetic relationships between owls and nightjars. PeerJ Preprints, e27478v1.

Sangster, G & Luksenburg, JA 2021. Scientific data laundering: chimeric mitogenomes of a sparrowhawk and a nightjar covered-up by forged phylogenies. Biochemical Systematics and Ecology 96: 104263.

Yang, C, Yang, M, Wang, Q, Lu, Y & Li, X 2018. The complete mitogenome of *Falco amurensis* (Falconiformes, Falconidae), and a comparative analysis of genus *Falco*. Zoological Science 35: 367-373.

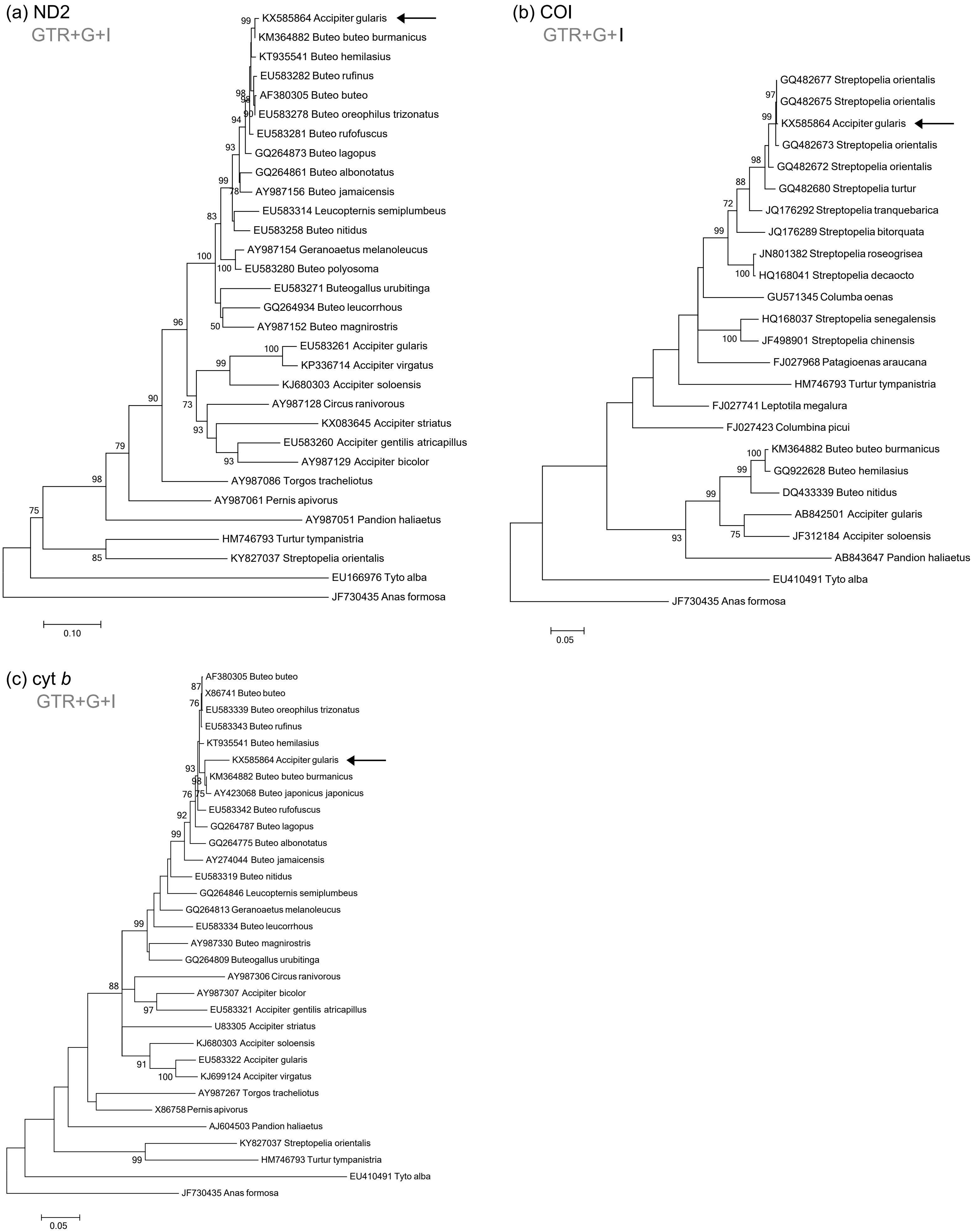

Figure S25. Maximum Likelihood phylogenies of *Accipiter gularis* (KX585864) and related taxa based on mitochondrial sequences. Numbers at branches are bootstrap support values (>70%) based on 1000 replicates.

32. “*Tyto longimembris*” KP893332 (Xu et al. 2016b)

Fig. S26

Metadata: “One individual was sampled in the Nanji Wetland National Nature Reserve of Poyang Lake, Jiangxi Province, China.” (Xu et al. 2016b: 2481).
Phylogenetic position:
ND2 = *Buteo buteo burmanicus* (identical to KM364882)
CO1 = *Tyto longimembris*, but on a very long branch
Cyt *b* = *Tyto longimembris*
Sequence integrity: COI bp 1 – 635 = *Tyto longimembris*; bp 636 insertion G; bp 640 insertion C; bp 643 insertion G. Thus, alignment from bp 643 – 696 has shifted by 3 positions,
Interpretation: chimera + three frameshift mutations in COI
Subsequent usage:
Re-used in **5 mitogenomic phylogenies published before 1 January 2020**: Liu et al. 2017a, Kang et al. 2018, Spiridonova & Surmach 2018, Liu et al. 2019a, Zhou et al. 2019a.
Problems noted in other works: None.
Relevance: This was the only mitogenome presumed to be of this species (January 2020).
References:

Kang, H, Li, B, Ma, X & Xu, Y 2018. Evolutionary progression of mitochondrial gene rearrangements and phylogenetic relationships in Strigidae (Strigiformes). Gene 674: 8-14.

Liu, G, Li, C, Du, Y & Liu, X 2017a. The complete mitochondrial genome of Japanese sparrowhawk (*Accipiter gularis*) and the phylogenetic relationships among some predatory birds. Biochemical Systematics and Ecology 70: 116-125.

Liu, G, Zhou, L & Zhao, G 2019a. Complete mitochondrial genomes of five raptors and implications for the phylogenetic relationships between owls and nightjars. PeerJ Preprints, e27478v1.

Spiridonova, LN & Surmach, SG 2018. Whole mitochondrial genome of Blakiston’s Fish Owl *Bubo* (*Ketupa*) *blakistoni* suggests its redescription in the genus *Ketupa*. Russian Journal of Genetics 54: 369-373.

Xu, P, Li, Y, Miao, L, Xie, G & Huang, Y 2016b. Complete mitochondrial genome of the *Tyto longimembris* (Strigiformes: Tytonidae). Mitochondrial DNA Part A 27: 2481-2482.

Zhou, C, Chen, Y, Hao, Y, Meng, Y, Yue, B & Zeng, T 2019a. Characterization of the complete mitochondrial genome and phylogenetic analysis of *Otus sunia* (Strigiformes: Strigidae). Mitochondrial DNA Part B 4: 804-805.

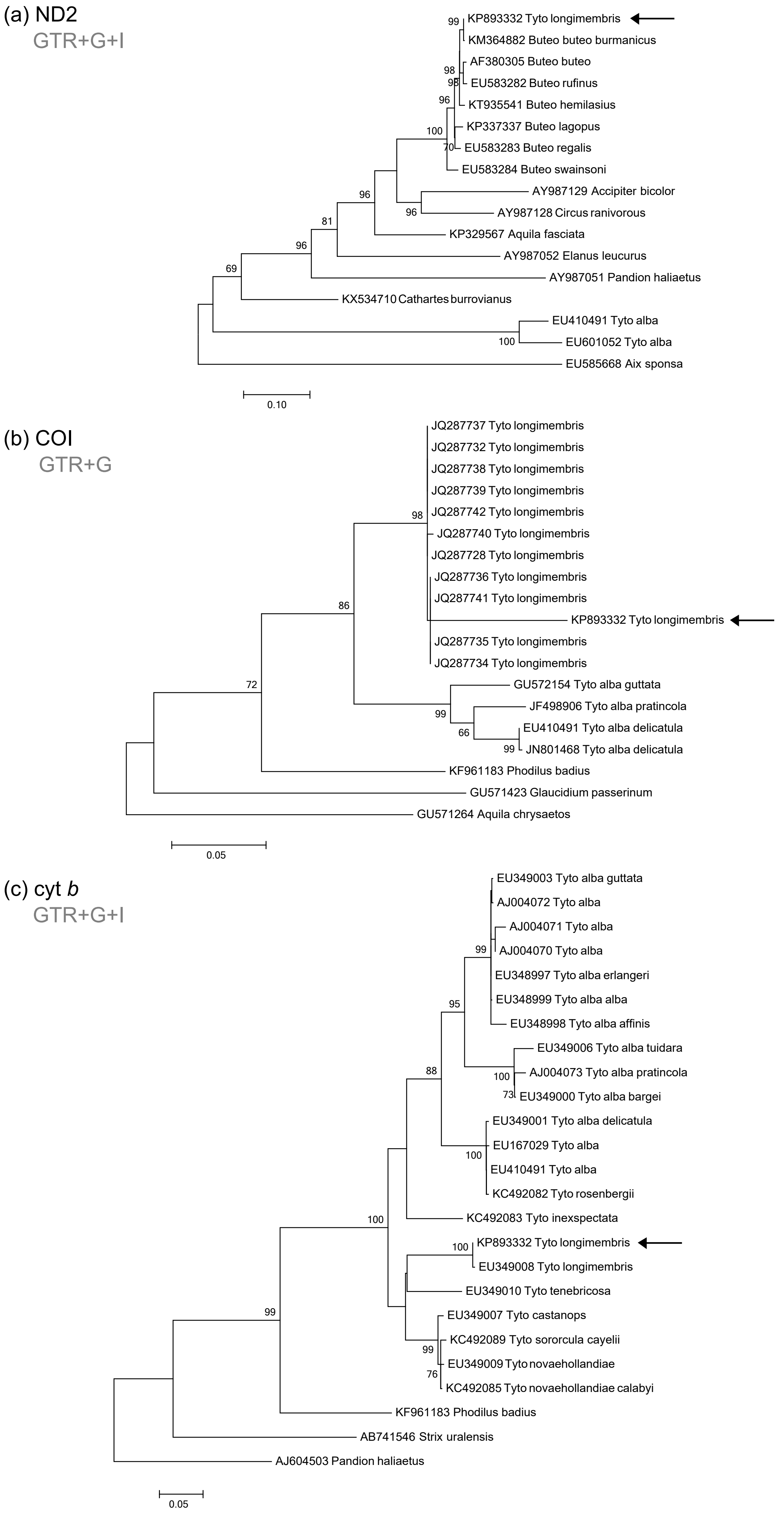

Figure S26. Maximum Likelihood phylogenies of *Tyto longimembris* (KP893332) and related taxa based on mitochondrial sequences. Numbers at branches are bootstrap support values (>70%) based on 1000 replicates.

33. “*Otus bakkamoena*” KT340631, NC_028163 (Park et al. 2019b)

Fig. S27

Metadata: “The carcass of *O. bakkamoena* was collected at Yeongju-si Branch of Korea Society for the Protection of Wild Animals in Yeongu-si city (36.852521 N 128.576801 E), Republic of Korea. This specimen was deposited in the Institute of Ornithology, Kyungpook National University, Daegu, Republic of Korea.” (Park et al. 2019b: 775)

Phylogenetic position:

ND2 = in a clade with *Sternula albifrons, S. antillarum, S. superciliaris* and *S. nereis*. Differed from two *S. albifrons* (AY631366, KT350612) by 4.6-5.4%, and from one *O. semitorques ussuriensis* (EU601035) by 19.3% (p-distance, with complete deletion of missing sites).

CO1 = *Sternula albifrons* (identical)

Cyt *b* = *Otus semitorques*

Sequence integrity: ND2: first part (bp 1 – circa 534) = *Sternula albifrons*; second part (circa bp 537 – 695) = *Otus semitorques ussuriensis*; third part (circa bp 699 – 1041) = *Sternula albifrons*.

Interpretation: chimera; with cyt *b* pure *O. semitorques*, COI pure *S. albifrons*, and ND2 a chimera of both species, but mostly *S. albifrons*.

Subsequent usage:

Re-used in **6 mitogenomic phylogenies published before 1 January 2020**: Sarker et al. 2016, Sun et al. 2017c, Liu et al. 2017a, Kang et al. 2018, Spiridonova & Surmach 2018, Lee et al. 2018.

Problems noted in other works: None.

Relevance: This was the only mitogenome presumed to be of this species (January 2020).

References:

Kang, H, Li, B, Ma, X & Xu, Y 2018. Evolutionary progression of mitochondrial gene rearrangements and phylogenetic relationships in Strigidae (Strigiformes). Gene 674: 8-14.

Lee, MY, Lee, SM, Jeon, HS, Lee, SH, Park, JY & An, J 2018. Complete mitochondrial genome of the Northern Long-eared Owl (*Asio otus* Linnaeus, 1758) determined using next-generation sequencing. Mitochondrial DNA Part B 3: 494-495.

Liu, G, Li, C, Du, Y & Liu, X 2017a. The complete mitochondrial genome of Japanese sparrowhawk (*Accipiter gularis*) and the phylogenetic relationships among some predatory birds. Biochemical Systematics and Ecology 70: 116-125.

Park, CE, Kim, MC, Ibal, JCP, Pham, HQ, Park, HC & Shin, JH 2019b. The complete mitochondrial genome sequence of *Otus bakkamoena* (Aves, Strigiformes, Strigidae). Mitochondrial DNA Part B 4: 775-776.

Sarker, S, Das, S, Forwood, J, Helbig, K & Raidal, SR 2016. The complete mitochondrial genome sequence of an Endangered powerful owl (*Ninox strenua*). Mitochondrial DNA Part B 1: 722-723.

Spiridonova, LN & Surmach, SG 2018. Whole mitochondrial genome of Blakiston’s Fish Owl *Bubo* (*Ketupa*) *blakistoni* suggests its redescription in the genus *Ketupa*. Russian Journal of Genetics 54: 369-373.

Sun, X, Zhao, R, Zhang, T, Gong, J, Jing, M & Huang, L 2017c. Two mitochondrial genomes in Alcedinidae (*Ceryle rudis*/*Halcyon pileata*) and the phylogenetic placement of Coraciiformes. Genetica 145: 431-440.

34. “*Otus scops*” KT340630, NC_028162 (Park et al. 2019a)

Fig. S27

Metadata: “*O scops* was collected by at Yeongju-si Branch of Korea Society for the Protection of Wild Animals in Yeongu-si city (36.816655 N 128.480288 E), Republic of Korea. This specimen was deposited in Institute of Ornithology, Kyungpook National University, Daegu, Republic of Korea.” (Park et al. 2019a: 764)

Phylogenetic position:

ND2 = *Otus sunia*

CO1 = *Otus sunia*

Cyt *b* = *Otus sunia*

Sequence integrity: not investigated

Interpretation: misidentification due to outdated taxonomy

Subsequent usage:

Re-used in **8 mitogenomic phylogenies published before 1 January 2020**: Sarker et al. 2016, Liu et al. 2017a, Sun et al. 2017c, Kang et al. 2018, Spiridonova & Surmach 2018, Lee et al. 2018, Zhou et al. 2019a, Park et al. 2019b.

The authors apparently did not realise that their sample (collected in Korea) was not from *O. scops*, despite their statement that “*O scops* breeds in southern Europe eastwards into western and central Asia.” (Park et al. 2019a: 764)

Problems noted in other works: None.

Relevance: This is one of two mitogenomes presumed of this species (January 2020). The other mitogenome (KY471456) was also misidentified.

References:

Kang, H, Li, B, Ma, X & Xu, Y 2018. Evolutionary progression of mitochondrial gene rearrangements and phylogenetic relationships in Strigidae (Strigiformes). Gene 674: 8-14.

Liu, G, Li, C, Du, Y & Liu, X 2017a. The complete mitochondrial genome of Japanese sparrowhawk (*Accipiter gularis*) and the phylogenetic relationships among some predatory birds. Biochemical Systematics and Ecology 70: 116-125.

Lee, MY, Lee, SM, Jeon, HS, Lee, SH, Park, JY & An, J 2018. Complete mitochondrial genome of the Northern Long-eared Owl (*Asio otus* Linnaeus, 1758) determined using next-generation sequencing. Mitochondrial DNA Part B 3: 494-495.

Park, CE, Kim, MC, Ibal, JCP, Pham, HQ, Park, HC & Shin, JH 2019a. The complete mitochondrial genome sequence of *Otus scops* (Aves, Strigiformes, Strigidae). Mitochondrial DNA Part B 4: 764-765.

Park, CE, Kim, MC, Ibal, JCP, Pham, HQ, Park, HC & Shin, JH 2019b. The complete mitochondrial genome sequence of *Otus bakkamoena* (Aves, Strigiformes, Strigidae). Mitochondrial DNA Part B 4: 775-776.

Sarker, S, Das, S, Forwood, J, Helbig, K & Raidal, SR 2016. The complete mitochondrial genome sequence of an Endangered powerful owl (*Ninox strenua*). Mitochondrial DNA Part B 1: 722-723.

Spiridonova, LN & Surmach, SG 2018. Whole mitochondrial genome of Blakiston’s Fish Owl *Bubo* (*Ketupa*) *blakistoni* suggests its redescription in the genus *Ketupa*. Russian Journal of Genetics 54: 369-373.

Sun, X, Zhao, R, Zhang, T, Gong, J, Jing, M & Huang, L 2017c. Two mitochondrial genomes in Alcedinidae (*Ceryle rudis*/*Halcyon pileata*) and the phylogenetic placement of Coraciiformes. Genetica 145: 431-440.

Zhou, C, Chen, Y, Hao, Y, Meng, Y, Yue, B & Zeng, T 2019a. Characterization of the complete mitochondrial genome and phylogenetic analysis of *Otus sunia* (Strigiformes: Strigidae). Mitochondrial DNA Part B 4: 804-805.

35. “*Otus scops*” KY471456 (Liu et al. 2019a)

Fig. S27

Metadata: “The *G. cuculoides*, *O. scops*, and *G. brodiei* tissue samples were collected from dead birds that were killed by bird repellent at Hefei Xinqiao International Airport, Anhui Province, China.” (Liu et al. 2019: 8).

Phylogenetic position:

ND2 = *Otus sunia*

CO1 = *Otus sunia*

Cyt *b* = *Otus sunia*

Sequence integrity: not investigated

Interpretation: misidentification due to outdated taxonomy

Subsequent usage: not cited or used in other papers (January 2020)

Problems noted in other works: None.

Relevance: This is one of two mitogenomes presumed of this species (January 2020). The other mitogenome (KT340630) was also misidentified.

References:

Liu, G, Zhou, L & Zhao, G 2019a. Complete mitochondrial genomes of five raptors and implications for the phylogenetic relationships between owls and nightjars. PeerJ Preprints, e27478v1.

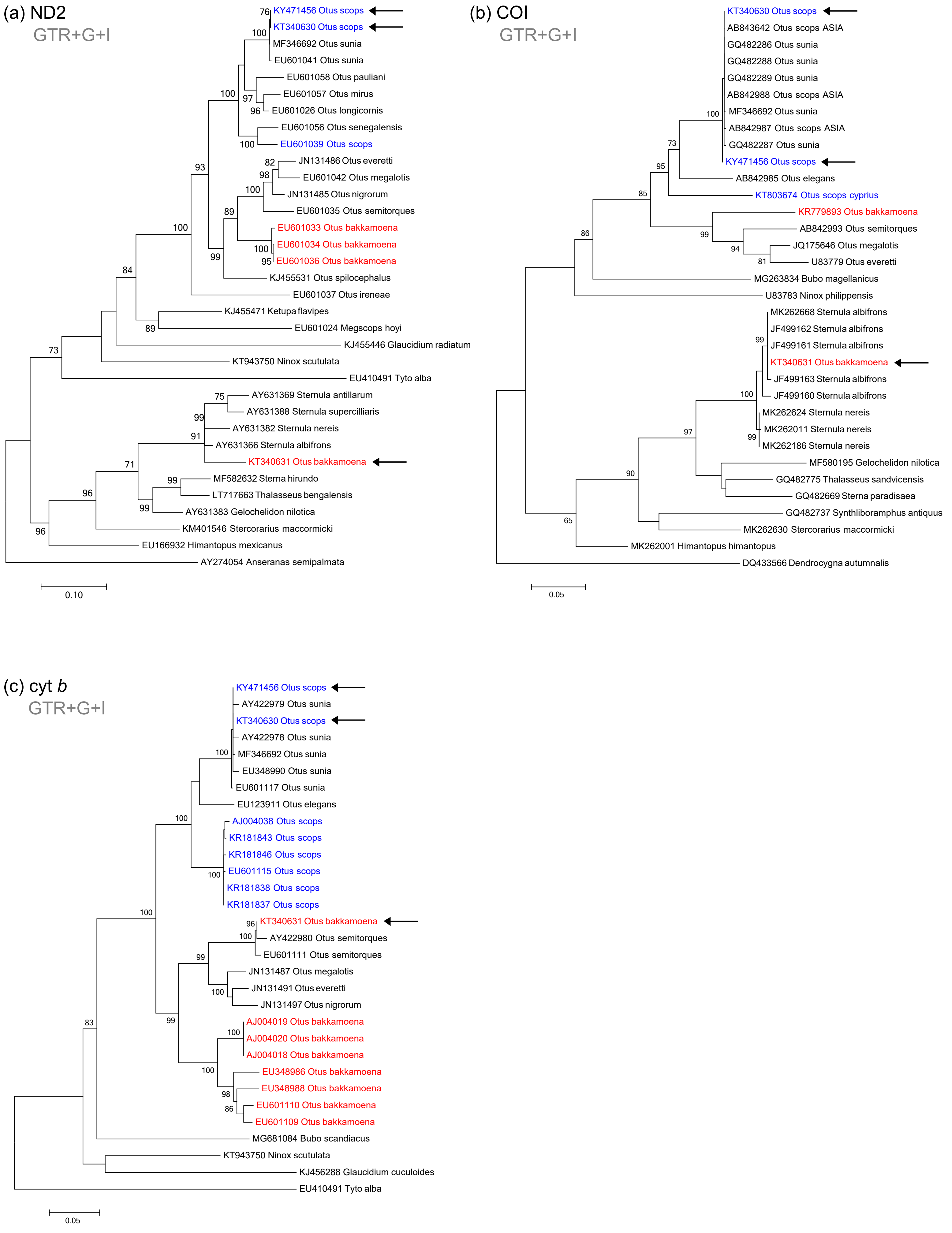

Figure S27. Maximum Likelihood phylogenies of *Otus bakkamoena* (KT340631), two *O. scops* (KT340630 and KY471456) and related taxa based on mitochondrial sequences. Numbers at branches are bootstrap support values (>70%) based on 1000 replicates.

36. “*Bubo bubo*” AB918148 (Tian et al. 2016)

Fig. S28

Metadata: No details about locality or voucher, or how ID was determined. GenBank entry says: country="China"

Phylogenetic position:

ND2 = *Bubo bubo*

CO1 = *Bubo bubo* but on a very long branch

Cyt *b* = among *Ninox* but did not match any species for which sequences are available

Sequence integrity:

ND2: insertions at pos. 1020C, 1036C.

COI: insertions at pos. 259G, 659A, 681T.

In the cyt *b* part of AB918148 there are at least 34 nucleotides that are unique, not just among *Bubo*, but among all Strigiformes and Accipitriformes. These are: 14A, 73G, 116C, 151G, 173G, 196A, 221A, 298C, 308A, 343T, 368G, 377G, 385A, 454A, 489T, 521T, 532G, 538A, 553G, 611G, 617G, 670A, 694C, 710A764T, 772T, 803T, 848C, 896A, 925G, 1023G, 1047A, 1079A, 1089G.

Interpretation: sequencing errors / partial numt.

Subsequent usage:

Re-used in **4 mitogenomic phylogenies published before 1 January 2020**: Spiridonova & Surmach 2018, Liu et al. 2019a, Sarker et al. 2016, Xu et al. 2016b

Cited by **3 other papers**: Hanna et al. 2017a, Hanna et al. 2017b, Kang et al. 2018.

Erroneous taxonomic conclusions: AB918148 was placed near two species of *Ninox*, rather than near four species of fish owls (*Bubo*), in a mitogenomic tree in Spiridonova & Surmach (2018). These authors stated that these findings “support the earlier taxonomic classification according to which all four Asian forms, *blakistoni*, *flavipes*, *zeylonensis*, and *ketupu*, constituted a separate *Ketupa* genus.”.

Problems noted in other works: Kang et al. (2018: 8): “The **ancestral gene order** in the previously published mitogenome of *B. bubo* was found to be incorrect.” Kang et al. (2018) excluded this mitogenome from their phylogenetic analysis due to numerous ambiguities in its PCGs.

Relevance: Another mitogenome of *Bubo bubo* was published by Kang et al. (2018).

References:

Hanna, ZR, Henderson, JB, Sellas, AB, Fuchs, J, Bowie, RCK & Dumbacher, JP 2017a. Complete mitochondrial genome sequences of the northern spotted owl (*Strix occidentalis caurina*) and the barred owl (*Strix varia*; Aves: Strigiformes: Strigidae) confirm the presence of a duplicated control region. PeerJ 5, e3901.

Hanna, ZR, Henderson, JB, Wall, JD, Emerling, CA, Fuchs, J, Runckel, C, Mindell, DP, Bowie, RCK, DeRisi, JL & Dumbacher, JP 2017b. Northern spotted owl (*Strix occidentalis caurina*) genome: divergence with the barred owl (*Strix varia*) and characterization of light-associated genes. Genome Biology and Evolution 9: 2522-2545.

Kang, H, Li, B, Ma, X & Xu, Y 2018. Evolutionary progression of mitochondrial gene rearrangements and phylogenetic relationships in Strigidae (Strigiformes). Gene 674: 8-14.

Liu, G, Zhou, L & Zhao, G 2019a. Complete mitochondrial genomes of five raptors and implications for the phylogenetic relationships between owls and nightjars. PeerJ Preprints, e27478v1.

Sarker, S, Das, S, Forwood, J, Helbig, K & Raidal, SR 2016. The complete mitochondrial genome sequence of an Endangered powerful owl (*Ninox strenua*). Mitochondrial DNA Part B 1: 722-723.

Spiridonova, LN & Surmach, SG 2018. Whole mitochondrial genome of Blakiston’s Fish Owl *Bubo* (*Ketupa*) *blakistoni* suggests its redescription in the genus *Ketupa*. Russian Journal of Genetics 54: 369-373.

Tian, H, Ji, J, Yang, S, Zhang, Z, Laghari, MY, Narejo, NT & Lashari, P 2016. Complete mitochondrial genome of Eagle Owl (*Bubo bubo*, Strigiformes; Strigidae) from China. Mitochondrial DNA Part A 27: 1455-1456.

Xu, P, Li, Y, Miao, L, Xie, G & Huang, Y 2016b. Complete mitochondrial genome of the *Tyto longimembris* (Strigiformes: Tytonidae). Mitochondrial DNA Part A 27: 2481-2482.

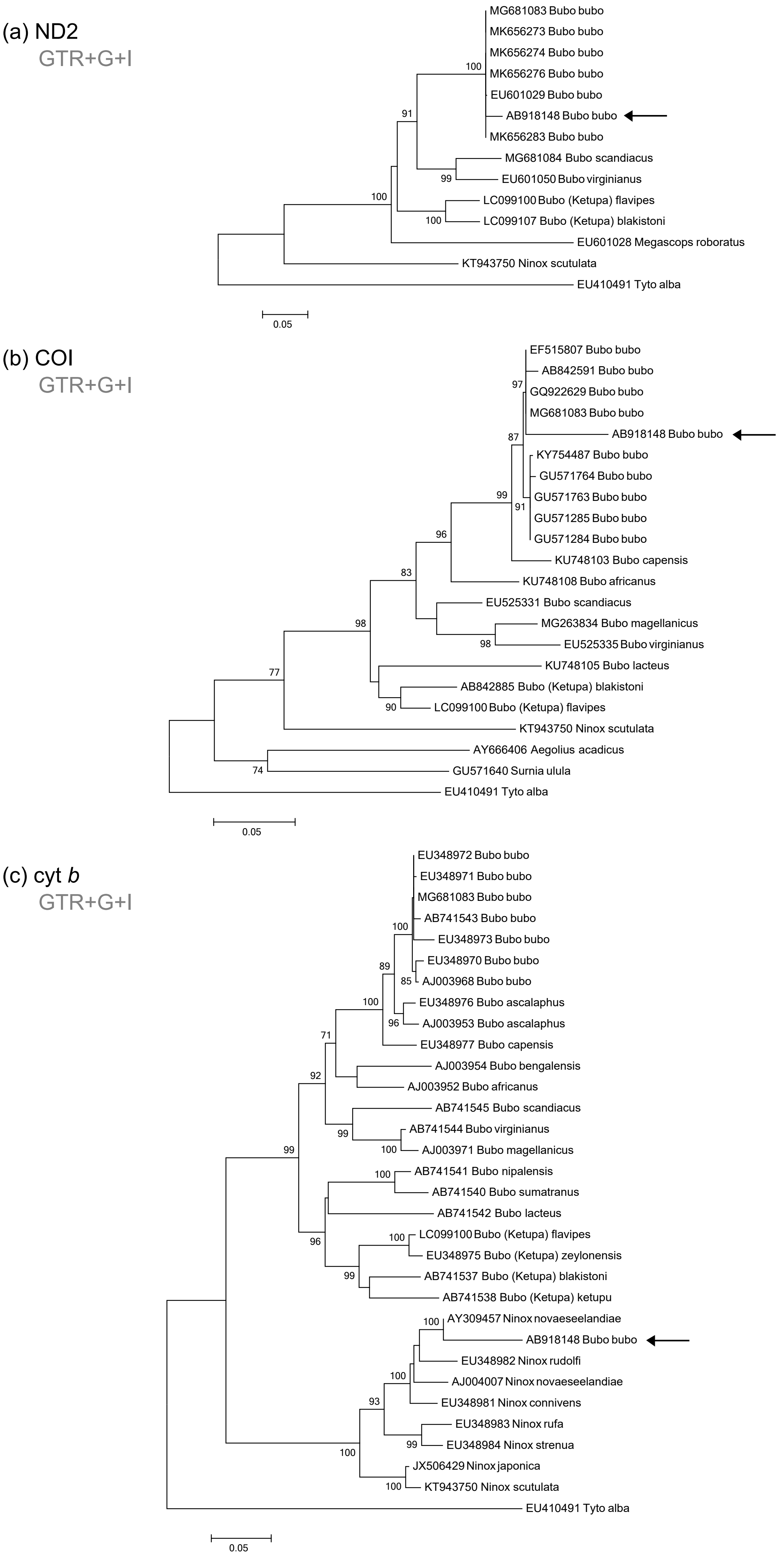

Figure S28. Maximum Likelihood phylogenies of *Bubo bubo* (AB918148) and related taxa based on mitochondrial sequences. Numbers at branches are bootstrap support values (>70%) based on 1000 replicates.

37. “*Strix leptogrammica*” KC953095, NC_021970 (record subsequently removed) (Liu et al. 2014, 2019a)

Fig. S29

Metadata: “the feather samples were collected from two young Brown wood owls rescued from Liao Yuan Village, Yinzhou Town, Jixi County, Anhui Province, China on 1 June 2012, and rearing in the South Anhui National Wild Animal Rescue Centre, Anhui Province, China.” (Liu et al. 2014: 370). “The *S. leptogrammica* feather samples were collected from two young rescued birds at the South Anhui National Wild Animal Rescue Centre, Anhui Province, China on June 1, 2012.” (Liu et al. 2019a: 8)

Phylogenetic position:

ND2 = *Strix leptogrammica*

CO1 = among *Strix*; no sequences available of *Strix leptogrammica*

Cyt *b* = among *Strix* on a very long branch

Sequence integrity: Cyt b: from position 936 to 1143 there are numerous substitutions that are unique among Strigidae. This fragment differs at least 15% from all other bird sequences.

ND2 and COI: no peculiarities.

Interpretation: Sequencing errors / numts

Subsequent usage:

Re-used in **13 mitogenomic phylogenies published before 1 January 2020:** Jiang et al. 2015, Sarker et al. 2016, Xu et al. 2016b, Eo 2017*,* Liu et al. 2017a, Spiridonova & Surmach 2018, Lee et al. 2018, Kang et al. 2018, Liu et al. 2019a, Zhou et al. 2019a, Park et al. 2019a (**long branch not noted**), Park et al. 2019b (**long branch not noted**), Jiang et al. 2019b.

Re-used in **1 non-mitogenomic phylogeny**: Sun et al. 2016e.

Cited by or used in **3 other papers**: Hanna et al. 2017a, Hanna et al. 2017b, Harvey et al. 2017 (**used for reference-based assembly of partial mitochondrial genomes in *Megascops***).

Problems noted in other works: Hanna et al. (2017a: 17): “Notably, this mitochondrial gene order was not reported as present in *S. leptogrammica* (Liu, Zhou & Gu, 2014). However, our alignments of this mitochondrial genome to our *S. o. caurina* and *S. varia* sequences as well as the sequences of other owl mitochondrial genomes indicated problems with the *S. leptogrammica* sequence from cyt *b* through ND6 to $tRNA^{Phe}$.

Spiridonova & Surmach (2018: 373): “It should be noted that, for some mitochondrial genomes deposited in the NCBI GenBank, we observed mismatches of certain nucleotide sequence regions to the general mtDNA structure. For example, *S. leptogrammica* mtDNA regions between the nucleotide positions 13100–13160 and 14610–14969 (KC953095) are markedly different from the corresponding regions of the mitochondrial genomes of other Strigiformes representatives. This is apparently the result of the incorrect genome assembly. After the above-mentioned positions in all the analyzed individual genomes were excluded from the analysis, the *S. leptogrammica* branch length decreased insignificantly with no effect on the general pattern of phylogenetic relationships between the *B. blakistoni* and *S. leptogrammica* clades.”

Relevance: This was the only mitogenome presumed to be of this species (January 2020).

References:

Eo, SH 2017. Complete mitochondrial genome of white-backed woodpecker *Dendrocopos leucotos* (Piciformes: Picidae) and its phylogenetic position. Mitochondrial DNA Part B 2: 451-452.

Hanna, ZR, Henderson, JB, Sellas, AB, Fuchs, J, Bowie, RCK & Dumbacher, JP 2017a. Complete mitochondrial genome sequences of the northern spotted owl (*Strix occidentalis caurina*) and the barred owl (*Strix varia*; Aves: Strigiformes: Strigidae) confirm the presence of a duplicated control region. PeerJ, 5, e3901.

Hanna, ZR, Henderson, JB, Wall, JD, Emerling, CA, Fuchs, J, Runckel, C, Mindell, DP, Bowie, RCK, DeRisi, JL & Dumbacher, JP 2017b. Northern spotted owl (*Strix occidentalis caurina*) genome: divergence with the barred owl (*Strix varia*) and

characterization of light-associated genes. Genome Biology and Evolution 9: 2522-2545.

Harvey, MG, Aleixo, A, Ribas, CC & Brumfield, RT 2017. Habitat association predicts genetic diversity and population divergence in Amazonian birds. American Naturalist 190: 631-648.

Jiang, L, Chen, J, Wang, P, Ren, Q, Yuan, J, Qian, C, Hua, X, Guo, Z, Zhang, L, Yang, J, Wang, Y, Zhang, Q, Ding, H, Bi, D, Zhang, Z, Wang, Q, Chen, D & Kan, X 2015. The mitochondrial genomes of *Aquila fasciata* and *Buteo lagopus* (Aves, Accipitriformes): sequence, structure and phylogenetic analyses. PloS one 10(8), e0136297.

Jiang, L, Peng, L, Tang, M, You, Z, Zhang, M, West, A, Ruan, G, Chen, W & Merilä, J 2019b. Complete mitochondrial genome sequence of the Himalayan Griffon, *Gyps himalayensis* (Accipitriformes: Accipitridae): Sequence, structure, and phylogenetic analyses. Ecology and Evolution 9: 8813–8828.

Kang, H, Li, B, Ma, X & Xu, Y 2018. Evolutionary progression of mitochondrial gene rearrangements and phylogenetic relationships in Strigidae (Strigiformes). Gene 674: 8-14.

Lee, MY, Lee, SM, Jeon, HS, Lee, SH, Park, JY & An, J 2018. Complete mitochondrial genome of the Northern Long-eared Owl (*Asio otus* Linnaeus, 1758) determined using next-generation sequencing. Mitochondrial DNA Part B 3: 494-495.

Liu, G, Li, C, Du, Y & Liu, X 2017a. The complete mitochondrial genome of Japanese sparrowhawk (*Accipiter gularis*) and the phylogenetic relationships among some predatory birds. Biochemical Systematics and Ecology 70: 116-125.

Liu, G, Zhou, L & Zhao, G 2019a. Complete mitochondrial genomes of five raptors and implications for the phylogenetic relationships between owls and nightjars. PeerJ Preprints, e27478v1.

Liu, G, Zhou, L & Gu, C 2014. The complete mitochondrial genome of Brown Wood Owl *Strix leptogrammica* (Strigiformes: Strigidae). Mitochondrial DNA 25: 370-371.

Park, CE, Kim, MC, Ibal, JCP, Pham, HQ, Park, HC & Shin, JH 2019a. The complete mitochondrial genome sequence of *Otus scops* (Aves, Strigiformes, Strigidae). Mitochondrial DNA Part B 4: 764-765.

Park, CE, Kim, MC, Ibal, JCP, Pham, HQ, Park, HC & Shin, JH 2019b. The complete mitochondrial genome sequence of *Otus bakkamoena* (Aves, Strigiformes, Strigidae). Mitochondrial DNA Part B 4: 775-776.

Sarker, S, Das, S, Forwood, J, Helbig, K & Raidal, SR 2016. The complete mitochondrial genome sequence of an Endangered powerful owl (*Ninox strenua*). Mitochondrial DNA Part B 1: 722-723.

Spiridonova, LN & Surmach, SG 2018. Whole mitochondrial genome of Blakiston's Fish Owl *Bubo* (*Ketupa*) *blakistoni* suggests its redescription in the genus *Ketupa*. Russian Journal of Genetics 54: 369-373.

Sun, X, Zhou, W, Sun, Z, Qian, L, Zhang, Y, Pan, T, & Zhang, B 2016e. The complete mitochondrial genome of *Glaucidium brodiei* (Strigiformes: Strigidae). Mitochondrial DNA Part A 27: 2508-2509.

Xu, P, Li, Y, Miao, L, Xie, G & Huang, Y 2016. Complete mitochondrial genome of the *Tyto longimembris* (Strigiformes: Tytonidae). Mitochondrial DNA Part A 27: 2481-2482.

Zhou, C, Chen, Y, Hao, Y, Meng, Y, Yue, B & Zeng, T 2019a. Characterization of the complete mitochondrial genome and phylogenetic analysis of *Otus sunia* (Strigiformes: Strigidae). Mitochondrial DNA Part B 4: 804-805.

(a) ND2

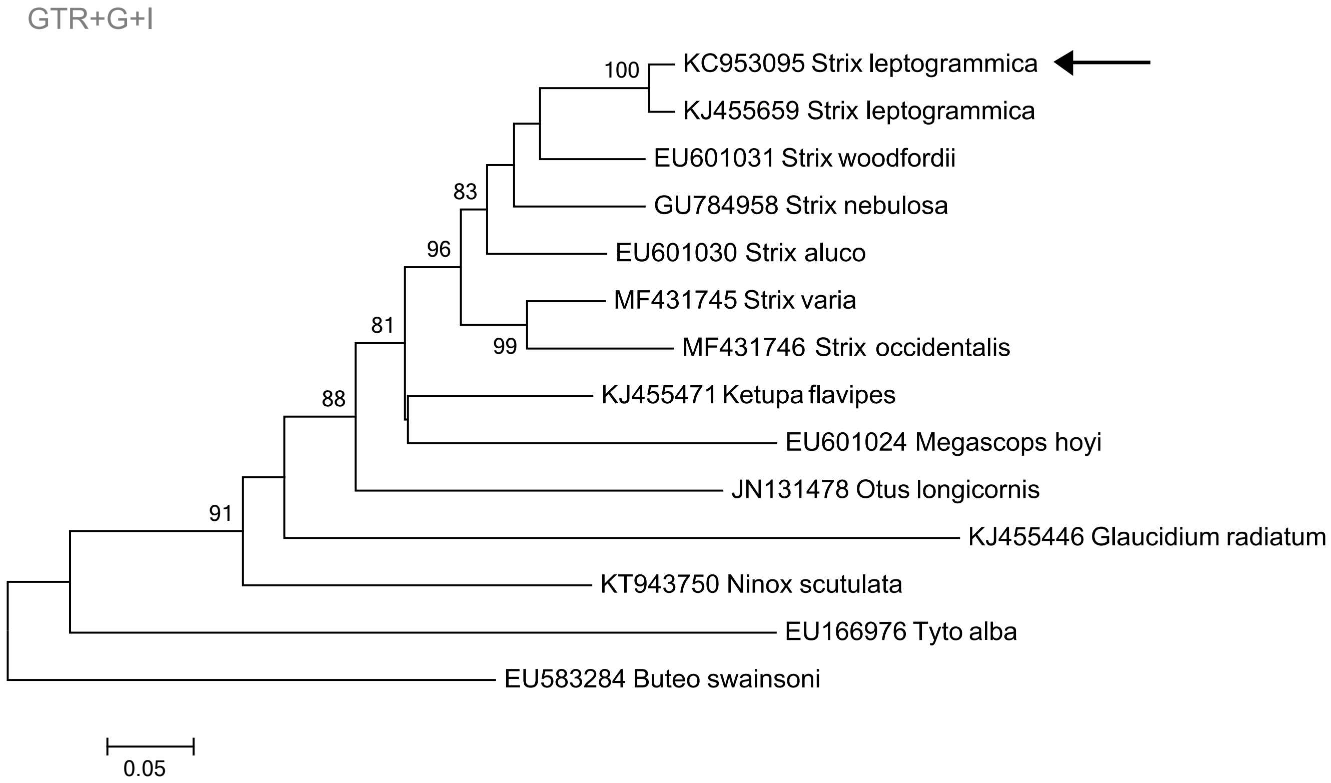

(b) cyt *b*

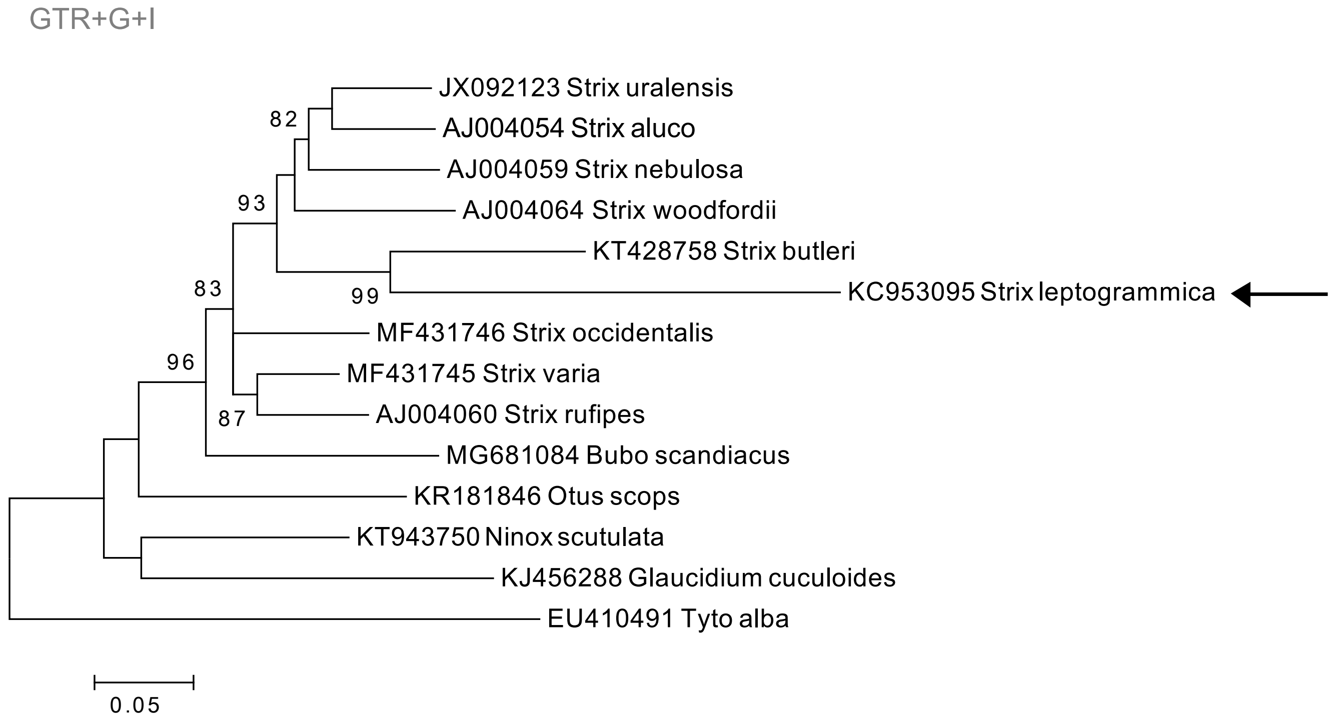

Figure S29. Maximum Likelihood phylogenies of *Strix leptogrammica* (KC953095) and related taxa based on mitochondrial sequences. Numbers at branches are bootstrap support values (>70%) based on 1000 replicates.

38. “*Glaucidium brodiei*” MF155890 (Liu et al. 2019a)

Fig. S30

Metadata: “The *G. cuculoides*, *O. scops*, and *G. brodiei* tissue samples were collected from dead birds that were killed by bird repellent at Hefei Xinqiao International Airport, Anhui Province, China.” (Liu et al. 2019a: 8).

Phylogenetic position:

ND2 = *Otus sunia*; identical to KY471456 '*Otus scops*' (=*O. sunia*), KT340630 '*Otus scops*' (=*O. sunia*)

CO1 = *Otus sunia*; identical to KY471456 '*Otus scops*' (=*O. sunia*), KT340630 '*Otus scops*' (=*O. sunia*)

Cyt *b* = *Otus sunia*, on a long branch; no reference sequences of *Glaucidium brodiei* available

Sequence integrity:

Cyt *b*: multiple unique substitutions at posituions 384-704. These did not match any known species.

Interpretation: misidentification, with additional sequencing errors / numts. This sequence turning up as sister to *G. cuculoides* (with high support) in Liu et al. (2019a) is almost certainly fraudulent; the phylogeny is presumably hand-drawn to reflect relationships found in previous studies, rather than based on the results of Lui et al.’s own data. (see also *Caprimulgus indicus* in Liu et al. (2017a, 2019a) for a similar problem.)

Subsequent usage: not cited or used in other papers (January 2020).

Problems noted in other works: None.

Relevance: A previous mitogenome of this species (KP684122) was published by Sun et al. (2016), which was not cited by Liu et al. (2019a).

References:

Liu, G, Li, C, Du, Y & Liu, X 2017. The complete mitochondrial genome of Japanese sparrowhawk (*Accipiter gularis*) and the phylogenetic relationships among some predatory birds. Biochemical Systematics and Ecology 70: 116-125.

Liu, G, Zhou, L & Zhao, G 2019a. Complete mitochondrial genomes of five raptors and implications for the phylogenetic relationships between owls and nightjars. PeerJ Preprints, e27478v1.

Sun, X, Zhou, W, Sun, Z, Qian, L, Zhang, Y, Pan, T, & Zhang, B 2016e. The complete mitochondrial genome of *Glaucidium brodiei* (Strigiformes: Strigidae). Mitochondrial DNA Part A 27: 2508-2509.

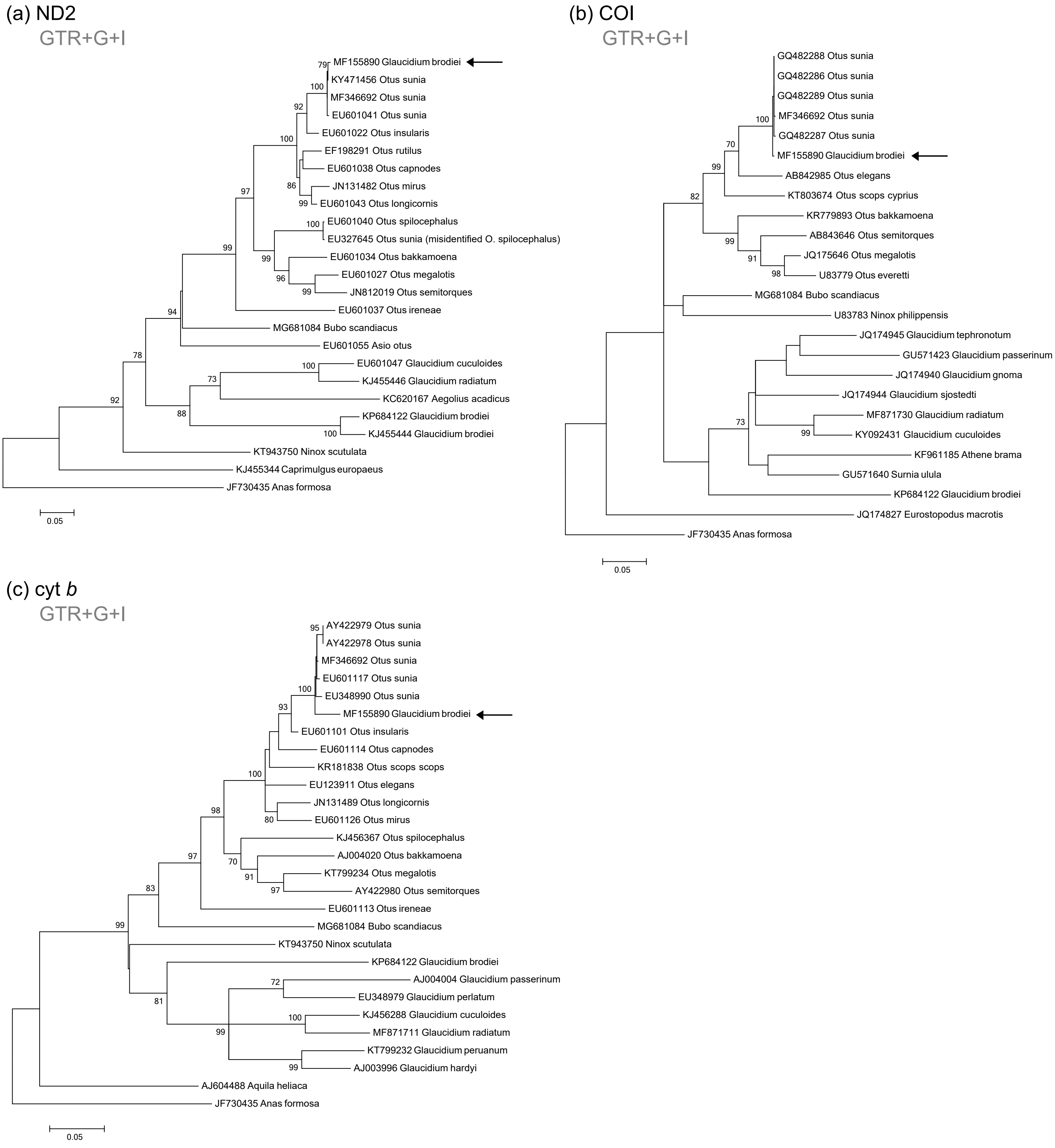

Figure S30. Maximum Likelihood phylogenies of *Glaucidium brodiei* (MF155890) and related taxa based on mitochondrial sequences. Numbers at branches are bootstrap support values (>70%) based on 1000 replicates.

39. "*Ninox strenua*" KX529654, NC_033967 (Sarker et al. 2016)

Fig. S31

Metadata: "The blood sample was obtained from a powerful owl in the wild (year of sampling: 2015; GPS location: 34º00'22.9''S, 151º02'18.6''E). The sample was immediately transferred to the Veterinary Diagnostic Laboratory (VDL), Charles Sturt University, and stored under the accession number CS15-3907." (Sarker et al. 2016: 722)

Phylogenetic position:

ND2 = *Ninox strenua* but on a long branch

CO1 = could not be verified; no sequences of *Ninox strenua* available

Cyt *b* = *Ninox strenua* but on a long branch

Sequence integrity:

ND2: 13 singleton sites (substitutions not found among two other ND2 sequences of *Ninox strenua*); of these, 7 were not found among 52 other ND2 sequences of *Ninox* owls.

cyt b: 16 singleton sites (substitutions not found among 7 other cyt *b* sequences of *Ninox* owls, including one *Ninox strenua*, EU348984).

Interpretation: sequencing errors / numts

Subsequent usage:

Re-used in **6 mitogenomic phylogenies published before 1 January 2020**: Kang et al. 2018, Lee et al. 2018, Liu et al. 2019a, Park et al. 2019a, Park et al. 2019b, Zhou et al 2019a.

Problems noted in other works: None.

Relevance: This was the only mitogenome presumed to be of this species (January 2020).

References:

Kang, H, Li, B, Ma, X & Xu, Y 2018. Evolutionary progression of mitochondrial gene rearrangements and phylogenetic relationships in Strigidae (Strigiformes). Gene 674: 8-14.

Lee, MY, Lee, SM, Jeon, HS, Lee, SH, Park, JY & An, J 2018. Complete mitochondrial genome of the Northern Long-eared Owl (*Asio otus* Linnaeus, 1758) determined using next-generation sequencing. Mitochondrial DNA Part B 3: 494-495.

Liu, G, Zhou, L & Zhao, G 2019a. Complete mitochondrial genomes of five raptors and implications for the phylogenetic relationships between owls and nightjars. PeerJ Preprints, e27478v1.

Park, CE, Kim, MC, Ibal, JCP, Pham, HQ, Park, HC & Shin, JH 2019a. The complete mitochondrial genome sequence of *Otus scops* (Aves, Strigiformes, Strigidae). Mitochondrial DNA Part B 4: 764-765.

Park, CE, Kim, MC, Ibal, JCP, Pham, HQ, Park, HC & Shin, JH 2019b. The complete mitochondrial genome sequence of *Otus bakkamoena* (Aves, Strigiformes, Strigidae). Mitochondrial DNA Part B 4: 775-776.

Sarker, S, Das, S, Forwood, J, Helbig, K & Raidal, SR 2016. The complete mitochondrial genome sequence of an Endangered powerful owl (*Ninox strenua*). Mitochondrial DNA Part B 1: 722-723.

Zhou, C, Chen, Y, Hao, Y, Meng, Y, Yue, B & Zeng, T 2019a. Characterization of the complete mitochondrial genome and phylogenetic analysis of *Otus sunia* (Strigiformes: Strigidae). Mitochondrial DNA Part B 4: 804-805.

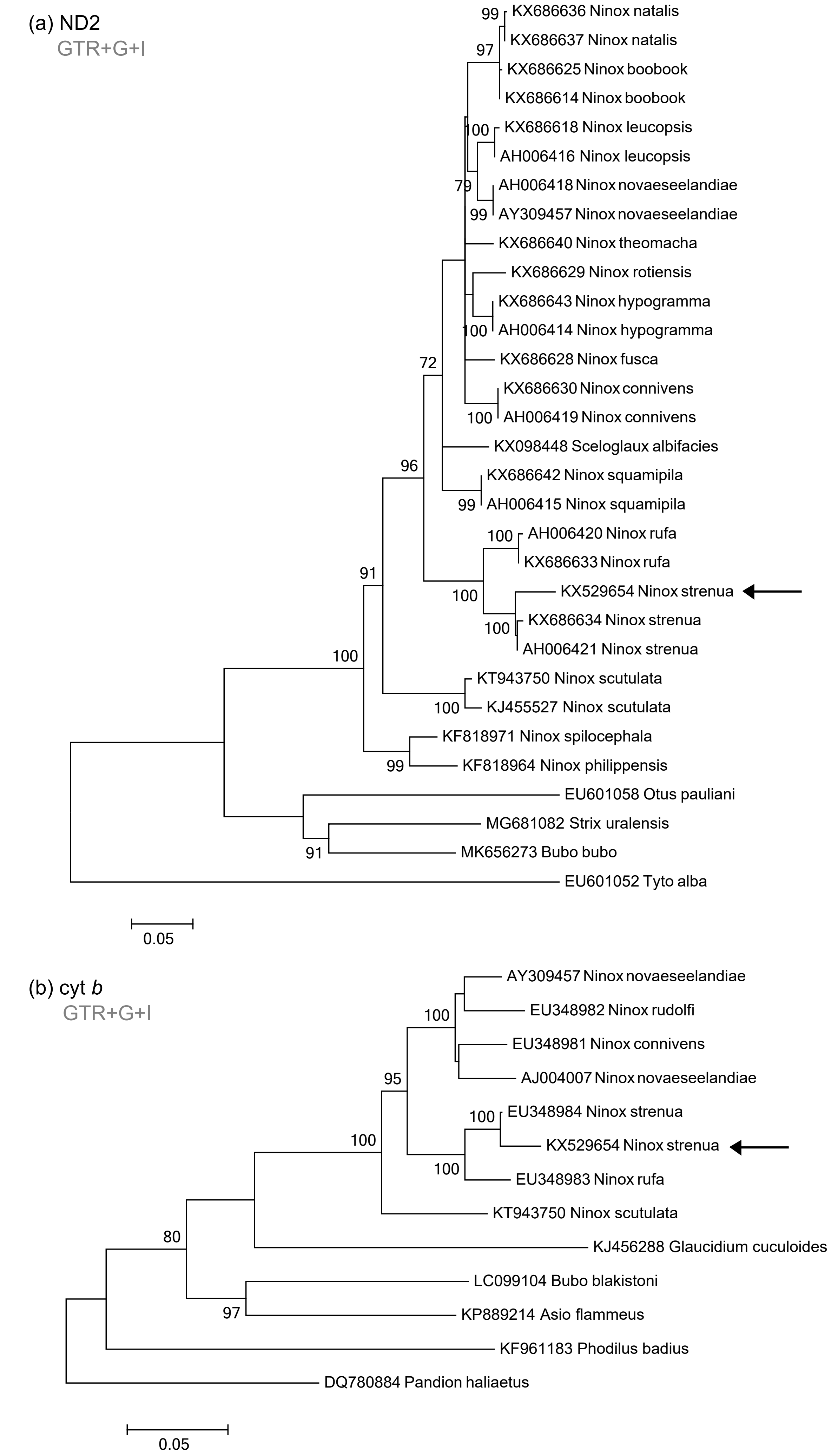

Figure S31. Maximum Likelihood phylogenies of *Ninox strenua* (KX529654) and related taxa based on mitochondrial sequences. Numbers at branches are bootstrap support values (>70%) based on 1000 replicates.

40. “*Trogon viridis*” EU410490, NC_011714 (Pratt et al. 2009)

Fig. S32

Metadata: The … white-tailed trogon (*T. viridis*) [was] provided by the Louisiana State University Museum of Natural Science Collection of Genetic Resources under sample [number] LSUMZ B-28495.

Phylogenetic position:

ND2 = *Trogon chionurus*

CO1 = sister to, but divergent from, *Trogon chionurus*

Cyt *b* = *Trogon chionurus*

Sequence integrity: not investigated

Interpretation: Misidentification due to outdated taxonomy. *T. chionurus* was formerly included in *T. viridis* but was treated as a separate species (Chesser et al. 2010) based on molecular differences (DaCosta & Klicka 2008).

Subsequent usage:

Re-used in **7 mitogenomic phylogenies published before 1 January 2020**: Pacheco et al. 2011, Fuchs et al. 2015, Nabholz et al. 2016, Eo 2017, Sun et al. 2017c, Duan et al. 2018b, Tamashiro et al. 2019.

Problems noted in other works: none.

Relevance: This was the only mitogenome presumed to be of this species (January 2020).

References:

Chesser, RT, Banks, RC, Barker, FK, Cicero C, Dunn, JL, Kratter, AW, Lovette, IJ, Rasmussen, PC, Remsen, JV, Rising, JD, Stotz, DF & Winker, K 2010. Fifty-first supplement to the American Ornithologists' Union: Check-list of North American Birds. Auk 127: 726–744.

Dacosta, JM & Klicka, J 2008. The Great American Interchange in birds: a phylogenetic perspective with the genus *Trogon*. Mol. Ecol. 17: 1328-1343.

Duan, Y, Li, Y, Liang, D, Shao, S & Luo, X 2018b. Complete mitochondrial genome of yellow-rumped honeyguide *Indicator xanthonotus* (Piciformes: Indicatoridae). Mitochondrial DNA Part B 3: 1278-1279.

Eo, SH 2017. Complete mitochondrial genome of white-backed woodpecker *Dendrocopos leucotos* (Piciformes: Picidae) and its phylogenetic position. Mitochondrial DNA Part B 2: 451-452.

Fuchs, J, Pons, JM, Pasquet, E & Bonillo, C 2015. Complete mitochondrial genomes of the white-browed piculet (*Sasia ochracea*, Picidae) and pale-billed woodpecker (*Campephilus guatemalensis*, Picidae). Mitochondrial DNA Part A 27: 3640-3641.

Nabholz, B, Lanfear, R & Fuchs, J 2016. Body mass-corrected molecular rate for bird mitochondrial DNA. Mol. Ecol. 25: 4438-4449.

Pacheco, MA, Battistuzzi, FU, Lentino, M, Aguilar, R, Kumar, S & Escalante, AA 2011. Evolution of modern birds revealed by mitogenomics: timing the radiation and origin of major orders. Mol. Biol. Evol. 28: 1927–1942.

Pratt, RC, Gibb, GC, Morgan-Richards, M, Phillips, MJ, Hendy, MD & Penny, D 2009. Towards resolving deep Neoaves phylogeny: data, signal enhancement and priors. Mol. Biol. Evol. 26: 313-326.

Sun, X, Zhao, R, Zhang, T, Gong, J, Jing, M & Huang, L 2017c. Two mitochondrial genomes in Alcedinidae (*Ceryle rudis/Halcyon pileata*) and the phylogenetic placement of Coraciiformes. Genetica 145: 431-440.

Tamashiro, RA, White, ND, Braun, MJ, Faircloth, BC, Braun, EL & Kimball, RT 2019. What are the roles of taxon sampling and model fit in tests of cyto-nuclear discordance using avian mitogenomic data? Molecular Phylogenetics and Evolution 130: 132-142.

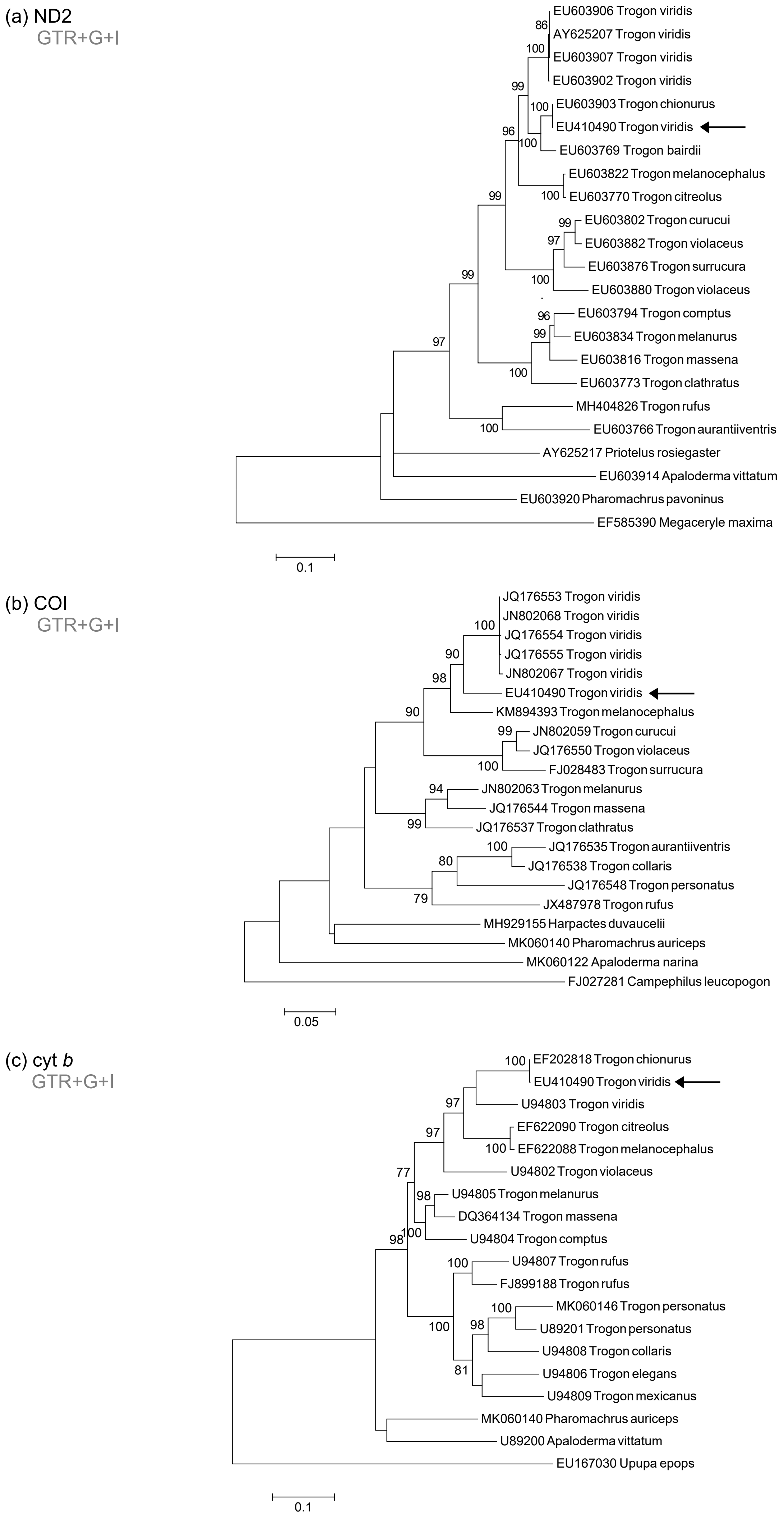

Figure S32. Maximum Likelihood phylogenies of *Trogon viridis* (EU410490) and related taxa based on mitochondrial sequences. Numbers at branches are bootstrap support values (>70%) based on 1000 replicates.

41. “*Anthracoceros coronatus*” MF435900, NC_038152 (Li et al. 2018)

Fig. S33

Metadata: “The sample was collected on occasion from Yunnan Province, southwestern China.” (Li et al. 2018: 401).
Phylogenetic position:
ND2 = identical to *Anthracoceros albirostris*, but no sequences of *Anthracoceros coronatus* available
CO1 = could not be verified; no sequences of *Anthracoceros coronatus* available
Cyt *b* = *Anthracoceros albirostris*
Sequence integrity: not investigated
Interpretation: misidentification due to outdated taxonomy. *Anthracoceros albirostris* and *A. coronatus* were formerly considered conspecific (under the name *A. coronatus*), but have been treated as species since 1983 (Frith & Frith 1983, Sibley & Monroe 1990).
Subsequent usage:
Cited by **1 paper**: Chen et al. 2019b.
Although *albirostris* was previously considerd a subspecies of *Anthracoceros coronatus*, Li et al. (2018) specifically discussed the taxon *coronatus* in a strict sense, i.e. Malabar Pied Hornbill (which excludes the taxon *albirostris*). “*Anthracoceros coronatus*, commonly called as Malabar Pied Hornbill” (Li et al. 2018: 401). “This species distributes mainly in tropical deciduous forests of central and southern India and Sri Lanka.” “*Anthracoceros coronatus* (Bucerotiformes: Bucerotidae) has been listed in near threatened category in the IUCN Red List of Threatened Species, due to population decline.” (Li et al. 2018: 401). *Anthracoceros albirostris* is Least Concern and stable.
Problems noted in other works: None.
Relevance: This was the only mitogenome presumed to be of this species (January 2020).
References:

Chen, Y, Yan, H, Sun, J, Li, C, Xiao, H & Chen, S 2019b. Characterization and phylogenetic analysis of the complete mitochondrial genome sequence of *Rhyticeros undulatus* (Bucerotiformes: Bucerotidae). Conservation Genetics Resources 11: 27-30.

Frith, CB & Frith, DW 1983. A systematic review of the hornbill genus *Anthracoceros* (Aves, Bucerotidae). Zoological Journal of the Linnean Society 78: 29-71.

Li, R, Chen, Y, Yan, H, Li, C, Xiao, H & Chen, S 2018. The complete mitochondrial genome sequence of *Anthracoceros coronatus* (Bucerotiformes: Bucerotidae). Conservation Genetics Resources 10: 401-404.

Sibley, CG & Monroe, BL 1990*. Distribution and taxonomy of birds of the world. Yale Univ. Press, New Haven.

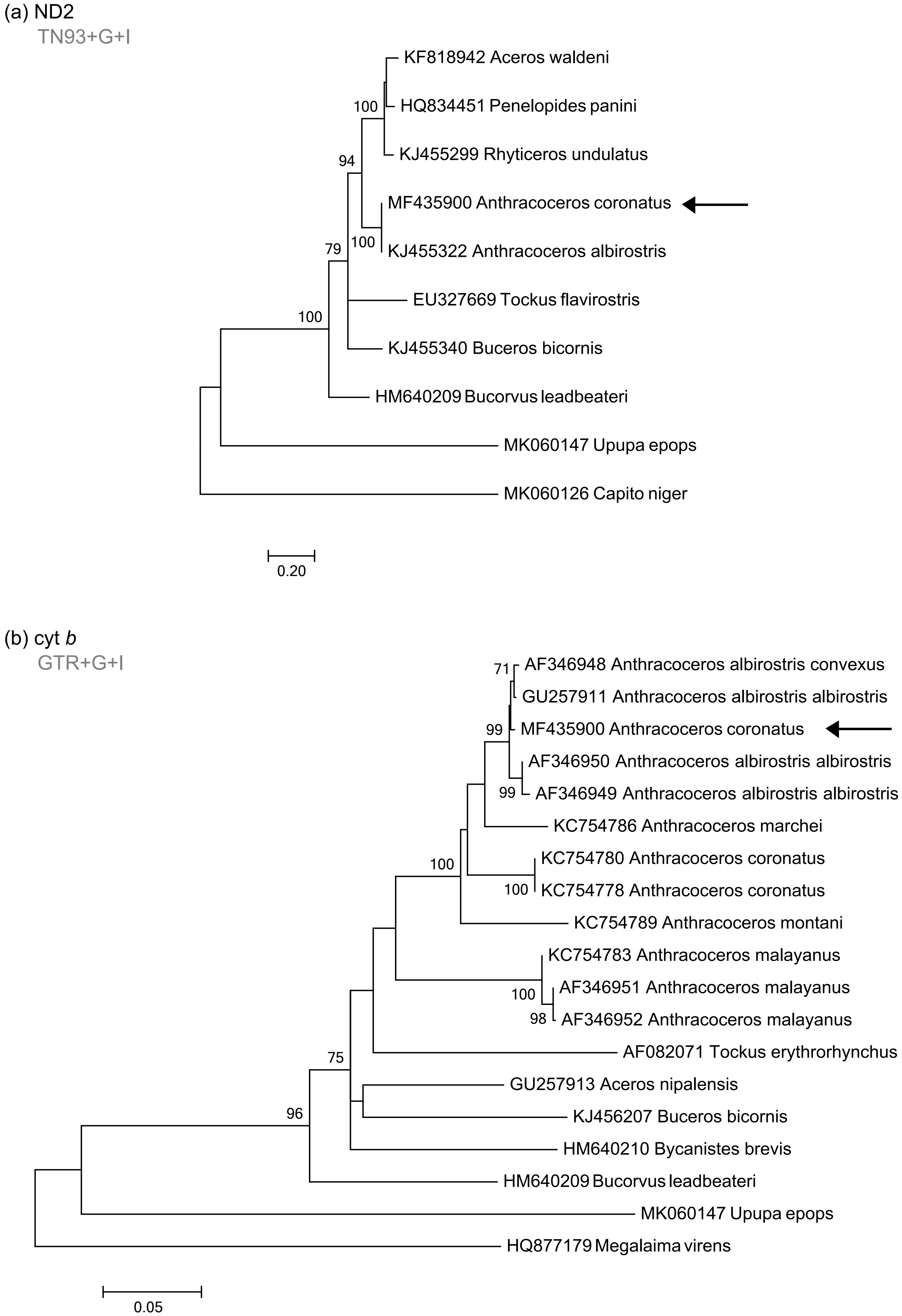

Figure S33. Maximum Likelihood phylogenies of *Anthracoceros coronatus* (MF435900) and related taxa based on mitochondrial sequences. Numbers at branches are bootstrap support values (>70%) based on 1000 replicates.

42. "*Alcedo atthis*" KY964271, NC_035868 (Jing et al. 2020)

Fig. S34

Metadata: "The samples of *Megaceryle lugubris*, *Alcedo atthis* and *Halcyon smyrnensis* were collected from Nantong national airport, Jiangsu Province, China. The identification of the specimens was according to external morphologies (Sibley and Monroe, 1990)." (Jing et al. 2020: 2).
Phylogenetic position:
ND2 = *Alcedo atthis*
CO1 = sister to all *Alcedo atthis* and on a long branch
Cyt *b* = *Alcedo atthis*
Sequence integrity: COI: pos 1-393 0-0.8% divergence with other *Alcedo atthis*; pos 394-694 BLAST 100% match with *Gallinula chloropus*.
Interpretation: chimera
Subsequent usage:
Re-used in **1 mitogenomic phylogeny published before 1 January 2020**: Duan et al. 2018.
Problems noted in other works: None.
Relevance: This was the only mitogenome presumed to be of this species (January 2020)
References:

Duan, Y, Li, Y, Liang, D, Shao, S & Luo, X 2018b. Complete mitochondrial genome of yellow-rumped honeyguide *Indicator xanthonotus* (Piciformes: Indicatoridae). Mitochondrial DNA Part B 3: 1278-1279.

Jing, M, Yang, H, Li, K & Huang, L 2020. Characterization of three new mitochondrial genomes of Coraciiformes (*Megaceryle lugubris*, *Alcedo atthis*, *Halcyon smyrnensis*) and insights into their phylogenetics. Genetics and Molecular Biology 43: 4, e20190392.

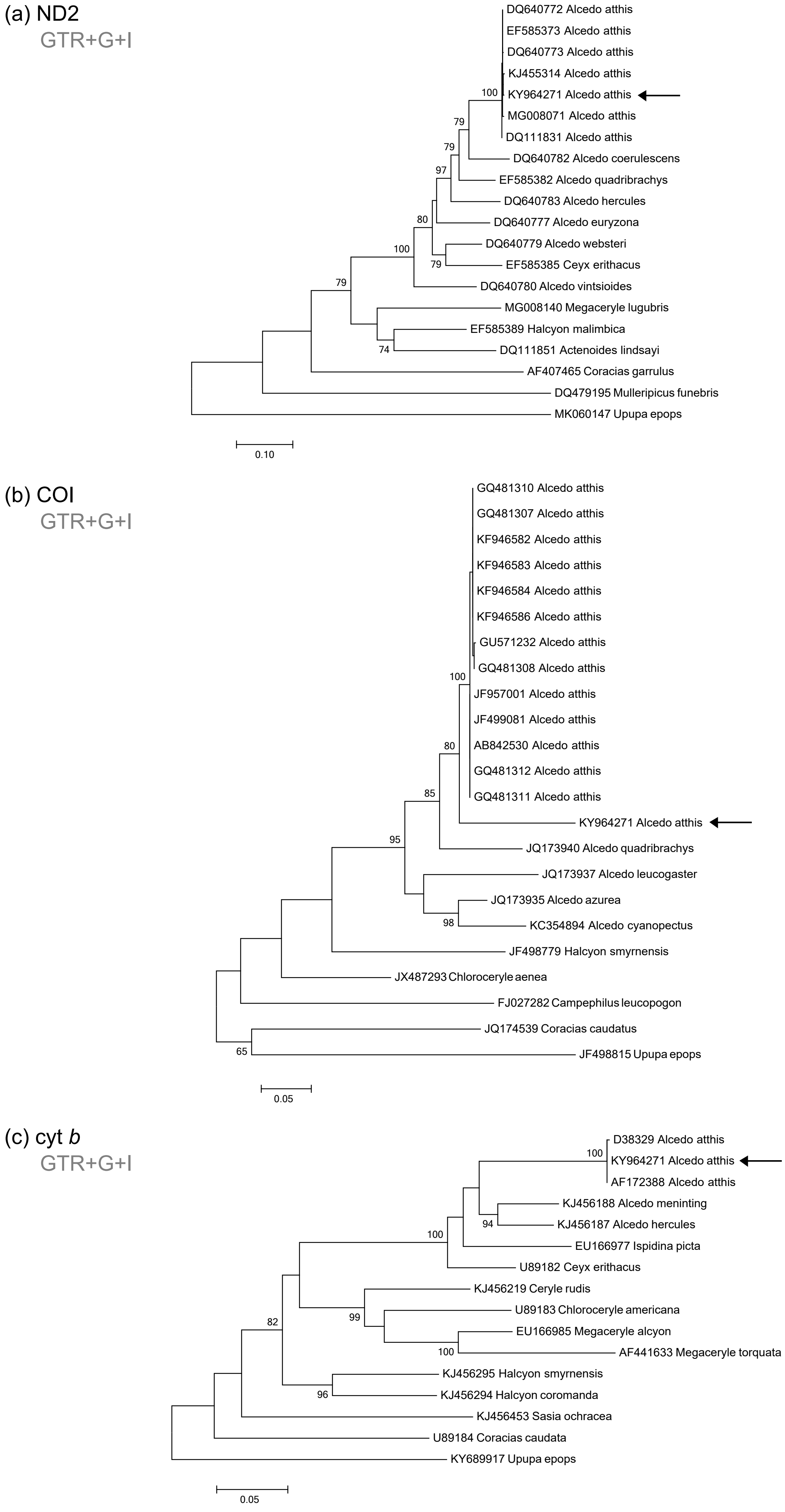

Figure S34. Maximum Likelihood phylogenies of *Alcedo atthis* (KY964271) and related taxa based on mitochondrial sequences. Numbers at branches are bootstrap support values (>70%) based on 1000 replicates.

43. “*Megaceryle (Ceryle) lugubris*” KY940558, NC_035658 (Jing et al. 2020)

Fig. S35

Metadata: “The samples of *Megaceryle lugubris*, *Alcedo atthis* and *Halcyon smyrnensis* were collected from Nantong national airport, Jiangsu Province, China. The identification of the specimens was according to external morphologies (Sibley and Monroe, 1990).” (Jing et al. 2020: 2). Note that Sibley & Monroe (1990) did not include any morphological characters or diagnosis.

Phylogenetic position:

ND2 = sister to four *Ceryle rudis*, but with deep divergence

CO1 = did not cluster with three *M. lugubris* but no sequence of *Ceryle rudis* available

Cyt *b* = clustered with *Ceryle rudis* but on a long branch; no sequence of *Megaceryle lugubris* available

Sequence integrity: ND2: partitions of 120 bp (i.e. bp 1-120, 121-240 etc) were examined with BLAST. Almost all fragments matched best with *Ceryle rudis*, but still rather poorly (92-96%, or less). No closer matches with any other species were found.

Interpretation: misidentification, with additional sequencing errors / numts

Subsequent usage:

Re-used in **1 mitogenomic phylogeny published before 1 January 2020**: Duan et al. 2018b.

Problems noted in other works: None.

Relevance: This was the only mitogenome presumed to be of this species (January 2020).

References:

Duan, Y, Li, Y, Liang, D, Shao, S & Luo, X 2018b. Complete mitochondrial genome of yellow-rumped honeyguide *Indicator xanthonotus* (Piciformes: Indicatoridae). Mitochondrial DNA Part B 3: 1278-1279.

Jing, M, Yang, H, Li, K & Huang, L 2020. Characterization of three new mitochondrial genomes of Coraciiformes (*Megaceryle lugubris*, *Alcedo atthis*, *Halcyon smyrnensis*) and insights into their phylogenetics. Genetics and Molecular Biology 43: 4, e20190392.

44. “*Ceryle rudis*” KJ461938, NC_024280 (Sun et al. 2017c)

Fig. S35

Metadata: “collected from Wuyi Mountain, Fujian Province, China” (Sun et al. 2017c: 432)
Phylogenetic position:
ND2 = sister to three *Ceryle rudis* but on a long branch
CO1 = nearly identical to three *Megaceryle lugubris*; no sequence of *Ceryle rudis* available
Cyt *b* = did not cluster with *Ceryle rudis* but no sequence of *Megaceryle lugubris* available
Sequence integrity:
ND2: bp 1–525 showed 99-100% similarity to *Megaceryle lugubris* (n=2); bp 537–1041 showed 99–100% similarity to *Ceryle rudis* (n=3).
Interpretation: chimera
Subsequent usage:
Re-used in **5 mitogenomic phylogenies published before 1 January 2020**: Nabholz et al. 2016, Eo 2017, Duan et al. 2018b, Tamashiro et al. 2019, Liu et al. 2019a,
Problems noted in other works: None.
Relevance: This was the only mitogenome presumed to be of this species (January 2020)
References:

Duan, Y, Li, Y, Liang, D, Shao, S & Luo, X 2018b. Complete mitochondrial genome of yellow-rumped honeyguide *Indicator xanthonotus* (Piciformes: Indicatoridae). Mitochondrial DNA Part B 3: 1278-1279.

Eo, SH 2017. Complete mitochondrial genome of white-backed woodpecker *Dendrocopos leucotos* (Piciformes: Picidae) and its phylogenetic position. Mitochondrial DNA Part B 2: 451-452.

Liu, G, Zhou, L & Zhao, G 2019a. Complete mitochondrial genomes of five raptors and implications for the phylogenetic relationships between owls and nightjars. PeerJ Preprints, e27478v1.

Nabholz, B, Lanfear, R & Fuchs, J 2016. Body mass-corrected molecular rate for bird mitochondrial DNA. Mol. Ecol. 25: 4438-4449.

Sun, X, Zhao, R, Zhang, T, Gong, J, Jing, M & Huang, L 2017c. Two mitochondrial genomes in Alcedinidae (*Ceryle rudis/Halcyon pileata*) and the phylogenetic placement of Coraciiformes. Genetica 145: 431-440.

Tamashiro, RA, White, ND, Braun, MJ, Faircloth, BC, Braun, EL & Kimball, RT 2019. What are the roles of taxon sampling and model fit in tests of cyto-nuclear discordance using avian mitogenomic data? Molecular Phylogenetics and Evolution 130: 132-142.

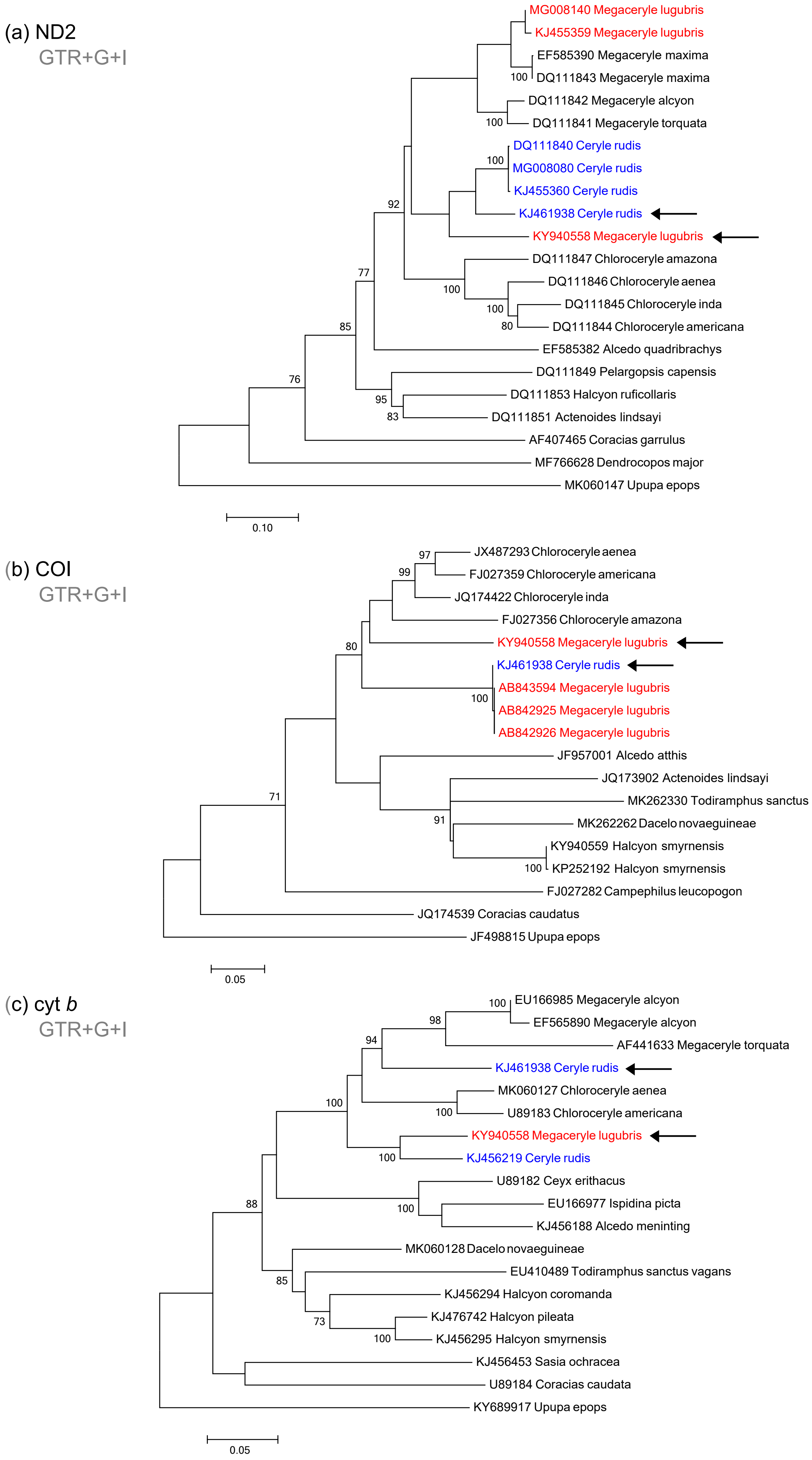

Figure S35. Maximum Likelihood phylogenies of *Megaceryle lugubris* (KY940558), *Ceryle rudis* (KJ461938) and related taxa based on mitochondrial sequences. Numbers at branches are bootstrap support values (>70%) based on 1000 replicates.

45. “*Yungipicus* (*Dendrocopos*) *canicapillus*” MK335534 (Bi et al. 2019)

Fig. S36

Metadata: “Two frozen muscle tissue samples *D. darjellensis* (code AHNUK0328) and *Y. canicapillus* (code AHNU-K0340) were chosen for this study. These frozen samples were provided by the Ningguo Museum of Natural History, Anhui Province, China.” (Bi et al. 2019: 685)
Phylogenetic position:
ND2 = *Yungipicus canicapillus*
CO1 = *Yungipicus canicapillus*
Cyt *b* = sister to, and divergent from, two *Y. canicapillus* and one *Picoides moluccensis*.
Sequence integrity: cyt *b*: bp 687-1143 are 99.1% *Dendrocopos darjellensis*. A mitogenome of this species was published in the same paper (Bi et al. 2019).
Interpretation: chimera
Subsequent usage: not used in subsequent works (January 2020)
Problems noted in other works: None.
Relevance: One other mitogenome of *Y. canicapillus* is available (MK015644, Lai et al. 2019).
References:
Bi, D, Ding, H, Wang, Q, Jiang, L, Lu, W, Wu, X, Zhu, R, Zeng, J, Zhou, S, Yang, X & Kan, X 2019. Two new mitogenomes of Picidae (Aves, Piciformes): sequence, structure and phylogenetic analyses. International Journal of Biological Macromolecules 133: 683-692.

Lai, WN, Yan, SQ, Jiao, SY, Yao, JY & Li, YM 2019*. Complete mitochondrial genome of *Dendrocopos canicapillus* (Piciformes: Picidae). Mitochondrial DNA Part B 4: 141-142.

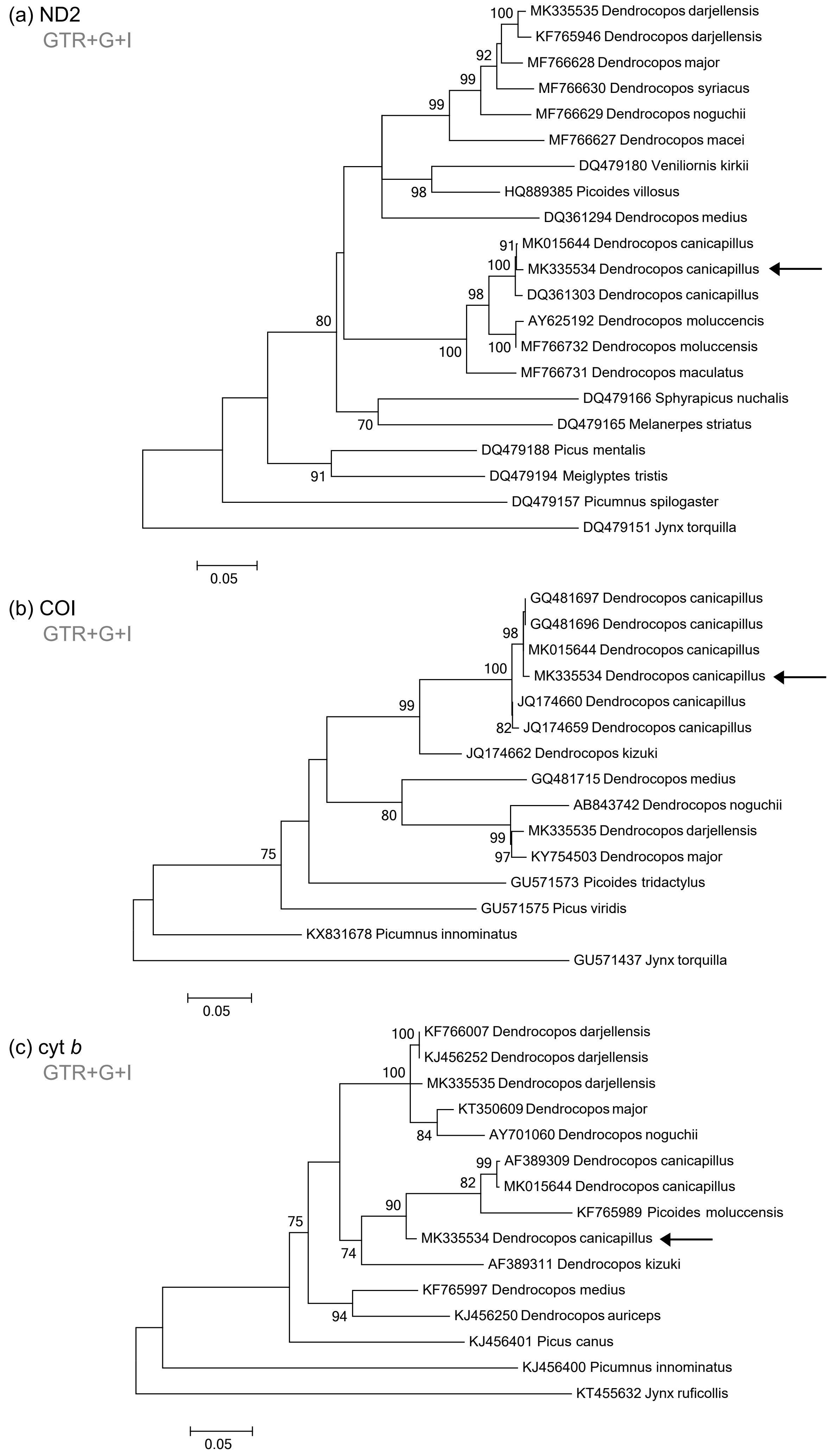

Figure S36. Maximum Likelihood phylogenies of *Dendrocopos* (*Yungipicus*) *canicapillus* (KY940558), and related taxa based on mitochondrial sequences. Numbers at branches are bootstrap support values (>70%) based on 1000 replicates.

46. "*Falco naumanni*" KM251414, NC_029846 (Wang et al. 2016)

Fig. S37

Metadata: no data in Wang et al. (2016); GenBank: "country = China: Yunnan"
Phylogenetic position:
ND2 = *Falco tinnunculus*
CO1 = *Falco tinnunculus*
Cyt *b* = *Falco tinnunculus*
Sequence integrity: not investigated
Interpretation: misidentification
Subsequent usage:
Re-used in **7 mitogenomic phylogenies published before 1 January 2020**: Liu et al. 2017a, Doyle et al. 2018, Yang et al. 2018, Liu et al. 2019a, Jiang et al. 2019b, Zhang et al. 2019b, Mackiewicz et al. 2019.
Problems noted in other works: None.
Relevance: This was the only mitogenome presumed to be of this species (January 2020). A sequence of *Falco tinnunculus* (EU196361) has been available since 2007.
References:

Doyle, JM, Bell, DA, Bloom, PH, Emmons, G, Fesnock, A, Katzner, TE, LaPré, L, Leonard, K, SanMiguel, P, Westerman, R & Andrew DeWoody, J 2018. New insights into the phylogenetics and population structure of the prairie falcon (*Falco mexicanus*). BMC Genomics 19:233.

Jiang, L, Peng, L, Tang, M, You, Z, Zhang, M, West, A, Ruan, G, Chen, W & Merilä, J 2019b. Complete mitochondrial genome sequence of the Himalayan Griffon, *Gyps himalayensis* (Accipitriformes: Accipitridae): Sequence, structure, and phylogenetic analyses. Ecology and Evolution 9: 8813–8828.

Liu, G, Li, C, Du, Y & Liu, X 2017a. The complete mitochondrial genome of Japanese sparrowhawk (*Accipiter gularis*) and the phylogenetic relationships among some predatory birds. Biochemical Systematics and Ecology 70: 116-125.

Liu, G, Zhou, L & Zhao, G 2019a. Complete mitochondrial genomes of five raptors and implications for the phylogenetic relationships between owls and nightjars. PeerJ Preprints, e27478v1.

Mackiewicz, P, Urantówka, AD, Kroczak, A & Mackiewicz, D 2019. Resolving phylogenetic relationships within Passeriformes based on mitochondrial genes and inferring the evolution of their mitogenomes in terms of duplications. Genome Biology and Evolution, 11: 2824-2849.

Wang, HW, Zhang, HF, Ren, L, Xu, Y, Zeng, YJ, Miao, YL, Luo, HY & Wang, KH 2016c. The whole mitochondrial genome of the Lesser Kestrel (*Falco naumanni*). Mitochondrial DNA Part A 27: 2385-2386.

Yang, C, Yang, M, Wang, Q, Lu, Y & Li, X 2018. The complete mitogenome of *Falco amurensis* (Falconiformes, Falconidae), and a comparative analysis of genus *Falco*. Zoological Science 35: 367-373.

Zhang, F, Zhou, L, Dong, Y & Song, Y 2019. Complete mitochondrial genome of *Accipiter trivirgatus*. Mitochondrial DNA Part B 4: 3652-3653.

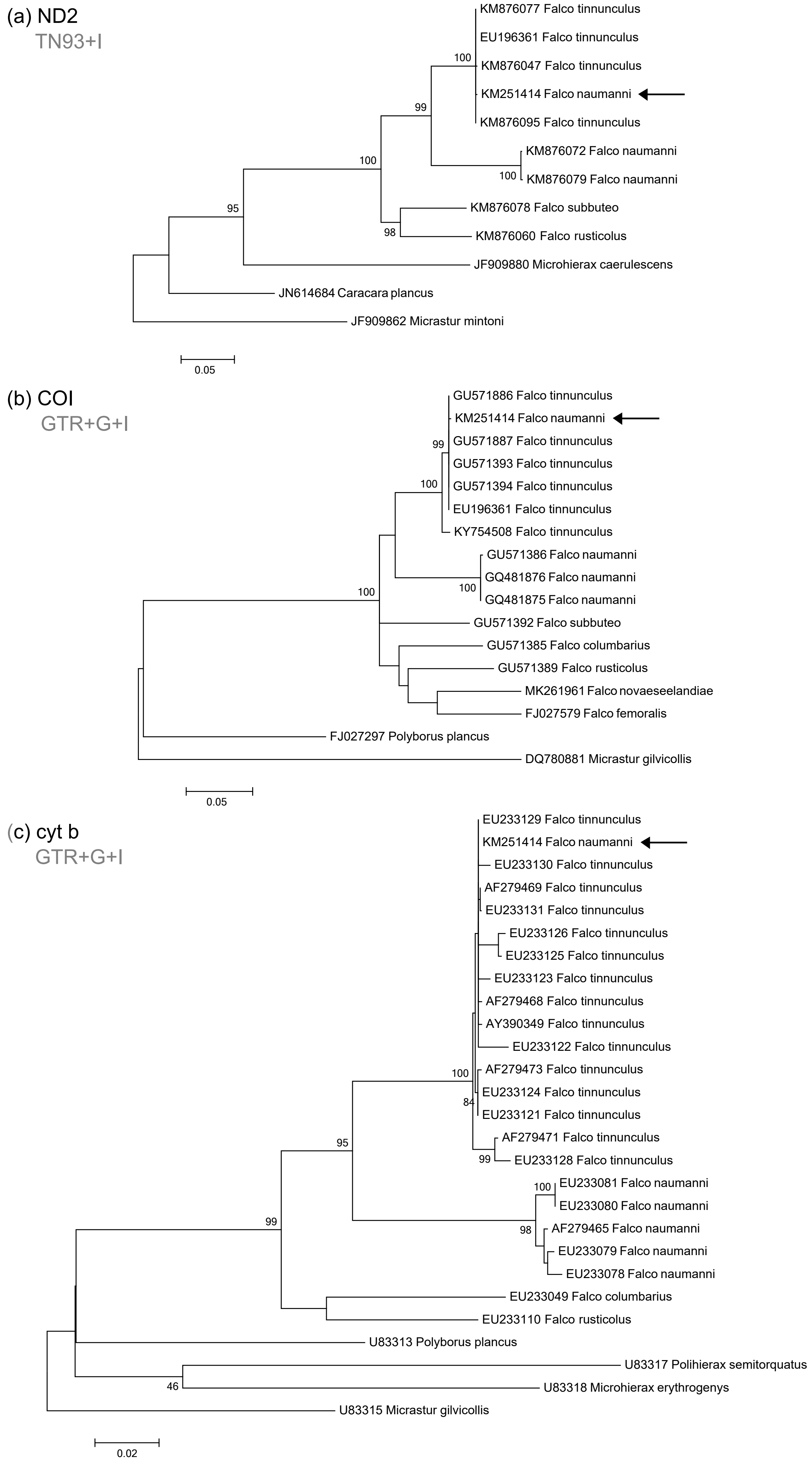

Figure S37. Maximum Likelihood phylogenies of *Falco naumanni* (KM251414), and related taxa based on mitochondrial sequences. Numbers at branches are bootstrap support values (>70%) based on 1000 replicates.

47. "*Falco mexicanus*" (Doyle et al. 2018)

Fig. S38

Note: this sequence is not available on GenBank but can be accessed at the Dryad digital repository (https://doi.org/10.5061/dryad.8b0s04t).

Metadata: "A female prairie falcon was captured in Siskiyou County, California on 7 June 2014. Two drops of blood were collected ..." (Doyle et al. 2018)

Phylogenetic position:

ND2 = sister to two *Falco mexicanus* but on a long branch

CO1 = sister to a clade formed by *Falco mexicanus, F. rusticolus/cherrug/biarmicus* and *F. peregrinus*

Cyt *b* = *Falco mexicanus*

Sequence integrity:

ND2: 6.0% and 6.1% divergent from two *F. mexicanus.* No less than 39 substitutions were unique among large falcons, i.e. were not found in *Falco mexicanus* (n=2)*, F. rusticolus* (n=4), *F. cherrug* (n=3), *F. biarmicus* (n=1), *F. jugger* (n=1), *F. fasciinucha* (n=1), and *F. peregrinus* (n=12), except for one highly divergent *F. peregrinus* sequence (EU327624) that was previously flagged by Nacer & do Amaral (2017) a sequence consisting of numts.

COI: 5.0% and 5.1% divergent from two *F. mexicanus.* No less than 26 substitutions were unique among large falcons, i.e. were not found in *Falco mexicanus* (n=2)*, F. rusticolus* (n=8), *F. cherrug* (n=1), *F. biarmicus* (n=1) and *F. peregrinus* (n=20).

Interpretation: sequencing errors / numts. Numts are common in falcons (Nacer & do Amaral 2017).

Subsequent usage: The sequence has not been used in any further studies (January 2020)

Problems noted in other works: None.

Relevance: This was the only mitogenome presumed to be of this species (January 2020).

References:

Doyle, JM, Bell, DA, Bloom, PH, Emmons, G, Fesnock, A, Katzner, TE, LaPré, L, Leonard, K, SanMiguel, P, Westerman, R & Andrew DeWoody, J 2018. New insights into the phylogenetics and population structure of the prairie falcon (*Falco mexicanus*). BMC Genomics 19, 233.

Nacer, DF & do Amaral, FR 2017. Striking pseudogenization in avian phylogenetics: numts are large and common in falcons. Molecular Phylogenetics and Evolution 115: 1-6.

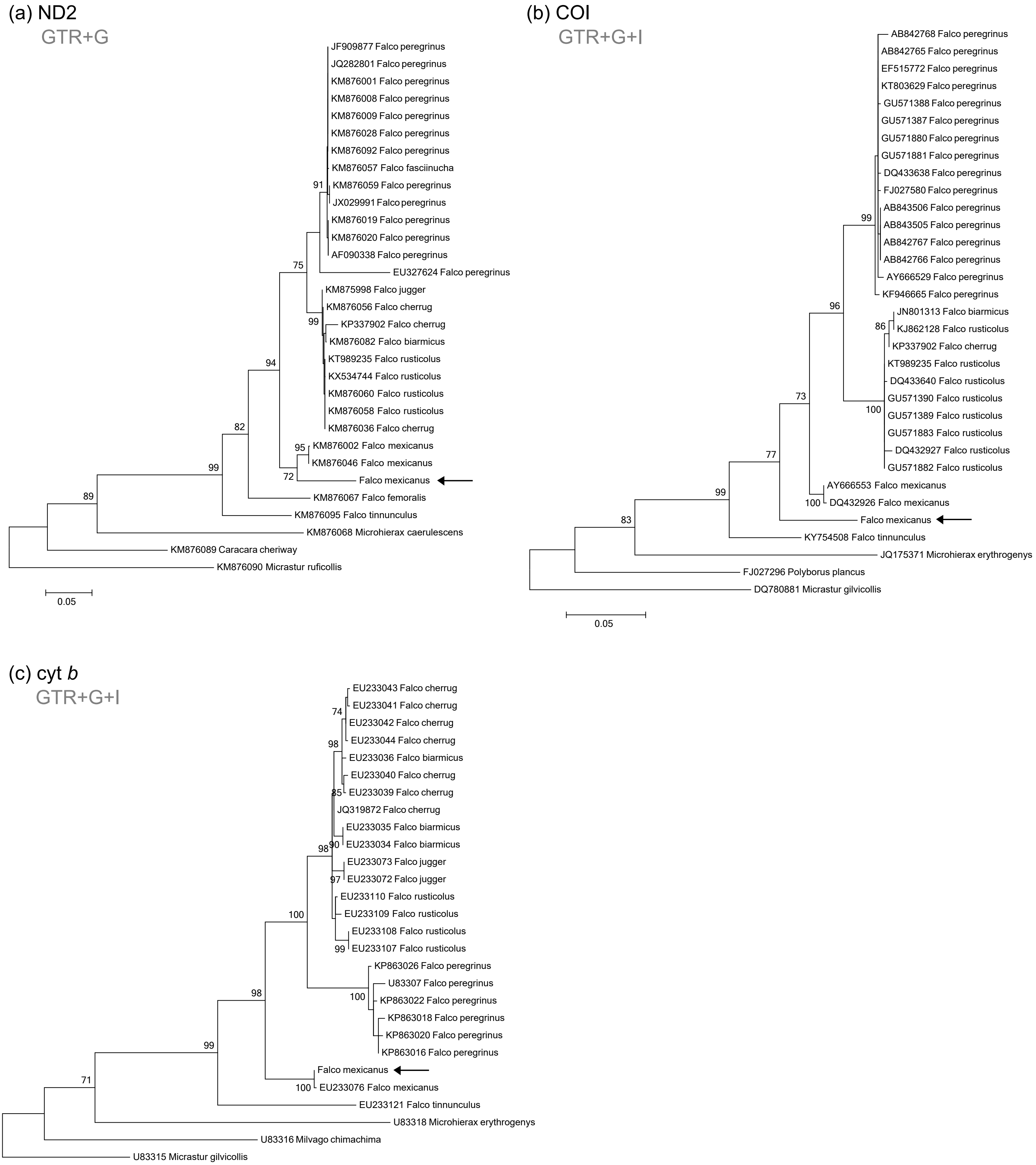

Figure S38. Maximum Likelihood phylogenies of *Falco mexicanus* (Doyle et al. 2018), and related taxa based on mitochondrial sequences. Numbers at branches are bootstrap support values (>70%) based on 1000 replicates.

48. “*Cacatua sanguinea*” MN126573 (Sarker et al. 2019c)

Fig. S39

Metadata: “A swab sample moistened in sterile phosphate-buffered saline, of the choana and cloaca was taken from a freeranging Little Corella (*C. sanguinea*) originating from Nunawading, Victoria (Sample ID: #46, GPS location: Latitude: 37º49’2.46’’S, Longitude: 145º10’37.2’’E) and was used in this study.” (Sarker et al. 2019c: 3792)

Phylogenetic position:

ND2 = *Psittacula krameri*

CO1 = *Psittacula krameri*

Cyt *b* = *Psittacula krameri*

Sequence integrity: not investigated

Interpretation: misidentification

Subsequent usage: not subsequently used (January 2020)

Problems noted in other works: None.

Relevance: This was the only mitogenome presumed to be of this species (January 2020).

References:

Sarker, S, Talukder, S, Sutherland, M, Forwood, JK, Helbig, K & Raidal, SR 2019c. Characterization of the first mitochondrial genome of a little Corella (*Cacatua sanguinea*) and its phylogenetic implications. Mitochondrial DNA Part B 4: 3792-3794.

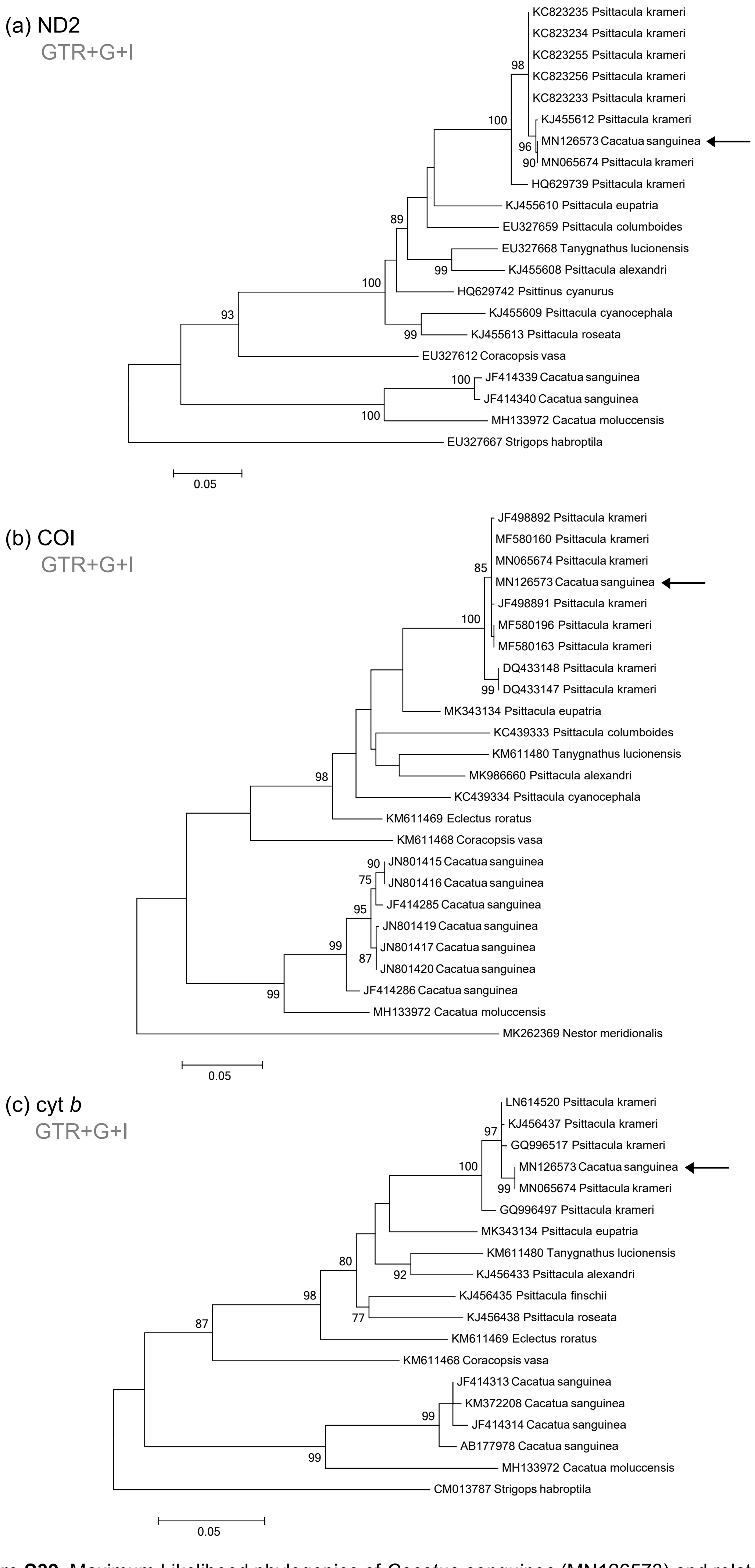

Figure S39. Maximum Likelihood phylogenies of *Cacatua sanguinea* (MN126573) and related taxa based on mitochondrial sequences. Numbers at branches are bootstrap support values (>70%) based on 1000 replicates.

49. “*Agapornis pullarius*” MN481404, NC_045368 (Chen et al. 2019e)

Fig. S40

Metadata: Captive-bred specimen of *A. personatus* was sampled from the Nanjing Hongshan Forest Zoo (N32º09’, E118º80’), Jiangsu province, China. Whole blood sample was collected from the individual and stored in the Forest Police Forensic Centre of State Forestry Administration (Accession S2019J1101201). (Chen et al. 2019e: 3772)

Phylogenetic position:

ND2 = could not be verified due to lack of sequences of *A. personatus* and *A. pullarius.*

CO1 = In an unresolved clade with *Agapornis nigrigenis, A. fischeri, A. lilianae*, and *A. personatus*; unfortunately, there were no sequences of *A. pullarius*.

Cyt *b* = In a well-supported but unresolved clade with *Agapornis nigrigenis, A. fischeri, A. lilianae*, and *A. personatus*; distant from several *A. pullarius*.

Sequence integrity: not investigated

Interpretation: mislabelled on GenBank as *Agapornis pullarius* (correctly identified in paper as *Agapornis personatus*)

Subsequent usage: not subsequently used (January 2020)

Problems noted in other works: None.

Relevance: This was the only mitogenome presumed to be of this species (January 2020).

References:

Chen, YX, Hou, SL, Zhou, YW & Huang, YL 2019e. Characterization of the complete mitochondrial genome of *Agapornis personatus* and its phylogenetic analysis, Mitochondrial DNA Part B 4: 3772-3773.

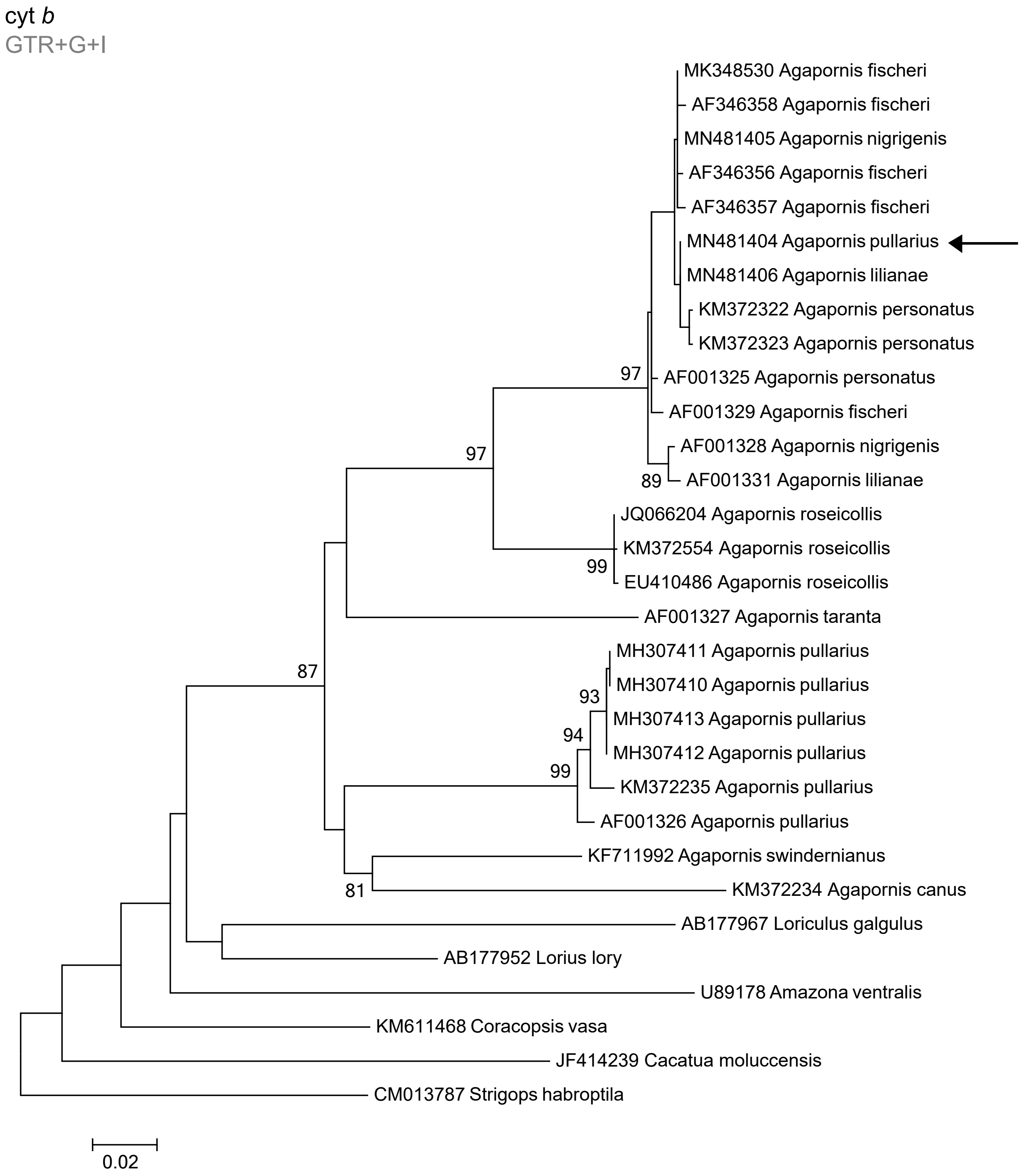

Figure S40. Maximum Likelihood phylogenies of *Agapornis pullarius* (MN481404) and related taxa based on mitochondrial sequences. Numbers at branches are bootstrap support values (>70%) based on 1000 replicates.

50. “*Brotogeris cyanoptera*” HM627323, NC_015530 (Pacheco et al. 2011)

Fig. S41

Metadata: The tissue sample was provided by the Phelps Ornithological Collection (Caracas, Venezuela), number ML675 (Pacheco et al. 2011).
Phylogenetic position:
ND2 = among *B. cyanoptera* but on a slightly longer branch
CO1 = could not be verified due to lack of reference sequences of *B. cyanoptera*
Cyt *b* = sister to *B. cyanoptera, B. chrysoptera, B. jugularis* and *B. pyrrhopterus*
Sequence integrity: cyt b: 63 nucleotides (between bp 105 and bp 1125, but mostly between bp 441 annd bp 1125) were not found among seven other sequences of *B. cyanoptera*. There was an inferred 3-bp gap at bp 858.
Interpretation: sequencing errors / numts
Subsequent usage:
Re-used in **10 mitogenomic phylogenies published before 1 January 2020**: Eberhard & Wright 2016, Nabholz et al. 2016, Johansson et al. 2018a, Lima et al. 2018, Sarker et al. 2018, Urantowka et al. 2018, Li & Duan 2019, Sarker et al. 2019b, Sarker et al. 2019c, Xu et al. 2019.
Problems noted in other works: none.
Relevance: This was the only mitogenome presumed to be of this species (January 2020).
References:

Eberhard, JR & Wright, TF 2016. Rearrangement and evolution of mitochondrial genomes in parrots. Molecular Phylogenetics and Evolution 94: 34-46.

Johansson, US, Ericson, PGP, Blom, MP & Irestedt, M 2018a. The phylogenetic position of the extinct Cuban Macaw *Ara tricolor* based on complete mitochondrial genome sequences. Ibis 160: 666-672.

Li, Y & Duan, Y 2019. Sequence and phylogenetic analysis of the mitochondrial genome for red-breasted parakeet *Psittacula alexandri* (Psittaciformes: Psittacidae). Mitochondrial DNA Part B 4: 3614-3615.

Lima, NCB, Soares, AER, Almeida, LGDP, Costa, IRD, Sato, FM, Schneider, P, Aleixo, A, Schneider, MP, Santos, FR, Mello, CV, Miyaki, C, Vasconcelos, TR & Prosdocimi, F 2018. Comparative mitogenomic analyses of *Amazona* parrots and Psittaciformes. Genetics and Molecular Biology 41: 593-604.

Nabholz, B, Lanfear, R & Fuchs, J 2016. Body mass-corrected molecular rate for bird mitochondrial DNA. Mol. Ecol. 25: 4438-4449.

Pacheco, MA, Battistuzzi, FU, Lentino, M, Aguilar, R, Kumar, S & Escalante, AA 2011. Evolution of modern birds revealed by mitogenomics: timing the radiation and origin of major orders. Mol. Biol. Evol. 28: 1927–1942.

Sarker, S, Das, S, Ghorashi, SA, Forwood, JK, Helbig, K & Raidal, SR 2018. The first complete mitogenome of Red-bellied Parrot (*Poicephalus rufiventris*) resolves phylogenetic status within Psittacidae. Mitochondrial DNA Part B 3: 195-197.

Sarker, S, Sutherland, M, Talukder, S, Das, S, Forwood, JK, Helbig, K & Raidal, SR 2019b. The first complete mitogenome of Indian ringneck (*Psittacula krameri*) demonstrates close phylogenetic relationship with Eclectus parrot. Mitochondrial DNA Part B 4: 3579-3581.

Sarker, S, Talukder, S, Sutherland, M, Forwood, JK, Helbig, K & Raidal, SR 2019c. Characterization of the first mitochondrial genome of a Little Corella (*Cacatua sanguinea*) and its phylogenetic implications. Mitochondrial DNA Part B 4: 3792-3794.

Urantówka, AD, Kroczak, A, Silva, T, Padrón, RZ, Gallardo, NF, Blanch, J, Blanch, B & Mackiewicz, P 2018. New insight into parrots’ mitogenomes indicates that their ancestor contained a duplicated region. Molecular Biology and Evolution 35: 2989-3009.

Xu, N, Zhang, Q, Chen, R & Liu, H 2019. The complete mitogenome of red-collared lorikeet (*Trichoglossus rubritorquis*) and its phylogenetic analysis. Mitochondrial DNA Part B 4: 3116-3117.

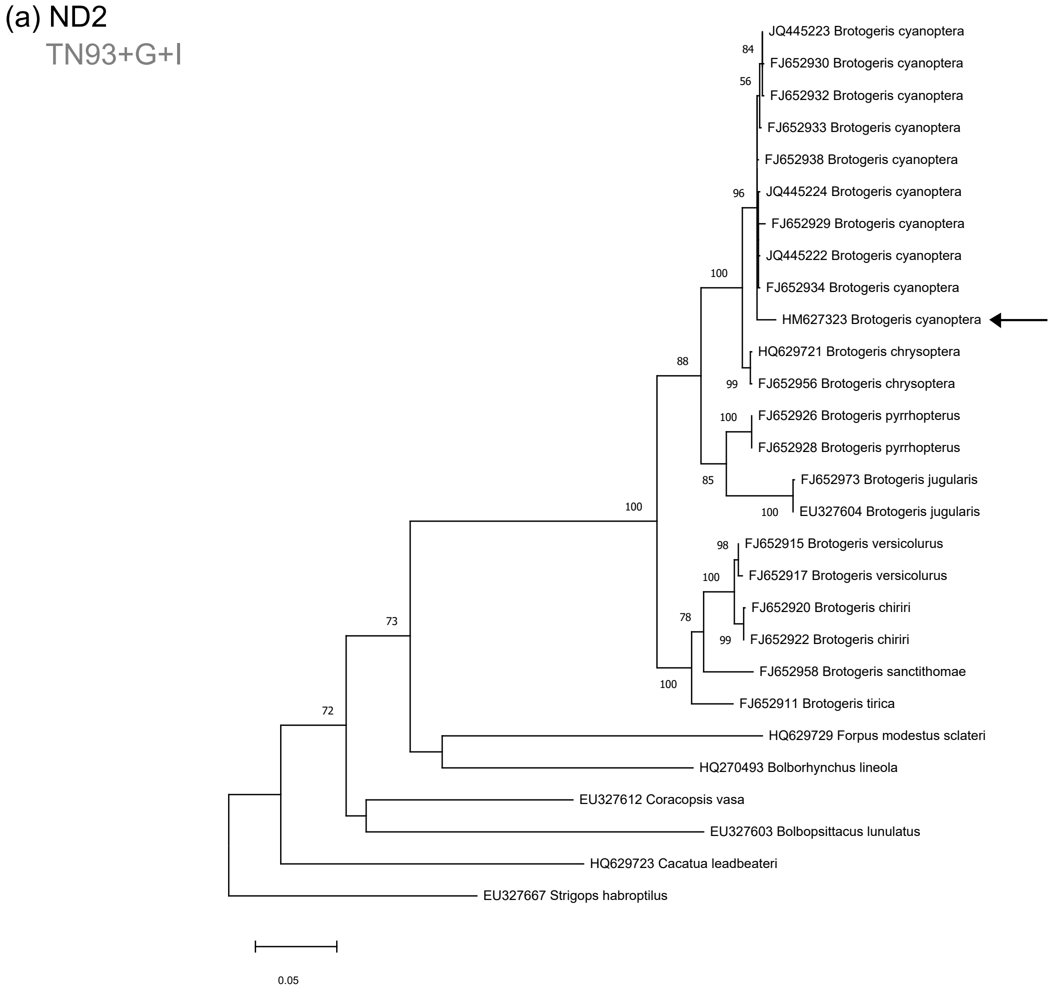

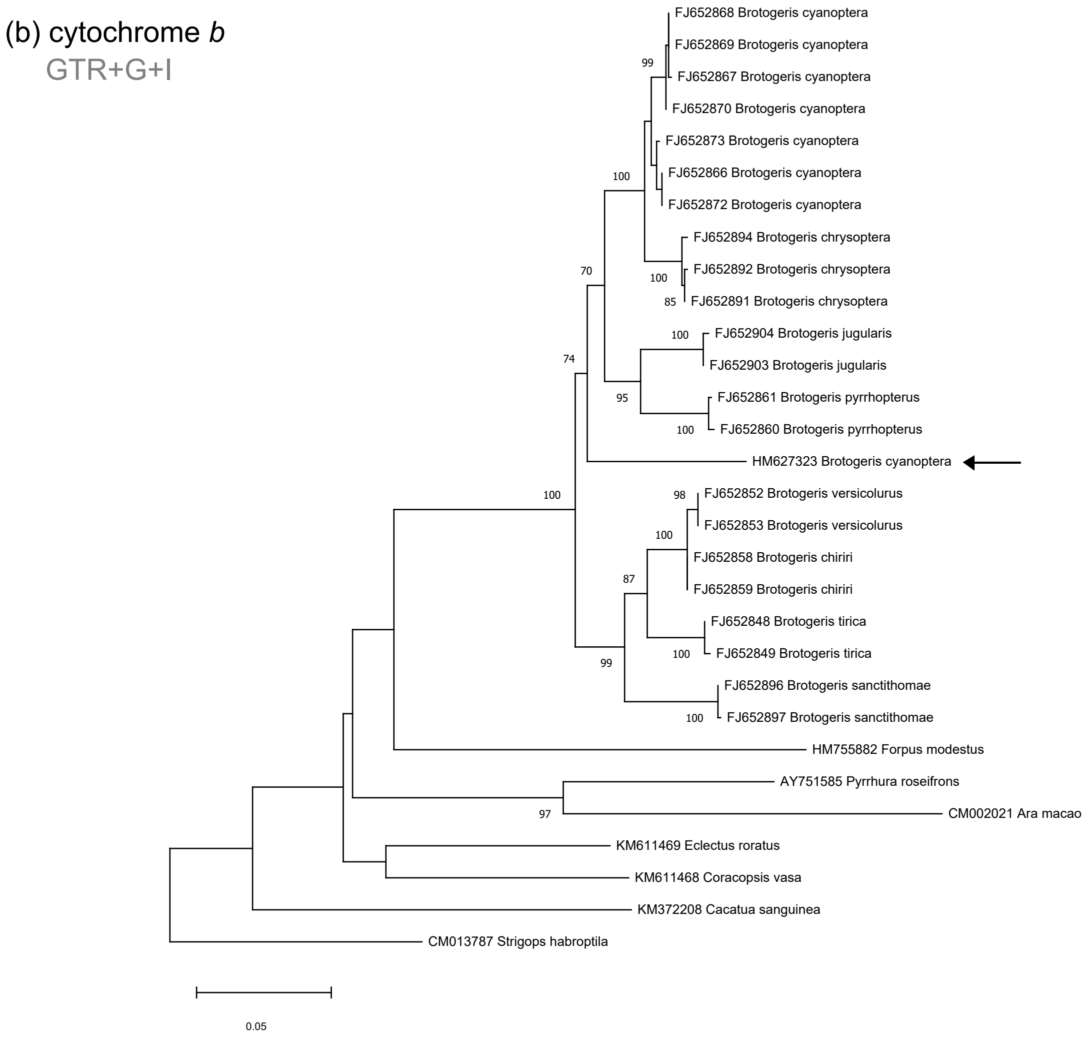

Figure S41. Maximum Likelihood phylogenies of *Brotogeris cyanoptera* (HM627323) and related taxa based on mitochondrial sequences. Numbers at branches are bootstrap support values (>70%) based on 1000 replicates.

51. “*Thamnophilus nigrocinereus*” KJ909192 (Barker 2014)

Fig. S42

Metadata: FMNH 391354 (Barker 2014). According to VERTNET (http://portal.vertnet.org/search?q=FMNH+391354+), the collection locality is Brazil, Amapa: Amapa, Fazenda Itapoa

Phylogenetic position:

ND2 = *Percnostola rufifrons*

CO1 = *Percnostola rufifrons*

Cyt *b* = sister to *Percnostola rufifrons* with a deep divergence

Sequence integrity:

cyt *b* problematic; sequence divergence with *Percnostola rufifrons* (EF639977) 5.6% (uncorrected p-distance, with complete deletion of missing sites); consecutive 60 bp partitions did not match with any species (BLAST < 97%), except bp 1080 – 1143 which showed a match with *Percnostola rufifrons* (BLAST 100% match).

Interpretation: misidentification + additional issues with cyt *b*

Subsequent usage:

Re-used in **5 mitogenomic phylogenies published before 1 January 2020**: Mitchell et al. 2016, Nabholz et al. 2016, Caparroz et al. 2018, Bustamante et al. 2019, Mackiewicz et al. 2019.

Problems noted in other works: None.

Relevance: This was the only mitogenome presumed to be of this species, genus and family (January 2020).

References:

Barker, FK 2014. Mitogenomic data resolve basal relationships among passeriform and passeridan birds. Molecular Phylogenetics and Evolution 79: 313–324.

Bustamante, DE, Hughey, JR, Mendoza, JE, Tineo, D, Perez, J, Oliva, M, Leiva, S & Calderon, MS 2019. The complete mitochondrial genome of the national bird of Peru: *Rupicola peruvianus* (Aves, Passeriformes, Cotingidae). Mitochondrial DNA Part B 4: 3975-3976.

Caparroz, R, Rocha, AV, Cabanne, GS, Tubaro, P, Aleixo, A, Lemmon, EM & Lemmon, AR 2018. Mitogenomes of two neotropical bird species and the multiple independent origin of mitochondrial gene orders in Passeriformes. Molecular Biology Reports 45: 279-285.

Mackiewicz, P, Urantówka, AD, Kroczak, A & Mackiewicz, D 2019. Resolving phylogenetic relationships within Passeriformes based on mitochondrial genes and inferring the evolution of their mitogenomes in terms of duplications. Genome Biology and Evolution, 11: 2824-2849.

Mitchell, KJ, Wood, JR, Llamas, B, McLenachan, PA, Kardailsky, O, Scofield, RP, Worthy, TH & Cooper, A 2016. Ancient mitochondrial genomes clarify the evolutionary history of New Zealand’s enigmatic acanthisittid wrens. Molecular Phylogenetics and Evolution 102: 295-304.

Nabholz, B, Lanfear, R & Fuchs, J 2016. Body mass-corrected molecular rate for bird mitochondrial DNA. Mol. Ecol. 25: 4438-4449.

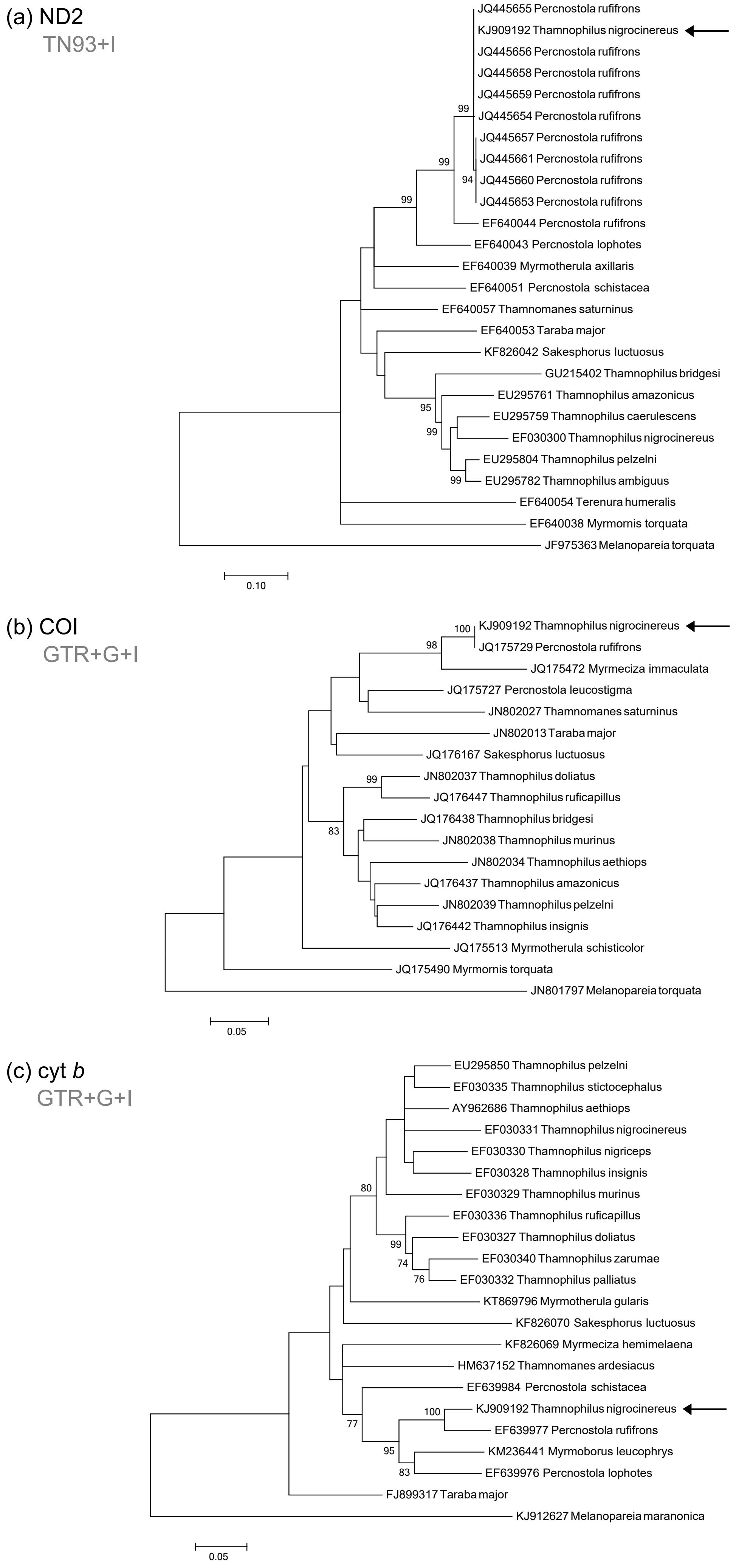

Figure S42. Maximum Likelihood phylogenies of *Thamnophilus nigrocinereus* (KJ909192), and related taxa based on mitochondrial sequences. Numbers at branches are bootstrap support values (>70%) based on 1000 replicates.

52. “*Lanius tephronotus*” JX486029, NC_021105 (Qian et al. 2013)

Fig. S43

Metadata: “an adult male *L. tephronotus* (code Kan-A0023) was extracted from the muscle tissue” (Qian et al. 2013)
Phylogenetic position:
ND2 = *Lanius schach*
CO1 = *Lanius schach*
Cyt *b* = *Lanius schach*
Sequence integrity: not investigated
Interpretation: misidentification
Subsequent usage:
Re-used in **13 mitogenomic phylogenies published before 1 January 2020**: Barker 2014, Gibb et al. 2015, Eo & An 2016b, Liu et al. 2016c, Yang et al. 2016a, Yang et al. 2016b, Liu et al. 2016d, Wang & Liang 2016, Krzeminska et al. 2016a, Nabholz et al. 2016, Zhang et al. 2018b, Sun et al. 2019b, Mackiewicz et al. 2019.
Cited by **4 other papers**: Kan et al. 2013, Liu et al. 2016c, Ren et al. 2016, Mu et al. 2014
Problems noted in other works: None.
Relevance: This was the only mitogenome presumed to be of this species (January 2020)
References:

Barker, FK 2014. Mitogenomic data resolve basal relationships among passeriform and passeridan birds. Molecular Phylogenetics and Evolution 79: 313–324.

Eo, SH & An, J 2016. Mitochondrial genome sequence of Black Paradise Flycatcher (Aves: Monarchidae) and its phylogenetic position. Mitochondrial DNA Part B1: 454-455.

Gibb, GC, England, R, Hartig, G, McLenachan, PA, Taylor Smith, BL, McComish, BJ, Cooper, A & Penny, D 2015. New Zealand passerines help clarify the diversification of major songbird lineages during the Oligocene. Genome Biol. Evol. 7: 2983–2995.

Kan, X, Yuan, J, Zhang, L, Li, X, Yu, L, Chen, L, Guo, Z & Yang, J 2013. Complete mitochondrial genome of the Tristram's Bunting, *Emberiza tristrami* (Aves: Passeriformes): The first representative of the family Emberizidae with six boxes in the central conserved domain II of control region. Mitochondrial DNA 24: 648-650.

Krzeminska, U, Wilson, R, Rahman, S, Song, BK, Seneviratne, S, Gan, HM & Austin, CM 2016a. Mitochondrial genomes of the jungle crow *Corvus macrorhynchos* (Passeriformes: Corvidae) from shed feathers and a phylogenetic analysis of genus *Corvus* using mitochondrial protein-coding genes. Mitochondrial DNA Part A 27: 2668-2670.

Liu, F, Bao, X, Fan, Y & Li, J 2016d. Sequencing and analysis of the complete mitochondrial genome of Rufous-tailed Shrike, *Lanius isabellinus* (Passeriformes, Laniidae). Mitochondrial DNA Part A 27: 2625-2626.

Liu, CZ, Wei GH, Hu JH, Liu XY 2016c. Complete mitochondrial genome of the Swan Goose (*Anser cygnoides* L.) and its phylogenetic analysis. Mitochondrial DNA A 27: 2427-2428.

Liu, F, Bao, X, Fan, Y, Li, J, & Yao, X 2016c. Sequencing and analysis of the complete mitochondrial genome of Brown Shrike, *Lanius cristatus* (Passeriformes, Laniidae). Mitochondrial DNA Part A 27(5): 3544-3546.

Mackiewicz, P, Urantówka, AD, Kroczak, A & Mackiewicz, D 2019. Resolving phylogenetic relationships within Passeriformes based on mitochondrial genes and inferring the evolution of their mitogenomes in terms of duplications. Genome Biology and Evolution, 11: 2824-2849.

Mu, CY, Huang, ZY, Chen, Y, Wang, B, Su, YH, Li, Y, Sun, ZM, Xu, Q, Zhao, WM & Chen, GH 2014. [Complete sequence and gene organization of the *Anser cygnoides* mitochondrial genome.] Journal of Agricultural Biotechnology 22: 1482-1493. [In Chinese.]

Nabholz, B, Lanfear, R & Fuchs, J 2016. Body mass-corrected molecular rate for bird mitochondrial DNA. Mol. Ecol. 25: 4438-4449.

Qian, C, Yan, X, Guo, Z, Wang, Y, Li, X, Yang, J & Kan, X 2013. Characterization of the complete mitochondrial genome of the Grey-backed Shrike, *Lanius tephronotus* (Aves:

Passeriformes): The first representative of the family Laniidae with a novel CAA stop codon at the end of cox2 gene. Mitochondrial DNA 24: 359-361.

Ren, Q, Qian, C, Yuan, J, Li, X, Yang, J, Wang, P, Jiang, L, Zhang, Q, Wang, Y & Kan, X 2016. Complete mitochondrial genome of the black-capped bulbul, *Pycnonotus melanicterus* (Passeriformes: Pycnonotidae). Mitochondrial DNA Part A 27: 1378-1380.

Sun, Z, Li, Y, Duan, Y & Ma, J 2019b. Characterization of the complete mitochondrial genome of Hair-crested Drongo *Dicrurus hottentottus* (Passeriformes: Dicruridae). Mitochondrial DNA Part B 4: 2013-2014.

Wang, N & Liang, B 2016. Complete mitochondrial genome of a sunbird, *Aethopyga gouldiae* (Aves: Passeriformes), the first representative of Nectariniidae. Mitochondrial DNA Part A 27: 2356-2358.

Yang, DC, Peng, LF, & Lu, CH 2016a. Sequencing and analysis of the complete mitochondrial genome of long-tailed Shrike, *Lanius schach* (Aves: Laniidae). Mitochondrial DNA Part B 1: 23-24.

Yang, MX, Wang, QX, Xiao, H & Yang, C 2016b. Sequencing complete mitochondrial genome of *Lanius sphenocercus sphenocercus* (Passeriformes: Laniidae) using Illunima HiSeq 2500. Mitochondrial DNA Part B 1: 306-307.

Zhang, H, Bai, Y, Shi, X, Sun, L, Wang, Z & Wu, X 2018b. The complete mitochondrial genomes of *Tarsiger cyanurus* and *Phoenicurus auroreus*: a phylogenetic analysis of Passeriformes. Genes & Genomics 40: 151-165.

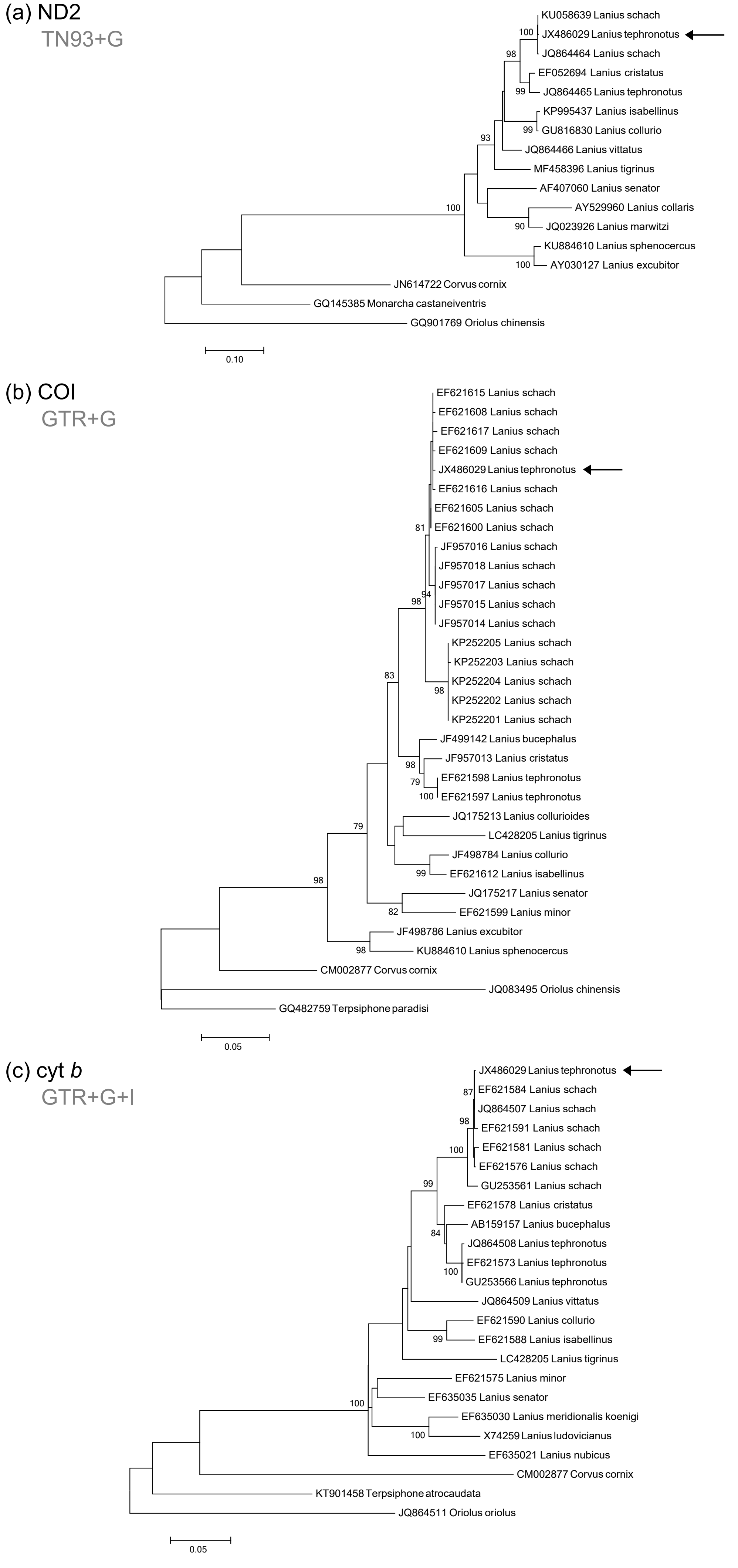

Figure S43. Maximum Likelihood phylogenies of *Lanius tephronotus* (JX486029) and related taxa based on mitochondrial sequences. Numbers at branches are bootstrap support values (>70%) based on 1000 replicates.

53. “*Corvus coronoides*” MF370524, NC_035877 (Sarker et al. 2017a)

Fig. S44

Metadata: “The blood sample used in this study was obtained from an Australian raven in the wild (year of sampling: 2015; GPS location: 35°21’24.92’’S, 149°13’20.182’’E), and stored in appropriate condition by the Veterinary Diagnostic Laboratory (VDL), Charles Sturt University under the accession number CS15-3763.” (Sarker et al. 2017: 473)

Phylogenetic position:

ND2 = verified with four *Corvus coronoides coronoides*, which are very similar to MF370524 (sequence divergence 0.8-1.2% p-distance, with complete deletion of missing sites).

CO1 = sister to *Corvus coronoides* (n=1, JN801437) but on a very long branch (sequence divergence 8.4%; p-distance, with complete deletion of missing sites).

Cyt *b* = sister to *Corvus coronoides* (n=1, AF197837) but on a very long branch (sequence divergence 5.9% p-distance, with complete deletion of missing sites)

Sequence integrity:

COI contains numerous single nucleotide differences from JN801437, especially from nucleotides 100 to 391. This 292-bp fragment differs >12% from all other bird sequences on GenBank, and 16% from a single sequence of *Corvus coronoides* (JN801437). A BLAST search showed a close match with the house mouse *Mus musculus*.

cyt *b* contains numerous single nucleotide differences from AF197837, especially from nucleotides 475 to 690. This 216-bp fragment differs >19% from all other bird sequences on GenBank, and 23% from a single sequence of *Corvus coronoides* (AF197837). A BLAST search showed a close match with the house mouse *Mus musculus*.

Problems noted in other works: None.

Interpretation: chimera

Subsequent usage:

Re-used in **2 mitogenomic phylogenies published before 1 January 2020**: Liu et al. 2018d, Mackiewicz et al. 2019.

Relevance: This was the only mitogenome presumed to be of this species (January 2020)

References:

Liu, R, Chen, R, Liu, J, Xiong, Y & Kan, X 2018d. Complete mitochondrial genome of *Urocissa erythroryncha* (Passeriformes: Corvidae). Mitochondrial DNA Part B 3: 691-692.

Mackiewicz, P, Urantówka, AD, Kroczak, A & Mackiewicz, D 2019. Resolving phylogenetic relationships within Passeriformes based on mitochondrial genes and inferring the evolution of their mitogenomes in terms of duplications. Genome Biology and Evolution, 11: 2824-2849.

Sarker, S, Helbig, K & Raidal, SR 2017a. The first complete mitochondrial genome sequence of an Australian raven (*Corvus coronoides*). Mitochondrial DNA Part B 2: 473-474.

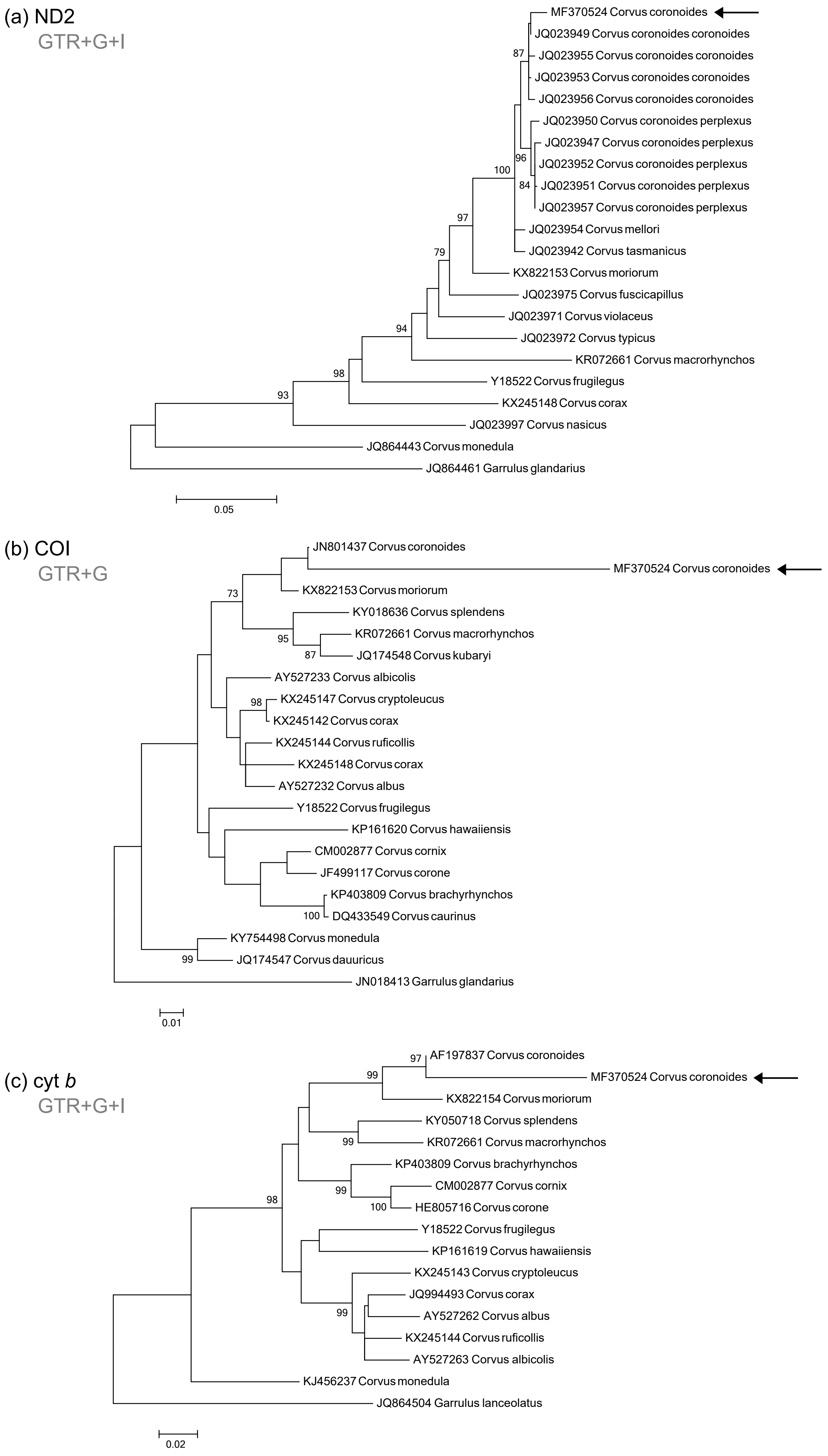

Figure S44. Maximum Likelihood phylogenies of *Corvus coronoides* (MF370524) and related taxa based on mitochondrial sequences. Numbers at branches are bootstrap support values (>70%) based on 1000 replicates.

54. "*Quoyornis* (*Eopsaltria*) *georgiana*" KM374638 (Morales et al. 2015)

Fig. S45

Metadata: individual sample name B48503, collection coordinates -33.17, 136.92 (Morales et al. 2015: online supporting materials).
Phylogenetic position:
ND2 = *Eopsaltria griseogularis*
CO1 = *Eopsaltria griseogularis*
Cyt *b* = *Eopsaltria griseogularis*
Sequence integrity: not investigated
Interpretation: misidentification. The sequence was not specifically identified in the paper as either species, but it was identified as *Quoyornis georgiana* both in the supporting information (**II**) and on GenBank.
Subsequent usage:
Used by **1 paper**: Yoon et al. 2017 (**DNA identification**).
Problems noted in other works: None.
Relevance: This was one of two mitogenomes of this species (Morales et al. 2015).
References:
Morales, HE, Pavlova, A, Joseph, L & Sunnucks, P 2015. Positive and purifying selection in mitochondrial genomes of a bird with mitonuclear discordance. Molecular Ecology 24: 2820-2837.
Yoon, TH, Kang, HE, Lee, SR, Lee, JB, Baeck, GW, Park, H & Kim, HW 2017. Metabarcoding analysis of the stomach contents of the Antarctic Toothfish (*Dissostichus mawsoni*) collected in the Antarctic Ocean. PeerJ, 5, e3977. (suppl data 2).

55. "*Eopsaltria griseogularis*" KM374625 (Morales et al. 2015)

Fig. S45

Metadata: individual sample name B31918, collection coordinates -34.46, 116.11 (Morales et al. 2015: online supporting materials).
Phylogenetic position:
ND2 = *Quoyornis* (*Eopsaltria*) *georgiana*
CO1 = *Quoyornis* (*Eopsaltria*) *georgiana*
Cyt *b* = *Quoyornis* (*Eopsaltria*) *georgiana*
Sequence integrity: not investigated
Interpretation: misidentification. The sequence was not specifically identified in the paper as either species, but it was identified as *Eopsaltria griseogularis* both in the supporting information (**II**) and on GenBank.
Subsequent usage:
Re-used in **1 mitogenomic phylogeny published before 1 January 2020**: Mackiewicz et al. 2019.
Problems noted in other works: None.
Relevance: This was one of five mitogenomes of this species (Morales et al. 2015).
References:
Mackiewicz, P, Urantówka, AD, Kroczak, A & Mackiewicz, D 2019. Resolving phylogenetic relationships within Passeriformes based on mitochondrial genes and inferring the evolution of their mitogenomes in terms of duplications. Genome Biology and Evolution 11: 2824-2849.
Morales, HE, Pavlova, A, Joseph, L & Sunnucks, P 2015. Positive and purifying selection in mitochondrial genomes of a bird with mitonuclear discordance. Molecular Ecology 24: 2820-2837.

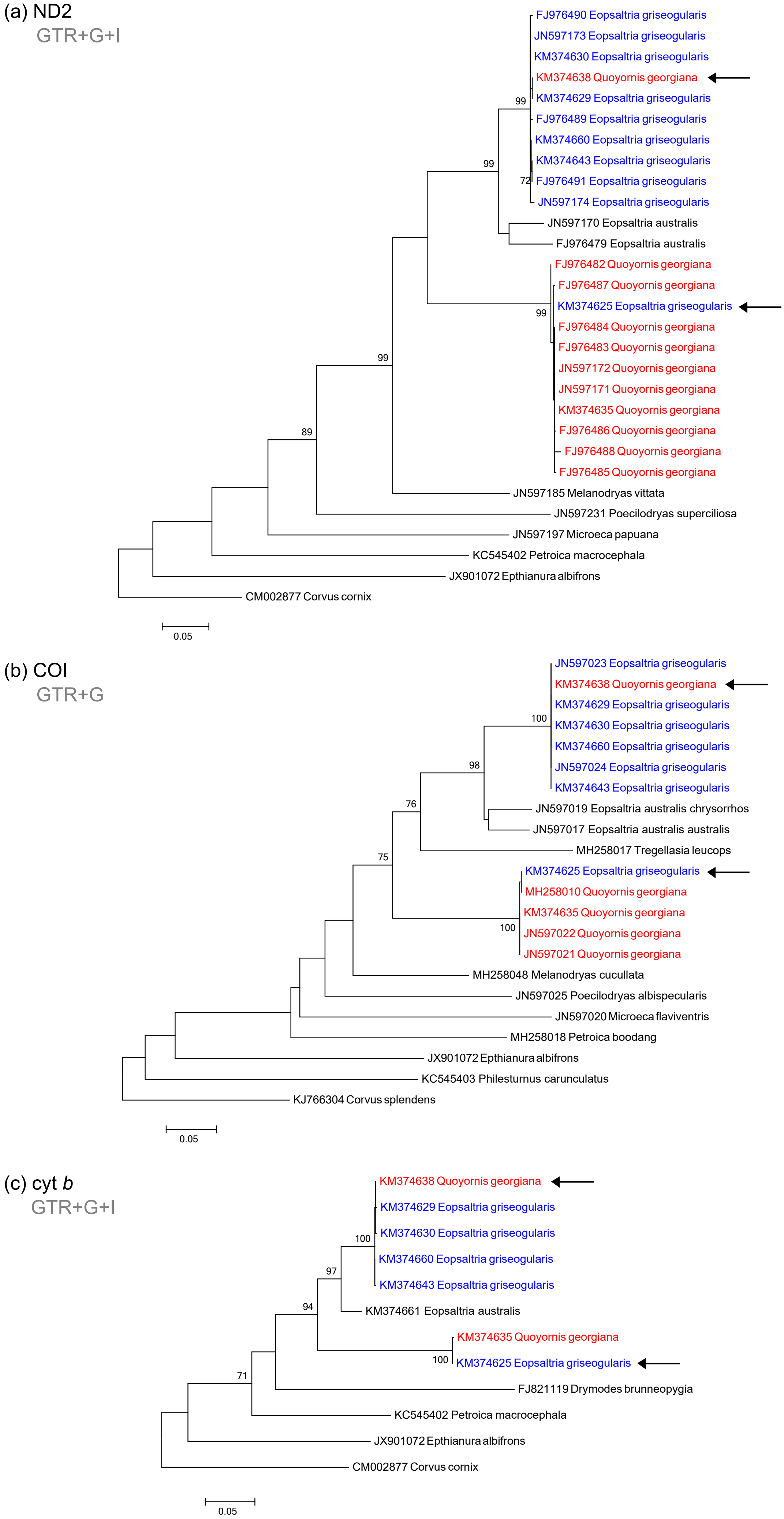

Figure S45. Maximum Likelihood phylogenies of *Eopsaltria griseogularis* (KM374625), *Quoyornis georgiana* (KM374638) and related taxa based on mitochondrial sequences. Numbers at branches are bootstrap support values (>70%) based on 1000 replicates.

56. "*Bombycilla cedrorum*" KJ909187 (Barker 2014)

Fig. S46

Metadata: MMNH 47265 (Barker 2014). According to VERTNET (http://portal.vertnet.org/search?q=MMNH+47265+), the collection locality is United States, Minnesota, Wright: Woodland State WMA

Phylogenetic position:

ND2 = *Bombycilla cedrorum*

CO1 = divergent from *Bombycilla cedrorum*; full COI was 7% divergent from other *B. cedrorum.*

Cyt *b* = sister to *Gracula religiosa*; full cyt *b* was >8% divergent from *B. cedrorum*.

Sequence integrity:

COI: bp 1 – circa 82 = *Bombycilla cedrorum*; bp circa 88 – 355 = *Gracula religiosa*; bp circa 388 – 586 = *Bombycilla cedrorum.*

cyt b: bp 139 – 703 contains multiple nucleotides not found in *B. cedrorum*; BLAST finds 99.8 – 100% similarity to *Gracula religiosa* (n=2);

Interpretation: chimera

Subsequent usage:

Re-used in **2 mitogenomic phylogenies published before 1 January 2020**: Nabholz et al. 2016, Mackiewicz et al. 2019.

Sequence mentioned / used in **1 paper**: Anmarkrud & Lifjeld (2017)

Problems noted in other works: None.

Relevance: This was the only mitogenome presumed to be of this species and the family Bombycillidae (January 2020).

References:

Anmarkrud, JA & Lifjeld, JT 2017. Complete mitochondrial genomes of eleven extinct or possibly extinct bird species. Molecular Ecology Resources 17: 334-341.

Barker, FK 2014. Mitogenomic data resolve basal relationships among passeriform and passeridan birds. Molecular Phylogenetics and Evolution 79: 313–324.

Mackiewicz, P, Urantówka, AD, Kroczak, A & Mackiewicz, D 2019. Resolving phylogenetic relationships within Passeriformes based on mitochondrial genes and inferring the evolution of their mitogenomes in terms of duplications. Genome Biology and Evolution 11: 2824-2849.

Nabholz, B, Lanfear, R & Fuchs, J 2016. Body mass-corrected molecular rate for bird mitochondrial DNA. Molecular Ecology 25: 4438-4449.

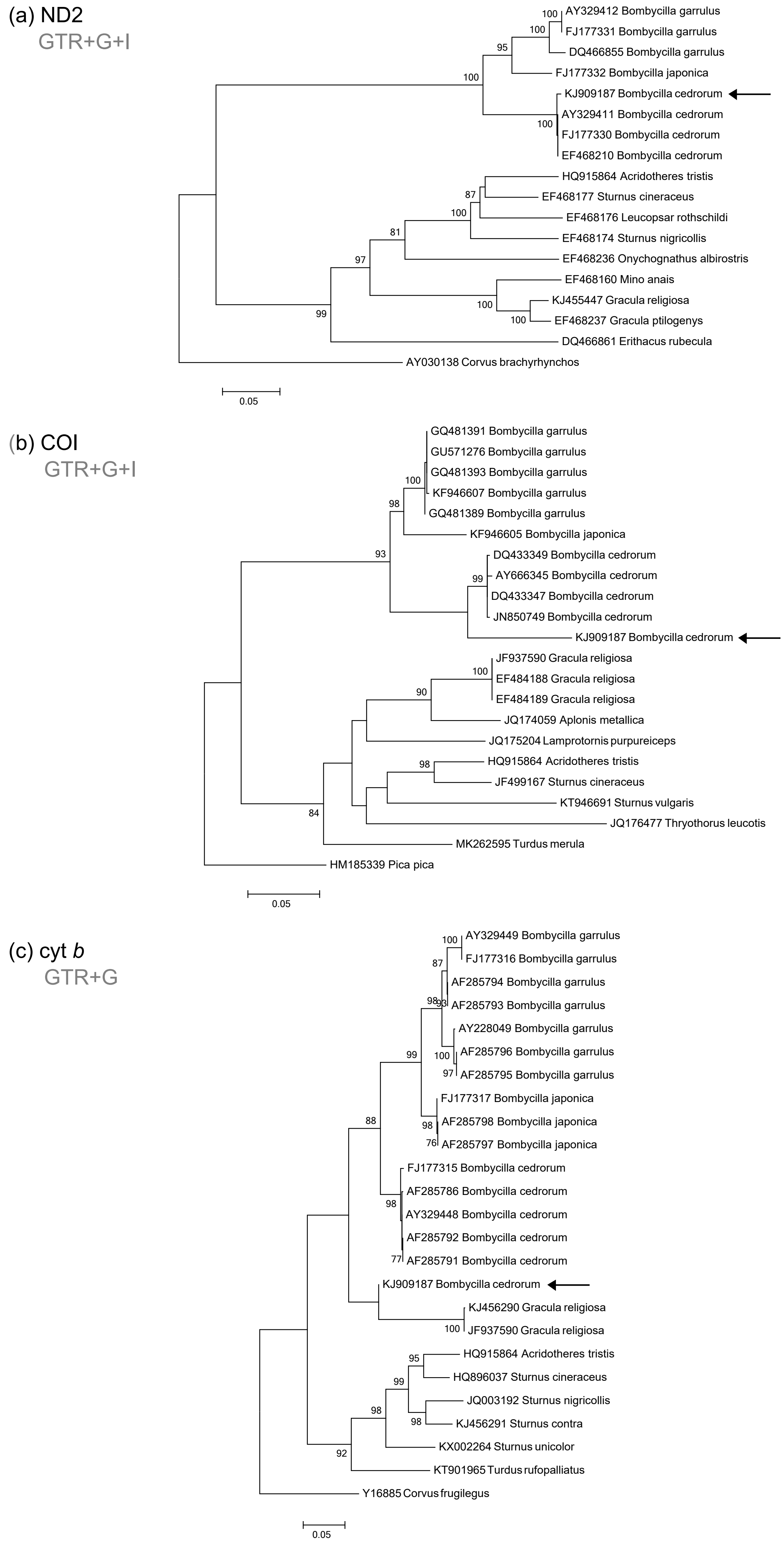

Figure S46. Maximum Likelihood phylogenies of *Bombycilla cedrorum* (KJ909187) and related taxa based on mitochondrial sequences. Numbers at branches are bootstrap support values (>70%) based on 1000 replicates.

57. “*Periparus ater*” KM588075, NC_026223 (Zhang et al. 2016g)

Fig. S47

Metadata: In this study, the sample was collected from Gansu, China (Zhang et al. 2016g: 2008).
Phylogenetic position:
ND2 = *Periparus rubidiventris*
CO1 = sister to all *P. ater*, but highly divergent. (One COI sequence of ‘*Periparus rubidiventris*’ on GenBank (HQ228194, Dai et al. 2010a) was nested among *P. ater* and appears to represent a misidentified *P. ater*.)
Cyt *b* = *Periparus rubidiventris*
Sequence integrity: not investigated
Interpretation: misidentification
Subsequent usage:
Re-used in **7 mitogenomic phylogenies published before 1 January 2020**: Li et al. 2016b, Li et al. 2016g, Wang et al. 2015b, Nabholz et al. 2016, Wen et al. 2017b, Liu et al. 2019m, Mackiewicz et al. 2019.
Mentioned in **1 other paper**: Li et al. 2017b
Problems noted in other works: None.
Relevance: This was the only mitogenome presumed to be of this species and genus (January 2020).
References:

Dai, C-Y, Zhang, R-Y, Yin, Z-H & Lei, F-M 2010a. Recognizing species of Paridae by DNA barcodes. Acta Zootaxonomica Sinica 35: 835-841. [in Chinese]

Li, S, Luo, A, Li, G & Li, W 2016g. Complete mitochondrial genome of the Isabelline Wheatear *Oenanthe isabellina* (Passeriformes, Muscicapidae). Mitochondrial DNA Part B 1: 355-356.

Li, X, Lin, L, Cui, A, Bai, J, Wang, X, Xin, C, Zhang, Z, Yang, C, Gao, R, Huang, Y & Lei, F 2016b. Taxonomic status and phylogenetic relationship of tits based on mitogenomes and nuclear segments. Molecular Phylogenetics and Evolution 104: 14-20.

Li, X, Lin, L, Cui, A, Bai, J, Wang, X, Xin, C, Zhang, Z, Yang, C, Gao, R, Huang, Y & Lei, F 2017b. Data on taxonomic status and phylogenetic relationship of tits. Data in Brief 10: 390-397.

Liu, Z, Liu, J, Chen, X, Jin, Z, Luo, A & Li, S 2019m. Phylogenetic relationship and characterization of the complete chloroplast genome of the alpine leaf-warbler in Qinghai-Tibet Plateau. Mitochondrial DNA Part B 4: 3582-3584.

Mackiewicz, P, Urantówka, AD, Kroczak, A & Mackiewicz, D 2019. Resolving phylogenetic relationships within Passeriformes based on mitochondrial genes and inferring the evolution of their mitogenomes in terms of duplications. Genome Biology and Evolution 11: 2824-2849.

Nabholz, B, Lanfear, R & Fuchs, J 2016. Body mass-corrected molecular rate for bird mitochondrial DNA. Molecular Ecology 25: 4438-4449.

Wang, X, Liu, N, Zhang, H, Yang, XJ, Huang, Y & Lei, F 2015b. Extreme variation in patterns of tandem repeats in mitochondrial control region of yellow-browed tits (*Sylviparus modestus*, Paridae). Scientific Reports 5, 13227.

Wen, L, Fu, Y, Dai, B & Liao, J 2017b. The complete mitochondrial genome of the green-backed tit *Parus monticolus* (Passeriformes: Paridae). Mitochondrial DNA Part A 28: 79-80.

Zhang, Z, Wang, XY, Xin, C & Huang, Y 2016g. The complete mitochondrial genome of *Parus ater* (Passeriformes, Paridae). Mitochondrial DNA Part A 27: 2008-2009.

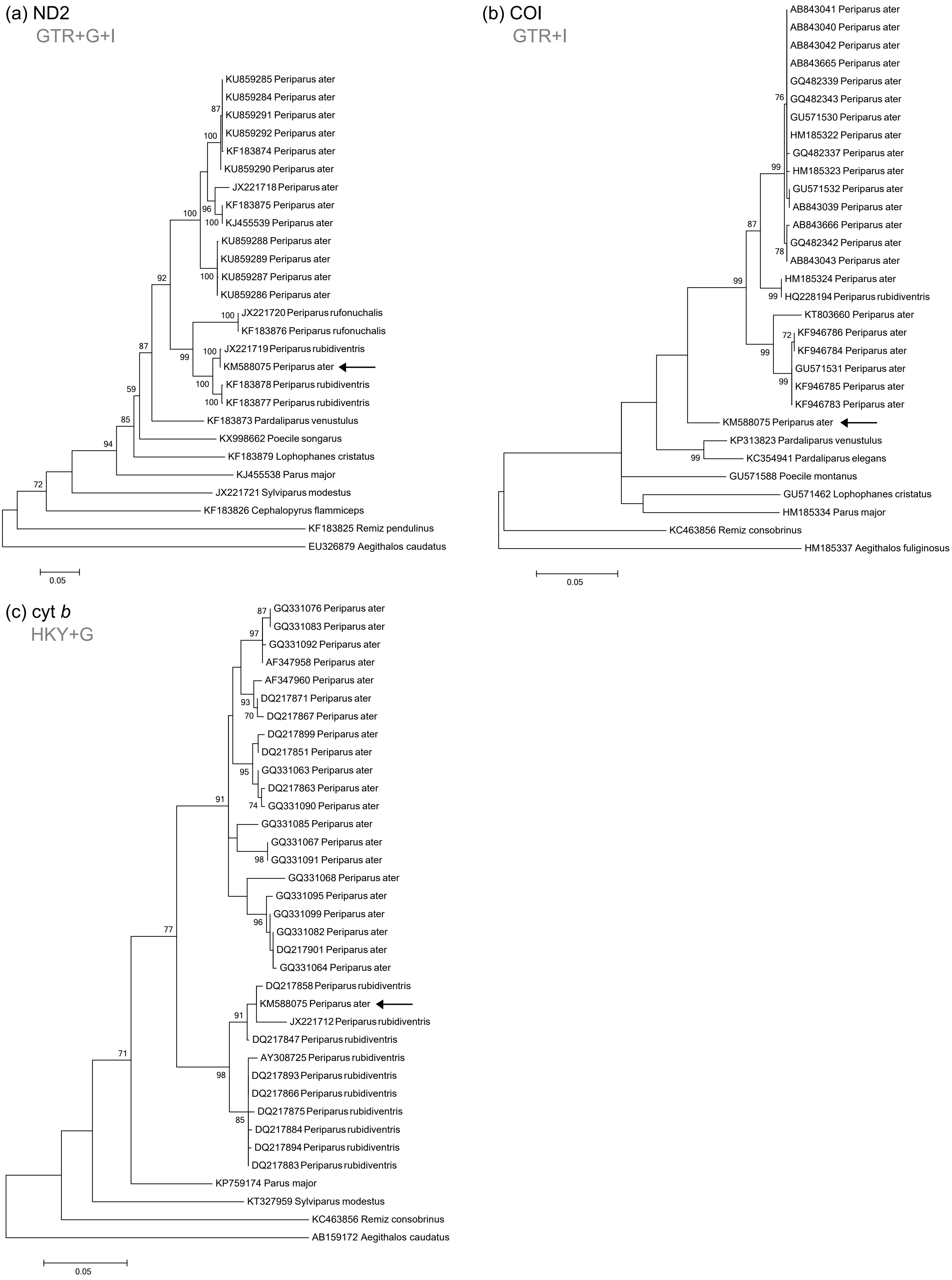

Figure S47. Maximum Likelihood phylogenies of *Periparus ater* (KM588075) and related taxa based on mitochondrial sequences. Numbers at branches are bootstrap support values (>70%) based on 1000 replicates.

58. “*Poecile palustris*” KX388475 (Li et al. 2016b, 2017b)

Fig. S48

Metadata: sample locality is Beach forestry centre, Zhouqu County, Gansu Province (Li et al. 2017b: 394)
Phylogenetic position:
ND2 = *Poecile weigoldicus*
CO1 = *Poecile weigoldicus*
Cyt *b* = *Poecile weigoldicus*
Sequence integrity: not investigated
Interpretation: misidentification. Nuclear DNA sequences of the same bird are therefore also misidentified: MOS (KX388400), FGB (KX388415), ALDOB (KX388430), PCBD1 (KX388445), CALB1 (KX388460) (Li et al. 2017).
Subsequent usage:
Re-used in **1 mitogenomic phylogeny published before 1 January 2020**: Liu et al. 2019m.
Problems noted in other works: None.
Relevance: Another mitogenome of *Poecile palustris* was published by Day et al. (2016) (KP184518).
References:

Dai, C, Chen, K, Zhang, R, Yang, X, Yin, Z, Tian, H, Zhang, Z, Hu, Y & Lei, F 2010b. Molecular phylogenetic analysis among species of Paridae, Remizidae and Aegithalos based on mtDNA sequences of COI and cyt b. Chinese Birds 1: 112-123.

Day, JC, Broughton, RK & Hinsley, SA 2016. Sequence and organization of the complete mitochondrial genome of the marsh tit *Poecile palustris* (Aves: Paridae). Mitochondrial DNA Part A 27: 3212-3213.

Li, X, Lin, L, Cui, A, Bai, J, Wang, X, Xin, C, Zhang, Z, Yang, C, Gao, R, Huang, Y & Lei, F 2016b. Taxonomic status and phylogenetic relationship of tits based on mitogenomes and nuclear segments. Molecular Phylogenetics and Evolution 104: 14-20.

Li, X, Lin, L, Cui, A, Bai, J, Wang, X, Xin, C, Zhang, Z, Yang, C, Gao, R, Huang, Y & Lei, F 2017b. Data on taxonomic status and phylogenetic relationship of tits. Data in Brief 10: 390-397.

Liu, Z, Liu, J, Chen, X, Jin, Z, Luo, A & Li, S 2019m. Phylogenetic relationship and characterization of the complete chloroplast genome of the alpine leaf-warbler in Qinghai-Tibet Plateau. Mitochondrial DNA Part B 4: 3582-3584.

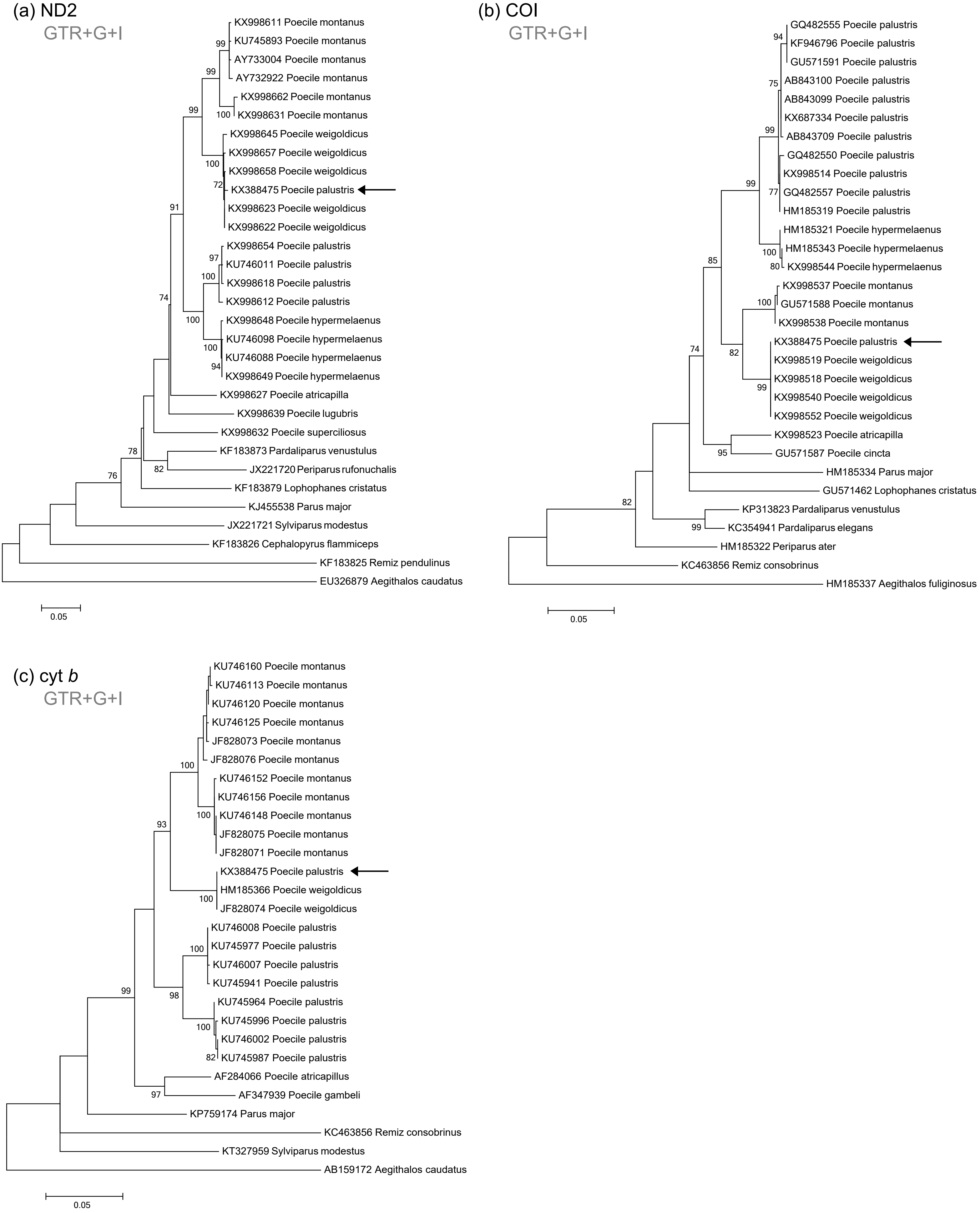

Figure S48. Maximum Likelihood phylogenies of *Poecile palustris* (KX388475) and related taxa based on mitochondrial sequences. Numbers at branches are bootstrap support values (>70%) based on 1000 replicates.

59. “*Pseudopodoces humilis*” HM535648, NC_014341 (Yang et al. 2010)

Fig. S49

Metadata: “The specimen of *Pseudopodoces humilis* was collected on August 6, 2003 in Qinghai Lake, Qinghai Province, and was immersed in absolute ethanol and stored in a freezer at −20 °C. The voucher specimen (4 783) is now stored in China. Bird Specimen, Key Laboratory of Animal Evolution and Systems, Institute of Zoology, Academy of Sciences.” (Yang et al. 2010: 334; translated from Chinese)

Phylogenetic position:

ND2 = *Aegithalos concinnus* but on a long branch.

CO1 = *Phoenicurus ochruros*

Cyt *b* = *Phoenicurus ochruros*

Sequence integrity: ND2 pos bp 1 – 601 = *Aegitalos concinnus*; bp 621 – 690 = *Phoenicurus ochruros*; bp 709 – 822 = *Aegitalos concinnus*; bp 832 – 1041 = *Phoenicurus ochruros*.

Interpretation: chimera of two other species.

Subsequent usage:

Re-used in **10 mitogenomic phylogenies published before 1 January 2020**: Marshall et al. 2013, Barker 2014, Ma et al. 2014 (**without comment on its strange position**), Gibb et al. 2015, Xu et al. 2016a, Peng et al. 2016b (**without comment on its strange position**), Peng et al. 2016c (**without comment on its strange position**), Zhang et al. 2018b, Wen & Liao 2016, Liu et al. 2019g.

Used in **10 other papers**: Yang et al. 2012, Wenzel et al. 2012, Li et al. 2014a, Li et al. 2014b, Li et al. 2015a, Wang et al. 2015, Chen et al. 2015, Xin et al. 2016, Li et al. 2016c, Yang et al. 2018.

Erroneous taxonomic conclusions: Paridae should be separated from the superfamily Sylvioidea and placed within the superfamily Muscicapoidea (Zhang et al. 2018b).

Problems noted in other works: Barker (2014): **“**One apparent artifact in these analyses regarded placement of a GenBank sequence (accession HM535648) labeled *Pseudopodoces* (Hume’s ground tit), which fell inside of a clade otherwise including members of the family Muscicapidae. This specimen is clearly mislabelled, as this genus is indisputably a highly-derived corvid-like member of the Paridae (James et al., 2003), ...**”**

Gibb et al. (2015, OSM): “*Pseudopodoces humilis* likely mislabeled, should probably be *Phoenicurus ochruros* (based on similarity to other sequences on Genbank, February 2014).”

Xin et al. (2016): “In fact, HM535648 was not complete sequence of ground tit.”

Relevance: only known mitogenome of this species until 2016, when a new sequence was published (Xin et al. 2016).

References:

Barker, FK 2014. Mitogenomic data resolve basal relationships among passeriform and passeridan birds. Molecular Phylogenetics and Evolution 79: 313–324.

Chen, DS, Qian, CJ, Ren, QQ, Wang, P, Yuan, J, Jiang, L, Bi, D, Zhang, Q, Wang, Y & Kan, XZ 2015. Complete mitochondrial genome of the Chinese Hwamei *Garrulax canorus* (Aves: Passeriformes): the first representative of the Leiothrichidae family with a duplicated control region. Genetics and Molecular Research 14: 8964-8976.

Gibb, GC, England, R, Hartig, G, McLenachan, PA, Taylor Smith, BL, McComish, BJ, Cooper, A & Penny, D 2015. New Zealand passerines help clarify the diversification of major songbird lineages during the Oligocene. Genome Biol. Evol. 7: 2983–2995.

Li, X, Huang, Y & Lei, F 2014a. Comparative and phylogenomic analyses on mitochondrial genomes of *Arborophila* species. Hereditas (Beijing) 36: 912-920.

Li, X, Huang, Y & Lei, F 2014b. Complete mitochondrial genome sequence of *Bonasa sewerzowi* (Galliformes: Phasianidae) and phylogenetic analysis. Zoological Systematics, 39: 359–371.

Li, X, Huang, Y & Lei, F 2015a. Comparative mitochondrial genomics and phylogenetic relationships of the *Crossoptilon* species (Phasianidae, Galliformes). BMC Genomics 16(1), 42.

Li, X, Huang, Y & Lei, F 2016c. Complete mitochondrial genome of the Yellow-bellied Tit *Parus venustulus* (Passeriformes, Paridae). Mitochondrial DNA Part A 27: 3216-3217.

Liu, B, Sun, CH, Wang, LB, Xue, DD, Xu, P, Xie, SB & Lu, CH 2019. The complete mitochondrial genome of Grey-streaked Flycatcher *Muscicapa griseisticta* (Passeriformes: Muscicapidae). Mitochondrial DNA Part B 4: 1857-1858.

Ma, Y-G, Huang, Y & Lei, FM 2014. Sequencing and phylogenetic analysis of the *Pyrgilauda ruficollis* (Aves, Passeridae) complete mitochondrial genome. Zoological Research 35: 81-91.

Marshall, HD, Baker, AJ & Grant, AR 2013. Complete mitochondrial genomes from four subspecies of common chaffinch (*Fringilla coelebs*): New inferences about mitochondrial rate heterogeneity, neutral theory, and phylogenetic relationships within the order Passeriformes. Gene 517: 37-45.

Peng, LF, Yang, DC & Lu, CH 2016b. Complete mitochondrial genome of oriental magpie-robin *Copsychus saularis* (Aves: Muscicapidae). Mitochondrial DNA Part B 1: 21-22.

Peng, LF, Yang, DC & Lu, CH 2016c. Complete mitochondrial genome sequence of Eurasian blackbird, *Turdus merula* (Aves: Turdidae). Mitochondrial DNA Part A 27: 4609-4610.

Wang, X, Huang, Y, Liu, N, Yang, J & Lei, F 2015a. Seven complete mitochondrial genome sequences of bushtits (Passeriformes, Aegithalidae, Aegithalos): the evolution pattern in duplicated control regions. Mitochondrial DNA 26: 350-356.

Wen, L & Liao, F 2016. Complete mitochondrial genome of *Pycnonotus xanthorrhous* (Passeriformes, Pycnonotidae) and phylogenetic consideration. Biochemical Systematics and Ecology 69: 83-90.

Wenzel, MA, Webster, LMI, Blanco, G, Burgess, MD, Kerbiriou, C, Segelbacher, G, Piertney, SB & Reid, JM 2012. Pronounced genetic structure and low genetic diversity in European red-billed chough (*Pyrrhocorax pyrrhocorax*) populations. Conserv. Gen. 13: 1213-1230.

Xin, C, Li, S, Zhang, Z, Wang, X & Huang, Y 2016. The complete mitochondrial genome of *Pseudopodoces humilis* (Passeriformes, Paridae). Mitochondrial DNA Part A 27: 4017-4018.

Xu, Q, Xia, Y, Dang, X & Chen, X 2016a. The complete mitochondrial genome sequence of Emperor Penguins (*Aptenodytes forsteri*). Mitochondrial DNA Part A 27: 3646-3647.

Yang, C, Wang, QX, Huang, Y & Xiao, H 2012. Analysis of the complete mitochondrial genome sequence of *Larus brunnicephalus* (Aves, Laridae). Yi chuan = Hereditas (Beijing) 34(11): 1434-1446.

Yang, C, Yang, M, Wang, Q, Lu, Y & Li, X 2018. The complete mitogenome of *Falco amurensis* (Falconiformes, Falconidae), and a comparative analysis of genus *Falco*. Zoological Science 35: 367-373.

Yang, C, Lei, FM & Huang, Y 2010. Sequencing and analysis of the complete mitochondrial genome of *Pseudopodoces humilis* (Aves, Paridae). Dongwuxue Yanjiu (Zool. Res.) 31: 333–344. (In Chinese.)

Zhang, H, Bai, Y, Shi, X, Sun, L, Wang, Z & Wu, X 2018b. The complete mitochondrial genomes of *Tarsiger cyanurus* and *Phoenicurus auroreus*: a phylogenetic analysis of Passeriformes. Genes & Genomics 40: 151-165.

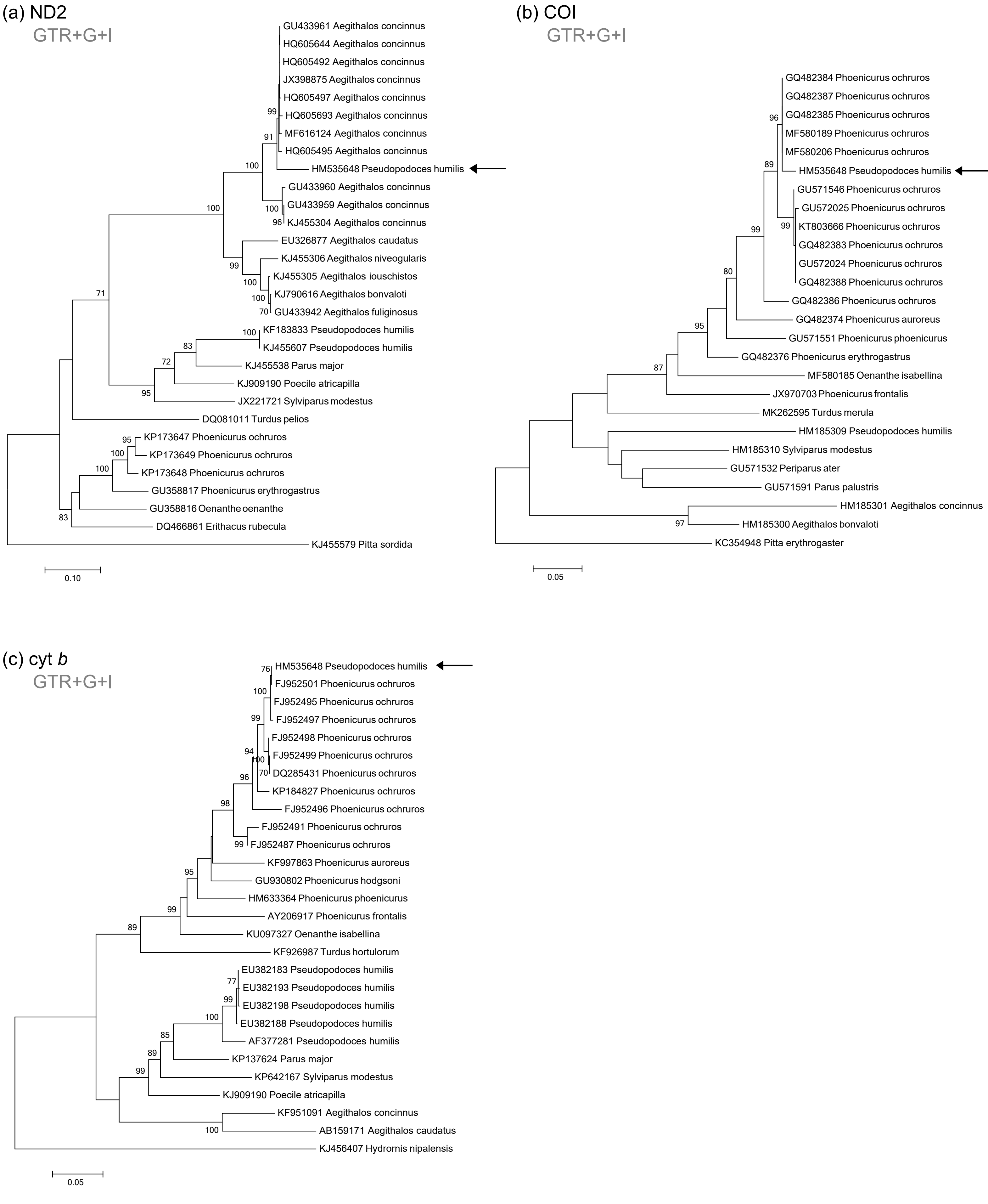

Figure S49. Maximum Likelihood phylogenies of *Pseudopodoces humilis* (HM535648) and related taxa based on mitochondrial sequences. Numbers at branches are bootstrap support values (>70%) based on 1000 replicates.

60. “*Hirundo rustica gutturalis*” KP148840 (Liu, J., Liu, S., Shao, C., Zhang, Y., Xie, Y., Tang, Q., Shen, Y., Xie, J., unpubl.; 2014)

Fig. S50

Metadata: No data on GenBank.
Phylogenetic position:
ND2 = divergent from all sequences of *Hirundo rustica*
CO1 = sister to, and divergent from, all sequences of *Hirundo rustica*
Cyt *b* = sister to all sequences of *Hirundo*
Sequence integrity:
ND2: differs (on average) by 4.8% from other *H. rustica.* There were 38 substitutions that are unique among *H. rustica* (n=40); these are found across the entire gene.
CO1: differs (on average) by 3.7% from other *H. rustica.* There were 18 substitutions that are unique among *H. rustica*; these are found across most of the barcoding part of the COI gene.
cyt b: differs (on average) by 4.3% from other *H. rustica* (uncorrected p-distance, with complete deletion of missing sites).
Problems noted in other works: None.
Interpretation: sequencing errors / numts
Subsequent usage:
Re-used in **4 mitogenomic phylogenies published before 1 January 2020**: Huang & Zeng 2016a, Nabholz et al. 2016, Wen et al. 2017c, Mackiewicz et al. 2019.
Relevance: This was the only mitogenome presumed to be of this subspecies (January 2020)
References:

Huang, L & Zeng, B 2016a. Complete mitochondrial genome of the Chinese hwamei (*Garrulax canorus*). Mitochondrial DNA Part A 27: 4559-4560.

Mackiewicz, P, Urantówka, AD, Kroczak, A & Mackiewicz, D 2019. Resolving phylogenetic relationships within Passeriformes based on mitochondrial genes and inferring the evolution of their mitogenomes in terms of duplications. Genome Biology and Evolution 11: 2824-2849.

Nabholz, B, Lanfear, R & Fuchs, J 2016. Body mass-corrected molecular rate for bird mitochondrial DNA. Mol. Ecol. 25: 4438-4449.

Wen, L, Wang, Y, Fu, Y & Dai, B 2017c. The complete mitochondrial genome of the white-browed laughingthrush *Garrulax sannio* (Passeriformes: Leiothrichidae). Mitochondrial DNA Part A 28: 23-24.

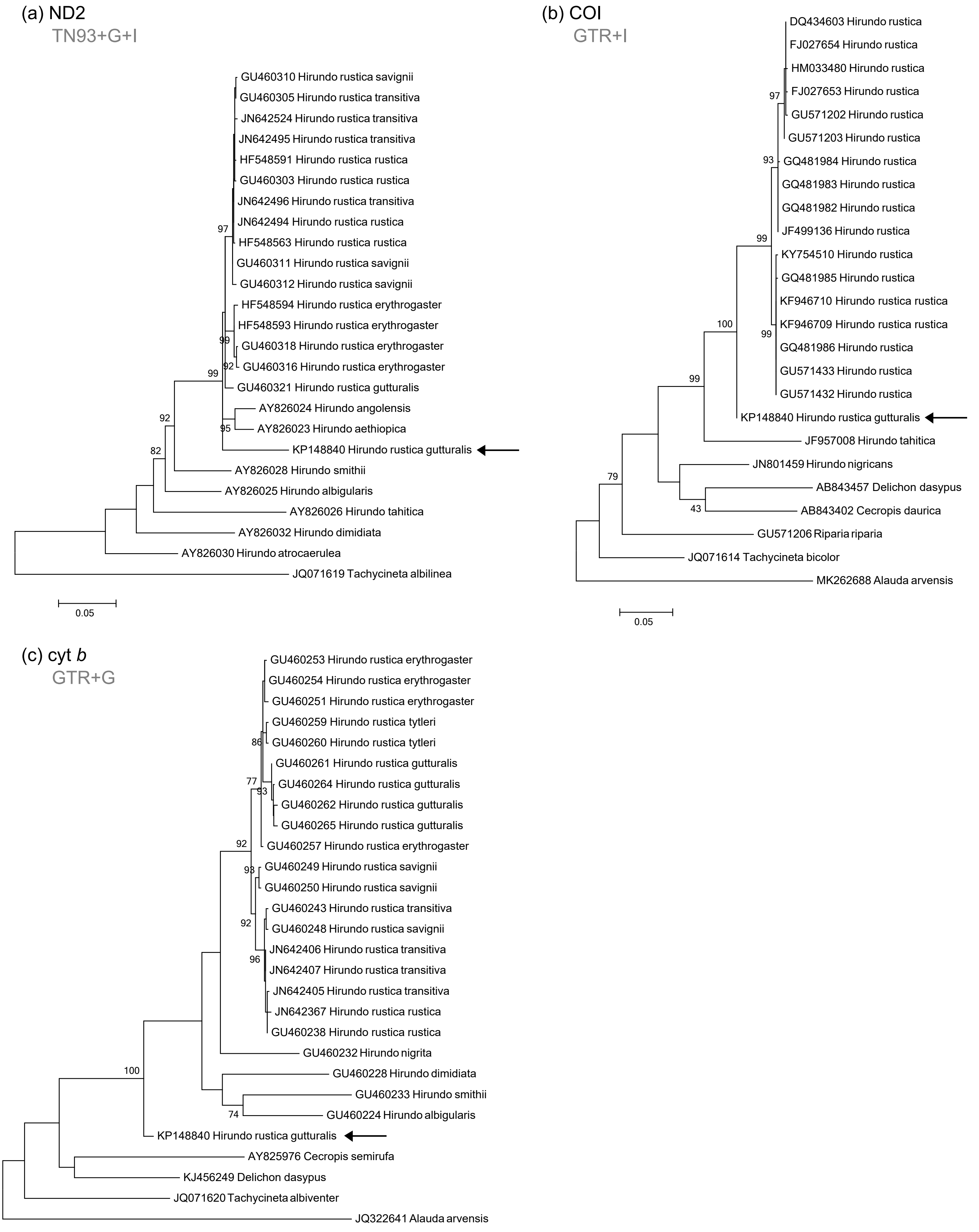

Figure S50. Maximum Likelihood phylogenies of *Hirundo rustica gutturalis* (KP148840) and related taxa based on mitochondrial sequences. Numbers at branches are bootstrap support values (>70%) based on 1000 replicates.

61. “*Seicercus burkii*” KX977449 (Zhang, W., Yue, B. and Jing, J., unpublished; 2019)

Fig. S51

Metadata: No data on GenBank.
Phylogenetic position:
ND2 = could not be verified due to lack of sequences of *Seicercus burkii.*
CO1 = *Seicercus affinis*
Cyt *b* = *Seicercus affinis*
Sequence integrity: not investigated
Interpretation: misidentification
Subsequent usage: not used in any further studies (January 2020)
Problems noted in other works: none
Relevance: This was the only mitogenome presumed to be of this species (January 2020)

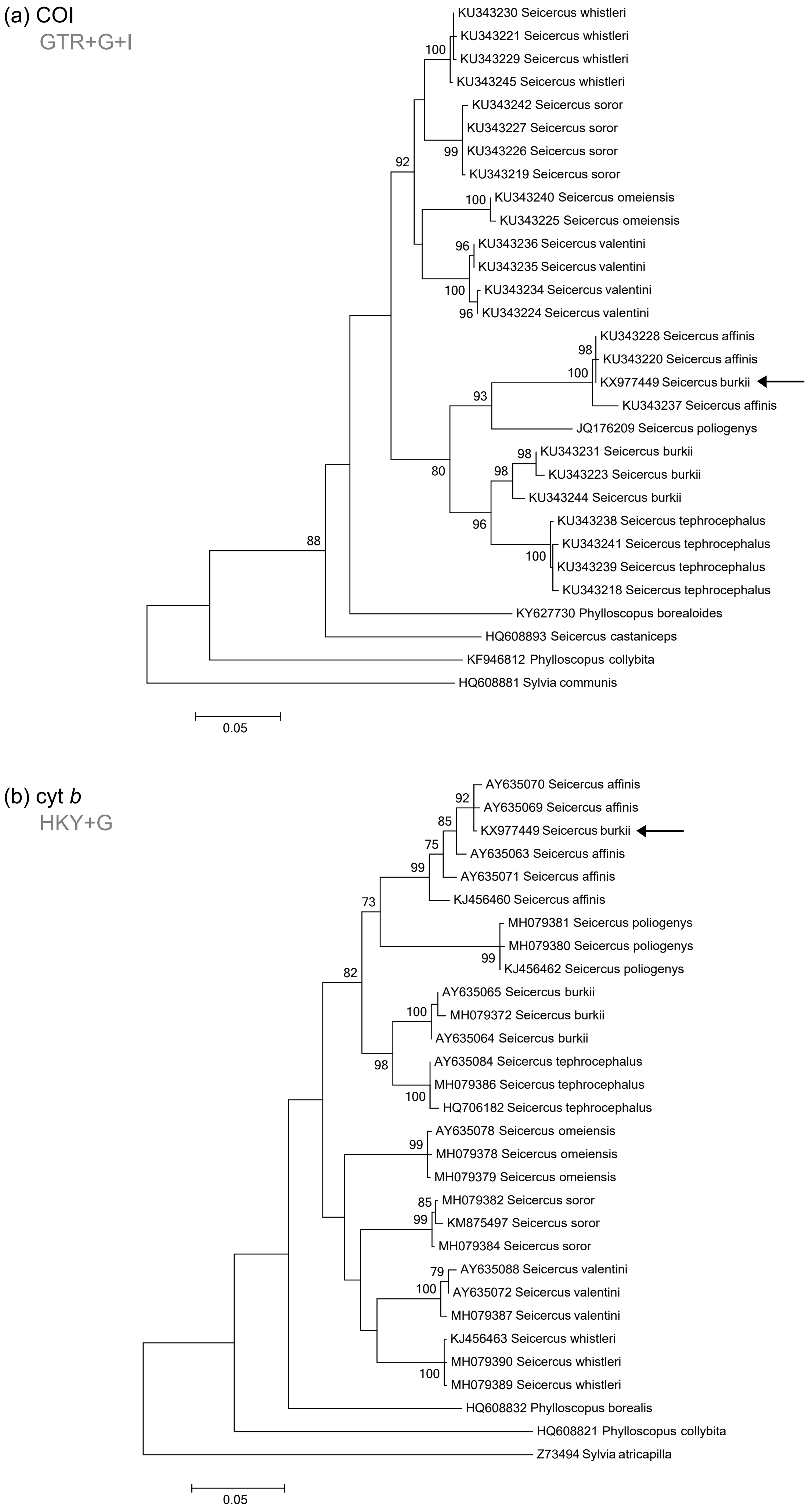

Figure S51. Maximum Likelihood phylogenies of *Seicercus burkii* (KX977449) and related taxa based on mitochondrial sequences. Numbers at branches are bootstrap support values (>70%) based on 1000 replicates.

62. “*Garrulax albogularis*” KX082660, NC_037464 (Liu et al. 2018a)

Fig. S52

Metadata: “The muscle sample of *G. albogularis* was collected from Ya’an, Sichuan province of China (N30anced E102ancedi The samples were then stored at the Wildlife Conservation Laboratory at Sichuan Agricultural University, Sichuan province, China.” (Liu et al. 2018a: 1272).

Phylogenetic position:

ND2 = *Garrulax albogularis*

CO1 = *Garrulax albogularis*

Cyt *b* = sister to *Minla ignotincta*

Sequence integrity: cyt b: first part (bp 1 – circa 504) = *Garrulax albogularis*; second part (circa bp 522 – 1143) = *Minla ignotincta*

Interpretation: chimera

Subsequent usage:

Re-used in **2 mitogenomic phylogenies published before 1 January 2020**: He et al. 2019c, Jiang et al. 2019a.

Problems noted in other works: None. Jiang et al. (2019: 1426) commented that “It was delusory that *Garrulax albogularis* did not cluster to *Garrulax* but cluster with *Minla ignotincta*” but did not further investigate this.

Relevance: This was the only mitogenome presumed to be of this species (January 2020)

References:

He, W, Xu, H, Li, D, Xie, M, Zhang, M, Ni, Q & Yao, Y 2019c. The complete mitochondrial genome of Blue-winged Minla (*Minla cyanouroptera*) and its phylogenetic analysis. Mitochondrial DNA Part B 4: 2784-2785.

Jiang, X, Gao, J, Zhou, C, Jing, J, Chen, M, Yue, B & Zhang, X 2019a. The complete mitochondrial genome of brown-flanked bush warbler (*Horornis fortipes*). Mitochondrial DNA Part B 4: 1426-1427.

Liu, X, Xu, H, Zhou, Y, Li, D, Ni, Q, Zhang, M, Xie, M, Wen, A, Wang, Q, Wu, J & & Yao, Y 2018a. Complete characteristics and phylogenetic relationships of the *Garrulax albogularis* mitochondrial genome (Passeriformes: Timaliidae). Mitochondrial DNA Part B 3: 1272-1273.

63. “*Garrulax poecilorhynchus*” KR909134, NC_028082 (Qi et al. 2016b)

Fig. S52

Metadata: “The specimen (code B33) was collected from Ya’an, Sichuan Province, China.” (Qi et al. 2016: 3636). GenBank: sex="male", country="China", collection_date="2014", collected_by="Huailiang Xu".

Phylogenetic position:

ND2 = *Leiothrix lutea*

CO1 = *Garrulax poecilorhynchus*

Cyt *b* = *Garrulax poecilorhynchus*

Sequence integrity: not investigated

Interpretation: chimera

Subsequent usage:

Re-used in **4 mitogenomic phylogenies published before 1 January 2020**: Duan et al. 2019, Huang et al. 2019a, Zhao et al. 2019, Mackiewicz et al. 2019.

Problems noted in other works: None.

Relevance: This was the only mitogenome presumed to be of this species (January 2020).

References:

Duan, Y, Li, Y & Luo, X 2019. Next-generation sequencing yields the mitochondrial genome of black-streaked Scimitar Babbler *Pomatorhinus gravivox* (Passeriformes: Timaliidae). Mitochondrial DNA Part B 4: 998-999.

Huang, Z, Tu, F & Tang, S 2019a. Two new mitogenomes of Pellorneidae (Aves: Passeriformes) and a phylogeny of the superfamily Sylvioidea. Australian Journal of Zoology 66:167-173.

Mackiewicz, P, Urantówka, AD, Kroczak, A & Mackiewicz, D 2019. Resolving phylogenetic relationships within Passeriformes based on mitochondrial genes and inferring the evolution of their mitogenomes in terms of duplications. Genome Biology and Evolution 11: 2824-2849.

Qi, Y, Zhou, YY, Yao, YF, Huan, ZJ, Li, DY, Xie, M, Ni, QY, Zhang, MW & Xu, HL 2016b. The complete mitochondrial genome sequence of *Garrulax poecilorhynchus* (Aves, Passeriformes, Timaliidae). Mitochondrial DNA Part A 27: 3636-3637.

Zhao, Q, Xu, HL & Yao, YF 2019. The complete mitochondrial genome and phylogeny of the Emei Shan liocichla (*Liocichla omeiensis*). Conservation Genetics Resources 11: 303–307.

64. "*Garrulax perspicillatus*" KF997865, NC_026068 (Zhang et al. 2016d)

Fig. S52

Metadata: no data in Zhang et al. (2016d) or GenBank
Phylogenetic position:
ND2 = among two *G. pectoralis*; no reference sequence of *G. perspicillatus* available
CO1 = close sister to *G. pectoralis*; no reference sequence of *G. perspicillatus* available
Cyt *b* = *G. pectoralis*
Sequence integrity: not investigated
Interpretation: misidentification
Subsequent usage:
Re-used in **21 mitogenomic phylogenies published before 1 January 2020**: Huang & Zeng 2016a, Li et al. 2016f, Huan et al. 2016, Qi et al. 2016a, Qi et al. 2016b, Zhou et al. 2016a, Zhou et al. 2016c, Huang et al. 2016a, Nabholz et al. 2016, Li et al. 2017a, Wen et al. 2017c, Liu et al. 2018a, Zhang et al. 2018a, Zhang et al. 2018b, Caparroz et al. 2018, Zhao et al. 2019, Huang et al. 2019a, He et al. 2019c, Huang et al. 2019d, Jiang et al. 2019a, Mackiewicz et al. 2019.
Problems noted in other works: None.
Relevance: This was the only mitogenome presumed to be of this species (January 2020).
References:

Caparroz, R, Rocha, AV, Cabanne, GS, Tubaro, P, Aleixo, A, Lemmon, EM & Lemmon, AR 2018. Mitogenomes of two neotropical bird species and the multiple independent origin of mitochondrial gene orders in Passeriformes. Molecular Biology Reports 45: 279-285.

He, W, Xu, H, Li, D, Xie, M, Zhang, M, Ni, Q & Yao, Y 2019c. The complete mitochondrial genome of Blue-winged Minla (*Minla cyanouroptera*) and its phylogenetic analysis. Mitochondrial DNA Part B 4: 2784-2785.

Huan, Z, Yao, Y, Zhou, Y, Qi, Y, Wang, Q, Li, D, Xie, M, Ni, Q, Zhang, M & Xu, H 2016. Complete mitochondrial genome sequence of *Garrulax formosus* (Aves, Passeriformes, Timaliidae) and its phylogenetic analysis. Mitochondrial DNA Part A 27: 2858-2859.

Huang, J, Zhou, C, Wang, L, Jiang, X, Zhang, X, Yue, B & Meng, Y 2019d. The complete mitochondrial genome of the *Minla cyanouroptera* (Passeriformes: Timaliidae). Mitochondrial DNA Part B 4: 3610-3611.

Huang, L & Zeng, B 2016a. Complete mitochondrial genome of the Chinese hwamei (*Garrulax canorus*). Mitochondrial DNA Part A 27: 4559-4560.

Huang, R, Zhou, Y, Yao, Y, Zhao, B, Zhang, Y & Xu, HL 2016a. Complete mitochondrial genome and phylogenetic relationship analysis of *Garrulax affinis* (Passeriformes, Timaliidae). Mitochondrial DNA Part A 27: 3502-3503.

Huang, Z, Tu, F & Tang, S 2019a. Two new mitogenomes of Pellorneidae (Aves: Passeriformes) and a phylogeny of the superfamily Sylvioidea. Australian Journal of Zoology 66:167-173.

Jiang, X, Gao, J, Zhou, C, Jing, J, Chen, M, Yue, B & Zhang, X 2019a. The complete mitochondrial genome of brown-flanked bush warbler (*Horornis fortipes*). Mitochondrial DNA Part B 4: 1426-1427.

Li, B, Yao, Y, Li, D, Ni, Q, Zhang, M, Xie, M & Xu, H 2016f. Complete mitochondrial genome of *Minla ignotincta* (Passeriformes: Timaliidae). Mitochondrial DNA Part B 1: 140-141.

Li, B, Yao, Y, Li, D, Ni, Q, Zhang, M, Xie, M & Xu, H 2017a. The complete mitochondrial genome sequence of White-collared Yuhina (*Yuhina diademata*). Mitochondrial DNA Part A 28: 21-22.

Liu, X, Xu, H, Zhou, Y, Li, D, Ni, Q, Zhang, M, Xie, M, Wen, A, Wang, Q, Wu, J & & Yao, Y 2018a. Complete characteristics and phylogenetic relationships of the *Garrulax albogularis* mitochondrial genome (Passeriformes: Timaliidae). Mitochondrial DNA Part B 3: 1272-1273.

Mackiewicz, P, Urantówka, AD, Kroczak, A & Mackiewicz, D 2019. Resolving phylogenetic relationships within Passeriformes based on mitochondrial genes and inferring the evolution of their mitogenomes in terms of duplications. Genome Biology and Evolution 11: 2824-2849.

Nabholz, B, Lanfear, R & Fuchs, J 2016. Body mass-corrected molecular rate for bird mitochondrial DNA. Mol. Ecol. 25: 4438-4449.

Qi, Y, Zhou, YY, Yao, YF, Huan, ZJ, Li, DY, Xie, M, Ni, QY, Zhang, MW & Xu, HL 2016a. The complete mitochondrial genome of *Babax lanceolatus* (Passeriformes: Timaliidae). Mitochondrial DNA Part A 27: 2925-2926.

Qi, Y, Zhou, YY, Yao, YF, Huan, ZJ, Li, DY, Xie, M, Ni, QY, Zhang, MW & Xu, HL 2016b. The complete mitochondrial genome sequence of *Garrulax poecilorhynchus* (Aves, Passeriformes, Timaliidae). Mitochondrial DNA Part A 27: 3636-3637.

Wen, L, Wang, Y, Fu, Y & Dai, B 2017c. The complete mitochondrial genome of the white-browed laughingthrush *Garrulax sannio* (Passeriformes: Leiothrichidae). Mitochondrial DNA Part A 28: 23-24.

Zhang, H, Bai, Y, Shi, X, Sun, L, Wang, Z & Wu, X 2018b. The complete mitochondrial genomes of *Tarsiger cyanurus* and *Phoenicurus auroreus*: a phylogenetic analysis of Passeriformes. Genes & Genomics 40: 151-165.

Zhang, H, Li, Y, Wu, X, Xue, H, Yan, P & Wu, XB 2016d. The complete mitochondrial genome of *Garrulax perspicillatus* (Passeriformes, Timaliidae). Mitochondrial DNA Part A 27: 1265-1266.

Zhang, L, Xu, D, Xia, T, Yang, X, Sun, G, Wei, Q, Sha, W & Zhang, H. 2018a. The complete mitochondrial genome of red-tailed laughingthrush (*Garrulax milnei*). Mitochondrial DNA Part B 3: 953-954.

Zhao, Q, Xu, HL & Yao, YF 2019. The complete mitochondrial genome and phylogeny of the Emei Shan liocichla (*Liocichla omeiensis*). Conservation Genetics Resources 11: 303–307.

Zhou, Y, Qi, Y, Xu, H, Huan, Z, Li, D, Xie, M, Ni, Q, Zhang, M & Yao, Y 2016c. The complete mitochondrial genome sequence of *Garrulax ocellatus* (Aves, Passeriformes, Timaliidae). Mitochondrial DNA Part A 27: 2689-2690.

Zhou, YY, Qi, Y, Yao, YF, Huan, ZJ, Li, DY, Xie, M, Ni, QY, Zhang, MW & Xu, HL 2016a. Characteristic of complete mitochondrial genome and phylogenetic relationship of *Garrulax sannio* (Passeriformes, Timaliidae). Mitochondrial DNA Part A 27: 2947-2948.

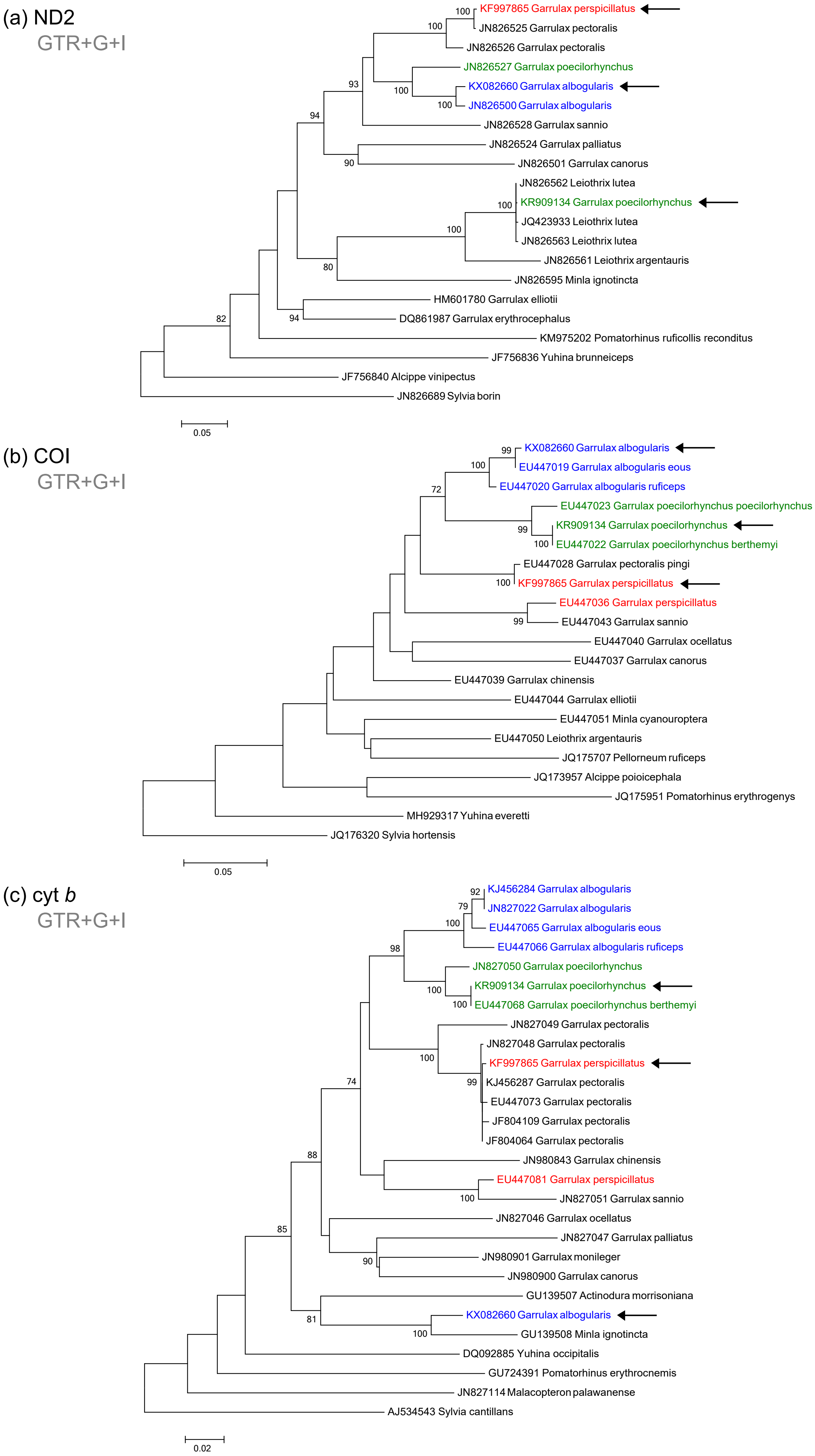

Figure S52. Maximum Likelihood phylogenies of *Garrulax albogularis* (KX082660), *G. poecilorhynchus* (KR909134), *G. perspicillatus* (KF997865) and related taxa based on mitochondrial sequences. Numbers at branches are bootstrap support values (>70%) based on 1000 replicates.

65. "*Trochalopteron milnei*" MH238447, NC_041141 (Zhang et al. 2018a)

Fig. S53

Metadata: "In this study, the muscle sample of *G. milnei* was collected through the field investigation in Guangzhou province, China, and the geo-spatial coordinates are 23º09'0 52''N latitude, and 1133º'30'40''E longitude. The samples are deposited in the Herbarium of the Institute of Protection and Utilization for Biological Resource at Qufu Normal University in Shandong province, China. The sample died naturally …" (Zhang et al. 2018a: 953).

Phylogenetic position:

ND2 = *Trochalopteron/Garrulax milnei*

CO1 = *Trochalopteron/Garrulax milnei*

Cyt *b* = *Trochalopteron/Garrulax milnei* but on a long branch

Sequence integrity: cyt *b*: bp 1-159 contain 24 nucleotides that were not found in two sequences of *T. milnei* (sequence divergence 15%; uncorrected p-value, with complete deletion of missing sites). Bp 22-159 are identical to sequences of *Abroscopus schisticeps*. The first 21 bp of cyt *b* are not present in sequences of *Abroscopus schisticeps* but these positions were identical to sequences of two other species of *Abroscopus.*

Interpretation: chimera

Subsequent usage:

Re-used in **1 mitogenomic phylogeny published before 1 January 2020**: Liu et al. 2019m.

Problems noted in other works: none.

Relevance: This was the only mitogenome presumed to be of this species (January 2020).

References:

Liu, Z, Liu, J, Chen, X, Jin, Z, Luo, A & Li, S 2019m. Phylogenetic relationship and characterization of the complete chloroplast genome of the Alpine Leaf-warbler in Qinghai-Tibet Plateau. Mitochondrial DNA Part B 4: 3582-3584.

Zhang, L, Xu, D, Xia, T, Yang, X, Sun, G, Wei, Q, Sha, W & Zhang, H. 2018a. The complete mitochondrial genome of red-tailed laughingthrush (*Garrulax milnei*). Mitochondrial DNA Part B 3: 953-954.

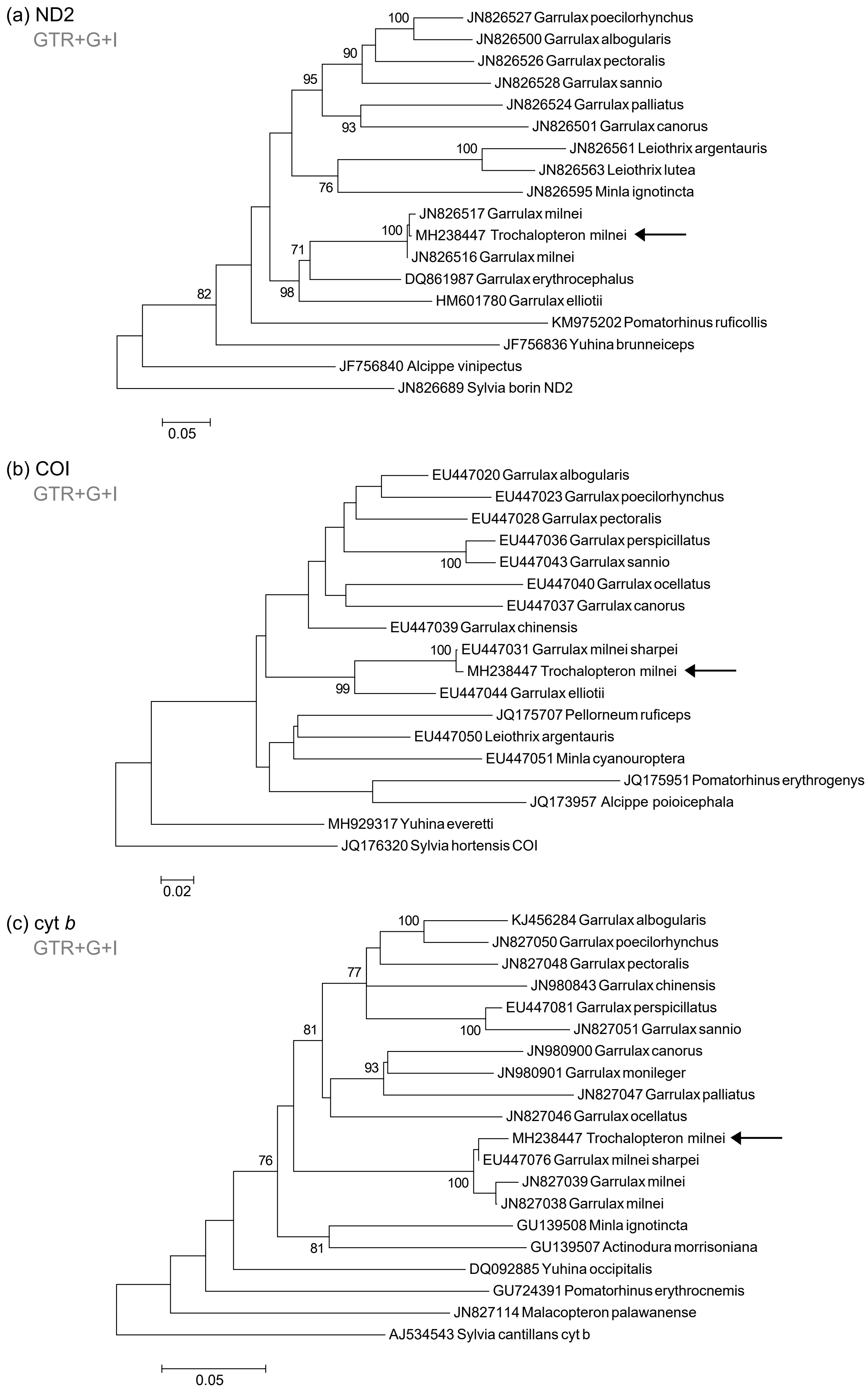

Figure S53. Maximum Likelihood phylogenies of *Garrulax* (*Trochalopteron*) *milnei* (MH238447) and related taxa based on mitochondrial sequences. Numbers at branches are bootstrap support values (>70%) based on 1000 replicates.

66. "*Sturnus nigricollis*" JQ003192 (Kan, X.-Z. and Qian, C.-J., unpublished; 2013)

Fig. S54

Metadata: information on GenBank was limited to: "/specimen_voucher="AHNU:A0036"
Phylogenetic position:
ND2 = *Sturnus nigricollis*
CO1 = sister to, and divergent from, two *Sturnus nigricollis* (JQ003191, JQ174953)
Cyt *b* = identical to another mitogenome of *Sturnus nigricollis*
Sequence integrity:
COI: bp 1-217 were identical to *Acridotheres cristatellus* (BLAST 100% match) and showed 16 singleton sites (not found in two *Sturnus nigricollis sequences* JQ003191, JQ174953)
Interpretation: chimera
Subsequent usage:
Re-used in **3 mitogenomic mitogenomic phylogenies published before 1 January 2020**: Wen et al. 2017a, Wen et al. 2017b, Mackiewicz et al. 2019.
Used in **2 other papers**: Husemann et al. 2016, Meimberg et al. 2016.
Problems noted in other works: none
Relevance: Another mitogenome of this species (JQ003191) was placed on GenBank by the same authors (January 2020).
References:

Husemann, M, Sturm, S, Curto, M, Meimberg, H & Habel, JC 2016. Four new mitochondrial genomes of the genus *Zosterops* (aves: passeriformes: zosteropidae) from East Africa with a phylogenetic evaluation of the group. Mitochondrial DNA Part B 1: 544-548.

Mackiewicz, P, Urantówka, AD, Kroczak, A & Mackiewicz, D 2019. Resolving phylogenetic relationships within Passeriformes based on mitochondrial genes and inferring the evolution of their mitogenomes in terms of duplications. Genome Biology and Evolution 11: 2824-2849.

Meimberg, H, Schachtler, C, Curto, M, Husemann, M & Habel, JC 2016. A new amplicon based approach of whole mitogenome sequencing for phylogenetic and phylogeographic analysis: an example of East African white-eyes (Aves, Zosteropidae). Molecular Phylogenetics and Evolution 102: 74–85.

Wen, L, Yang, X, Liao, J, Fu, Y & Dai, B 2017a. The complete mitochondrial genome of the Fulvous Parrotbill *Paradoxornis fulvifrons* (Passeriformes: Muscicapidae). Mitochondrial DNA Part A 28: 143-144.

Wen, L, Fu, Y, Dai, B & Liao, J 2017b. The complete mitochondrial genome of the Green-Backed tit *Parus monticolus* (Passeriformes: Paridae). Mitochondrial DNA Part A 28: 79-80.

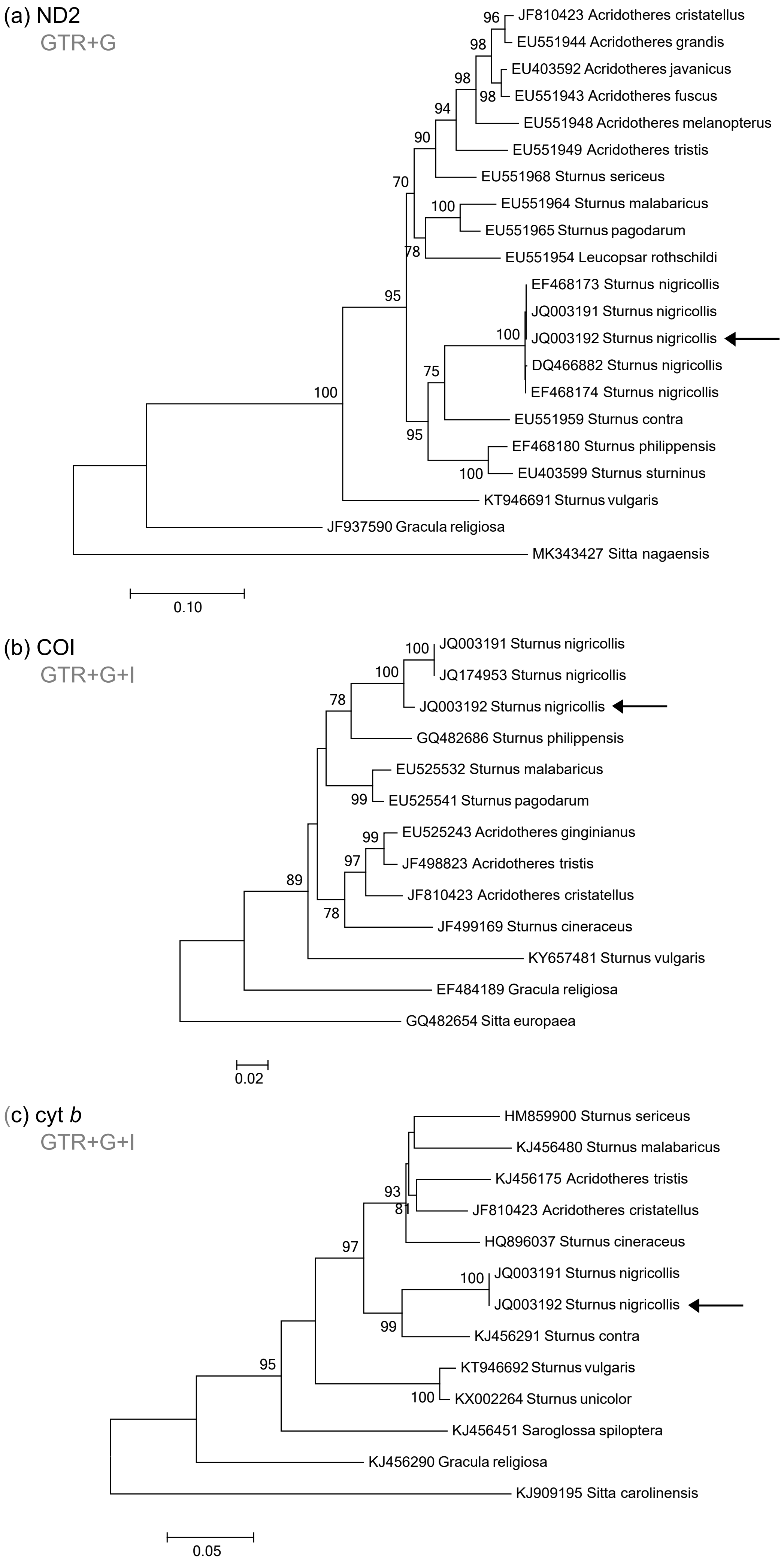

Figure S54. Maximum Likelihood phylogenies of *Sturnus nigricollis* (JQ003192) and related taxa based on mitochondrial sequences. Numbers at branches are bootstrap support values (>70%) based on 1000 replicates.

67. "*Turdus merula*" KT373849, NC_028188 (Wen, L., Fu, Y., Dai, B. and Liao, J., unpublished; 2015)

Fig. S55

Metadata: GenBank data: specimen_voucher="TMER20150704V3"; country="China"
Phylogenetic position:
ND2 = distant from *T. merula*; no reference sequences of *T. mandarinus* available
CO1 = distant from *T. merula*; no reference sequences of *T. mandarinus* available
Cyt *b* = *T. mandarinus*
Sequence integrity: not investigated
Interpretation: misidentification due to outdated taxonomy. *T. mandarinus* was formerly included in *T. merula* but was split based on its distant phylogenetic position (Dickinson & Christidis 2014).
Subsequent usage:
Re-used in **4 mitogenomic phylogenies published before 1 January 2020**: Song et al. 2018, Mackiewicz et al. 2019, Park et al. 2019d, Zhou et al. 2019b.
Re-used in **3 additional non-mitogenome phylogenies**: Lu et al. 2019, Min et al. 2019, Sun et al. 2019a.
Problems noted in other works: none.
Relevance: Another mitogenome presumed of this species (KT601060) was also a *T. mandarinus*.
References:

Lu, CH, Sun, CH, Hou, SL, Huang, YL & Lu, CH 2019. The complete mitochondrial genome of dark-sided flycatcher *Muscicapa sibirica* (Passeriformes: Muscicapidae). Mitochondrial DNA Part B 4: 2675-2676.
Mackiewicz, P, Urantówka, AD, Kroczak, A & Mackiewicz, D 2019. Resolving phylogenetic relationships within Passeriformes based on mitochondrial genes and inferring the evolution of their mitogenomes in terms of duplications. Genome Biology and Evolution 11: 2824-2849.
Min, X, Lu, CH, Chen, TY, Liu, B & Lu, CH 2019. The complete mitochondrial genome of Asian brown flycatcher *Muscicapa latirostris* (Passeriformes: Muscicapidae). Mitochondrial DNA Part B 4: 3880-3881.
Park, CE, Kim, MC, Quang, HP, Park, HC & Shin, JH 2019d. The complete mitochondrial genome sequence of *Zoothera aurea* (Aves, Passeriformes, Turdidae). Mitochondrial DNA Part B 4: 797-798.
Song, S, Qin, J, Luo, J, Li, D, Jiang, B & Chang, C 2018. Analysis of complete mitochondrial genome sequence of Kessleri [sic] Thrush, Turdus kessleri (Passeriformes, Turdidae). Mitochondrial DNA Part B 3: 818-819.
Sun, CH, Liu, B & Lu, CH 2019a Complete mitochondrial genome of the Siberian Thrush, Geokichla sibirica sibirica (Aves, Turdidae). Mitochondrial DNA Part B 4: 1150-1151.
Zhou, C, Jin, J, Chen, Y, Hao, Y, Zhang, X, Meng, Y, Yang, N & Yue, B 2019b. Two new complete mitochondrial genomes (*Paradoxornis gularis* and *Niltava davidi*) and their phylogenetic and taxonomic implications. Mitochondrial DNA Part B 4: 820-821.

68. “*Turdus merula*” KT601060 (Peng et al. 2016c)

Fig. S55

Metadata:
Phylogenetic position:
ND2 = distant from *T. merula*; no reference sequences of *T. mandarinus* available
CO1 = distant from *T. merula*; no reference sequences of *T. mandarinus* available
Cyt *b* = *T. mandarinus*
Sequence integrity: not investigated
Interpretation: misidentification due to outdated taxonomy. *T. mandarinus* was formerly included in *T. merula* but was split based on its distant phylogenetic position (Dickinson & Christidis 2014).
Subsequent usage:
Re-used in **one mitogenomic phylogeny published before 1 January 2020**: Peng et al. 2016b.
Problems noted in other works: none.
Relevance: Another mitogenome presumed of this species (KT373849, NC_028188) was also a *T. mandarinus*.
References:
Peng, LF, Yang, DC & Lu, CH 2016b. Complete mitochondrial genome of oriental magpie-robin *Copsychus saularis* (Aves: Muscicapidae). Mitochondrial DNA Part B 1: 21-22.
Peng, LF, Yang, DC & Lu, CH 2016c. Complete mitochondrial genome sequence of Eurasian blackbird, *Turdus merula* (Aves: Turdidae). Mitochondrial DNA Part A 27: 4609-4610.

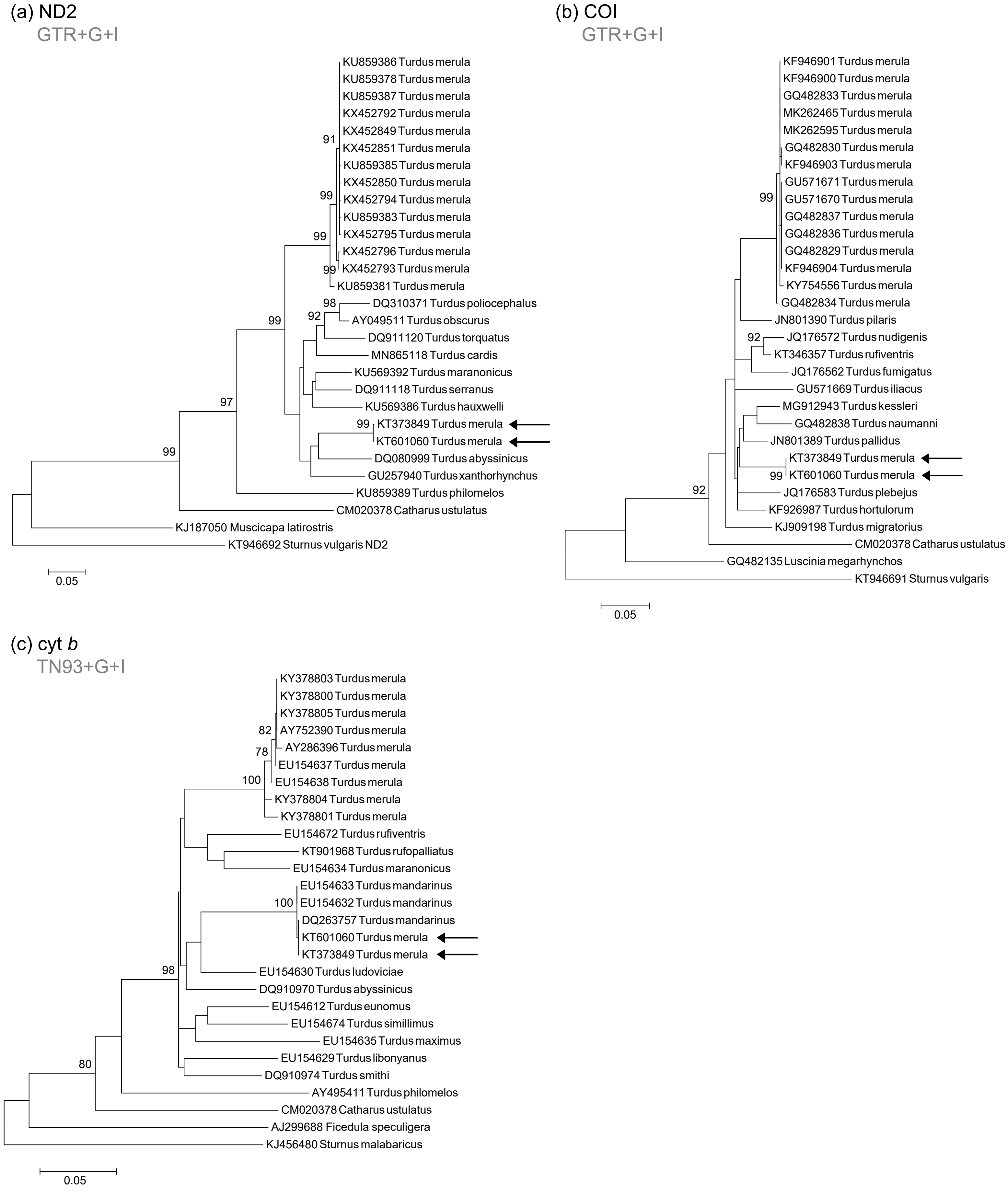

Figure S55. Maximum Likelihood phylogenies of *Turdus merula* (KT373849 and KT601060) and related taxa based on mitochondrial sequences. Numbers at branches are bootstrap support values (>70%) based on 1000 replicates.

69. “*Muscicapa griseisticta*” MK390479, NC_045181 (Liu et al. 2019g)

Fig. S56

Metadata: “The sample of *M. griseisticta* was collected from Yancheng City, Jiangsu Province, China.” (Liu et al. 2019g: 1857)
Phylogenetic position:
ND2 = *Muscicapa sibirica*
CO1 = *Muscicapa sibirica*
Cyt *b* = *Muscicapa sibirica*
Sequence integrity: not investigated
Interpretation: misidentification
Subsequent usage: not used in any further studies (January 2020)
Problems noted in other works: none
Relevance: This was the only mitogenome presumed to be of this species (January 2020).
References:
Liu, B, Sun, CH, Wang, LB, Xue, DD, Xu, P, Xie, SB & Lu, CH 2019g. The complete mitochondrial genome of Grey-streaked Flycatcher *Muscicapa griseisticta* (Passeriformes: Muscicapidae). Mitochondrial DNA Part B 4: 1857-1858.

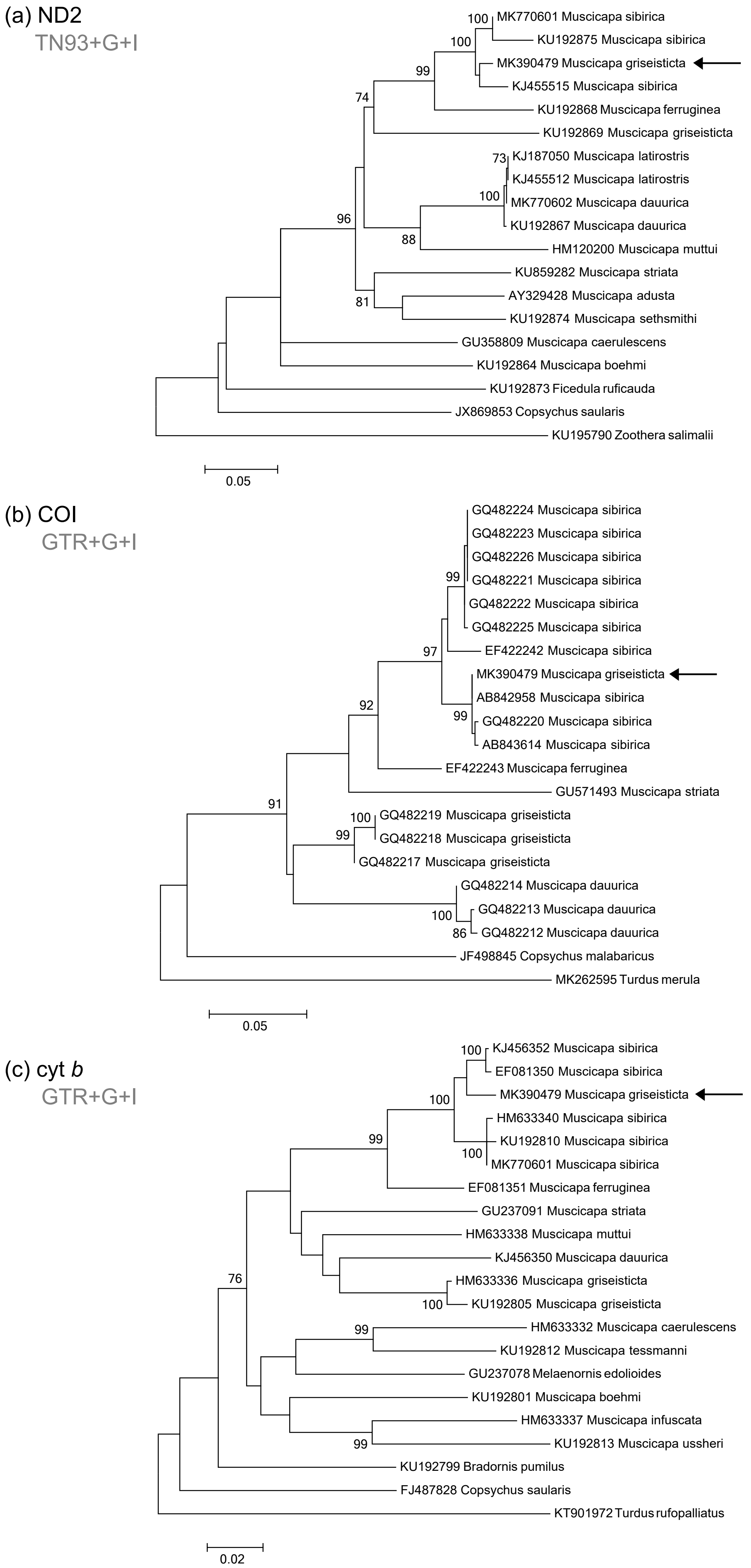

Figure S56. Maximum Likelihood phylogenies of *Muscicapa griseisticta* (MK390479) and related taxa based on mitochondrial sequences. Numbers at branches are bootstrap support values (>70%) based on 1000 replicates.

70. "*Cyanoptila cyanomelana*" HQ896033, NC_015232 (Kan,X.-Z. and Li,X.-F., unpublished; 2011)

Fig. S57

Metadata: no data on GenBank
Phylogenetic position:
ND2 = in clade among *Cyornis hainanus* and *Cyornis rubeculoides*
CO1 = in clade among *Cyornis hainanus* and *Cyornis rubeculoides*
Cyt *b* = in clade among *Cyornis hainanus* and *Cyornis rubeculoides*
Sequence integrity: not investigated
Interpretation: misidentification
Subsequent usage:
Re-used in **23 mitogenomic phylogenies published before 1 January 2020**: Gao et al. 2013, Marshall et al. 2013, Barker 2014, Ma et al. 2014, Gibb et al. 2015, Li et al. 2016g, Peng et al. 2016b, Peng et al. 2016c, Nabholz et al. 2016, Wen & Liao 2016, Wang & Liang 2016, Zhang et al. 2016, Gomes de Sa et al. 2017, Wen et al. 2017a, Wen et al. 2017b, Caparroz et al. 2018, Liu et al. 2018d, Jiao et al. 2018, Zhang et al. 2018, Zhou et al. 2019b, Sun et al. 2019b, Liu et al. 2019g Mackiewicz et al. 2019.
Re-used in **1 additional non-mitogenome phylogeny**: Zhang & Lu 2019.
Used in **3 other papers**: Husemann et al. 2016, Meimberg et al. 2016, Dong et al. 2018
Problems noted in other works: None.
Relevance: This was the only mitogenome presumed to be of this species (January 2020).
References:

Barker, FK 2014. Mitogenomic data resolve basal relationships among passeriform and passeridan birds. Molecular Phylogenetics and Evolution 79: 313–324.

Caparroz, R, Rocha, AV, Cabanne, GS, Tubaro, P, Aleixo, A, Lemmon, EM & Lemmon, AR 2018. Mitogenomes of two neotropical bird species and the multiple independent origin of mitochondrial gene orders in Passeriformes. Molecular Biology Reports 45: 279-285.

Dong, Y, Li, B & Zhou, L 2018. A new insight into the classification of dusky thrush complex: bearings on the phylogenetic relationships within the Turdidae. Mitochondrial DNA Part A 29: 1245-1252.

Gao, RR, Huang, Y & Lei, FM 2013. Sequencing and analysis of the complete mitochondrial genome of *Remiz consobrinus*. Zool. Res. 34: 228−237.

Gibb, GC, England, R, Hartig, G, McLenachan, PA, Taylor Smith, BL, McComish, BJ, Cooper, A & Penny, D 2015. New Zealand passerines help clarify the diversification of major songbird lineages during the Oligocene. Genome Biol. Evol. 7: 2983–2995.

Gomes de Sá, P, Veras, A, Fontana, CS, Aleixo, A, Burlamaqui, T, Mello, CV, de Vasconcelos, ATR, Prosdocimi, F, Ramos, R, Schneider, M & Silva, A 2017. The assembly and annotation of the complete Rufous-bellied Thrush mitochondrial genome. Mitochondrial DNA Part A. 28: 231–232.

Husemann, M, Sturm, S, Curto, M, Meimberg, H & Habel, JC 2016. Four new mitochondrial genomes of the genus *Zosterops* (aves: passeriformes: zosteropidae) from East Africa with a phylogenetic evaluation of the group. Mitochondrial DNA Part B 1: 544-548.

Jiao, SY, Liu, ZX, Yu, F, Yao, JY, Li, YM & Yan, SQ 2018. Complete mitochondrial genome of Pallas's Leaf Warbler (*Phylloscopus proregulus*). Mitochondrial DNA Part B 3: 211-212.

Li, S, Luo, A, Li, G & Li, W 2016g. Complete mitochondrial genome of the Isabelline Wheatear *Oenanthe isabellina* (Passeriformes, Muscicapidae). Mitochondrial DNA Part B 1: 355-356.

Liu, B, Sun, CH, Wang, LB, Xue, DD, Xu, P, Xie, SB & Lu, CH 2019. The complete mitochondrial genome of Grey-streaked Flycatcher *Muscicapa griseisticta* (Passeriformes: Muscicapidae). Mitochondrial DNA Part B 4: 1857-1858.

Liu, R, Chen, R, Liu, J, Xiong, Y & Kan, X 2018d. Complete mitochondrial genome of *Urocissa erythroryncha* (Passeriformes: Corvidae). Mitochondrial DNA Part B 3: 691-692.

Ma, Y-G, Huang, Y & Lei, FM 2014. Sequencing and phylogenetic analysis of the *Pyrgilauda ruficollis* (Aves, Passeridae) complete mitochondrial genome. Zoological Research 35: 81-91.

Mackiewicz, P, Urantówka, AD, Kroczak, A & Mackiewicz, D 2019. Resolving phylogenetic relationships within Passeriformes based on mitochondrial genes and inferring the evolution of their mitogenomes in terms of duplications. Genome Biology and Evolution 11: 2824-2849.

Marshall, HD, Baker, AJ & Grant, AR 2013. Complete mitochondrial genomes from four subspecies of common chaffinch (*Fringilla coelebs*): New inferences about mitochondrial rate heterogeneity, neutral theory, and phylogenetic relationships within the order Passeriformes. Gene 517: 37-45.

Meimberg, H, Schachtler, C, Curto, M, Husemann, M & Habel, JC 2016. A new amplicon based approach of whole mitogenome sequencing for phylogenetic and phylogeographic analysis: an example of East African white-eyes (Aves, Zosteropidae). Molecular Phylogenetics and Evolution 102: 74–85.

Nabholz, B, Lanfear, R & Fuchs, J 2016. Body mass-corrected molecular rate for bird mitochondrial DNA. Molecular Ecology 25: 4438-4449.

Peng, LF, Yang, DC & Lu, CH 2016b. Complete mitochondrial genome of oriental magpie-robin *Copsychus saularis* (Aves: Muscicapidae). Mitochondrial DNA Part B 1: 21-22.

Peng, LF, Yang, DC & Lu, CH 2016c. Complete mitochondrial genome sequence of Eurasian blackbird, *Turdus merula* (Aves: Turdidae). Mitochondrial DNA Part A 27: 4609-4610.

Sun, Z, Li, Y, Duan, Y & Ma, J 2019b. Characterization of the complete mitochondrial genome of Hair-crested Drongo *Dicrurus hottentottus* (Passeriformes: Dicruridae). Mitochondrial DNA Part B 4: 2013-2014.

Wang, N & Liang, B 2016. Complete mitochondrial genome of a sunbird, *Aethopyga gouldiae* (Aves: Passeriformes), the first representative of Nectariniidae. Mitochondrial DNA Part A 27: 2356-2358.

Wen, L & Liao, F 2016. Complete mitochondrial genome of *Pycnonotus xanthorrhous* (Passeriformes, Pycnonotidae) and phylogenetic consideration. Biochemical Systematics and Ecology 69: 83-90.

Wen, L, Yang, X, Liao, J, Fu, Y & Dai, B 2017a. The complete mitochondrial genome of the Fulvous Parrotbill *Paradoxornis fulvifrons* (Passeriformes: Muscicapidae). Mitochondrial DNA Part A 28: 143-144.

Wen, L, Fu, Y, Dai, B & Liao, J 2017b. The complete mitochondrial genome of the Green-Backed tit *Parus monticolus* (Passeriformes: Paridae). Mitochondrial DNA Part A 28: 79-80.

Zhang, H, Cheng, Y, Zhou, L & Dong, Y 2016. Complete mitochondrial genome of White-throated Rock-thrush *Monticola cinclorhynchus gularis* (Passeriformes: muscicapidae). Mitochondrial DNA Part B 1: 684-685.

Zhang, H, Bai, Y, Shi, X, Sun, L, Wang, Z & Wu, X 2018a. The complete mitochondrial genomes of *Tarsiger cyanurus* and *Phoenicurus auroreus*: a phylogenetic analysis of Passeriformes. Genes & Genomics 40: 151-165.

Zhang, XR & Lu, CH 2019. The complete mitochondrial genome of red-throated flycatcher *Ficedula albicilla* (Passeriformes: Ficedula). Mitochondrial DNA Part B 4: 3322-3323.

Zhou, C, Jin, J, Chen, Y, Hao, Y, Zhang, X, Meng, Y, Yang, N & Yue, B 2019b. Two new complete mitochondrial genomes (*Paradoxornis gularis* and *Niltava davidi*) and their phylogenetic and taxonomic implications. Mitochondrial DNA Part B 4: 820-821.

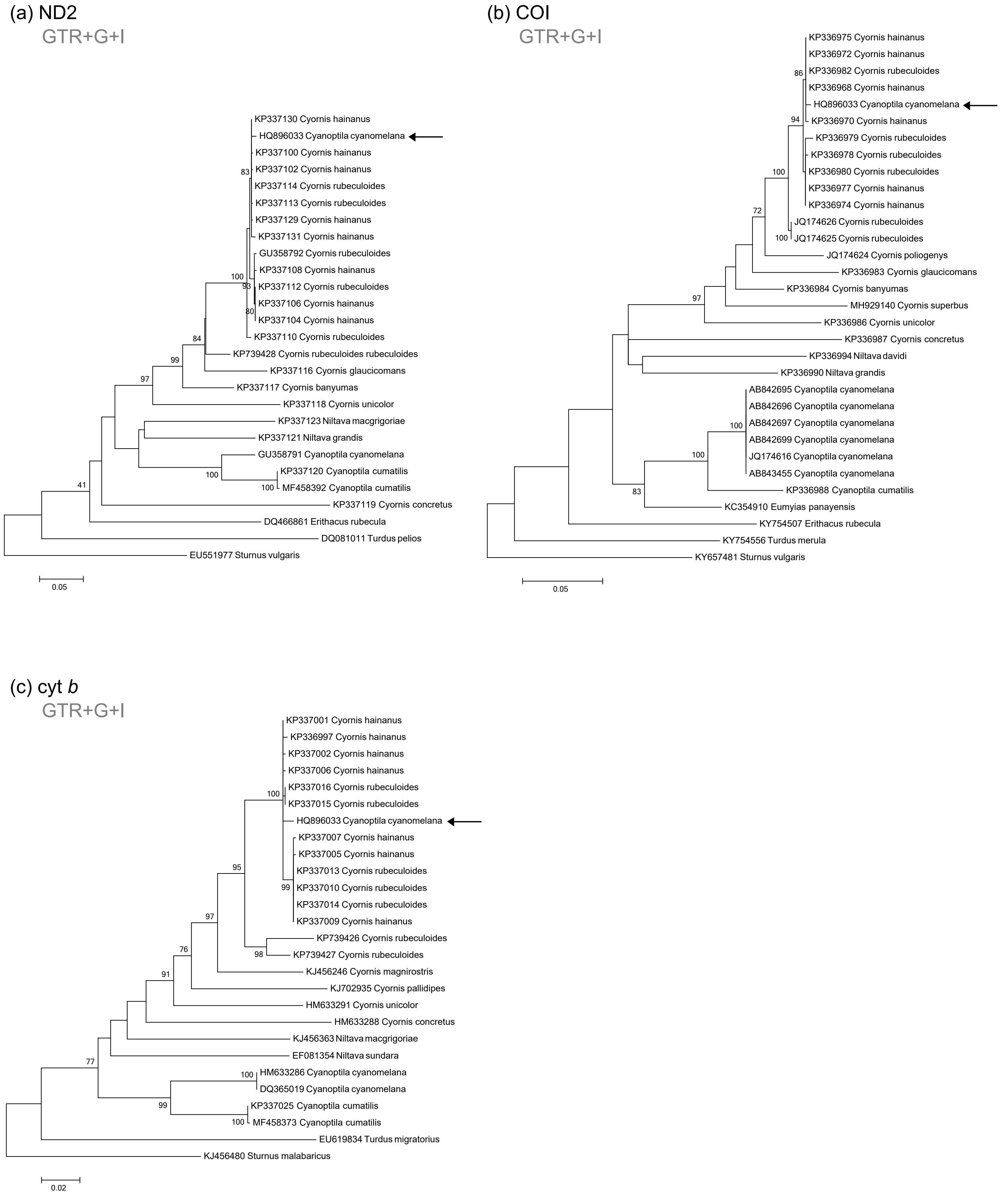

Figure S57. Maximum Likelihood phylogenies of *Cyanoptila cyanomelana* (HQ896033) and related taxa based on mitochondrial sequences. Numbers at branches are bootstrap support values (>70%) based on 1000 replicates.

71. "*Passer ammodendri*" KT895996 (Fan et al. 2017)

Fig. S58

Metadata: The blood sample of the Saxaul Sparrow was collected from the AnXi extremely arid desert national nature reserve (N40°21'–40°22', E96°13'–96°14', at an altitude of 1306 m) in Guazhou, Gansu province. Then we stored the sample in -20 °C. (Fan et al. 2017: 337)

Phylogenetic position:

ND2 = *Emberiza cia / E. godlewskii / E. cioides* (Emberizidae)

CO1 = *Emberiza godlewskii* (Emberizidae)

Cyt *b* = *Passer ammodendri*

Sequence integrity: not investigated

Interpretation: chimera

Subsequent usage:

Re-used in **5 mitogenomic phylogenies published before 1 January 2020**: Wen & Liao 2016, Shi et al. 2017a, Mereu et al. 2017, Gao et al. 2019b, Mackiewicz et al. 2019.

Problems noted in other works: Mackiewicz et al. (2019) noted: "*Passer ammodendri* is grouped in the IQ-TREE phylogeny with other members of its genus, but in the Bayesian trees it is placed unexpectedly among *Emberiza*. This inconsistent placement may result from contamination or misassembly of its mitochondrial genome. We found that 22 subsequent genes in the region from 2719 bp to 9972 bp show greater similarity to the sequences from *Emberiza* than from *Passer*, while the control region and 14 genes in other regions (1-2718 bp and 9974-16782 bp) are more similar to *Passer*. Therefore, the sequence of this genome should be verified.".

Relevance: This was the only mitogenome presumed to be of this species (January 2020)

References:

Fan, Y, Bao, X, Liu, F, Li, J & Yao, X 2017. Sequence and analysis of the complete mitochondrial genome of the Saxaul Sparrow, *Passer ammodendri* (Passeriformes, Passeridae). Mitochondrial DNA Part A 28: 337-339.

Gao, X, Xu, D, Xia, T, Dou, H, Sha, W & Zhang, H 2019b. The complete mitochondrial genome of Eastern Yellow Wagtail (*Motacilla tschutschensis*). Mitochondrial DNA Part B 4: 3486-3487.

Mackiewicz, P, Urantówka, AD, Kroczak, A & Mackiewicz, D 2019. Resolving phylogenetic relationships within Passeriformes based on mitochondrial genes and inferring the evolution of their mitogenomes in terms of duplications. Genome Biology and Evolution 11: 2824-2849.

Mereu, P, Satta, V, Frongia, GN, Berlinguer, F, Muzzeddu, M, Campus, A, Decandia, L, Pirastru, M, Manca, L, Naitana, S & Leoni, GG 2017. The complete mtDNA sequence of the griffon vulture (*Gyps fulvus*): Phylogenetic analysis and haplotype frequency variations after restocking in the Sardinian population. Biological Conservation 214: 195-205.

Shi, R, Chen, K & Li, S 2017a. A novel gene organization of the rock sparrow *Petronia petronia* (Aves: Passeriformes) revealed by complete mitochondrial genome. Mitochondrial DNA Part B 2: 858-859.

Wen, L & Liao, F 2016. Complete mitochondrial genome of *Pycnonotus xanthorrhous* (Passeriformes, Pycnonotidae) and phylogenetic consideration. Biochemical Systematics and Ecology 69: 83-90.

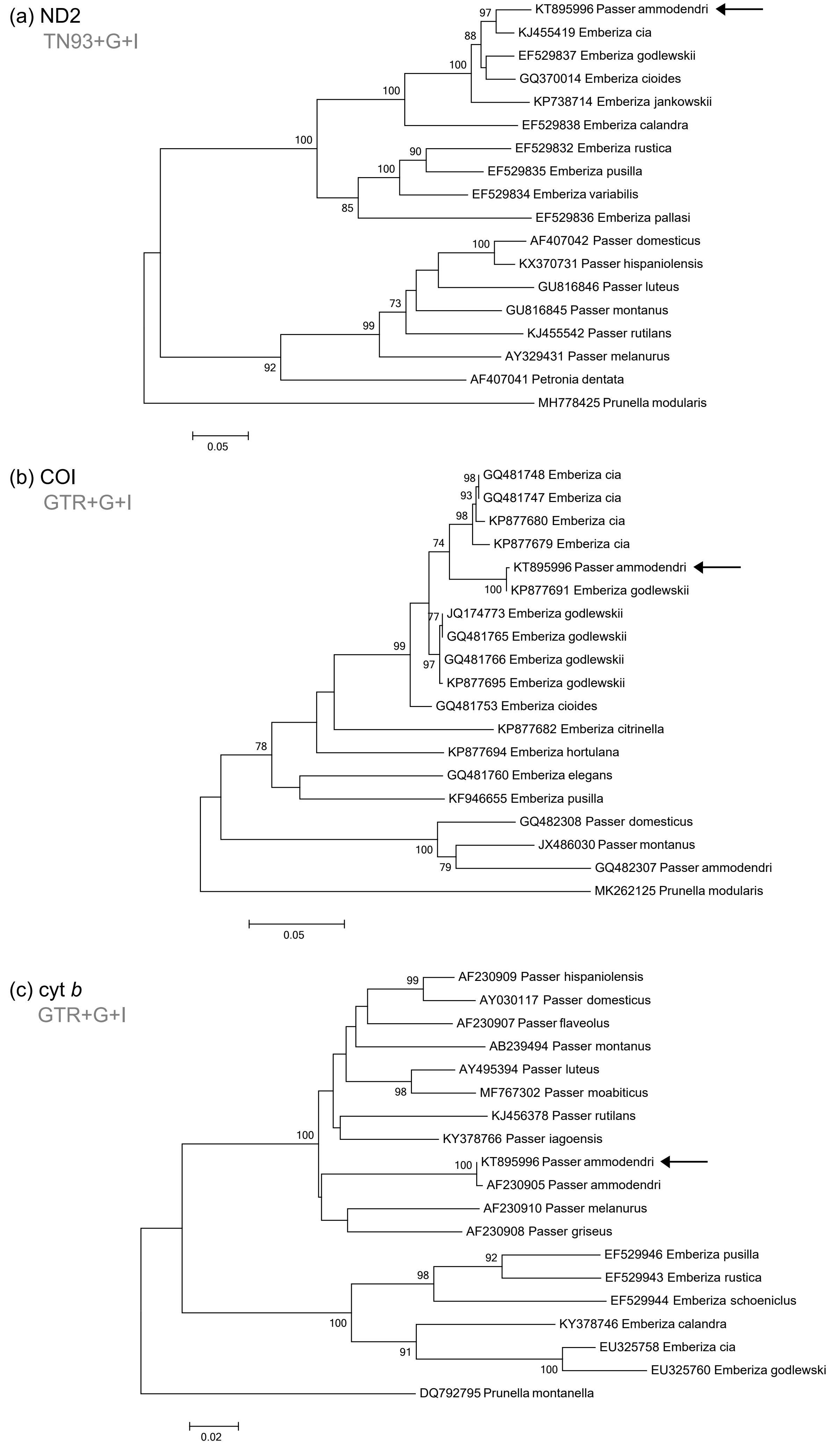

Figure S58. Maximum Likelihood phylogenies of *Passer ammodendri* (KT895996) and related taxa based on mitochondrial sequences. Numbers at branches are bootstrap support values (>70%) based on 1000 replicates.

72. "*Passer montanus*" MH211399 (Huang, Z. and Tu, F., unpublished; 2019)

Fig. S59

Metadata: no data on GenBank
Phylogenetic position:
ND2 = *Passer domesticus*
CO1 = *Passer domesticus*
Cyt *b* = *Passer domesticus*
Sequence integrity: not investigated
Interpretation: misidentification
Subsequent usage: not used in any further studies (January 2020)
Problems noted in other works: none
Relevance: As of January 2020, five other mitogenomes of *Passer montanus* have been published (JX486030, MH211396, MH211397, MH211398, MH211399).

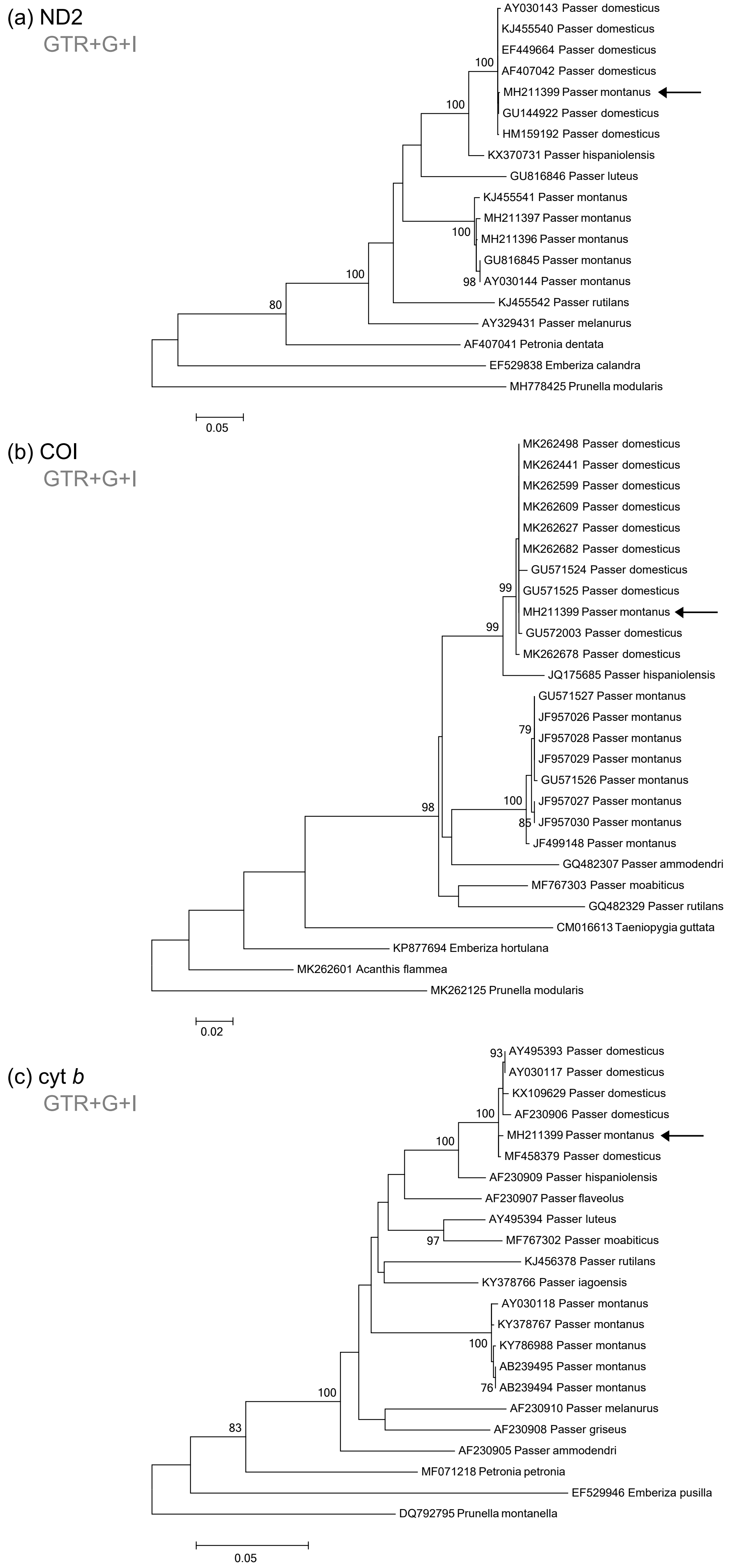

Figure S59. Maximum Likelihood phylogenies of *Passer montanus* (MH211399) and related taxa based on mitochondrial sequences. Numbers at branches are bootstrap support values (>70%) based on 1000 replicates.

73. “*Motacilla lugens*” KU246035, NC_029703 (Park, C.E., Park, G.S., Park, Y.J., Kim, M.C., Park, H.C. & Shin, J.H., unpublished; 2015)

Fig. S60

Metadata: no data on GenBank
Phylogenetic position:
ND2 = among *M. alba/lugens* but on a long branch
CO1 = *Motacilla alba* (incl. *lugens*)
Cyt *b* = *Cyanopica cyanus* (Corvidae)
Sequence integrity:
ND2: bp 1 – circa 891 = *M. alba/lugens*; bp circa 898 – circa 1038 = *Cyanopica cyanus.*
Interpretation: chimera.
Subsequent usage:
Re-used in **4 mitogenomic phylogenies published before 1 January 2020**: Sun et al. 2016a, Wang et al. 2019a, Gao et al. 2019b, Mackiewicz et al. 2019
Re-used in **1 non-mitogenomic phylogeny**: Sun et al. 2016b (on a long branch),
Problems noted in other works: None.
Relevance: This was the only mitogenome presumed to be of this species (January 2020).
References:

Gao, X, Xu, D, Xia, T, Dou, H, Sha, W & Zhang, H 2019b. The complete mitochondrial genome of Eastern Yellow Wagtail (*Motacilla tschutschensis*). Mitochondrial DNA Part B 4: 3486-3487.

Mackiewicz, P, Urantówka, AD, Kroczak, A & Mackiewicz, D 2019. Resolving phylogenetic relationships within Passeriformes based on mitochondrial genes and inferring the evolution of their mitogenomes in terms of duplications. Genome Biology and Evolution 11: 2824-2849.

Sun, P, Zhang, C, Pang, M, Qian, L, Pan, T, Wang, H & Zhang, B 2016b. The complete mitochondrial genome of *Anthus hodgsoni* (Passeriformes: Motacillidae). Mitochondrial DNA Part B 1: 504-505.

Sun, X, Sun, Z, Liu, D & Zhou, W 2016a. Phylogenetic studies of *Prunella strophiata* (Passeridae: *Prunella*) based on complete mitochondrial DNA sequences. Mitochondrial DNA Part B 1: 450-451.

Wang, L, Dong, Y, Xuan, J, Zhang, F, Fu, R, Liu, G, Xiang, X & Zhou, L 2019a. Complete mitochondrial genome of *Anthus richardi* (Passeriformes: Motacillidae). Mitochondrial DNA Part B 4: 271-272.

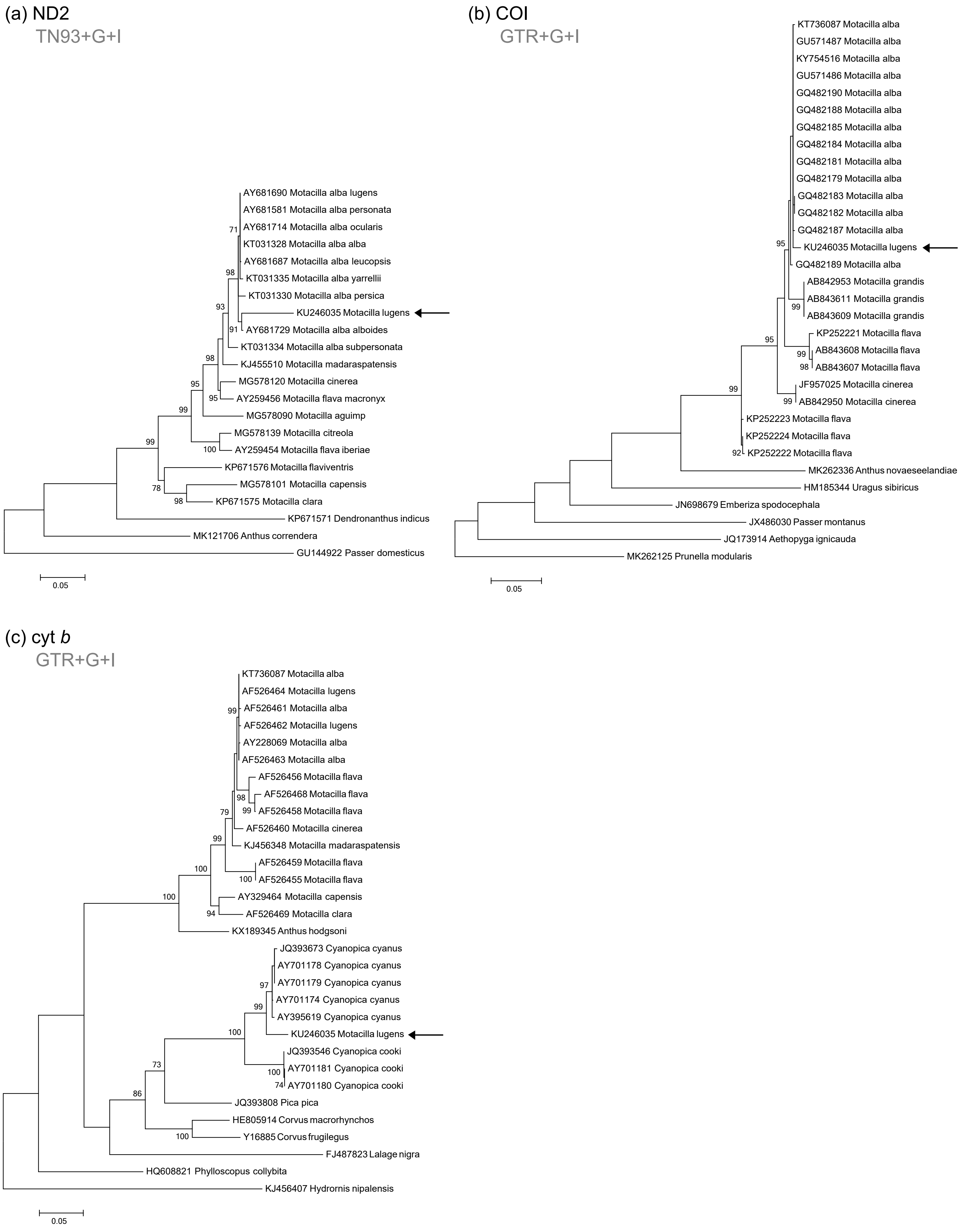

Figure S60. Maximum Likelihood phylogenies of *Motacilla lugens* (KU246035) and related taxa based on mitochondrial sequences. Numbers at branches are bootstrap support values (>70%) based on 1000 replicates.

74. "*Leucosticte arctoa*" KM078791, NC_025615 (Lerner et al. 2011)

Fig. S61

Metadata: USNM 609762, Colorado, USA (Lerner et al. 2011)
Phylogenetic position:
ND2 = outside the *L. arctoa* clade; instead in clade with *L. tephrocotis, L. atrata, L. australis*
CO1 = *Leucosticte tephrocotis*
Cyt *b* = *Leucosticte tephrocotis*
Sequence integrity: not investigated
Interpretation: misidentification due to outdated taxonomy
Subsequent usage:
Re-used in **5 mitogenomic phylogenies published before 1 January 2020**: Li et al. 2016e, Nabholz et al. 2016, Shi et al. 2017, Liu et al. 2019, Mackiewicz et al. 2019.
Problems noted in other works: None.
Relevance: This was the only mitogenome presumed to be of this species (January 2020)
References:

Lerner, HRL, Meyer, M, James, HF, Hofreiter, M & Fleischer, RC 2011. Multilocus resolution of phylogeny and timescale in the extant adaptive radiation of Hawaiian honeycreepers. Curr. Biol. 21: 1838-1844.

Li, YM, Bai, CY, Zhang, Y & Yan, SQ 2016e. Complete mitochondrial genome of the Common redpoll (*Carduelis flammea*). Mitochondrial DNA Part A 27: 2953-2954.

Liu, F, Antalffy, J & Wen, L 2019. The complete mitochondrial genome sequences of two *Emberiza* (Aves, Passeriformes). Mitochondrial DNA Part B 4: 914-915.

Mackiewicz, P, Urantówka, AD, Kroczak, A & Mackiewicz, D 2019. Resolving phylogenetic relationships within Passeriformes based on mitochondrial genes and inferring the evolution of their mitogenomes in terms of duplications. Genome Biology and Evolution 11: 2824-2849.

Nabholz, B, Lanfear, R & Fuchs, J 2016. Body mass-corrected molecular rate for bird mitochondrial DNA. Mol. Ecol. 25: 4438-4449.

Shi, Q, Liu, Y & Zhao, HF 2017b. Characterization of the complete mitochondrial genome of slaty bunting *Emberiza siemsseni* (Passeriformes: Fringillidae). Conservation Genetics Resources 9: 107-110.

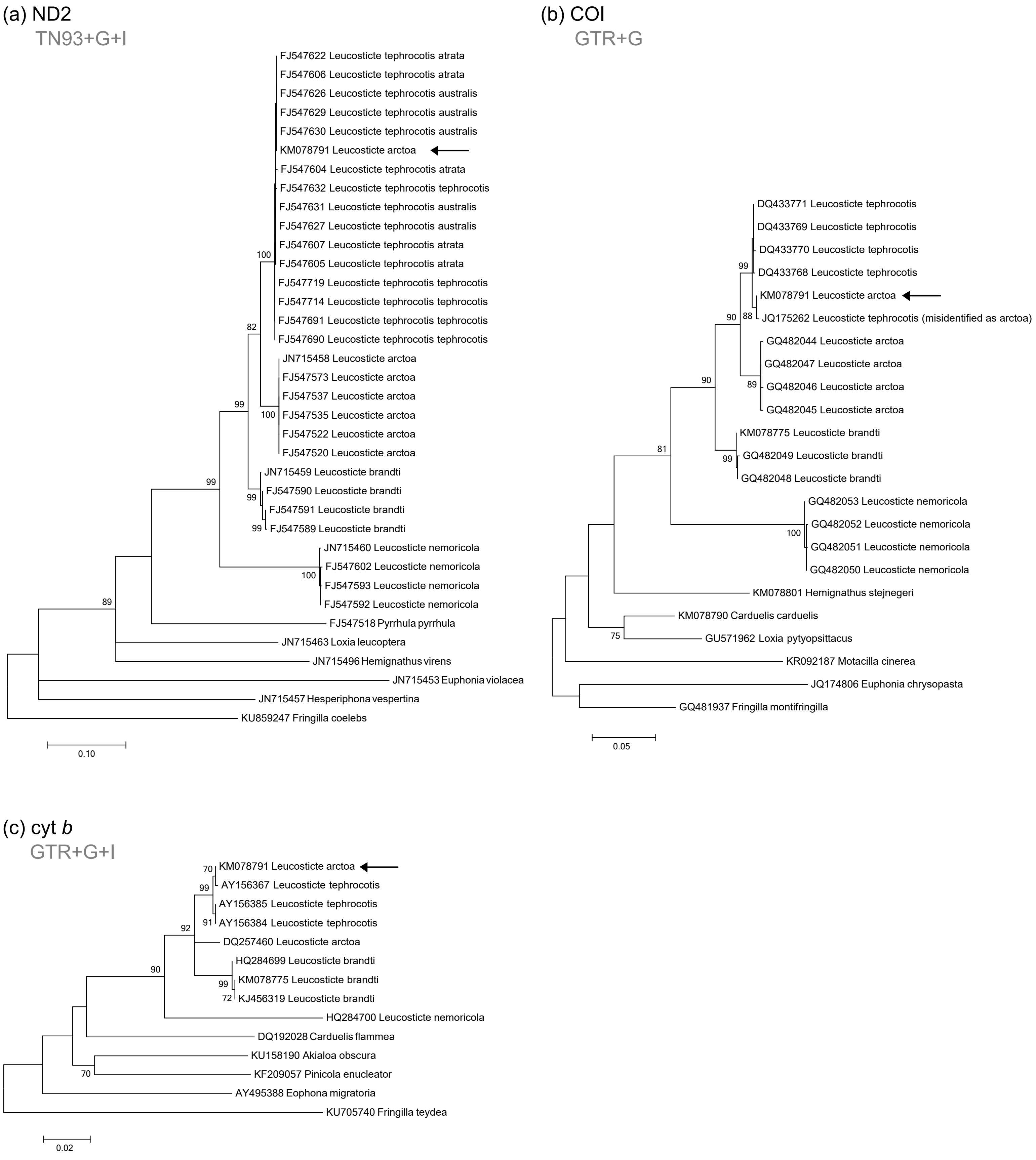

Figure S61. Maximum Likelihood phylogenies of *Leucosticte arctoa* (KM078791) and related taxa based on mitochondrial sequences. Numbers at branches are bootstrap support values (>70%) based on 1000 replicates.

75. “*Haemorhous mexicanus*” FJ236300 (Carson, R.J. and Spicer, G.S., unpublished; 2008)

Fig. S62

Metadata: GenBank: country="USA: California, Monterey County". "SFSU SL93"
Phylogenetic position:
ND2 = *H. purpureus*
CO1 = *H. purpureus*
Cyt *b* = *H. purpureus*
Sequence integrity: not investigated
Interpretation: misidentification
Subsequent usage: As of 1 January 2020, this sequence was neither cited nor re-used.
Problems noted in other works: None.
Relevance: A correctly-identified sequence of this species (KM078782) was published by Lerner et al. (2011).
References:

Lerner, HRL, Meyer, M, James, HF, Hofreiter, M & Fleischer, RC 2011. Multilocus resolution of phylogeny and timescale in the extant adaptive radiation of Hawaiian honeycreepers. Curr. Biol. 21: 1838-1844.

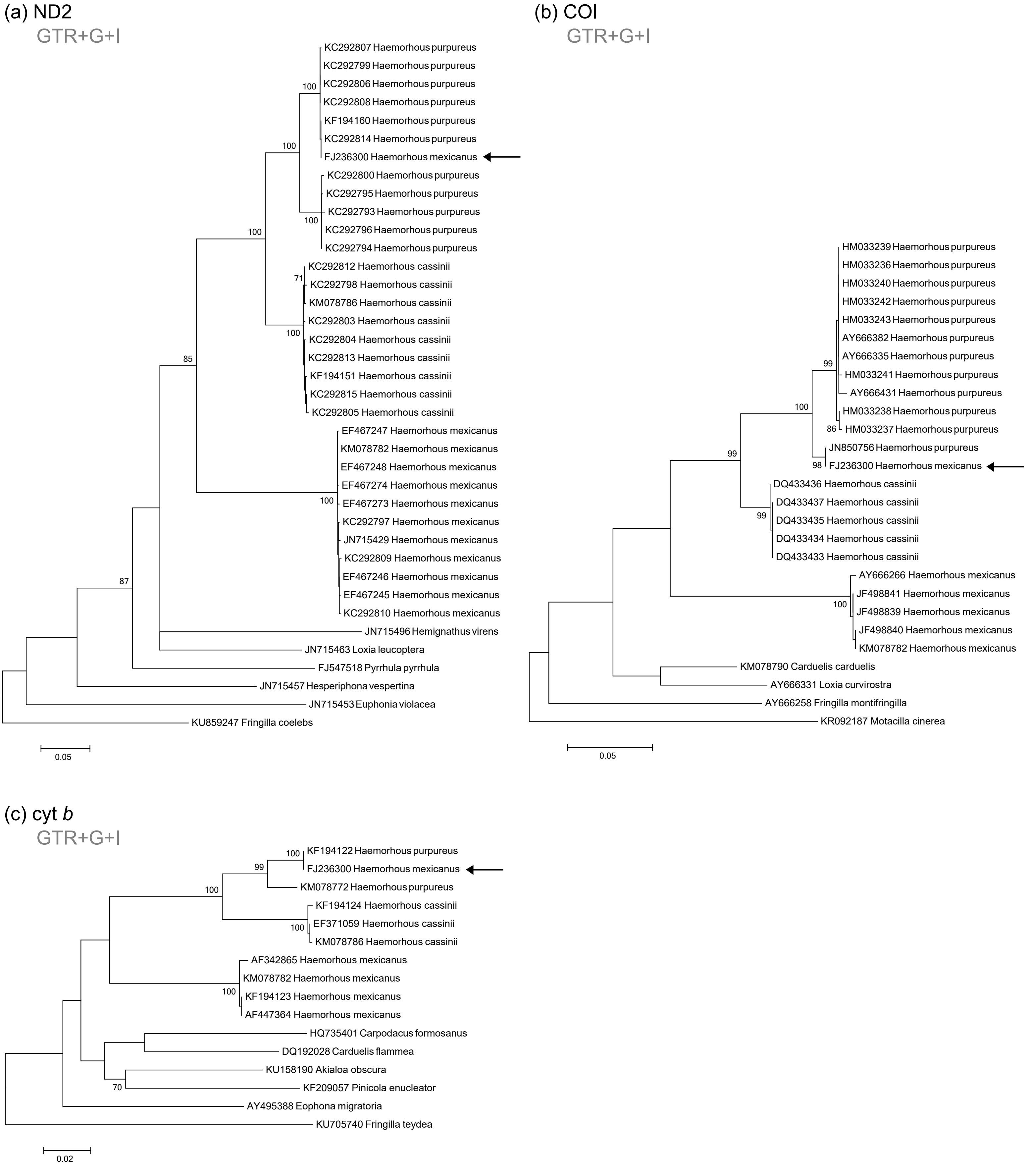

Figure S62. Maximum Likelihood phylogenies of *Haemorhous mexicanus* (FJ236300) and related taxa based on mitochondrial sequences. Numbers at branches are bootstrap support values (>70%) based on 1000 replicates.

76. “*Emberiza chrysophrys*” HQ896034, NC_015233 (Ren et al. 2014a)

Fig. S63

Metadata: The *E. chrysophrys* specimen (code Kan-K0095) was collected from Wuhu (31°21’N, 118°22’E), Anhui, China. The voucher specimen was deposited in the College of Life Sciences, Anhui Normal University, China. (Ren et al. 2014a: 700)

Phylogenetic position:

ND2 = could not be verified due to lack of sequences of *Emberiza chrysophrys*

CO1 = highly divergent from three *Emberiza chrysophrys*; sister to multiple species of *Emberiza*

Cyt *b* = *Emberiza chrysophrys*

Sequence integrity:

COI (i) contains an inferred 3 bp gap at position 151; (ii) contains several nucleotides that are unique among COI sequences of *Emberiza* (n=291): 27T, 290C, 291T, 338G, 431A, 521G, 558A, 587T; (iii) is 9.0-9.5% divergent from three sequences of *E. chrysophrys*.

Interpretation: Sequencing errors / numts

Subsequent usage:

Re-used in **12 mitogenomic phylogenies published before 1 January 2020**: Gao et al. 2013, Marshall et al. 2013, Ma et al. 2014, Nabholz et al. 2016, Zhang et al. 2016, Zhao et al. 2016, Shi et al. 2017, Lee et al. 2017, Zhang et al. 2018, Liu et al. 2019, Wu et al. 2019b, Mackiewicz et al. 2019.

Cited / mentioned in **4 further papers**: Toews et al. 2013 (**assembly of mitogenome**), Pan et al. 2015b, Husemann et al. 2016, Meimberg et al. 2016.

Problems noted in other works: None.

Relevance: this was the only presumed mitogenome of this species (January 2020).

References:

Gao, RR, Huang, Y & Lei, FM 2013. Sequencing and analysis of the complete mitochondrial genome of *Remiz consobrinus*. Zool. Res. 34: 228−237.

Husemann, M, Sturm, S, Curto, M, Meimberg, H & Habel, JC 2016. Four new mitochondrial genomes of the genus *Zosterops* (aves: passeriformes: zosteropidae) from East Africa with a phylogenetic evaluation of the group. Mitochondrial DNA Part B 1: 544-548.

Lee, MY, Jeon, HS & An, J 2017. Complete mitochondrial genome sequence of *Emberiza sulphurata* (Emberizidae: *Emberiza*). Mitochondrial DNA Part B 2: 111-112.

Liu, F, Antalffy, J & Wen, L 2019. The complete mitochondrial genome sequences of two *Emberiza* (Aves, Passeriformes). Mitochondrial DNA Part B 4: 914-915.

Ma, Y-G, Huang, Y & Lei, FM 2014. Sequencing and phylogenetic analysis of the *Pyrgilauda ruficollis* (Aves, Passeridae) complete mitochondrial genome. Zoological Research 35: 81-91.

Mackiewicz, P, Urantówka, AD, Kroczak, A & Mackiewicz, D 2019. Resolving phylogenetic relationships within Passeriformes based on mitochondrial genes and inferring the evolution of their mitogenomes in terms of duplications. Genome Biology and Evolution 11: 2824-2849.

Marshall, HD, Baker, AJ & Grant, AR 2013. Complete mitochondrial genomes from four subspecies of common chaffinch (*Fringilla coelebs*): New inferences about mitochondrial rate heterogeneity, neutral theory, and phylogenetic relationships within the order Passeriformes. Gene 517: 37-45.

Meimberg, H, Schachtler, C, Curto, M, Husemann, M & Habel, JC 2016. A new amplicon based approach of whole mitogenome sequencing for phylogenetic and phylogeographic analysis: an example of East African white-eyes (Aves, Zosteropidae). Molecular Phylogenetics and Evolution 102: 74–85.

Nabholz, B, Lanfear, R & Fuchs, J 2016. Body mass-corrected molecular rate for bird mitochondrial DNA. Mol. Ecol. 25: 4438-4449.

Ren, Q, Yuan, J, Ren, L, Zhang, L, Zhang, L, Jiang, L, Chen, D, Kan, X & Zhang, B 2014a. The complete mitochondrial genome of the Yellow-browed Bunting, *Emberiza chrysophrys* (Passeriformes: Emberizidae), and phylogenetic relationships within the genus *Emberiza*. Journal of Genetics 93: 699-707.

Shi, Q, Liu, Y & Zhao, HF 2017b. Characterization of the complete mitochondrial genome of slaty bunting *Emberiza siemsseni* (Passeriformes: Fringillidae). Conservation Genetics Resources 9: 107-110.

Toews, DPL, Mandic, M, Richards, JG & Irwin, DE 2013. Migration, mitochondria, and the Yellow-rumped Warbler. Evolution 68: 241-255.

Wu, G, Zhang, H, Pan, T, Zhang, Y & Zhang, B 2019b. Complete mitochondrial genome sequence of *Emberiza pallasi* (Emberizidae: *Emberiza*). Mitochondrial DNA Part B 4: 2015-2016.

Zhang, H, Bai, Y, Shi, X, Sun, L, Wang, Z & Wu, X 2018. The complete mitochondrial genomes of *Tarsiger cyanurus* and *Phoenicurus auroreus*: a phylogenetic analysis of Passeriformes. Genes & Genomics 40: 151-165.

Zhang, Y, Li, K, Pan, T, Peng, Z, Sun, X, Kang, X, Zhang, Y & Zhang, B 2016c. Mitochondrial genome of the *Melophus lathami*. Mitochondrial DNA Part B 1: 929-930.

Zhao, YJ, Li, D, Li, S, Xi, YY & Jiang, YL 2016. Mitochondrial genome of the *Emberiza jankowskii* (Emberizidae: *Emberiza*). Mitochondrial DNA Part A 27: 2458-2459.

77. “*Emberiza aureola*” KF111713, NC_022150 (Pan et al. 2015a)

Fig. S63

Metadata: no data in Pan et al. (2015a) or on GenBank.
Phylogenetic position:
ND2 = sister to *Emberiza rutila* but on a very long branch
CO1 = *Emberiza rutila*
Cyt *b* = *Emberiza rutila*
Sequence integrity: ND2: positions 1 – circa 354 = *E. rutila*. Positions 364 – 966 were divergent from *Emberiza rutila* (n=1) and *E. aureola* (n=1) and showed many unique mutations relative to both species. However, the latter fragment did not closely match any other species.
Interpretation: misidentification, in combination with poor sequence quality / numt in part of ND2.
Subsequent usage:
Re-used in **12 mitogenomic phylogenies published before 1 January 2020**: Ren et al. 2014a, Gibb et al. 2015, Wang & Liang 2016, Zhao et al. 2016, Nabholz et al. 2016, Zhang et al. 2016c, Shi et al. 2017a; Cao et al. 2017, Lee et al. 2017, Liu et al. 2019, Wu et al. 2019b, Mackiewicz et al. 2019.
Cited / mentioned in **1 further paper**: Pan et al. 2015b
Problems noted in other works: None.
Relevance: This was the only presumed mitogenome of this species (January 2020). A mitogenome of *E. rutila* was already available (Ren et al. 2014). Critically Endangered (IUCN 2017).
References:

Cao, Z, Li, G, Chen, C & Li, S 2017. Characterization of complete mitochondrial genome of the Brown Accentor *Prunella fulvescens* on the Tibet Plateau. Mitochondrial DNA Part B 2: 435-436.

Gibb, GC, England, R, Hartig, G, McLenachan, PA, Taylor Smith, BL, McComish, BJ, Cooper, A & Penny, D 2015. New Zealand passerines help clarify the diversification of major songbird lineages during the Oligocene. Genome Biol. Evol. 7: 2983–2995.

IUCN 2017. *IUCN Red List of Threatened Species*. Version 2017-1. http://www.iucnredlist.org. Downloaded in June 2017.

Lee, MY, Jeon, HS & An, J 2017. Complete mitochondrial genome sequence of *Emberiza sulphurata* (Emberizidae: *Emberiza*). Mitochondrial DNA Part B 2: 111-112.

Liu, F, Antalffy, J & Wen, L 2019. The complete mitochondrial genome sequences of two *Emberiza* (Aves, Passeriformes). Mitochondrial DNA Part B 4: 914-915.

Mackiewicz, P, Urantówka, AD, Kroczak, A & Mackiewicz, D 2019. Resolving phylogenetic relationships within Passeriformes based on mitochondrial genes and inferring the evolution of their mitogenomes in terms of duplications. Genome Biology and Evolution 11: 2824-2849.

Nabholz, B, Lanfear, R & Fuchs, J 2016. Body mass-corrected molecular rate for bird mitochondrial DNA. Mol. Ecol. 25: 4438-4449.

Pan, T, Ren, L, Zhang, C, Hu, C, Yu, L, Hou, Y, Chang, X & Zhang, B 2015b. Mitochondrial genome of the *Emberiza cioides* (Emberizidae: *Emberiza*). Mitochondrial DNA 26: 295-296.

Pan, T, Ren, L, Zhu, X, Yan, L, Hu, C, Chang, Q & Zhang, B 2015a. Mitochondrial genome of the *Emberiza aureola* (Emberizidae: *Emberiza*). Mitochondrial DNA 26(1): 121-122.

Ren, Q, Yuan, J, Ren, L, Zhang, L, Zhang, L, Jiang, L, Chen, D, Kan, X & Zhang, B 2014a. The complete mitochondrial genome of the yellow-browed bunting, *Emberiza chrysophrys* (Passeriformes: Emberizidae), and phylogenetic relationships within the genus *Emberiza*. Journal of Genetics *93:* 699-707.

Shi, R, Chen, K & Li, S 2017a. A novel gene organization of the rock sparrow *Petronia petronia* (Aves: Passeriformes) revealed by complete mitochondrial genome. Mitochondrial DNA Part B 2: 858-859.

Shi, Q, Liu, Y & Zhao, HF 2017b. Characterization of the complete mitochondrial genome of slaty bunting *Emberiza siemsseni* (Passeriformes: Fringillidae). Conservation Genetics Resources 9: 107-110.

Wang, N & Liang, B 2016. Complete mitochondrial genome of a sunbird, *Aethopyga gouldiae* (Aves: Passeriformes), the first representative of Nectariniidae. Mitochondrial DNA Part A 27: 2356-2358.
Wu, G, Zhang, H, Pan, T, Zhang, Y & Zhang, B 2019b. Complete mitochondrial genome sequence of *Emberiza pallasi* (Emberizidae: *Emberiza*). Mitochondrial DNA Part B 4: 2015-2016.
Zhang, Y, Li, K, Pan, T, Peng, Z, Sun, X, Kang, X, Zhang, Y & Zhang, B 2016c. Mitochondrial genome of the *Melophus lathami*. Mitochondrial DNA Part B 1: 929-930.
Zhao, YJ, Li, D, Li, S, Xi, YY & Jiang, YL 2016. Mitochondrial genome of the *Emberiza jankowskii* (Emberizidae: *Emberiza*). Mitochondrial DNA Part A 27: 2458-2459.

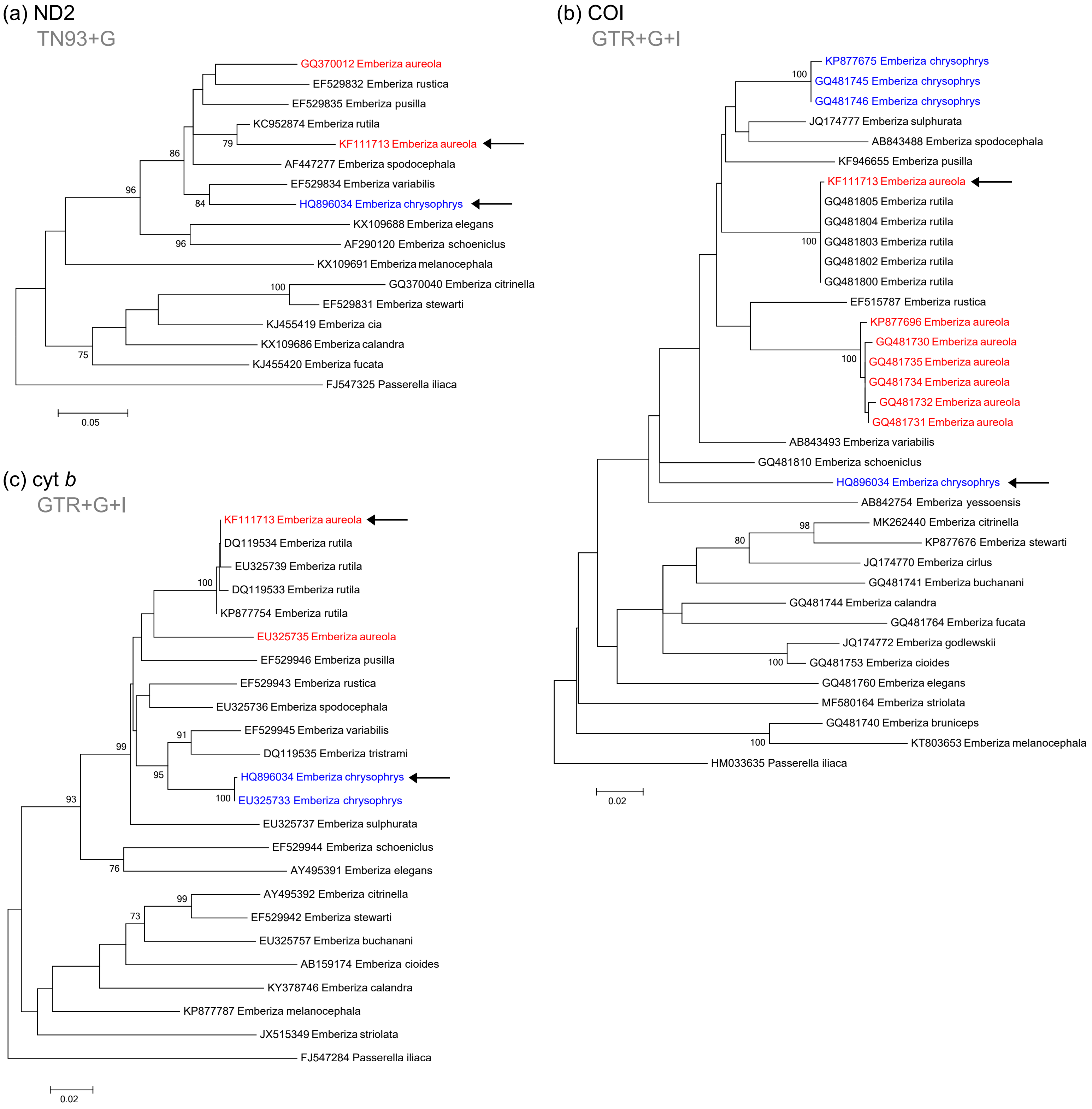

Figure S63. Maximum Likelihood phylogenies of *Emberiza chrysophrys* (HQ896034), *E. aureola* (KF111713) and related taxa based on mitochondrial sequences. Numbers at branches are bootstrap support values (>70%) based on 1000 replicates.

78. “*Emberiza pallasi*” MK687386 (Wu et al. 2019b)

Fig. S64

Metadata: “The *E. pallasi* used in this study was collected from Dashu Mountain, Hefei, Anhui Province, China (November, 2016) and stored in the museum of Anhui Key Laboratory of Ecoengineering and Bio-technique, Anhui University, China.” (Wu et al. 2019b: 2015)

Phylogenetic position:

ND2 = = sister to, but divergent from, one *Emberiza pallasi* (EF529836)

CO1 = in a clade with seven *Emberiza pallasi* but on a slightly longer branch

Cyt *b* = sister to, but highly divergent from, six *Emberiza pallasi*

Sequence integrity: COI: MK687386 includes 5 substitutions that are not found in six *E. pallasi*; four of these are also not found in 24 *E. schoeniclus.*

Cyt b: bp 1-435 of MK687386 include 23 substitutions that are neither found in six *E. pallasi* nor in five *E. schoeniclus*. A BLAST search showed that the closest match to this fragment is a sequence of *E. schoeniclus* (EF529944) but with only 93.7% similarity.

Interpretation: Sequencing errors / numts

Subsequent usage: As of 1 January 2020, this sequence was not re-used.

Problems noted in other works: None.

Relevance: This was the only presumed mitogenome of this species (January 2020).

References:

Wu, G, Zhang, H, Pan, T, Zhang, Y & Zhang, B 2019b. Complete mitochondrial genome sequence of *Emberiza pallasi* (Emberizidae: Emberiza). Mitochondrial DNA Part B 4: 2015-2016.

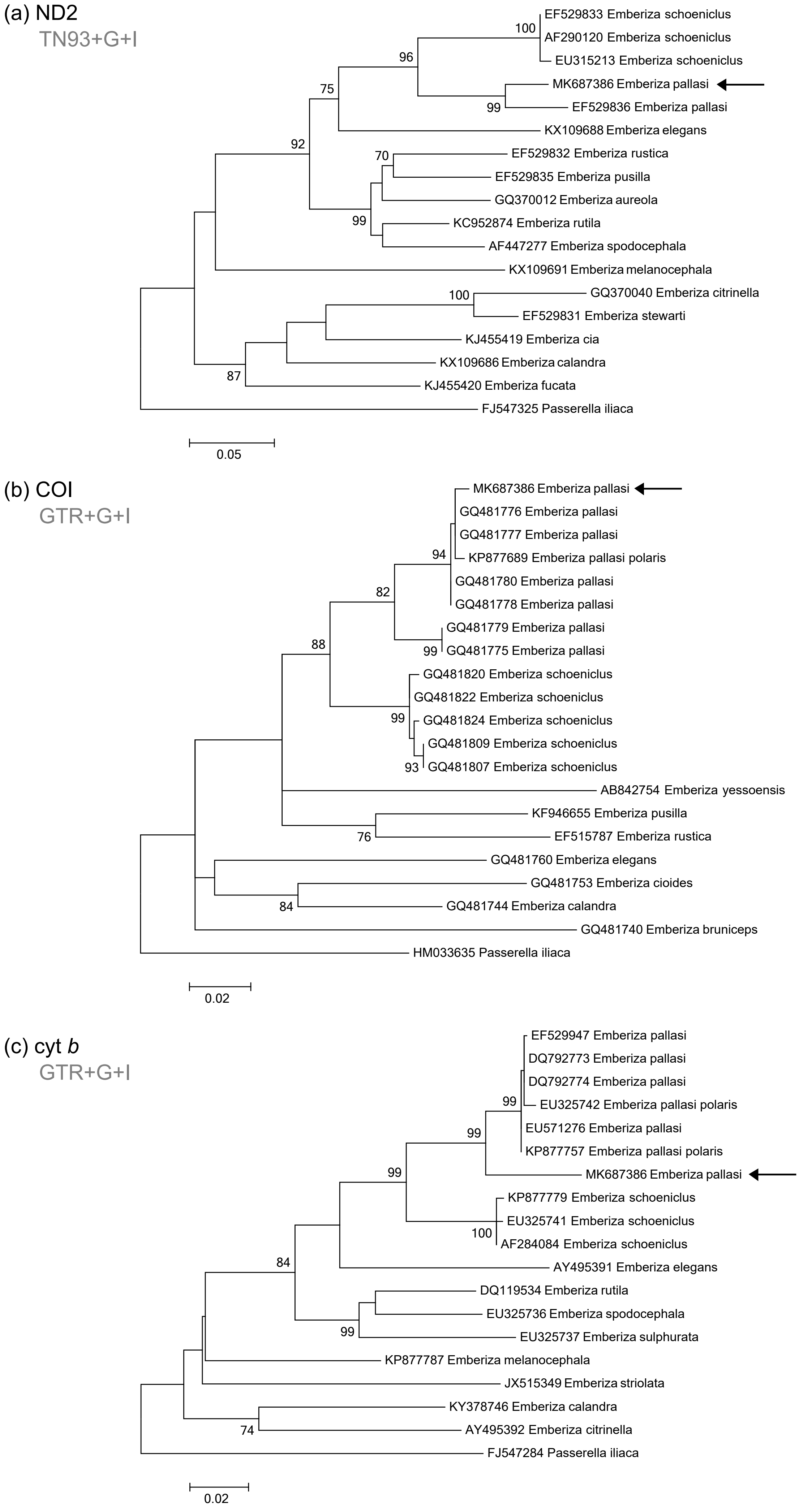

Figure S64. Maximum Likelihood phylogenies of *Emberiza pallasi* (MK687386) and related taxa based on mitochondrial sequences. Numbers at branches are bootstrap support values (>70%) based on 1000 replicates.

APPENDIX S3. Problematic mitogenomes reported by others which could not be confirmed in the present study.

1. "*Ninox novaeseelandiae*" AY309457 (Harrison et al. 2004)
2. "*Bubo blakistoni*" LC099104 (Spiridinova & Surmach 2018)

Fig. S65

Background
The mitogenomes of *Ninox novaeseelandiae* (AY309457, Harrison et al., 2004) and *Bubo blakistoni* (LC099104, Spiridinova & Surmach 2018) were excluded in the phylogenetic analysis "for numerous ambiguities in its PCGs" (Kang et al. 2018). AY309457 represents a complete mitogenome of 16,223 bp, whereas LC099104 represents a partial mitogenome of 19,484 bp. The latter was unpublished at the time of Kang et al. (2018) but was subsequently published with nine other mitogenomes of *B. blakistoni* (Spiridinova & Surmach 2018). It is unclear what Kang et al. (2018) meant by 'numerous ambiguities', but if these sequences show major problems, one would expect either (i) issues with their phylogenetic position, or (ii) unusually long branches in mitogenomic trees or at least some single-gene trees.

Methods
We conducted two tests:
(1) Phylogenetic analyses of Cyt *b*, COI, ND2 to assess the phylogenetic position and branch length of the two sequences in comparison with other sequences of *Ninox* and *Bubo*.
(2) Phylogenetic analysis of complete or partial mitogenomes of owls (n=29; 13 PCGs). The analysis was performed using *Pandion haliaetus* (DQ780884) as outgroup and with complete deletion of missing sites. There were a total of 8474 positions in the final dataset.

Results
Ninox novaeseelandiae:
ND2: AY309457 was nearly identical to two sequences of *N. novaeseelandiae* (**Fig. S65a**).
COI: AY309457 was nearly identical to five sequences of *N. novaeseelandiae* (**Fig. S65b**).
Cyt *b*: AY309457 was placed among *N. novaeseelandiae* and was nearly identical to *N. n. novaeseelandiae* and *N. n. undulata* (**Fig. S65c**).
In the mitogenomic tree (13 PCGs), AY309457 was placed with *Ninox scutulata* and *Sceloglaux albifacies*, and its branch length was unremarkable (**Fig. S65d**).

Bubo blakistoni:
ND2: LC099104 was identical to three other sequences of *B. blakistoni.*
COI: LC099104 was identical to seven other sequences of *B. blakistoni.*
Cyt *b*: LC099104 was nearly identical to four sequences of *B. blakistoni.*
Mitogenomic tree (13 PCGs): LC099104 was identical to three other *B. blakistoni.*

Conclusions
We found no evidence for major problems with these two sequences. The sequences of *Ninox novaeseelandiae* (AY309457) and *Bubo blakistoni* (LC099104) ended up in their expected phylogenetic position in the single-gene and mitogenomic trees. Branches were not unexpectedly long in single-gene and mitogenomic trees.

References
Harrison, GL, McLenachan, PA, Phillips, MJ, Slack, KE, Cooper, A & Penny, D 2004. Four new avian mitochondrial genomes help get to basic evolutionary questions in the Late Cretaceous. Mol. Biol. Evol. 21: 974-983.
Kang, H, Li, B, Ma, X & Xu, Y 2018. Evolutionary progression of mitochondrial gene rearrangements and phylogenetic relationships in Strigidae (Strigiformes). Gene 674: 8-14.

Spiridonova, LN & Surmach, SG 2018. Whole mitochondrial genome of Blakiston's Fish Owl *Bubo* (*Ketupa*) *blakistoni* suggests its redescription in the genus *Ketupa*. Russian Journal of Genetics 54: 369-373.

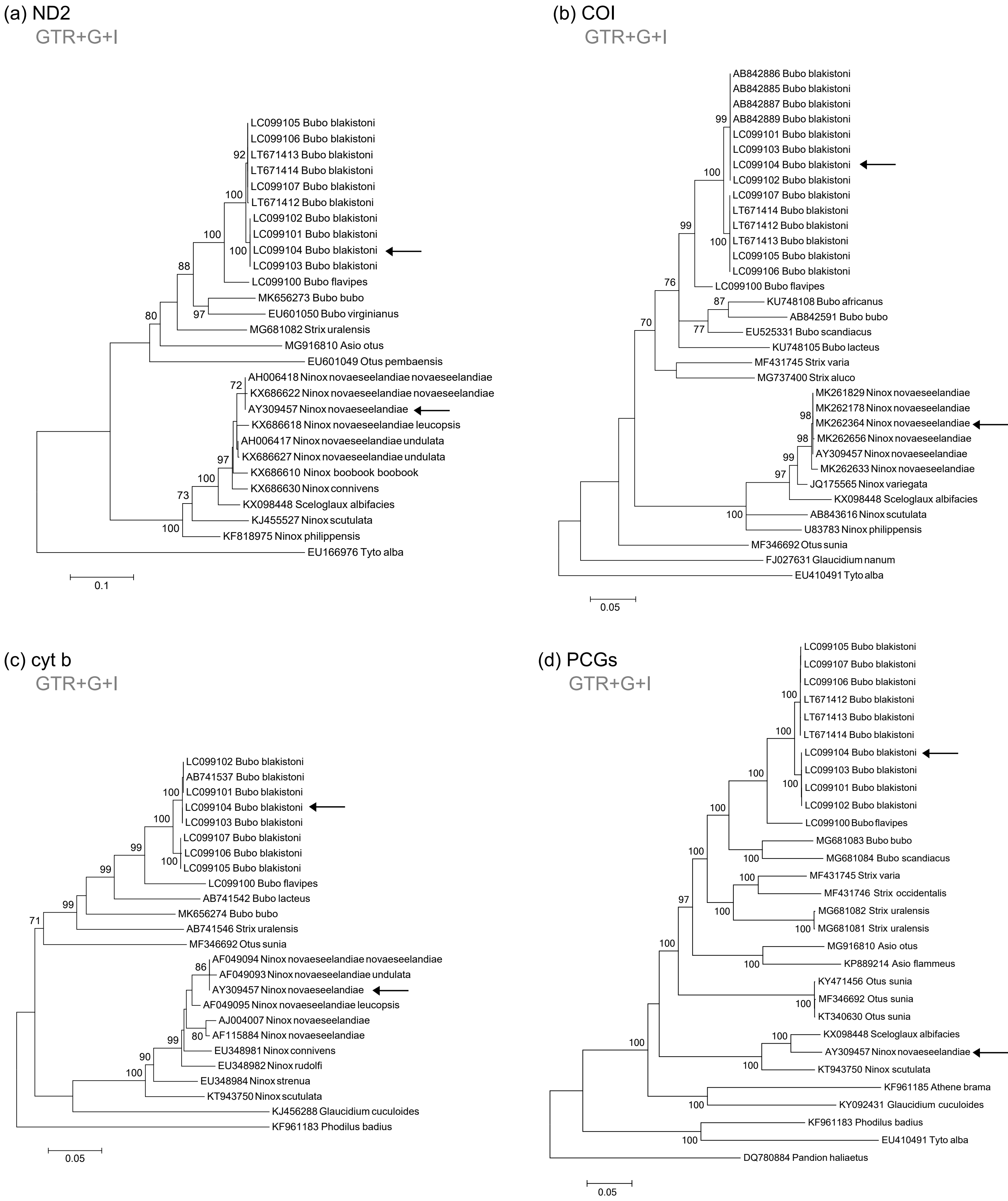

Figure S65. Maximum Likelihood phylogenies of *Ninox novaeseelandiae* (AY309457), *Bubo blakistoni* (LC099104) and related taxa based on mitochondrial sequences. Numbers at branches are bootstrap support values (>70%) based on 1000 replicates.

3. “*Sternula albifrons*” KT350612 (Park, C.E., Park, G.S., Jung, B.K., Park, Y.J., Kim, M.C., Park, H.C., Shin, J.H., unpublished; 2015)

Fig. S66-S67

Background
According to Spiridonova & Surmach (2018), KT350612, an unpublished mitogenomic sequence of Little Tern *Sternula albifrons* available on GenBank, is a chimera. No details were provided. The laboratory that produced mitogenome KT350612 also published a mitogenome sequence of the owl *Otus bakkamoena* (KT340631, Park et al. 2019b) that included fragments of *Sternula albifrons* (see **Appendix S2, Table S3**). Thus, we tested whether KT350612 represents a chimera, and specifically whether the sequence contained any *Otus* owl DNA fragments.

Methods
We conducted two tests:
(1) Phylogenetic analyses (ML) of cytochrome *b*, COI and ND2 to assess the phylogenetic position and branch length of KT350612 in comparison with other sequences of Charadriiformes, and two Strigidae (*Otus bakkamoena* and *Tyto alba*). This included multiple sequences of *Sternula albifrons*.
(2) Phylogenetic analyses (ML) of mitogenomic protein-coding gene (PCGs) sequences of KT350612 and other mitogenomes of terns (two *Sterna hirundo, Sterna paradisaea* and *Gelochelidon nilotica*), gulls, waders and owls (ND1, ND2, COI, COII, ATP8-ATP6-COIII, ND3, ND4, ND5, cytochrome *b* and ND6). The analysis was performed using *Anas formosa* (JF730435) as outgroup and with complete deletion of missing sites. No (other) mitogenomes of *Sternula* were available for comparison; however, if KT350612 is an authentic sequence of Little Tern then it should turn up in a single clade with other terns in all gene trees of individual PCGs.

Results
Our comparisons of KT350612 with cytochrome *b*, COI and ND2 sequences of Charadriiformes placed it consistently with *Sternula albifrons* (**Fig. S66**). Phylogenetic analysis of mitochondrial protein-coding genes invariably placed KT350612 in a clade with *Sterna hirundo, Sterna paradisaea* and *Gelochelidon nilotica* (**Fig. S67**). However, as expected, in several individual PCG trees (ND1, ND2, COI, COII, ATP8-ATP6-COIII, ND3), the chimeric *Otus bakkamoena* mitogenome was placed in the tern clade, as the sister to *S. albifrons*.

Conclusions
We conclude that KT350612 is a *bona fide* sequence of *S. albifrons* and that there is no evidence for it being a chimera. It is possible that the close similarity of portions of the *Otus bakkamoena* (KT340631) mitogenome to that of *S. albifrons* was interpreted by Spiridonova & Surmach (2018) as evidence that the *S. albifrons* mitogenome is a chimera.

References
Park, CE, Kim, MC, Ibal, JCP, Pham, HQ, Park, HC & Shin, JH 2019b. The complete mitochondrial genome sequence of *Otus bakkamoena* (Aves, Strigiformes, Strigidae). Mitochondrial DNA Part B 4: 775-776.
Spiridonova, LN & Surmach, SG 2018. Whole mitochondrial genome of Blakiston’s Fish Owl *Bubo* (*Ketupa*) *blakistoni* suggests its redescription in the genus *Ketupa*. Russian Journal of Genetics 54: 369-373.

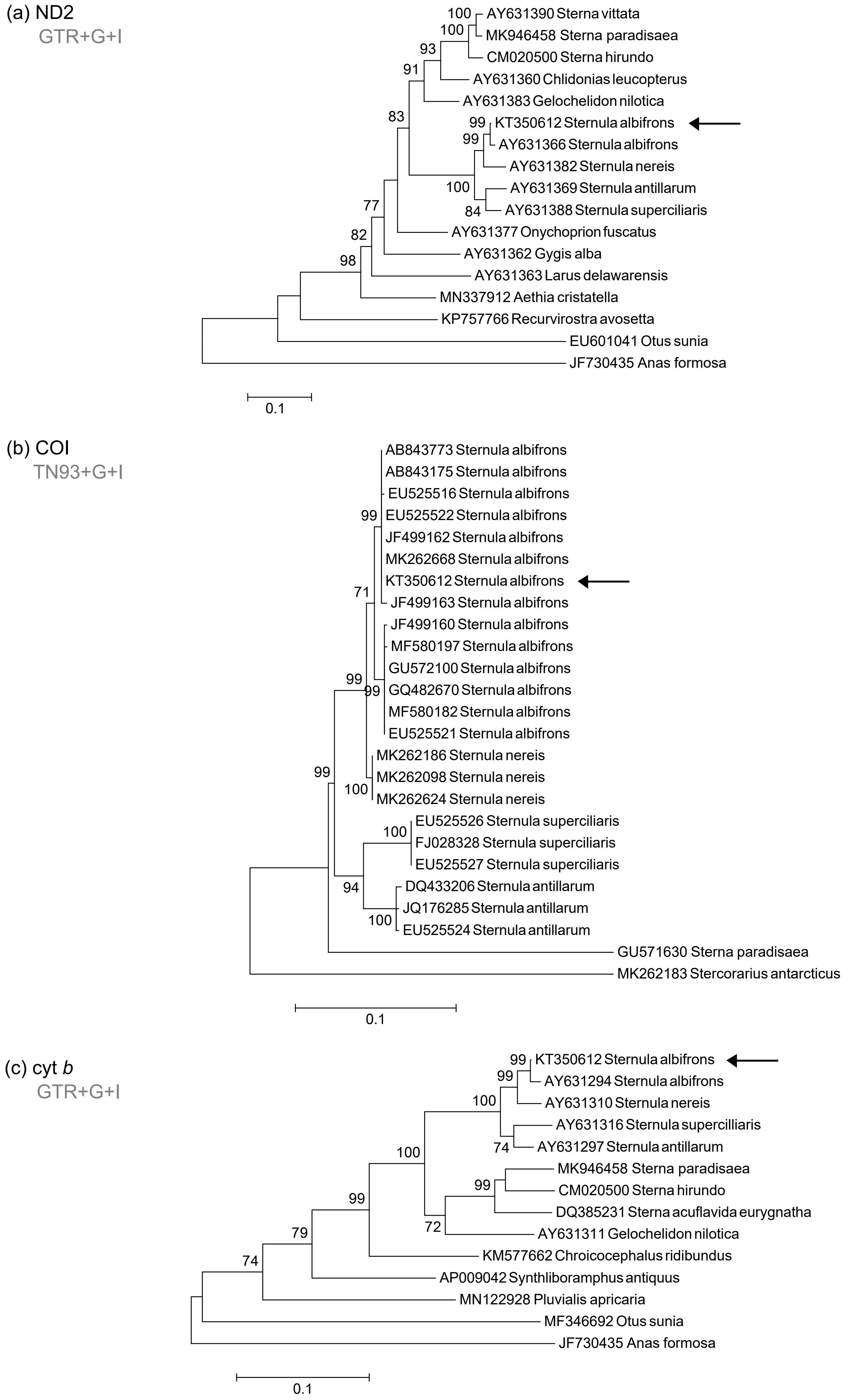

Fig. S66. Maximum Likelihood phylogenies of *Sternula albifrons* (KT350612) and related taxa based on three mitochondrial markers. Numbers at branches are bootstrap support values (>70%) based on 1000 replicates.

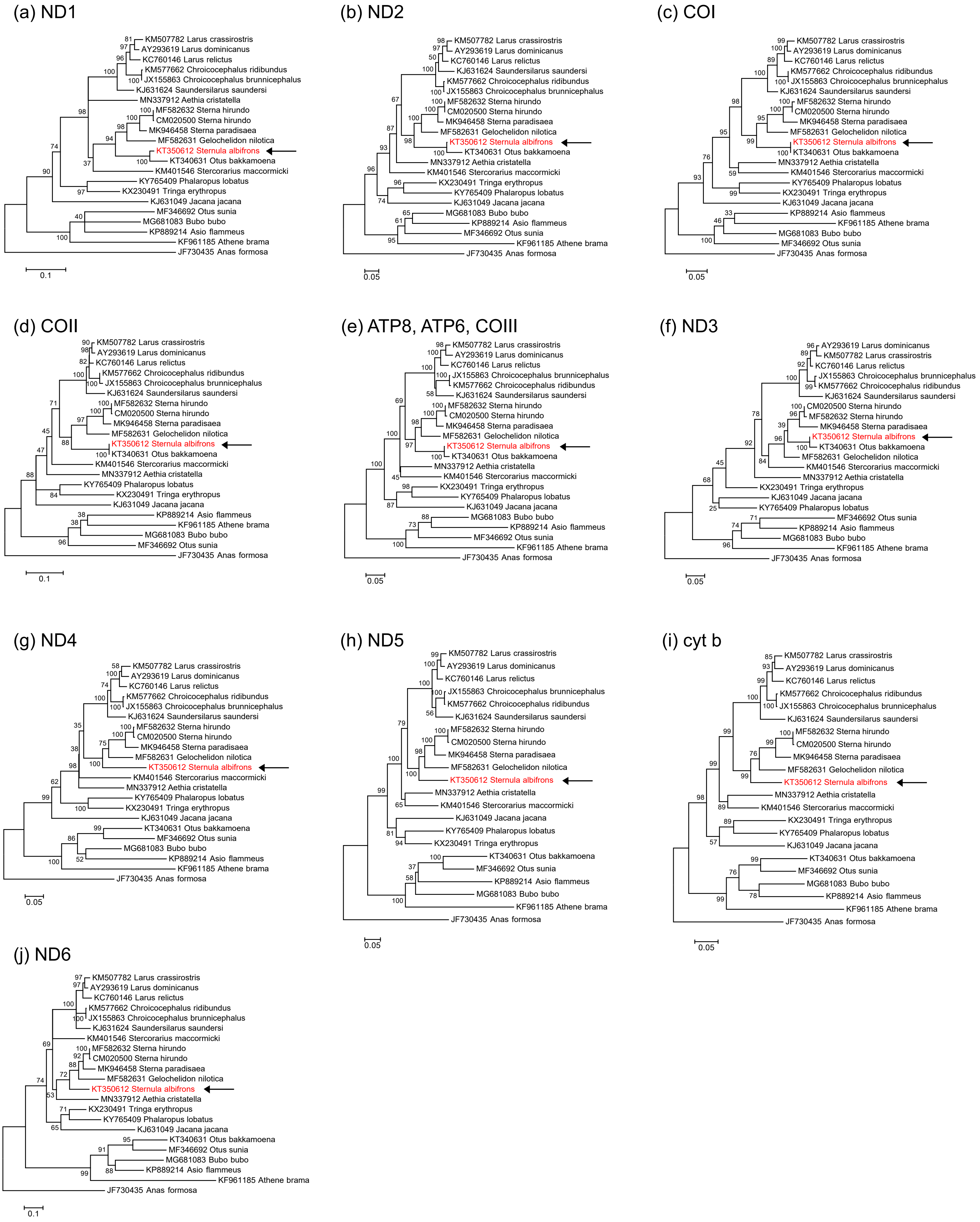

Fig. S67. Maximum Likelihood phylogenies of *Sternula albifrons* (KT350612) and related taxa based on twelve mitochondrial markers. Numbers at branches are bootstrap support values based on 1000 replicates. Note that in some phylogenies (a - f) a chimeric sequence of the owl *Otus bakkamoena* (KT340631) clusters with KT350612.
